# Checklist of British and Irish Hymenoptera - Ichneumonidae

**DOI:** 10.3897/BDJ.4.e9042

**Published:** 2016-07-05

**Authors:** Gavin R. Broad

**Affiliations:** ‡ The Natural History Museum, London, United Kingdom

**Keywords:** Britain, Ireland, fauna, Ichneumonoidea

## Abstract

**Background:**

The checklist of British and Irish Ichneumonidae is revised, based in large part on the collections of the Natural History Museum, London and the National Museums of Scotland, Edinburgh. Distribution records are provided at the country level.

**New information:**

Of the 2,447 species regarded as valid and certainly identified, 214 are here recorded for the first time from the British Isles. Neorhacodinae is considered to be a separate subfamily rather than a synonym of Tersilochinae. Echthrini is treated as a junior synonym of the tribe Cryptini, not Hemigastrini. *Echthrus* Gravenhorst and *Helcostizus* Förster are classified in Cryptini rather than, respectively, Hemigastrini and Phygadeuontini.

## Introduction

The Ichneumonidae is one of two families of the superfamily Ichneumonoidea, along with the Braconidae. Given the size of each family in Britain (over 1,300 braconids and nearly 2,500 ichneumonids) we are publishing the two checklists separately. This is one part of a series of papers revising the British and Irish list of Hymenoptera, that started with Broad (2014), Broad and Livermore (2014b), Broad and Livermore (2014a) and Liston et al. (2014). For the background and rationale behind these British Hymenoptera checklists see Broad (2014). The bases for the ichneumonid taxonomy are Yu and Horstmann (1997) and Taxapad (Yu et al. 2012) (ichneumonid data for both compiled by Klaus Horstmann). I have not relied on Yu et al. (2012) for data on species occurrence in Britain. Rather, I have relied mostly on the primary literature and collections, especially NMS and BMNH. Indeed, large parts of the checklist rely on original work, identifying specimens in these collections. The collections of the NMS have been invaluable in updating this checklist as Mark Shaw has very actively encouraged taxonomists to use the collections, which contain a large amount of recently collected, often reared, material. The late J.F. Perkins also did a huge amount to improve the BMNH collection of Ichneumonidae that was not really reflected in his published output. I have made great use of Perkins’s identifications as well as Horstmann’s, Andrey Khalaim’s and many other workers, including my own. Many Irish records are taken from O'Connor et al. (2007).

All additions to and deletions from the British list since Fitton (1978) are recorded as well as country-level distribution within the British Isles (but regarding the Republic of Ireland and Northern Ireland as one geographical unit), i.e. England, Scotland, Wales, Ireland, Isle of Man. The current numbers of valid, certainly identified ichneumonid species are listed per subfamily and by country in Table 1. This highlights the lack of data from countries other than England. Because the ichneumonid literature is extensive and scattered I also provide many taxonomic references. Some changes to classification are employed here, following molecular phylogenetic studies (Laurenne et al. 2006, Quicke et al. 2009). Two genera that are usually classified in Hemigastrini and Phygadeuontini - *Echthrus* Gravenhorst and *Helcostizus* Förster - are transferred to Cryptini (as originally suggested by Laurenne et al. 2006), which means that Echthrini Narayanan & Lal, 1958, becomes a junior synonym of Cryptini Kirby, 1837. And, contrary to Quicke et al. (2009), Neorhacodinae is again regarded as a valid subfamily, not as a synonym of Tersilochinae, following phylogenetic work by A.M.R. Bennett *et al.* (in prep.) and reflecting differences in host use.

Figs 1, 2, 3, 4, 5 illustrate a tiny part of the diversity of Ichneumonidae. A handbook to the British fauna (Broad, Shaw & Fitton, in prep.) provides an introduction to the biology, classification and identification of this family of parasitoid wasps and will be published soon.

## Materials and methods

For a more detailed description of the background and rationale to the Hymenoptera checklist, see Broad (2014). I provide rather extensive Palaearctic synonymy and citations because if you do not have access to the Taxapad database (Yu et al. 2012) it can be very difficult to trace the fate of names in the voluminous and scattered literature, although some of the functionality of the catalogue is available online. Conventions and abbreviations are listed below.

[***species***] taxon deleted from the British and Irish list

BMNH Natural History Museum, London

NMS National Museums of Scotland, Edinburgh

UM Ulster Museum [J. Brock collection]

# known introductions occurring only under artificial conditions

? status (including uncertain synonymy) or identification in the British Isles uncertain

misident. has been misidentified as this name

nom. dub. *nomen dubium*, a name of doubtful status

nom. ob. *nomen oblitum*, ‘forgotten name’, does not have priority over a younger name

nom. nov. *nomen novum*, a replacement name

nom. nud. *nomen nudum*, an unavailable name, with no type specimen

preocc. name preoccupied (junior homonym)

stat. rev. *status revocatus*, revived status (e.g., raised from synonymy)

unavailable name unavailable under provisions of the ICZN code

var. variety, only available as a valid name under certain provisions of the ICZN code

When there are no countries listed for a species, there are two explanations. First, the species has been carried over from the previous checklist (Fitton 1978) and, although I have not seen any British specimens I have no reason to doubt the original identification. Second, specimens in collections are labelled with imprecise locality data, such as 'Great Britain'. This is the case with many of the older collections.

Alternative versions of the checklist can be downloaded here as a Word document or Excel spreadsheet under supplementary materials: Suppl. materials 1, 2. The British and Irish ichneumonid checklist, together with the entire Hymenoptera checklist, will be kept up to date in a Scratchpad, Hymenoptera of the British Isles.

## Checklists

### Acaenitinae

#### ACAENITINAE

Förster, 1869

##### Notes

Tribes within Acaenitinae (formerly Acaenitini and Coleocentrini) were abandoned by Wahl and Gauld (1998). Distribution data from Fitton (1981) and Shaw and Wahl (1989), with further references given.

#### Acaenitus

Latreille, 1809

ACOENITES Latreille, 1810
ACOENITUS Griffith, 1832

#### Acaenitus dubitator

(Panzer, 1800)

Ichneumon dubitator Panzer, 1800

##### Distribution

Scotland

#### Arotes

Gravenhorst, 1829

ASTHENOMERIS Förster, 1869
SPHALERUS Kriechbaumer, 1878

##### Notes

*annulicornis* synonymised by Varga (2013)

#### Arotes albicinctus

Gravenhorst, 1829

bifasciatus (Kriechbaumer, 1878, *Sphalerus*)
annulicornis Kriechbaumer, 1894

##### Distribution

England

#### Coleocentrus

Gravenhorst, 1829

MACROCOLEUS Desvignes, 1850

#### Coleocentrus croceicornis

(Gravenhorst, 1829)

Macrus croceicornis Gravenhorst, 1829

##### Distribution

England

##### Notes

*Coleocentrus soleatus* (Gravenhorst, 1829, *Macrus*) was removed from synonymy by Kasparyan and Khalaim (2007a).

#### Coleocentrus excitator

(Poda, 1761)

Ichneumon excitator Poda, 1761
segmentator (Fabricius, 1793, *Ichneumon*)
gigantor (Thunberg, 1824, *Ichneumon*)
longiventris (Gravenhorst, 1829, *Macrus*)
segmentatrix (Schulz, 1906, *Lissonota*)

##### Distribution

Scotland

##### Notes

added by Shaw (1986)

#### Leptacoenites

Strobl, 1902

#### Leptacoenites notabilis

(Desvignes, 1856)

Lampronota notabilis Desvignes, 1856
frauenfeldi (Tschek, 1869, *Lissonota*)
marginatus (Kriechbaumer, 1899, *Heterolabis*)
petiolaris (Kriechbaumer, 1899, *Heterolabis*)
tscheki (Strobl, 1902, *Procinetus*)

#### Phaenolobus

Förster, 1869

CHORISCHIZUS Förster, 1869
MOLDACOENITUS Constantineanu & Constantineanu, 1968

#### Phaenolobus terebrator

(Scopoli, 1763)

Ichneumon terebrator Scopoli, 1763
arator (Rossi, 1790, *Ichneumon*)

##### Distribution

England

### Adelognathinae

#### ADELOGNATHINAE

Thomson, 1888

##### Notes

Distribution data from Fitton et al. (1982)supplemented by the collections of NMS, with further references given. Note that, according to the molecular phylogenetic results of Laurenne et al. (2006), *Adelognathus* may be an aberrant genus of Cryptinae.

#### Adelognathus

Holmgren, 1857

PAMMICRA Förster, 1869
NOTOMERIS Förster, 1869
CNEMISCHYS Förster, 1869
EPITROPUS Rossem, 1990

##### Notes

*Epitropus* synonymised by Broad (2004)

#### Adelognathus acantholydae

Kasparyan, 1986

##### Distribution

Scotland

##### Notes

NMS, det. Shaw, added here

#### Adelognathus brevicornis

Holmgren, 1857

limbatus Thomson, 1888
montivagator Aubert, 1976

##### Distribution

England, Scotland, Ireland

##### Notes

The *chrysopygus* (Grav.) referred to by Fitton et al. (1982) actually refers to *punctulatus* Thoms.; *chrysopygus* is the correct name for the species referred to as *granulatus* Perkins by Fitton et al. (1982) (Kasparyan 1986). Some distribution data from Bennett et al. (2002).

#### Adelognathus britannicus

Perkins, 1943

##### Distribution

England

#### Adelognathus chrysopygus

(Gravenhorst, 1829)

Hemiteles chrysopygus Gravenhorst, 1829
granulatus Perkins, 1943

##### Distribution

England, Scotland, Ireland, Isle of Man

#### Adelognathus difformis

Holmgren, 1857

##### Distribution

England

##### Notes

NMS, det. Shaw, added here

#### Adelognathus dorsalis

(Gravenhorst, 1829)

Hemiteles dorsalis Gravenhorst, 1829
melanius Roman, 1918
insolitus (Rossem, 1990, *Epitropus*)

##### Distribution

England, Scotland, Wales, Ireland, Isle of Man

##### Notes

*insolitus* synonymised by Broad (2004)

#### Adelognathus laevicollis

Thomson, 1883

##### Distribution

England, Ireland

#### Adelognathus leucotrochi

Shaw & Wahl, 2014

##### Distribution

England, Scotland

##### Notes

added by Shaw and Wahl (2014)

#### Adelognathus nigriceps

Thomson, 1888

##### Distribution

England, Wales

##### Notes

some distribution data from Askew (2000)

#### Adelognathus nigrifrons

Holmgren, 1857

##### Distribution

England, Ireland

##### Notes

added by Fitton et al. (1982)

#### Adelognathus obscurus

Kasparyan, 1986

##### Distribution

England, Scotland

##### Notes

added by Kasparyan (1990)

#### Adelognathus pallipes

(Gravenhorst, 1829)

Plectiscus pallipes Gravenhorst, 1829
ruthei Holmgren, 1857
pallidipes (Marshall, 1872, *Plectiscus*)

##### Distribution

England, Scotland, Ireland

#### Adelognathus pilosus

Thomson, 1888

##### Distribution

England

#### Adelognathus punctulatus

Thomson, 1883

chrysopygus misident.
pallipes Holmgren, 1857 preocc.
dimidiatus Thomson, 1888
pallidipes Dalla Torre, 1901

##### Distribution

England, Ireland

#### Adelognathus pusillus

Holmgren, 1857

##### Distribution

England, Wales, Ireland

#### Adelognathus stelfoxi

Fitton, Gauld & Shaw, 1982

##### Distribution

England, Scotland, Wales, Ireland

##### Notes

added by Fitton et al. (1982); Ely (2001)

#### Adelognathus tenthredinarum

(Giraud, 1872)

Plectiscus tenthredinarum Giraud, 1872
nigricornis Thomson, 1888

##### Distribution

England

#### Adelognathus tetratinctorius

(Thunberg, 1824)

Ichneumon tetratinctorius Thunberg, 1824
fasciatus Thomson, 1883
scabriculus Thomson, 1883

##### Distribution

England, Ireland

#### Adelognathus thomsoni

Schmiedeknecht, 1911

thuringiacus Schmiedeknecht, 1911

##### Distribution

England, Ireland

### Agriotypinae

#### AGRIOTYPINAE

Haliday, 1838

##### Notes

Distribution data from Bennett (2001), Howe and Howe (2005) and the collections of the NMS.

#### Agriotypus

Curtis, 1832

CROTOPUS Holmgren, 1859
ATOPOTYPUS Chao, 1992

##### Notes

*Atopotypus* synonymised by Bennett (2001)

#### Agriotypus armatus

Curtis, 1832

abnormis (Holmgren, 1859, *Crotopus*)

##### Distribution

England, Scotland, Wales

### Alomyinae

#### ALOMYINAE

Förster, 1869

##### Notes

*Alomya* has frequently been placed in its own subfamily, Alomyinae (e.g. Perkins 1959), or in a separate tribe within the Ichneumoninae (Diller 1981, Selfa and Diller 1994) but a close relationship with *Colpognathus* and *Centeterus* was proposed by Wahl and Mason (1995), with the result that the ichneumonine tribe here called Phaeogenini took the name Alomyini. Based on the molecular phylogenetic results of Laurenne et al. (2006) *Alomya* and *Megalomya* Uchida are considered here to comprise a separate subfamily.

#### Alomya

Panzer, 1806

ALOMYIA misspelling
HALOMYA Billberg, 1820

##### Notes

Distribution data from Perkins (1952), Perkins (1959) and the NMS.

#### Alomya debellator

(Fabricius, 1775)

Ichneumon debellator Fabricius, 1775
fischeri (Schrank, 1776, *Ichneumon*)
trituberculata (Gmelin, 1790, *Ichneumon*)
ovator (Fabricius, 1793, *Ichneumon*)
victor Curtis, 1826
nigra Gravenhorst, 1829
debellatrix Schulz, 1906
victrix Schulz, 1906
silvicola Ulbricht, 1909 unavailable

##### Distribution

England, Scotland, Ireland, Isle of Man

#### Alomya semiflava

Stephens, 1835

minor Ulbricht, 1909 unvailable
minor Ulbricht, 1911 preocc.

##### Distribution

England

### Anomaloninae

#### ANOMALONINAE

Viereck, 1918

ANOMALINAE misspelling

##### Notes

Distribution data mostly taken from the collections of the NMS and BMNH, supplemented by Gauld and Mitchell (1977a), unless noted otherwise. Most of the NMS and some of the BMNH material has been identified recently by H. Schnee.

#### ANOMALONINI

Viereck, 1918

ANOMALINI misspelling

#### Anomalon

Panzer, 1804

TRACHYNOTUS Gravenhorst, 1829 preocc.
OCHLERUS Gistel, 1848 preocc.
NOTOTRACHYS Marshall, 1872
ANOMALUM Schulz, 1906
TRACHYOPTERUS Morley, 1912
PSEUDONOTOTRACHYS Meyer, 1930
MICROCREMASTUS Hedwig, 1961

#### Anomalon cruentatum

(Geoffroy, 1785)

Ichneumon cruentatus Geoffroy, 1785
petiolatum (Geoffroy, 1785, *Ichneumon*)
foliator (Fabricius, 1798, *Ophion*)
cruentatum Panzer, 1804
humerale (Brullé, 1832, *Trachynotus*)
epiphanii Izquierdo, 1977

##### Distribution

England

#### GRAVENHORSTIINI

Enderlein, 1912

THERIONINI Viereck, 1918

##### Notes

Species of Gravenhorstiini excluded from the British and Irish list:

[PERISPHINCTER Townes, 1961 *brevicollis* (Wesmael, 1849, *Anomalon*)] Gauld and Mitchell (1977a) recorded one specimen of *brevicollis* from Killiecrankie, Scotland, as a species of *Agrypon*. As this species is now included in *Perisphincter* (Schnee 1978), a genus which Gauld knew at the time, and the specimen is lost (M.R. Shaw, pers. comm.), this is considered here to be a misidentification.

#### Agrypon

Förster, 1860

AGRYPUM Schulz, 1906

##### Notes

much synonymy from Schnee (2008)

species of *Agrypon* excluded from the British and Irish list:

[*interruptus* (Desvignes, 1856, *Anomalon*)] Listed as a doubtfully placed species of *Agrypon* by Fitton (1978), but Gauld and Mitchell (1977b) had established that this species is actually an exotic cremastine. Currently listed in Yu and Horstmann (1997) as a doubtfully placed species of *Cremastus*.

#### Agrypon anomelas

(Gravenhorst, 1829)

Anomalon anomelas Gravenhorst, 1829
anomalas misspelling
furtivum Förster, 1860
trochanteratum (Holmgren, 1860, *Anomalon*)
rufipes Kiss, 1926

##### Distribution

England

##### Notes

George (1978) provides information on its occurrence in Britain

#### Agrypon anxium

(Wesmael, 1849)

Anomalon anxium Wesmael, 1849
pictum Kiss, 1924

##### Distribution

England, Scotland, Wales, Ireland

#### Agrypon batis

(Ratzeburg, 1855)

Anomalon batis Ratzeburg, 1855
serpentinum Förster, 1860
stenostigma (Thomson, 1892, *Anomalon*)
segne (Tosquinet, 1896, *Anomalon*)

##### Distribution

England, Scotland, Ireland

##### Notes

NMS, BMNH, det. Schnee, added here

#### Agrypon brachycerum

Hellén, 1950

##### Distribution

England, Scotland

##### Notes

NMS, BMNH, det. Schnee, added here. Listed as a synonym of *anxium* in Yu and Horstmann (1997).

#### Agrypon canaliculatum

(Ratzeburg, 1844)

Anomalon canaliculatum Ratzeburg, 1844

##### Distribution

England, Scotland

##### Notes

NMS, BMNH, det. Schnee, added here. Misidentified *canaliculatum* of authors is *Habronyx nigricornis* (Wesm.).

#### Agrypon clandestinum

(Gravenhorst, 1829)

Anomalon clandestinum Gravenhorst, 1829
delarvatum misident.
capillosum (Hartig, 1838, *Anomalon*)
affine (Holmgren, 1857, *Anomalon*)
brachypterum Förster, 1860
clandestinum Förster, 1860 preocc.
ruficoxis (Szépligeti, 1899, *Labrorychus*)
flavopunctatum (Kiss, 1933, *Blaptocampus*)

##### Distribution

England, Scotland, Ireland, Isle of Man

#### Agrypon flaveolatum

(Gravenhorst, 1807)

Ophion flaveolatum Gravenhorst, 1807
cribrator (Thunberg, 1824, *Ichneumon*)
laedator (Thunberg, 1824, *Ichneumon*)
arquatum (Gravenhorst, 1829, *Anomalon*)
septentrionale (Holmgren, 1857, *Anomalon*)
aggressorium Förster, 1860
confusum Förster, 1860
elegantulum Förster, 1860
rubricatum Förster, 1860
rubricatum (Förster, 1878, *Atrometus*) preocc.

##### Distribution

England, Scotland, Wales, Ireland

#### Agrypon flexorium

(Thunberg, 1824)

Ichneumon flexorium Thunberg, 1824
tenuicorne (Gravenhorst, 1829, *Anomalon*)
subclavatum Förster, 1860
anaitidis (Szépligeti, 1899, *Labrorychus*)
sibiricum (Shestakov, 1923, *Labrorychus*)

##### Distribution

England, Scotland, Wales, Ireland

##### Notes

*Agrypon polyxenae* (Szépligeti, 1899, *Labrorychus*) removed from synonymy by Schnee (2008).

#### Agrypon gracilipes

(Curtis, 1839)

Therion gracilipes Curtis, 1839
debile (Wesmael, 1849, *Anomalon*)
meridionator Aubert, 1964 preocc.

##### Distribution

England, Wales

#### Agrypon interstitiale

Schnee, 1989

##### Distribution

England, Scotland

##### Notes

NMS, BMNH, det. Schnee, added here

#### Agrypon minutum

(Bridgman & Fitch, 1884)

Anomalon minutum Bridgman & Fitch, 1884
minutum (Bridgman, 1884, *Anomalon*) preocc.

##### Distribution

England, Scotland, Wales

##### Notes

Listed as a synonym of *anxium* (Wesm.) in Yu and Horstmann (1997), treated by Schnee as a valid species, but unpublished.

#### Agrypon rugifer

(Thomson, 1894)

Anomalon rugifer Thomson, 1894

##### Distribution

Wales

##### Notes

NMS, det. Schnee, added here

#### Agrypon varitarsum

(Wesmael, 1849)

Anomalon varitarsum Wesmael, 1849
cognatum Förster, 1860
nigripes (Bridgman, 1887, *Anomalon*)
variitarsum Dalla Torre, 1901

##### Distribution

England, Scotland, Ireland

#### Agrypon sp. H

##### Distribution

England, Wales

##### Notes

NMS, BMNH, det. Schnee, added here

#### Aphanistes

Förster, 1869

ANOCHILACRUM Enderlein, 1921

#### Aphanistes bellicosus

(Wesmael, 1849)

Anomalon bellicosum Wesmael, 1849

##### Distribution

England

##### Notes

NMS, det. Schnee, added here

#### Aphanistes gliscens

(Hartig, 1838)

Anomalon gliscens Hartig, 1838
bellicosus misident.
xanthopus misident.
armatus (Wesmael, 1849, *Anomalon*)

##### Distribution

England, Scotland, Wales

#### Aphanistes ruficornis

(Gravenhorst, 1829)

Anomalon ruficorne Gravenhorst, 1829
excavatus (Ratzeburg, 1848, *Anomalon*)
wesmaeli (Holmgren, 1856, *Anomalon*)

##### Distribution

England, Scotland, Wales, Ireland

#### Atrometus

Förster, 1869

##### Notes

synonymy follows Schnee (2008)

#### Atrometus insignis

Förster, 1878

rubricatus Förster, 1878
trachynotus (Brauns, 1895, *Anomalon*)
melanosoma Szépligeti, 1899
pulchellator Aubert, 1971

##### Notes

No British specimen could be located in the depositories cited by Gauld and Mitchell (1977b) (M.R. Shaw, pers comm.).

#### Barylypa

Förster, 1869

LAPHYCTES Förster, 1869
SARNTHEINIA Dalla Torre, 1901
HADROMANUS Szépligeti, 1905
MAGNIBUCCA Morley, 1913
TROCHISCOMERUS Meyer, 1931

##### Notes

some synonymy from Schnee (2008)

#### Barylypa delictor

(Thunberg, 1824)

Ichneumon delictor Thunberg, 1824
perspicillator (Gravenhorst, 1829, *Anomalon*)
affinis (Lucas, 1849, *Anomalon*)
menyanthidis (Boie, 1855, *Anomalon*)
mesozona (Förster, 1878, *Laphyctes*)
genalis (Thomson, 1892, *Anomalon*)
frisiaca Habermehl, 1922
temporalis Meyer, 1935

##### Distribution

England, Scotland, Wales

#### Barylypa propugnator

(Förster, 1855)

Anomalon propugnator Förster, 1855
insidiator (Förster, 1878, *Laphyctes*)
carinata (Brischke, 1880, *Anomalon*)
cylindrica (Bridgman & Fitch, 1884, *Anomalon*)
cylindrica (Bridgman, 1884, *Anomalon*) preocc.
rufa (Habermehl, 1920, *Anomalon*) preocc.

##### Distribution

England, Wales, Ireland

##### Notes

Listed as a species of *Erigorgus* in Yu and Horstmann (1997), transferred to *Barylypa* by Schnee (2008).

#### Barylypa rubricator

(Szépligeti, 1899)

Laphyctes rubricator Szépligeti, 1899
rubricatrix (Schulz, 1906, *Sarntheinia*)
rossica Meyer, 1935

##### Distribution

England

##### Notes

BMNH, Hunterian, det. Schnee, added here

#### Barylypa uniguttata

(Gravenhorst, 1829)

Anomalon uniguttatum Gravenhorst, 1829

##### Distribution

England

##### Notes

Although Gauld and Mitchell (1977b) record *uniguttata* as British on the basis of one specimen from Norfolk, no British specimen could be located in the depositories cited by Gauld & Mitchell (M.R. Shaw, pers comm.); Gauld was not aware at the time of the presence of *rubricator* in Britain.

#### Erigorgus

Förster, 1869

SYMPRATIS Förster, 1869

##### Notes

Although Gauld (1976b), Gauld (1997) regarded *Erigorgus* as a subgenus of *Gravenhorstia*, most recent works regard it as a separate genus, a position followed by Yu and Horstmann (1997). As the relative merits of genus versus subgenus are rather subjective I have followed the majority opinion.

#### Erigorgus cerinops

(Gravenhorst, 1829)

Anomalon cerinops Gravenhorst, 1829
flavifrons (Gravenhorst, 1807, *Ophion*) preocc.
xantha (Boie, 1855, *Anomalon*)
facialis (Boie, 1857, *Campoplex*)
lapponicus (Thomson, 1892, *Anomalon*)
rufofemoralis (Schmiedeknecht, 1936, *Anomalon*)

##### Distribution

England, Scotland, Wales, Ireland

#### Erigorgus fibulator

(Gravenhorst, 1829)

Anomalon fibulator Gravenhorst, 1829
claripennis (Thomson, 1892, *Anomalon*)

##### Distribution

England, Wales

##### Notes

BMNH, det. Schnee, added here

#### Erigorgus foersteri

(Mocsáry, 1897)

Anomalon foersteri Mocsáry, 1897
melanops misident.
brevicorne (Förster, 1855, *Anomalon*) preocc.

##### Notes

NMS, det. Schnee, added here; reared by M.R. Shaw: Bradwell Village, Burford, Oxon. The name *foersteri* was overlooked by Yu and Horstmann (1997) and other authors (Schnee 2008).

#### Erigorgus melanops

(Förster, 1855)

Anomalon melanops Förster, 1855
melanobata misident.
varians (Brauns, 1895, *Anomalon*)
flavimana (Szépligeti, 1899, *Erigorgus*)
interstitialis (Szépligeti, 1899, *Erigorgus*)
similis (Szépligeti, 1899, *Erigorgus*)
purpuratae (Kriechbaumer, 1900, *Erigorgus*)

##### Distribution

England, Scotland

#### Erigorgus procerus

(Gravenhorst, 1829)

Anomalon procerum Gravenhorst, 1829

##### Distribution

England

##### Notes

BMNH, det. Schnee, added here; various authors included *procerus* as a British species but Gauld and Mitchell (1977b) did not see any British or Irish material and there were no such specimens in BMNH or NMS at that time.

#### Erigorgus varicornis

(Thomson, 1894)

Anomalon varicorne Thomson, 1894

##### Distribution

England, Scotland

##### Notes

BMNH, det Schnee, added here. Listed as a synonym of *propugnator* (Förster) in Yu and Horstmann (1997).

#### Gravenhorstia

Boie, 1836

ODONTOPSIS Förster, 1869

#### Gravenhorstia picta

Boie, 1836

fasciata (Giraud, 1857, *Anomalon*)
fasciata (Marshall, 1873, *Anomalon*) preocc.
septemfasciata (Taschenberg, 1875, *Ophion*)
picta (Rudow, 1882, *Anomalon*)

##### Distribution

England

#### Habrocampulum

Gauld, 1976

#### Habrocampulum biguttatum

(Gravenhorst, 1829)

Anomalon biguttatum Gravenhorst, 1829

##### Distribution

England

#### Habronyx

Förster, 1869

#### Camposcopus

Förster, 1869

LABRORYCHUS Förster, 1869
BLAPTOCAMPUS Thomson, 1892

##### Notes

species of Habronyx (Camposcopus) deleted from the British and Irish list:

[*perspicuus* (Wesmael, 1849, *Anomalon*)] Recorded by Gauld and Mitchell (1977b) but there are no British specimens in BMNH or NMS and the identification must be regarded as doubtful.

#### Habronyx (Camposcopus) nigricornis

(Wesmael, 1849)

Anomalon nigricorne Wesmael, 1849
canaliculatus misident.
melanomerus (Förster, 1860, *Agrypon*)
maidan (Shestakov, 1923, *Blaptocampus*)

##### Distribution

England, Scotland, Wales, Ireland

#### Habronyx

Förster, 1869

ACANTHOSTOMA Kriechbaumer, 1895
MACROSTEMMA Shestakov, 1923

#### Habronyx (Habronyx) heros

(Wesmael, 1849)

Anomalon heros Wesmael, 1849
mirabilis (Desvignes, 1856, *Anomalon*)
gravenhorstii Förster, 1860
gigas (Kriechbaumer, 1880, *Anomalon*)
oti (Kriechbaumer, 1895, *Anomalon*)

##### Distribution

England

#### Heteropelma

Wesmael, 1849

SCHIZOLOMA Wesmael, 1849
SCHIZOPOMA Förster, 1869

#### Heteropelma amictum

(Fabricius, 1775)

Ichneumon amictus Fabricius, 1775
xanthopus (Schrank, 1781, *Ichneumon*)
amictor (Thunberg, 1824, *Ichneumon*)
xanthopor (Thunberg, 1824, *Ichneumon*)
capitatum (Desvignes, 1856, *Anomalon*)
bucephalum (Vollenhoven, 1858, *Anomalon*)
bucephalum (Brauns, 1898, *Schizoloma*) preocc.

##### Distribution

England, Scotland, Ireland, Isle of Man

##### Notes

distribution data from Gauld (1976a)

#### Heteropelma megarthrum

(Ratzeburg, 1848)

Anomalon megarthrum Ratzeburg, 1848
calcator Wesmael, 1849
scabridum (Boie, 1855, *Anomalon*)
megalarthrum (Schulz, 1906, *Anomalon*)
nigriscutum (Fahringer, 1941, *Anomalon*)

##### Distribution

England, Scotland, Ireland

#### Parania

Morley, 1913

#### Parania geniculata

(Holmgren, 1857)

Anomalon geniculatum Holmgren, 1857

##### Distribution

England

#### Therion

Curtis, 1829

THERIUM Agassiz, 1846
EXOCHILUM Wesmael, 1849

#### Therion circumflexum

(Linnaeus, 1758)

Ichneumon circumflexus Linnaeus, 1758
brevicorne misident.
ramidulum (Christ, 1791, *Ichneumon*)
unicolor (Ratzeburg, 1844, *Anomalon*)
callosum (Shestakov, 1923, *Exochilum*)
curticorne Bauer, 1967

##### Distribution

England, Scotland, Wales, Ireland

##### Notes

Specimens identified as *brevicorne* (Gauld and Mitchell 1977b) are misidentifications of *circumflexum* (, and pers. comm. to M.R. Shaw). 

#### Trichomma

Wesmael, 1849

TRICHOMELLA Szépligeti, 1910

#### Trichomma enecator

(Rossi, 1790)

Ichneumon enecator Rossi, 1790
ruficoxis Förster, 1860

##### Distribution

England, Isle of Man

#### Trichomma fulvidens

Wesmael, 1849

bituberculatum Schmiedeknecht, 1902

##### Distribution

England

#### Trichomma intermedium

Krieger, 1904

##### Distribution

England

#### Trichomma occisor

Habermehl, 1909

##### Distribution

England

### Banchinae

#### BANCHINAE

Wesmael, 1845

LISSONOTINAE Förster, 1869

##### Notes

Taxonomy and distribution data follow Brock’s (in prep.) handbook to the British Banchinae, with some additions from NMS, BMNH, Aubert (1978) and Fitton (1976). Additional references are given.

#### ATROPHINI

Seyrig, 1932

LISSONOTINI Förster, 1869

#### Alloplasta

Förster, 1869

ASYMMICTUS Förster, 1869
TRYSICAMPE Förster, 1869

#### Alloplasta piceator

(Thunberg, 1824)

Ichneumon piceator Thunberg, 1824
creditor (Thunberg, 1824, *Ichneumon*)
albitarsus (Gravenhorst, 1829, *Exetastes*)
lata (Gravenhorst, 1829, *Exetastes*)
murina (Gravenhorst, 1829, *Lissonota*)
murina (Gravenhorst, 1829, *Tryphon*)
albitarsoria (Zetterstedt, 1838, *Tryphon*)
genucincta (Rudow, 1886, *Cryptus*)
variipes (Szépligeti, 1899, *Meniscus*)

##### Distribution

England, Scotland, Ireland

#### Alloplasta plantaria

(Gravenhorst, 1829)

Phytodietus plantarius Gravenhorst, 1829

##### Distribution

England, Wales

#### Arenetra

Holmgren, 1859

LASIOPS Holmgren, 1856 preocc.

#### Arenetra pilosella

(Gravenhorst, 1829)

Tryphon pilosellus Gravenhorst, 1829

##### Distribution

England, Scotland

#### Cryptopimpla

Taschenberg, 1863

APHANODON Förster, 1869
XENACIS Förster, 1869
XENOCORNIA Schmiedeknecht, 1900

#### Cryptopimpla altipes

(Holmgren, 1860)

Lissonota altipes Holmgren, 1860

##### Distribution

England, Scotland

##### Notes

added by Brock (in prep.). Removed from synomymy with *quadrilineata* by Brock (in prep.).

#### Cryptopimpla anomala

(Holmgren, 1860)

Lissonota anomala Holmgren, 1860

##### Distribution

England, Scotland, Wales

##### Notes

added by Brock (in prep.)

#### Cryptopimpla arvicola

(Gravenhorst, 1829)

Lissonota arvicola Gravenhorst, 1829
brachycentra (Gravenhorst, 1829, *Lissonota*)
kaisdii (Kiss, 1929, *Arenetra*)

##### Distribution

England

#### Cryptopimpla calceolata

(Gravenhorst, 1829)

Phytodietus calceolatus Gravenhorst, 1829
leptogaster (Holmgren, 1860, *Lissonota*)

##### Distribution

England, Scotland

#### Cryptopimpla caligata

(Gravenhorst, 1829)

Lissonota caligata Gravenhorst, 1829

##### Distribution

England, Ireland

#### Cryptopimpla errabunda

(Gravenhorst, 1829)

Phytodietus errabundus Gravenhorst, 1829

##### Distribution

England, Ireland

#### Cryptopimpla hertrichi

Heinrich, 1952

##### Distribution

England, Scotland

##### Notes

added by Brock (in prep.)

#### Cryptopimpla quadrilineata

(Gravenhorst, 1829)

Tryphon quadrilineatus Gravenhorst, 1829
blanda (Gravenhorst, 1829, *Phytodietus*)
hungarica (Szépligeti, 1899, *Xenacis*)
vaga (Szépligeti, 1899, *Lissonota*)

##### Distribution

England, Wales

#### Lissonota

Gravenhorst, 1829

LAMPRONOTA Curtis, 1832
STILBONOTA Stephens, 1835
MENISCUS Schiødte, 1839
ASYNIDA Gistel, 1848
AMERSIBIA Förster, 1869
BATHYCETES Förster, 1869
BOTHYNOPHRYS Förster, 1869
ENSIMUS Förster, 1869
OPISORHYSSA Kriechbaumer, 1890
ANARTHRONOTA Schmiedeknecht, 1900
CAMPOCINETA Schmiedeknecht, 1900
ECHTHRODOCA Schmiedeknecht, 1900
PIMPLOPTERUS Ashmead, 1900
ADELOPIMPLA Schulz, 1906
LOPHANTIUM Clément, 1925
GIBBONOTA Heinrich, 1937
LOXONOTA Aubert, 1993

##### Notes

Contra Brock (in prep.), subgenera are not employed as there is little justification for their use and some ‘subgenera’ seem to be of use only in Europe, whereas there is a great variety of species in the wider world referred to Lissonota (Lissonota) by default.

species of *Lissonota* excluded from British and Irish list:

[*bilineata* Gravenhorst, 1829] J.P. Brock (pers. comm.) has not seen any authentic British material of this species.

[*funebris* Habermehl, 1923] Only known in Britain from L. Carr material (supposedly from Lichfield) and hence should have been excluded from the previous checklist (J.P. Brock, pers. comm.). See Perkins (1953) and Shaw (2003) for discussions on the inadmissability of species to the British list that are only represented by Carr’s material.

[*impressor* Gravenhorst, 1829; syn. *basalis* Brischke, 1865] Identified by Morley (1908) and Aubert (1978) as *impressor* Grav., based on Morley’s interpretation of Thomson’s interpretation; the species involved is apparently undescribed (J.P. Brock, pers. comm.).

#### Lissonota accusator

(Fabricius, 1793)

Ichneumon accusator Fabricius, 1793
segmentator misident.
rusticator (Thunberg, 1824, *Ichneumon*)
humeralis (Zetterstedt, 1838, *Tryphon*) preocc.
unicincta Holmgren, 1860
thomsoni Schmiedeknecht, 1900
nigricoxa Strobl, 1902
accusatrix Schulz, 1906
segmentellator Aubert, 1967

##### Distribution

England, Ireland

##### Notes

Brock (in prep.) does not explain his use of *rusticator* as the valid name for the species Horstmann (2001a) refers to as *accusator*, so the latter is followed here. The name *accusator* has often been applied to *Cylloceria melancholica* (Gravenhorst).

#### Lissonota admontensis

Strobl, 1902

alpina Strobl, 1902
praebellator Aubert, 1967

##### Distribution

England, Scotland

##### Notes

added by Brock (in prep.)

#### Lissonota anomala

Holmgren, 1860

##### Distribution

Ireland

#### Lissonota antennalis

Thomson, 1877

##### Distribution

England, Wales

##### Notes

added by Brock (in prep.)

#### Lissonota argiola

Gravenhorst, 1829

eximia Habermehl, 1918

##### Distribution

England, Scotland, Wales, Ireland

#### Lissonota biguttata

Holmgren, 1860

femorata Holmgren, 1860
crassipes Thomson, 1877

##### Distribution

England, Ireland

#### Lissonota buccator

(Thunberg, 1824)

Ichneumon buccator Thunberg, 1824
varicoxa Thomson, 1887
iridipennis Kriechbaumer, 1900

##### Distribution

England, Scotland

##### Notes

There is some doubt about the correct name of this speceis as it is not certain which species Thunberg’s type belongs to within the *buccator* group of species (Brock, in prep.); distribution data from Horstmann (2003b); Brock (in prep.) states that Irish records require confirmation.

#### Lissonota canaliculata

(Szépligeti, 1899)

Meniscus canaliculatus Szépligeti, 1899
pimplator misident.
flavipes Lucas, 1849

##### Distribution

England

##### Notes

added by Brock (in prep.)

#### Lissonota carbonaria

Holmgren, 1860

melania Holmgren, 1860
artemisiae Tschek, 1871

##### Distribution

England, Scotland

##### Notes

Brock (in prep.) states that Irish records require confirmation.

#### Lissonota clypealis

Thomson, 1877

albobarbata Strobl, 1902

##### Distribution

England, Scotland, Ireland

#### Lissonota clypeator

(Gravenhorst, 1820)

Ichneumon clypeator Gravenhorst, 1820
cylindrator misident.
Lissonota clypeator ?*coccinea* (Christ, 1791, *Ichneumon*) preocc.
unicornis Strobl, 1902
nigrescens Constantineanu, 1929
spectabilis Schmiedeknecht, 1935
magna Heinrich, 1952

##### Distribution

England, Scotland, Ireland

#### Lissonota coracina

(Gmelin, 1790)

Ichneumon coracinus Gmelin, 1790
bellator (Gravenhorst, 1807, *Ichneumon*) preocc.
tricoloria (Thunberg, 1824, *Ichneumon*)
irrigua Thomson, 1888
bellatrix Schulz, 1906
meridionalis Seyrig, 1928

##### Distribution

England, Scotland, Ireland

#### Lissonota cruentator

(Panzer, 1809)

Alomya cruentator Panzer, 1809
insignita Gravenhorst, 1829
verberans Gravenhorst, 1829
cruentatrix (Schulz, 1906, *Alomya*)
rufifemur Kiss, 1926
szepligeti Kiss, 1926

##### Distribution

England, Ireland

#### Lissonota culiciformis

Gravenhorst, 1829

lateralis Gravenhorst, 1829
cruenta Vollenhoven, 1858
assimilis Brischke, 1880
sziladyi Kiss, 1926

##### Distribution

England, Wales

##### Notes

added by Brock (in prep.)

#### Lissonota deversor

Gravenhorst, 1829

##### Distribution

England

#### Lissonota digestor

(Thunberg, 1824)

Ichneumon digestor Thunberg, 1824
vocator (Thunberg, 1824, *Ichneumon*)
hians Thomson, 1877

##### Distribution

England, Scotland

#### Lissonota distincta

Bridgman, 1889

##### Distribution

England

#### Lissonota dubia

Holmgren, 1856

jugorum (Strobl, 1903, *Mesoleius*)
duplanae (Heinrich, 1937, *Gibbonota*)

##### Distribution

England, Scotland

##### Notes

Brock (in prep.) states that Irish records require confirmation.

#### Lissonota erythrina

Holmgren, 1860

pusilla Habermehl, 1918

##### Distribution

Scotland

##### Notes

added by Brock (in prep.)

#### Lissonota fletcheri

Bridgman, 1882

##### Distribution

England

##### Notes

Brock (in prep.) states that Irish records require confirmation.

#### Lissonota folii

Thomson, 1877

transversa Bridgman, 1889
areolata (Kiss, 1924, *Clistopyga*) preocc.

##### Distribution

England, Ireland

#### Lissonota freyi

(Hellén, 1915)

Meniscus freyi Hellén, 1915
tuberculata (Hellén, 1915, *Meniscus*)
sesiae Habermehl, 1918

##### Distribution

England

##### Notes

added by Brock (in prep.)

#### Lissonota frontalis

(Desvignes, 1856)

Lampronota frontalis Desvignes, 1856
canaliculata misident.
sulcator (Morley, 1908, *Meniscus*)

##### Distribution

England, Scotland

#### Lissonota fulvipes

(Desvignes, 1856)

Lampronota fulvipes Desvignes, 1856
piffardi (Morley, 1908, *Meniscus*)

##### Distribution

England

#### Lissonota fundator

(Thunberg, 1824)

Ichneumon fundator Thunberg, 1824
sulphurifera Gravenhorst, 1829
rimator Thomson, 1877
affinis (Szépligeti, 1899, *Meniscus*) preocc.
caudata (Szépligeti, 1899, *Meniscus*)
ruficoxis Schmiedeknecht, 1900
nigricoxis Pfankuch, 1920 preocc., unavailable

##### Distribution

England, Scotland, Ireland

#### Lissonota genator

Aubert, 1972

##### Distribution

Scotland

##### Notes

added by Brock (in prep.)

#### Lissonota gracilenta

Holmgren, 1860

##### Distribution

England, Scotland

##### Notes

added by Brock (in prep.)

#### Lissonota gracilipes

Thomson, 1877

##### Distribution

England

##### Notes

added by Brock (in prep.)

#### Lissonota halidayi

Holmgren, 1860

##### Distribution

England

#### Lissonota histrio

(Fabricius, 1798)

Banchus histrio Fabricius, 1798
marginator (Fabricius, 1804, *Bassus*) synonymy by Horstmann (2001a)
parallela Gravenhorst, 1829
dioszeghyi (Kiss, 1924, *Syzeuctus*)
nigrobasalis Constantineanu & Pisica, 1960

##### Distribution

England, Scotland, Ireland

#### Lissonota impressor

Gravenhorst, 1829

basalis Brischke, 1865
nigricoxis Ulbricht, 1913 preocc.
humerella Habermehl, 1918 preocc.

##### Distribution

England

##### Notes

added by Brock (in prep.)

#### Lissonota linearis

Gravenhorst, 1829

varicornis (Schmiedeknecht, 1900, *Campocineta*)
incerta Habermehl, 1918

##### Distribution

England

##### Notes

Brock (in prep.) states that Irish records require confirmation.

#### Lissonota lineata

Gravenhorst, 1829

flavovariegatus (Lucas, 1849, *Mesoleptus*) synonymy by Horstmann (1997)

##### Distribution

England, Wales

#### Lissonota lineolaris

(Gmelin, 1790)

Ichneumon lineolaris Gmelin, 1790
catenator (Panzer, 1804, *Ichneumon*)
gladiator (Thunberg, 1824, *Ichneumon*) preocc.
mammillator (Thunberg, 1824, *Ichneumon*)
signator (Thunberg, 1824, *Ichneumon*)
excavator (Zetterstedt, 1838, *Tryphon*)
facialis (Desvignes, 1862, *Ephialtes*)

##### Distribution

England, Scotland, Ireland, Isle of Man

#### Lissonota luffiator

Aubert, 1969

##### Distribution

England

##### Notes

added by Brock (in prep.)

#### Lissonota maculata

Brischke, 1865

affinis Brischke, 1865

##### Distribution

England

##### Notes

Brock (in prep.) states that Irish records require confirmation.

#### Lissonota magdalenae

Pfankuch, 1921

vernalis Roman, 1925

##### Distribution

England, Scotland, Ireland

##### Notes

added by Stelfox (1932); recorded originally from Ireland and therefore not listed in Fitton (1978), then from Scotland by Ashmole et al. (1983).

#### Lissonota mutator

Aubert, 1969

##### Distribution

England

##### Notes

distribution data from Shaw (1999)

#### Lissonota nigridens

Thomson, 1889

##### Distribution

England, Scotland, Ireland

#### Lissonota nitida

Gravenhorst, 1829

agnata Gravenhorst, 1829
rhenana Ulbricht, 1916 unavailable
lissonotoides (Habermehl, 1917, *Meniscus*)

##### Distribution

England

#### Lissonota obsoleta

Bridgman, 1889

##### Distribution

England

#### Lissonota palpalis

Thomson, 1889

oudemansi Smits
exareolata (Habermehl, 1923, *Meniscus*)
inareolata (Kiss, 1824, *Meniscus*) preocc.

##### Distribution

England, Ireland

#### Lissonota palpator

Aubert, 1969

parasitellae Horstmann, 2003 synonymy by Brock (in prep.)
errabunda misident. (Horstmann 2003b)

##### Distribution

England, Scotland

##### Notes

added by Horstmann (2003b)

#### Lissonota picticoxis

Schmiedeknecht, 1900

##### Distribution

England, Scotland

##### Notes

added by Brock (in prep.)

#### Lissonota pimplator

(Zetterstedt, 1838)

Tryphon pimplator Zetterstedt, 1838

##### Distribution

Scotland

##### Notes

added by Aubert (1978); Morley’s (Morley 1908) *pimplator* refers to *canaliculata* (Brock in prep.).

#### Lissonota pleuralis

Brischke, 1880

strigifrons Schmiedeknecht, 1900

##### Distribution

England

##### Notes

added by Aubert (1978)

#### Lissonota proxima

Fonscolombe, 1854

varipes (Desvignes, 1856, *Lampronota*)
commixta Holmgren, 1860
lapponica Holmgren, 1860
opacula Szépligeti, 1899
variipes Dalla Torre, 1901

##### Distribution

England, Ireland

#### Lissonota punctiventrator

Aubert, 1977

punctiventris misident.

##### Distribution

England, Scotland, Wales

##### Notes

added by Aubert (1978)

#### Lissonota punctiventris

Thomson, 1877

Lissonota punctiventris ?*errabunda* Holmgren

##### Distribution

England, Scotland

##### Notes

added by Brock (in prep.)

#### Lissonota quadrinotata

Gravenhorst, 1829

leucogona Gravenhorst, 1829
carinifrons Thomson, 1877

##### Distribution

England, Wales

#### Lissonota saturator

(Thunberg, 1824)

Ichneumon saturator Thunberg, 1824
pubescens (Zetterstedt, 1838, *Bassus*)
vicina Holmgren, 1860
basalis Thomson, 1889 preocc.
mutanda Schmiedeknecht, 1900

##### Distribution

England, Scotland, Wales

##### Notes

Brock (in prep.) states that Irish records require confirmation.

#### Lissonota semirufa

(Desvignes, 1856)

Lampronota semirufa Desvignes, 1856

##### Distribution

England, Scotland

##### Notes

some distribution data from Horstmann (2004c)

#### Lissonota setosa

(Geoffroy, 1785)

Ichneumon setosus Geoffroy, 1785
enervator (Fabricius, 1793, *Ichneumon*) preocc.
cryptator (Thunberg, 1824, *Ichneumon*)
renovator (Thunberg, 1824, *Ichneumon*)
nigra (Szépligeti, 1914, *Odinophora*)

##### Distribution

England

#### Lissonota silvatica

Habermehl, 1918

palpator Aubert, 1969

##### Distribution

England

##### Notes

added by Aubert (1978)

#### Lissonota stigmator

Aubert, 1972

##### Distribution

England, Scotland

#### Lissonota subaciculata

Bridgman, 1886

nitida Bridgman, 1886 preocc.

##### Distribution

England

##### Notes

Brock (in prep.) states that Irish records require confirmation.

#### Lissonota tenerrima

Thomson, 1877

variabilis Holmgren, 1860 synonymy by Brock (in prep.)
fracta Taschenberg, 1863
rufomedia Bridgman, 1886
trochanterata Bridgman, 1889 preocc.
trochanteralis Dalla Torre, 1901
procera Pfeffer, 1913
bimaculata Constantineanu & Ciochia, 1968 preocc.

##### Distribution

England, Scotland, Ireland

#### Lissonota trochanterator

Aubert, 1972

##### Distribution

England

#### Lissonota versicolor

Holmgren, 1860

formosa Bridgman, 1888
coxata Smits
rufithorax Habermehl, 1918

##### Distribution

England, Ireland

#### Lissonota sp. A

##### Distribution

England

##### Notes

added by Brock (in prep.)

#### Lissonota sp. C

##### Distribution

England

##### Notes

added by Brock (in prep.)

#### Lissonota sp. D

##### Distribution

England, Scotland, Wales

##### Notes

added by Brock (in prep.)

#### Lissonota sp. P

##### Distribution

Scotland

##### Notes

added by Brock (in prep.)

#### Lissonota sp. S

##### Distribution

England

##### Notes

added by Brock (in prep.); impressor misident.

#### Lissonota sp. SI

##### Distribution

England

##### Notes

added by Brock (in prep.)

#### Lissonota sp. V

basalis Brischke, 1865]

##### Distribution

England

##### Notes

added by Brock (in prep.)

#### Syzeuctus

Förster, 1869

DICERATOPS Förster, 1869
SYZEUCTA Thomson, 1889

##### Notes

Species of *Syzeuctus* excluded from the British and Irish list:

[*irrisorius* (Rossius, 1794, *Ichneumon*)] Brock (in prep.) found no British specimens of *irrisorius*.

#### Syzeuctus bicornis

(Gravenhorst, 1829)

Lissonta bicornis Gravenhorst, 1829

##### Distribution

England

#### Syzeuctus fuscator

(Panzer, 1809)

Ophion fuscator Panzer, 1809
maculatorius (Fabricius, 1787, *Ichneumon*) preocc.
bicolor Szépligeti, 1899
rufipes Kiss, 1933

##### Distribution

England, Wales

#### BANCHINI

Wesmael, 1845

#### Banchus

Fabricius, 1798

##### Notes

Distribution data from Fitton (1985a) and the NMS.

#### Banchus crefeldensis

Ulbricht, 1916

croaticus Hensch, 1928

##### Distribution

Scotland, Ireland

#### Banchus dilatatorius

(Thunberg, 1824)

Ichneumon dilatatorius Thunberg, 1824
variegator misident.
acuminator (Fabricius, 1787, *Ichneumon*)
compressus (Fabricius, 1787, *Ichneumon*)
sibiricus Meyer, 1927

##### Distribution

England, Scotland

#### Banchus falcatorius

(Fabricius, 1775)

Ichneumon falcatorius Fabricius, 1775
variegator (Fabricius, 1775, *Ichneumon*)
tricolor (Schrank, 1776, *Ichneumon*)
intersectus (Geoffroy, 1785, *Ichneumon*)
aries (Christ, 1791, *Ichneumon*)
notatorius (Olivier, 1792, *Ichneumon*) preocc.
histrio (Schrank, 1802, *Ichneumon*) preocc.
labiatus (Schrank, 1802, *Ichneumon*)
falcator Fabricius, 1804
luteofasciatus Ulbricht, 1911 unavailable
nobilitator Morley, 1915
sanguinator Meyer, 1922
lavrovi Meyer, 1927
nigromarginatus Constantineanu & Pisica, 1960
propitius Kuslitzky, 1979

##### Distribution

England

#### Banchus hastator

(Fabricius, 1793)

Ichneumon hastator Fabricius, 1793
pungitor (Thunberg, 1824, *Ichneumon*)
reticulator (Thunberg, 1824, *Ichneumon*)
femoralis Thomson, 1897
kolosovi Meyer, 1925

##### Distribution

England, Scotland, Ireland

#### Banchus moppiti

Fitton, 1985

##### Notes

added by Fitton (1985a); known in Britain only from an unlabelled specimen from Desvignes’s collection.

#### Banchus palpalis

Ruthe, 1859

monileatus misident.
groenlandicus Aurivilius, 1890

##### Distribution

England, Scotland, Ireland

##### Notes

added by Fitton (1985a)

#### Banchus pictus

Fabricius, 1798

cultratus (Gmelin, 1790, *Ichneumon*) preocc.
mutillatus (Christ, 1791, *Ichneumon*) preocc.
bipunctatus Hensch, 1928
zagoriensis Hensch, 1928

##### Distribution

England, Wales

#### Banchus volutatorius

(Linnaeus, 1758)

Ichneumon volutatorius Linnaeus, 1758
venator (Linnaeus, 1758, *Ichneumon*)
umbellatarum (Schrank, 1786, *Ichneumon*)
certator (Thunberg, 1824, *Ichneumon*)
monileatus Gravenhorst, 1829
farrani Curtis, 1836
moniliatus Marshall, 1872
alticola Schmiedeknecht, 1910
calcaratus Szépligeti, 1910
obscurus Meyer, 1926

##### Distribution

England, Scotland, Wales, Ireland

#### Exetastes

Gravenhorst, 1829

LEPTOBATUS Gravenhorst, 1829
RHIMPHALEA Förster, 1869
SEMNOPHRYS Förster, 1869
ALLEXETASTES Kokujev, 1904

#### Exetastes adpressorius

(Thunberg, 1824)

Ichneumon adpressorius Thunberg, 1824
guttatorius Gravenhorst, 1829
tristis Gravenhorst, 1829
procera Kriechbaumer, 1894 unavailable
guttifer Thomson, 1897
medianus Szépligeti, 1898
albopictus Aubert, 1959
albopictor Aubert, 1972

##### Distribution

England, Scotland, Ireland

#### Exetastes atrator

(Forster, 1771)

Ichneumon atrator Forster, 1771
cinctipes (Retzius, 1783, *Ichneumon*)
junci (Geoffroy, 1785, *Ichneumon*)
osculatorius (Fabricius, 1787, *Ichneumon*)
obscurator (Gmelin, 1790, *Ichneumon*)
clavator (Fabricius, 1793, *Ichneumon*) preocc.
tarsator (Fabricius, 1804, *Ophion*)
sinuatorius (Thunberg, 1824, *Ichneumon*)

##### Distribution

England, Scotland, Ireland

#### Exetastes calobatus

Gravenhorst, 1829

calobates Dalla Torre, 1901

##### Distribution

England

#### Exetastes femorator

Desvignes, 1856

##### Distribution

England

##### Notes

Brock (in prep.) states that Irish records require confirmation.

#### Exetastes fornicator

(Fabricius, 1781)

Ichneumon fornicator Fabricius, 1781
exapansor (Thunberg, 1824, *Ichneumon*)
punctulatus Kokujev, 1905

##### Distribution

England, Ireland

#### Exetastes illusor

Gravenhorst, 1829

minor Szépligeti, 1901
annulatus Habermehl, 1927
Exetastes illusor ?*geniculosus* Holmgren, 1860

##### Distribution

England, Scotland, Ireland, Isle of Man

#### Exetastes illyricus

Strobl, 1904

##### Distribution

England

##### Notes

added by Brock (in prep.)

#### Exetastes laevigator

(Villers, 1789)

Ichneumon laevigator Villers, 1789
cothurnatus (Gravenhorst, 1807, *Ichneumon*) preocc.
incurvator (Thunberg, 1824, *Ichneumon*)
alpinus Kriechbaumer, 1888
puberulus (Szépligeti, 1898,)
levigator Dalla Torre, 1901
similis Kokujev, 1905
nigriventris Meyer, 1927

##### Distribution

England

#### Exetastes maurus

Desvignes, 1856

facialis Desvignes, 1856
benoisti Seyrig, 1926
melanopus Meyer, 1927
croaticus Hensch, 1928

##### Distribution

England

#### Exetastes nigripes

Gravenhorst, 1829

##### Distribution

England, Scotland, Wales

##### Notes

Brock (in prep.) states that Irish records require confirmation.

#### Exetastes tibialis

Pfankuch, 1921

##### Distribution

England

##### Notes

added by Brock (in prep.)

#### Rynchobanchus

Kriechbaumer, 1894

#### Rynchobanchus flavopictus

Heinrich, 1937

##### Distribution

England

##### Notes

added by Fitton (1987)

#### GLYPTINI

Cushman & Rohwer, 1920

#### Apophua

Morley, 1913

#### Apophua bipunctoria

(Thunberg, 1824)

Ichneumon bipunctorius Thunberg, 1824
cubitoria (Thunberg, 1824, *Ichneumon*)
flavolineata (Gravenhorst, 1829, *Glypta*)
baltica (Habermehl, 1926, *Glypta*)

##### Distribution

England, Scotland, Wales, Ireland, Isle of Man

#### Apophua cicatricosa

(Ratzeburg, 1848)

Glypta cicatricosa Ratzeburg, 1848
crenulata (Thomson, 1889, *Glypta*)

#### Apophua evanescens

(Ratzeburg, 1848)

Glypta evanescens Ratzeburg, 1848
albifrons (Holmgren, 1856, *Glypta*)

##### Distribution

England, Scotland, Ireland

#### Apophua genalis

(Möller, 1883)

Glypta genalis Möller, 1883
superba (Hellén, 1915, *Glypta*)

##### Distribution

Ireland

#### Diblastomorpha

Förster, 1869

##### Notes

Reinstated as a valid genus, from synonymy under *Glypta*, by Watanabe and Maeto (2013).

#### Diblastomorpha cylindrator

(Fabricius, 1787)

Ichneumon cylindrator Fabricius, 1787
erythrogaster Lucas, 1849
bicornis Boie, 1850
bicornis Desvignes, 1856 preocc.
corniculata Brischke, 1865
elegans Vollenhoven, 1873
ephippigera Kriechbaumer, 1895
ruficornis Szépligeti, 1898 preocc.
paleanae Kriechbaumer, 1900
szepligetii Dalla Torre, 1901
cylindatrix (Schulz, 1906, *Lissonota*)
abundans (Schmiedeknecht, 1934, *Diblastomorpha*)

##### Distribution

England, Scotland, Wales, Ireland

#### Diblastomorpha rostrata

Holmgren, 1860

##### Distribution

England, Ireland, Isle of Man

##### Notes

Treated as a valid species, rather than a synonym of *cylindrator*, by Brock (in prep.).

#### Glypta

Gravenhorst, 1829

CONOBLASTA Förster, 1869
FOVEOGLYPTA Hellén, 1915

##### Notes

Species of *Glypta* excluded from the British and Irish list:

[*schneideri* Krieger, 1897] Recorded from Ireland by Johnson (1921) (and not listed by Fitton 1978 as not known from Britain), but J.P. Brock has seen no authentic British or Irish material.

[*scalaris* Gravenhorst, 1829] Misidentified *scalaris* are described by Brock (in prep.) as Glypa sp. PU.

[*teres* Gravenhorst, 1829] British specimens identified as *teres* have proved to be misidentified *bifoveolata* (Brock, in prep.).

#### Glypta bifoveolata

Gravenhorst, 1829

setosa Roman, 1909

##### Distribution

England, Scotland, Wales, Ireland

#### Glypta ceratites

Gravenhorst, 1829

##### Distribution

England, Scotland, Ireland, Isle of Man

#### Glypta consimilis

Holmgren, 1860

brevicornis Rudow, 1883
parvicornuta Bridgman, 1886
xanthognatha Thomson, 1889
berolinae (Strand, 1918, *Conoblasta*)

##### Distribution

England, Scotland, Ireland

#### Glypta elongata

Holmgren, 1860

##### Distribution

England, Scotland, Wales, Ireland

#### Glypta extincta

Ratzeburg, 1852

nigriventris Thomson, 1889

##### Distribution

England, Scotland

#### Glypta femorator

Desvignes, 1856

filicornis Thomson, 1889
femoratrix Schulz, 1906
elegantula Hellén, 1915
obscurata Kiss, 1929
pellucida Schmiedeknecht, 1935
triangularis Schmiedeknecht, 1935
curvicoxa Kuslitzky, 1977

##### Distribution

England, Scotland, Ireland

#### Glypta fronticornis

Gravenhorst, 1829

dispar Schiødte, 1839 synonymy by Horstmann (2004a)

##### Distribution

England, Scotland, Ireland

#### Glypta haesitator

Gravenhorst, 1829

haesitatrix Schulz, 1906
australis (Hedwig, 1959, *Lycorina*)

##### Distribution

England, Scotland, Ireland

#### Glypta incisa

Gravenhorst, 1829

##### Distribution

England, Wales

#### Glypta lapponica

Holmgren, 1860

annulata Bridgman, 1890
areolaris Hellén, 1915
nigricoxa (Kokujev, 1927, *Conoblasta*)
alpina (Heinrich, 1949, *Conoblasta*)

##### Distribution

England, Scotland, Ireland

#### Glypta lineata

Desvignes, 1856

##### Distribution

England

#### Glypta longicauda

Hartig, 1838

nigrotrochanterata Strobl, 1902

##### Distribution

Ireland

#### Glypta longispinis

(Gmelin, 1790)

Ichneumon longispinis Gmelin, 1790
provincialis Fonscolombe, 1854
rubicunda Bridgman, 1890
algerica Habemehl, 1917
zangezurica Kuslitzky, 1974

##### Distribution

England

#### Glypta mensurator

(Fabricius, 1775)

Ichneumon mensurator Fabricius, 1775
lugubrina Holmgren, 1860
macropyga Hellén, 1915
heydeni Habemehl, 1917
jaroslavensis Shestakov, 1927

##### Distribution

England, Scotland, Wales, Ireland

#### Glypta microcera

Thomson, 1899

segrex Kokujev, 1913

##### Distribution

England

##### Notes

added by Aubert (1978)

#### Glypta monoceros

Gravenhorst, 1829

##### Distribution

England, Scotland, Wales, Ireland

#### Glypta nigricornis

Thomson, 1899

rufipes Brischke, 1865 preocc.
brischkei Dalla Torre, 1901 preocc.
papyri Speiser, 1908

##### Distribution

England, Scotland, Ireland

##### Notes

added by Aubert (1978)

#### Glypta nigrina

Desvignes, 1856

flavipes Desvignes, 1856
ruficeps Desvignes, 1856
fractigena Thomson, 1889
obscura Pfankuch, 1924 unavailable
clypeodentata Bauer, 1959
habermani Ozols, 1959

##### Distribution

England, Scotland, Ireland

#### Glypta nigrotrochanterator

Strobl, 1902

mensurator misident.
longicauda misident.

##### Distribution

England, Scotland, Ireland

##### Notes

added by Brock (in prep.)

#### Glypta parvicaudata

Bridgman, 1889

##### Distribution

England, Scotland, Ireland

#### Glypta pedata

Desvignes, 1856

##### Distribution

England

#### Glypta pictipes

Taschenberg, 1863

##### Distribution

England, Scotland, Ireland

##### Notes

added by Aubert (1978)

#### Glypta punctifrons

Bridgman, 1890

##### Distribution

Scotland

##### Notes

Synonymised under *scalaris* by Aubert (1978) but this synonymy rejected by Brock (in prep.). Known only from the male type specimen, *punctifrons* is essentially an unknown species within *Glypta*.

#### Glypta resinanae

Hartig, 1838

arreptans Hellén, 1915
summimontis Heinrich, 1953

##### Distribution

England

#### Glypta rufata

Bridgman, 1887

##### Distribution

England, Ireland

#### Glypta sculpturata

Gravenhorst, 1829

macrura Habermehl, 1918
rufoclypeata Kiss, 1924

##### Distribution

England, Ireland

#### Glypta scutellaris

Thomson, 1899

##### Distribution

England, Scotland

##### Notes

added by Brock (in prep.)

#### Glypta similis

Bridgman, 1886

rufipes Thomson, 1889 preocc.
thomsonii Dalla Torre, 1901
thomsoni Strobl, 1902 preocc.

##### Distribution

England, Ireland

#### Glypta tenuicornis

Thomson, 1889

pygmaea Shestakov, 1927

##### Distribution

England

#### Glypta trochanterata

Bridgman, 1886

##### Distribution

England, Scotland, Ireland

#### Glypta ulbrichti

Habermehl, 1926

##### Distribution

England

##### Notes

added by Brock (in prep.)

#### Glypta vulnerator

Gravenhorst, 1829

vulneratrix Schulz, 1906
monstrosa Hellén, 1915

##### Distribution

England, Scotland, Ireland

#### Glypta woerzi

(Hedwig, 1952)

Conoblasta woerzi Hedwig, 1952

##### Distribution

England

##### Notes

added by Brock (in prep.)

#### Glypta sp. PA

##### Distribution

England

##### Notes

added by Brock (in prep.)

#### Glypta sp. PU

scalaris misident.

##### Distribution

Ireland

##### Notes

added by Brock (in prep.)

#### Teleutaea

Förster, 1869

HOPLITOPHRYS Förster, 1869
TELEUTEA Thomson, 1889

#### Teleutaea brischkei

(Holmgren, 1860)

Glypta brischkei Holmgren, 1860

##### Distribution

England

##### Notes

added by Aubert (1978); specimens also in NMS and Horniman Museum

### Campopleginae

#### CAMPOPLEGINAE

Förster, 1869

##### Notes

Many of the species new to the fauna have been identified by K. Horstmann from material in NMS and BMNH. Additional references are given.

#### Alcima

Förster, 1869

#### Alcima orbitale

(Gravenhorst, 1829)

Campoplex orbitalis Gravenhorst, 1829
alboscutellare (Thomson, 1887, *Casinaria*)
carinata (Kriechbaumer, 1898, *Casinaria*)

##### Distribution

England, Wales

##### Notes

Listed as *Casinaria orbitalis* by Fitton (1978).

#### Bathyplectes

Förster, 1869

CANIDIA Holmgren, 1860
RHEXINEURA Förster, 1869
BIOLYSIA Schmiedeknecht, 1907
BATHYPIESTA Aubert, 1979

#### Bathyplectes anurus

(Thomson, 1887)

Canidia anura Thomson, 1887

##### Distribution

England, Ireland

#### Bathyplectes balteatus

(Thomson, 1887)

Canidia balteata Thomson, 1887
trisculptus (Habermehl, 1926, *Canidia*)

##### Distribution

England

##### Notes

NMS, det. Horstmann, added here

#### Bathyplectes curculionis

(Thomson, 1887)

Canidia curculionis Thomson, 1887
carthaginiensis (Smits van Burgst, 1913, *Canidia*) synonymy by Horstmann (2009d)

##### Distribution

England, Ireland

##### Notes

added by Horstmann (1974); omitted by Fitton (1978)

#### Bathyplectes exiguus

(Gravenhorst, 1829)

Campoplex exiguus Gravenhorst, 1829
subcinctus (Gravenhorst, 1829, *Campoplex*)

##### Distribution

England, Scotland, Ireland

#### Bathyplectes immolator

(Gravenhorst, 1829)

Campoplex immolator Gravenhorst, 1829
marginellus (Thomson, 1887, *Nepiesta*)

##### Distribution

Ireland

#### Bathyplectes infernalis

(Gravenhorst, 1820)

Ichneumon infernalis Gravenhorst, 1820
infernalis (Gravenhorst, 1829, *Mesoleptus*) preocc.
tristis (Gravenhorst, 1829, *Campoplex*)
trochantellus (Thomson, 1887, *Canidia*)

##### Distribution

England, Ireland

#### Bathyplectes quinqueangularis

(Ratzeburg, 1852)

Campoplex quinqueangularis Ratzeburg, 1852

##### Distribution

Ireland

##### Notes

added by Horstmann (1974); omitted by Fitton (1978)

#### Bathyplectes rostratus

(Thomson, 1887)

Canidia rostrata Thomson, 1887

##### Distribution

Scotland, Ireland

#### Bathyplectes rufipes

Horstmann, 1974

##### Distribution

England

##### Notes

NMS, det. Horstmann, added here

#### Bathyplectes tibiator

(Gravenhorst, 1820)

Ichneumon tibiator Gravenhorst, 1820
corvinus (Thomson, 1887, *Canidia*)

##### Distribution

England

##### Notes

BMNH, added here

#### Callidora

Förster, 1869

PANTROPA Förster, 1869
NEOCALLIDORA Ozols, 1966

#### Callidora analis

(Gravenhorst, 1829)

Campoplex analis Gravenhorst, 1829

##### Distribution

England

##### Notes

NMS, det. Horstmann, added here

#### Campoletis

Förster, 1869

ANILASTUS Förster, 1869
ECPHORA Förster, 1869 preocc.
ANILASTA Thomson, 1877
ECPHOROPSIS Ashmead, 1900

#### Campoletis agilis

(Holmgren, 1860)

Sagaritis agilis Holmgren, 1860

##### Distribution

Ireland

#### Campoletis annulata

(Gravenhorst, 1829)

Campoplex annulatus Gravenhorst, 1829
maculipes (Tschek, 1871, *Sagaritis*)
trochanterata (Kriechbaumer, 1894, *Sagaritis*)
nigripes (Seyrig, 1928, *Sagaritis*) preocc.

##### Distribution

England, Scotland, Ireland

#### Campoletis cognata

(Tschek, 1871)

Sagaritis cognata Tschek, 1871

#### Campoletis crassicornis

(Tschek, 1871)

Sagaritis crassicornis Tschek, 1871
brachycera (Thomson, 1877, *Sagaritis*)

##### Distribution

England, Scotland, Ireland

#### Campoletis dilatator

(Thunberg, 1824)

Ichneumon dilatator Thunberg, 1824
mediator (Zetterstedt, 1838, *Porizon*)

##### Distribution

Ireland

#### Campoletis ensator

(Gravenhorst, 1829)

Campoplex ensator Gravenhorst, 1829
holmgreni (Tschek, 1871, *Sagaritis*) synonymy by Horstmann (2000e)

##### Distribution

England, Scotland, Ireland

#### Campoletis fasciata

(Bridgman, 1888)

Sagaritis fasciata Bridgman, 1888

##### Distribution

England

#### Campoletis femoralis

(Gravenhorst, 1829)

Campoplex femoralis Gravenhorst, 1829
laticollis (Holmgren, 1860, *Sagaritis*)

##### Distribution

Ireland

#### Campoletis fuscipes

(Holmgren, 1856)

Campoplex fuscipes Holmgren, 1856
semirufa (Szépligeti, 1916, *Omorgus*)

##### Distribution

England, Ireland

#### Campoletis incisa

(Bridgman, 1883)

Sagaritis incisa Bridgman, 1883

##### Distribution

Ireland

#### Campoletis latrator

(Gravenhorst, 1829)

Campoplex latrator Gravenhorst, 1829
assimilis (Gravenhorst, 1829, *Campoplex*) synonymy by Horstmann (2000e)
mitis (Holmgren, 1860, *Sagaritis*)
latratrix (Schulz, 1906, *Sagaritis*)
bicingulata (Szépligeti, 1916, *Omorgus*)

##### Distribution

England, Scotland, Ireland, Isle of Man

##### Notes

Horstmann (2000e) shows that this is not a junior homonym (treated as such in Yu and Horstmann 1997).

#### Campoletis postica

(Bridgman & Fitch, 1885)

Sagaritis postica Bridgman & Fitch, 1885
postica (Bridgman, 1886, *Sagaritis*) preocc.

##### Distribution

England, Scotland, Ireland

#### Campoletis punctata

(Bridgman, 1886)

Sagaritis punctata Bridgman, 1886

##### Distribution

England, Scotland, Ireland

#### Campoletis rapax

(Gravenhorst, 1829)

Campoplex rapax Gravenhorst, 1829
erythropus (Thomson, 1887, *Sagaritis*) synonymy by Horstmann (2000e)
curticaudis (Szépligeti, 1916, *Omorgus*)

##### Distribution

England, Scotland, Wales, Ireland

##### Notes

J.F. Perkins, in his curation of the BMNH collection, applied the name *rapax* Grav. to a species of *Hyposoter*; *Campoletis* specimens were under *erythropus* (Thomson).

#### Campoletis raptor

(Zetterstedt, 1838)

Porizon raptor Zetterstedt, 1838
raptrix (Schulz, 1906, *Sagaritis*)
dubiosa (Szépligeti, 1916, *Omorgus*)
rufator Aubert, 1960

#### Campoletis thomsoni

(Roman, 1915)

Sagaritis thomsoni Roman, 1915

##### Distribution

England, Scotland

##### Notes

NMS, det. Horstmann, added here

#### Campoletis trichoptili

(Bauer, 1936)

Sagaritis trichoptili Bauer, 1936

##### Distribution

England

##### Notes

NMS, det. Horstmann, added here

#### Campoletis varians

(Thomson, 1887)

Sagaritis varians Thomson, 1887
completa (Szépligeti, 1916, *Omorgus*)

##### Distribution

England, Scotland, Wales, Ireland

##### Notes

added by Johnson (1929); only known originally from Ireland so omitted by Fitton (1978); also specimens in NMS and BMNH, det. K. Horstmann.

#### Campoletis viennensis

(Gravenhorst, 1829)

Campoplex viennensis Gravenhorst, 1829
annulator (Zetterstedt, 1838, *Porizon*)
vexans (Holmgren, 1860, *Limneria*)
maculipes (Strobl, 1904, *Anilasta*)
subdentata (Hellén, 1949, *Sagaritopsis*)

##### Distribution

England, Ireland

#### Campoletis vimmeri

(Gregor, 1935)

Sagaritis vimmeri Gregor, 1935

##### Distribution

Scotland

##### Notes

NMS, det. Riedel, added here

#### Campoletis zonata

(Gravenhorst, 1829)

Campoplex zonatus Gravenhorst, 1829

##### Distribution

England, Scotland, Ireland

#### Campoplex

Gravenhorst, 1829

DIORATICA Förster, 1869
OMORGUS Förster, 1869
OMORGA Förster, 1869

##### Notes

The *Campoplex* material in BMNH was sorted and determined by J.F. Perkins and more recently identified by K. Horstmann.

doubtfully placed species of *Campoplex*:

[*arvensis* Gravenhorst, 1829 nom. dub.] Listed as *Sinophorus arvensis* by Fitton (1978), K. Horstmann (pers. comm.) has seen only the type specimen of this species, which remains uninterpreted at present

species of *Campoplex* excluded from the British and Irish list:

[*borealis* (Zetterstedt, 1838, *Porizon*)] All British specimens in NMS were reidentified as *C. caloptiliae* by Horstmann

[*melanostoma* (Strobl, 1904, *Limneria*); syn. *anterior* Aubert, 1960] On earlier versions of this checklist, *C. melanostoma* was included based on a reared specimen in NMS, but this was based on a misidentification of *C. punctulatus*.

#### Campoplex abbreviatus

(Brischke, 1880)

Limneria abbreviata Brischke, 1880

#### Campoplex alticolellae

Horstmann, 1980

##### Distribution

England, Scotland, Wales

##### Notes

NMS, det. Horstmann, added here

#### Campoplex bilobus

(Thomson, 1887)

Omorga biloba Thomson, 1887

##### Distribution

England

#### Campoplex brevicornis

(Szépligeti, 1916)

Omorgus brevicornis Szépligeti, 1916
flavocinctus (Seyrig, 1928, *Sagaritis*)

##### Distribution

England

##### Notes

NMS, det. Horstmann, added here

#### Campoplex caloptiliae

Horstmann, 2013

##### Distribution

England, Scotland

##### Notes

added by Horstmann (2013)

#### Campoplex cingulatus

(Brischke, 1880)

Limneria cingulata Brischke, 1880

#### Campoplex continuus

(Thomson, 1887)

Omorga continua Thomson, 1887

##### Distribution

England

##### Notes

The true *continuus* has been identified by K. Horstmann (specimen in NMS; see note under *pyraustae*).

#### Campoplex coracinus

(Thomson, 1887)

Omorga coracina Thomson, 1887
submarginatus (Bridgman, 1899, *Limneria*)

##### Distribution

England

#### Campoplex crassus

Horstmann, 1980

##### Distribution

England

##### Notes

NMS, det. Horstmann, added here

#### Campoplex cursitans

(Holmgren, 1860)

Limneria cursitans Holmgren, 1860

##### Distribution

England, Ireland

#### Campoplex deficiens

Gravenhorst, 1829

algerica (Habermehl, 1922, *Omorga*)

##### Distribution

England

##### Notes

Listed as *Venturia deficiens* by Yu and Horstmann (1997) but treated as a species of *Campoplex* by Horstmann (2000e). Has been misidentified as *Campoplex difformis*.

#### Campoplex difformis

(Gmelin, 1790)

Ichneumon difformis Gmelin, 1790
lineolatus Ratzeburg, 1844
mutabilis (Holmgren, 1860, *Limneria*)

##### Distribution

England, Ireland

#### Campoplex eudoniae

Horstmann & Yu, 1999

rufipes (Bridgman, 1883, *Nemeritis*) preocc.
ruficoxa (Thomson, 1887, *Omorga*) preocc.

##### Distribution

England, Scotland, Ireland

##### Notes

some distribution data from Horstmann and Yu (1999)

#### Campoplex faunus

Gravenhorst, 1829

xanthocarpus (Szépligeti, 1916, *Omorgus*)

##### Distribution

England, Ireland

#### Campoplex formosanae

Horstmann, 2012

##### Distribution

England

##### Notes

NMS, det. Horstmann, added here

#### Campoplex fusciplica

(Thomson, 1887)

Omorga fusciplica Thomson, 1887

#### Campoplex hadrocerus

(Thomson, 1887)

Omorga hadrocera Thomson, 1887
fasciatus (Bridgman, 1889, *Limneria*)

##### Distribution

England, Ireland

#### Campoplex interruptus

Horstmann, 1993

##### Distribution

England, Scotland

##### Notes

NMS, det. Horstmann, added here

#### Campoplex investigator

(Habermehl, 1923)

Omorga investigator Habermehl, 1923

##### Distribution

England

##### Notes

NMS, det. Horstmann, added here

#### Campoplex jaeckhi

(Bauer, 1936)

Dioctes jaeckhi Bauer, 1936

##### Distribution

England

##### Notes

added by Horstmann (2012a)

#### Campoplex lugubrinus

(Holmgren, 1860)

Limneria lugubrina Holmgren, 1860
pusillus (Szépligeti, 1916, *Angitia*)

##### Distribution

England, Scotland, Ireland

#### Campoplex lyratus

(Thomson, 1887)

Omorga lyrata Thomson, 1887

##### Distribution

England, Scotland, Wales, Ireland, Isle of Man

##### Notes

added by Shaw (1984)

#### Campoplex melanostictus

Gravenhorst, 1829

##### Distribution

England, Scotland

#### Campoplex molestus

Gravenhorst, 1829

##### Distribution

England

#### Campoplex multicinctus

Gravenhorst, 1829

excentricus (Bauer, 1937, *Omorgus*) synonymy by Horstmann (2000d)

##### Distribution

England, Ireland

#### Campoplex ovatus

(Brischke, 1880)

Limneria ovata Brischke, 1880

##### Distribution

England, Scotland

#### Campoplex procerus

(Brischke, 1880)

Limneria procera Brischke, 1880

#### Campoplex psammae

(Morley, 1915)

Omorga psammae Morley, 1915

##### Distribution

England, Scotland, Ireland

##### Notes

Described as a variety of *Omorga* (=*Campoplex*) *ensator* (Grav., 1829) and listed as *Campoplex psammae* by Fitton (1978) but not not listed in Yu and Horstmann (1997) and treated, without justification, as a subspecies of *ensator* by Yu et al. (2012). Horstmann (2012a) regarded this as a valid species.

#### Campoplex punctipleuris

Horstmann, 1980

alhpictus (Pfankuch, 1924, *Omorga*) unavailable
albipictus Horstmann, 1986 unavailable

##### Distribution

England, Scotland, Wales

##### Notes

added by Horstmann (1980a)

#### Campoplex punctulatus

(Szépligeti, 1916)

Omorgus punctulatus Szépligeti, 1916

##### Distribution

England, Scotland, Ireland

##### Notes

BMNH, NMS, det. Horstmann, added here

#### Campoplex pyraustae

Smith, 1931

continuus misident.

##### Distribution

England, Scotland, Wales, Ireland, Isle of Man

##### Notes

Added by Shaw and Aeschliman (1994); *Campoplex pyraustae* has sometimes been misidentified as *continuus* (K. Horstmann, pers. comm.).

#### Campoplex ramidulus

(Brischke, 1880)

Limneria ramidula Brischke, 1880

##### Distribution

England

#### Campoplex raschkiellae

Horstmann, 1980

##### Distribution

England, Scotland

##### Notes

added by Horstmann (1980a)

#### Campoplex restrictor

Aubert, 1960

##### Distribution

England

##### Notes

added by Shaw (1981)

#### Campoplex rothii

(Holmgren, 1860)

Limneria rothii Holmgren, 1860

##### Distribution

England, Scotland

#### Campoplex rufipes

Gravenhorst, 1829

angulatus (Thomson, 1887, *Omorga*) synonymy by Horstmann (2000d)

##### Distribution

England, Scotland, Ireland

#### Campoplex striatus

Horstmann, 1985

##### Distribution

England

##### Notes

BMNH, det. Horstmann, added here

#### Campoplex sulcatus

Horstmann, 1985

##### Distribution

England, Scotland, Isle of Man

##### Notes

added by Horstmann (1985)

#### Campoplex tibialis

(Szépligeti, 1916)

Nemeritis tibialis Szépligeti, 1916
dioszeghyi (Kiss, 1929, *Omorgus*)
corsicator Aubert, 1960

##### Distribution

England, Wales

##### Notes

BMNH, det. Horstmann, added here; also some Scottish specimens in NMS, tentatively identified as this species by K. Horstmann.

#### Campoplex tumidulus

Gravenhorst, 1829

ensator misident.
nigrifemur (Seyrig, 1928, *Omorgus*)
rufinator Aubert, 1971

##### Distribution

England, Scotland, Wales, Ireland, Isle of Man

##### Notes

Recorded as *Campoplex rufinator* by Shaw (1984) and Shaw and Aeschliman (1994).

#### Campoplex tussilaginis

Horstmann, 2013

##### Distribution

England

##### Notes

added by Horstmann (2013)

#### Campoplex unicingulatus

(Schmiedeknecht, 1909)

Omorgus unicingulatus Schmiedeknecht, 1909

##### Distribution

England, Scotland, Isle of Man

##### Notes

added by Horstmann (1985)

#### Campoplex variabilis

(Bridgman, 1886)

Limneria variabilis Bridgman, 1886

##### Distribution

England

#### Campoplex volubilis

(Holmgren, 1860)

Limneria volubilis Holmgren, 1860
anterior Aubert, 1960]

##### Distribution

Scotland

##### Notes

NMS, det. Horstmann, added here

#### Casinaria

Holmgren, 1859

AMORPHOTA Förster, 1869
ANEMPHERES Förster, 1869
CAMPOTREPHUS Förster, 1869
HOROGENES Förster, 1869
NOTHANOMALON Szépligeti, 1905
TROPHOCAMPA Schmiedeknecht, 1907
CASINARIODES Aubert, 1960

#### Casinaria affinis

Tschek, 1871

##### Distribution

Ireland

#### Casinaria albipalpis

(Gravenhorst, 1829)

Campoplex albipalpis Gravenhorst, 1829

##### Distribution

Ireland

#### Casinaria ischnogaster

Thomson, 1887

##### Distribution

England, Ireland

#### Casinaria moesta

(Gravenhorst, 1829)

Campoplex moestus Gravenhorst, 1829
maesta Dalla Torre, 1901 preocc.

##### Distribution

England

##### Notes

added by Horstmann (2000d)

#### Casinaria morionella

Holmgren, 1860

##### Distribution

England

#### Casinaria pallipes

Brischke, 1880

pallidipes Dalla Torre, 1901

##### Distribution

England, Ireland

#### Casinaria petiolaris

(Gravenhorst, 1829)

Mesoleptus petiolaris Gravenhorst, 1829
claviventris Holmgren, 1860

##### Distribution

England

#### Casinaria tenuiventris

(Gravenhorst, 1829)

Campoplex tenuiventris Gravenhorst, 1829
conica (Ratzeburg, 1844, *Campoplex*)
latifrons Holmgren, 1860
protensa Thomson, 1887

##### Distribution

England

#### Charops

Holmgren, 1859

#### Charops cantator

(DeGeer, 1778)

Ichneumon cantator DeGeer, 1778
decipiens (Gravenhorst, 1829, *Campoplex*)
tenuitarsus (Gravenhorst, 1829, *Anomalon*)
nigropetiolatus Strobl, 1904
nigrifacies (Kiss, 1924, *Anomalon*)

##### Distribution

England, Wales

#### Clypeoplex

Horstmann, 1987

#### Clypeoplex cerophagus

(Gravenhorst, 1829)

Campoplex cerophagus Gravenhorst, 1829
picticrus (Thomson, 1887, *Omorga*)

##### Distribution

England, Scotland

##### Notes

BMNH, NMS, UM, added here

#### Cymodusa

Holmgren, 1859

SAGARITIS Holmgren, 1859 preocc.
THERSITIA Schmiedeknecht, 1907
SAGARITOPSIS Hincks, 1944

#### Cymodusa antennator

Holmgren, 1860

flavipes Brischke, 1880
anntenatrix Schulz, 1906

##### Distribution

England, Scotland, Ireland

#### Cymodusa cruentata

(Gravenhorst, 1829)

Campoplex cruentata Gravenhorst, 1829
marginella (Zetterstedt, 1838, *Porizon*)
longicalcar Thomson, 1887

##### Distribution

Scotland, Ireland

#### Cymodusa declinator

(Gravenhorst, 1829)

Campoplex declinator Gravenhorst, 1829
fasciata (Bridgman & Fitch, 1885, *Thymaris*)
fasciata (Bridgman, 1886, *Thymaris*) preocc.
declinatrix (Schulz, 1906, *Sagaritis*)

##### Distribution

England, Scotland

##### Notes

The name *declinator* Grav. was applied to a species of *Campoletis* by J.F. Perkins in his curation of the BMNH collection.

#### Cymodusa exilis

Holmgren, 1860

petulans Holmgren, 1860
convergens (Thomson, 1887, *Nemeritis*)

##### Distribution

England, Ireland

#### Cymodusa leucocera

Holmgren, 1859

pulchricornis Szépligeti, 1901
egregia (Schmiedeknecht, 1907, *Thersitia*)

##### Distribution

England, Scotland, Ireland

#### Diadegma

Förster, 1869

ANGITIA Holmgren, 1859
NYTHOBIA Förster, 1869
PECTINELLA Morley, 1915
NEOANGITIA Horstmann, 1969
AUMA Dbar, 1984

##### Notes

Distribution data for some species (the ‘*Nythobia*’ group) taken from Shaw and Horstmann (1997).

#### Diadegma aculeatum

(Bridgman, 1889)

Limneria aculeata Bridgman, 1889
atrum (Kokujev, 1915, *Angitia*)
politor (Aubert, 1960, *Horogenes*)

##### Distribution

England, Wales

#### Diadegma agile

(Brischke, 1880)

Limneria agilis Brischke, 1880

#### Diadegma angitiaeforma

Horstmann, 1969

##### Distribution

England, Scotland

##### Notes

NMS, det. Horstmann, added here

#### Diadegma angulator

(Aubert, 1963)

Horogenes angulator Aubert, 1963

##### Distribution

England, Ireland

##### Notes

NMS, det. Horstmann, added here

#### Diadegma annulicrus

(Thomson, 1887)

Angitia annulicrus Thomson, 1887

#### Diadegma anurum

(Thomson, 1887)

Angitia anura Thomson, 1887

##### Distribution

England, Scotland

##### Notes

added by Shaw and Horstmann (1997)

#### Diadegma areolare

(Holmgren, 1860)

Limneria areolaris Holmgren, 1860

##### Distribution

Ireland

##### Notes

Added by O'Connor et al. (2007) det. J.F. Perkins, also recorded from the Isle of Man by Walker (1872) but this may not be reliable.

#### Diadegma argentellae

Horstmann, 2004

##### Distribution

Scotland

##### Notes

added by Horstmann (2004b); described in the subgenus *Nythobia* but subgenera have not been used here.

#### Diadegma armillatum

(Gravenhorst, 1829)

Campoplex armillatus Gravenhorst, 1829
tibiale (Gravenhorst, 1829, *Campoplex*)
pseudocombinatum (Szépligeti, 1916, *Angitia*)

##### Distribution

England, Scotland, Wales, Ireland

##### Notes

*Diadegma monospilum* (Thomson, 1887, *Angitia*) was removed from *synonymy* by Horstmann (2006a).

#### Diadegma berberatae

Horstmann, 2013

##### Distribution

England

##### Notes

added by Horstmann (2013)

#### Diadegma brevipetiolatum

Horstmann, 1969

##### Distribution

England

##### Notes

NMS, det. Horstmann, added here

#### Diadegma brevivalve

(Thomson, 1887)

Angitia brevivalvis Thomson, 1887

##### Distribution

England

##### Notes

NMS, det. Horstmann, added here

#### Diadegma callisto

Horstmann, 1993

##### Distribution

Scotland

##### Notes

NMS, det. Horstmann, added here

#### Diadegma chrysostictos

(Gmelin, 1790)

Ichneumon chrysostictos Gmelin, 1790
corsicator (Aubert, 1961, *Horogenes*)
orientator Aubert, 1965

##### Distribution

England, Scotland, Ireland

##### Notes

distribution data from Horstmann and Shaw (1984)

#### Diadegma claripenne

(Thomson, 1887)

Angitia claripennis Thomson, 1887

##### Distribution

Ireland

##### Notes

added by O'Connor et al. (2007); also a specimen lacking locality data in BMNH, det. J.F. Perkins.

#### Diadegma clavicorne

(Brischke, 1880)

Limneria clavicornis Brischke, 1880

#### Diadegma coleophorarum

(Ratzeburg, 1852)

Campoplex coleophorarum Ratzeburg, 1852

##### Distribution

England, Scotland

#### Diadegma combinatum

(Holmgren, 1860)

Limneria combinata Holmgren, 1860
alpinator Aubert, 1970

##### Distribution

England, Scotland, Ireland

#### Diadegma compunctellae

Horstmann, 2013

##### Distribution

Scotland

##### Notes

added by Horstmann (2013)

#### Diadegma consumtor

(Gravenhorst, 1829)

Campoplex consumtor Gravenhorst, 1829
varians (Brischke, 1880, *Limneria*)

#### Diadegma crassicorne

(Gravenhorst, 1829)

Campoplex crassicornis Gravenhorst, 1829
carnifex (Gravenhorst, 1829, *Campoplex*)
brevicorne (Holmgren, 1860, *Limneria*)
normannicum (Rudow, 1883, *Limneria*)

##### Distribution

England, Scotland, Wales, Ireland

##### Notes

Listed as a species of *Meloboris* in Fitton (1978); some distribution data from Horstmann (2000d).

#### Diadegma crassiseta

(Thomson, 1887)

Angitia crassiseta Thomson, 1887

##### Distribution

England

##### Notes

BMNH, det. Perkins, added here

#### Diadegma crassum

(Bridgman, 1889)

Limneria crassa Bridgman, 1889

##### Distribution

England, Scotland

#### Diadegma crataegi

Horstmann, 1980

##### Distribution

England, Scotland

##### Notes

added by Horstmann (1980a)

#### Diadegma cylindricum

(Brischke, 1880)

Limneria cylindrica Brischke, 1880

##### Distribution

England, Ireland

##### Notes

Noted as occurring in England and Ireland by various authors but not listed by Fitton (1978).

#### Diadegma duplicatum

Horstmann, 1980

##### Distribution

England, Scotland

##### Notes

added by Shaw and Horstmann (1997)

#### Diadegma elishae

(Bridgman, 1884)

Limneria elishae Bridgman, 1884

##### Distribution

England, Scotland, Ireland

#### Diadegma ericinellae

Horstmann, 2013

##### Distribution

England

##### Notes

added by Horstmann (2013)

#### Diadegma erucator

(Zetterstedt, 1838)

Porizon erucator Zetterstedt, 1838
rufipes misident.
fumipennis (Holmgren, 1856, *Campoplex*)

##### Distribution

England, Scotland, Ireland

#### Diadegma exareolator

Aubert, 1964

##### Distribution

England, Scotland, Ireland

##### Notes

added by Shaw and Horstmann (1997)

#### Diadegma fabricianae

Horstmann & Shaw, 1984

##### Distribution

England, Scotland, Wales

##### Notes

added by Horstmann and Shaw (1984)

#### Diadegma fenestrale

(Holmgren, 1860)

Limneria fenestralis Holmgren, 1860
Diadegma fenestrale ?*gracile* (Gravenhorst, 1829, *Campoplex*)

##### Distribution

England, Scotland, Wales, Ireland, Isle of Man

##### Notes

distribution data from Azidah et al. (2000) and NMS

#### Diadegma flexum

Horstmann, 1973

##### Distribution

England

##### Notes

NMS, det. Horstmann, added here

#### Diadegma fungicola

Horstmann, 2008

##### Distribution

England

##### Notes

added by Horstmann (2008b)

#### Diadegma grisescens

(Gravenhorst, 1829)

Mesoleptus grisescens Gravenhorst, 1829
rufiventris (Gravenhorst, 1829, *Campoplex*)
hydropota (Holmgren, 1860, *Limneria*)

##### Distribution

England, Scotland, Ireland

#### Diadegma holopygum

(Thomson, 1887)

Angitia holopyga Thomson, 1887

##### Distribution

England, Scotland, Ireland

#### Diadegma hygrobium

(Thomson, 1887)

Meloboris hygrobia Thomson, 1887
ischnocerum (Thomson, 1887, *Meloboris*)
pechlaneri (Hedwig, 1957, *Angitia*) unavailable

##### Distribution

England, Scotland, Wales, Ireland

#### Diadegma incompletum

Horstmann, 1973

##### Distribution

Scotland

##### Notes

NMS, det. Horstmann, added here

#### Diadegma insectator

(Schrank, 1781)

Ichneumon insectator Schrank, 1781
insectatrix (Schulz, 1906, *Angitia*)

##### Distribution

England, Ireland

#### Diadegma kyffhusanae

Horstmann, 1973

##### Distribution

England

##### Notes

Tentative identification.

#### Diadegma laricinellum

(Strobl, 1904)

Angitia laricinella Strobl, 1904

##### Distribution

Scotland

##### Notes

added by Shaw and Horstmann (1997)

#### Diadegma laterale

(Gravenhorst, 1829)

Campoplex lateralis Gravenhorst, 1829

##### Distribution

England, Ireland

##### Notes

There is one specimen labelled as ‘?*lateralis*’ by K. Horstmann in NMS, from the Isle of Coll. and specimens in NMI det. A.W. Stelfox (O'Connor et al. 2007).

#### Diadegma latungulum

(Thomson, 1887)

Angitia latungula Thomson, 1887
deletum (Morley, 1915, *Pectinella*)

##### Distribution

England, Scotland, Wales, Ireland

#### Diadegma ledicola

Horstmann, 1969

##### Distribution

England, Wales

##### Notes

added by Shaw and Horstmann (1997)

#### Diadegma lithocolletis

Horstmann, 1969

##### Distribution

England, Scotland

##### Notes

added by Shaw and Horstmann (1997)

#### Diadegma litorale

(Holmgren, 1856)

Campoplex litoralis Holmgren, 1856

##### Distribution

England, Ireland

#### Diadegma majale

(Gravenhorst, 1829)

Campoplex majalis Gravenhorst, 1829

##### Distribution

England, Scotland, Ireland

#### Diadegma melanium

(Thomson, 1887)

Angitia melania Thomson, 1887

##### Distribution

England

#### Diadegma monospilum

(Thomson, 1887)

Angitia monospila Thomson, 1887

##### Distribution

England, Scotland, Wales

##### Notes

NMS, det. Horstmann, added here

#### Diadegma nanus

(Gravenhorst, 1829)

Campoplex nanus Gravenhorst, 1829

##### Distribution

England, Scotland

#### Diadegma naryciae

Horstmann, 2008

narcyiae misspelling

##### Distribution

England, Scotland

##### Notes

NMS, det. Horstmann, added here; originally, incorrectly, spelt *narcyiae*, emended by Horstmann (2012a).

#### Diadegma neocerophagum

Horstmann, 1969

cerophaga misident.

#### Diadegma neomajale

Horstmann, 1969

##### Distribution

England

##### Notes

BMNH, det. Horstmann, added here

#### Diadegma pusio

(Holmgren, 1860)

Meloboris pusio Holmgren, 1860
annulipes (Bridgman, 1889, *Limneria*)

##### Distribution

England, Scotland

#### Diadegma rufatum

(Bridgman, 1884)

Limneria rufata Bridgman, 1884

##### Distribution

England, Scotland

#### Diadegma ruficeps

(Holmgren, 1860)

Limneria ruficeps Holmgren, 1860
gracile (Ratzeburg, 1848, *Campoplex*) preocc.
rimator (Thomson, 1887, *Angitia*)
rimatrix (Schulz, 1906, *Angitia*)

##### Distribution

England, Scotland

##### Notes

BMNH, NMS, det. Horstmann, added here; there is also a specimen in NMS, from Co. Clare, Ireland, labelled ?*ruficeps* by K. Horstmann.

#### Diadegma scotiae

(Bridgman, 1889)

Limneria scotiae Bridgman, 1889

##### Distribution

Scotland

#### Diadegma semiclausum

(Hellén, 1949)

Limneria semiclausa Hellén, 1949
tibialis misident.
cerophaga misident.
eucerophagum Horstmann, 1969

##### Distribution

England, Scotland, Ireland, Isle of Man

##### Notes

distribution data from Azidah et al. (2000)

#### Diadegma sordipes

(Thomson, 1887)

Angitia sordipes Thomson, 1887

##### Distribution

England, Scotland, Wales

#### Diadegma stagnale

(Holmgren, 1856)

Campoplex stagnalis Holmgren, 1856

##### Distribution

England, Ireland

#### Diadegma stigmatellae

Horstmann, 1980

##### Distribution

England, Scotland

##### Notes

added by Horstmann (1980a)

#### Diadegma tenuipes

(Thomson, 1887)

Angitia tenuipes Thomson, 1887

##### Distribution

England, Scotland, Ireland

#### Diadegma tripunctatum

(Bridgman, 1886)

Limneria tripunctata Bridgman, 1886

##### Distribution

England, Ireland

#### Diadegma trochanteratum

(Thomson, 1887)

Angitia trochanterata Thomson, 1887

##### Distribution

England, Ireland

#### Diadegma truncatum

(Thomson, 1887)

Campoplex truncata Thomson, 1887
subbuccatum (Thomson, 1887, *Angitia*)

##### Distribution

England, Wales, Ireland

#### Dolophron

Förster, 1869

#### Dolophron pedellum

(Holmgren, 1860)

Limneria pedella Holmgren, 1860
albicoxis (Schmiedeknecht, 1909, *Synetaeris*)

##### Distribution

England, Scotland, Isle of Man

#### Dusona

Cameron, 1901

DELOPIA Cameron, 1903

##### Notes

Most distribution data from Horstmann (2011a), based on R. Hinz’s and K. Horstmann’s identifications of specimens in NMS and BMNH; older determinations of material in BMNH cannot be relied upon. Some species have been carried over from the 1978 checklist but no recent specimens have been seen, these are indicated by a ‘?’. Synonymy follows Hinz and Horstmann (2004) and Horstmann (2009c).

Species of *Dusona* excluded from the British and Irish list:

[*limnobia* (Thomson, 1887, *Campoplex*)] K. Horstmann had identified specimens in NMS as *limnobia*, which appeared on earlier versions of this checklist, but, following the redescription of the species (Horstmann 2009c), older identifications need to be checked (Horstmann 2011a).

[*vidua* (Gravenhorst, 1829, *Campoplex*)] Specimens identified in BMNH as *Dusona vidua* are actually *Hyposoter tricolor* (det. K. Horstmann); Shaw (2008) also notes that Irish records almost certainly refer to *H. tricolor*.

#### Dusona admontina

(Speiser, 1908)

Campoplex admontinus Speiser, 1908
rufiventris (Strobl, 1904, *Campoplex*) preocc.

##### Distribution

England, Scotland

##### Notes

added by Horstmann (2011a)

#### Dusona aemula

(Förster, 1868)

Campoplex aemulus Förster, 1868
discrepans (Förster, 1868, *Campoplex*)
dissepta (Förster, 1868, *Campoplex*)
parvula (Förster, 1868, *Campoplex*) preocc.
filicornis (Holmgren, 1872, *Campoplex*)

##### Distribution

England, Scotland, Wales

##### Notes

added by Horstmann (2011a)

#### Dusona alpigena

Hinz, 1972

##### Distribution

Scotland

##### Notes

Tentative identification of a specimen in NMS by K. Horstmann.

#### Dusona anceps

(Holmgren, 1860)

Campoplex anceps Holmgren, 1860
auriculata (Förster, 1868, *Campoplex*)
disparilis (Förster, 1868, *Campoplex*)
costulata (Bridgman & Fitch, 1885, *Campoplex*)
costulata (Bridgman, 1886, *Campoplex*)
libauensis (Strand, 1918, *Campoplex*)

##### Distribution

England

#### Dusona angustata

(Thomson, 1887)

Campoplex angustatus Thomson, 1887

##### Distribution

England, Scotland

#### Dusona angustifrons

(Förster, 1868)

Campoplex angustifrons Förster, 1868
Dusona angustifrons ?*obreptans* (Förster, 1868, *Campoplex*)
zonella (Förster, 1868, *Campoplex*)
cornella (Teunissen, 1947, *Campoplex*)

##### Distribution

England, Scotland, Wales

#### Dusona annexa

(Förster, 1868)

Campoplex annexus Förster, 1868
limnobia (Thomson, 1887, *Campoplex*)
facialis (Holmgren, 1872, *Campoplex*) preocc.
americana (Ashmead, 1890, *Casinaria*)
mariae (Dalla Torre, 1901, *Campoplex*)
oyamadai Hinz, 1994

##### Distribution

England, Scotland, Ireland, Isle of Man

#### Dusona aurita

(Kriechbaumer, 1883)

Campoplex auritus Kriechbaumer, 1883

##### Distribution

England

##### Notes

added by Horstmann (2011a)

#### Dusona aversa

(Förster, 1868)

Campoplex aversus Förster, 1868
dubiosa (Förster, 1868, *Campoplex*)
tschekii (Holmgren, 1872, *Campoplex*)
crassipes (Thomson, 1887, *Campoplex*)

##### Distribution

England, Scotland

#### Dusona bellipes

(Holmgren, 1872)

Campoplex bellipes Holmgren, 1872
vernalis Hinz, 1957

##### Distribution

England

##### Notes

added by Horstmann (2011a)

#### Dusona bicoloripes

(Ashmead, 1906)

Campoplex bicoloripes Ashmead, 1906
pugillator misident.
foersteri (Roman, 1942, *Campoplex*)

##### Distribution

England, Scotland

#### Dusona blanda

(Förster, 1868)

Campoplex blandus Förster, 1868
remota (Förster, 1868, *Campoplex*)
forsselli (Holmgren, 1872, *Campoplex*)
punctiventris (Woldstedt, 1877, *Casinaria*)

##### Distribution

England, Scotland, Isle of Man

#### Dusona bucculenta

(Holmgren, 1860)

Campoplex bucculentus Holmgren, 1860
melampus (Förster, 1868, *Campoplex*)

##### Distribution

England, Scotland

#### Dusona carinifrons

(Holmgren, 1860)

Campoplex carinifrons Holmgren, 1860
minax (Förster, 1868, *Campoplex*)
geometrae (Rudow, 1883, *Campoplex*) synonymy by Horstmann (1999a)

##### Distribution

England

#### Dusona carpathica

(Szépligeti, 1916)

Casinaria carpathica Szépligeti, 1916
zonella misident.
adriaansei (Teunissen, 1947, *Campoplex*)

##### Distribution

England, Scotland

##### Notes

added by Horstmann (2011a)

#### Dusona circumcinctus

(Förster, 1868)

Campoplex circumcinctus Förster, 1868
subcinctus (Förster, 1868, *Campoplex*) preocc.

##### Distribution

England

##### Notes

added by Horstmann (2011a)

#### Dusona circumspectans

(Förster, 1868)

Campoplex circumspectans Förster, 1868
vagula (Förster, 1868, *Campoplex*)
subsulcata (Holmgren, 1872, *Campoplex*)

##### Distribution

England, Scotland

##### Notes

added by Horstmann (2011a)

#### Dusona confusa

(Förster, 1868)

Campoplex confusus Förster, 1868
lacunosa (Kriechbaumer, 1883, *Campoplex*)
consimilis (Schmiedeknecht, 1908, *Campoplex*)

##### Distribution

England, Scotland, Wales, Isle of Man

#### Dusona cultrator

(Gravenhorst, 1829)

Campoplex cultrator Gravenhorst, 1829

##### Distribution

England

#### Dusona disclusa

(Förster, 1868)

Campoplex disclusus Förster, 1868

##### Distribution

England, Scotland

##### Notes

added by Horstmann (2011a)

#### Dusona dubitor

Hinz, 1977

oxyacanthae misident.

##### Distribution

England, Scotland

##### Notes

Added by Horstmann (2011a); frequently misidentified as *oxyacanthae* (K. Horstmann, pers. comm.)

#### Dusona erythrogaster

(Förster, 1868)

Campoplex erythrogaster Förster, 1868
indefessa (Förster, 1868, *Campoplex*)

##### Distribution

England, Scotland

#### Dusona falcator

(Fabricius, 1775)

Ichneumon falcator Fabricius, 1775

##### Distribution

England

#### Dusona flagellator

(Fabricius, 1793)

Ichneumon flagellator Fabricius, 1793
debilis (Förster, 1868, *Campoplex*) synonymy by Horstmann (1999a)
heterocera (Förster, 1868, *Campoplex*) synonymy by Horstmann (2001a)

##### Distribution

England

##### Notes

added by Horstmann (2011a)

#### Dusona holmgrenii

(Dalla Torre, 1901)

Campoplex Dalla Torre, 1901
unicincta (Holmgren, 1872, *Campoplex*) preocc.

#### Dusona humilis

(Förster, 1868)

Campoplex humilis Förster, 1868
eurynotus (Holmgren, 1872, *Campoplex*)

##### Distribution

England, Scotland

##### Notes

added by Horstmann (2011a)

#### Dusona incompleta

(Bridgman, 1889)

Campoplex incompletus Bridgman, 1889

##### Distribution

England, Scotland

#### Dusona inermis

(Förster, 1868)

Campoplex inermis Förster, 1868

##### Distribution

England, Scotland

##### Notes

added by Horstmann (2011a)

#### Dusona infesta

(Förster, 1868)

Campoplex infestus Förster, 1868
terrifica (Förster, 1868, *Campoplex*)

##### Distribution

Scotland, Wales

#### Dusona insignita

(Förster, 1868)

Campoplex insignitus Förster, 1868
bistrigosa (Holmgren, 1872, *Campoplex*)

##### Distribution

England, Scotland

#### Dusona juvenilis

(Förster, 1868)

Campoplex juvenilis Förster, 1868
victor (Thunberg, 1824, *Ichneumon*) preocc.
monozona (Förster, 1868, *Campoplex*)

##### Distribution

England, Wales

#### Dusona leptogaster

(Holmgren, 1860)

Campoplex leptogaster Holmgren, 1860
macrostylus (Förster, 1868, *Campoplex*)
sylvicola (Habermehl, 1922, *Campoplex*)

##### Distribution

England, Scotland

#### Dusona libertatis

(Teunissen, 1947)

Campoplex libertatis Teunissen, 1947

##### Distribution

England

##### Notes

added by Horstmann (2011a)

#### Dusona mercator

(Fabricius, 1793)

Ichneumon mercator Fabricius, 1793
venditor (Thunberg, 1824, *Ichneumon*)
oxyacanthae (Boie, 1855,)
mesoxantha (Förster, 1868, *Campoplex*)

##### Distribution

England, Scotland

#### Dusona minor

(Provancher, 1879)

Campoplex minor Provancher, 1879

##### Distribution

England

##### Notes

added by Horstmann (2011a)

#### Dusona montana

(Roman, 1929)

Campoplex montanus Roman, 1929
carinifer (Teunissen, 1947, *Campoplex*)

##### Distribution

England

##### Notes

added by Horstmann (2011a)

#### Dusona myrtilla

(Desvignes, 1856)

Campoplex myrtillus Desvignes, 1856
tenthredinum (Tschek, 1871, *Campoplex*)
nobilitata (Holmgren, 1872, *Campoplex*)

##### Distribution

England

#### Dusona nidulator

(Fabricius, 1804)

Ophion nidulator Fabricius, 1804
nitidulator (Holmgren, 1856, *Campoplex*)
circumscripta (Förster, 1868, *Campoplex*)
martialis (Förster, 1868, *Campoplex*)
vindex (Förster, 1868, *Campoplex*)
bifida (Thomson, 1887, *Campoplex*)
obscura (Kiss, 1926, *Campoplex*)

##### Distribution

England, Scotland

#### Dusona notabilis

(Förster, 1868)

Campoplex notabilis Förster, 1868
scolator misident.

##### Distribution

England, Scotland

#### Dusona obliterata

(Holmgren, 1872)

Campoplex obliteratus Holmgren, 1872
limniventris (Kriechbaumer, 1883, *Campoplex*)

##### Distribution

England

##### Notes

added by Horstmann (2011a)

#### Dusona opaca

(Thomson, 1887)

Campoplex opacus Thomson, 1887

#### Dusona petiolator

(Fabricius, 1804)

Ophion petiolator Fabricius, 1804
lapponica (Holmgren, 1860, *Campoplex*)
callizona (Förster, 1868, *Campoplex*)
punctata (Bridgman & Fitch, 1885, *Campoplex*)
punctata (Bridgman, 1886, *Campoplex*) preocc.
nigra (Kiss, 1924, *Campoplex*)

##### Distribution

England, Scotland

#### Dusona pineticola

(Holmgren, 1872)

Campoplex pineticola Holmgren, 1872
litigiosa (Habermehl, 1922, *Campoplex*)
sibirica Hinz, 1985

##### Distribution

England, Scotland

##### Notes

added by Horstmann (2011a)

#### Dusona polita

(Förster, 1868)

Campoplex politus Förster, 1868
flavipalpis (Förster, 1868, *Campoplex*)
mediana (Förster, 1868, *Campoplex*)
spoliator (Förster, 1868, *Campoplex*)
trisculpta (Holmgren, 1872, *Campoplex*)
femorator (Bridgman & Fitch, 1885, *Campoplex*)
femorator (Bridgman, 1886, *Campoplex*) preocc.
latungula (Thomson, 1887, *Campoplex*)
splendens (Thomson, 1887, *Campoplex*)

##### Distribution

England, Scotland

#### Dusona prominula

(Förster, 1868)

Campoplex prominulus Förster, 1868
contumax (Förster, 1868, *Campoplex*)
foveolata (Förster, 1868, *Campoplex*)

##### Distribution

England, Scotland

#### Dusona pugillator

(Linnaeus, 1758)

Ichneumon pugillator Linnaeus, 1758
canaliculata (Förster, 1868, *Campoplex*) preocc.

##### Distribution

England, Scotland

#### Dusona pulchripes

(Holmgren, 1872)

Campoplex pulchripes Holmgren, 1872
praecox (Teunissen, 1947, *Campoplex*)

##### Distribution

England, Ireland

##### Notes

added by Horstmann (2011a)

#### Dusona recta

(Thomson, 1887)

Campoplex rectus Thomson, 1887

##### Distribution

England

##### Notes

added by Horstmann (2011a)

#### Dusona rubidatae

Horstmann, 2009

##### Distribution

Scotland

##### Notes

added by Horstmann (2011a)

#### Dusona rugifer

(Förster, 1868)

Campoplex rugifer Förster, 1868
subaequalis (Förster, 1868, *Campoplex*)
puncta (Kriechbaumer, 1883, *Campoplex*)

##### Distribution

England, Scotland

##### Notes

English record from W.A. Ely (pers. comm.).

#### Dusona rugulosa

(Förster, 1868)

Campoplex rugulosus Förster, 1868

#### Dusona semiflava

(Costa, 1883)

Campoplex semiflavus Costa, 1883
flaviscapus (Thomson, 1887, *Campoplex*)

##### Distribution

England

##### Notes

added by Horstmann (2011a)

#### Dusona sobolicida

(Förster, 1868)

Campoplex sobolicida Förster, 1868
ulcerata (Holmgren, 1872, *Campoplex*)

##### Distribution

England, Scotland, Wales

#### Dusona spinipes

(Thomson, 1887)

Campoplex spinipes Thomson, 1887

##### Distribution

England

##### Notes

added by Horstmann (2011a)

#### Dusona stenogaster

(Förster, 1868)

Campoplex stenogaster Förster, 1868
monticola (Habermehl, 1922, *Campoplex*)

##### Distribution

England

##### Notes

added by Horstmann (2011a)

#### Dusona stragifex

(Förster, 1868)

Campoplex stragifex Förster, 1868
delusor misident.
adjuncta (Förster, 1868, *Campoplex*)
areolata (Brauns, 1895, *Campoplex*)

##### Distribution

England, Scotland, Isle of Man

#### Dusona stygia

(Förster, 1868)

Campoplex stygius Förster, 1868

##### Distribution

England

##### Notes

added by Horstmann (2011a)

#### Dusona subimpressa

(Förster, 1868)

Campoplex subimpressus Förster, 1868
transitoria (Kiss, 1924, *Campoplex*)

##### Distribution

England

##### Notes

added by Horstmann (2011a)

#### Dusona tenuis

(Förster, 1868)

Campoplex tenuis Förster, 1868
agnata (Förster, 1868, *Campoplex*)
anxia (Förster, 1868, *Campoplex*)
peraffinis (Förster, 1868, *Campoplex*)
proxima (Förster, 1868, *Campoplex*)

##### Distribution

England, Scotland

#### Dusona terebrator

(Förster, 1868)

Campoplex terebrator Förster, 1868
added by

##### Distribution

England, Scotland, Wales, Ireland, Isle of Man

##### Notes

added by Horstmann (2011a)

#### Dusona thomsoni

Hinz, 1966

##### Distribution

England

##### Notes

added by Horstmann (2011a)

#### Dusona vigilator

(Förster, 1868)

Campoplex vigilator Förster, 1868

##### Distribution

England, Scotland

#### Dusona xenocampta

(Förster, 1868)

Campoplex xenocamptus Förster, 1868
polyxantha (Strobl, 1904, *Campoplex*)
baltica (Habermehl, 1926, *Campoplex*)

##### Distribution

Scotland

#### Echthronomas

Förster, 1869

#### Echthronomas facialis

(Thomson, 1887)

Anilasta facialis Thomson, 1887

##### Distribution

England

##### Notes

Horniman, det. Shaw, added here

#### Echthronomas ochrostoma

(Holmgren, 1860)

Casinaria ochrostoma Holmgren, 1860

##### Notes

BMNH, added here

#### Echthronomas tricincta

(Gravenhorst, 1829)

Campoplex tricinctus Gravenhorst, 1829

#### Enytus

Cameron, 1905

IOCTES Förster, 1869
NAREOLATA Ellinger & Sachtleben, 1928

#### Enytus apostata

(Gravenhorst, 1829)

Campoplex apostata Gravenhorst, 1829
exareolata (Ratzeburg, 1852, *Campoplex*)
reticulata (Bridgman, 1884, *Limneria*)

##### Distribution

England, Scotland, Ireland

##### Notes

distribution data from Shaw (1981)

#### Enytus appositor

(Aubert, 1970)

Diadegma appositor Aubert, 1970

##### Distribution

England, Scotland, Wales, Isle of Man

##### Notes

NMS, BMNH, det. Horstmann, added here

#### Enytus crataegellae

(Thomson, 1887)

Angitia crataegellae Thomson, 1887

##### Distribution

England

##### Notes

NMS, det. Horstmann, added here

#### Enytus neoapostata

(Horstmann, 1969)

Diadegma neoapostata Horstmann, 1969
neapostatus misspelling

##### Distribution

England, Scotland, Ireland

#### Enytus parvicanda

(Thomson, 1887)

Angitia parvicanda Thomson, 1887
parvicauda misspelling

#### Enytus styriacus

(Horstmann, 1980)

Diadegma styriacum Horstmann, 1980

##### Distribution

Scotland

##### Notes

NMS, det. Horstmann, added here

#### Eriborus

Förster, 1869

ZAPORUS Förster, 1869

##### Notes

Distribution data for *Eriborus* species from Horstmann (1987) and the collections of BMNH, NMS and UM.

#### Eriborus braccatus

(Gmelin, 1790)

Ichneumon braccatus Gmelin, 1790
jocator (Fabricius, 1793, *Ichneumon*)
nigriventris (Habermehl, 1922, *Anilasta*)

##### Distribution

England

#### Eriborus dorsalis

(Gravenhorst, 1829)

Campoplex dorsalis Gravenhorst, 1829
micorocephalus (Gravenhorst, 1829, *Ischnoceros*)

##### Distribution

England, Scotland

##### Notes

Listed twice by Fitton (1978), under *Meloboris* and *Eriborus*.

#### Eriborus perfidus

(Gravenhorst, 1829)

Campoplex perfidus Gravenhorst, 1829
aberrans (Gravenhorst, 1829, *Campoplex*)
obscuriventris Kiss, 1926

##### Distribution

England

#### Gonotypus

Förster, 1869

GONOTYPA Thomson, 1887

#### Gonotypus melanostoma

(Thomson, 1887)

Gonotypa melanostoma Thomson, 1887

##### Distribution

England, Wales, Ireland, Isle of Man

#### Hyposoter

Förster, 1869

AMELOCTONUS Förster, 1869
ISCHNOSCOPUS Förster, 1869
RHYTHMONOTUS Förster, 1869

##### Notes

species of *Hyposoter* excluded from the British and Irish list:

[*anglicanus* (Habermehl, 1923, *Anilasta*)] Only included on the British list on the basis that it was described from Carr material, of doubtful origin (see Perkins 1953, Shaw 2003).

#### Hyposoter albonotatus

(Bridgman, 1889)

Limneria albonotata Bridgman, 1889
melaleucus (Schmiedeknecht, 1909, *Anilastus*)

##### Distribution

England, Scotland

#### Hyposoter alienus

(Brischke, 1880)

Limneria aliena Brischke, 1880

##### Distribution

Ireland

#### Hyposoter barrettii

(Bridgman, 1881)

Limneria barrettii Bridgman, 1881
teucrii (Bridgman, 1889, *Limneria*)

##### Distribution

England

#### Hyposoter boops

(Thomson, 1887)

Anilasta boops Thomson, 1887

##### Distribution

England

##### Notes

Added by Horstmann (2013)and raised from synonymy with *brischkei*.

#### Hyposoter brischkei

(Bridgman, 1882)

Limneria brischkei Bridgman, 1882

##### Distribution

England, Scotland, Ireland

#### Hyposoter caedator

(Gravenhorst, 1829)

Campoplex caedator Gravenhorst, 1829
henscheli (Smits van Burgst, 1910, *Anilastus*)
persimilis (Szépligeti, 1916, *Anilastus*)
parvulus (Kiss, 1926, *Anilastus*)

##### Distribution

England

#### Hyposoter carbonarius

(Ratzeburg, 1844)

Campoplex carbonaria Ratzeburg, 1844

##### Distribution

England, Scotland

#### Hyposoter clausus

(Brischke, 1880)

Limneria clausa Brischke, 1880

##### Distribution

England, Scotland

##### Notes

Many specimens in BMNH are only doubtfully identified; material in NMS has been recently identified by K. Horstmann.

#### Hyposoter coxator

(Thomson, 1887)

Anilasta coxator Thomson, 1887

##### Distribution

England

##### Notes

BMNH, NMS, det. Horstmann, added here

#### Hyposoter didymator

(Thunberg, 1824)

Ichneumon didymator Thunberg, 1824
rotundator (Thunberg, 1824, *Ichneumon*)
ruficinctus (Gravenhorst, 1829, *Campoplex*)
schmiedeknechti (Smits van Burgst, 1913, *Anilastus*)

##### Distribution

England, Scotland, Wales, Ireland

#### Hyposoter discedens

(Schmiedeknecht, 1909)

Anilastus discedens Schmiedeknecht, 1909

##### Distribution

Ireland

#### Hyposoter dolosus

(Gravenhorst, 1829)

Campoplex dolosus Gravenhorst, 1829
rufimanus (Gravenhorst, 1829, *Campoplex*) synonymy by Horstmann (2000d)
oculatus (Tschek, 1871, *Limneria*)

##### Distribution

England, Scotland

#### Hyposoter fitchii

(Bridgman, 1881)

Limneria fitchii Bridgman, 1881

#### Hyposoter inquinatus

(Holmgren, 1860)

Limneria inquinata Holmgren, 1860

##### Distribution

Scotland

##### Notes

One British specimen (no locality) in BMNH but pointing the other direction, implying erroneous identification (by J.F. Perkins?). Carried over from the 1978 checklist and on the basis of material in UM, det. J.P. Brock.

#### Hyposoter leucomerus

(Thomson, 1887)

Anilasta leucomera Thomson, 1887
tricinctus (Holmgren, 1858, *Limneria*) unavailable

##### Distribution

England, Ireland

##### Notes

NMS, det. Horstmann, added here

#### Hyposoter longulus

(Thomson, 1887)

Anilasta longula Thomson, 1887

##### Distribution

England, Scotland, Wales

#### Hyposoter neglectus

(Holmgren, 1860)

Limneria neglecta Holmgren, 1860
varicoxa (Thomson, 1887, *Anilasta*)
variicoxus (Dalla Torre, 1901, *Anilastus*)

##### Distribution

Scotland

##### Notes

NMS, det. Horstmann, added here

#### Hyposoter notatus

(Gravenhorst, 1829)

Campoplex notatus Gravenhorst, 1829

##### Distribution

England, Scotland, Wales, Ireland

#### Hyposoter obscurellus

(Holmgren, 1860)

Limneria obscurella Holmgren, 1860

#### Hyposoter orbator

(Gravenhorst, 1829)

Campoplex orbator Gravenhorst, 1829
rufus (Bridgman, 1882, *Limneria*)

#### Hyposoter placidus

(Desvignes, 1856)

Campoplex placidus Desvignes, 1856

##### Distribution

England

#### Hyposoter rhodocerae

(Rondani, 1877)

Tryphon rhodocerae Rondani, 1877
ebeninus misident.

##### Distribution

England, Ireland

#### Hyposoter ruficrus

(Thomson, 1887)

Anilasta ruficrus Thomson, 1887

##### Distribution

England

##### Notes

NMS, det. Horstmann, added here

#### Hyposoter thuringiacus

(Schmiedeknecht, 1909)

Anilastus thuringiacus Schmiedeknecht, 1909

##### Distribution

England, Ireland

#### Hyposoter tricolor

(Ratzeburg, 1844)

Campoplex tricolor Ratzeburg, 1844
vidua misident.
henaultii (Desvignes, 1856, *Campoplex*)

##### Distribution

England, Scotland

#### Hyposoter virginalis

(Gravenhorst, 1829)

Campoplex virginalis Gravenhorst, 1829

##### Distribution

England

##### Notes

distribution data from Horstmann (2000d)

#### Hyposoter vividus

(Holmgren, 1860)

Limneria vivida Holmgren, 1860
albicrus (Thomson, 1877, *Anilasta*)

##### Distribution

England

##### Notes

BMNH, added here

#### Lathroplex

Förster, 1869

#### Lathroplex clypearis

Thomson, 1887

##### Distribution

England

##### Notes

NMS, det. Horstmann, added here

#### Lathrostizus

Förster, 1869

LATHROSTIZA Thomson, 1887

#### Lathrostizus clypeatus

(Brischke, 1880)

Limneria clypeata Brischke, 1880
sternocerus (Thomson, 1887, *Lathrostiza*) synonymy by Horstmann (2004b)

##### Distribution

Scotland

#### Lathrostizus lugens

(Gravenhorst, 1829)

Campoplex lugens Gravenhorst, 1829
vestigialis (Ratzeburg, 1852, *Campoplex*)

##### Distribution

England, Ireland, Isle of Man

#### Lemophagus

Townes, 1965

HOLOCREMNODES Aubert, 1986 synonymy by Horstmann (2004b)

#### Lemophagus curtus

Townes, 1965

##### Distribution

England, Wales

##### Notes

added by Cox (2007); specimens in BMNH; NMS

#### Lemophagus errabundus

(Gravenhorst, 1829)

Campoplex errabundus Gravenhorst, 1829

##### Distribution

England

##### Notes

added by Salisbury (2003)

#### Leptocampoplex

Horstmann, 1970

#### Leptocampoplex cremastoides

(Holmgren, 1860)

Nemeritis cremastoides Holmgren, 1860

##### Distribution

England, Scotland

##### Notes

NMS, BMNH, added here; Horstmann (1999a) removed *punctulatus* (Ratze.) to a synonym of *Porizon moderator* (L.).

#### Macrus

Gravenhorst, 1829

#### Macrus parvulus

(Gravenhorst, 1829)

Campoplex parvulus Gravenhorst, 1829
fusicornis (Roman, 1923, *Lathroplex*)

##### Distribution

England, Scotland

#### Melanoplex

Horstmann, 1987

#### Melanoplex bucculentus

(Holmgren, 1860)

Limneria bucculentus Holmgren, 1860

##### Distribution

England

##### Notes

BMNH, det. Horstmann, added here

#### Meloboris

Holmgren, 1859

ASINAMORA Förster, 1869
NEPIERA Förster, 1869
PSEUDOCYMODUSA Habermehl, 1922
ANOIXIS Townes, 1970

#### Meloboris alternans

(Gravenhorst, 1829)

Campoplex alternans Gravenhorst, 1829
elachistae (Brischke, 1880, *Cymodusa*)
ruficornis (Bridgman, 1884, *Limneria*) preocc.

##### Distribution

England, Scotland, Ireland

#### Meloboris cingulata

Horstmann, 2004

##### Distribution

Scotland

##### Notes

added by Horstmann (2004b)

#### Meloboris collector

(Thunberg, 1824)

Ichneumon collector Thunberg, 1824
concinna (Holmgren, 1860, *Limneria*)
signata (Szépligeti, 1916, *Omorgus*)
foersteri (Kiss, 1924, *Idechthis*)
albicincta (Seyrig, 1927, *Angitia*)

##### Distribution

England, Scotland, Wales, Ireland, Isle of Man

#### Meloboris gracilis

Holmgren, 1859

monticolana (Bridgman, 1881, *Limneria*)

##### Distribution

England, Scotland, Ireland

#### Meloboris neglecta

(Habermehl, 1923)

Pseudocymodusa neglecta Habermehl, 1923

#### Meloboris proxima

(Perkins, 1942)

Nepiera proxima Perkins, 1942

##### Distribution

England, Scotland, Ireland

##### Notes

BMNH, NMS, det. Horstmann, added here

#### Nemeritis

Holmgren, 1860

PSEUDONEMERITIS Szépligeti, 1916

##### Notes

Distribution data from Horstmann (1994a).

#### Nemeritis breviventris

Horstmann, 1975

##### Distribution

England

##### Notes

BMNH, det. Horstmann, added here

#### Nemeritis caudatula

Thomson, 1887

rhaphidiae Kriechbaumer, 1892
raphidiae Dalla Torre, 1901
monticola Habermehl, 1922

##### Distribution

England

#### Nemeritis cingulata

Horstmann, 1980

##### Distribution

England

#### Nemeritis fallax

(Gravenhorst, 1829)

Campoplex fallax Gravenhorst, 1829
crassiceps Habermehl, 1922

##### Distribution

England

##### Notes

NMS, BMNH, det. Horstmann, added here

#### Nemeritis lativentris

Thomson, 1887

##### Distribution

England

#### Nemeritis macrocentra

(Gravenhorst, 1829)

Campoplex macrocentrus Gravenhorst, 1829
sordida (Gravenhorst, 1829, *Campoplex*)
varipes (Gravenhorst, 1829, *Campoplex*)
antennalis (Szépligeti, 1916, *Angitia*)
caudata (Szépligeti, 1916, *Omorgus*) preocc.
transsylvanica (Szépligeti, 1916, *Canidia*)
caudata (Gregor, 1940, *Idechthis*)

##### Distribution

England, Ireland

#### Nemeritis silvicola

Horstmann, 1973

##### Distribution

England

#### Nemeritis stenura

Thomson, 1887

##### Notes

One English specimen in NMS tentatively identified by K. Horstmann; carried over from Fitton (1978).

#### Nepiesta

Förster, 1869

#### Nepiesta mandibularis

(Holmgren, 1860)

Limneria mandibularis Holmgren, 1860
aberrans misident.
umbrata (Brischke, 1880, *Canidia*)
nigra Szépligeti, 1901

##### Distribution

England, Scotland, Ireland

#### Nepiesta subclavata

Thomson, 1887

##### Distribution

England

##### Notes

NMS, det Horstmann, added here

#### Nepiesta tricingulata

Horstmann, 1973

##### Distribution

England

##### Notes

NMS, det Horstmann, added here

#### Olesicampe

Förster, 1869

LIMNERIA Förster, 1859
HOLOCREMNUS Förster, 1869
OMOBORUS Förster, 1869
HOLOCREMNA Thomson, 1887
OLESICAMPA Thomson, 1887

##### Notes

Species of *Olesicampe* excluded from the British and Irish list:

[*monticola* (Hedwig, 1938, *Holocremna*)] Welsh specimens in BMNH, ex *Cephalcia alpina* (Hymenoptera: Pamphiliidae) (not a British native), det. K. Horstmann and I.D. Gauld, presumably brought over for biocontrol purposes.

[*ratzeburgi* (Tschek, 1871, *Limneria*)] Released in Wales for biocontrol purposes but with no evidence of successful establishment (Billany et al. 1983).

Doubtfully placed species of *Olesicampe*:

[*affinis* (Parfitt, 1882, *Limneria*) nom. dub.]

[*alienata* (Gravenhorst, 1829, *Campoplex*) nom. dub.]

#### Olesicampe alboplica

(Thomson, 1887)

Olesicampa alboplica Thomson, 1887
simplex (Thomson, 1887, *Olesicampa*)

##### Distribution

England, Ireland

#### Olesicampe argentata

(Gravenhorst, 1829)

Campoplex argentatus Gravenhorst, 1829

##### Distribution

Scotland

#### Olesicampe auctor

(Gravenhorst, 1829)

Campoplex auctor Gravenhorst, 1829
limbata (Gravenhorst, 1829, *Campoplex*)
auctrix (Schulz, 1906, *Olesicampa*)

##### Distribution

England

#### Olesicampe binotata

(Thomson, 1887)

Olesicampa binotata Thomson, 1887

##### Distribution

England, Scotland

##### Notes

BMNH, det. Perkins, added here

#### Olesicampe buccata

(Thomson, 1887)

Holocremna buccata Thomson, 1887

##### Distribution

Ireland

#### Olesicampe canaliculata

(Gravenhorst, 1829)

Campoplex canaliculatus Gravenhorst, 1829

##### Distribution

Isle of Man

##### Notes

NMS, det. Horstmann, added here

#### Olesicampe cavigena

(Thomson, 1887)

Olesicampa cavigena Thomson, 1887

#### Olesicampe clandestina

(Holmgren, 1860)

Limneria clandestina Holmgren, 1860

##### Distribution

England, Ireland

##### Notes

distribution data from Shaw (1999)

#### Olesicampe crassitarsis

(Thomson, 1887)

Olesicampa crassitarsis Thomson, 1887

##### Distribution

Scotland, Ireland

#### Olesicampe erythropyga

(Holmgren, 1860)

Limneria erythropyga Holmgren, 1860

##### Distribution

Ireland

#### Olesicampe femorella

(Thomson, 1887)

Olesicampa femorella Thomson, 1887

##### Distribution

England

##### Notes

BMNH, det. Perkins, added here

#### Olesicampe forticostata

(Schmiedeknecht, 1909)

Anilastus forticostatus Schmiedeknecht, 1909

##### Distribution

England

#### Olesicampe fulcrans

(Thomson, 1887)

Olesicampa fulcrans Thomson, 1887

##### Distribution

England, Scotland, Ireland

#### Olesicampe fulviventris

(Gmelin, 1790)

Ichneumon fulviventris Gmelin, 1790

##### Distribution

England, Ireland

#### Olesicampe geniculella

(Thomson, 1887)

Olesicampa geniculella Thomson, 1887

##### Distribution

Scotland, Ireland

#### Olesicampe gracilipes

(Thomson, 1887)

Olesicampa gracilipes Thomson, 1887

##### Distribution

Ireland

#### Olesicampe longipes

(Müller, 1776)

Ichneumon longipes Müller, 1776
canescens (Gmelin, 1790, *Ichneumon*)

##### Distribution

Ireland

#### Olesicampe macellator

(Thunberg, 1824)

Ichneumon macellator Thunberg, 1824
retecta (Hartig, 1838, *Campoplex*)
cothurnata (Holmgren, 1860, *Limneria*)
frutetorum (Thomson, 1887, *Holocremna*)

##### Distribution

England, Scotland

#### Olesicampe nigroplica

(Thomson, 1887)

Olesicampa nigroplica Thomson, 1887

##### Distribution

England, Scotland

#### Olesicampe pagana

(Holmgren, 1860)

Limneria pagana Holmgren, 1860

#### Olesicampe paludicola

(Holmgren, 1860)

Limneria paludicola Holmgren, 1860
inculcator misident.
Olesicampe paludicola ?*sagittaria* (Müller, 1776, *Ichneumon*)

##### Distribution

England, Ireland

#### Olesicampe patellana

(Thomson, 1887)

Olesicampa patellana Thomson, 1887

##### Distribution

Scotland, Ireland

##### Notes

BMNH, det. Perkins, added here

#### Olesicampe praecox

(Holmgren, 1860)

Limneria praecox Holmgren, 1860

##### Distribution

Ireland

#### Olesicampe proterva

(Brischke, 1880)

Limneria proterva Brischke, 1880
luteipes (Thomson, 1887, *Olesicampa*)
subcallosa (Thomson, 1887, *Olesicampa*)

##### Distribution

Ireland

#### Olesicampe pubescens

(Ratzeburg, 1844)

Campoplex pubescens Ratzeburg, 1844
hyalinata (Holmgren, 1860, *Limneria*)

##### Distribution

Ireland

#### Olesicampe retusa

(Thomson, 1887)

Olesicampa retusa Thomson, 1887

#### Olesicampe sericea

(Holmgren, 1856)

Campoplex sericeus Holmgren, 1856

##### Distribution

England, Ireland

#### Olesicampe sinuata

(Thomson, 1887)

Holocremna sinuata Thomson, 1887

##### Distribution

England, Scotland

##### Notes

BMNH, det. Perkins, UM, det. Brock, added here

#### Olesicampe transiens

(Ratzeburg, 1848)

Campoplex transiens Ratzeburg, 1848
incrassator (Holmgren, 1856, *Campoplex*) synonymy by Horstmann (2007a)

##### Distribution

England, Scotland, Wales, Ireland

#### Olesicampe vexata

(Holmgren, 1860)

Limneria vexata Holmgren, 1860

#### Olesicampe vitripennis

(Holmgren, 1860)

Limneria vitripennis Holmgren, 1860

#### Phobocampe

Förster, 1869

HYPOTHEREUTES Förster, 1869
PHOBOCAMPA Thomson, 1887

#### Phobocampe alticollis

(Thomson, 1887)

Phobocampa alticollis Thomson, 1887

##### Distribution

England

##### Notes

added by Horstmann (2006a)

#### Phobocampe bicingulata

(Gravenhorst, 1829)

Campoplex bicingulatus Gravenhorst, 1829

##### Distribution

England, Scotland, Wales

#### Phobocampe brumatae

Horstmann, 2009

##### Distribution

England, Scotland

##### Notes

added by Horstmann (2006a)

#### Phobocampe confusa

(Thomson, 1887)

Phobocampa confusa Thomson, 1887

##### Distribution

England, Scotland, Ireland

##### Notes

Omitted by Fitton (1978) as it had previously only been recorded from Ireland (Stelfox 1929), although it was also discussed as a British species by Shaw and Askew (1976).

#### Phobocampe coniferella

(Roman, 1914)

Phobocampa coniferella Roman, 1914
facialis (Szépligeti, 1916, *Holocremnus*)

##### Distribution

England

##### Notes

added by Šedivý (2004); Britain given as a locality by Šedivý (2004) but the specimens in BMNH are destroyed, leaving only the parasitoid cocoons; however there are English specimens in NMS, det. K. Horstmann.

#### Phobocampe crassiuscula

(Gravenhorst, 1829)

Campoplex crassiusculus Gravenhorst, 1829

##### Distribution

England, Scotland

#### Phobocampe croceipes

(Marshall, 1876)

Limneria croceipes Marshall, 1876
albitarsis Szépligeti, 1916

##### Distribution

England, Scotland

#### Phobocampe flavicincta

(Thomson, 1887)

Phobocampa flavicincta Thomson, 1887

##### Distribution

Scotland

##### Notes

BMNH, det. Perkins, added here

#### Phobocampe horstmanni

Šedivý, 2004

##### Distribution

England, Scotland

##### Notes

NMS, det. Horstmann, added here

#### Phobocampe lymantriae

Gupta, 1983

##### Distribution

England

##### Notes

NMS, det. Horstmann, added here

#### Phobocampe neglecta

(Holmgren, 1860)

Limneria neglecta Holmgren, 1860
varicoxa (Thomson, 1887, *Anilasta*)
variicoxa (Dalla Torre, 1901, *Anilastus*)

##### Distribution

England, Scotland

#### Phobocampe nigra

Šedivý, 2004

##### Distribution

England

##### Notes

BMNH, det. Horstmann, added here

#### Phobocampe pulchella

(Thomson, 1887)

Phobocampa pulchella Thomson, 1887

##### Distribution

England, Scotland

##### Notes

NMS, det. Horstmann, added here

#### Phobocampe quercus

Horstmann, 2008

##### Distribution

England

##### Notes

added by Horstmann (2008b)

#### Phobocampe tempestiva

(Holmgren, 1860)

Limneria tempestiva Holmgren, 1860

##### Distribution

England, Scotland

##### Notes

NMS, det. Horstmann, added here

#### Phobocampe unicincta

(Gravenhorst, 1829)

Campoplex unicinctus Gravenhorst, 1829
disparis (Viereck, 1911, *Hyoposoter*)

##### Distribution

England

#### Phobocampe variabilis

Šedivý, 2004

##### Distribution

England

##### Notes

NMS, det. Horstmann, added here

#### Porizon

Fallén, 1813

PHAEDROCTONUS Förster, 1869 synonymy by Horstmann (2004b)

#### Porizon humuli

(Horstmann, 1987)

Phaedroctonus humuli Horstmann, 1987

##### Distribution

England

##### Notes

NMS, det. Horstmann, added here

#### Porizon moderator

(Linnaeus, 1758)

Ichneumon moderator Linnaeus, 1758
strobinellae (Christ, 1791, *Cynipsichneumon*)
flaviventris (Ratzeburg, 1844, *Campoplex*)
punctulatus (Ratzeburg, 1844, *Cremastus*) synonymy by Horstmann (1999a)
ensifer (Brischke, 1880, *Limneria*)

##### Distribution

England, Scotland

#### Porizon transfuga

(Gravenhorst, 1829)

Campoplex transfuga Gravenhorst, 1829
syringellae Hedwig, 1944

##### Distribution

England, Scotland, Wales, Ireland, Isle of Man

#### Pyracmon

Holmgren, 1859

#### Pyracmon fumipennis

(Zetterstedt, 1838)

Porizon fumipennis Zetterstedt, 1838

##### Distribution

Ireland

#### Pyracmon sepiellus

(Holmgren, 1860)

Limneria sepiella Holmgren, 1860

##### Distribution

England

##### Notes

BMNH, det. Horstmann, added here

#### Rhimphoctona

Förster, 1869

PARAPYRACMON Clément, 1924

#### Xylophylax

Kriechbaumer, 1878

#### Rhimphoctona (Xylophylax) megacephalus

(Gravenhorst, 1829)

Campoplex megacephalus Gravenhorst, 1829
corvina (Gravenhorst, 1829, *Phytodietus*)
austriaca (Tschek, 1871, *Pyracmon*)
megalocephalus (Schulz, 1906, *Campoplex*)
rufipes (Lange, 1911, *Pyracmon*) preocc.
hungarica (Kiss, 1926, *Pyracmon*)

##### Distribution

England

#### Rhimphoctona (Xylophylax) melanura

(Holmgren, 1860)

Pyracmon melanurus Holmgren, 1860
signata (Habermehl, 1922, *Pyracmon*)

#### Rhimphoctona (Xylophylax) obscuripes

(Holmgren, 1860)

Pyracmon obscuripes Holmgren, 1860
alpina (Strobl, 1904, *Pyracmon*)

##### Distribution

Ireland

#### Rhimphoctona (Xylophylax) xoridiformis

(Holmgren, 1860)

Pyracmon xoridiformis Holmgren, 1860
nigerrima (Kiss, 1924, *Eclytus*)

#### Scirtetes

Hartig, 1838

SPUDASTICA Förster, 1869

#### Scirtetes robustus

(Woldstedt, 1874)

Limneria robusta Woldstedt, 1874
kriechbaumeri (Bridgman, 1882, *Limneria*)
petiolaris (Thomson, 1887, *Spudastica*)

##### Distribution

England, Scotland, Ireland

#### Sinophorus

Förster, 1869

EULIMNERIA Schmiedeknecht, 1907

##### Notes

doubtfully placed species of *Sinophorus*:

[*paniscus* (Gravenhorst, 1829, *Campoplex*) nom. dub.] Morley (1915) identified *Sinophorus paniscus* as British but this name is now a synonym of *Macrus filiventris* Gravenhorst, 1829 (Horstmann 1978a), a rarely collected southern European species; the identity of Morley’s specimens is not known.

#### Sinophorus albidus

(Gmelin, 1790)

Ichneumon albidus Gmelin, 1790
hungaricus (Szépligeti, 1916, *Omorgus*)

##### Distribution

England, Scotland

#### Sinophorus bridgmanii

(Dalla Torre, 1901)

Limnerium bridgmanii Dalla Torre, 1901
distinctus (Bridgman, 1887, *Limneria*) preocc.
renominatus (Morley, 1915, *Limnerium*)

##### Distribution

England

#### Sinophorus costalis

(Thomson, 1887)

Limneria costalis Thomson, 1887

##### Distribution

Ireland

#### Sinophorus crassifemur

(Thomson, 1887)

Limneria crassifemur Thomson, 1887

##### Distribution

Ireland

#### Sinophorus fuscicarpus

(Thomson, 1887)

Limneria fuscicarpus Thomson, 1887

##### Distribution

England

##### Notes

added by Sanborne (1984)

#### Sinophorus geniculatus

(Gravenhorst, 1829)

Campoplex geniculatus Gravenhorst, 1829
nigritellus (Thomson, 1887, *Limneria*)
argentator (Aubert, 1960, *Campoplex*)

##### Distribution

Ireland

#### Sinophorus juniperinus

(Holmgren, 1856)

Campoplex juniperinus Holmgren, 1856
ornatus (Gregor, 1941, *Omorgus*)

##### Distribution

England, Ireland

##### Notes

added by Sanborne (1984)

#### Sinophorus pleuralis

(Thomson, 1887)

Limneria pleuralis Thomson, 1887

##### Distribution

England

##### Notes

added by Sanborne (1984)

#### Sinophorus turionum

(Ratzeburg, 1844)

Campoplex turionum Ratzeburg, 1844
spectabilis (Rudow, 1883, *Limneria*)
planiscapus (Thomson, 1887, *Limneria*)
rufifemur (Thomson, 1887, *Limneria*)
nigrotibialis (Kiss, 1926, *Eulimneria*)
alkae (Ellinger & Sachtleben, 1928, *Limnerium*)

##### Distribution

England, Scotland, Ireland

##### Notes

some distribution data from Sanborne (1984)

#### Sinophorus xanthostomus

(Gravenhorst, 1829)

Campoplex xanthostomus Gravenhorst, 1829
pineticola (Thomson, 1887, *Limneria*)
deserticola (Tosquinet, 1896, *Campoplex*)

#### Synetaeris

Förster, 1869

#### Synetaeris heteropus

Thomson, 1887

##### Distribution

Scotland

#### Tranosema

Förster, 1869

#### Tranosema carbonellum

(Thomson, 1887)

Synetaeris carbonella Thomson, 1887
aterrimum (Strobl, 1904, *Pyracmon*)
rossicum (Szépligeti, 1916, *Canidia*)

##### Distribution

England, Scotland

##### Notes

BMNH, det. Broad, Perkins, added here

#### Tranosema exoletum

(Thomson, 1887)

Omorga exoleta Thomson, 1887
geniculatum (Ulbricht, 1910, *Omorgus*) unavailable

##### Distribution

England

##### Notes

BMNH, det. Perkins, UM, added here

#### Tranosema hyperboreum

(Thomson, 1887)

Limneria hyperborea Thomson, 1887

##### Distribution

England

##### Notes

NMS, det. Horstmann, added here

#### Tranosema intermedium

(Szépligeti, 1916)

Gonotypus intermedius Szépligeti, 1916
majus (Szépligeti, 1916, *Gonotypus*)
minus (Szépligeti, 1916, *Gonotypus*)

##### Distribution

England

##### Notes

NMS, det. Horstmann, added here

#### Tranosema latiusculum

(Thomson, 1887)

Omorga latiuscula Thomson, 1887

##### Distribution

England, Scotland

##### Notes

NMS, BMNH, det. Horstmann, added here

#### Tranosema nigridens

(Thomson, 1887)

Omorga nigridens Thomson, 1887
striolatum (Thomson, 1887, *Omorga*)
alpinator Aubert, 1966

##### Distribution

England

##### Notes

added by Horstmann (1978a)

#### Tranosema rostrale

(Brischke, 1880)

Limneria rostralis Brischke, 1880
arenicola Thomson, 1887
thuringiacum (Schmiedeknecht, 1907, *Sinophorus*)

##### Distribution

England, Scotland, Wales

#### Tranosemella

Horstmann, 1978

#### Tranosemella citrofrontalis

(Hedwig, 1939)

Anilasta citrofrontalis Hedwig, 1939

##### Distribution

England, Scotland, Wales, Ireland

##### Notes

BMNH, NMS, det. Horstmann, Shaw, Broad, added here

#### Tranosemella coxalis

(Brischke, 1880)

Limneria coxalis Brischke, 1880

##### Distribution

England

##### Notes

One British specimen in BMNH is probably *coxalis*; another British specimen has been labelled '*robusta* Wold.' (=*Scirtetes*) and an English specimen has been labelled as '*interruptus*'. Locality data from reared specimens in NMS, det. K. Horstmann.

#### Tranosemella praerogator

(Linnaeus, 1758)

Ichneumon praerogator Linnaeus, 1758
chrysogaster (Gmelin, 1790, *Ichneumon*) preocc.
mandibulator (Thunberg, 1824, *Ichneumon*)
interrupta (Holmgren, 1858, *Limneria*)
laticrus (Thomson, 1887, *Angitia*)

##### Distribution

England, Scotland, Wales, Ireland, Isle of Man

##### Notes

some distribution data from Shaw (1981)

#### Venturia

Schrottky, 1902

IDECHTHIS Förster, 1869
DEVORGILLA Cameron, 1907
NEMERITIS misident.

#### Venturia canescens

(Gravenhorst, 1829)

Campoplex canescens Gravenhorst, 1829
frumentaria (Rondani, 1874, *Campoplex*)
orientalis (Schmiedeknecht, 1909, *Omorgus*)
compressa (Hedwig, 1962, *Angitia*)

##### Distribution

England, Scotland, Ireland

### Collyriinae

#### COLLYRIINAE

Cushman, 1924

##### Notes

Distribution data from Fitton (1984) and the collections of NMS.

#### Collyria

Schiødte, 1839

PACHYMERUS Gravenhorst, 1829

#### Collyria coxator

(Villers, 1789)

Ichneumon coxator Villers, 1789
Collyria coxator ?*falcata* (Geoffroy, 1785, *Ichneumon*)
Collyria coxator ?*arcuata* (Olivier, 1792, *Ichneumon*)
calcitrator (Gravenhorst, 1807, *Bassus*)
puncticeps (Thomson, 1877, *Pachymerus*)
calcitratrix Schulz, 1906

##### Distribution

England

#### Collyria trichophthalma

(Thomson, 1877)

Pachymerus trichophthalmus Thomson, 1877

##### Distribution

England

### Cremastinae

#### CREMASTINAE

Förster, 1869

##### Notes

Distribution data from Fitton and Gauld (1980) and the collections of NMS and BMNH.

#### Cremastus

Gravenhorst, 1829

##### Notes

species excluded from the British and Irish list by Fitton and Gauld (1980):

[*crassicornis* Thomson, 1890]

#### Cremastus bellicosus

Gravenhorst, 1829

partitus Szépligeti, 1899
meridionator Aubert, 1960

##### Distribution

England

#### Cremastus cephalotes

Šedivý, 1970

ponticus Kolarov, 1982

##### Distribution

England

#### Cremastus geminus

Gravenhorst, 1829

areolaris Strand, 1918

##### Distribution

England, Scotland, Wales, Ireland

#### Cremastus infirmus

Gravenhorst, 1829

filicaudis Szépligeti, 1905

##### Distribution

England, Ireland

#### Cremastus kratochvili

Šedivý, 1970

##### Distribution

England

##### Notes

added by Fitton and Gauld (1980)

#### Cremastus pungens

Gravenhorst, 1829

laeviusculus Thomson, 1890

##### Distribution

England, Ireland

#### Cremastus spectator

Gravenhorst, 1829

binotatus Gravenhorst, 1829
melanarius Szépligeti, 1901

##### Distribution

England, Wales, Ireland

#### Dimophora

Förster, 1869

DIMOPHORUS Thomson, 1889

#### Dimophora nitens

(Gravenhorst, 1829)

Campoplex nitens Gravenhorst, 1829
robusta Brischke, 1880
similis Brischke, 1880
arenicola (Thomson, 1890, *Dimophorus*)

##### Distribution

England

#### Pristomerus

Curtis, 1836

#### Pristomerus armatus

(Lucas, 1849)

Collyria armata Lucas, 1849
glandarius (Rondani, 1877, *Odontomerus*)
gratiosus Tosquinet, 1896
cingulatus Szépligeti, 1905

##### Distribution

England

##### Notes

NMS, det. Shaw & Narolsky, added here

#### Pristomerus horribilis

Narolsky, 1987

##### Distribution

England

##### Notes

BMNH, det. Broad, added here

#### Pristomerus vulnerator

(Panzer, 1799)

Ichneumon vulnerator Panzer, 1799
marginalis Habermehl, 1923
stigmaticus Hellén, 1949

##### Distribution

England, Ireland

#### Temelucha

Förster, 1869

PARACREMASTUS Szépligeti, 1899

##### Notes

species excluded from the British and Irish list by Fitton and Gauld (1980):

[*decorata* (Gravenhorst, 1829, *Cremastus*)]

[*subnasuta* (Thomson, 1890, *Cremastus*)]

#### Temelucha arenosa

(Szépligeti, 1899)

Cremastus arenosus Szépligeti, 1899

##### Distribution

England, Ireland

##### Notes

added by Fitton and Gauld (1980)

#### Temelucha interruptor

(Gravenhorst, 1829)

Cremastus interruptor Gravenhorst, 1829
buoliana (Curtis, 1854, *Cremastus*)

##### Distribution

England

#### Temelucha ophthalmica

(Holmgren, 1860)

Cremastus ophthalmicus Holmgren, 1860

##### Distribution

England

##### Notes

added by Fitton and Gauld (1980)

#### Temelucha signata

(Holmgren, 1860)

Cremastus signatus Holmgren, 1860

##### Distribution

England, Ireland

##### Notes

added by Fitton and Gauld (1980)

### Cryptinae

#### CRYPTINAE

Kirby, 1837

PHYGADEUONTINAE Förster, 1869
GELINAE Viereck, 1918
HEMITELINAE Förster, 1869

#### CRYPTINI

Kirby, 1837

MESOSTENINI Ashmead, 1900
ECHTHRINI Narayanan & Kundanlal, 1958

##### Notes

Unless noted otherwise, distribution data from Schwarz and Shaw (1998) and the collections of BMNH and UM, with further references given.

#### Acroricnus

Ratzeburg, 1852

XENODOCON Förster, 1855
MACROBATUS Holmgren, 1856
LINOCERAS Taschenberg, 1865
LEPTOBATIDES Buysson, 1896

#### Acroricnus stylator

(Thunberg, 1824)

Ichneumon stylator Thunberg, 1824
macrobatus (Gravenhorst, 1829, *Cryptus*)
schaumii Ratzeburg, 1852
clavator (Holmgren, 1856, *Macrobatus*)
exannulatus (Kriechbaumer, 1894, *Linoceras*) unavailable

##### Distribution

England, Ireland

#### Agrothereutes

Förster, 1850

SPILOCRYPTUS Thomson, 1873

#### Agrothereutes abbreviatus

(Fabricius, 1794)

Ichneumon abbreviatus Fabricius, 1794
abbreviator (Fabricius, 1793, *Ichneumon*) misident.
abbreviator (Fabricius, 1798, *Ichneumon*) preocc.
breviator (Thunberg, 1824, *Ichneumon*)
marginellus (Gravenhorst, 1829, *Cryptus*) synonymy by Horstmann (2001b)
pygoleucus (Gravenhorst, 1829, *Cryptus*)
tibiator (Gravenhorst, 1829, *Cryptus*)
ocellator (Zetterstedt, 1838, *Cryptus*)
evanescens (Ratzeburg, 1852, *Cryptus*)
leucomerus (Ratzeburg, 1852, *Cryptus*)
dispar (Thomson, 1873, *Spilocryptus*)
destitutus Vollenhoven, 1879
spectabilis (Rudow, 1886, *Aptesis*)
tricolor (Rudow, 1886, *Aptesis*)
brevipennis (Kriechbaumer, 1893, *Spilocryptus*) preocc.
spectabilis (Rudow, 1914, *Aptesis*) preocc.
atratus (Rudow, 1917, *Stibeutes*) preocc.
livonensis (Rudow, 1917, *Aptesis*)
spectabilis (Rudow, 1917, *Aptesis*) preocc.
tricolor (Rudow, 1917, *Aptesis*)
cingulatus (Kiss, 1924, *Gambrus*)
variegatus (Kiss, 1924, *Gambrus*) preocc.
alpium Heinrich, 1951 synonymy by Schwarz (2005)

##### Distribution

England, Wales, Ireland, Isle of Man

##### Notes

Schwarz and Shaw (1998) separately list the distributions of three forms of *abbreviatus*, f. *brevipennis* (Marshall, 1867, *Cryptus*) (syn. *batavus* Vollenhoven, 1873), f. *hopei* (Gravenhorst, 1829, *Pezomachus*) and f. *incubitor* (Gravenhorst, 1829, *Cryptus*).

#### Agrothereutes adustus

(Gravenhorst, 1829)

Cryptus adustus Gravenhorst, 1829
albolineatus (Gravenhorst, 1829, *Cryptus*)
nubeculatus (Gravenhorst, 1829, *Cryptus*)
opisoleucus (Gravenhorst, 1829, *Cryptus*)
leucostictus (Hartig, 1838, *Cryptus*)
melanocerus (Ulbricht, 1916, *Spilocryptus*) unavailable

#### Agrothereutes aterrimus

(Gravenhorst, 1829)

Cryptus aterrimus Gravenhorst, 1829
bicingulatus (Gravenhorst, 1829, *Cryptus*)

#### Agrothereutes fumipennis

(Gravenhorst, 1829)

Cryptus fumipennis Gravenhorst, 1829
zygaenarum (Thomson, 1873, *Spilocryptus*)
hymotomadum (Rudow, 1883, *Cryptus*)
nigricans (Kiss, 1915, *Spilocryptus*)
gracilentus (Habermehl, 1929, *Spilocryptus*)

##### Distribution

England, Wales

#### Agrothereutes hospes

(Tschek, 1871)

Cryptus hospes Tschek, 1871
solitarius (Tschek, 1871, *Cryptus*)
intermedius (Verhoeff, 1890, *Cryptus*) preocc.

#### Agrothereutes leucorhaeus

(Donovan, 1810)

Ichneumon leucorhaeus Donovan, 1810
migrator misident.
bombycis (Boudier, 1836, *Cryptus*)
Agrothereutes leucorhaeus ?*tibialis* (Thomson, 1873, *Spilocryptus*)
temporalis (Szépligeti, 1916, *Gambrus*) synonymy by Schwarz (2005)

##### Distribution

England, Scotland

##### Notes

*Spilocryptus tibialis* was tentatively synonymised by Schwarz and Shaw (1998) but treated as a valid species (occuring in England) by M. Schwarz in his identifications of BMNH specimens.

#### Agrothereutes mandator

(Linnaeus, 1758)

Ichneumon mandator Linnaeus, 1758
ischioleucus (Gravenhorst, 1829, *Cryptus*) synonymy by Schwarz (2005)
cimbicis (Tschek, 1871, *Cryptus*)

##### Distribution

England, Scotland

#### Agrothereutes mansuetor

(Tschek, 1871)

Cryptus mansuetor Tschek, 1871
nasutus (Thomson, 1873, *Spilocryptus*)
curiosus (Szépligeti, 1916, *Gambrus*) synonymy by Schwarz (2005)

##### Distribution

Scotland

##### Notes

added by Schwarz and Shaw (1998)

#### Agrothereutes saturniae

(Boie, 1855)

Cryptus saturniae Boie, 1855
pavoniae (Bauer, 1937, *Spilocryptus*)

##### Distribution

England, Scotland, Ireland

#### Apsilops

Förster, 1869

DAPANUS Förster, 1869
HETEROTYPUS Förster, 1869
SOBAS Förster, 1869
TRICHOCRYPTUS Thomson, 1873

#### Apsilops aquaticus

(Thomson, 1874)

Trichocrytus aquaticus Thomson, 1874
napiformis (Rudow, 1882, *Cryptus*)

##### Distribution

England

#### Apsilops cinctorius

(Fabricius, 1775)

Ichneumon cinctorius Fabricius, 1775
scirpi (Geoffroy, 1785, *Ichneumon*)
spinuosus (Rudow, 1886, *Phygadeuon*)

##### Distribution

England, Wales

#### Aritranis

Förster, 1869

PYCNOCRYPTUS Thomson, 1873

##### Notes

Schwarz and Shaw (1998) clarified the uses of the generic names *Aritranis*, *Hoplocryptus* and *Pycnocryptus*.

#### Aritranis director

(Thunberg, 1824)

Ichneumon director Thunberg, 1824
peregrinator misident.

##### Distribution

England, Scotland, Wales, Ireland, Isle of Man

#### Aritranis nigripes

(Gravenhorst, 1829)

Cryptus nigripes Gravenhorst, 1829
fuscomarginatus (Gravenhorst, 1829, *Cryptus*)
insectator (Tschek, 1871, *Cryptus*)
jonicus (Tschek, 1872, *Cryptus*)

##### Distribution

England

#### Aritranis occisor

(Gravenhorst, 1829)

Cryptus occisor Gravenhorst, 1829
gracilis (Taschenberg, 1865, *Cryptus*) preocc.
fuscicornis (Tschek, 1871, *Cryptus*)
notabilis (Habermehl, 1926, *Hoplocryptus*)
punguri (Kiss, 1915, *Spilocryptus*)

##### Notes

Listed as a valid species by Yu and Horstmann (1997), as a synonym of *nigripes* by Schwarz and Shaw (1998) and then taken out of synonymy with *nigripes* by Sawoniewicz (2003) and treated as a valid species, with revised synonymy of both species, by Schwarz (2005).

#### Ateleute

Förster, 1869

ATELEUTA Schulz, 1906

#### Ateleute linearis

Förster, 1871

lissonotoides (Thomson, 1885, *Hemiteles*)
egregia (Schmiedeknecht, 1933, *Hemiteles*)

##### Distribution

England

##### Notes

added by Schwarz and Shaw (1998)

#### Buathra

Cameron, 1903

#### Buathra laborator

(Thunberg, 1824)

Ichneumon laborator Thunberg, 1824
fabricii (Schiødte, 1839, *Cryptus*)
fulvipes (Magretti, 1884, *Cryptus*) synonymy by Horstmann (2004c)
fulvipes (Habermehl, 1902, *Cryptus*)

##### Distribution

England, Scotland, Wales

#### Buathra tarsoleuca

(Schrank, 1781)

Ichneumon tarsoleucos Schrank, 1781
leucopus (Gmelin, 1790, *Ichneumon*)
leucotarsos (Gmelin, 1790, *Ichneumon*)
curvicauda (Thomson, 1896, *Cryptus*)

##### Distribution

England, Scotland

#### Caenocryptus

Thomson, 1873

#### Caenocryptus rufiventris

(Gravenhorst, 1829)

Cryptus rufiventris Gravenhorst, 1829
eborinus (Ratzeburg, 1852, *Cryptus*)
collaris (Rudow, 1883, *Cryptus*) preocc.

##### Distribution

England, Scotland

##### Notes

British specimens belong to the subspecies *impunctatus* Schwarz, 1991 (Schwarz and Shaw 1998).

#### Cryptus

Fabricius, 1804

EUCRYPTUS Haldeman, 1842
ITAMOPLEX Förster, 1869

#### Cryptus apparitorius

(Villers, 1789)

Ichneumon apparitorius Villers, 1789
pungens Gravenhorst, 1829
gratiosus Tschek, 1871
histrionicus Rudow, 1882

#### Cryptus arenicola

Thomson, 1873

##### Distribution

England

##### Notes

added by Schwarz (2005), as *Cryptus macellus* Tschek, 1871 with *arenicola* synonymised under *macellus* but later Schwarz (2015) raised *arenicola* from synonymy and confirmed that British specimens are *arenicola*.

#### Cryptus armator

Fabricius, 1804

albatorius misident.
cunctator (Fabricius, 1793, *Ichneumon*) synonymy by Horstmann (2001a)
rusticator Zetterstedt, 1838
filicornis Rudow, 1886 preocc.

##### Distribution

England, Scotland, Wales

#### Cryptus dianae

Gravenhorst, 1829

gracilicornis Gravenhorst, 1829
leucostomus Gravenhorst, 1829
stenogaster Gravenhorst, 1829
seticornis (Ratzeburg, 1844, *Ichneumon*)
bolivari Kriechbaumer, 1898
solitarius Habermehl, 1909 preocc.
solitarius Habermehl, 1918 preocc.

##### Distribution

England

#### Cryptus fibulatus

Gravenhorst, 1829

rhenanus Ulbricht, 1911
antennalis Szépligeti, 1916

##### Distribution

Scotland, Wales, Ireland

##### Notes

added by Schwarz and Shaw (1998)

#### Cryptus inculcator

(Linnaeus, 1758)

Ichneumon inculcator Linnaeus, 1758
sponsor (Fabricius, 1793, *Ichneumon*)
regenerator (Panzer, 1804, *Ichneumon*)
quadrilineatus Gravenhorst, 1829
filicornis Ratzeburg, 1844
bicolor Rudow, 1882 preocc.
erythrostoma Rudow, 1882
lippensis Rudow, 1883
albopictus Seyrig, 1928 preocc.

#### Cryptus minator

Gravenhorst, 1829

#### Cryptus moschator

(Fabricius, 1787)

Ichneumon moschator Fabricius, 1787
polytropus Heinrich, 1951 synonymy by Schwarz (2005)

##### Distribution

England

#### Cryptus obscuripes

Zetterstedt, 1838

borealis Thomson, 1873 preocc.
carpathicus Szépligeti, 1916

##### Distribution

England

##### Notes

BMNH, det. Schwarz, added here

#### Cryptus spinosus

Gravenhorst, 1829

armatorius misident.
leucostictus Gravenhorst, 1829 synonymy by Horstmann (2001b)

##### Distribution

England

##### Notes

Schwarz and Shaw (1998) list *Ichneumon armatorius* Fabricius, 1787, as a synonym of *Cryptus spinosus* but Horstmann (Horstmann 1982, Horstmann 2001a) treats it as a species of *Hoplismenus* (Ichneumoninae), tentatively as a synonym of *albifrons* (Gravenhorst) (as *axillatorius* (Thunberg))

#### Cryptus spiralis

(Geoffroy, 1785)

Ichneumon spiralis Geoffroy, 1785
inconspicuus Gravenhorst, 1829
hispanicus Habermehl, 1918

#### Cryptus titubator

(Thunberg, 1824)

Ichneumon titubator Thunberg, 1824
difficilis Tschek, 1871
infumatus Thomson, 1873

##### Distribution

England, Scotland, Wales, Ireland, Isle of Man

#### Cryptus tuberculatus

Gravenhorst, 1829

investigator Tschek, 1871
solivagus Rossem, 1989

##### Distribution

England

#### Cryptus viduatorius

Fabricius, 1804

germari Taschenberg, 1865

##### Distribution

England, Scotland, Wales, Ireland

#### Echthrus

Gravenhorst, 1829

SPHAETES Bremi, 1849

##### Notes

*Echthrus* is placed in the Cryptini, following Laurenne et al. (2006), rather than in the Hemigastrini, where it was placed by Townes (1970). Townes and Townes (1960) had previously placed *Echthrus* in the Cryptini based on similarities to the genera that Townes (1970) classified as the subtribe Gabuniina. The molecular results of Laurenne et al. (2006) support this interpretation, in agreement with Townes and Townes (1962).

#### Echthrus reluctator

(Linnaeus, 1758)

Ichneumon reluctator Linnaeus, 1758
usurpator (Scopoli, 1763, *Ichneumon*)
obex (Müller, 1776, *Ichneumon*)
rubiginosus (Christ, 1791, *Ichneumon*)
carbonator (Thunberg, 1824, *Ichneumon*)
chirothecator (Thunberg, 1824, *Ichneumon*)
ternator (Thunberg, 1824, *Ichneumon*)
crassicrus (Bremi, 1849, *Sphaetes*)
corsicus (Marshall, 1901, *Nyxeophilus*)
nigerrimus Strobl, 1902

##### Distribution

England

#### Enclisis

Townes, 1970

#### Enclisis alpicola

(Habermehl, 1926)

Caenocryptus alpicola Habermehl, 1926

##### Distribution

England

##### Notes

Added by Schwarz (1989); omitted from the British list bySchwarz and Shaw (1998).

#### Enclisis macilenta

(Gravenhorst, 1829)

Cryptus macilentus Gravenhorst, 1829
remex (Tschek, 1871, *Cryptus*)
inflata (Thomson, 1873, *Caenocryptus*)
gracilipes (Gravenhorst, 1829, *Cryptus*) synonymy by Sawoniewicz (2003)
antennata (Bridgman, 1881, *Cryptus*)
laticrus (Thomson, 1896, *Caenocryptus*)
exareolata (Strobl, 1901, *Chaeretymma*)
rubi (Habermehl, 1921, *Microcryptus*)
alboclypeata (Kiss, 1924, *Hoplocryptus*)

##### Distribution

England, Wales

#### Enclisis ruficeps

(Desvignes, 1856)

Cryptus ruficeps Desvignes, 1856
pulchella Schwarz, 1989

#### Enclisis vindex

(Tschek, 1871)

Cryptus vindex Tschek, 1871
pubiventris (Thomson, 1873, *Caenocryptus*)
tener (Thomson, 1873, *Caenocryptus*)
nubifer (Thomson, 1896, *Caenocryptus*)
striolata (Thomson, 1896, *Caenocryptus*)
nigriventris (Habermehl, 1919, *Caenocryptus*)

##### Distribution

England, Scotland

#### Gambrus

Förster, 1869

KALTENBACHIA Förster, 1869
HYGROCRYPTUS Thomson, 1873

##### Notes

Synonymy follows Schwarz and Shaw (1998) and Schwarz (2005).

#### Gambrus amoenus

(Gravenhorst, 1829)

Cryptus amoenus Gravenhorst, 1829

#### Gambrus bipunctatus

(Tschek, 1872)

Cryptus bipunctatus Tschek, 1872
ornatus misident.; Schwarz and Shaw (1998)
maculatus Brischke, 1888 synonymy by Schwarz (2005)

##### Distribution

England, Scotland, Wales

##### Notes

Added by Schwarz and Shaw (1998)and transferred from *Aritranis*.

#### Gambrus carnifex

(Gravenhorst, 1829)

Cryptus carnifex Gravenhorst, 1829
varicoxis (Taschenberg, 1865, *Cryptus*)

##### Distribution

England, Scotland, Wales

#### Gambrus incubitor

(Linnaeus, 1758)

Ichneumon incubitor Linnaeus, 1758
vibex (Müller, 1776, *Ichneumon*)
upsaliensis (Geoffroy, 1785, *Ichneumon*)
superus Thomson, 1896
quadricinctus (Strobl, 1901, *Spilocryptus*)
incertus Habermehl, 1935 preocc.

##### Distribution

England, Scotland

##### Notes

Schwarz and Shaw (1998) recorded *Gambrus incubitor* (including *ornatus*) from England, Scotland and Wales but, following Schwarz’s (Schwarz 2005) separation of *incubitor* and *ornatus*, most specimens in NMS actually belong to *ornatus* (M. Schwarz, pers. comm.).

#### Gambrus ornatus

(Gravenhorst, 1829)

Cryptus ornatus Gravenhorst, 1829
ornatulus (Thomson, 1873, *Spilocryptus*)
inferus Thomson, 1896
quadricinctus (Strobl, 1901, *Spilocryptus*)
ruficoxis Habermehl, 1919
meridionator (Aubert, 1965, *Agrothereutes*) preocc.

##### Distribution

England, Scotland, Wales, Ireland

##### Notes

added by Schwarz (2005); see note under *incubitor*. Irish occurrence confirmed by a specimen (coll. A. Anderson) identified by M. Schwarz. The species referred to as *ornatus* in older literature is now known to be *bipunctatus* (Schwarz and Shaw 1998).

#### Gambrus tricolor

(Gravenhorst, 1829)

Cryptus tricolor Gravenhorst, 1829
subcinctus (Gravenhorst, 1829, *Cryptus*)
opacus Szépligeti, 1916

##### Distribution

England, Scotland, Wales

##### Notes

Listed as a species of *Thrybius* by Yu and Horstmann (1997), as a species of *Gambrus* by Schwarz and Shaw (1998).

#### Helcostizus

Förster, 1869

BRACHYCENTRUS Taschenberg, 1865 preocc.
CYRTOCRYPTUS Marshall, 1872
MESOCRYPTUS Thomson, 1873
HETEROCRYPTUS Woldstedt, 1874
CHENBERGUS Navás, 1930

##### Notes

*Helcostizus* is transferred here from the Phygadeuontini, following the molecular phylogenetic results of Laurenne et al. (2006). The combination of morphological characters associated with parasitising wood-boring hosts had previously led Townes & Townes (1962) to classify this genus in the Cryptini. Distribution data from Townes (1983) and Schwarz and Shaw (2010).

#### Helcostizus restaurator

(Fabricius, 1775)

Ichneumon restaurator Fabricius, 1775
albator (Thunberg, 1824, *Ichneumon*)
brachycentrus (Gravenhorst, 1829, *Cryptus*)
crassipes (Hartig, 1847, *Echthrus*)
hercynianus (Hartig, 1847, *Echthrus*)
pimplarius (Taschenberg, 1865, *Brachycentrus*)
fuscitarsis (Haupt, 1917, *Perosis*)
turcicus (Fahringer, 1944, *Brachycentrus*)
serraticornis (Haupt, 1954, *Perosis*)

##### Distribution

England, Ireland

#### Hidryta

Förster, 1869

BRACHYCRYPTUS Thomson, 1873
EUTHYCRYPTUS Jussel, 1907

#### Hidryta nigricoxa

(Provancher, 1888)

Cryptus nigricoxus Provancher, 1888
scrobiculifer (Jussel, 1907, *Euthycryptus*)

##### Distribution

Wales

##### Notes

added by Schwarz and Shaw (1998)

#### Hidryta sordida

(Tschek, 1871)

Cryptus sordidus Tschek, 1871
melanopus (Taschenberg, 1865, *Cryptus*) preocc., synonymy by Schwarz (2005)
erythrocera (Thomson, 1873, *Brachycryptus*)
sordidula (Thomson, 1873, *Brachycryptus*)
hueberi (Dalla Torre, 1901, *Cryptus*) synonymy by Schwarz (2005)
nigritarsis (Habermehl, 1918, *Idiolispa*)
pygmaea (Habermehl, 1918, *Idiolispa*)
henrichi (Kiss, 1924, *Habrocryptus*)
Hidryta sordida ?*atlantica* Horstmann, 1990 tentative synonymy by Schwarz (2005)

##### Distribution

England, Scotland, Wales, Ireland

##### Notes

Added by Edgar (1971); overlooked by Fitton (1978).

#### Hoplocryptus

Thomson, 1873

#### Hoplocryptus bellosus

(Curtis, 1837)

Cryptus bellosus Curtis, 1837
signatorius (Fabricius, 1793, *Ichneumon*) preocc.
pulcher Thomson, 1873
fuscipes (Tschek, 1871, *Cryptus*)
thoracicus (Brischke, 1881, *Hygrocryptus*)
macrophyiae (Rudow, 1911, *Cryptus*)

##### Distribution

England, Ireland

#### Hoplocryptus bohemani

(Holmgren, 1856)

Cryptus bohemani Holmgren, 1856
rufoniger (Desvignes, 1856, *Cryptus*) synonymy by Horstmann (2000a)
mesoxanthus Thomson, 1873

#### Hoplocryptus confector

(Gravenhorst, 1829)

Cryptus confector Gravenhorst, 1829
albus (Taschenberg, 1865, *Cryptus*)
brachysoma (Taschenberg, 1865, *Cryptus*) synonymy by Schwarz (2005)
dubius (Taschenberg, 1865, *Cryptus*) synonymy by Schwarz and Shaw (1998)
elegans Thomson, 1873
thomsoni (Bridgman, 1881, *Cryptus*)
gladiator Kriechbaumer, 1899
caudatus Szépligeti, 1916
quadratus (Szépligeti, 1916, *Gambrus*) preocc.
gallicus (Habermehl, 1923, *Gambrus*)
enslini (Habermehl, 1923, *Spilocryptus*) preocc.
exannulatus Habermehl, 1926
hungaricus Habermehl, 1926 preocc.

##### Distribution

England

#### Hoplocryptus melanocephalus

(Gravenhorst, 1829)

Cryptus melanocephalus Gravenhorst, 1829

##### Distribution

England

##### Notes

added by Schwarz (2007)

#### Hoplocryptus murarius

(Börner, 1782)

Ichneumon murarius Börner, 1782
olitorius (Fabricius, 1793, *Ichneumon*) synonymy by Horstmann (2001a)
fugitivus (Gravenhorst, 1829, *Cryptus*) synonymy by Schwarz (2007)
gracilis (Gravenhorst, 1829, *Cryptus*)
binotatulus Thomson, 1873
pseudocryptus (Szépligeti, 1916, *Gambrus*)
tegularis (Szépligeti, 1916, *Gambrus*)
ignalinoensis Strand, 1918
ratzeburgi (Habermehl, 1919, *Spilocryptus*)
obscurata (Kiss, 1929, *Habrocryptus*) preocc.

##### Notes

*Ichneumon murarius* has priority over *fugitivus* (as used by Schwarz and Shaw 1998) or *olitorius*; Hoplocryptus binotatulus f. grandis Habermehl, 1926 was removed from synonymy and is a junior synonym of the extralimital *H. besseianus* (Seyrig, 1926) (Schwarz 2007).

#### Hoplocryptus quadriguttatus

(Gravenhorst, 1829)

Cryptus quadriguttatus Gravenhorst, 1829
cognatus (Fonscolombe, 1850, *Cryptus*) synonymy by Schwarz (2005)
mallorcanus Kriechbaumer, 1894 synonymy by Schwarz (2007)
enslini Habermehl, 1921 synonymy by Schwarz (2007)
hebraicator (Aubert, 1970, *Aritranis*) synonymy by Schwarz (2005)
tiloidalis (Kolarov & Beyarslan, 1994, *Agrothereutes*) synonymy by Schwarz (2005)

##### Distribution

England

#### Idiolispa

Förster, 1869

LIOCRYPTUS Thomson, 1873
PARACRYPTUS Szépligeti, 1916

##### Notes

species of *Idiolispa* deleted from the British and Irish list by Schwarz and Shaw (1998):

[*obfuscator* (Villers, 1789, *Ichneumon*)] Fitton (1978) listed *obfuscator* (Villers) as a doubtfully placed species of *Trychosis* but it was probably misidentified.

#### Idiolispa analis

(Gravenhorst, 1807)

Bassus analis Gravenhorst, 1807
cursor (Thunberg, 1824, *Ichneumon*) preocc.
elevata (Zetterstedt, 1838, *Cryptus*)
dubiosa (Kiss, 1924, *Spilocryptus*)

##### Distribution

England, Scotland, Wales, Ireland

#### Idiolispa hungarica

(Szépligeti, 1916)

Paracryptus hungaricus Szépligeti, 1916
Idiolispa hungarica ?*grossa* misident.; Schwarz and Shaw (1999)
meyeri (Habermehl, 1926, *Spilocryptus*)

##### Notes

Status as a British species (listed as *Agrothereutes grossus* (Grav.) by Fitton 1978) regarded as tentative by Schwarz and Shaw (1998).

#### Idiolispa subalpina

(Schmiedeknecht, 1904)

Spilocryptus subalpinus Schmiedeknecht, 1904
heydeni (Habermehl, 1919, *Spilocryptus*)

##### Distribution

Scotland

##### Notes

added by Schwarz and Shaw (1998)

#### Ischnus

Gravenhorst, 1829

HABROCRYPTUS Thomson, 1873

#### Ischnus agitator

(Olivier, 1792)

Ichneumon agitator Olivier, 1792
destructorius (Fabricius, 1793, *Ichneumon*) synonymy by Horstmann (2001a)
rubricator (Panzer, 1801, *Ichneumon*)
constrictor (Fabricius, 1804, *Cryptus*)
minutorius (Fabricius, 1804, *Cryptus*)
minor (Thunberg, 1824, *Ichneumon*)
pictor (Thunberg, 1824, *Ichneumon*)
dineurae (Rudow, 1882, *Cryptus*)
oriicus (De Stefani, 1886, *Cryptus*) synonymy by Horstmann (2000a)

#### Ischnus alternator

(Gravenhorst, 1829)

Cryptus alternator Gravenhorst, 1829
unicinctus (Gravenhorst, 1829, *Cryptus*)
striatellus (Zetterstedt, 1838, *Cryptus*)
annulipes (Taschenberg, 1865, *Cryptus*)
annulitarsis (Rudow, 1882, *Cryptus*)

##### Distribution

England, Scotland, Wales

#### Ischnus inquisitorius

(Müller, 1776)

Ichneumon inquisitorius Müller, 1776
migrator misident.
dictator (Geoffroy, 1785, *Ichneumon*)
porrectorius (Fabricius, 1787, *Ichneumon*)
leucostictos (Gmelin, 1790, *Ichneumon*)
sanguinolentus (Gmelin, 1790, *Ichneumon*)
assertorius (Fabricius, 1793, *Ichneumon*)
zonator (Fabricius, 1793, *Ichneumon*)
triplicatorius (Thunberg, 1824, *Ichneumon*)
brachyurus (Gravenhorst, 1829, *Cryptus*)
geminus (Gravenhorst, 1829, *Cryptus*) synonymy by Sawoniewicz (2003)
sannio (Gravenhorst, 1829, *Cryptus*)
sedulus (Gravenhorst, 1829, *Cryptus*) synonymy by Sawoniewicz (2003)
flavopictus (Rudow, 1883, *Cryptus*)
alpinus (Strobl, 1901, *Habrocryptus*)
obscuratus (Kiss, 1924, *Habrocryptus*)

##### Distribution

England

##### Notes

Horstmann (1968) misidentified Fabricius’s *migrator*, a mistake followed by Schwarz and Shaw (1998). Horstmann’s subsequent (Horstmann 2001a) lectotype designations resulted in *inquisitorius* becoming the valid name for *migrator* *sensu* Horstmann (1968) and Schwarz and Shaw (1998) and *migrator* being the senior synonym for *collaris*. Sawoniewicz (2003) correctly applied the names (Horstmann, pers. comm.).

#### Ischnus migrator

(Fabricius, 1775)

Ichneumon migrator Fabricius, 1775
collaris (Tschek, 1872, *Cryptus*) synonymy by Horstmann (2001a)
punctiger (Thomson, 1896, *Habrocryptus*) synonymy by Schwarz and Shaw (1998)
insulanus (Krieger, 1897, *Habrocryptus*) synonymy by Schwarz and Shaw (1998)
helveticator Aubert, 1968 synonymy by Schwarz (2005)

##### Distribution

England, Scotland

#### Listrognathus

Tschek, 1871

MESOSTENIDEA Viereck, 1914
MESOSTENUS misident.

#### Listrognathus firmator

(Fabricius, 1798)

Ichneumon firmator Fabricius, 1798
ligator (Gravenhorst, 1829, *Mesostenus*)
senilis (Rudow, 1882, *Cryptus*)
aculeatus (Rudow, 1883, *Cryptus*)

##### Distribution

England

##### Notes

The valid name according to Horstmann (2001a); listed as *ligator* by Schwarz and Shaw (1998). Distribution data from Horstmann (1990b).

#### Listrognathus mactator

(Thunberg, 1824)

Ichneumon mactator Thunberg, 1824
niveatus (Gravenhorst, 1829, *Mesostenus*)
pygostolus (Gravenhorst, 1829, *Mesostenus*)
tricolor Tschek, 1872
intermedius (Szépligeti, 1916, *Mesostenus*)

#### Listrognathus mengersseni

Schmiedeknecht, 1905

##### Distribution

England

##### Notes

added by Horstmann (1990b)

#### Listrognathus obnoxius

(Gravenhorst, 1829)

Mesostenus obnoxius Gravenhorst, 1829
zygaenarum (Ratzeburg, 1847, *Cryptus*) synonymy by Horstmann (1997)
subovalis (Thomson, 1873, *Mesostenus*)
robustus (Rudow, 1882, *Cryptus*)
subcircularis (Thomson, 1896, *Mesostenus*)

##### Distribution

England, Wales

#### Meringopus

Förster, 1869

GONIOCRYPTUS Thomson, 1873

#### Meringopus attentorius

(Panzer, 1804)

Ichneumon attentorius Panzer, 1804
confiscator (Fabricius, 1804, *Cryptus*) synonymy by Horstmann (2001a)
alboannulatus (Szépligeti, 1916, *Cryptus*)

#### Meringopus cyanator

(Gravenhorst, 1829)

Cryptus cyanator Gravenhorst, 1829
Meringopus cyanator ?*fuscescens* (Gmelin, 1790, *Ichneumon*)
Meringopus cyanator ?*roeselii* (Bechstein & Scharfenberg, 1805, *Ichneumon*)

#### Meringopus titillator

(Linnaeus, 1758)

Ichneumon titillator Linnaeus, 1758
recreator (Fabricius, 1804, *Cryptus*)
tornator (Panzer, 1804, *Ichneumon*)
pupurator (Thunberg, 1824, *Ichneumon*)
australis (Tschek, 1871, *Cryptus*) preocc.
latitarsis (Thomson, 1873, *Cryptus*)
pectinitarsis (Rudow, 1882, *Cryptus*)
titillatrix (Schulz, 1906, *Trychosis*)
meridionalis (Szépligeti, 1916, *Cryptus*)
orientalis (Szépligeti, 1916, *Cryptus*)
nigripes (Seyrig, 1927, *Cryptus*)

##### Distribution

England

#### Mesostenus

Gravenhorst, 1829

STENARAEUS Thomson, 1896

#### Mesostenus transfuga

Gravenhorst, 1829

gallarum (Rudow, 1881, *Hemiteles*)
gallarum (Rudow, 1882, *Hemiteles*) preocc.
ingenuus Tosquinet, 1896
niger Kiss, 1929 synonymy by Schwarz (2005)

##### Distribution

England

#### Nematopodius

Gravenhorst, 1829

PSEUDOPIMPLA Fahringer, 1935

#### Nematopodius debilis

(Ratzeburg, 1852)

Mesostenus debilis Ratzeburg, 1852
formosus misident.
tricolor (Haupt, 1954, *Mesostenus*) preocc.
homonymator (Aubert, 1959, *Mesostenus*)

##### Distribution

England

#### Picardiella

Lichtenstein, 1920

BORCIELLA Constantineanu, 1929
NIPPORICNUS Uchida, 1931
PARETHA Seyrig, 1952

#### Picardiella melanoleuca

(Gravenhorst, 1829)

Cryptus melanoleucus Gravenhorst, 1829
argiola (Rudow, 1882, *Mesostenus*)
peregrina (Schmiedeknecht, 1905, *Mesostenus*)
crenulata (Constantineanu, 1929, *Borciella*)
tarsoleuca (Kiss, 1929, *Habrocryptus*)

##### Distribution

England

##### Notes

BMNH, det. Broad, added here

#### Polytribax

Förster, 1869

EPIPHOBUS Förster, 1869
NELEOPHRON Förster, 1869
PLESIGNATHUS Förster, 1869

##### Notes

*Polytribax* is transferred here from the Hemigastrini, following the molecular phylogenetic results of Laurenne et al. (2006). Unlike hemigastrine genera (where known), *Polytribax* attack Lepidoptera pupae.

#### Polytribax arrogans

(Gravenhorst, 1829)

Cryptus arrogans Gravenhorst, 1829
sectator (Gravenhorst, 1829, *Phygadeuon*)
longipes (Hartig, 1838, *Cryptus*)
halensis (Taschenberg, 1865, *Phygadeuon*)
nigriventris (Habermehl, 1917, *Microcryptus*) preocc.
tricolor (Fahringer, 1935, *Plectocryptus*)

##### Distribution

England, Scotland, Wales, Ireland, Isle of Man

#### Polytribax perspicillator

(Gravenhorst, 1807)

Ichneumon perspicillator Gravenhorst, 1807
desertor (Gravenhorst, 1829, *Phygadeuon*)
obscuripes (Taschenberg, 1865, *Phygadeuon*)
errator (Marshall, 1868, *Phygadeuon*)
rufofemoratus (Strobl, 1901, *Plectocryptus*)
nigrifemur (Kiss, 1929, *Cryptus*)
mocsari (Györfi, 1944, *Megaplectes*)

##### Distribution

England, Scotland, Ireland

#### Polytribax picticornis

(Ruthe, 1859)

Cryptus picticornis Ruthe, 1859
gravenhorstii (Thomson, 1883, *Microcryptus*)

##### Distribution

England, Scotland, Wales

##### Notes

NMS, BMNH, added here

#### Polytribax rufipes

(Gravenhorst, 1829)

Cryptus rufipes Gravenhorst, 1829
curvus (Schrank, 1802, *Ichneumon*) preocc.
rufipes (Schrank, 1835, *Ichneumon*) preocc.
vexator (Pfankuch, 1921, *Microcryptus*)

##### Distribution

England, Ireland

#### Sphecophaga

Westwood, 1840

CHRYONOMON Desvignes, 1856
CACOTROPA Förster, 1869

#### Sphecophaga vesparum

(Curtis, 1828)

Anomalon vesparum Curtis, 1828
striata (Zetterstedt, 1838, *Bassus*)
vesparum (Ratzeburg, 1852, *Tryphon*) preocc.
sericea (Thomson, 1888, *Cacotropa*)
thuringiaca Schmiedeknecht, 1914

##### Distribution

England, Scotland, Ireland

#### Thrybius

Townes, 1965

#### Thrybius brevispina

(Thomson, 1896)

Hygrocryptus brevispina Thomson, 1896
puhlmanni (Ulbricht, 1909, *Hygrocryptus*)

##### Distribution

England

##### Notes

Incorrectly listed as a synonym of *praedator* in Yu and Horstmann (1997).

#### Thrybius praedator

(Rossi, 1792)

Ichneumon praedator Rossi, 1792
leucopygus (Gravenhorst, 1829, *Hoplismenus*)
praedator (Gravenhorst, 1829, *Cryptus*) preocc.
sanguinolentus (Gravenhorst, 1829, *Cryptus*)
elegans (Desvignes, 1856, *Cryptus*)
drewseni (Thomson, 1873, *Hygrocryptus*)
picticornis (Rudow, 1882, *Cryptus*) preocc.
praedatrix (Schulz, 1906, *Aritranis*)
puhlmanni (Ulbricht, 1909, *Hygrocryptus*)
continuus (Ulbricht, 1910, *Hygrocryptus*) unavailable
atrocoxatus (Ulbricht, 1916, *Hygrocryptus*) unavailable

##### Distribution

England, Wales

#### Trychosis

Förster, 1869

#### Trychosis ambigua

(Tschek, 1871)

Cryptus ambiguus Tschek, 1871
mesocastana (Tschek, 1871, *Cryptus*) synonymy by Horstmann (2005a)
molesta (Tschek, 1871, *Cryptus*)
annulicornis (Thomson, 1896, *Goniocryptus*)
trisculpta (Habermehl, 1929, *Goniocryptus*) synonymy by Schwarz (2005)

##### Distribution

England

##### Notes

BMNH, det. Schwarz, added here

#### Trychosis atripes

(Gravenhorst, 1829)

Cryptus atripes Gravenhorst, 1829
castaniventris (Tschek, 1871, *Cryptus*)
curvipes (Tschek, 1871, *Cryptus*)
jugorum (Strobl, 1901, *Idiolispa*)

##### Distribution

England

##### Notes

BMNH, det. Schwarz, added here

#### Trychosis ingrata

(Tschek, 1871)

Cryptus ingratus Tschek, 1871
macroura (Thomson, 1873, *Goniocryptus*)

##### Notes

added by Schwarz and Shaw (1998); tentative identification 

#### Trychosis insularis

Rossem, 1990

##### Distribution

England

##### Notes

added by Schwarz and Shaw (1998)

#### Trychosis legator

(Thunberg, 1824)

Ichneumon legator Thunberg, 1824
mesocastana misident.
titillator misident.
bicolor (Lucas, 1849, *Cryptus*)
abnormis (Tschek, 1871, *Cryptus*)
inimica (Tschek, 1871, *Cryptus*)
plebeja (Tschek, 1871, *Cryptus*)
rustica (Tschek, 1871, *Cryptus*)
simulator (Tschek, 1871, *Cryptus*)
clypearis (Thomson, 1873, *Goniocryptus*)
parvula (Kriechbaumer, 1894, *Goniocryptus*)
simulatrix Schulz, 1906
timenda Rossem, 1990

##### Distribution

England, Scotland, Wales, Ireland, Isle of Man

#### Trychosis neglecta

(Tschek, 1871)

Cryptus neglectus Tschek, 1871

##### Distribution

England

##### Notes

BMNH, det. Schwarz, added here

#### Trychosis picta

(Thomson, 1873)

Goniocryptus pictus Thomson, 1873

##### Distribution

England, Scotland

##### Notes

added by Schwarz and Shaw (1998) and removed from synonymy with *legator*

#### Trychosis tristator

(Tschek, 1871)

Cryptus tristator Tschek, 1871
glabricula (Thomson, 1873, *Goniocryptus*)
pleuralis (Thomson, 1896, *Goniocryptus*)
tristatrix Schulz, 1906

##### Distribution

England

##### Notes

added by Schwarz and Shaw (1998); UM

#### Xylophrurus

Förster, 1869

NYXEOPHILUS Förster, 1869
MACROCRYPTUS Thomson, 1873
NYXEOPHILUS Thomson, 1885

#### Xylophrurus lancifer

(Gravenhorst, 1829)

Echthrus lancifer Gravenhorst, 1829
dispar (Thunberg, 1824, *Ichneumon*)
Xylophrurus lancifer ?*nubeculatus* (Gravenhorst, 1829, *Echthrus*)
Xylophrurus lancifer ?*dentifer* (Thomson, 1896, *Caenocryptus*)
Xylophrurus lancifer ?*castaniventris* (Habermehl, 1909, *Kaltenbachia*)
Xylophrurus lancifer ?*rufescens* (Ozolz, 1942, *Kaltenbachia*)

##### Distribution

England, Scotland

##### Notes

added by Schwarz and Shaw (1998); it is unclear whether several names are synonyms of *lancifer* or *tumidus*.

#### Xylophrurus tumidus

(Desvignes, 1856)

Cryptus tumidus Desvignes, 1856
longiseta (Rudow, 1882, *Cryptus*)

##### Distribution

England

##### Notes

Separated from *lancifer* by Schwarz and Shaw (1998).

#### Stenarella

Szépligeti, 1916

##### Notes

Species of *Stenarella* excluded from the British and Irish list by Schwarz and Shaw (1998):

[*domator* (Poda, 1761, *Ichneumon*); syn. *gladiator* (Scopoli, 1763, *Ichneumon*)]

#### HEMIGASTRINI

Ashmead, 1900

APTESINI Smith & Shenefelt, 1955
ECHTHRINI Narayanan & Kundanlal, 1958

##### Notes

Following the molecular phylogenetic results of Laurenne et al. (2006), the genera *Echthrus* and *Polytribax* have been transferred to the Cryptini, together with *Helcostizus* from Phygadeuontini. ‘Hemigastrini’ is to be preferred over ‘Hemigasterini’ (Burks 2012). Distribution data from BMNH, NMS (mostly det. M. Schwarz and J. Sawoniewicz in the latter) and UM; Fitton (1976) also supplies some data on collection localities of type specimens.

#### Aconias

Cameron, 1904

#### Aconias tarsatus

(Bridgman, 1881)

Phygadeuon tarsatus Bridgman, 1881
pectoralis (Thomson, 1896, *Plectocryptus*)
lateannulatus (Strobl, 1901, *Chaeretymma*)
nigrofemoratus (Strobl, 1901, *Plectocryptus*)

##### Distribution

England, Scotland, Ireland

#### Aptesis

Förster, 1850

PEZOPORUS Förster, 1869
CLYPEODIODON Aubert, 1968

##### Notes

doubtfully placed species of *Aptesis*:

[*leucosticta* (Gravenhorst, 1829, *Cryptus*) nom. dub.]

#### Aptesis assimilis

(Gravenhorst, 1829)

Phygadeuon assimilis Gravenhorst, 1829
distans (Thomson, 1883, *Microcryptus*)

##### Distribution

Ireland, Isle of Man

#### Aptesis cretata

(Gravenhorst, 1829)

Phygadeuon cretatus Gravenhorst, 1829

##### Distribution

England

#### Aptesis femoralis

(Thomson, 1883)

Microcryptus femoralis Thomson, 1883
zonata (Kriechbaumer, 1893, *Microcryptus*)
alpina (Strobl, 1901, *Microcryptus*)

##### Distribution

England, Ireland

#### Aptesis flagitator

(Rossius, 1794)

Icheumon flagitator Rossius, 1794
pumilio (Gravenhorst, 1829, *Phygadeuon*)
tyranna (Gravenhorst, 1829, *Phygadeuon*) preocc.
hopei (Desvignes, 1856, *Cryptus*)
proximator (Costa, 1886, *Phygadeuon*)
tricolor (Kriechbaumer, 1894, *Microcryptus*)
hopei (Morley, 1907, *Acanthocryptus*) preocc.
feketei (Kiss, 1915, *Acanthocryptus*)
rufipes (Obrtel, 1953, *Acanthocryptus*)

##### Distribution

England, Ireland

#### Aptesis improba

(Gravenhorst, 1829)

Phygadeuon improbus Gravenhorst, 1829
exigua (Habermehl, 1909, *Microcryptus*)
bisignata (Habermehl, 1919, *Microcryptus*)

##### Distribution

Ireland

#### Aptesis jejunator

(Gravenhorst, 1807)

Ichneumon jejunator Gravenhorst, 1807
abdominator (Gravenhorst, 1829, *Phygadeuon*)
nematorum (Rudow, 1886, *Phygadeuon*)
genalis (Kriechbaumer, 1895, *Microcryptus*)
albilarva (Speiser, 1908, *Microcryptus*)
brumatae (Silvestri, 1941, *Microcryptus*)

##### Distribution

England, Ireland

#### Aptesis nigricollis

(Thomson, 1883)

Acanthocryptus nigricollis Thomson, 1883

##### Distribution

England, Ireland

##### Notes

Listed as a species of *Rhembobius* by Fitton (1978).

#### Aptesis nigritula

(Thomson, 1885)

Microcryptus nigritulus Thomson, 1885
nigripes (Strobl, 1901, *Stenocryptus*)

##### Distribution

England, Ireland

#### Aptesis nigrocincta

(Gravenhorst, 1815)

Ichneumon nigrocinctus Gravenhorst, 1815
Aptesis nigrocincta ?*bimaculata* (Christ, 1791, *Ichneumon*) preocc.
sudetica (Gravenhorst, 1815, *Ichneumon*)
duplicatoria (Thunberg, 1824, *Ichneumon, Ichneumon*)
flaveolata (Gravenhorst, 1829, *Phygadeuon*)
hostilis (Gravenhorst, 1829, *Cryptus*)
jucunda (Gravenhorst, 1829, *Phygadeuon*)
ephippia (Rudow, 1914, *Agrothereutes*)
fulvipes (Rudow, 1914, *Agrothereutes*)
haemorrhoidalis (Rudow, 1914, *Theroscopus*)
analis (Rudow, 1917, *Theroscopus*)
borealis Rudow, 1917
clythrae (Rudow, 1917, *Pezomachus*)
ephippia (Rudow, 1917, *Agrothereutes*) preocc.
fulvipes (Rudow, 1917, *Agrothereutes*) preocc.
haemorhoidalis (Rudow, 1917, *Theroscopus*) preocc.
nigrocincta (Rudow, 1917, *Pezomachus*) preocc.

##### Distribution

England, Scotland, Wales, Ireland

#### Aptesis orbitalis

(Thomson, 1883)

Microcryptus orbitalis Thomson, 1883

##### Notes

BMNH, added here; specimen labelled as from British Isles, Billups coll.

#### Aptesis scotica

(Marshall, 1868)

Phygadeuon scoticus Marshall, 1868

##### Distribution

Scotland

#### Aptesis terminata

(Gravenhorst, 1829)

Phygadeuon terminatus Gravenhorst, 1829
gilvipes (Gravenhorst, 1829, *Phygadeuon*)
ceilonota (Taschenberg, 1865, *Phygadeuon*)

##### Distribution

England

#### Colocnema

Förster, 1869

COELOCRYPTUS Thomson, 1873

#### Colocnema rufina

(Gravenhorst, 1829)

Phygadeuon rufinus Gravenhorst, 1829
romani (Pfankuch, 1914, *Plectocryptus*)

##### Distribution

England

#### Cratocryptus

Thomson, 1873

#### Cratocryptus furcator

(Gravenhorst, 1829)

Cryptus Gravenhorst, 1829

##### Distribution

Scotland

##### Notes

Material in UM, det. J.P. Brock, but see note under *subpetiolatus*.

#### Cratocryptus subpetiolatus

(Gravenhorst, 1829)

Cryptus subpetiolatus Gravenhorst, 1829

##### Distribution

England

##### Notes

Transferred from *Cubocephalus* by Sawoniewicz and Wanat (2003). This species seems to have been confused with *furcator* and it is not clear if *furcator* occurs in Britain or Ireland.

#### Cubocephalus

Ratzeburg, 1848

CHAERETYMMA Förster, 1869
ECPORTHETOR Förster, 1869
PAMMACHUS Förster, 1869
MICROCRYPTUS Thomson, 1873
STENOCRYPTUS Thomson, 1873
PLANOCRYPTUS Heinrich, 1949

#### Cubocephalus anatorius

(Gravenhorst, 1829)

Cryptus anatorius Gravenhorst, 1829
Cubocephalus anatorius ?*dumetorum* (Geoffroy, 1785, *Ichneumon*)
stomaticus (Gravenhorst, 1829, *Cryptus*) synonymy by Sawoniewicz (2003)
exareolatus (Habermehl, 1917, *Cratocryptus*)
albopictus (Kiss, 1924, *Plectocryptus*)

##### Distribution

England, Wales, Ireland

#### Cubocephalus associator

(Thunberg, 1824)

Ichneumon associator Thunberg, 1824
ruficoxis (Thomson, 1873, *Cratocryptus*)

##### Distribution

England

#### Cubocephalus brevicornis

(Taschenberg, 1865)

Phygadeuon brevicornis Taschenberg, 1865
oviventris misident.

##### Distribution

England, Ireland

#### Cubocephalus distinctor

(Thunberg, 1824)

Ichneumon distinctor Thunberg, 1824
fortipes misident.

##### Distribution

England, Scotland, Wales, Ireland

##### Notes

Sawoniewicz (2003) treats *fortipes* (Gravenhorst, 1829, *Cryptus*), with *canaliculatus* (Gravenhorst, 1829, *Ichneumon*) as a synonym, as distinct from *distinctor*. Despite Sawoniewicz’s quote of Morley (1907) for the concept of *fortipes*, all the British material in BMNH is referrable to *distinctor*, based on Sawoniewicz’s characters for separation of the species. NMS material has been identified by J. Sawoniewicz as *distinctor*.

#### Cubocephalus femoralis

(Thomson, 1873)

Cratocryptus femoralis Thomson, 1873
kriegeri (Habermehl, 1911, *Cratocryptus*)

#### Cubocephalus nigriventris

(Thomson, 1874)

Stenocryptus nigriventris Thomson, 1874

##### Distribution

England, Scotland, Wales, Ireland

#### Cubocephalus sperator

(Müller, 1776)

Ichneumon sperator Müller, 1776
bilineatus (Gravenhorst, 1829, *Cryptus*)
erythrinus (Gravenhorst, 1829, *Cryptus*) synonymy by Sawoniewicz (2003)
lacteator (Gravenhorst, 1829, *Cryptus*)
semiorbitalis (Gravenhorst, 1829, *Phygadeuon*)
cruentus (Kriechbaumer, 1891, *Microcryptus*)

##### Distribution

England, Scotland, Ireland

##### Notes

Listed as a species of *Pleolophus* in Yu and Horstmann (1997) but included in *Cubocephalus* by Sawoniewicz (2003).

#### Demopheles

Förster, 1869

#### Demopheles corruptor

(Taschenberg, 1865)

Phygadeuon corruptor Taschenberg, 1865

##### Distribution

England, Scotland, Ireland

##### Notes

*Phygadeuon caliginosus* Grav. is listed as a synonym of *D. corruptor* by Fitton (1978) but is now treated as a synonym of *Phygadeuon ovatus* Grav.

#### Giraudia

Förster, 1869

CALOCRYPTUS Thomson, 1873
PSEUDOCRYPTUS Kriechbaumer, 1893

#### Giraudia grisescens

(Gravenhorst, 1829)

Cryptus grisescens Gravenhorst, 1829
scansor (Thomson, 1890, *Plectocryptus*)
nigritarsis (Ulbricht, 1910, *Plectocryptus*) unavailable

##### Distribution

Ireland

#### Giraudia gyratoria

(Thunberg, 1824)

Ichneumon gyratorius Thunberg, 1824
congruens (Gravenhorst, 1829, *Cryptus*)
dimimilis (Kiss, 1924, *Megaplectes*)

#### Javra

Cameron, 1903

MONOCRYPTUS Hellén, 1957

#### Javra anomala

(Morley, 1908)

Diadegma anomala Morley, 1908
hedwigi (Habermehl, 1929, *Microcryptus*)

##### Distribution

England, Scotland, Wales

#### Javra opaca

(Thomson, 1873)

Cratocryptus opacus Thomson, 1873
gracilicornis (Kriechbaumer, 1891, *Microcryptus*)
jenneri (Heinrich, 1949, *Microcryptus*)

##### Distribution

Scotland

#### Javra tricincta

(Gravenhorst, 1829)

Cryptus tricinctus Gravenhorst, 1829
areolaris (Thomson, 1883, *Microcryptus*)
prominens (Schmiedeknecht, 1931, *Microcryptus*)

##### Distribution

England, Wales

#### Listrocryptus

Brauns, 1905

#### Listrocryptus spatulatus

Brauns, 1905

##### Distribution

England

##### Notes

NMS (det. Schwarz), BMNH, added here

#### Megaplectes

Förster, 1869

IOCRYPTUS Thomson, 1873
MEGALOPLECTES Schulz, 1906

#### Megaplectes monticola

(Gravenhorst, 1829)

Ichneumon monticola Gravenhorst, 1829
regius (Taschenberg, 1865, *Phygadeuon*)
andrei (Berthoumieu, 1897, *Eurylabus*)
lucens Torka, 1935

#### Oresbius

Marshall, 1867

OPIDNUS Förster, 1869

#### Oresbius arridens

(Gravenhorst, 1829)

Phygadeuon arridens Gravenhorst, 1829
niveatus (Desvignes, 1856, *Ichneumon*)
rhombifer (Kriechbaumer, 1893, *Microcryptus*)

##### Distribution

England, Ireland

#### Oresbius castaneus

Marshall, 1867

terrestris (Roman, 1909, *Microcryptus*)

##### Distribution

Scotland

#### Oresbius funereus

(Schmiedeknecht, 1905)

Microcryptus funereus Schmiedeknecht, 1905

#### Oresbius galactinus

(Gravenhorst, 1829)

Phygadeuon galactinus Gravenhorst, 1829
fulgens (Taschenberg, 1865, *Phygadeuon*)
nigricans (Pfankuch, 1923, *Microcryptus*) unavailable

##### Distribution

Ireland

#### Oresbius leucopsis

(Gravenhorst, 1829)

Cryptus leucopsis Gravenhorst, 1829
nycthemerus (Gravenhorst, 1829, *Phygadeuon*)
brumatae (Rudow, 1886, *Phygadeuon*)
nigriventris (Thomson, 1896, *Mesocryptus*)
victorovi Jonaitis, 1981

##### Distribution

England, Ireland

#### Oresbius nivalis

(Zetterstedt, 1838)

Cryptus nivalis Zetterstedt, 1838
opacus (Taschenberg, 1865, *Cryptus*) preocc.
borealis (Thomson, 1883, *Microcryptus*)
exannulatus (Roman, 1909, *Microcryptus*) preocc.

#### Oresbius subguttatus

(Gravenhorst, 1829)

Cryptus subguttatus Gravenhorst, 1829
contracta (Gravenhorst, 1829, *Cryptus*)
punctata (Ratzeburg, 1844, *Cryptus*)
abscissa (Ratzeburg, 1852, *Cryptus*)
incerta (Ratzeburg, 1852, *Cryptus*)
silesiacus (Habermehl, 1920, *Platylabus*) synonymy by Horstmann (2006d)
discedens (Habermehl, 1929, *Platylabus*) synonymy by Horstmann (2006d)

##### Distribution

England, Ireland

##### Notes

Listed as a species of *Aptesis* by Yu and Horstmann (1997).

#### Parmortha

Townes, 1962

#### Parmortha parvula

(Gravenhorst, 1829)

Cryptus parvulus Gravenhorst, 1829
erythropus (Gravenhorst, 1829, *Cryptus*)

##### Distribution

England, Scotland, Ireland

#### Parmortha pleuralis

(Thomson, 1873)

Cratocryptus pleuralis Thomson, 1873

##### Distribution

England, Scotland, Ireland, Isle of Man

#### Plectocryptus

Thomson, 1873

#### Plectocryptus albulatorius

(Gravenhorst, 1829)

Cryptus albulatorius Gravenhorst, 1829
hilarulus Schmiedeknecht, 1905

#### Plectocryptus digitatus

(Gmelin, 1790)

Ichneumon digitatus Gmelin, 1790
bivinctus (Gravenhorst, 1829, *Cryptus*)
poecilopus (Rudow, 1883, *Cryptus*)
niger (Kiss, 1926, *Habrocryptus*)

##### Distribution

England, Scotland

#### Plectocryptus effeminatus

(Gravenhorst, 1829)

Cryptus effeminatus Gravenhorst, 1829
flavopunctatus (Bridgman, 1889, *Phygadeuon*)
armatus (Kriechbaumer, 1893, *Microcryptus*)
clavatus (Kriechbaumer, 1893, *Microcryptus*)
sellatus Ulbricht, 1911 unavailable
lancifer (Roman, 1925, *Acanthocryptus*)

##### Distribution

England

#### Plectocryptus periculosus

(Schmiedeknecht, 1905)

Microcryptus periculosus Schmiedeknecht, 1905

##### Distribution

England

##### Notes

NMS, det. Schwarz, added here

#### Pleolophus

Townes, 1962

#### Pleolophus basizonus

(Gravenhorst, 1829)

Phygadeuon basizonus Gravenhorst, 1829
larvincola (Scharfenberg, 1805, *Ichneumon*) *nom. ob*.
varicolor (Gravenhorst, 1829, *Cryptus*)
pteronum (Hartig, 1838, *Phygadeuon*)
commutatus (Ratzeburg, 1848, *Phygadeuon*)
obscurus (Ulbricht, 1913, *Microcryptus*) unavailable
nigrinus (Fahringer, 1941, *Spilocryptus*)

##### Distribution

England, Scotland, Ireland

##### Notes

Sawoniewicz (2003) established that *basizonus* Grav. is a junior synonym of *larvincola* but under article 23.9 of the Code (ICZN 1999), *bazizonus* should be a protected name and *larvincola* a *nomen oblitum* (Horstmann 2006b).

#### Pleolophus brachypterus

(Gravenhorst, 1815)

Ichneumon brachypterus Gravenhorst, 1815
micropterus misident.
assimilis (Förster, 1850, *Aptesis*)
curtulus (Kriechbaumer, 1891, *Microcryptus*)
antennalis (Kiss, 1924, *Habrocryptus*)
hungaricus (Kis, 1924, *Hemichneumon*)

##### Distribution

England, Scotland, Wales, Ireland, Isle of Man

#### Pleolophus sericans

(Gravenhorst, 1829)

Phygadeuon sericans Gravenhorst, 1829
pictus (Gmelin, 1790, *Ichneumon*) preocc.
eximius (Habermehl, 1935, *Microcryptus*)

##### Distribution

England, Ireland

#### Pleolophus vestigialis

(Förster, 1850)

Aptesis vestigialis Förster, 1850
aphyopterus (Förster, 1850, *Aptesis*)
formosus (Förster, 1850, *Aptesis*)
unifasciatus (Schmiedeknecht, 1905, *Microcryptus*)
alpinus (Rudow, 1917, *Aptesis*)
triangularis (Kiss, 1924, *Microcryptus*)
angustipetiolatus (Ozols, 1934, *Microcryptus*)
piceus (Fahringer, 1935, *Stibeutes*)

#### Rhembobius

Förster, 1869

ULOTHYMUS Förster, 1869
ACANTHOCRYPTUS Thomson, 1873

##### Notes

Since Townes (1970), *Rhembobius* has been placed in the Phygadeuontini, subtribe Ethelurgina, along with other parasitoids of syrphid (Diptera) larvae, but Quicke et al. (2009) always found *Rhembobius* to group with the Hemigastrini in their molecular phylogenetic analyses. Distribution data from Horstmann (2000e), Schwarz and Shaw (2010), BMNH, plus additional reference.

#### Rhembobius bifrons

(Gmelin, 1790)

Ichneumon bifrons Gmelin, 1790
rufoniger (Bridgman, 1889, *Phygadeuon*)
minimus (Lange, 1911, *Microcryptus*)

##### Distribution

England, Scotland, Wales, Ireland

##### Notes

Listed as a species of *Aptesis* by Fitton (1978).

#### Rhembobius perscrutator

(Thunberg, 1824)

Ichneumon perscrutator Thunberg, 1824
basalis (Smith, 1874, *Cryptus*)

##### Distribution

England, Scotland, Wales, Ireland

##### Notes

some distribution data from Godfrey and Whitehead (2001)

#### Rhembobius quadrispinus

(Gravenhorst, 1829)

Phygadeuon quadrispinus Gravenhorst, 1829
ambiguus (Berthoumieu, 1914, *Platylabus*)
albicoxis (Kiss, 1915, *Ryssolabus*)
limnophilus (Smits van Burgst, 1920, *Acanthocryptus*) synonymy by Horstmann (2000e)
nigrobasicus (Kiss, 1924, *Acanthocryptus*)

##### Distribution

England, Scotland, Wales, Ireland, Isle of Man

#### Schenkia

Förster, 1869

ECPAGLUS Förster, 1869
SCHENCKIA Dalla Torre, 1901

#### Schenkia graminicola

(Gravenhorst, 1829)

Phygadeuon graminicola Gravenhorst, 1829
brevicornis (Gravenhorst, 1829, *Cryptus*)
humilis (Gravenhorst, 1829, *Cryptus*)
alta Jonaitis, 1981

##### Distribution

England, Scotland, Ireland

#### Schenkia labralis

(Gravenhorst, 1829)

Phygadeuon labralis Gravenhorst, 1829

##### Distribution

Ireland

#### Schenkia spinolae

(Gravenhorst, 1829)

Phygadeuon spinolae Gravenhorst, 1829

#### PHYGADEUONTINI

Förster, 1869

HEMITELINI Förster, 1869
GELINI Viereck, 1918

##### Notes

*Helcostizus* is transferred here to the Cryptini, following the molecular phylogenetic results of Laurenne et al. (2006), which also accords better with its morphology, very aberrant within the Phygadeuontini. See notes under ‘Cryptini’. Distribution data mainly taken from Schwarz and Shaw (1999), Schwarz and Shaw (2000), Schwarz and Shaw (2010), Schwarz and Shaw (2011), with some additional data from BMNH, in particular, also UM and some type localities from Fitton (1976); other sources are provided under the relevant genera/species.

#### Aclastus

Förster, 1869

DAETORA Förster, 1869
MICROPLEX Förster, 1869
OPISTHOSTENUS Förster, 1869
FETIALIS Rossem, 1990 synonymy by anon. (2001), Broad (2004)

#### Aclastus borealis

(Boheman, 1866)

Hemiteles borealis Boheman, 1866
septentrionalis (Holmgren, 1869, *Hemiteles*)

##### Distribution

Scotland

##### Notes

added by Horstmann (1980b)

#### Aclastus eugracilis

Horstmann, 1980

##### Distribution

England, Scotland, Wales

##### Notes

added by Schwarz and Shaw (2000)

#### Aclastus flavipes

Horstmann, 1980

##### Distribution

England

##### Notes

added by Horstmann (1980b)

#### Aclastus gracilis

(Thomson, 1884)

Hemiteles gracilis Thomson, 1884
furcifer Hellén, 1967

##### Distribution

England, Scotland, Wales, Ireland, Isle of Man

#### Aclastus micator

(Gravenhorst, 1807)

Ichneumon micator Gravenhorst, 1807
necator misident.
caudator Hellén, 1967

##### Distribution

England, Scotland, Wales

##### Notes

Listed, as *necator* (Fab.) (a name belonging to the Braconidae, Horstmann 2001a), as a doubtfully placed species of *Hemiteles* by Fitton (1978); Morley’s (Morley 1907) redescription of *necator* refers partly to *micator* (Schwarz and Shaw 2000).

#### Aclastus minutus

(Bridgman, 1886)

Hemiteles minutus Bridgman, 1886

##### Distribution

England, Scotland, Ireland

##### Notes

Irish occurrence from Anderson et al. (2006)

#### Aclastus pilosus

Horstmann, 1980

##### Distribution

England, Scotland, Wales, Ireland

##### Notes

added by Horstmann (1980b)

#### Aclastus solutus

(Thomson, 1884)

Hemiteles solutus Thomson, 1884

##### Distribution

England, Scotland, Wales, Ireland

#### Aclastus transversalis

Horstmann, 1980

##### Distribution

England, Wales

##### Notes

added by Horstmann (1980b)

#### Acrolyta

Förster, 1869

RHADINOCERA Förster, 1869

#### Acrolyta flagellator

Schwarz & Shaw, 2000

##### Distribution

Scotland

##### Notes

added by Schwarz and Shaw (2000)

#### Acrolyta marginata

(Bridgman, 1883)

Hemiteles marginatus Bridgman, 1883

##### Distribution

England, Scotland

#### Acrolyta nens

(Hartig, 1838)

Hemiteles nens Hartig, 1838
submarginata (Bridgman, 1883, *Hemiteles*)
rufizonata (Schmiedeknecht, 1905, *Hemiteles*)

##### Distribution

England, Scotland, Wales, Ireland

#### Acrolyta okadai

(Uchida, 1942)

Adiastola okadai Uchida, 1942

##### Notes

Added by Schwarz & Shaw (2000)Schwarz and Shaw (2000)and transferred from *Eudelus*.

#### Acrolyta pseudonens

Schwarz & Shaw, 2000

##### Distribution

England, Scotland

##### Notes

added by Schwarz and Shaw (2000)

#### Acrolyta rufocincta

(Gravenhorst, 1829)

Hemiteles rufocinctus Gravenhorst, 1829
distincta (Bridgman, 1883, *Hemiteles*)
capreolus (Thomson, 1884, *Hemiteles*)
quadrimaculata (Lange, 1911, *Hemiteles*)
obscurata (Kiss, 1924, *Hemiteles*)
unifasciata (Kiss, 1924, *Hemiteles*)

##### Distribution

England, Scotland, Wales

##### Notes

The name *rufocincta* was listed as a doubtfully placed species of *Hemiteles* by Fitton (1978) whilst the names *distincta* and *capreola* were listed as species of *Acrolyta* and *Eudelus*, respectively.

#### Acrolyta semistrigosa

(Schmiedeknecht, 1897)

Hemiteles semistrigosus Schmiedeknecht, 1897

##### Distribution

England

##### Notes

added by Schwarz and Shaw (2000)

#### Agasthenes

Förster, 1869

ASTHENOPTERA Förster, 1869

##### Notes

Some distribution data from Townes (1983).

#### Agasthenes subarcticus

(Jussila, 1965)

Hemiteles subarcticus Jussila, 1965

##### Distribution

Scotland, Ireland

##### Notes

added by Horstmann (1998a)

#### Agasthenes varitarsus

(Gravenhorst, 1829)

Hemiteles varitarsus Gravenhorst, 1829
stagnalis (Thomson, 1884, *Hemiteles*)

##### Distribution

England, Wales, Ireland

#### Amphibulus

Kriechbaumer, 1893

##### Notes

Some dstribution data from Sawoniewicz (2003).

#### Amphibulus gracilis

Kriechbaumer, 1893

bispinus (Thomson, 1894, *Cratocryptus*)
aertsi (Habermehl, 1926, *Stylocryptus*)

##### Distribution

England, Scotland, Ireland

#### Arotrephes

Townes, 1970

##### Notes

Some distribution data from Horstmann (1993c).

#### Arotrephes laeviscutum

Horstmann, 1993

##### Distribution

England

##### Notes

added by Horstmann (1993c)

#### Arotrephes parvipennis

(Thomson, 1884)

Phygadeuon parvipennis Thomson, 1884

##### Distribution

England, Scotland

##### Notes

added by Horstmann (1993c)

#### Arotrephes perfusor

(Gravenhorst, 1829)

Cryptus perfusor Gravenhorst, 1829
nitidus (Bridgman, 1889, *Hemiteles*)

##### Distribution

England, Scotland, Ireland

##### Notes

Listed by Fitton (1978) as *Charitopes nitidus* (Bridg.).

#### Arotrephes speculator

(Gravenhorst, 1829)

Phygadeuon speculator Gravenhorst, 1829

##### Distribution

England, Scotland, Ireland

#### Atractodes

Gravenhorst, 1829

##### Notes

Distribution data from Jussila (Jussila 1979, Jussila 1983, Jussila 2001), and the collections of NMS (det. Jussila), with additional references given.

doubtfully placed species of *Atractodes*: 

[*dionaeus* Haliday, 1837 nom. nud., from England, Ireland; Fitton (1976)]

[*piceicornis* Haliday, 1837 nom. nud., from Ireland; Fitton (1976)]

[*salius* Haliday, 1837 nom. nud., from Ireland; Fitton (1976)]

[*vestalis* Haliday, 1837 nom. nud.]

#### Asyncrita

Förster, 1876

#### Atractodes (Asyncrita) acuminator

Roman, 1909

##### Distribution

England

##### Notes

added byJussila (2001)

#### Atractodes (Asyncrita) albovinctus

Haliday, 1837

mediatus (Förster, 1876, *Asyncrita*)

##### Distribution

Ireland

##### Notes

Treated as a nomen nudum by Fitton (1976) but was established as a senior synonym of *mediatus* by Jussila (1994a).

#### Atractodes (Asyncrita) ambiguus

Ruthe, 1859

truncator Roman, 1909

##### Distribution

England

#### Atractodes (Asyncrita) angustipennis

Förster, 1876

adversarius Förster, 1876
affinis Förster, 1876
cryptonastes Förster, 1876
gracilentus Förster, 1876
invalidus Förster, 1876
subdentatus Förster, 1876
vilis Förster, 1876
flavicoxa Thomson, 1884
thomsonii Dalla Torre, 1902 preocc.

##### Distribution

Scotland, Ireland

#### Atractodes (Asyncrita) assimilis

Förster, 1876

minusculus Förster, 1876
sordidus Förster, 1876
sponsus Förster, 1876

##### Distribution

Scotland

##### Notes

added by Jussila (2001)

#### Atractodes (Asyncrita) croceicornis

Haliday, 1839

acceptus Förster, 1876
aemulator Förster, 1876
alticola Förster, 1876
atricornis Förster, 1876
contrarius Förster, 1876
designatus Förster, 1876
difficilis Förster, 1876
distinctus Förster, 1876
engadinus Förster, 1876
exosus Förster, 1876
expertus Förster, 1876
fatalis Förster, 1876
inclinans Förster, 1876
infestus Förster, 1876
intemperans Förster, 1876
laboriosus Förster, 1876
minax Förster, 1876
modestus Förster, 1876
nodifer Förster, 1876
obsoletus Förster, 1876
placidus Förster, 1876
praepotens Förster, 1876
progenitus Förster, 1876
quaerulosus Förster, 1876
rapinatorius Förster, 1876
reconditus Förster, 1876
ruficinctus Förster, 1876
singularis Förster, 1876
solivagus Förster, 1876
sollicitator Förster, 1876
sulcatulus Förster, 1876
ultorius Förster, 1876
vanus Förster, 1876
vorax Förster, 1876
ruficornis Brischke, 1880
compressus Thomson, 1884 preocc.

##### Distribution

England, Scotland, Ireland, Isle of Man

#### Atractodes (Asyncrita) cryptobius

Förster, 1876

eryptobius misspelling
carinatus Förster, 1876
conspicuus Förster, 1876
custoditor Förster, 1876
fulvicornis Förster, 1876
parallelus Thomson, 1884

##### Distribution

Ireland

##### Notes

added by Jussila (1979)

#### Atractodes (Asyncrita) cultellator

Haliday, 1839

##### Distribution

Ireland

#### Atractodes (Asyncrita) exilis

Haliday, 1839

alpicola (Förster, 1876, *Asyncrita*)
angustulus Förster, 1876
delicatulus Förster, 1876
dispar Förster, 1876
flavicoxis Förster, 1876
longiventris (Förster, 1876, *Asyncrita*)
suspicax Förster, 1876
xanthocarpus Förster, 1876
alpicola Strobl, 1901 preocc.

##### Distribution

England, Scotland, Ireland

#### Atractodes (Asyncrita) exitialis

Förster, 1876

breviusculus Förster, 1876
callidus Förster, 1876
debilis Förster, 1876
difformis Förster, 1876
infimus Förster, 1876
parilis Förster, 1876
particeps Förster, 1876
perpusillus Förster, 1876

##### Distribution

Scotland

##### Notes

added by Jussila (2001)

#### Atractodes (Asyncrita) foveolatus

Gravenhorst, 1829

canaliculatus (Hellén, 1944, *Asyncrita*)

##### Distribution

England

#### Atractodes (Asyncrita) picipes

Holmgren, 1860

nigripes Förster, 1876

#### Atractodes (Asyncrita) spiraculator

Roman, 1918

##### Distribution

Scotland

##### Notes

added by Jussila (2001)

#### Atractodes

Gravenhorst, 1829

ZETESIMA Förster, 1876

#### Atractodes (Atractodes) alpestris

Roman, 1918

##### Distribution

Ireland

##### Notes

added by Jussila (1979)

#### Atractodes (Atractodes) arator

Haliday, 1839

rufiventris Strobl, 1901 preocc.

##### Distribution

Scotland, Ireland

#### Atractodes (Atractodes) bicolor

Gravenhorst, 1829

pygmaeator (Zetterstedt, 1838, *Ichneumon*)
alpigradus Förster, 1876
analogus Förster, 1876
cultrarius Förster, 1876 preocc.
destructor Förster, 1876
incommodus Förster, 1876
indigena Förster, 1876
lepidus Förster, 1876
mesoxanthus Förster, 1876
montivagus Förster, 1876
tenax Förster, 1876

##### Distribution

Scotland

#### Atractodes (Atractodes) citator

Haliday, 1839

##### Distribution

Ireland

#### Atractodes (Atractodes) fumatus

Haliday, 1839

abnormis Förster, 1876
ambifarius Förster, 1876
avidus Förster, 1876
castus Förster, 1876
discolor Förster, 1876
dissidens Förster, 1876
ecarinatus Förster, 1876
homologus Förster, 1876
incongruens Förster, 1876
isomorphus Förster, 1876
melanocerus Förster, 1876
melanostomus Förster, 1876
nigrocoxis Förster, 1876
proprius Förster, 1876
separatus Förster, 1876
subdolus Förster, 1876
tenuicinctus Förster, 1876
tenuis Förster, 1876
unicinctus Förster, 1876

##### Distribution

England, Scotland

#### Atractodes (Atractodes) gilvipes

Holmgren, 1860

#### Atractodes (Atractodes) gravidus

Gravenhorst, 1829

fraternus Förster, 1876
areolaris (Habermehl, 1909, *Exolytus*)
archangelicae Roman, 1913
brevicornis Bauer, 1958

##### Distribution

England

#### Atractodes (Atractodes) magnus

Jussila, 2001

##### Distribution

England

##### Notes

Ely coll., det Jussila, added here

#### Atractodes (Atractodes) obsoletor

(Zetterstedt, 1838)

Porizon obsoletor Zetterstedt, 1838
agilis Förster, 1876
declinis Förster, 1876
neophytus Förster, 1876
niger Förster, 1876 preocc.
foersteri Dalla Torre, 1901

##### Distribution

England, Wales

##### Notes

added by Jussila (1979)

#### Atractodes (Atractodes) pauxillus

Förster, 1876

montanus Förster, 1876
breviscapus Thomson, 1884

##### Distribution

England, Scotland, Ireland

#### Atractodes (Atractodes) pusillus

Förster, 1876

calceatus Förster, 1876
linearis Förster, 1876
tenellus Förster, 1876
liogaster Förster, 1876
pernitens Kokujev, 1909

##### Distribution

England, Scotland, Ireland

#### Atractodes (Atractodes) tenuipes

Thomson, 1884

##### Distribution

England, Scotland

##### Notes

added by Bass and Cooling (1983)

#### Atractodes (Atractodes) townesi

Jussila, 1983

thomsoni Jussila, 1979

##### Distribution

Scotland

##### Notes

added by Jussila (2001)

#### Cyclaulatractodes

Jussila, 1979

#### Atractodes (Cyclaulatractodes) helveticus

Förster, 1876

aequilongus Förster, 1876
oreophilus Förster, 1876 synonymy by Jussila (2001)

##### Distribution

Ireland

#### Atractodes (Cyclaulatractodes) punctator

Roman, 1909

##### Distribution

England, Scotland, Wales

##### Notes

added by Jussila (2001)

#### Hadratractodes

Jussila, 1979

#### Atractodes (Hadratractodes) vicinus

Förster, 1876

absconditus Förster, 1876
cautior Förster, 1876
inquilinus Förster, 1876
intersectus Förster, 1876
lentus Förster, 1876
rufipes Förster, 1876 preocc.
sectator Förster, 1876
venustulus Förster, 1876
crassicornis Förster, 1876
sarntheinii Dalla Torre, 1901

##### Distribution

Scotland

##### Notes

NMS, det. Jussila, added here

#### Rugratractodes

Jussila, 1979

#### Atractodes (Rugratractodes) alpinus

Förster, 1876

inimicus Förster, 1876

##### Distribution

Scotland

##### Notes

added by Jussila (2001)

#### Atractodes (Rugratractodes) incrassator

Roman, 1926

##### Distribution

Scotland

##### Notes

added by Jussila (2001)

#### Bathythrix

Förster, 1869

GAUSOCENTRUS Förster, 1869
ISCHNURGOPS Förster, 1869
PANARGYROPS Förster, 1869
STEGANOPS Förster, 1869
LEPTOCRYPTUS Thomson, 1873

##### Notes

Some distribution data from Sawoniewicz (1980).

#### Bathythrix aerea

(Gravenhorst, 1829)

Cryptus aereus Gravenhorst, 1829
brevis (Thomson, 1884, *Leptocryptus*)

##### Distribution

England, Scotland, Wales, Ireland

#### Bathythrix alter

(Kerrich, 1942)

Panargyrops alter Kerrich, 1942

##### Distribution

England, Wales

#### Bathythrix argentata

(Gravenhorst, 1829)

Hemiteles argentatus Gravenhorst, 1829
lacustris (Schmiedeknecht, 1905, *Leptocryptus*)

##### Distribution

England

#### Bathythrix claviger

(Taschenberg, 1865)

Cryptus claviger Taschenberg, 1865
atra (Brischke, 1881, *Cryptus*)

##### Distribution

England, Scotland, Ireland

#### Bathythrix collaris

(Thomson, 1896)

Leptocryptus collaris Thomson, 1896

##### Distribution

England, Scotland

#### Bathythrix decipiens

(Gravenhorst, 1829)

Hemiteles decipiens Gravenhorst, 1829
gyrini (Parfitt, 1881, *Hemiteles*)
signata (Habermehl, 1919, *Leptocryptus*)
meridionator (Aubert, 1960, *Panargyrops*)

##### Distribution

England, Ireland, Isle of Man

##### Notes

Listed (twice, as *decipiens* and *gyrini*) as doubtfully placed species of *Hemiteles* in Fitton (1978).

#### Bathythrix formosa

(Desvignes, 1860)

Hemiteles formosus Desvignes, 1860
albomarginata (Kriechbaumer, 1892, *Leptocryptus*)
Bathythrix formosa ?*grandimacula* (Kriechbaumer, 1892, *Leptocryptus*) unavailable
geniculosa (Thomson, 1884, *Leptocryptus*)

##### Distribution

England

##### Notes

*Bathythrix formosa* was separated from *fragilis* by Horstmann (1998a) (although the name *grandimacula* was not mentioned).

#### Bathythrix fragilis

(Gravenhorst, 1829)

Hemiteles fragilis Gravenhorst, 1829
bellula (Kriechbaumer, 1892, *Leptocryptus*)
urticarum (Habermehl, 1920, *Leptocryptus*)

##### Distribution

England, Scotland, Wales

#### Bathythrix lamina

(Thomson, 1884)

Leptocryptus lamina Thomson, 1884

##### Distribution

England, Scotland, Wales, Ireland, Isle of Man

##### Notes

some distribution data from Kerrich (1942)

#### Bathythrix linearis

(Gravenhorst, 1829)

Nematopodius linearis Gravenhorst, 1829
heteropus (Thomson, 1886, *Leptocryptus*)

##### Distribution

England

#### Bathythrix margaretae

Sawoniewicz, 1980

##### Distribution

England

##### Notes

added by Schwarz and Shaw (2010)

#### Bathythrix pellucidator

(Gravenhorst, 1829)

Cryptus pellucidator Gravenhorst, 1829
ruficaudata (Bridgman, 1883, *Hemiteles*)

##### Distribution

England, Scotland, Wales, Ireland, Isle of Man

#### Bathythrix prominens

(Strobl, 1901)

Leptocryptus prominens Strobl, 1901

##### Distribution

England, Scotland, Wales, Ireland, Isle of Man

##### Notes

added by Sawoniewicz (1980)

#### Bathythrix rugulosa

(Thomson, 1884)

Leptocryptus rugulosus Thomson, 1884

##### Distribution

England, Scotland, Ireland

##### Notes

added by Sawoniewicz (1980)

#### Bathythrix spheginus

(Gravenhorst, 1829)

Mesoleptus spheginus Gravenhorst, 1829
sphecinus (Schulz, 1906, *Mesoleptus*)

##### Distribution

England

##### Notes

added by Sawoniewicz (1980)

#### Bathythrix strigosa

(Thomson, 1884)

Leptocryptus strigosus Thomson, 1884
ruficollis (Habermehl, 1919, *Leptocryptus*)

##### Distribution

England, Scotland

##### Notes

added by Schwarz and Shaw (2010)

#### Bathythrix tenuis

(Gravenhorst, 1829)

Cryptus tenuis Gravenhorst, 1829
rubens (Kriechbaumer, 1892, *Leptocryptus*)

#### Bathythrix thomsoni

(Kerrich, 1942)

Thysiotorus thomsoni Kerrich, 1942
corsicator (Aubert, 1961, *Panargyrops*)

##### Distribution

England, Scotland, Wales, Ireland, Isle of Man

#### Blapsidotes

Förster, 1869

#### Blapsidotes vicinus

(Gravenhorst, 1829)

Hemiteles vicinus Gravenhorst, 1829
melanarius (Gravenhorst, 1829, *Hemiteles*)
pimplarius (Berthoumieu, 1904, *Platylabus*)

##### Distribution

England, Scotland, Ireland

##### Notes

Listed (as *melanarius*) as a doubtfully placed species of *Hemiteles* by Fitton (1978).

#### Cephalobaris

Kryger, 1915

#### Cephalobaris eskelundi

Kryger, 1915

##### Distribution

England

##### Notes

added by Schwarz and Shaw (2011)

#### Ceratophygadeuon

Viereck, 1924

EUROMONZIA Aubert, 1965

#### Ceratophygadeuon bellus

(Gravenhorst, 1829)

Ichneumon bellus Gravenhorst, 1829
longiceps (Thomson, 1884, *Phygadeuon*)

##### Notes

Not included in Fitton (1978) but described from a British specimen.

#### Ceratophygadeuon gracilicornis

Horstmann, 1979

##### Distribution

England

##### Notes

added by Schwarz and Shaw (2011)

#### Ceratophygadeuon parvicaudator

(Aubert, 1965)

Remonzia ?parvicaudator Aubert, 1965

##### Distribution

England

##### Notes

added by Schwarz and Shaw (2011); tentative identification

#### Ceratophygadeuon varicornis

(Thomson, 1885)

Phygadeuon varicornis Thomson, 1885
maritimus Horstmann, 1979 synonymy by Horstmann (2001b)

##### Distribution

England

##### Notes

added by Horstmann (1993a)

#### Charitopes

Förster, 1869

ADIASTOLA Förster, 1869

##### Notes

Some distribution data from Townes (1983) and Horstmann (1998a).

#### Charitopes areolaris

(Thomson, 1884)

Hemiteles areolaris Thomson, 1884
brunneus (Morley, 1907, *Hemiteles*)

##### Distribution

England, Scotland

##### Notes

Koponen et al. (1999) treat *brunneus* as a species distinct from *areolaris* but Horstmann (1998a) and Townes (1983) retain it as a synonym.

#### Charitopes carri

(Roman, 1923)

Cecidonomus carri Roman, 1923
londinensis (Morley, 1947, *Phygadeuon*)
hemerobii (Pfankuch, 1914, *Hemiteles*)
pusillus (Habermehl, 1920, *Hemiteles*) preocc.

##### Distribution

England, Scotland, Isle of Man

##### Notes

Horstmann (1998a) removed *carri* from synonymy with *areolaris*.

#### Charitopes clausus

(Thomson, 1888)

Hemiteles clausus Thomson, 1888

##### Distribution

England, Scotland, Ireland, Isle of Man

##### Notes

added by Townes (1983)

#### Charitopes gastricus

(Holmgren, 1868)

Hemiteles gastricus Holmgren, 1868
chrysopae (Brischke, 1890, *Hemiteles*)
flavigaster (Schmiedekecht, 1897, *Hemiteles*)
flavocinctus (Strobl, 1901, *Hemiteles*)
brunnescens (Schmiedekecht, 1905, *Hemiteles*)
sylvicola (Habermehl, 1920, *Hemiteles*)

##### Distribution

England, Scotland, Wales, Ireland

#### Charitopes wesmaeliicida

(Roman, 1934)

Hemiteles wesmaeliicida Roman, 1934

##### Distribution

England, Scotland

#### Chirotica

Förster, 1869

ALLOCOTA Förster, 1869 preocc.
DIAGLYPTA Förster, 1869
SPINOLIA Förster, 1869 preocc.
SYNECHES Förster, 1869 preocc.
DEUTEROSPINOLIA Dalla Torre, 1902

#### Chirotica maculipennis

(Gravenhorst, 1829)

Hemiteles maculipennis Gravenhorst, 1829
excellens (Imhoff, 1850, *Hemiteles*)
mulsantii (Fonscolombe, 1852, *Hemiteles*)
glyptonota (Thomson, 1885, *Hemiteles*)
schiefereri (Strobl, 1904, *Hemiteles*)

##### Distribution

England

##### Notes

Listed as a doubtfully placed species of *Hemiteles* by Fitton (1978).

#### Clypeoteles

Horstmann, 1974

#### Clypeoteles distans

(Thomson, 1884)

Hemiteles distans Thomson, 1884
rugifrons (Thomson, 1884, *Hemiteles*)
pseudorubiginosus (Strobl, 1901, *Hemiteles*)
xylonomoides (Morley, 1907, *Cecidonomus*)
fennicus (Hellén, 1967, *Catalytus*)

##### Distribution

England, Scotland

##### Notes

Listed as *Acrolyta xylonomoides* by Fitton (1978).

#### Cremnodes

Förster, 1850

CAENOMERIS Förster, 1869
STYGERA Förster, 1869
CREMNIAS Roman, 1939

#### Cremnodes atricapillus

(Gravenhorst, 1815)

Icheumon atricapillus Gravenhorst, 1815
combustus Förster, 1850
nanodes Förster, 1850

##### Distribution

England, Scotland, Wales, Ireland

##### Notes

some distribution data from Horstmann (1993c)

#### Cremnodes costalis

Horstmann, 1992

##### Distribution

England, Scotland

##### Notes

added by Horstmann (1992b)

#### Cremnodes rufipes

(Perkins, 1962)

Stygera rufipes Perkins, 1962

##### Distribution

England, Wales

##### Notes

some distribution data from Perkins (1962)

#### Diaglyptidea

Viereck, 1913

#### Diaglyptidea conformis

(Gmelin, 1790)

Ichneumon conformis Gmelin, 1790
secernenda (Schmiedeknecht, 1897, *Hemiteles*)

##### Distribution

England, Scotland, Ireland, Isle of Man

#### Dichrogaster

Doumerc, 1855

BRACHYCEPHALUS Förster, 1869
MICROTORUS Förster, 1869
OTACUSTES Förster, 1869
XENOBRACHYS Förster, 1869

##### Notes

Some distribution data from Townes (1983) and Horstmann (1992b).

#### Dichrogaster aestivalis

(Gravenhorst, 1829)

Hemiteles aestivalis Gravenhorst, 1829
ruficollis (Gravenhorst, 1829, *Hemiteles*)
geniculata (Thomson, 1884, *Hemiteles*)

##### Distribution

England, Scotland, Wales, Ireland

#### Dichrogaster bischoffi

(Schmiedeknecht, 1905)

Phygadeuon bischoffi Schmiedeknecht, 1905
rufovaria (Schmiedeknecht, 1905, *Phygadeuon*)

##### Distribution

England

##### Notes

added by Schwarz and Shaw (2000)

#### Dichrogaster genalis

(Habermehl, 1925)

Phygadeuon genalis Habermehl, 1925
varsoviensis (Sawoniewicz, 1978, *Ethelurgus*)

##### Distribution

England, Scotland, Ireland

##### Notes

added by Townes (1983)

#### Dichrogaster heteropus

(Thomson, 1896)

Phygadeuon heteropus Thomson, 1896
rufithorax (Schmiedeknecht, 1932, *Phygadeuon*)

##### Distribution

England, Scotland

##### Notes

added by Horstmann (1992b)

#### Dichrogaster liostylus

(Thomson, 1885)

Hemiteles liostylus Thomson, 1885
schaffneri (Schmiedeknecht, 1897, *Hemiteles*)

##### Distribution

England, Scotland, Ireland

#### Dichrogaster longicaudata

(Thomson, 1884)

Hemiteles longicaudatus Thomson, 1884
diatropus Townes, 1983

##### Distribution

England, Scotland

##### Notes

NMS, det. Schwarz, added here

#### Dichrogaster mandibularis

Horstmann, 1973

##### Distribution

England

##### Notes

added by Horstmann (1992b)

#### Dichrogaster modesta

(Gravenhorst, 1829)

Hemiteles modestus Gravenhorst, 1829
brunnea (Kiss, 1924, *Herpestomus*)

##### Distribution

England, Scotland, Wales

##### Notes

added by Townes (1983)

#### Dichrogaster perlae

(Doumerc, 1855)

Microgaster perlae Doumerc, 1855

##### Distribution

England

##### Notes

added by Townes (1983)

#### Dichrogaster schimitscheki

(Fahringer, 1935)

Phygadeuon schimitscheki Fahringer, 1935
nigrithorax Horstmann, 1976

##### Distribution

England

##### Notes

added by Townes (1983)

#### Encrateola

Strand, 1917

ENCRATES Förster, 1869

#### Encrateola glabra

Horstmann, 1998

##### Distribution

England

##### Notes

added by Schwarz and Shaw (2000)

#### Encrateola laevigata

(Ratzeburg, 1848)

Hemiteles laevigatus Ratzeburg, 1848
furcata (Taschenberg, 1865, *Hemiteles*)
subimpressa (Brischke, 1892, *Hemiteles*)

##### Distribution

England, Scotland, Wales, Ireland, Isle of Man

##### Notes

Listed as a doubtfully placed species of *Hemiteles* by Fitton (1978).

#### Endasys

Förster, 1869

BACHIA Förster, 1869 preocc.
SCINACOPUS Förster, 1869
STYLOCRYPTUS Thomson, 1873
BACHIANA Strand, 1929

##### Notes

Distribution data from Sawoniewicz and Luhman (1992), supplemented by material in BMNH and NMS. Several species were listed under *Glyphicnemis* by Fitton (1978).

#### Endasys alutaceus

(Habermehl, 1912)

Stylocryptus alutaceus Habermehl, 1912
nigriventris (Aerts, 1953, *Stylocryptus*)

##### Distribution

England

##### Notes

BMNH, det. Sawoniewicz & Luhman, added here

#### Endasys analis

(Thomson, 1883)

Stylocryptus analis Thomson, 1883

##### Distribution

England

##### Notes

BMNH, det. Sawoniewicz & Luhman, added here

#### Endasys anglianus

Sawoniewicz & Luhman, 1992

##### Distribution

England, Scotland, Wales

##### Notes

added by Sawoniewicz and Luhman (1992)

#### Endasys brevis

(Gravenhorst, 1829)

Phygadeuon brevis Gravenhorst, 1829

##### Distribution

England, Wales

##### Notes

added by Sawoniewicz and Luhman (1992)

#### Endasys brunnulus

Sawoniewicz & Luhman, 1992

##### Distribution

England

##### Notes

added by Sawoniewicz and Luhman (1992)

#### Endasys erythrogaster

(Gravenhorst, 1829)

Phygadeuon erythrogaster Gravenhorst, 1829
nigricoxis (Habermehl, 1912, *Stylocryptus*)

#### Endasys minutulus

(Thomson, 1883)

Stylocryptus minutulus Thomson, 1883
nigripes (Strobl, 1904, *Stylocryptus*)
fusciventris (Habermehl, 1916, *Stylocryptus*)

##### Distribution

England, Wales, Ireland

##### Notes

added by Sawoniewicz and Luhman (1992)

#### Endasys parviventris

(Gravenhorst, 1829)

Phygadeuon parviventris Gravenhorst, 1829
pictipes (Rudow, 1886, *Phygadeuon*)
tyrolensis (Schmiedeknecht, 1905, *Stylocryptus*)

##### Distribution

England

#### Endasys petiolus

Sawoniewicz & Luhman, 1992

##### Distribution

England

##### Notes

added by Sawoniewicz and Luhman (1992)

#### Endasys plagiator

(Gravenhorst, 1829)

Phygadeuon plagiator Gravenhorst, 1829
braunsi (Lange, 1911, *Acanthocryptus*)
laetus (Habermehl, 1929, *Stylocryptus*)

##### Distribution

England, Scotland, Isle of Man

##### Notes

added by Sawoniewicz and Luhman (1992)

#### Endasys proteuryopsis

Sawoniewicz & Luhman, 1992

##### Distribution

England

##### Notes

added by Sawoniewicz and Luhman (1992)

#### Endasys rusticus

(Habermehl, 1912)

Stylocryptus rusticus Habermehl, 1912

##### Distribution

England

#### Endasys senilis

(Gmelin, 1790)

Ichneumon senilis Gmelin, 1790

##### Distribution

England

#### Endasys striatus

(Kiss, 1924)

Acanthocryptus striatus Kiss, 1924

##### Distribution

England

##### Notes

added bySawoniewicz and Luhman (1992)

#### Endasys talitzkii

(Telenga, 1961)

Phygadaeuon talitzkii Telenga, 1961

##### Distribution

England

##### Notes

BMNH, det. Sawoniewicz & Luhman, added here

#### Endasys testaceipes

(Brischke, 1881)

Phygadeuon testaceipes Brischke, 1881
coxalis (Schmiedeknecht, 1905, *Stylocryptus*)

##### Distribution

England

##### Notes

added by Sawoniewicz and Luhman (1992)

#### Endasys thunbergi

Sawoniewicz & Luhman, 1992

rubricator (Thunberg, 1824, *Ichneumon*)

##### Distribution

England

#### Endasys transverseareolatus

(Strobl, 1901)

Stylocryptus transverseareolatus Strobl, 1901

#### Endasys triannulatus

Sawoniewicz & Luhman, 1992

##### Distribution

England

##### Notes

added by Sawoniewicz and Luhman (1992)

#### Endasys varipes

(Gravenhorst, 1829)

Phygadeuon varipes Gravenhorst, 1829

##### Distribution

England, Isle of Man

#### Ethelurgus

Förster, 1869

NUNECHES Förster, 1869
TOLMERUS Förster, 1869
PLATYCRYPTUS Kriechbaumer, 1893

##### Notes

Some distribution and synonymic data from Horstmann (2000e).

#### Ethelurgus sodalis

(Taschenberg, 1865)

Phygadeuon sodalis Taschenberg, 1865
pseudovulnerator (Strobl, 1901, *Phygadeuon*)
pici (Berthoumieu, 1908, *Platylabus*)
flavocinctus (Habermehl, 1909, *Phygadeuon*)
inermis (Habermehl, 1919, *Phygadeuon*)
niger (Pfankuch, 1824, *Phygadeuon*) preocc., unavailable

##### Distribution

England, Scotland, Wales, Ireland, Isle of Man

#### Ethelurgus vulnerator

(Gravenhorst, 1829)

Phygadeuon vulnerator Gravenhorst, 1829

##### Distribution

England, Scotland

##### Notes

Townes (1983) recorded *vulnerator* from Ireland but his interpretation of the species also included *sodalis* (Horstmann 2000e); I have seen an Irish specimen of *sodalis* coll. A. Anderson.

#### Eudelus

Förster, 1869

CALLIPHRURUS Förster, 1869
IDEMUM Förster, 1869

#### Eudelus pallicarpus

(Thomson, 1884)

Hemiteles pallicarpus Thomson, 1884
pallidicarpus (Dalla Torre, 1902, *Hemiteles*)
crassiformis (Viereck, 1917, *Hemiteles*) synonymy by Schwarz and Shaw (2000)

##### Distribution

England

##### Notes

Raised from synonymy with *simillimus* by Schwarz and Shaw (2000).

#### Eudelus scabriculus

(Thomson, 1884)

Hemiteles scabriculus Thomson, 1884

##### Distribution

England

##### Notes

Raised from synonymy with *simillimus* by Schwarz and Shaw (2000).

#### Eudelus simillimus

(Taschenberg, 1865)

Hemiteles simillimus Taschenberg, 1865
Eudelus simillimus ?*sericeus* (Rudow, 1886, *Hemiteles*)
Eudelus simillimus ?*albidus* (Pfankuch, 1925, *Hemiteles*)
Eudelus simillimus ?*meridionator* (Aubert, 1960, *Astomaspis*)

##### Distribution

England, Scotland, Ireland

##### Notes

Listed as a doubtfully placed species of *Hemiteles* by Fitton (1978). *Eudelus nigricoxis* (Kiss, 1924, *Hemiteles*) was treated as a valid species by Horstmann (2009c).

#### Eudelus mediovittatus

(Schmiedeknecht, 1897)

Hemiteles mediovittatus Schmiedeknecht, 1897

##### Distribution

England

##### Notes

Listed as *Encrateola mediovittatus* by Fitton (1978), it was transferred to *Acrolyta* by Horstmann (1983) but left as possibly belonging to *Eudelus* but probably to a new genus by Schwarz and Shaw (2000); although its generic position is in doubt its status as a British species is not. Recent specimens from Worcestershire (coll. J. Rush, specimens in BMNH and Schwarz coll.) have been identified by M. Schwarz.

#### Fianoniella

Horstmann, 1992

#### Fianoniella punctiscutum

(Horstmann, 1990)

Odontoneura punctiscutum Horstmann, 1990

##### Distribution

England

##### Notes

added by Horstmann (1990a)

#### Gelis

Thunberg, 1827

PEZOMACHUS Gravenhorst, 1829
PEZOLOCHUS Förster, 1850
CATALYTUS Förster, 1851
HEMIMACHUS Ratzeburg, 1852
ALEGINA Förster, 1869
ASCHISTUS Förster, 1869
BARYDOTIRA Förster, 1869
ILAPINASTES Förster, 1869
PHILONYGMUS Förster, 1869
PLESIOMMA Förster, 1869 preocc.
RHADIURGUS Förster, 1869 preocc.
TERPIPHORA Förster, 1869
URITHEPTUS Förster, 1869
LEPTOGELIS Ceballos, 1925
FIANONIA Seyrig, 1952
HOLCOGELIS Aubert, 1957
ARCTODEUON Hellén, 1967
RHADIURGINUS Hellén, 1967

##### Notes

species of *Gelis* excluded from the British list by Schwarz and Shaw (1999)

[*alpivagus* (Strobl, 1901, *Hemiteles*) misident.]

[*stevenii* (Gravenhorst, 1829, *Pezomachus*) misident.]

[*taschenbergii* (Schmiedeknecht, 1897, *Hemiteles*) misident.] Listed as a doubtfully placed species of *Hemiteles* by Fitton (1978).

#### Gelis acarorum

(Linnaeus, 1758)

Ichneumon acarorum Linnaeus, 1758
nigricornis (Retzius, 1783, *Ichneumon*)
audax (Förster, 1850, *Pezomachus*)
cautus (Förster, 1850, *Pezomachus*)
circumcinctus (Förster, 1850, *Pezomachus*)
fraudulentus (Förster, 1850, *Pezomachus*)
integer (Förster, 1850, *Pezomachus*)
providus (Förster, 1850, *Pezomachus*)
sericeus (Förster, 1850, *Pezomachus*)
cruentatus (Rudow, 1917, *Pezomachus*)
fulvicornis (Rudow, 1917, *Pezomachus*)
unicinctus (Rudow, 1917, *Pezomachus*)
muscae Pisica & Fabritius, 1986

#### Gelis agilis

(Fabricius, 1775)

Ichneumon agilis Fabricius, 1775
cursor (Schrank, 1780, *Ichneumon*)
fuscicornis (Retzius, 1783, *Ichneumon*)
ruficornis (Retzius, 1783, *Ichneumon*) synonymy by Schwarz (2002)
apterus (Geoffroy, 1785, *Ichneumon*)
celer (Olivier, 1792, *Ichneumon*)
instabilis (Förster, 1850, *Pezomachus*)
mediocris (Förster, 1850, *Pezomachus*)
thoracicus (Brischke, 1878, *Pezomachus*)
breviceps (Thomson, 1884, *Pezomachus*)
alpigena (Strobl, 1901, *Pezomachus*)
rossicus (Szépligeti, 1901, *Pezomachus*)
albulae (Rudow, 1917, *Pezomachus*)
cuculliae (Rudow, 1917, *Pezomachus*)
eupitheciae (Rudow, 1917, *Pezomachus*)
intrans (Rudow, 1917, *Pezomachus*)
lineatus (Rudow, 1917, *Pezomachus*)
microrum (Rudow, 1917, *Pezomachus*)
monozonius (Rudow, 1917, *Pezomachus*) preocc.
nigerrimus (Rudow, 1917, *Pezomachus*) preocc.
rosarum (Rudow, 1917, *Pezomachus*)
rufostictus (Rudow, 1917, *Pezomachus*)
vanessae (Rudow, 1917, *Pezomachus*)
leucurus Ulbricht, 1926
laricellae (Fahringer, 1937, *Pezomachus*)
cephalotes Hellén, 1970

##### Distribution

England, Scotland, Wales, Isle of Man

#### Gelis albicinctoides

Schwarz, 1998

##### Distribution

England

##### Notes

added by Schwarz (1998)

#### Gelis albipalpus

(Thomson, 1884)

Hemiteles albipalpus Thomson, 1884
austriacus (Fahringer, 1937, *Hemiteles*)

##### Distribution

England, Scotland, Ireland

#### Gelis albopilosus

Schwarz, 2002

##### Distribution

England

##### Notes

added by Schwarz (2002)

#### Gelis anthracinus

(Förster, 1850)

Pezomachus anthracinus Förster, 1850
linearis (Förster, 1851, *Pezomachus*)
gonatopinus (Thomson, 1884, *Pezomachus*)

##### Distribution

England, Scotland, Ireland

#### Gelis areator

(Panzer, 1804)

Icneumon areator Panzer, 1804
aberrans (Gravenhorst, 1829, *Pezomachus*)
orbiculatus (Gravenhorst, 1829, *Hemiteles*)
pulchellus (Gravenhorst, 1829, *Hemiteles*)
coelebs (Ratzeburg, 1852, *Hemiteles*)
variabilis (Ratzeburg, 1852, *Hemiteles*) synonymy by Horstmann (2001b)
ephippium (Rudow, 1886, *Hemimachus*)
microgastri (Rudow, 1886, *Hemiteles*)
ruficollis (Rudow, 1886, *Hemiteles*) preocc.
cognatus (Brischke, 1891, *Hemiteles*) synonymy by Horstmann (2001b)
minimus (Glowacki, 1967, *Hemiteles*)

##### Distribution

England, Scotland, Wales, Ireland

#### Gelis avarus

(Förster, 1850)

Pezomachus avarus Förster, 1850

##### Distribution

England, Scotland, Wales, Ireland

##### Notes

added by Schwarz and Shaw (1999)

#### Gelis balteatus

(Thomson, 1885)

Hemiteles balteatus Thomson, 1885
brevistylus (Hellén, 1967, *Charitopes*)

##### Distribution

England, Wales

##### Notes

Listed by Fitton (1978) as a doubtfully placed species of *Hemiteles*.

#### Gelis bicolor

(Villers, 1789)

Ichneumon bicolor Villers, 1789
alacer (Förster, 1850, *Pezomachus*)
brachyurus (Förster, 1850, *Pezomachus*)
distinctus (Förster, 1850, *Pezomachus*)
furtivus (Förster, 1850, *Pezomachus*)
incertus (Förster, 1850, *Pezomachus*)
molestus (Förster, 1850, *Pezomachus*)
muelleri (Förster, 1850, *Pezomachus*)
petulans (Förster, 1850, *Pezomachus*)
sordidus (Förster, 1850, *Pezomachus*)
spadiceus (Förster, 1850, *Pezomachus*)
timidus (Förster, 1850, *Pezomachus*)
vicinus (Förster, 1850, *Pezomachus*)
fusculus (Förster, 1851, *Pezomachus*)
rigii (De Stefani, 1884, *Pezomachus*)
facialis (Brishcke, 1891, *Pezomachus*)
riggioi (Schmiedeknecht, 1906, *Pezomachus*)
aphidicola (Rudow, 1917, *Pezomachus*)
formicarius (Rudow, 1917, *Pezomachus*) preocc.
Gelis bicolor ?*latus* Jonaitis, 1981

##### Distribution

England, Scotland

#### Gelis brevis

(Bridgman, 1883)

Pezomachus brevis Bridgman, 1883

#### Gelis caudatulus

Horstmann, 1997

caudator Horstmann, 1986

##### Distribution

Scotland

##### Notes

added by Schwarz and Shaw (1999)

#### Gelis cayennator

(Thunberg, 1824)

Ichneumon cayennator Thunberg, 1824
brassicae Horstmann, 1986 synonymy by Schwarz (2009)
sulcatus (Blunck, 1951, *Hemiteles*) preocc.

##### Distribution

England, Scotland, Wales

##### Notes

added by Schwarz and Shaw (1999)

#### Gelis cinctus

(Linnaeus, 1758)

Ichneumon cinctus Linnaeus, 1758
cinctor (Thunberg, 1824, *Ichneumon*)
bicolorinus (Gravenhorst, 1829, *Hemiteles*)

##### Distribution

England, Scotland

#### Gelis cursitans

(Fabricius, 1775)

Ichneumon cursitans Fabricius, 1775
tuberculatus (Hartig, 1838, *Pezomachus*)
decipiens (Förster, 1850, *Pezomachus*)
peregrinator (Förster, 1850, *Pezomachus*)
alpinus (Rudow, 1917, *Pezomachus*) preocc.
braconidum (Rudow, 1917, *Pezomachus*)
helicis (Rudow, 1917, *Pezomachus*)
psychivorus (Rudow, 1917, *Pezomachus*)

#### Gelis curvicauda

Horstmann, 1993

##### Distribution

England

##### Notes

added by Schwarz (1994)

#### Gelis discedens

(Förster, 1850)

Pezomachus discedens Förster, 1850
vagans misident.
calvus (Förster, 1850, *Pezomachus*)
quaesitorius (Förster, 1850, *Pezomachus*)
collaris (Rudow, 1917, *Pezomachus*)
exareolatus (Rudow, 1917, *Pezomachus*) preocc.
potentillae (Rudow, 1917, *Pezomachus*)
psychidum (Rudow, 1917, *Pezomachus*)
nigrithorax (Habermehl, 1920, *Pezomachus*)

##### Distribution

England, Scotland, Wales

#### Gelis divaricatus

Horstmann, 1993

##### Distribution

England, Wales

##### Notes

added by Horstmann (1993a)

#### Gelis edentatus

(Förster, 1850)

Pezomachus edentatus Förster, 1850
imbellis (Förster, 1850, *Pezomachus*)
modestus (Förster, 1850, *Pezomachus*)
vagantiformis (Bridgman, 1886, *Pezomachus*)
dusmeti Ceballos, 1925

##### Distribution

England

#### Gelis exareolatus

(Förster, 1850)

Pezomachus exareolatus Förster, 1850
nigritus (Förster, 1850, *Pezomachus*)
simulans (Förster, 1850, *Pezomachus*)
micromelas (Kriechbaumer, 1894, *Phygadeuon*)
lapponicus Hellén, 1970

##### Distribution

England, Scotland

#### Gelis falcatus

Horstmann, 1986

##### Distribution

Scotland

##### Notes

added by Horstmann (1986)

#### Gelis fallax

(Förster, 1850)

Pezomachus fallax Förster, 1850
nigricornis (Förster, 1850, *Pezomachus*) preocc.
iglesiasi Ceballos, 1925

##### Distribution

Wales, Ireland

#### Gelis fasciitinctus

(Dalla Torre, 1901)

Hemiteles fasciitinctus Dalla Torre, 1901
fasciipennis (Brischke, 1881, *Hemiteles*) preocc.

##### Distribution

England, Scotland

##### Notes

added by Schwarz and Shaw (1999)

#### Gelis festinans

(Fabricius, 1798)

Ichneumon festinans Fabricius, 1798
nanus (Förster, 1850, *Pezomachus*)
pothumus (Förster, 1850, *Pezomachus*)
pumilus (Förster, 1850, *Pezomachus*)
tener (Förster, 1850, *Pezomachus*)
anguinus (Förster, 1851, *Pezomachus*)
ocissimus (Förster, 1851, *Pezomachus*)
brunneus (Brischke, 1890, *Pezomachus*)

##### Distribution

England, Scotland, Wales, Ireland

#### Gelis formicarius

(Linnaeus, 1758)

Mutilla formicaria Linnaeus, 1758
ratzeburgi (Förster, 1850, *Pezomachus*)
confusus (Bridgman, 1883, *Hemimachus*) synonymy by Schwarz and Shaw (1999)
verrucosus (Rudow, 1917, *Pezomachus*)

##### Distribution

England

#### Gelis forticornis

(Förster, 1850)

Pezomachus forticornis Förster, 1850
manevali Seyrig, 1927

##### Distribution

England

##### Notes

added by Schwarz (1998)

#### Gelis fuscicornis

(Retzius, 1783)

Ichneumon fuscicornis Retzius, 1783
longulus (Zetterstedt, 1838, *Cryptus*) synonymy by Schwarz (2002)

##### Distribution

England, Scotland

##### Notes

added by Schwarz and Boriani (1994)

#### Gelis hortensis

(Christ, 1791)

Ichneumon hortensis Christ, 1791
acarorum misident.
callidus (Förster, 1850, *Pezomachus*)
canaliculatus (Förster, 1850, *Pezomachus*)
gentilis (Förster, 1850, *Pezomachus*)
impotens (Förster, 1850, *Pezomachus*)
inermis (Förster, 1850, *Pezomachus*)
latrator (Förster, 1850, *Pezomachus*)
lepidus (Förster, 1850, *Pezomachus*)
xylochophilus (Förster, 1850, *Pezomachus*)
avidus (Förster, 1851, *Pezomachus*)
filicornis (Förster, 1851, *Pezomachus*)
nomas (Förster, 1851, *Pezomachus*)
subtilis (Förster, 1851, *Pezomachus*)
albipennis (Ratzeburg, 1852, *Hemiteles*)

##### Distribution

England, Scotland, Wales, Ireland

#### Gelis intermedius

(Förster, 1850)

Pezomachus intermedius Förster, 1850
furax (Förster, 1850, *Pezomachus*)

##### Distribution

England

##### Notes

reinstated by Schwarz and Shaw (1999)

#### Gelis kiesenwetteri

(Förster, 1850)

Pezomachus kiesenwetteri Förster, 1850
bellicosus (Förster, 1850, *Pezomachus*)
debeyii (Förster, 1850, *Pezomachus*)
egregius (Förster, 1850, *Pezomachus*)
costatus (Bridgmanm, 1886, *Pezomachus*)

##### Distribution

England, Scotland, Ireland

#### Gelis limbatus

(Gravenhorst, 1829)

Hemiteles limbatus Gravenhorst, 1829

##### Notes

Listed by Fitton (1978) as a doubtfully placed species of *Hemiteles*.

#### Gelis liparae

(Giraud, 1863)

Hemiteles liparae Giraud, 1863
ilicicola (Seyrig, 1927, *Hemiteles*) synonymy by Schwarz and Shaw (1999)
ilicicolator Aubert, 1966 synonymy by Schwarz and Shaw (1999)

##### Distribution

England

##### Notes

added by Horstmann (1986)

#### Gelis longicauda

(Thomson, 1884)

Hemiteles longicauda Thomson, 1884

##### Distribution

England, Scotland, Ireland

#### Gelis lucidulus

(Förster, 1850)

Pezomachus lucidulus Förster, 1850
inquilinus (Förster, 1850, *Pezomachus*)
microstylus (Förster, 1851, *Pezomachus*)

#### Gelis mangeri

(Gravenhorst, 1815)

Ichneumon mangeri Gravenhorst, 1815
fulveolatus (Gravenhorst, 1829, *Pezomachus*)
longipennis (Gravenhorst, 1829, *Pezomachus*)
foersteri (Bridgman, 1882, *Aptesis*)

##### Distribution

England, Wales

#### Gelis meigenii

(Förster, 1850)

Pezomachus meigenii Förster, 1850
denudatus (Förster, 1850, *Pezomachus*)
geochares (Förster, 1850, *Pezomachus*)
insolens (Förster, 1850, *Pezomachus*)
Gelis meigenii ?*rufotinctus* (Bridgman, 1883, *Hemimachus*) tentative synonymy by Schwarz and Shaw (1999)
noricus (Strobl, 1901, *Pezomachus*)
ephippium (Rudow, 1914, *Pezomachus*) preocc.
ephippium (Rudow, 1917, *Pezomachus*) preocc.

##### Distribution

England, Wales, Scotland

#### Gelis melanocephalus

(Schrank, 1781)

Mutilla melanocephala Schrank, 1781
fasciatus (Fabricius, 1793, *Ichneumon*) preocc.
fasciatus (Ratzeburg, 1852, *Hemiteles*) preocc.
hercyniae (Rudow, 1917, *Pezomachus*)

##### Distribution

England, Scotland, Wales, Ireland, Isle of Man

#### Gelis melanogaster

(Thomson, 1884)

Hemiteles melanogaster Thomson, 1884

##### Distribution

Wales

##### Notes

Added by Schwarz and Shaw (1999), but it was overlooked that Fitton (1978) had treated this as a species of *Charitopes*.

#### Gelis melanophorus

(Förster, 1851)

Pezomachus melanophorus Förster, 1851
fuscicornis (Förster, 1850, *Pezomachus*) synonymy by Schwarz and Shaw (1999)
foersteri (Bridgman, 1886, *Pezomachus*) synonymy by Schwarz and Shaw (1999)

##### Distribution

England, Scotland, Ireland

##### Notes

added by Schwarz and Shaw (1999)

#### Gelis micrurus

(Förster, 1850)

Pezomachus micrurus Förster, 1850
pardosae (Giard, 1895, *Hemiteles*)

##### Distribution

England, Scotland, Wales, Ireland

#### Gelis mitis

Schwarz, 1994

##### Distribution

England

##### Notes

added by Schwarz (1994)

#### Gelis mutillatus

(Gmelin, 1790)

Ichneumon mutillatus Gmelin, 1790
mutillarius (Fabricius, 1787, *Ichneumon*) preocc.
vagans (Olivier, 1792, *Ichneumon*)
pedicularius (Fabricius, 1793, *Ichneumon*)

#### Gelis nigritulus

(Zetterstedt, 1838)

Cryptus nigritulus Zetterstedt, 1838
terebrator (Ratzeburg, 1848, *Pezomachus*)

##### Distribution

England, Scotland

#### Gelis nitidus

Horstmann, 1986

##### Distribution

England

##### Notes

added by Horstmann (1986)

#### Gelis obscuripes

Horstmann, 1986

##### Distribution

England, Scotland, Wales

##### Notes

added by Horstmann (1986)

#### Gelis papaveris

(Förster, 1856)

Pezomachus papaveris Förster, 1856
hieracii (Bridgman, 1883, *Pezomachus*)
grandiceps (Thomson, 1884, *Pezomachus*)

#### Gelis problemator

Aubert, 1989

##### Distribution

England, Scotland

##### Notes

added by Schwarz (1994)

#### Gelis proximus

(Förster, 1850)

Pezomachus proximus Förster, 1850
analis (Förster, 1850, *Pezomachus*)
attentus (Förster, 1850, *Pezomachus*)
celer (Förster, 1850, *Pezomachus*)
consociatus (Förster, 1850, *Pezomachus*)
corruptor (Förster, 1850, *Pezomachus*)
derasus (Förster, 1850, *Pezomachus*)
dubitator (Förster, 1850, *Pezomachus*)
ephippiger (Förster, 1850, *Pezomachus*)
faunus (Förster, 1850, *Pezomachus*)
hostilis (Förster, 1850, *Pezomachus*)
incubitor (Förster, 1850, *Pezomachus*)
latro (Förster, 1850, *Pezomachus*)
ochraceus (Förster, 1850, *Pezomachus*)
parvulus (Förster, 1850, *Pezomachus*)
sedulus (Förster, 1850, *Pezomachus*)
tonsus (Förster, 1850, *Pezomachus*)
vigil (Förster, 1850, *Pezomachus*)
vorax (Förster, 1850, *Pezomachus*)
xenoctonus (Förster, 1850, *Pezomachus*)
ageletes (Förster, 1851, *Pezomachus*)
ambulans (Förster, 1851, *Pezomachus*)
conveniens (Förster, 1851, *Pezomachus*)
decurtatus (Förster, 1851, *Pezomachus*)
dysalotus (Förster, 1851, *Pezomachus*)
elaphrus (Förster, 1851, *Pezomachus*)
erythropus (Förster, 1851, *Pezomachus*)
fugitivus (Förster, 1851, *Pezomachus*)
heydeni (Förster, 1851, *Pezomachus*)
histrio (Förster, 1851, *Pezomachus*)
imbecillus (Förster, 1851, *Pezomachus*)
indagator (Förster, 1851, *Pezomachus*)
indigator misspelling
insidiosus (Förster, 1851, *Pezomachus*)
inspector (Förster, 1851, *Pezomachus*)
lustrator (Förster, 1851, *Pezomachus*)
migrator (Förster, 1851, *Pezomachus*)
navus (Förster, 1851, *Pezomachus*)
procursorius (Förster, 1851, *Pezomachus*)
prudens (Förster, 1851, *Pezomachus*)
secretus (Förster, 1851, *Pezomachus*)
tentator (Förster, 1851, *Pezomachus*)
versatilis (Förster, 1851, *Pezomachus*)
violentus (Förster, 1851, *Pezomachus*)
hyponomeutae (Bridgman, 1883, *Hemimachus*)
ovatus (Bridgman, 1883, *Hemimachus*) synonymy by Schwarz and Shaw (1999)
rufipes (Bridgman, 1883, *Hemimachus*) synonymy by Schwarz and Shaw (1999)
tricinctus (Brischke, 1891, *Pezomachus*)
evanescens (Kriechbaumer, 1891, *Pezomachus*) unavailable
rufiventris (Kriechbaumer, 1891, *Pezomachus*) unavailable
sesquifasciatus (Kriechbaumer, 1891, *Pezomachus*)
Gelis proximus ?*alpinus* (Strobl, 1901, *Pezomachus*)
transsylvanicus (Kiss, 1915, *Pezomachus*)
borealis (Rudow, 1917, *Pezomachus*)
retiniae (Rudow, 1917, *Pezomachus*)
versicolor (Rudow, 1917, *Pezomachus*)
parisiensis Aubert, 1957
inflatipes Hellén, 1970

##### Distribution

England, Scotland, Wales, Ireland, Isle of Man

#### Gelis pulicarius

(Fabricius, 1793)

Ichneumon pulicarius Fabricius, 1793
hoffmannseggii (Gravenhorst, 1815, *Ichneumon*)

#### Gelis recens

Schwarz, 2002

##### Distribution

England

##### Notes

added by Schwarz (2002)

#### Gelis rufipes

(Förster, 1850)

Pezolochus rufipes Förster, 1850
aries (Förster, 1850, *Pezomachus*)
ecarinatus (Förster, 1850, *Pezomachus*)

##### Distribution

England, Ireland

##### Notes

added by Schwarz and Shaw (1999)

#### Gelis rufogaster

Thunberg, 1827

aemulus (Förster, 1850, *Pezomachus*)
alienus (Förster, 1850, *Pezomachus*)
anceps (Förster, 1850, *Pezomachus*)
astutus (Förster, 1850, *Pezomachus*)
bicinctus (Förster, 1850, *Pezomachus*)
carnifex (Förster, 1850, *Pezomachus*)
consobrinus (Förster, 1850, *Pezomachus*)
currens (Förster, 1850, *Pezomachus*)
debilis (Förster, 1850, *Pezomachus*)
detritus (Förster, 1850, *Pezomachus*)
emarcidus (Förster, 1850, *Pezomachus*)
flavipes (Förster, 1850, *Pezomachus*)
gracilis (Förster, 1850, *Pezomachus*)
helvolus (Förster, 1850, *Pezomachus*)
immaturus (Förster, 1850, *Pezomachus*)
insectator (Förster, 1850, *Pezomachus*)
juvenilis (Förster, 1850, *Pezomachus*)
languidus (Förster, 1850, *Pezomachus*)
lividus (Förster, 1850, *Pezomachus*)
lugubris (Förster, 1850, *Pezomachus*)
lutescens (Förster, 1850, *Pezomachus*)
puberulus (Förster, 1850, *Pezomachus*)
puerilis (Förster, 1850, *Pezomachus*)
pulcher (Förster, 1850, *Pezomachus*)
pulex (Förster, 1850, *Pezomachus*)
rufulus (Förster, 1850, *Pezomachus*)
scitulus (Förster, 1850, *Pezomachus*)
squalidus (Förster, 1850, *Pezomachus*)
unicolor (Förster, 1850, *Pezomachus*)
venustus (Förster, 1850, *Pezomachus*)
annulicornis (Bridgman, 1883, *Hemimachus*)
areneicola (Rudow, 1914, *Pezomachus*)
aphidum (Rudow, 1917, *Pezomachus*)
areneicolus (Rudow, 1917, *Pezomachus*) preocc.
balteatus (Rudow, 1917, *Pezomachus*) preocc.
isabellinus (Rudow, 1917, *Pezomachus*)
pemphigicola (Rudow, 1917, *Pezomachus*)
pieridis (Rudow, 1917, *Pezomachus*)
ulmicola (Rudow, 1917, *Pezomachus*)

##### Distribution

England, Scotland, Wales, Ireland

##### Notes

*Pezomachus annulicornis* synonymised with *rufulus* (Förster), now a synonym of *rufogaster*, listed as a separate species in Yu and Horstmann (1997) but synonymy confirmed by Schwarz and Shaw (1999).

#### Gelis rugifer

(Thomson, 1884)

Hemiteles rugifer Thomson, 1884

##### Distribution

England, Scotland, Wales

#### Gelis seyrigi

Ceballos, 1925

##### Distribution

England

##### Notes

added by Schwarz (1998)

#### Gelis spinula

(Thomson, 1884)

Pezomachus spinula Thomson, 1884

##### Distribution

England, Scotland, Ireland

#### Gelis spurius

(Förster, 1850)

Pezomachus spurius Förster, 1850
ruficornis misident. in Schwarz and Shaw (1999)Schwarz (2002)

##### Distribution

England, Scotland

#### Gelis terribilis

Schwarz, 2002

##### Distribution

England, Scotland

##### Notes

added by Schwarz (2002)

#### Gelis thomsoni

(Schmiedeknecht, 1933)

Hemiteles thomsoni Schmiedeknecht, 1933
dispar (Thomson, 1885, *Hemiteles*) preocc.

##### Distribution

England

##### Notes

Listed by Fitton (1978) as a doubtfully placed species of *Hemiteles*.

#### Gelis trux

(Förster, 1850)

Pezomachus trux Förster, 1850
ruficornis Thunberg, 1827 preocc.
blandus (Förster, 1850, *Pezomachus*)
comes (Förster, 1850, *Pezomachus*)
transfuga (Förster, 1850, *Pezomachus*)

##### Distribution

England, Wales

#### Gelis viduus

(Förster, 1850)

Pezomachus viduus Förster, 1850
congruus (Förster, 1850, *Pezomachus*)
doliopus (Förster, 1851, *Pezomachus*)
mandibularis (Thomson, 1884, *Pezomachus*)

##### Distribution

England, Scotland, Wales, Ireland

#### Gelis vulnerans

(Förster, 1850)

Pezomachus vulnerans Förster, 1850
affinis (Magretti, 1884, *Pezomachus*)

#### Gelis zeirapherator

(Aubert, 1966)

Alegina zeirapherator Aubert, 1966

##### Distribution

Scotland

##### Notes

added by Schwarz and Shaw (1999)

#### Glyphicnemis

Förster, 1869

GNATHOCRYPTUS Thomson, 1873

##### Notes

Some distribution data from Fitton (1976) and Sawoniewicz (1985).

#### Glyphicnemis atrata

(Strobl, 1901)

Stylocryptus atratus Strobl, 1901
alpina (Strobl, 1901, *Stylocryptus*)
suffolciensis Morley, 1907

##### Distribution

England, Scotland, Wales

#### Glyphicnemis clypealis

(Thomson, 1883)

Stylocryptus clypealis Thomson, 1883

##### Distribution

England

#### Glyphicnemis profligator

(Fabricius, 1775)

Ichneumon profligator Fabricius, 1775
abdominalis (Geoffroy, 1785, *Ichneumon*)
nigricornis (Gmelin, 1790, *Ichneumon*) preocc.
textor (Thunberg, 1824, *Ichneumon*)
frequentoria (Zetterstedt, 1838, *Ichneumon*)
pygmaea (Habermehl, 1916, *Stylocryptus*)
ruficoxis (Habermehl, 1916, *Stylocryptus*) preocc.

##### Distribution

England, Scotland, Wales, Ireland, Isle of Man

#### Glyphicnemis vagabunda

(Gravenhorst, 1829)

Phygadeuon vagabundus Gravenhorst, 1829
podagrica (Gravenhorst, 1829, *Phygadeuon*)
exannulata (Hedwig, 1956, *Stylocryptus*)

##### Distribution

England

#### Gnotus

Förster, 1869

#### Gnotus chionops

(Gravenhorst, 1829)

Hemiteles chionops Gravenhorst, 1829
scutellator (Lange, 1911, *Hemiteles*)

##### Distribution

England, Scotland

#### Gnotus macrurus

(Thomson, 1884)

Hemiteles macrurus Thomson, 1884

##### Distribution

Scotland

##### Notes

Listed as a doubtfully placed species of *Hemiteles* by Fitton (1978).

#### Gnotus rugipectus

(Thomson, 1886)

Phygadeuon rugipectus Thomson, 1886

##### Notes

Transferred from *Phygadeuon* by Horstmann (1998a).

#### Gnotus tenuipes

(Gravenhorst, 1829)

Phygadeuon tenuipes Gravenhorst, 1829
tenuicornis (Gravenhorst, 1829, *Hemiteles*)
nebulosus (Rudow, 1886, *Hemiteles*)
cryptiformis (Kiss, 1924, *Hemiteles*)
nigripes (Bauer, 1958, *Panargyrops*)

##### Distribution

England

#### Gnypetomorpha

Förster, 1869

TRISACRA Förster, 1869
VICTOROVIA Tobias, 1963

#### Gnypetomorpha obscura

(Bridgman, 1883)

Hemiteles obscurus Bridgman, 1883
aperta (Thomson, 1884, *Hemiteles*)

##### Distribution

England, Scotland

#### Gnypetomorpha tubertae

Horstmann, 2012

##### Distribution

England

##### Notes

added by Horstmann (2012a)

#### Grasseiteles

Aubert, 1965

DIAGLYPTELLANA Horstmann, 1976 synonymy by Schwarz (2005)

#### Grasseiteles opaculus

(Thomson, 1884)

Hemiteles opaculus Thomson, 1884

##### Distribution

England, Scotland

##### Notes

Not mentioned by Schwarz and Shaw (2000); included in Fitton (1978) and recently identified by M. Schwarz from material in NMS.

#### Grasseiteles punctus

(Holmgren, 1857)

Adelognathus punctus Holmgren, 1857
sisyphii (Verhoeff, 1891, *Hemiteles*)
punctata Horstmann, 1986 synonymy by Schwarz and Shaw (2000)

##### Distribution

England, Scotland

##### Notes

added by Schwarz and Shaw (2000)

#### Hemiteles

Gravenhorst, 1829

OCYMORUS Förster, 1869

##### Notes

Many species listed as doubtfully placed species of *Hemiteles* by Fitton (1978) have since been transferred to other genera of Phygadeuontini. *Hemiteles niger* (Taschenberg) is now recognised to be a species of *Thymaris* (Tryphoninae).

doubtfully placed species of *Hemiteles*:

[*liambus* Thomson, 1885 nom. dub.]

[*piceus* (Bridgman, 1883, *Hemimachus*) nom. dub., from England; Fitton (1976)]

#### Hemiteles bipunctator

(Thunberg, 1824)

Ichneumon bipunctator Thunberg, 1824
cingulator Gravenhorst, 1829
tristator Gravenhorst, 1829
rufipleuris Szépligeti, 1901

##### Distribution

England, Scotland, Ireland

#### Hemiteles maricesca

Schwarz & Shaw, 2000

##### Distribution

England, Scotland, Wales

##### Notes

added by Schwarz and Shaw (2000)

#### Hemiteles rubropleuralis

Kiss, 1929

##### Distribution

Scotland

##### Notes

added by Schwarz and Shaw (2000)

#### Hemiteles similis

(Gmelin, 1790)

Ichneumon similis Gmelin, 1790
Hemiteles similis ?*debellator* (Schrank, 1781, *Ichneumon*) preocc.
meridionalis Gravenhorst, 1829
unicolor Thomson, 1884

##### Distribution

England, Scotland, Wales, Ireland, Isle of Man

#### Holcomastrus

Horstmann, 2012

#### Holcomastrus bituberculatus

(Schmiedeknecht, 1905)

Hemiteles bituberculatus Schmiedeknecht, 1905

##### Distribution

England

##### Notes

BMNH, det. Broad, added here

#### Isadelphus

Förster, 1869

PEROSIS Förster, 1869
CECIDONOMUS Bridgman, 1880

##### Notes

See note for *Mastrus*.

#### Isadelphus armatus

(Gravenhorst, 1829)

Echthrus armatus Gravenhorst, 1829
mandibulator (Dufour & Perris, 1840, *Anomalon*)
bidentulus (Thomson, 1844, *Hemiteles*)

##### Distribution

England, Wales

#### Isadelphus coriarius

(Taschenberg, 1865)

Hemiteles coriarius Taschenberg, 1865
rixator (Woldstedt, 1877, *Polyblastus*)
trochanteratus (Strobl, 1903, *Erromenus*) synonymy by Horstmann (1999a)
carbonarius (Schmiedeknecht, 1905, *Hemiteles*)

#### Isadelphus gallicola

(Bridgman, 1880)

Cecidonomus gallicola Bridgman, 1880
nigriventris (Thomson, 1884, *Hemiteles*)
carpathicus (Kiss, 1924, *Lissonota*)

##### Distribution

England, Scotland

#### Isadelphus inimicus

(Gravenhorst, 1829)

Hemiteles inimicus Gravenhorst, 1829
rufus (Bridgman, 1880, *Cecidonomus*)
obscuripes (Thomson, 1884, *Hemiteles*)

##### Distribution

England, Scotland, Wales, Ireland, Isle of Man

#### Isadelphus longisetosus

(Schmiedeknecht, 1897)

Hemiteles longisetosus Schmiedeknecht, 1897
added by

##### Distribution

England, Scotland

##### Notes

added by Horstmann (2009b); treated as a separate species by Horstmann (2009b), rather than as a synonym of *inimicus*, as listed in Yu and Horstmann (1997).

#### Isadelphus minutus

Horstmann, 2009

##### Distribution

England, Scotland

##### Notes

added by Horstmann (2009b)

#### Leptocryptoides

Horstmann, 1976

#### Leptocryptoides clavipes

(Thomson, 1888)

Leptocryptus clavipes Thomson, 1888

##### Distribution

England, Ireland

##### Notes

added by Schwarz and Shaw (2011)

#### Lochetica

Kriechbaumer, 1892

##### Notes

Some distribution data from Townes (1983).

#### Lochetica westoni

(Bridgman, 1880)

Cecidonomus westoni Bridgman, 1880
pimplaria (Thomson, 1884, *Phygadeuon*) synonymy by Townes (1983)

##### Distribution

England, Scotland

#### Lysibia

Förster, 1869

PEMON Förster, 1869
STIBOSCOPUS Förster, 1869
HAPLASPIS Townes, 1944

##### Notes

Some distribution data from Perkins (1962) and Townes (1983).

#### Lysibia ceylonensis

(Kerrich, 1956)

Haplaspis ceylonensis Kerrich, 1956
proxima (Perkins, 1962, *Pemon*)

##### Distribution

England

#### Lysibia nanus

(Gravenhorst, 1829)

Tryphon nanus Gravenhorst, 1829
fulvipes (Gravenhorst, 1829, *Hemiteles*)
socialis (Ratzeburg, 1844, *Hemiteles*)
populnea (Boie, 1855, *Hemiteles*)

##### Distribution

England, Scotland, Wales, Ireland, Isle of Man

#### Lysibia tenax

Townes, 1983

##### Distribution

Scotland, Ireland

##### Notes

added by Townes (1983)

#### Mastrulus

Horstmann, 1978

#### Mastrulus marshalli

(Bridgman & Fitch, 1882)

Phygadeuon marshalli Bridgman & Fitch, 1882
marshalli (Bridgman, 1883, *Phygadeuon*) preocc.
capra (Thomson, 1884, *Hemiteles*)
disputabilis (Schmiedeknecht, 1897, *Hemiteles*)

##### Distribution

England, Scotland

##### Notes

Listed as a species of *Theroscopus* by Fitton (1978).

#### Mastrus

Förster, 1869

AENOPLEX Förster, 1869
DAICTES Förster, 1869

##### Notes

*Mastrus* *sensu* Townes (1970) was split by Horstmann (1978b) into *Isadelphus*, *Mastrus*, *Micromonodon*, *Odontoneura* and *Zoophthorus* (and subsequently *Fianoniella* and *Odontomastrus*).

species of *Mastrus* excluded from the British and Irish list:

[*pictipes* (Gravenhorst, 1829, *Hemiteles*)] Listed as a doubtfully placed species of *Hemiteles* by Fitton (1978). Specimens under the name *pictipes* in the BMNH have now been identified as *albobasalis* and *longicauda* and there is no evidence that *pictipes* has ever been found here.

#### Mastrus albobasalis

(Schmiedeknecht, 1933)

Hemiteles albobasalis Schmiedeknecht, 1933

##### Distribution

England

##### Notes

added by Schwarz and Shaw (2010)

#### Mastrus boreaphilus

(Roman, 1939)

Cecidonomus boreaphilus Roman, 1939

##### Distribution

Scotland

##### Notes

added bySchwarz and Shaw (2010)

#### Mastrus costalis

(Thomson, 1884)

Hemiteles costalis Thomson, 1884

##### Distribution

England, Scotland

##### Notes

added by Schwarz and Shaw (2010)

#### Mastrus deminuens

(Hartig, 1838)

Hemiteles deminuens Hartig, 1838
castaneus (Taschenberg, 1865, *Hemiteles*)
bredensis (Smits$)
ripicola (Habermehl, 1920, *Hemiteles*)

##### Distribution

England, Scotland, Wales, Ireland

#### Mastrus fumipennis

(Thomson, 1884)

Hemiteles fumipennis Thomson, 1884

##### Distribution

Ireland

##### Notes

Listed as a doubtfully placed species of *Hemiteles* by Fitton (1978).

#### Mastrus longicauda

Horstmann, 1990

##### Distribution

England

##### Notes

BMNH, det. Broad & Horstmann, added here

#### Mastrus longulus

Horstmann, 1990

##### Distribution

England

##### Notes

added by Horstmann (1990a)

#### Mastrus mandibularis

Horstmann, 1990

##### Distribution

England

##### Notes

added by Horstmann (1990a)

#### Mastrus parviceps

(Hellén, 1967)

Isadelphus parviceps Hellén, 1967

##### Distribution

Scotland

##### Notes

added by Schwarz and Shaw (2010)

#### Mastrus ridibundus

(Gravenhorst, 1829)

Hemiteles ridibundus Gravenhorst, 1829

##### Distribution

England, Ireland

##### Notes

Listed as a doubtfully placed species of *Hemiteles* by Fitton (1978).

#### Mastrus rufobasalis

(Habermehl, 1920)

Hemiteles Habermehl, 1920

##### Distribution

Isle of Man

##### Notes

added by Schwarz and Shaw (2010); tentative identification by K. Horstmann.

#### Mastrus rufulus

(Thomson, 1884)

Hemiteles rufulus Thomson, 1884
nigrobasalis (Schmiedeknecht, 1905, *Hemiteles*)
rusticus (Habermehl, 1920, *Hemiteles*)
nigricoxis (Hedwig, 1959, *Phygadeuon*) unavailable

##### Distribution

England, Ireland

##### Notes

Listed as a doubtfully placed species of *Hemiteles* by Fitton (1978).

#### Mastrus silbernageli

(Kiss, 1929)

Hemiteles silbernageli Kiss, 1929
leptocryptoides (Schmiedeknecht, 1933, *Hemiteles*)

##### Distribution

England, Scotland, Wales

##### Notes

added by Schwarz and Shaw (2010)

#### Mastrus sordipes

(Gravenhorst, 1829)

Hemiteles sordipes Gravenhorst, 1829
karpinskii (Glowacki, 1967, *Hemiteles*)

##### Distribution

England, Scotland

##### Notes

Listed as a doubtfully placed species of *Hemiteles* by Fitton (1978).

#### Mastrus tenuicosta

(Thomson, 1884)

Phygadeuon tenuicosta Thomson, 1884

##### Distribution

England, Scotland

##### Notes

added by Schwarz and Shaw (2010)

#### Mastrus varicoxis

(Taschenberg, 1865)

Hemiteles varicoxis Taschenberg, 1865
Mastrus varicoxis ?*coactus* (Ratzeburg, 1852, *Hemiteles*)
tricoloripes (Schmiedeknecht, 1932, *Hemiteles*)

##### Distribution

England, Scotland, Ireland

##### Notes

Listed as a doubtfully placed species of *Hemiteles* by Fitton (1978).

#### Medophron

Förster, 1869

BARYNTICA Förster, 1869
HEDYLUS Förster, 1869
SUBHEMITELES Horstmann, 1976

##### Notes

Some distribution data from Horstmann (1998a). Townes (1983), whilst noting that *crassicornis* Ashmead 'might be segregated in a separate genus', retained *crassicornis* as a species of *Medophron*, whilst Yu and Horstmann (1997) follow in treating *Hedylus* as a separate genus. Schwarz and Shaw (2010) follow Townes (1983), noting that separate of *Hedylus* is inconsistent with the synonymy of *Subhemiteles* with *Medophron*.

#### Medophron afflictor

(Gravenhorst, 1829)

Phygadeuon afflictor Gravenhorst, 1829
nigritus (Gravenhorst, 1829, *Phygadeuon*)
niger Brischke, 1881

##### Distribution

Wales

#### Medophron armatulus

(Thomson, 1888)

Phygadeuon armatulus Thomson, 1888

##### Distribution

England, Scotland, Isle of Man

##### Notes

added by Schwarz and Shaw (2010)

#### Medophron crassicornis

(Gravenhorst, 1829)

Hemiteles crassicornis Gravenhorst, 1829

#### Medophron mixtus

(Bridgman, 1883)

Hemiteles mixtus Bridgman, 1883
flavipes (Thomson, 1888, *Phygadeuon*)
mandibularis (Brischke, 1891, *Phygadeuon*)
flavitarsis (Dalla Torre, 1901, *Phygadeuon*)

##### Distribution

England, Ireland

#### Medophron nigriceps

(Thomson, 1883)

Acanthocryptus nigriceps Thomson, 1883
elegans (Schmiedeknecht, 1932, *Stylocryptus*)

##### Distribution

England

#### Medophron nitidus

(Horstmann, 1976)

Subhemiteles nitidus Horstmann, 1976

##### Distribution

England

##### Notes

added by Horstmann (1998a)

#### Medophron recurvus

(Thomson, 1884)

Phygadeuon recurvus Thomson, 1884

##### Distribution

England, Scotland

##### Notes

added by Schwarz and Shaw (2010)

#### Medophron setosus

(Hellén, 1967)

Aclastus setosus Hellén, 1967
crassicornis Ashmead, 1899

##### Distribution

England, Scotland

##### Notes

added by Townes (1983)

#### Megacara

Townes, 1970

##### Notes

Some distribution data from Townes (1983).

#### Megacara hortulana

(Gravenhorst, 1829)

Cryptus hortulanus Gravenhorst, 1829
postica (Wollaston, 1858, *Hemiteles*)
rusticellae (Bridgman, 1886, *Phygadeuon*)
hispanator (Aubert, 1968, *Phygadeuon*)

##### Distribution

England, Scotland, Wales, Ireland

#### Megacara vagans

(Gravenhorst, 1829)

Phygadeuon vagans Gravenhorst, 1829
apicalis (Gravenhorst, 1829, *Cryptus*)
alteareolata (Schmiedeknecht, 1905, *Phygadeuon*)
nova (Kiss, 1929, *Acanthocryptus*)

##### Distribution

England, Scotland, Wales, Ireland

#### Mesoleptus

Gravenhorst, 1829

EXOLYTUS Holmgren, 1859

##### Notes

Distribution and syonymic data from Jussila et al. (2010) and the collections of BMNH and NMS (det. Jussila). Jussila et al. (2010) placed a large number of Förster names in synonymy; we have not repeated all of the synonymy here, just listing those names that have appeared in the British literature.

doubtfully placed species of *Mesoleptus*:

[*coarctatus* (Gravenhorst, 1829, *Cryptus*) nom. dub.] Listed as a doubtfully placed species of *Trychosis* by Fitton (1978).

[*mirabilis* Stephens, 1835 nom. dub, from England; Fitton (1976)]

[*speciosus* Curtis, 1837 nom. dub.]

[*splendens* Gravenhorst, 1829 nom. dub.; Jussila et al. (2010)]

[*subcompressus* Stephens, 1835 nom. dub., from England; Fitton (1976)]

#### Mesoleptus congener

(Förster, 1876)

Exolytus congener Förster, 1876

##### Distribution

England, Scotland, Wales, Ireland

##### Notes

added by Jussila et al. (2010)

#### Mesoleptus devotus

(Förster, 1876)

Exolytus devotus Förster, 1876
fulvipes (Förster, 1876, *Exolytus*)
secretus (Förster, 1876, *Exolytus*)
sollicitus (Förster, 1876, *Exolytus*)

##### Distribution

England

#### Mesoleptus distinctus

(Förster, 1876)

Exolytus distinctus Förster, 1876
flavipes (Thomson, 1884, *Atractodes*)

##### Distribution

England, Scotland, Wales

##### Notes

added by Jussila et al. (2010)

#### Mesoleptus incessor

(Haliday, 1839)

Atractodes incessor Haliday, 1839
scrutator (Haliday, 1839, *Atractodes*)
ambiguus (Förster, 1876, *Exolytus*)
incertus (Förster, 1876, *Exolytus*)
speculum (Förster, 1876, *Exolytus*)
marginatus (Thomson, 1884, *Atractodes*)
petiolaris (Thomson, 1884, *Atractodes*)

##### Distribution

England, Scotland, Ireland

#### Mesoleptus laevigatus

(Gravenhorst, 1820)

Ichneumon laevigatus Gravenhorst, 1820
transversor (Thunberg, 1824, *Ichneumon*)
aequalis (Förster, 1876, *Exolytus*)
transsylvanicus (Kiss, 1924, *Exolytus*)

##### Distribution

England

#### Mesoleptus laticinctus

(Walker, 1874)

Mesostenus laticinctus Walker, 1874
angustulus (Förster, 1876, *Exolytus*)
ruficoxatus (Förster, 1876, *Exolytus*)
filicornis (Thomson, 1884, *Atractodes*)

##### Distribution

England, Scotland, Wales

##### Notes

added by Jussila et al. (2010); if *ruficoxatus* is raised to species level, this should also be on the list, based on specimens from England and Scotland in the NMS, det. Jussila. *Mesoleptus laticinctus* *s.s.* has not been recorded from Scotland.

#### Mesoleptus pronus

(Förster, 1876)

Exolytus pronus Förster, 1876
consortius (Förster, 1876, *Exolytus*)

##### Distribution

England, Scotland, Wales

##### Notes

added by Jussila et al. (2010)

#### Mesoleptus vigilatorius

(Förster, 1876)

Exolytus vigilatorius Förster, 1876
ripicola (Thomson, 1884, *Atractodes*)

##### Distribution

England, Scotland, Wales

#### Micromonodon

Förster, 1869

HEMICRYPTUS Kriechbaumer, 1893

##### Notes

See note for *Mastrus*.

#### Micromonodon tener

(Kriechbaumer, 1893)

Hemicryptus tener Kriechbaumer, 1893

##### Distribution

England

##### Notes

added by Laurenne et al. (2006)

#### Neopimpla

Ashmead, 1900

#### Neopimpla aleiodesi

Schwarz & Shaw, 2000

##### Distribution

England

##### Notes

added by Schwarz and Shaw (2000)

#### Obisiphaga

Morley, 1907

#### Obisiphaga stenoptera

(Marshall, 1868)

Aptesis stenoptera Marshall, 1868
longicauda (Vollenhoven, 1873, *Aptesis*)
similis (Brischke, 1891, *Thysiotorus*) invalid
dimidiatipennis (Schmiedeknecht, 1905, *Hemiteles*)
ineptipennis (Speiser, 1908, *Hemiteles*)

##### Distribution

Scotland, Wales, Ireland

#### Odontoneura

Förster, 1869

##### Notes

See note for *Mastrus*.

#### Odontoneura annulicornis

(Thomson, 1884)

Phygadeuon annulicornis Thomson, 1884
csikii (Szépligeti, 1901, *Phygadeuon*)
formosa (Pfankuch, 1921, *Phygadeuon*)

##### Distribution

England, Scotland

##### Notes

Listed as a species of *Theroscopus* by Fitton (1978).

#### Odontoneura sp. A

##### Distribution

Scotland

##### Notes

added by Schwarz and Shaw (2010); to be described by the late K. Horstmann (in prep.).

#### Oecotelma

Townes, 1970

##### Notes

Townes (1970) refers to an undetermined species from Ireland.

#### Orthizema

Förster, 1869

NAETES Förster, 1869
PHYZELUS Förster, 1869

##### Notes

Some distribution data from Fitton (1976) and Horstmann (1993c).

#### Orthizema amabile

(Hedwig, 1939)

Hemiteles amabilis Hedwig, 1939

##### Distribution

England

##### Notes

added by Horstmann (1993c)

#### Orthizema francescae

Schwarz & Shaw, 2011

##### Distribution

England, Scotland, Wales

##### Notes

added by Schwarz and Shaw (2011)

#### Orthizema graviceps

(Marshall, 1868)

Aptesis graviceps Marshall, 1868

##### Distribution

England, Wales

#### Orthizema hadrocerum

(Thomson, 1884)

Hemiteles hadrocerus Thomson, 1884
fasciatum (Brischke, 1888, *Phyzelus*)
rufum (Brischke, 1892, *Hemiteles*)

##### Distribution

England, Scotland

#### Orthizema obscurum

Horstmann, 1993

##### Notes

added by Horstmann (1993c)

#### Orthizema subannulatum

(Bridgman, 1883)

Hemiteles subannulatus Bridgman, 1883
maculipennis (Rudow, 1886, *Hemiteles*)
ornatum (Brischke, 1890, *Hemiteles*)

##### Distribution

England, Scotland

#### Orthizema triannulatum

(Thomson, 1884)

Hemiteles triannulatus Thomson, 1884

##### Distribution

England, Scotland

##### Notes

Listed as a doubtfully placed species of *Hemiteles* by Fitton (1978).

#### Phygadeuon

Gravenhorst, 1829

APTEROPHYGAS Förster, 1869
BATHYMETIS Förster, 1869
ERNOCTONA Förster, 1869
GUNOPACHES Förster, 1869
HABROMMA Förster, 1869
HOMELYS Förster, 1869
ISELIX Förster, 1869
ISOCHRESTA Förster, 1869a
PANTOLISPA Förster, 1869
ZAPHLEGES Förster, 1869
ISCHNOCRYPTUS Kriechbaumer, 1892

##### Notes

Distribution data from Fitton (1976), Horstmann (1993a), Horstmann (2001d) and Schwarz and Shaw (2011).

#### Phygadeuon acutipennis

Thomson, 1884

##### Distribution

England, Scotland

#### Phygadeuon atropos

Kriechbaumer, 1892

##### Distribution

England

##### Notes

added by Horstmann (2001d); listed as a synonym of *forticornis* by Yu and Horstmann (1997).

#### Phygadeuon brachyurus

Thomson, 1884

#### Phygadeuon brevitarsis

Thomson, 1884

##### Distribution

England

##### Notes

added by Horstmann (2001d); listed as a synonym of *hercynicus* by Yu and Horstmann (1997).

#### Phygadeuon canaliculatus

Thomson, 1889

#### Phygadeuon cephalotes

Gravenhorst, 1829

transfuga (Gravenhorst, 1829, *Ichneumon*)

#### Phygadeuon clotho

Kriechbaumer, 1892

grossae Horstmann, 1981

##### Distribution

England, Scotland, Wales

##### Notes

added by Horstmann (1981b)

#### Phygadeuon clypearis

Strobl, 1901

##### Distribution

England

##### Notes

added by Horstmann (2012a)

#### Phygadeuon cubiceps

Thomson, 1884

#### Phygadeuon cylindraceus

Ruthe, 1859

sudvoldensis Morley, 1947

##### Distribution

England, Scotland

#### Phygadeuon detestator

(Thunberg, 1824)

Ichneumon detestator Thunberg, 1824

#### Phygadeuon devonensis

Morley, 1947

##### Distribution

England

##### Notes

*Phygadeuon neoflavicans* Horstmann, 1967 was removed from synonymy by Horstmann (2008a).

#### Phygadeuon dimidiatus

Thomson, 1884

cylindricus Brischke, 1891

#### Phygadeuon dromicus

(Gravenhorst, 1815)

Ichneumon Gravenhorst, 1815

##### Notes

Listed as a doubtfully placed species of *Hemiteles* by Fitton (1978). Male specimens in BMNH identified as *dromicus* may not be conspecific with Horstmann’s (Horstmann 1993c) female neotype.

#### Phygadeuon dubius

(Gravenhorst, 1829)

Hemiteles dubius Gravenhorst, 1829
scaposus Thomson, 1884

#### Phygadeuon elegans

(Förster, 1850)

Theroscopus elegans Förster, 1850
cingulatus (Förster, 1850, *Theroscopus*)
confusus Hedwig, 1959 preocc.

##### Distribution

England, Scotland, Wales, Ireland

##### Notes

added by Horstmann (1993c)

#### Phygadeuon elliotti

Morley, 1947

##### Distribution

Scotland

#### Phygadeuon exiguus

Gravenhorst, 1829

gallevensis Morley, 1947

##### Distribution

England

#### Phygadeuon flavimanus

Gravenhorst, 1829

##### Distribution

England

#### Phygadeuon forticornis

Kriechbaumer, 1892

##### Distribution

England

#### Phygadeuon fraternae

Horstmann, 2001

##### Distribution

Scotland

##### Notes

added by Horstmann (2001d)

#### Phygadeuon fumator

Gravenhorst, 1829

lycaenae Rudow, 1886
britannicus Habermehl, 1923
ragensis Morley, 1947

##### Distribution

England, Scotland

#### Phygadeuon geniculatus

Kriechbaumer, 1892

#### Phygadeuon gracilentus

Horstmann, 1997

gracilicornis Horstmann, 1993 preocc.

##### Distribution

England, Scotland

##### Notes

added by Horstmann (1993c)

#### Phygadeuon hercynicus

Gravenhorst, 1829

##### Distribution

England

#### Phygadeuon infelix

Dalla Torre, 1901

inflatus Thomson, 1884 invalid

#### Phygadeuon laeviventris

Thomson, 1884

compactus Morley, 1947

##### Distribution

England

#### Phygadeuon leucostigmus

Gravenhorst, 1829

punctigena Thomson, 1884

##### Distribution

England

#### Phygadeuon liosternus

Thomson, 1886

#### Phygadeuon magnicornis

(Thomson, 1884)

Hemiteles magnicornis Thomson, 1884

##### Notes

Listed as a doubtfully placed species of *Hemiteles* by Fitton (1978).

#### Phygadeuon melanopygus

(Gravenhorst, 1829)

Hemiteles melanopygus Gravenhorst, 1829
validicornis (Thomson, 1884, *Hemiteles*)
semicroceus (Schmiedeknecht, 1897, *Hemiteles*)

##### Distribution

England, Scotland, Isle of Man

##### Notes

Listed as a doubtfully placed species of *Hemiteles* by Fitton (1978), as a species of *Theroscopus* in Yu and Horstmann (1997) and transferred to *Phygadeuon* by Schwarz and Shaw (2011).

#### Phygadeuon nanus

(Gravenhorst, 1829)

Cryptus nanus Gravenhorst, 1829

#### Phygadeuon nigrifemur

Horstmann, 2001

##### Notes

added by Horstmann (2001d)

#### Phygadeuon nitidus

Gravenhorst, 1829

#### Phygadeuon ovaliformis

Dalla Torre, 1901

ovalis Thomson, 1884 preocc.

#### Phygadeuon ovatus

Gravenhorst, 1829

caliginosus Gravenhorst, 1829
montanus (Lange, 1911, *Stylocryptus*)

#### Phygadeuon pallicarpus

Thomson, 1884

pallidicarpus Dalla Torre, 1902

##### Distribution

Scotland, Ireland

#### Phygadeuon palus

Schwarz & Shaw, 2011

##### Distribution

England, Wales

##### Notes

added by Schwarz and Shaw (2011)

#### Phygadeuon paradoxus

(Bridgman, 1889)

Apterophygas paradoxus Bridgman, 1889
hungaricus (Kiss, 1915, *Phygadeuon*)
insulanus Hedwig, 1939

##### Distribution

England

#### Phygadeuon pegomyiae

Habermehl, 1928

#### Phygadeuon punctiventris

Thomson, 1884

#### Phygadeuon rotundipennis

Thomson, 1884

differens Hedwig, 1938

##### Distribution

England, Scotland, Wales, Isle of Man

#### Phygadeuon rubricaudus

Morley, 1947

##### Distribution

England

#### Phygadeuon rugulosus

Gravenhorst, 1829

semipolitus Taschenberg, 1865

#### Phygadeuon subtilis

Gravenhorst, 1829

flavicans Thomson, 1884
oppositus Thomson, 1884
subalpinus Roman, 1909
lincolniae Morley, 1947

##### Distribution

England

#### Phygadeuon surriensis

Morley, 1947

##### Distribution

England

#### Phygadeuon tenuiscapus

Thomson, 1884

#### Phygadeuon thomsoni

Roman, 1925

##### Distribution

England

##### Notes

added by Horstmann (2001d)

#### Phygadeuon trichops

Thomson, 1884

ocularis Thomson, 1889

##### Distribution

Scotland

#### Phygadeuon troglodytes

Gravenhorst, 1829

anthracinus Kriechbaumer, 1894

#### Phygadeuon variabilis

Gravenhorst, 1829

confinis Smits

#### Phygadeuon varicornis

(Gravenhorst, 1829)

Hemiteles varicornis Gravenhorst, 1829

##### Notes

Listed as a doubtfully placed species of *Hemiteles* by Fitton (1978).

#### Phygadeuon vexator

(Thunberg, 1824)

Ichneumon vexator Thunberg, 1824
patellator (Thunberg, 1824, *Ichneumon*)
diaphanus Gravenhorst, 1829
minor Fonscolombe, 1851
nigripes Aubert, 1959

##### Distribution

England

##### Notes

*Phygadeuon domesticae* Horstmann, 1986 was removed from synonymy by Horstmann (2008a).

#### Platyrhabdus

Townes, 1970

##### Notes

Some distribution data from Horstmann (1998a).

#### Platyrhabdus clypeatus

Horstmann, 1998

##### Distribution

England, Scotland, Isle of Man

##### Notes

added by Horstmann (1998a)

#### Platyrhabdus inflatus

(Thomson, 1884)

Hemiteles inflatus Thomson, 1884
rufus (Morley, 1907, *Aritranis*)
elongatus (Smits van Burgst, 1913, *Hemiteles*) preocc.
tunisiae (Morley, 1926, *Hemiteles*)
tunetanus (Schmiedeknecht, 1932, *Hemiteles*)

##### Distribution

England, Scotland, Ireland, Isle of Man

#### Platyrhabdus monodon

(Thomson, 1884)

Hemiteles monodon Thomson, 1884
graciliventris (Schmiedeknecht, 1933, *Hemiteles*)

##### Distribution

England

#### Platyrhabdus nervellator

Horstmann, 1998

##### Distribution

England

##### Notes

added by Horstmann (1998a)

#### Pleurogyrus

Townes, 1970

#### Pleurogyrus persector

(Parfitt, 1882)

Hemiteles persector Parfitt, 1882

##### Distribution

England

##### Notes

distribution data from Fitton (1976)

#### Pleurogyrus pumilus

(Hellén, 1967)

Uchidella pumila Hellén, 1967

##### Distribution

England

##### Notes

added by Horstmann (1995)

#### Polyaulon

Förster, 1869

THAUMATOTYPUS Förster, 1869 synonymy by Horstmann (1998a)
THAUMATOTYPIDEA Viereck, 1912
RHACODOPTERON Čapek, 1956

#### Polyaulon paradoxus

(Zetterstedt, 1838)

Cryptus paradoxus Zetterstedt, 1838
billupsi (Bridgman, 1882, *Thaumatotypus*)
evertsi (Smits van Burgst, 1912, *Thaumatotypus*)

##### Distribution

England, Scotland, Wales

#### Polyaulon stiavnicensis

(Čapek, 1956)

Rhacodopteron stiavnicense Čapek, 1956

##### Distribution

England

##### Notes

added by Schwarz and Shaw (2000)

#### Pygocryptus

Roman, 1925

#### Pygocryptus brevicornis

(Brischke, 1881)

Macrocryptus brevicornis Brischke, 1881
grandis (Thomson, 1884, *Phygadeuon*) synonymy by Sawoniewicz (2003)

##### Distribution

England

##### Notes

added by Townes (1983)

#### Stibeutes

Förster, 1850

CHAMAEZELUS Förster, 1869
SCHIZOPLEURON Aubert, 1968

##### Notes

Some distribution data from Horstmann (1993c), Horstmann (2010b).

#### Stibeutes blandi

Schwarz & Shaw, 2011

##### Distribution

Scotland

##### Notes

added by Schwarz and Shaw (2011)

#### Stibeutes breviareolatus

(Thomson, 1884)

Hemiteles breviareolatus Thomson, 1884
rugiventris (Strobl, 1901, *Acanthocryptus*) synonymy by Horstmann (2000e)

##### Notes

Listed as a doubtfully placed species of *Hemiteles* by Fitton (1978).

#### Stibeutes brevicornis

(Lange, 1911)

Stilpnus brevicornis Lange, 1911

##### Distribution

England

##### Notes

added by Horstmann (2010b)

#### Stibeutes calderonae

Bordera & Hernández-Rodríguez, 2004

##### Distribution

England

##### Notes

added by Horstmann (2010b)

#### Stibeutes curvispina

(Thomson, 1884)

Phygadeuon curvispina Thomson, 1884

##### Distribution

England, Scotland, Wales

#### Stibeutes gravenhorstii

Förster, 1850

##### Distribution

England

#### Stibeutes heinemanni

Förster, 1850

##### Distribution

England, Scotland

#### Stibeutes heterogaster

(Thomson, 1885)

Phygadeuon heterogaster Thomson, 1885

##### Distribution

England, Scotland, Wales, Isle of Man

##### Notes

added by Horstmann (2010b)

#### Stibeutes intermedius

Horstmann, 2010

##### Distribution

England

##### Notes

added by Horstmann (2010b)

#### Stibeutes nigrinus

Horstmann, 2010

##### Distribution

England

##### Notes

added by Horstmann (2010b)

#### Stibeutes rozsypali

(Gregor, 1941)

Phygadeuon rozsypali Gregor, 1941

##### Distribution

England

##### Notes

added by Horstmann (2010b)

#### Stilpnus

Gravenhorst, 1829

##### Notes

Distribution data from Jussila (1987), Jussila (1999) and BMNH and NMS (det. Jussila).

#### Polyrhembia

Förster, 1869

#### Stilpnus (Polyrhembia) tenebricosus

(Gravenhorst, 1829)

Hemiteles tenebricosus Gravenhorst, 1829
nitidulator (Zetterstedt, 1838, *Ichneumon*)
vestalis (Haliday, 1839, *Atractodes*)
albicinctus (Förster, 1876, *Polyrhembia*)
anthracinus (Förster, 1876, *Polyrhembia*)
canaliculatus (Förster, 1876, *Polyrhembia*) preocc.
carbonarius (Förster, 1876, *Polyrhembia*)
corvinus (Förster, 1876, *Polyrhembia*)
discoloripes (Förster, 1876, *Atractodes*)
nigratus (Förster, 1876, *Polyrhembia*)
nigripes (Förster, 1876, *Polyrhembia*)
procerulus (Förster, 1876, *Polyrhembia*)
splendidus (Förster, 1876, *Polyrhembia*)
stygius (Förster, 1876, *Polyrhembia*)
rodnensis Kiss, 1924 synonymy by Horstmann (2011b)

##### Distribution

England, Scotland, Wales, Ireland

#### Stilpnus

Gravenhorst, 1829

#### Stilpnus (Stilpnus) blandus

Gravenhorst, 1829

assimilis Förster, 1876
callens Förster, 1876
cyclogaster Förster, 1876
fuscicornis Förster, 1876
pellucens Förster, 1876

##### Distribution

England, Scotland, Wales, Ireland

#### Stilpnus (Stilpnus) crassicornis

Thomson, 1884

##### Distribution

England, Scotland, Ireland

#### Stilpnus (Stilpnus) deplanatus

Gravenhorst, 1829

##### Distribution

England

##### Notes

distribution data from UM

#### Stilpnus (Stilpnus) gagates

(Gravenhorst, 1807)

Ichneumon gagates Gravenhorst, 1807
aequilongus Förster, 1876
cyclodes Förster, 1876
denticulatus Förster, 1876
diffinis Förster, 1876
dimidiatus Förster, 1876
elimatus Förster, 1876
eurygaster Förster, 1876
fulvicornis Förster, 1876
gallicus Förster, 1876
robinsoni Roman, 1920

##### Distribution

England, Scotland, Wales, Ireland

#### Stilpnus (Stilpnus) parvulus

Förster, 1876

inaequalis Förster, 1876

##### Distribution

England

##### Notes

added by Jussila (1987)

#### Stilpnus (Stilpnus) pavoniae

(Scopoli, 1763)

Ichneumon pavoniae Scopoli, 1763
agilis Förster, 1876
ambulatorius Förster, 1876
arridens Förster, 1876
conformatus Förster, 1876
declinis Förster, 1876
morionellus Förster, 1876
neglectus Förster, 1876
nigricoxis Förster, 1876
politus Förster, 1876 preocc.
retritus Förster, 1876
subtilis Förster, 1876
tersus Förster, 1876
trivialis Förster, 1876
unctus Förster, 1876
xanthopus Förster, 1876
angustatus Thomson, 1884

##### Distribution

England, Scotland, Wales, Ireland

#### Stilpnus (Stilpnus) subzonulus

Förster, 1876

canaliculatus Förster, 1876
diversus Förster, 1876
latens Förster, 1876
placitus Förster, 1876
tenuipes Thomson, 1884

##### Distribution

England, Scotland, Wales, Ireland

#### Xestophyes

Förster, 1869

XESTOPHYA Förster, 1876

#### Stilpnus (Xestophyes) dryadum

Curtis, 1832

##### Distribution

England, Ireland

##### Notes

Listed as a synonym of *Adelognathus dorsalis* in Yu and Horstmann (1997) but according to Jussila (1987), whilst a paralectotype is referable to *dorsalis*, the lectotype specimen is a species of *Stilpnus*.

#### Stilpnus (Xestophyes) fallax

(Förster, 1876)

Xestophya fallax Förster, 1876

##### Distribution

England

##### Notes

NMS, det. Jussila, added here

#### Sulcarius

Townes, 1970

##### Notes

Some distribution data from Townes (1983).

#### Sulcarius biannulatus

(Gravenhorst, 1829)

Hemiteles biannulatus Gravenhorst, 1829

##### Distribution

Scotland

#### Sulcarius bispinosus

(Rudow, 1886)

Phygadeuon bispinosus Rudow, 1886
hellbachi (Schmiedeknecht, 1905, *Hemiteles*)

##### Distribution

England

##### Notes

added by Townes (1983)

#### Sulcarius fontinalis

(Ruschka, 1926)

Hemiteles fontinalis Ruschka, 1926

##### Distribution

England, Scotland

##### Notes

added by Schwarz and Shaw (2011)

#### Sulcarius laevipleuris

Horstmann, 1992

##### Distribution

England

##### Notes

added by Horstmann (1992b)

#### Sulcarius nigricornis

(Thomson, 1884)

Hemiteles nigricornis Thomson, 1884
homocerus (Thomson, 1885, *Hemiteles*)

##### Distribution

England, Scotland, Wales, Ireland

##### Notes

Listed (as *homocerus*) as a doubtfully placed species of *Hemiteles* by Fitton (1978).

#### Sulcarius nigridens

Horstmann, 1992

##### Distribution

England, Scotland

##### Notes

added by Horstmann (1992b)

#### Sulcarius sp. A

##### Distribution

England, Scotland

##### Notes

added by Schwarz and Shaw (2011); to be described by the late K. Horstmann.

#### Thaumatogelis

Schwarz, 1995

THAUMATOGELIS Schmiedeknecht, 1933 unavailable
CRYPTOGELIS Hellén, 1944 nom. nud.

##### Notes

species of *Thaumatogelis* excluded from the British and Irish list by Schwarz (1995):

[*pilosus* (Capron, 1888, *Pezomachus*)] The type of *pilosus* was ostensibly British but Schwarz (1995) has excluded this southern European species from the British list.

#### Thaumatogelis audax

(Olivier, 1792)

Ichneumon audax Olivier, 1792
arnearum (Geoffroy, 1785, *Ichneumon*) preocc.
zonatus (Förster, 1850, *Pezomachus*)

##### Distribution

England, Wales

#### Thaumatogelis innoxius

Schwarz, 2001

mingetshauricus misident.

##### Distribution

England

##### Notes

Added by Schwarz (2001); tentative identification as *mingetshauricus* (Bogačev, 1946, *Gelis*) by Schwarz and Shaw (2000) was a misidentification.

#### Thaumatogelis lichtensteini

(Pfankuch, 1913)

Thaumatotypidea lichtensteini Pfankuch, 1913
cabrerai (Duchaussoy, 1915, *Thaumatotypidea*)
graecus (Rudow, 1917, *Pezomachus*)
siculus (Rudow, 1917, *Pezomachus*)
Thaumatogelis lichtensteini ?*maroccanus* (Ceballos, 1925, *Gelis*) synonymy by Schwarz (2001)
dentatus (Seyrig, 1926, *Thaumatotypidea*)
longicornis (Seyrig, 1926, *Thaumatotypidea*)
medianus (Seyrig, 1926, *Thaumatotypidea*)
micariae (Seyrig, 1926, *Thaumatotypidea*) preocc.
muticus (Seyrig, 1926, *Thaumatotypidea*)
nigripes (Seyrig, 1926, *Thaumatotypidea*)

##### Distribution

England

##### Notes

added by Schwarz and Shaw (2000)

#### Thaumatogelis neesii

(Förster, 1850)

Pezomachus neesii Förster, 1850
quadrifasciatus (Kriechbaumer, 1899, *Pezomachus*) unavailable

##### Distribution

England, Scotland

##### Notes

added by Schwarz and Shaw (2000)

#### Thaumatogelis sylvicola

(Förster, 1850)

Pezomachus sylvicola Förster, 1850
luceus (Seyrig, 1928, *Gelis*)

##### Distribution

England

#### Thaumatogelis vulpinus

(Gravenhorst, 1815)

Ichneumon vulpinus Gravenhorst, 1815
aquisgranensis (Förster, 1850, *Pezomachus*)

##### Distribution

England, Scotland, Wales

#### Theroscopus

Förster, 1850

CHAMERPES Förster, 1869
ERIPLANUS Förster, 1869
PHYRTUS Förster, 1869
THYSIOTORUS Förster, 1869

##### Notes

Some distribution data from Horstmann (1993c) and Fitton (1976).

#### Theroscopus bonelli

(Gravenhorst, 1815)

Ichneumon bonelli Gravenhorst, 1815
ingrediens Förster, 1850
rufiventris (Rudow, 1917, *Agrothereutes*)

##### Distribution

England, Wales

##### Notes

added by Horstmann (1993c)

#### Theroscopus coriaceus

Horstmann, 1993

##### Distribution

England

##### Notes

added by Horstmann (1993c)

#### Theroscopus esenbeckii

(Gravenhorst, 1815)

Ichneumon esenbeckii Gravenhorst, 1815
subzonatus (Gravenhorst, 1815, *Ichneumon*)
gravenhorstii (Ratzeburg, 1844, *Pezomachus*)
inaequalis Förster, 1850
transsylvanicus (Kiss, 1929, *Hemiteles*)

##### Distribution

England, Scotland, Wales, Ireland

##### Notes

*Theroscopus esenbeckii*, *subzonatus* and *inaequalis* are all listed as doubtfully placed species of *Hemiteles* by Fitton (1978).

#### Theroscopus fasciatulus

Horstmann, 1979

fasciatus (Thomson, 1884, *Hemiteles*) preocc.

##### Distribution

England

##### Notes

Listed as a doubtfully placed species of *Hemiteles* (as *fasciatus*) by Fitton (1978).

#### Theroscopus hemipteron

(Riche, 1791)

Ichneumon hemipteron Riche, 1791
hemipterus (Fabricius, 1793, *Ichneumon*)
hemipterator (Thunberg, 1824, *Ichneumon*)
dissimilis (Gravenhorst, 1829, *Hemiteles*)
scrupulosus (Gravenhorst, 1829, *Hemiteles*)
brevipennis (Brischke, 1891, *Thysiotorus*)
nanopterus (Kieffer, 1903, *Phygadeuon*)
insignipennis (Schmiedeknecht, 1905, *Hemiteles*)
kandaviensis (Ozols, 1934, *Hemiteles*)

##### Distribution

England, Scotland, Wales, Ireland

#### Theroscopus horsfieldi

Schwarz & Shaw, 2011

##### Distribution

Scotland

##### Notes

added by Schwarz and Shaw (2011)

#### Theroscopus mariae

Schwarz & Shaw, 2011

##### Distribution

England, Scotland

##### Notes

added by Schwarz and Shaw (2011)​

#### Theroscopus megacentrus

(Schiødte, 1839)

Cryptus megacentrus Schiødte, 1839
ornaticornis (Schmiedeknecht, 1897, *Hemiteles*) synonymy by Horstmann (2004a)
occisor (Habermehl, 1923, *Phygadeuon*) synonymy by Horstmann (2000c)

##### Distribution

England, Scotland

#### Theroscopus naninae

Schwarz & Shaw, 2011

##### Distribution

Scotland

##### Notes

added by Schwarz and Shaw (2011)​

#### Theroscopus ochrogaster

(Thomson, 1888)

Phygadeuon ochrogaster Thomson, 1888
rotundator Aubert, 1989 synonymy by Schwarz and Shaw (2011)​

##### Distribution

England, Scotland, Isle of Man

##### Notes

added by Schwarz and Shaw (2011)​

#### Theroscopus opacinotum

(Hellén, 1967)

Aclastus opacinotum Hellén, 1967

##### Notes

Added by Schwarz and Shaw (2011)​ and transferred from *Orthizema*.

#### Theroscopus pedestris

(Fabricius, 1775)

Ichneumon pedestris Fabricius, 1775
pilosellus (Rudow, 1917, *Pezomachus*)

#### Theroscopus pullator

(Gravenhorst, 1829)

Cryptus pullator Gravenhorst, 1829
notaulium (Morley, 1947, *Phygadeuon*)

##### Distribution

England, Scotland, Ireland

##### Notes

Listed as *Stiboscopus notaulius* (Morley) (with *pullator* as a doubtfully placed species of *Hemiteles*) by Fitton (1978); listed as *Orthizema pullator* by Yu and Horstmann (1997), transferred to *Theroscopus* by Schwarz and Shaw (2011)​.

#### Theroscopus rufulus

(Gmelin, 1790)

Ichneumon rufulus Gmelin, 1790
micator misident.
luteiventris (Gravenhorst, 1829, *Hemiteles*)
oxyphymus (Gravenhorst, 1829, *Hemiteles*)
rufulus (Gravenhorst, 1829, *Hemiteles*) preocc.
litoreus (Parfitt, 1882, *Hemiteles*)
politus (Bridgman, 1883, *Hemiteles*)
silesiacus (Habermehl, 1919, *Phygadeuon*)

##### Distribution

England, Scotland, Wales, Ireland

#### Theroscopus ungularis

(Thomson, 1884)

Phygadeuon ungularis Thomson, 1884
ungularis (Thomson, 1884, *Hemiteles*)
heteroneurus (Schmiedeknecht, 1933, *Hemiteles*)

##### Distribution

England

##### Notes

Listed as a doubtfully placed species of *Hemiteles* by Fitton (1978).

#### Tricholinum

Förster, 1869

STIBOSCOPELLUS Roman, 1930

#### Tricholinum ischnocerum

(Thomson, 1888)

Hemiteles ischnocerus Thomson, 1888
pimploides (Roman, 1930, *Stiboscopellus*)

##### Distribution

England, Scotland, Wales

##### Notes

added by Schwarz and Shaw (2011)​

#### Tropistes

Gravenhorst, 1829

PSEUDOLIMERODES Strobl, 1902
BOLESLAWIA Sawoniewicz, 1996 synonymy by Schwarz and Shaw (2011)​

#### Tropistes falcatus

(Thomson, 1884)

Hemiteles falcatus Thomson, 1884
nigriventris Kriechbaumer, 1894 preocc.
rufipes Kriechbaumer, 1894
compressiventris (Strobl, 1902, *Pseudolimerodes*)

##### Distribution

England

##### Notes

added by Schwarz and Shaw (2011)​

#### Tropistes nitidipennis

Gravenhorst, 1829

fuscipes Kriechbaumer, 1894 unavailable
nigriventris Kriechbaumer, 1894 unavailable

##### Distribution

England, Scotland

#### Tropistes scoticus

Schwarz & Shaw, 2011

##### Distribution

England, Scotland

##### Notes

added by Schwarz and Shaw (2011)​; English distribution from specimen in BMNH, det. Broad

#### Uchidella

Townes, 1957

ITAMUS Förster, 1869

#### Uchidella brevicauda

Horstmann, 1993

##### Distribution

England, Scotland

##### Notes

added by Horstmann (1993b)

#### Uchidella flavilabris

Horstmann, 1993

##### Distribution

England, Scotland

##### Notes

added by Schwarz and Shaw (2011)​

#### Uchidella longicaudata

Horstmann, 1997

longicauda Horstmann, 1993 preocc.

##### Distribution

England, Scotland, Ireland

##### Notes

added by Schwarz and Shaw (2011)​

#### Xenolytus

Förster, 1869

STERNOCRYPTUS Roman, 1925

##### Notes

Some distribution data from Townes (1983).

#### Xenolytus bitinctus

(Gmelin, 1790)

Ichneumon bitinctus Gmelin, 1790
expulsor (Thunberg, 1824, *Ichneumon*)

##### Distribution

England, Scotland

#### Xenolytus substriatus

Townes, 1983

##### Distribution

England

##### Notes

added by Townes (1983)

#### Xiphulcus

Townes, 1970

NOTOSTILBUS Townes, 1983

#### Xiphulcus floricolator

(Gravenhorst, 1807)

Ichneumon floricolator Gravenhorst, 1807
imbecillus (Gravenhorst, 1829, *Hemiteles*)
longiventris (Schiødte, 1839, *Hemiteles*) synonymy by Horstmann (2004a)
longulus (Thomson, 1884, *Hemiteles*)
lucidus (Szépligeti, 1901, *Phygadeuon*)
muelleri (Kiss, 1924, *Hemiteles*)
ramellosus (Kiss, 1924, *Leptocryptus*)
longicauda Hellén, 1967, Uchidella)

##### Distribution

England, Scotland

##### Notes

Recorded by Kerrich (1935); English record from A.C. Galsworthy coll.

#### Zoophthorus

Förster, 1869

CHAETOMASTRUS Hellén, 1967

##### Notes

See note for *Mastrus*. Some distribution data from Fitton (1976).

#### Zoophthorus anglicanus

(Morley, 1907)

Hemiteles anglicanus Morley, 1907

##### Distribution

England, Isle of Man

#### Zoophthorus bridgmani

(Schmiedeknecht, 1897)

Hemiteles bridgmani Schmiedeknecht, 1897
niger (Bridgman, 1883, *Theroscopus*) invalid
pfankuchi (Smits van Burgst, 1913, *Hemiteles*)

##### Distribution

England, Scotland

#### Zoophthorus cynipinus

(Thomson, 1884)

Hemiteles cynipinus Thomson, 1884

##### Distribution

England, Scotland

#### Zoophthorus dodecellae

(Obrtel & Šedivý, 1960)

Hemiteles dodecellae Obrtel & Šedivý, 1960
added by

##### Distribution

England, Scotland

##### Notes

added by Schwarz and Shaw (2010)

#### Zoophthorus graculus

(Gravenhorst, 1829)

Bassus graculus Gravenhorst, 1829
auriculatus (Thomson, 1884, *Hemiteles*)
albomarginatus (Bridgman, 1887, *Hemiteles*)

##### Distribution

England, Isle of Man

#### Zoophthorus infirmus

(Gravenhorst, 1829)

Hemiteles infirmus Gravenhorst, 1829
tenerrimus (Gravenhorst, 1829, *Hemiteles*)

##### Notes

Transferred from *Eudelus* by Schwarz and Shaw (2000).

#### Zoophthorus notaticrus

(Thomson, 1888)

Hemiteles notaticrus Thomson, 1888

##### Distribution

England, Scotland

##### Notes

added by Schwarz and Shaw (2010)

#### Zoophthorus palpator

(Müller, 1776)

Ichneumon palpator Müller, 1776
incisus (Bridgman, 1883, *Hemiteles*)
hilarellus (Schmiedeknecht, 1905, *Hemiteles*)

##### Distribution

England, Scotland, Wales, Isle of Man

#### Zoophthorus plumbeus

(Thomson, 1884)

Hemiteles plumbeus Thomson, 1884

##### Distribution

England

##### Notes

added by Schwarz and Shaw (2010)

#### Zoophthorus sp. A

##### Distribution

England

##### Notes

added by Schwarz and Shaw (2010); to be described by the late K. Horstmann (in prep.)

### Ctenopelmatinae

#### CTENOPELMATINAE

Förster, 1869

SCOLOBATINAE Schmiedeknecht, 1911

##### Notes

Parts of the NHM collection have relatively recently been determined by J. Barron, R. Hinz, K. Horstmann, M. Idar and J.-F. Aubert, parts of the NMS have been determined by D.R. Kasparyan. the catalogue of Aubert (2000) frequently has different dates of publication and endings of specific names to those in Yu and Horstmann (1997), the latter is followed. Unless stated otherwise, distribution data from Aubert (2000), Fitton (1976), for type localities, and the collections of NHM, NMS and UM.

#### CHRIONOTINI

Uchida, 1957

OLETHRODOTINI Townes, 1970

##### Notes

Distribution data from Shaw and Kasparyan (2002). Note that Olethrodotini has generally been used as the name for this small tribe but Chrionotini has precedence.

#### Olethrodotis

Förster, 1869

TASCHENBERGIA Schmiedeknecht, 1888

#### Olethrodotis modestus

(Gravenhorst, 1829)

Mesoleptus modestus Gravenhorst, 1829
evolans (Gravenhorst, 1829, *Tryphon*)
microtamia (Gravenhorst, 1829, *Phytodietus*)

##### Distribution

England, Scotland

#### CTENOPELMATINI

Förster, 1869

##### Notes

The checklist of British Ctenopelmatini was revised by Shaw et al. (2003), including nomenclatural changes and distribution data.

#### Ctenopelma

Holmgren, 1857

DIEDRUS Förster, 1869
ERYMA Förster, 1869
XANIOPELMA Tschek, 1869
ZACHRESTA Förster, 1869
HOLMGRENIA Kriechbaumer, 1877
KRIECHBAUMERIA Dalla Torre, 1885
POLYOMORUS Kriechbaumer, 1894
POLYHOMORUS Schulz, 1906
PSEUDOBANCHUS Szépligeti, 1911

#### Ctenopelma ruficorne

Holmgren, 1857

Ctenopelma ruficornis Holmgren, 1857

##### Distribution

Scotland

##### Notes

added by Shaw et al. (2003)

#### Ctenopelma tomentosum

(Desvignes, 1856)

Campoplex tomentosus Desvignes, 1856
nigrum misident.
lucifer misident.
luteum Holmgren, 1857
xanthostigma Holmgren, 1857; synonymy by Kasparyan (2004a)
variabile Tschek, 1869
gagatinum (Kriechbaumer, 1894, *Polyomorus*)
athimi Kriechbaumer, 1896; synonymy by Kasparyan (2004a)
pulchrum (Kriechbaumer, 1877, *Holmgrenia*)
braunsii Pfankuch, 1904
dispar Ulbricht, 1916

##### Distribution

England

##### Notes

Although Aubert (2000) placed *tomentosum* in synonymy with *luteum*, Holmgren’s publication actually dates from 1857, not 1855, so *tomentosum* is the valid name (Shaw et al. 2003).

#### Homaspis

Förster, 1869

NEOHOMASPSIS Heinrich, 1949

#### Homaspis analis

(Holmgren, 1857)

Notopygus analis Holmgren, 1857
subalpina misident.
defectivus (Tschek, 1869, *Ctenopelma*); synonymy by Kasparyan (2004a)
pectator Aubert, 1989; synonymy by Kasparyan (2004a)

##### Distribution

England

##### Notes

Listed as a synonym of *H. narrator* (Gravenhorst, 1829, *Mesoleptus*) by Yu and Horstmann (1997) but this was not accepted by Shaw et al. (2003).

#### Notopygus

Holmgren, 1857

ANTIPYGUS Tschek, 1869

#### Notopygus emarginatus

Holmgren, 1857

sinifer Ulbricht, 1922

##### Distribution

England, Scotland

#### Xenoschesis

Förster, 1869

#### Xenoschesis

Förster, 1869

HOMOBIA Förster, 1869
GLYPTOCENTRUS Kriechbaumer, 1894

#### Xenoschesis (Xenoschesis) fulvipes

(Gravenhorst, 1829)

Exetastes fulvipes Gravenhorst, 1829
ruficornis (Rudow, 1883, *Exetastes*)
ruficornis (Rudow, 1886, *Exetastes*)
varicoxa Heinrich, 1949; synonymy by Aubert (2000)

##### Distribution

England, Scotland

#### Polycinetis

Förster, 1869

ERIGLOEA Förster, 1869
PROSMORUS Förster, 1869
POLYCINETUS Thomson, 1893

#### Xenoschesis (Polycinetis) ustulata

(Desvignes, 1856)

Tryphon ustulatus Desvignes, 1856
resplendens (Holmgren, 1857, *Notopygus*); synonymy by Shaw et al. (2003)
polita (Kriechbaumer, 1891, *Erigloea*)
montana (Habermehl, 1922, *Hadrodactylus*)

##### Distribution

England, Scotland

##### Notes

Horstmann (2006b) removed *fulvicornis* (Kriechbeumer, 1891, *Erigloea*) from synonymy.

#### EURYPROCTINI

Thomson, 1883

#### Anisotacrus

Schmiedeknecht, 1913

#### Anisotacrus bipunctatus

(Gravenhorst, 1829)

Mesoleptus bipunctatus Gravenhorst, 1829

##### Distribution

England

#### Anisotacrus tenellus

(Holmgren, 1857)

Mesoleius tenellus Holmgren, 1857

##### Distribution

England, Scotland

##### Notes

BMNH, NMS added here; treated as a synonym of *bipunctatus* by Aubert (2000) but as a valid species in Yu and Horstmann (1997).

#### Anisotacrus xanthostigma

(Gravenhorst, 1829)

Mesoleptus xanthostigma Gravenhorst, 1829
vividus (Woldstedt, 1874, *Mesoleptus*)

##### Distribution

England

#### Euryproctus

Holmgren, 1857

HYPOCRYPTUS Förster, 1869
SYCHNOLETER Förster, 1869
XENONASTES Förster, 1869

#### Euryproctus alpinus

Holmgren, 1857

exareolatus Thomson, 1889

##### Distribution

England

#### Euryproctus annulatus

(Gravenhorst, 1829)

Mesoleptus annulatus Gravenhorst, 1829
annulator (Stephens, 1835, *Mesoleptus*)

##### Distribution

England, Scotland

#### Euryproctus bivinctus

Holmgren, 1857

##### Distribution

England, Wales, Scotland

##### Notes

added by Aubert (2000)

#### Euryproctus crassicornis

Thomson, 1889

##### Distribution

England

#### Euryproctus geniculosus

(Gravenhorst, 1829)

Mesoleptus geniculosus Gravenhorst, 1829

##### Distribution

England, Scotland, Wales, Isle of Man

#### Euryproctus holmgreni

Kerrich, 1942

##### Distribution

England

#### Euryproctus inferus

Thomson, 1889

##### Distribution

England, Scotland

#### Euryproctus luteicornis

(Gravenhorst, 1829)

Tryphon luteicornis Gravenhorst, 1829

##### Distribution

England

##### Notes

added by Aubert (2000)

#### Euryproctus mundus

(Gravenhorst, 1820)

Ichneumon mundus Gravenhorst, 1820
aberrans Woldstedt, 1877
testaceicornis (Brischke, 1892, *Mesoleptus*)
strandi (Gregor, 1937, *Mesoleptus*); synonymy by Horstmann (2002b)

##### Distribution

England, Scotland

#### Euryproctus nemoralis

(Geoffroy, 1785)

Ichneumon nemoralis Geoffroy, 1785
digitator (Thunberg, 1824, *Ichneumon*)
Euryproctus nemoralis ?*suborbitalis* (Stephens, 1835, *Mesoleptus*)
affinis (Holmgren, 1856, *Mesoleptus*)
vafer Woldstedt, 1874
foersteri Kriechbaumer, 1897; synonymy by Horstmann (2002c)

##### Distribution

England, Scotland, Isle of Man

#### Euryproctus plantator

(Thunberg, 1824)

Ichneumon plantator Thunberg, 1824
albipes Holmgren, 1857
tuberculatus Holmgren, 1857
exareolatus Thomson, 1889

##### Distribution

England

##### Notes

added by Aubert (2000)

#### Euryproctus ratzeburgi

(Gorski, 1852)

Tryphon ratzeburgi Gorski, 1852
sinister Brischke, 1871; synonymy by Horstmann (1998b)
nitidulus Thomson, 1889
phygadeuontoides (Kriechbaumer, 1896, *Polyblastus*)
pictus Habermehl, 1925

##### Distribution

England

#### Gunomeria

Schmiedeknecht, 1907

#### Gunomeria macrodactylus

(Holmgren, 1856)

Mesoleptus macrodactylus Holmgren, 1856
scutellata (Bridgman, 1886, *Mesoleptus*)

##### Distribution

England, Scotland, Wales, Ireland

##### Notes

Treated as a synonym of *sordida* in Yu and Horstmann (1997), as a separate species by Aubert (2000) and then by Horstmann (2008a).

#### Gunomeria sordida

(Gravenhorst, 1829)

Mesoleptus sordidus Gravenhorst, 1829

##### Distribution

England, Scotland, Wales, Isle of Man

#### Hadrodactylus

Förster, 1869

DIZEMON Förster, 1869
NARCOPOEA Förster, 1869
ZEMIODES Förster, 1869
MEROPACHES Schmiedeknecht, 1913

##### Notes

Distribution data from Idar (1974), Idar (1975), Idar (1981), Aubert (2000), Kasparyan and Shaw (2009) and BMNH.

Species of *Hadrodactylus* excluded from the British and Irish list

[*bidentulus* Thomson, 1883] Kasparyan and Shaw (2009) could not find any British or Irish specimens; those in BMNH under *bidentulus* were misidentified.

[*larvatus* Kriechbaumer, 1891] Erroneously listed as occurring in the British Isles by Kasparyan (2011).

#### Hadrodactylus confusus

(Holmgren, 1858)

Mesoleptus confusus Holmgren, 1858
albicoxa Thomson, 1883

##### Distribution

England

##### Notes

Listed, presumably erroneously, as occurring in Scotland by Kasparyan (2011).

#### Hadrodactylus faciator

(Thunberg, 1824)

Ichneumon faciator Thunberg, 1824
gracilis (Holmgren, 1856, *Mesoleptus*)
curtus (Holmgren, 1857, *Mesoleptus*)

##### Distribution

England, Scotland, Isle of Man

#### Hadrodactylus femoralis

(Holmgren, 1857)

Mesoleptus femoralis Holmgren, 1857
intrepidus Kriechbaumer, 1891; synonymy by Horstmann (2000c)
nigricoxa (Thomson, 1893, *Mesoleptus*)
thomsoni Schmiedeknecht, 1913

##### Distribution

England, Scotland, Ireland

##### Notes

Added by Idar (1975); omitted by Fitton (1978).

#### Hadrodactylus flavofacialis

Horstmann, 2000

flavifrontator misident.

##### Distribution

England, Scotland

##### Notes

Added by Idar (1981), some distribution data from Horstmann (2000b) and UM; listed as *flavifrontator* (Thunberg, 1824, *Ichneumon*) in Aubert (2000) (Horstmann 2000b).

#### Hadrodactylus fugax

(Gravenhorst, 1829)

Mesoleptus fugax Gravenhorst, 1829
ventralis (Curtis, 1837, *Mesoleptus*)
marginatus (Bridgman, 1886, *Mesoleptus*)
alticola (Strobl, 1903, *Mesoleptus*)
branderi Jussila, 1967

##### Distribution

England, Scotland, Wales, Ireland

#### Hadrodactylus genalis

Thomson, 1883

pygmaeus Habermehl

##### Distribution

England

##### Notes

NHM, det. Broad, added here

#### Hadrodactylus gracilipes

Thomson, 1883

meridionator Villemant, 1982; synonymy by Kasparyan (2011)

##### Distribution

England, Scotland

#### Hadrodactylus gracilis

(Stephens, 1835)

Mesoleptus gracilis Stephens, 1835

##### Distribution

England

#### Hadrodactylus graminicola

Idar, 1979

##### Distribution

England, Scotland

##### Notes

added by Kasparyan and Shaw (2009)

#### Hadrodactylus idari

Kasparyan & Shaw, 2009

gracilipes misident.

##### Distribution

England, Scotland

##### Notes

added by Kasparyan and Shaw (2009)

#### Hadrodactylus indefessus

(Gravenhorst, 1820)

Ichneumon indefessus Gravenhorst, 1820
tarsator Thomson, 1883

##### Distribution

England, Scotland, Ireland

##### Notes

added by Kasparyan and Shaw (2009)

#### Hadrodactylus insignis

Kriechbaumer, 1891

varicoxa (Thomson, 1893, *Mesoleptus*)
variicoxa Dalla Torre, 1901

##### Distribution

England, Scotland

#### Hadrodactylus nigrifemur

Thomson, 1883

##### Distribution

England, Scotland, Wales, Ireland

#### Hadrodactylus paludicola

(Holmgren, 1856)

Mesoleptus paludicola Holmgren, 1856
subalpinus Schmiedeknecht, 1913

##### Distribution

England, Scotland, Ireland, Isle of Man

#### Hadrodactylus semirufus

(Holmgren, 1858)

Mesoleptus semirufus Holmgren, 1858
erythropus Kriechbaumer, 1891; synonymy by Horstmann (2000c)
pubescens Ulbricht, 1922

##### Distribution

England, Scotland, Wales

##### Notes

added by Aubert (2000)

#### Hadrodactylus spiraculator

Idar, 1979

##### Distribution

England, Scotland

##### Notes

added by Idar (1981)

#### Hadrodactylus tiphae

(Geoffroy, 1785)

Ichneumon tiphae Geoffroy, 1785
luteolus (Gmelin, 1790, *Ichneumon*)
laticeps Thomson, 1883
erythropus Kriechbaumer, 1891

##### Distribution

England, Scotland, Ireland

#### Hadrodactylus villosulus

Thomson, 1883

##### Distribution

England, Isle of Man

#### Hadrodactylus vulneratus

(Zetterstedt, 1838)

Tryphon vulneratus Zetterstedt, 1838

##### Distribution

England, Scotland

##### Notes

added by Aubert (2000)

#### Hypamblys

Förster, 1869

APYSTUS Förster, 1869
LATHROPHAGUS Förster, 1869

#### Hypamblys albopictus

(Gravenhorst, 1829)

Tryphon albopictus Gravenhorst, 1829
transfuga (Holmgren, 1857, *Mesoleius*)
instabilis (Ruthe, 1859, *Tryphon*)

##### Distribution

England, Scotland

#### Hypsantyx

Pfankuch, 1906

#### Hypsantyx lituratorius

(Linnaeus, 1761)

Ichneumon lituratorius Linnaeus, 1761
tenthredinum (Scharfenberg, 1805, *Ichneumon*)
impressus (Gravenhorst, 1829, *Tryphon*)
crassicornis (Zetterstedt, 1838, *Pimpla*)

#### Mesoleptidea

Viereck, 1912

GNATHONOPHORUS Schmiedeknecht, 1912

#### Mesoleptidea cingulata

(Gravenhorst, 1829)

Mesoleptus cingulatus Gravenhorst, 1829
bidens (Fabricius, 1798, *Ichneumon*) nom. ob.: Horstmann (2001c)
bidentor (Thunberg, 1824, *Ichneumon*) nom. ob.: Horstmann (2006b)
pectoralis (Gravenhorst, 1829, *Mesoleptus*)
submarginata (Stephens, 1835, *Mesoleptus*)
undecimnotata (Desvignes, 1856, *Mesoleptus*)

##### Distribution

England, Scotland

#### Mesoleptidea hilaris

(Gravenhorst, 1829)

Mesoleptus hilaris Gravenhorst, 1829

##### Distribution

England

#### Mesoleptidea prosoleuca

(Gravenhorst, 1820)

Ichneumon prosoleucus Gravenhorst, 1820
tricolor (Fabricius, 1793, *Ophion*) nom. ob.: Horstmann (2001c)
neglecta (Holmgren, 1857, *Mesoleptus*)
glacialis (Woldstedt, 1874, *Mesoleptus*)
similis (Brischke, 1878, *Mesoleptus*)
holmgreni (Thomson, 1893, *Mesoleptus*)
nigriventris (Habermehl, 1925, *Mesoleptus*)

##### Distribution

England, Scotland, Ireland

#### Mesoleptidea stallii

(Holmgren, 1858)

Mesoleptus stallii Holmgren, 1858

#### Occapes

Townes, 1970

#### Occapes selandriae

(Brischke, 1878)

Polyblastus selandriae Brischke, 1878

##### Distribution

England, Wales

##### Notes

BMNH, det. Broad, added here

#### Pantorhaestes

Förster, 1869

TROPHOCTONUS Förster, 1869

#### Pantorhaestes xanthostomus

(Gravenhorst, 1829)

Tryphon xanthostomus Gravenhorst, 1829
ochrostomus (Gravenhorst, 1829, *Tryphon*)
rufocinctus (Gravenhorst, 1829, *Mesoleptus*)
hilarellus (Holmgren, 1858, *Euryproctus*)
intensicolor (Heinrich, 1953, *Dialges*)

##### Distribution

England, Scotland, Wales, Isle of Man

#### Phobetes

Förster, 1869

IPOCTONUS Förster, 1869
PHILOTYMMA Förster, 1869
PHOBETUS Thomson, 1889
GRIPHODES Kriechbaumer, 1894
IPOCTONINUS Hincks, 1944
PHOBETELLUS Hincks, 1944

#### Phobetes atomator

(Müller, 1776)

Ichneumon atomator Müller, 1776
croatica (Kiss, 1926, *Brischkea*); synonymy by Horstmann (2007b)

##### Distribution

England, Wales, Isle of Man

#### Phobetes cerinostomus

(Gravenhorst, 1829)

Mesoleptus cerinostomus Gravenhorst, 1829

##### Distribution

England

##### Notes

NMS, det. Kasparyan, added here

#### Phobetes chrysostomus

(Gravenhorst, 1820)

Ichneumon chrysostomus Gravenhorst, 1820

##### Distribution

England

#### Phobetes femorator

(Thomson, 1893)

Phobetus femorator Thomson, 1893
subalpinus (Strobl, 1903, *Euryproctus*)

##### Distribution

England

#### Phobetes fuscicornis

(Holmgren, 1856)

Tryphon fuscicornis Holmgren, 1856
fulviventris (Thomson, 1893, *Phobetus*)

#### Phobetes leptocerus

(Gravenhorst, 1820)

Ichneumon leptocerus Gravenhorst, 1820
stigmaticus (Brischke, 1878, *Mesoleptus*)
schmiedeknechti (Lange, 1911, *Hadrodactylus*)
aigneri (Kiss, 1926, *Brischkea*); synonymy by Horstmann (2007b)

##### Distribution

England, Scotland, Wales

#### Phobetes liopleuris

(Thomson, 1889)

Euryproctus liopleuris Thomson, 1889

##### Distribution

England

##### Notes

Added by Aubert (2000); treated as a separate species by Aubert (2000), as a synonym of *leptocerus* in Yu and Horstmann (1997).

#### Phobetes nigriceps

(Gravenhorst, 1829)

Tryphon nigriceps Gravenhorst, 1829
praetermissus (Woldstedt, 1874, *Mesoleptus*)

##### Distribution

England, Scotland

##### Notes

*Eclytus transsylvanicus* Kiss, 1924 removed from synonymy with *nigriceps* and placed in synonymy with *Phobetes latipes* (Thomson, 1895, *Phobetus*) by Horstmann (2007b); *latipes* and *nigriceps* were differentiated by Horstmann (2007b).

#### Syndipnus

Förster, 1869

POLYPYSTIS Förster, 1869
TLEMON Förster, 1869
DICKSONIA Holmgren, 1880
NEASTUS Holmgren, 1883

#### Syndipnus alutaceus

(Holmgren, 1857)

Trematopygus alutaceus Holmgren, 1857
alutaceus (Woldstedt, 1874, *Mesoleius*)

##### Distribution

Scotland

##### Notes

added by Aubert (2000)

#### Syndipnus decipiens

(Woldstedt, 1877)

Mesoleius decipiens Woldstedt, 1877
subscaber Thomson, 1893

##### Distribution

England

##### Notes

NMS, added here

#### Syndipnus lateralis

(Gravenhorst, 1829)

Tryphon lateralis Gravenhorst, 1829
punctiscuta Thomson, 1894

##### Distribution

England, Scotland

#### Syndipnus macrocerus

(Thomson, 1883)

Euryproctus macrocerus Thomson, 1883

##### Distribution

England

##### Notes

NMS, added here

#### Synodites

Förster, 1869

CAMPONASTES Förster, 1869
LISTROTA Förster, 1869
POLYTERUS Förster, 1869
SARCORYCHUS Förster, 1869
SYCHNOPORTUS Förster, 1869
ZOOTREPHES Förster, 1869
ZOOTREPHUS Thomson, 1890
SYNODYTES Thomson, 1893
ANAGLYMMUS Roman, 1914

##### Notes

species of *Synodites* excluded from the British and Irish list:

[*breviventris* (Gravenhorst, 1829, *Hemiteles*)] Listed as a species of *Dichrogaster* (Cryptinae: Phygadeuontini) in Fitton (1978), there are no identified specimens in BMNH and Aubert (2000) does not list it as occurring in Britain or Ireland.

#### Synodites breviusculus

(Fonscolombe, 1849)

Tryphon breviusculus Fonscolombe, 1849
delicatus (Fonscolombe, 1849, *Tryphon*)
buccatus (Holmgren, 1857, *Mesoleius*)

##### Distribution

England

##### Notes

Placed in *Hypamblys* in Yu and Horstmann (1997), in *Synodites* by Aubert (2000).

#### Synodites carinatus

(Holmgren, 1857)

Mesoleius carinatus Holmgren, 1857

##### Distribution

England

##### Notes

added by Aubert (2000)

#### Synodites erosus

(Holmgren, 1857)

Trematopygus erosus Holmgren, 1857

##### Distribution

Scotland

##### Notes

Added by Aubert (2000); one specimen in BMNH from 'Wissant', which could not be located in a Gazetteer. ‘England’ is given in Aubert (2000) but all other BMNH specimens are from Scotland.

#### Synodites facialis

(Thomson, 1893)

Spudaeus facialis Thomson, 1893

##### Distribution

England

##### Notes

In Yu and Horstmann (1997) as a synonym of *discolor* (Holmgren, 1857, *Trematopygus*) (placed in *Syndipnus* in Aubert 2000); the two species were differentiated by Horstmann (2011b).

#### Synodites hilaris

(Woldstedt, 1880)

Bassus hilaris Woldstedt, 1880

##### Distribution

Scotland

##### Notes

NMS, added here

#### Synodites lineiger

(Thomson, 1893)

Syndipnus lineiger Thomson, 1893

##### Distribution

England

##### Notes

added by Aubert (2000)

#### Synodites notatus

(Gravenhorst, 1829)

Tryphon notatus Gravenhorst, 1829
bimaculatus (Desvignes, 1856, *Tryphon*)
assimilis (Holmgren, 1858, *Tryphon*)
aberrans (Brischke, 1871, *Polyblastus*)
hungaricus (Kiss, 1924, *Ipoctonus*); synonymy by Horstmann (2007b)

##### Distribution

England, Scotland, Wales

#### Synodites parviceps

(Thomson, 1894)

Syndipnus parviceps Thomson, 1894

##### Distribution

Scotland

##### Notes

NMS, det. Kasparyan, added here

#### Synomelix

Förster, 1869

##### Notes

Distribution data from Idar (1983).

#### Synomelix albipes

(Gravenhorst, 1829)

Tryphon albipes Gravenhorst, 1829
sieboldii Kriechbaumer, 1897
kriechbaumeri Schmiedeknecht, 1913

##### Distribution

England, Scotland

#### Synomelix faciator

Idar, 1983

##### Distribution

England, Scotland

##### Notes

added by Idar (1983)

#### Synomelix perfida

(Woldstedt, 1874)

Tryphon perfidus Woldstedt, 1874
curvula (Thomson, 1895, *Syndipnus*)

##### Distribution

Scotland

#### Zemiophora

Förster, 1869

ZEMIOPHORUS Thomson, 1893

#### Zemiophora scutulata

(Hartig, 1838)

Tryphon scutulatus Hartig, 1838
brischkei (Holmgren, 1871, *Mesoleius*)
nobilis (Habermehl, 1909, *Otlophorus*)

##### Distribution

England

#### MESOLEIINI

Thomson, 1883

#### Alexeter

Förster, 1869

ADRANES Förster, 1869
ZEMIOPHRON Förster, 1869

##### Notes

Distribution data from Gauld and Mitchell (1977a), Aubert (2000) and BMNH.

Species of *Alexeter* excluded from the British and Irish list:

[*attenuatus* (Bridgman, 1887, *Mesoleius*)] will be synonymised by Broad & Rose (in prep.)

#### Alexeter clavator

(Müller, 1776)

Ichneumon clavator Müller, 1776
testaceator misident.
testaceus misident.
venosus (Gmelin, 1790, *Ichneumon*)

##### Distribution

England

#### Alexeter coxalis

(Brischke, 1871)

Mesoleptus coxalis Brischke, 1871
inconspicuus Schiedeknecht, 1914
rufus Kiss, 1926

##### Distribution

Scotland, Ireland

##### Notes

BMNH, det. Aubert and Broad, added here

#### Alexeter fallax

(Holmgren, 1857)

Mesoleius fallax Holmgren, 1857

##### Distribution

England, Scotland

#### Alexeter multicolor

(Gravenhorst, 1829)

Tryphon multicolor Gravenhorst, 1829
dives (Holmgren, 1857, *Mesoleius*)
napaeus (Holmgren, 1857, *Mesoleius*)

##### Distribution

England, Scotland, Wales, Isle of Man

#### Alexeter nebulator

(Thunberg, 1824)

Ichneumon nebulator Thunberg, 1824
melanocephalus (Gravenhorst, 1829, *Mesoleptus*)
gracilipes (Curtis, 1837, *Mesoleptus*)
paludicola Habermehl, 1922

##### Distribution

England, Scotland

#### Alexeter niger

(Gravenhorst, 1829)

Tryphon niger Gravenhorst, 1829

##### Distribution

England, Ireland

#### Alexeter rapinator

(Gravenhorst, 1829)

Tryphon rapinator Gravenhorst, 1829
laevissimus (Strobl, 1903, *Mesoleius*)

##### Distribution

England

#### Alexeter segmentarius

(Fabricius, 1787)

Ichneumon segmentarius Fabricius, 1787
sectator (Thunberg, 1824, *Ichneumon*); synonymy by Horstmann (2001c)
fraternarius (Thunberg, 1824, *Ichneumon*)
maxillarius (Thunberg, 1824, *Ichneumon*)
ruficornis (Gravenhorst, 1829, *Mesoleptus*)
lugubris (Woldstedt, 1874, *Mesoleptus*)
sibiricus Kiss, 1926
rufopetiolaris Kiss, 1933

##### Distribution

England, Scotland

#### Anoncus

Townes, 1970

##### Notes

Distribution data from Shaw and Kasparyan (2003).

Species of *Anoncus* excluded from the British and Irish list:

[*linitus* (Holmgren, 1857, *Mesoleius*)] Apparently only recorded as British by Carr (1924) and thus should have been deleted from the British list (see note under *Lissonota funebris*)

#### Anoncus femorator

(Thomson, 1893)

Mesoleius femorator Thomson, 1893

##### Distribution

Scotland

##### Notes

added by Shaw and Kasparyan (2003)

#### Anoncus gracilicornis

(Holmgren, 1857)

Mesoleius gracilicornis Holmgren, 1857

##### Distribution

England

#### Arbelus

Townes, 1970

#### Arbelus athaliaeperda

(Curtis, 1860)

Bassus athaliaeperda Curtis, 1860
athaliiperdus (Marshall, 1872, *Bassus*)

##### Distribution

England, Ireland

#### Azelus

Förster, 1869

##### Notes

Distribution data from Shaw and Kasparyan (2003).

#### Azelus erythropalpus

(Gmelin, 1790)

Ichneumon erythropalpus Gmelin, 1790
laterator (Thunberg, 1824, *Ichneumon*)
triangulatus (Bridgman, 1886, *Perilissus*)
bipunctatus (Szépligeti, 1901, *Mesoleius*)
csikii (Kiss, 1926, *Barytarbes*); synonymy by Horstmann (2007b)

##### Distribution

England, Wales, Scotland

#### Barytarbes

Förster, 1869

HYBRISTES Förster, 1869
ISODIAETA Förster, 1869
POLYTRERA Förster, 1869
BARYTARBUS Thomson, 1883
POLYTRERES Thomson, 1892
APHOLIUM Townes, 1970; synonymy by Aubert (2000)

#### Barytarbes colon

(Gravenhorst, 1829)

Tryphon colon Gravenhorst, 1829
ventosus (Holmgren, 1876, *Mesoleius*)

##### Distribution

England

#### Barytarbes flavicornis

(Thomson, 1892)

Mesoleius flavicornis Thomson, 1892
segmentarius (Perkins, 1962, *Isodiaeta*); unavailable (Horstmann 2004b, Horstmann 2005a)

##### Distribution

England

##### Notes

This species has often been referred to as *Barytarbes segmentarius* (Fabricius, 1787) *sensu* Gravenhorst, but that taxon is actually a species of *Alexeter* (Horstmann 2001c).

#### Barytarbes flavoscutellatus

(Thomson, 1892)

Mesoleius flavoscutellatus Thomson, 1892

##### Distribution

England

#### Barytarbes laeviusculus

(Thomson, 1883)

Mesoleius laeviusculus Thomson, 1883

##### Distribution

England

#### Campodorus

Förster, 1869

PHAGESORUS Förster, 1869
CUBOSCOPESIS Heinrich, 1952

##### Notes

Distribution data for species of *Mesoleius* and *Campodorus*, unless stated otherwise, are taken from Shaw and Kasparyan (2003).

#### Campodorus alticola

(Holmgren, 1857)

Mesoleius alticola Holmgren, 1857

##### Distribution

Scotland

##### Notes

added by Shaw and Kasparyan (2003)

#### Campodorus amictus

(Holmgren, 1857)

Mesoleius amictus Holmgren, 1857

##### Distribution

England, Scotland

#### Campodorus astutus

(Holmgren, 1876)

Mesoleius astutus Holmgren, 1876

##### Distribution

England, Scotland

#### Campodorus caligatus

(Gravenhorst, 1829)

Tryphon caligatus Gravenhorst, 1829
nemati (Ratzeburg, 1852, *Tryphon*)

##### Distribution

England, Scotland

#### Campodorus ciliatus

(Holmgren, 1857)

Mesoleius ciliatus Holmgren, 1857

##### Distribution

Scotland

##### Notes

added by Shaw and Kasparyan (2003)

#### Campodorus commotus

(Holmgren, 1876)

Mesoleius commotus Holmgren, 1876
perturbatus (Holmgren, 1876, *Mesoleius*)

##### Distribution

Scotland

##### Notes

added by Shaw and Kasparyan (2003)

#### Campodorus corrugatus

(Holmgren, 1876)

Mesoleius corrugatus Holmgren, 1876

#### Campodorus difformis

(Holmgren, 1876)

Mesoleius difformis Holmgren, 1876

##### Distribution

England, Scotland, Wales

##### Notes

added by Shaw and Kasparyan (2003)

#### Campodorus dorsalis

(Gravenhorst, 1829)

Tryphon dorsalis Gravenhorst, 1829

##### Distribution

England, Scotland, Wales, Isle of Man

#### Campodorus efferus

(Holmgren, 1876)

Mesoleius efferus Holmgren, 1876

##### Distribution

England, Scotland

##### Notes

added by Shaw and Kasparyan (2003)

#### Campodorus elegans

(Parfitt, 1882)

Mesoleius elegans Parfitt, 1882

##### Distribution

England

##### Notes

Transferred from *Mesoleius* by Shaw and Kasparyan (2003).

#### Campodorus flavescens

Kasparyan, 2003

##### Distribution

Scotland

##### Notes

NMS, det. Kasparyan, added here

#### Campodorus gallicus

(Thomson, 1893)

Mesoleius gallicus Thomson, 1893

##### Distribution

Scotland

##### Notes

added by Shaw and Kasparyan (2003)

#### Campodorus haematodes

(Gravenhorst, 1829)

Tryphon haematodes Gravenhorst, 1829
alni (Woldstedt, 1874, *Mesoleius*)

##### Distribution

England, Ireland

#### Campodorus hamulus

(Gravenhorst, 1829)

Tryphon hamulus Gravenhorst, 1829
nobilis (Holmgren, 1857, *Mesoleius*)

##### Distribution

England

#### Campodorus ignavus

(Holmgren, 1857)

Mesoleius ignavus Holmgren, 1857

#### Campodorus immarginatus

(Thomson, 1893)

Mesoleius immarginatus Thomson, 1893

##### Distribution

Scotland

##### Notes

Transferred from *Mesoleius* by Shaw and Kasparyan (2003).

#### Campodorus incidens

(Thomson, 1893)

Mesoleius incidens Thomson, 1893

##### Distribution

England, Scotland

#### Campodorus liosternus

(Thomson, 1893)

Mesoleius liosternus Thomson, 1893

#### Campodorus luctuosus

(Holmgren, 1857)

Mesoleius luctuosus Holmgren, 1857

#### Campodorus maculicollis

(Stephens, 1835)

Tryphon maculicollis Stephens, 1835
vigens (Holmgren, 1857, *Mesoleius*)

##### Distribution

England, Scotland

#### Campodorus marginalis

(Geoffroy, 1785)

Ichneumon marginalis Geoffroy, 1785
limbarius (Olivier, 1792, *Ichneumon*)
histrio (Fabricius, 1793, *Ichneumon*)
formosus (Gravenhorst, 1829, *Tryphon*)
limbarius (Fonscolombe, 1854, *Lissonota*)
lepidus (Giraud, 1872, *Mesoleius*); synonymy by Horstmann (2008c)
ornatus (Habermehl, 1925, *Mesoleius*); synonymy by Horstmann (2008c)

##### Distribution

England

#### Campodorus mediosanguineus

(Heinrich, 1950)

Mesoleius mediosanguineus Heinrich, 1950

##### Distribution

Ireland

##### Notes

BMNH, det. Broad, added here

#### Campodorus melanogaster

(Holmgren, 1857)

Mesoleius melanogaster Holmgren, 1857

##### Distribution

England, Scotland

##### Notes

added by Shaw and Kasparyan (2003)

#### Campodorus mixtus

(Holmgren, 1857)

Mesoleius mixtus Holmgren, 1857

#### Campodorus molestus

(Holmgren, 1857)

Mesoleius molestus Holmgren, 1857

#### Campodorus nigridens

(Thomson, 1893)

Spudaeus nigridens Thomson, 1893

##### Distribution

Scotland

#### Campodorus patagiatus

(Holmgren, 1876)

Mesoleius patagiatus Holmgren, 1876
modestus (Holmgren, 1876, *Mesoleius*)

#### Campodorus pectinator

Kasparyan, 2003

##### Distribution

England

##### Notes

added by Kasparyan (2003)

#### Campodorus pictipes

(Habermehl, 1923)

Mesoleius pictipes Habermehl, 1923

##### Notes

Described from a Carr specimen, this species should be deleted from the list if there are no other British or Irish specimens (see note under *Lissonota funebris*). Transferred from *Mesoleius* by Horstmann (2000e).

#### Campodorus scapularis

(Stephens, 1835)

Tryphon scapularis Stephens, 1835
humerellus (Thomson, 1893, *Mesoleius*); synonymy by Kasparyan (2003)

##### Distribution

England, Scotland

#### Campodorus tristis

(Holmgren, 1857)

Mesoleius tristis Holmgren, 1857

#### Campodorus variegatus

(Jurine, 1807)

Anomalon variegatum Jurine, 1807
sanguinicollis (Gravenhorst, 1829, *Tryphon*)

##### Distribution

England, Scotland

#### Campodorus viduus

(Holmgren, 1857)

Mesoleius viduus Holmgren, 1857
annulatus (Brischke, 1878, *Trematopygus*)

##### Distribution

England

##### Notes

distribution data from UM

#### Campodorus vitosaensis

(Gregor, 1933)

Mesoleius vitosaensis Gregor, 1933
trochanteratus (Kriechbaumer, 1896, *Mesoleius*) preocc.

##### Notes

*Mesoleius trochanteratus* Kriechbaumer is listed as a subspecies of *vitosaensis* in Yu and Horstmann (1997), but is preoccupied by *Mesoleius trochanteratus* Brischke, 1871. The value of subspecific names in such little-known species is doubtful anyway.

#### Himerta

Förster, 1869

CLEPSIPORTHUS Förster, 1869
DOLIOCTONUS Förster, 1869
ENOECETIS Förster, 1869
ITHAGENES Förster, 1869
HIMERTUS Thomson, 1883

##### Notes

Distribution data from Horstmann (2002a) who also provides synonymy, and BMNH.

#### Himerta bisannulata

(Thomson, 1883)

Euryproctus bisannulatus Thomson, 1883
pfeifferi (Bauer, 1939, *Himertus*)

##### Notes

Added by Horstmann (2002a)and taken out of synonymy with *defectiva*; one specimen in BMNH, ‘British Isles, Desvignes coll.’

#### Himerta defectiva

(Gravenhorst, 1820)

Ichneumon defectivus Gravenhorst, 1820
varicornis (Gravenhorst, 1829, *Tryphon*)
biannulata (Ulbricht, 1922, *Barytarbes*)
ihsseni (Bauer, 1939, *Himertus*)

##### Distribution

England, Scotland

#### Himerta scutellaris

(Kriechbaumer, 1897)

Enoecetis scutellaris Kriechbaumer, 1897
NHM, det. Broad, added

##### Distribution

England

##### Notes

BMNH, det. Broad, added here

#### Himerta sepulchralis

(Holmgren, 1876)

Mesoleius sepulchralis Holmgren, 1876
sexannulatus Kriechbaumer, 1891
alboannulata (Strobl, 1903, *Euryproctus*)

##### Distribution

England, Scotland

#### Hyperbatus

Förster, 1869

##### Notes

Distribution data from Shaw and Kasparyan (2003).

#### Hyperbatus orbitalis

(Thomson, 1893)

Mesoleius orbitalis Thomson, 1893

##### Distribution

England, Scotland

##### Notes

Added by Shaw and Kasparyan (2003); removed from synonymy with *segmentator* by Kasparyan (1998).

#### Hyperbatus segmentator

(Holmgren, 1857)

Mesoleius segmentator Holmgren, 1857
solitarius (Holmgren, 1876, *Mesoleius*)

##### Distribution

Scotland

#### Hyperbatus sternoxanthus

(Gravenhorst, 1829)

Tryphon sternoxanthus Gravenhorst, 1829
pulchellus (Holmgren, 1857, *Mesoleius*)

##### Distribution

England

#### Lagarotis

Förster, 1869

DASPLETIS Förster, 1869
DYSANTES Förster, 1869
NYTHOPHONA Förster, 1869
ONEISTA Förster, 1869
LAGAROTUS Thomson, 1892

#### Lagarotis debitor

(Thunberg, 1824)

Ichneumon debitor Thunberg, 1824
insolens (Gravenhorst, 1829, *Tryphon*)

##### Distribution

England

#### Lagarotis erythrocera

(Gravenhorst, 1829)

Tryphon erythrocerus Gravenhorst, 1829

##### Distribution

England

##### Notes

Placed in *Lagarotis* in Yu and Horstmann (1997) and by Horstmann (2011b), although Aubert (2000) treated this as a species of *Alexeter*.

#### Lagarotis semicaligata

(Gravenhorst, 1820)

Ichneumon semicaligatus Gravenhorst, 1820
longicornis (Woldstedt, 1874, *Mesoleius*)

##### Distribution

England, Scotland

#### Lamachus

Förster, 1869

ADEXIOMA Förster, 1869
ZAPHTHORA Förster, 1869
BATHYGLYPTUS Schmiedeknecht, 1913
TOROCAMPUS Schmiedeknecht, 1913

#### Lamachus coalitorius

(Thunberg, 1824)

Ichneumon coalitorius Thunberg, 1824
variabilis (Ratzeburg, 1844, *Tryphon*); synonymy by Horstmann (2004b)
ophthalmicus (Holmgren, 1857, *Mesoleius*)
marginatus (Brischke, 1871, *Mesoleius*)
spectabilis (Holmgren, 1876, *Mesoleius*)

##### Distribution

England, Wales

##### Notes

Also released, for biocontrol (Billany et al. 1983).

#### Lamachus eques

(Hartig, 1838)

Tryphon eques Hartig, 1838
silvarum (Holmgren, 1876, *Mesoleius*)
aterrimus (Scönwiese, 1934, *Torocampus*)

##### Distribution

England, Scotland

##### Notes

Distribution data from Shaw and Kasparyan (2003)

#### Lamachus frutetorum

(Hartig, 1838)

Tryphon frutetorum Hartig, 1838
lophyrum (Hartig, 1838, *Tryphon*)
nigrescens Kiss, 1926
altipeta Heinrich, 1953

##### Distribution

Scotland

##### Notes

added by Aubert (2000)

#### Lamachus pini

(Bridgman, 1882)

Mesoleius pini Bridgman, 1882
caledonicus Laidlaw, 1933

##### Distribution

Scotland

#### Lamachus virgultorum

(Gravenhorst, 1829)

Tryphon virgultorum Gravenhorst, 1829
flavoscutellatus (Strobl, 1903, *Notopygus*)

##### Distribution

England

#### Mesoleius

Holmgren, 1856

ALLOCRITUS Förster, 1869
ALFKENIA Pfankuch, 1906
MESOLIUS Pfankuch, 1906
HABRODEMUS Schmiedeknecht, 1913

##### Notes

Distribution data principally from Shaw and Kasparyan (2003), plus Fitton (1976).

doubtfully placed species of *Mesoleius*:

[*brachyacanthus* Parfitt, 1881 nom. dub., from England]

#### Mesoleius aceris

Kasparyan & Shaw, 2003

##### Distribution

England, Scotland, Isle of Man

##### Notes

added by Shaw and Kasparyan (2003)

#### Mesoleius armillatorius

(Gravenhorst, 1807)

Ichneumon armillatorius Gravenhorst, 1807
luteifrons (Gravenhorst, 1829, *Tryphon*)
flavipes Brischke, 1871; synonymy by Horstmann (2012b)
similis Brischke, 1892
bilineolatus Strobl, 1903; synonymy by Horstmann (2012b)
chyzeri (Kiss, 1926, *Perilissus*); synonymy by Horstmann (2007b)

##### Distribution

England, Scotland, Wales, Isle of Man

#### Mesoleius aulicus

(Gravenhorst, 1829)

Tryphon aulicus Gravenhorst, 1829

##### Distribution

England, Scotland

#### Mesoleius axillaris

(Stephens, 1835)

Tryphon axillaris Stephens, 1835
amabilis Holmgren, 1857
leptogaster Holmgren, 1857
tenuiventris Holmgren, 1858
erythrogaster Holmgren, 1876

##### Distribution

England, Scotland, Wales Isle of Man

##### Notes

Transferred from *Campodorus* by Kasparyan (2000), who provides synonymy.

#### Mesoleius brevipalpis

Thomson, 1893

##### Distribution

England

##### Notes

added by Shaw and Kasparyan (2003)

#### Mesoleius caninae

Bridgman, 1886

##### Distribution

England

##### Notes

Transferred from *Otlophorus* (where it is listed as a synonym of *O. congruens* (Holmgren) by Yu and Horstmann 1997) by Kasparyan (2000).

#### Mesoleius dubius

Holmgren, 1857

##### Distribution

England

#### Mesoleius filicornis

Holmgren, 1876

##### Distribution

England, Scotland, Wales, Isle of Man

#### Mesoleius flavopictus

(Gravenhorst, 1829)

Mesoleptus flavopictus Gravenhorst, 1829
trimaculatus (Stephens, 1835, *Mesoleptus*)

##### Distribution

England, Scotland

#### Mesoleius frenalis

Thomson, 1893

#### Mesoleius furax

Holmgren, 1857

#### Mesoleius fuscipes

Holmgren, 1857

##### Distribution

Scotland, Isle of Man

##### Notes

Transferred from *Campodorus* by Kasparyan (2000).

#### Mesoleius geniculatus

Holmgren, 1857

##### Distribution

Wales, Scotland

##### Notes

added by Shaw and Kasparyan (2003)

#### Mesoleius intermedius

(Gravenhorst, 1829)

Tryphon intermedius Gravenhorst, 1829
sinuatus Thomson, 1893; synonymy by Kasparyan (2000)

##### Distribution

England, Wales

##### Notes

Added by Shaw and Kasparyan (2003); transferred from *Campodorus* by Kasparyan (2000).

#### Mesoleius laricis

Teunissen, 1953

##### Distribution

Scotland

##### Notes

added by Shaw and Kasparyan (2003)

#### Mesoleius lindemansi

Teunissen, 1953

antennator Kasparyan, 2000; synonymy by Kasparyan (2004b)

##### Distribution

England, Scotland

##### Notes

added by Shaw and Kasparyan (2003)

#### Mesoleius melanoleucus

(Gravenhorst, 1829)

Tryphon melanoleucus Gravenhorst, 1829

##### Distribution

England, Scotland

#### Mesoleius nivalis

Holmgren, 1857

aemulus (Ruthe, 1859, *Tryphon*)

##### Distribution

England, Scotland, Wales, Isle of Man

#### Mesoleius opticus

(Gravenhorst, 1829)

Tryphon opticus Gravenhorst, 1829

##### Distribution

England, Scotland, Ireland

#### Mesoleius peronatus

(Marshall, 1876)

Bassus peronatus Marshall, 1876

##### Distribution

Ireland

##### Notes

Transferred from *Campodorus* by Kasparyan (2000).

#### Mesoleius phyllotomae

Cushman, 1933

##### Distribution

Scotland

##### Notes

added by Shaw and Kasparyan (2003)

#### Mesoleius placidus

Holmgren, 1857

#### Mesoleius pyriformis

(Ratzeburg, 1852)

Tryphon pyriformis Ratzeburg, 1852
unifasciatus Holmgren, 1857

##### Distribution

England, Scotland

#### Mesoleius ribesii

Bauer, 1961

##### Distribution

England, Scotland

##### Notes

added by Shaw & Kasparyan (2003)

#### Mesoleius roepkii

Teunissen, 1945

##### Distribution

Wales, Scotland

##### Notes

added by Shaw and Kasparyan (2003)

#### Mesoleius tenthredinis

Morley, 1912

romani Teunissen, 1945

##### Distribution

England

##### Notes

distribution data from UM

#### Mesoleius varicoxa

Thomson, 1893

#### Otlophorus

Förster, 1869

AEOLOMETIS Förster, 1869
DIALGES Förster, 1869
HOLMGRENIA Förster, 1869
NEALES Förster, 1869
TACHYPORTHUS Förster, 1869
AELOMETIS Thomson, 1893
OTLOPHORINUS Hincks, 1944

#### Otlophorus anceps

(Holmgren, 1857)

Mesoleius anceps Holmgren, 1857

##### Distribution

England

##### Notes

added by Aubert (2000)

#### Otlophorus congruens

(Holmgren, 1858)

Mesoleius congruens Holmgren, 1858

##### Distribution

England

##### Notes

added by Aubert (2000)

#### Otlophorus italicus

(Gravenhorst, 1829)

Scolobates italicus Gravenhorst, 1829
corallinus (Vollenhoven, 1873, *Scolobates*)

##### Distribution

England

#### Otlophorus pulverulentus

(Holmgren, 1857)

Mesoleius pulverulentus Holmgren, 1857
minutus (Rudow, 1881, *Meniscus*)

##### Distribution

England

#### Otlophorus senilis

(Holmgren, 1876)

Mesoleius senilis Holmgren, 1876

##### Distribution

England

##### Notes

BMNH, det. Aubert, added here

#### Otlophorus vepretorum

(Gravenhorst, 1829)

Tryphon vepretorum Gravenhorst, 1829
verpetorum misspelling

##### Distribution

England

#### Perispuda

Förster, 1869

GENARCHES Förster, 1869
ZAPLETHIS Förster, 1869
PERISPUDUS Thomson, 1888

#### Perispuda bignellii

(Bridgman, 1881)

Mesoleius bignellii Bridgman, 1881
flavitarsis (Thomson, 1893, *Mesoleius*)
sulphuripes (Strobl, 1902, *Procinetus*)

##### Distribution

England, Scotland, Ireland

##### Notes

As a synonym of *sulphurata* in Yu and Horstmann (1997) but treated as a separate species by Aubert (2000).

#### Perispuda facialis

(Gravenhorst, 1829)

Mesoleptus facialis Gravenhorst, 1829

##### Distribution

England

#### Perispuda sulphurata

(Gravenhorst, 1807)

Ichneumon sulphuratus Gravenhorst, 1807

##### Distribution

England, Ireland

#### Protarchus

Förster, 1869

ZACALLES Förster, 1869

#### Protarchus melanurus

(Thomson, 1893)

Mesoleius melanurus Thomson, 1893
rufus misident.

##### Distribution

England, Scotland

##### Notes

Added by Aubert (2000); also specimens in UM

#### Protarchus testatorius

(Thunberg, 1824)

Ichneumon testatorius Thunberg, 1824
binarius (Thunberg, 1824, *Ichneumon*) preocc.
decorius (Thunberg, 1824, *Ichneumon*)
rufus (Gravenhorst, 1829, *Tryphon*)
conspicuus (Stephens, 1835, *Tryphon*)
rufulus (Stephens, 1835, *Tryphon*)

##### Distribution

England, Scotland

#### Rhinotorus

Förster, 1869

SPUDAEA Förster, 1869
SPUDAEUS Thomson, 1883
PROSPUDAEA Hincks, 1944

#### Rhinotorus compactor

(Thunberg, 1824)

Ichneumon compactor Thunberg, 1824
atratus (Holmgren, 1857, *Trematopygus*)
quadriguttatus (Vollenhoven, 1873, *Bassus*)
albotrochanteratus (Strobl, 1913, *Polyblastus*)

##### Distribution

England

#### Rhinotorus leucostomus

(Gravenhorst, 1829)

Tryphon leucostomus Gravenhorst, 1829
impressus (Brischke, 1871, *Mesoleius*)
subimpressus (Thomson, 1873, *Spudaeus*)

##### Distribution

England

#### Rhinotorus longicornis

(Schmiedeknecht, 1914)

Spudaea longicornis Schmiedeknecht, 1914

##### Distribution

England

#### Rhinotorus mesocastanus

(Thomson, 1892)

Spudaeus mesocastanus Thomson, 1892

##### Distribution

England

##### Notes

BMNH, det. Reshchikov, added here

#### Rhinotorus nasutus

(Gravenhorst, 1829)

Tryphon nasutus Gravenhorst, 1829
confusus (Thomson, 1883, *Mesoleius*)

##### Distribution

England

##### Notes

BMNH, det. Perkins, added here

#### Rhinotorus similis

(Brischke, 1892)

Mesoleius similis Brischke, 1892

#### Saotis

Förster, 1869

SAOTUS Thomson, 1883
ISKARUS Kolarov, 1987; synonymy by Kasparyan and Shaw (2003)

##### Notes

Distribution data taken from Kasparyan and Shaw (2003), Shaw and Kasparyan (2003), Kasparyan and Khalaim (2007a) and Fitton (1976).

#### Saotis albionis

Kasparyan, 2007

##### Distribution

England

##### Notes

added by Kasparyan and Khalaim (2007a)

#### Saotis albiventris

Kasparyan, 2007

##### Distribution

England, Scotland

##### Notes

added by Kasparyan and Khalaim (2007a)

#### Saotis compressiuscula

(Thomson, 1883)

Mesoleius compressiusculus Thomson, 1883

##### Distribution

England

#### Saotis morleyi

Fitton, 1976

emarginata (Morley, 1911, *Homocidus*)

##### Distribution

England, Scotland

##### Notes

Listed as a synonym of *S. liopleuris* (Thomson, 1888, *Mesoleius*) in Yu and Horstmann (1997) but Kasparyan and Shaw (2003) treat the two as separate species.

#### Saotis nigriscuta

(Thomson, 1888)

Mesoleius nigriscuta Thomson, 1888

##### Distribution

England

##### Notes

added by Kasparyan and Shaw (2003)

#### Saotis renovata

(Morley, 1911)

Mesoleius renovatus Morley, 1911

##### Distribution

England

##### Notes

This species was not included in the key by Kasparyan and Shaw (2003) but they did include notes on its British occurrence.

#### Saotis varicoxa

(Thomson, 1893)

Saotus varicoxa Thomson, 1893

##### Distribution

England, Scotland

#### Scopesis

Förster, 1869

SCOPARCHES Förster, 1869
SCOPESUS Thomson, 1893

#### Scopesis bicolor

(Gravenhorst, 1829)

Tryphon bicolor Gravenhorst, 1829
praecatoria (Holmgren, 1876, *Mesoleius*)
longigena (Thomson, 1893, *Mesoleius*); synonymy by Horstmann (2006a)

##### Distribution

England

#### Scopesis depressa

(Thomson, 1893)

Mesoleius depressus Thomson, 1893

##### Distribution

England

#### Scopesis fraterna

(Holmgren, 1857)

Mesoleius fraternus Holmgren, 1857

##### Distribution

England

#### Scopesis frontator

(Thunberg, 1824)

Ichneumon frontator Thunberg, 1824
rufolabris (Zetterstedt, 1838, *Bassus*)

##### Distribution

England, Scotland

##### Notes

Aubert (2000) gives *guttiger* (Holmgren, 1857, *Mesoleius*) as a junior synonym but Horstmann (2006d) differentiates the taxa.

#### Scopesis gesticulator

(Thunberg, 1824)

Ichneumon gesticulator Thunberg, 1824
longipes (Gravenhorst, 1829, *Tryphon*)
nigricollis (Gravenhorst, 1829, *Tryphon*)

##### Distribution

England

#### Scopesis macropus

(Thomson, 1893)

Mesoleius macropus Thomson, 1893

##### Distribution

England

#### Scopesis obscura

(Holmgren, 1857)

Mesoleius obscurus Holmgren, 1857

#### Scopesis rufonotata

(Holmgren, 1876)

Mesoleius rufonotatus Holmgren, 1876
thomsoni (Habermehl, 1925, *Scopesus*)

##### Distribution

England

#### Scopesis tegularis

(Thomson, 1893)

Mesoleius tegularis Thomson, 1893

##### Distribution

England

##### Notes

distribution data from UM

#### Semimesoleius

Ozols, 1963

#### Semimesoleius exophthalmicus

Ozols, 1963

##### Distribution

Scotland

##### Notes

added by Shaw and Kasparyan (2003)

#### Smicrolius

Thomson, 1893

#### Smicrolius parvicalcar

(Thomson, 1895)

Syndipnus parvicalcar Thomson, 1895
parumpictus (Roman, 1909, *Mesoleius*); synonymy by Kasparyan (1998)

##### Distribution

England, Scotland

##### Notes

added by Shaw and Kasparyan (2003)

#### PERILISSINI

Thomson, 1883

#### Absyrtus

Holmgren, 1859

ECZETESIS Förster, 1869

#### Absyrtus vernalis

Bauer, 1961

##### Distribution

England

##### Notes

added by Aubert (2000)

#### Absyrtus vicinator

(Thunberg, 1824)

Ichneumon vicinator Thunberg, 1824
luteus Holmgren, 1859
exareolatus Ulbricht, 1926 unavailable

##### Distribution

England, Scotland, Wales, Ireland

#### Lathiponus

Förster, 1869

POLYSELASMUS Schmiedeknecht, 1912
CERATOSAOTIS Gregor, 1939

#### Lathiponus semiluctuosus

(Vollenhoven, 1878)

Eclytus semiluctuosus Vollenhoven, 1878
frigidus (Woldstedt, 1874, *Perilissus*) preocc.
bicolor (Brischke, 1878, *Perilissus*)
pulcherrimus (Thomson, 1888, *Mesoleius*)
ornatus (Gregor, 1939, *Ceratosaotis*)

##### Distribution

England

##### Notes

BMNH, NMS, det. Perkins, Kasparyan, Broad, added here

#### Lathrolestes

Förster, 1869

CAMPORYCHUS Förster, 1869
ECCLINOPS Förster, 1869
HOMALOMMA Förster, 1869
LAPHYROSCOPUS Förster, 1869
POLYONCUS Förster, 1869
LATHROLESTUS Thomson, 1883
LUPHYROSCOPUS Thomson, 1883
TRYPHONOPSIS Brauns, 1898
RITZEMABOSIA Smits
CULMINA Benoit, 1955

#### Lathrolestes bipunctatus

(Bridgman, 1886)

Grypocentrus bipunctatus Bridgman, 1886

##### Distribution

England, Scotland

#### Lathrolestes buccinator

(Holmgren, 1857)

Perilissus buccinator Holmgren, 1857
vollenhoveni (Gribodo, 1880, *Perilissus*); synonymy by Horstmann (2003a)

##### Distribution

England

##### Notes

Treated as a species of *Perilissus* by Horstmann (2003a) but as a species of *Lathrolestes* by Aubert (2000) and then by Horstmann (2004a).

#### Lathrolestes caudatus

(Thomson, 1883)

Lathrolestus caudatus Thomson, 1883

##### Distribution

England, Scotland

##### Notes

BMNH, NMS, added here

#### Lathrolestes citreus

(Brischke, 1878)

Perilissus citreus Brischke, 1878

##### Distribution

England

##### Notes

BMNH, added here

#### Lathrolestes clypeatus

(Zetterstedt, 1838)

Tryphon clypeatus Zetterstedt, 1838

##### Distribution

England, Scotland

##### Notes

Added by Heath (1961); overlooked by Fitton (1978); distribution data from Reshchikov (2015) and specimens in BMNH and UM.

#### Lathrolestes ensator

(Brauns, 1898)

Tryphonopsis ensator Brauns, 1898
dilatatus (Nordenström, 1905, *Lathrolestus*)
ensatrix (Schulz, 1906, *Tryphonopsis*)

##### Distribution

England

#### Lathrolestes erythrocephalus

(Gravenhorst, 1829)

Tryphon erythrocephalus Gravenhorst, 1829

##### Distribution

England

##### Notes

Transferred from *Perilissus* by Aubert (2000).

#### Lathrolestes lucidulus

(Holmgren, 1857)

Perilissus lucidulus Holmgren, 1857

##### Distribution

England

##### Notes

American Entomological Institute, det. Reshchikov, added here

#### Lathrolestes luteolator

(Gravenhorst, 1829)

Mesoleptus luteolator Gravenhorst, 1829
gorskii (Ratzeburg, 1852, *Tryphon*)

##### Distribution

England

#### Lathrolestes macropygus

(Holmgren, 1857)

Perilissus macropygus Holmgren, 1857

##### Distribution

England, Scotland

#### Lathrolestes moravicus

(Habermehl, 1923)

Perilissus moravicus Habermehl, 1923

##### Distribution

England

##### Notes

BMNH, NMS, added here

#### Lathrolestes nigricollis

(Thomson, 1883)

Perilissus nigricollis Thomson, 1883
minutus (Bridgman, 1887, *Perilissus*)

##### Distribution

England

#### Lathrolestes orbitalis

(Gravenhorst, 1829)

Tryphon orbitalis Gravenhorst, 1829
bucculentus (Holmgren, 1857, *Perilissus*)

##### Distribution

England, Scotland, Wales

#### Lathrolestes pictilis

(Holmgren, 1857)

Perilissus pictilis Holmgren, 1857

##### Distribution

England, Scotland, Ireland

##### Notes

Irish record based on a specimen in American Entomological Institute, det. A. Reshchikov.

#### Lathrolestes pleuralis

(Thomson, 1883)

Lathrolestus pleuralis Thomson, 1883

##### Distribution

England

#### Lathrolestes soperi

Reshchikov, 2010

##### Distribution

England

##### Notes

added by Reshchikov (2015)

#### Lathrolestes tripunctor

(Thunberg, 1824)

Ichneumon tripunctor Thunberg, 1824
distichor (Thunberg, 1824, *Ichneumon*)
longicornis (Brischke, 1871, *Perilissus*)
luteocephalus (Giraud, 1872, *Perilissus*)
singularis (Vollenhoven, 1878, *Perilissus*)
grandiceps (Thomson, 1883, *Perilissus*)

##### Distribution

England

##### Notes

BMNH, added here; transferred from *Perilissus* by Aubert (2000).

#### Lathrolestes ungularis

(Thomson, 1883)

Lathrolestus ungularis Thomson, 1883
citrofrontalis Schmiedeknecht, 1912; synonymy by Reshchikov (2011)

##### Distribution

England

#### Lathrolestes verticalis

(Brischke, 1871)

Perilissus verticalis Brischke, 1871
abdominalis (Brischke, 1878, *Perilissus*); synonymy by Horstmann (2006a)
marginatus (Thomson, 1883, *Lathrolestus*)

##### Distribution

England, Scotland, Ireland

#### Lophyroplectus

Thomson, 1883

##### Notes

Some distribution data from Huddleston and Gauld (1988).

#### Lophyroplectus oblongopunctatus

(Hartig, 1838)

Paniscus oblongopunctatus Hartig, 1838
luteator (Thunberg, 1824, *Ichneumon*)

##### Distribution

England

#### Oetophorus

Förster, 1869

SYMPHOBUS Förster, 1869

#### Oetophorus naevius

(Gmelin, 1790)

Ichneumon naevius Gmelin, 1790
dilector (Thunberg, 1824, *Ichneumon*)
limitaris (Gravenhorst, 1829, *Mesoleptus*)

##### Distribution

England, Scotland, Ireland, Isle of Man

#### Opheltes

Holmgren, 1859

##### Notes

Some distribution data from Huddleston and Gauld (1988).

#### Opheltes glaucopterus

(Linnaeus, 1758)

Ichneumon glaucopterus Linnaeus, 1758
pteromelas (Villers, 1789, *Ichneumon*)

##### Distribution

England, Scotland, Ireland

#### Perilissus

Holmgren, 1857

EXACRODUS Förster, 1869
ICHNAEOPS Förster, 1869
SPANOTECNUS Förster, 1869
UDENIA Förster, 1869
DAUGNA Seyrig, 1935
PSEUDOCHORUS Rao, 1953

#### Perilissus albitarsis

Thomson, 1883

emarginatus Thomson, 1883

##### Distribution

England, Scotland

##### Notes

BMNH, NMS, UM, added here

#### Perilissus compressus

Thomson, 1883

##### Distribution

England

##### Notes

BMNH, added here

#### Perilissus coxalis

Thomson, 1883

##### Distribution

England

##### Notes

BMNH, det. Broad, added here

#### Perilissus lutescens

Holmgren, 1857

##### Distribution

England

#### Perilissus pallidus

(Gravenhorst, 1829)

Mesoleptus pallidus Gravenhorst, 1829
holmgreni Habermehl, 1925

##### Distribution

England

#### Perilissus rufoniger

(Gravenhorst, 1820)

Ichneumon rufoniger Gravenhorst, 1820
vernalis (Gravenhorst, 1820, *Ichneumon*)
petulans (Gravenhorst, 1829, *Tryphon*)
herrichii Kriechbaumer, 1892

##### Distribution

England

#### Perilissus sericeus

(Gravenhorst, 1829)

Mesoleptus sericeus Gravenhorst, 1829
spiniger Thomson, 1883

#### Perilissus spilonotus

(Stephens, 1835)

Mesoleptus spilonotus Stephens, 1835
subcinctus Holmgren, 1857
stigmaticus Woldstedt, 1874
dissimilis Woldstedt, 1878
thuringiacus Schmiedeknecht, 1912
alpinus Habermehl, 1935

##### Distribution

England, Scotland, Wales

#### Perilissus variator

(Müller, 1776)

Ichneumon variator Müller, 1776
filicornis (Gravenhorst, 1820, *Ichneumon*)
interruptor (Thunberg, 1824, *Ichneumon*)
seminiger (Gravenhorst, 1829, *Mesoleptus*)

##### Distribution

England, Scotland, Wales, Ireland, Isle of Man

##### Notes

As *filicornis* in Aubert (2000).

#### Priopoda

Holmgren, 1856

PRIONOPODA misspelling

#### Priopoda apicaria

(Geoffroy, 1785)

Ichneumon apicarius Geoffroy, 1785
stictica misident.
luteolus (Thunberg, 1789, *Ichneumon*); synonymy by Horstmann (1999b)
glabrator (Thunberg, 1824, *Ichneumon*)

##### Distribution

England, Scotland

##### Notes

Some distribution data from Gauld (1970).

#### Priopoda xanthopsana

(Gravenhorst, 1829)

xanthospana misspelling

##### Distribution

England

#### Synoecetes

Förster, 1869

POLYRHYSIA Förster, 1869
SYNAGRYPNUS Förster, 1869
POLYRHYSIUS Thomson, 1893

#### Synoecetes anterior

(Thomson, 1893)

Syndipnus anterior Thomson, 1893

##### Distribution

England, Scotland, Ireland

##### Notes

BMNH, NMS, UM, added here

#### Trematopygodes

Aubert, 1968

#### Trematopygodes aprilinus

(Giraud, 1872)

Trematopygus aprilinus Giraud, 1872
blancoburgensis (Schmiedeknecht, 1912, *Lathrolestes*)

##### Distribution

England

##### Notes

added by Hinz and Horstmann (1998)

#### Trematopygodes rarus

Horstmann, 1990

##### Distribution

England

##### Notes

NMS, det. Kasparyan, added here

#### Zaplethocornia

Schmiedeknecht, 1912

#### Zaplethocornia exstinctor

Aubert, 1985

##### Distribution

England

##### Notes

added by Aubert (1985)

#### PIONINI

Smith & Shenefelt, 1955

#### Asthenara

Förster, 1869

ASTHENARUS Thomson, 1889

#### Asthenara scabricula

(Thomson, 1893)

Catoglyptus scabriculus Thomson, 1893

##### Distribution

England, Scotland, Wales, Ireland

##### Notes

BMNH, NMS, UM, added here

#### Asthenara socia

(Holmgren, 1857)

Euryproctus socius Holmgren, 1857
crassifemur (Thomson, 1889, *Asthenarus*)

##### Distribution

England, Scotland, Ireland

##### Notes

BMNH, NMS, added here

#### Glyptorhaestus

Thomson, 1894

LOXONEURUS Schmiedeknecht, 1913

#### Glyptorhaestus boschmai

Teunissen, 1953

##### Distribution

England

##### Notes

BMNH, NMS, added here

#### Glyptorhaestus periclistor

Hinz, 1975

##### Distribution

England

##### Notes

NMS, added here

#### Glyptorhaestus punctatus

(Thomson, 1890)

Rhaestus punctatus Thomson, 1890

##### Distribution

England

##### Notes

NMS, BMNH, added here

#### Glyptorhaestus punctulatus

(Woldstedt, 1877)

Mesoleius punctulatus Woldstedt, 1877
wuestneii (Thomson, 1893, *Rhaestus*)
thuringiacus (Schmiedeknecht, 1913, *Loxoneurus*)

##### Distribution

England

#### Glyptorhaestus selandrivorus

(Giraud, 1872)

Trematopygus selandrivorus Giraud, 1872

##### Distribution

England

##### Notes

BMNH, added here

#### Labrossyta

Förster, 1869

LABROSSYTUS Thomson, 1893
LIOTRYPHON Strobl, 1903

#### Labrossyta scotoptera

(Gravenhorst, 1820)

Ichneumon scotopterus Gravenhorst, 1820
fumata (Bridgman, 1880, *Perilissus*)

##### Distribution

England

#### Lethades

Davis, 1897

#### Lethades cingulator

Hinz, 1976

##### Distribution

England, Scotland

##### Notes

BMNH, NMS, added here

#### Lethades curvispina

(Thomson, 1883)

Trematopygus curvispina Thomson, 1883
alpinus (Zetterstedt, 1838, *Tryphon*) nom. ob.
flavifrons (Zetterstedt, 1838, *Tryphon*) nom. ob.

##### Distribution

Scotland

##### Notes

BMNH, NMS, added here; Aubert (2000) uses *alpinus* as the valid name but this is considered a nomen oblitum, as is *flavifrons* (Yu and Horstmann 1997).

#### Lethades facialis

(Brischke, 1871)

Trematopygus facialis Brischke, 1871

##### Distribution

England, Scotland, Wales

##### Notes

BMNH, det. Hinz & Horstmann, added here; material in BMNH has been determined by K. Horstmann as this species and *curvispina*; D.R. Kasparyan has determined Scottish material (Cairngorms) in NMS as *facialis*. Aubert (2000) treats *facialis* as a syonym of *alpinus* Zett. but it is recognised as a valid species by Hinz (1996).

#### Lethades imperfecti

Hinz, 1996

##### Distribution

England

##### Notes

added by Hinz (1996)

#### Lethades subcoriaceus

(Strobl, 1903)

Mesoleius subcoriaceus Strobl, 1903
laricis Hinz, 1976; synonymy by Horstmann (2011b)

##### Distribution

England, Scotland

##### Notes

NMS, det. Kasparyan, added here

#### Phaestus

Förster, 1869

#### Phaestus anomalus

(Brischke, 1871)

Grypocentrus anomalus Brischke, 1871
heterocerus (Thomson, 1893, *Rhaestus*)

##### Distribution

England

#### Pion

Schiødte, 1839

CATOGLYPTUS Förster, 1855

#### Pion nigripes

Schiødte, 1839

crassipes (Holmgren, 1857, *Catoglyptus*); synonymy by Horstmann (2004a)

##### Distribution

England, Scotland

##### Notes

BMNH, NMS, added here

#### Pion fortipes

(Gravenhorst, 1829)

Mesoleptus fortipes Gravenhorst, 1829
pictus (Pfankuch, 1924, *Catoglyptus*)
transsylvanicus (Kiss, 1924, *Mesoleptus*); synonymy by Horstmann (2007b)
clarus (Kiss, 1933, *Brischkea*); synonymy by Horstmann (2007b)

##### Distribution

England, Scotland, Ireland, Isle of Man

#### Rhaestus

Thomson, 1883

RHAESTES Förster, 1869

#### Rhaestus lativentris

(Holmgren, 1858)

Grypocentrus lativentris Holmgren, 1858

##### Distribution

England, Scotland

#### Rhaestus rufipes

(Holmgren, 1857)

Grypocentrus rufipes Holmgren, 1857
assimilis (Holmgren, 1858, *Trematopygus*)
femoralis Thomson, 1893

##### Distribution

England, Scotland, Ireland

##### Notes

added by Aubert (2000)

#### Rhorus

Förster, 1869

DOLICHOBLASTUS Strobl, 1903

##### Notes

Species excluded from the British and Irish list:

[*neustriae* (Schrank, 1802, *Ichneumon*)] *Ichneumon neustriae* has traditionally been treated as a species of *Rhorus* but the species involved has been in doubt (Aubert 1988) and Horstmann (2006a) tentatively transferred the species to *Cotesia* (Braconidae: Microgastrinae). According to Horstmann (2006a) *neustriae* auctt. is referable to *austriator* Aubert, 1988.

#### Rhorus anglicator

Aubert, 1988

##### Distribution

England, Scotland, Wales

##### Notes

added by Aubert (1988)

#### Rhorus binotatus

(Kriechbaumer, 1897)

Polyblastus binotatus Kriechbaumer, 1897

##### Distribution

England

##### Notes

added by Aubert (2000)

#### Rhorus brunnifemur

Kasparyan, 2015

##### Distribution

England, Scotland

##### Notes

added by Kasparyan 2015

#### Rhorus chrysopus

(Gmelin, 1790)

Ichneumon chrysopus Gmelin, 1790
caproni (Bridgman, 1882, *Monoblastus*)
capronii misspelling

##### Distribution

England, Scotland

#### Rhorus chrysopygus

(Roman, 1909)

Monoblastus chrysopyga Roman, 1909

##### Distribution

England

#### Rhorus exstirpatorius

(Gravenhorst, 1829)

Tryphon exstirpatorius Gravenhorst, 1829
laevigatus (Holmgren,1856, *Polyblastus*)
levigatus (Dalla Torre, 1901, *Monoblastus*)

##### Distribution

England, Scotland

##### Notes

added by Aubert (2000)

#### Rhorus fasciatus

(Gravenhorst, 1829)

Tryphon fasciatus Gravenhorst, 1829

##### Distribution

England

#### Rhorus flavopictus

(Strobl, 1903)

Monoblastus flavopictus Strobl, 1903
braunsi Habermehl, 1903

##### Distribution

England

##### Notes

BMNH, added here

#### Rhorus gauldi

Kasparyan, 2014

##### Distribution

England

##### Notes

added by Kasparyan (2014)

#### Rhorus lapponicus

(Roman, 1909)

Monoblastus lapponicus Roman, 1909

##### Distribution

England, Scotland

#### Rhorus laricis

Kasparyan, 2014

##### Distribution

Scotland

##### Notes

added by Kasparyan (2014)

#### Rhorus longicornis

(Holmgren, 1858)

Monoblastus longicornis Holmgren, 1858
glaber (Bridgman, 1886, *Prionopoda*)
flavomaculatus (Strobl, 1903, *Ischyrocnemis*); synonymy by Horstmann (2012b)
vitosaensis (Gregor, 1933, *Monoblastus*)

##### Distribution

England, Scotland

#### Rhorus longigena

(Thomson, 1883)

Monoblastus longigena Thomson, 1883

##### Distribution

Scotland

#### Rhorus neuter

Aubert, 1988

##### Distribution

England

##### Notes

added by Aubert (1988)

#### Rhorus nigrifrons

(Holmgren, 1883)

Polyblastus nigrifrons Holmgren, 1883

##### Distribution

Scotland

##### Notes

added by Kasparyan (2014)

#### Rhorus palustris

(Holmgren, 1857)

Polyblastus palustris Holmgren, 1857

##### Distribution

England, Scotland

#### Rhorus punctus

(Gravenhorst, 1829)

Tryphon punctus Gravenhorst, 1829
mesoxanthus (Gravenhorst, 1829, *Tryphon*)
scoticus (Desvignes, 1856, *Tryphon*)
conspicuus Kriechbaumer, 1891; synonymy by Horstmann (2001a)
spectabilis Kriechbaumer, 1891; synonymy by Horstmann (2001a)

##### Distribution

England

##### Notes

Treated as a synonym of *mesoxanthus* in Aubert (2000).

#### Rhorus romani

Kasparyan, 2014

##### Distribution

England

##### Notes

added by Kasparyan (2014)

#### Rhorus subfasciatus

(Stephens, 1835)

Tryphon subfasciatus Stephens, 1835

##### Distribution

England

#### Rhorus versator

Aubert, 1994

##### Distribution

Scotland

##### Notes

added by Aubert (1994)

#### Sympherta

Förster, 1869

ATRESTES Förster, 1869
CAMPOGENES Förster, 1869
STIPHROSOMUS Förster, 1869
TRAPEZOCORA Förster, 1869
EUSTIPHROSOMUS Hincks, 1944

#### Sympherta antilope

(Gravenhorst, 1829)

Mesoleptus antilope Gravenhorst, 1829
irata (Gravenhorst, 1829, *Tryphon*)
pulchricornis (Holmgren, 1857, *Catoglyptus*)
scabra (Brischke, 1871, *Catoglyptus*)

##### Distribution

England, Wales

#### Sympherta foveolator

(Holmgren, 1856)

Mesoleptus foveolator Holmgren, 1856

##### Distribution

England, Scotland

##### Notes

BMNH, Ely coll., det. Broad and W.A. Ely, added here

#### Sympherta obligator

(Thunberg, 1824)

Ichneumon obligator Thunberg, 1824
fuscicornis (Gmelin, 1790, *Ichneumon*)
waltoni (Curtis, 1837, *Mesoleptus*)

##### Distribution

England, Scotland

#### Sympherta splendens

(Strobl, 1903)

Catoglyptus splendens Strobl, 1903

##### Distribution

England

##### Notes

BMNH, added here

#### Sympherta sulcata

(Thomson, 1893)

Catoglyptus sulcatus Thomson, 1893

##### Notes

Added by Aubert (2000), who refers to material in BMNH but the only specimen that could be found is one labelled as *sulcatus* or *splendens* det. Aubert.

#### Sympherta tenthredinarum

Horstmann, 1999

ambulator (Thunberg, 1824, *Ichneumon*)

##### Distribution

England, Scotland

##### Notes

Replacement name, *ambulator* preoccupied; treated as a synonym of *jactator* Thunberg by Aubert (2000) but the two species were separated by Horstmann (1999b).

#### Sympherta ullrichi

(Tschek, 1869)

Catoglyptus ullrichi Tschek, 1869

##### Distribution

England, Wales, Ireland

##### Notes

BMNH, added here

#### Syntactus

Förster, 1869

TROMOPOEA Förster, 1869
BRISCHKEA Kriechbaumer, 1897

#### Syntactus delusor

(Linnaeus, 1758)

Ichneumon delusor Linnaeus, 1758
trochantericus (Geoffroy, 1785, *Ichneumon*)
parvulus (Kriechbaumer, 1897, *Brischkea*)

##### Distribution

England, Scotland

#### Syntactus minor

(Holmgren, 1857)

Catoglyptus minor Holmgren, 1857

##### Distribution

England

#### Syntactus minutus

(Bridgman, 1886)

Euryproctus minutus Bridgman, 1886

##### Distribution

England

#### Trematopygus

Holmgren, 1857

AMORPHOGNATHON Förster, 1869
ASELASMA Förster, 1869
CAMPOPORUS Förster, 1869
RHIGELUS Förster, 1869

#### Trematopygus horvathi

(Kiss, 1926)

Polyblastus horvathi Kiss, 1926

##### Distribution

Scotland

##### Notes

BMNH, det. Horstmann, added here; treated as a subspecies of *vellicans* by Hinz (1986), elevated to full species by Horstmann (2007b).

#### Trematopygus melanocerus

(Gravenhorst, 1829)

Tryphon melanocerus Gravenhorst, 1829
kriechbaumeri Thomson, 1893
thalhammeri Strobl, 1901
romani Heinrich, 1929

##### Distribution

England, Scotland, Wales

##### Notes

BMNH, added here

#### Trematopygus nigricornis

Holmgren, 1857

dictator (Thunberg, 1824, *Ichneumon*)

##### Distribution

England

#### Trematopygus rufator

Hinz, 1986

##### Distribution

England, Scotland

##### Notes

BMNH, det. Horstmann, added here; tentative identification of males.

#### Trematopygus spiniger

Hinz, 1976

##### Distribution

England

#### Trematopygus vellicans

(Gravenhorst, 1829)

Tryphon vellicans Gravenhorst, 1829
bicolor (Zetterstedt, 1838, *Bassus*)
ruficornis Holmgren, 1857

##### Distribution

England, Scotland

#### SCOLOBATINI

Schmiedeknecht, 1911

#### Scolobates

Gravenhorst, 1829

AGLYPHUS Giraud, 1872
PARABRACONIA Schmiedeknecht, 1914

#### Scolobates auriculatus

(Fabricius, 1804)

Ichneumon auriculatus Fabricius, 1804
auriculator (Thunberg, 1824, *Ichneumon*)
elevator (Thunberg, 1824, *Ichneumon*)
crassitarsus Gravenhorst, 1829
hylotomae Kriechbaumer, 1897
niger Roman, 1917
nigrifacies Teunissen, 1953

##### Distribution

England, Scotland, Wales, Ireland

### Cylloceriinae

#### CYLLOCERIINAE

Wahl, 1990

#### Allomacrus

Förster, 1869

SIBIRIAKOFFIA Holmgren, 1880
KENTROTRYPHON Strobl, 1903 synonymy by Schwarz (2003)

#### Allomacrus arcticus

(Holmgren, 1880)

Sibiriakoffia arctica Holmgren, 1880
pimplarius Thomson, 1888

##### Distribution

England, Scotland, Ireland

##### Notes

BMNH, NMS, UM, added here

#### Cylloceria

Schiødte, 1838

CHALINOCERUS Ratzeburg, 1852
ASPHRAGIS Förster, 1869

##### Notes

Most distribution data from BMNH and NMS, some from Fitton (1976) and Rossem (1981).

#### Cylloceria caligata

(Gravenhorst, 1829)

Phytodietus caligatus Gravenhorst, 1829
nunciator misident.
nigra (Gravenhorst, 1829, *Phytodietus*)
crenicornis (Curtis, 1832, *Lampronota*)
nuntiator (Zetterstedt, 1838, *Bassus*)
manca (Ruthe, 1855, *Chalinocerus*)

##### Distribution

England, Ireland

##### Notes

Raised from synonymy (Dasch 1992, Yu and Horstmann 1997) by Humala (2002). Humala (2002) did not report synonymy, which therefore mostly follows Rossem (1981).

#### Cylloceria melancholica

(Gravenhorst, 1820)

Ichneumon melancholicus Gravenhorst, 1820
accusator misidentification
defectiva (Gravenhorst, 1829, *Lissonota*)
affinis (Zetterstedt, 1838, *Bassus*)
marginator Schiødte, 1838
denticornis (Haliday, 1839, *Lampronota*)
fracticornis (Haliday, 1839, *Lampronota*)
longicornis (Ratzeburg, 1852, *Chalinocerus*)
marginatrix (Schulz, 1906, *Lampronota*)
rugulosa (Haupt, 1917, *Tropistes*)
altior (Heinrich, 1953, *Chalinocerus*)

##### Distribution

England, Scotland, Ireland

#### Cylloceria sylvestris

(Gravenhorst, 1829)

Tryphon sylvestris Gravenhorst, 1829
striolata (Hellén, 1915, *Lampronota*)

##### Distribution

England

##### Notes

BMNH, NMS, det. Broad, added here

#### Hyperacmus

Holmgren, 1858

CUSHMANIA Dasch, 1992 synonymy by Wahl and Gauld (1998)

##### Notes

*Hyperacmus* has sometimes been included in the Microleptinae *sensu stricto* (e.g. Humala 1997, Yu and Horstmann 1997) but the Microleptinae is restricted to the single genus, *Microleptes* (Broad 2004). Wahl and Gauld (1998) argued that *Hyperacmus* is better placed in the Orthocentrinae but, on the basis of phylogenetic studies (Quicke et al. 2009), *Hyperacmus* is placed here in the Cylloceriinae.

#### Hyperacmus crassicornis

(Gravenhorst, 1829)

Exochus crassicornis Gravenhorst, 1829
brunniventris (Rudow, 1883, *Exochus*)
suerinensis (Brauns, 1905, *Lampronota*) synonymy by Humala (2002)

##### Distribution

England, Scotland, Ireland

### Diacritinae

#### DIACRITINAE

Townes, 1965

##### Notes

Treated by Fitton et al. (1988), who give distribution data, as a tribe of the Pimplinae.

#### Diacritus

Förster, 1869

PHIDIAS Vollenhoven, 1878 preocc.
STENOLABIS Kriechbaumer, 1894
PHOSPHORUS Rossem, 1981 preocc.
PHOSPHORIANA Rossem, 1987 synonymy by Humala (2007)

#### Diacritus aciculatus

(Vollenhoven, 1878)

Phidias aciculatus Vollenhoven, 1878
cingulatus (Kriechbaumer, 1894, *Stenolabis*)
rugosissima (Strobl, 1904, *Entypoma*) synonymy by Humala (2007)

##### Distribution

England, Scotland, Ireland

### Diplazontinae

#### DIPLAZONTINAE

Viereck, 1918

##### Notes

Unless noted otherwise, distribution data from Beirne (1941), Kerrich (1949), Cowin (1953), Klopfstein (2014) and the collections of BMNH, NMS and UM, with data on type localities from Fitton (1976). Material collected by Fraser et al. (2007) is deposited in NMS. Additional references are given. Taxonomy, including synonymy, follows Klopfstein (2014).

#### Bioblapsis

Förster, 1869

TRICHOMASTIX Vollenhoven, 1878

#### Bioblapsis cultiformis

(Davis, 1897)

Otoblastus cultiformis Davis, 1897
mallochi Rotheray, 1990

##### Distribution

England, Scotland

##### Notes

added by Rotheray (1990)

#### Bioblapsis polita

(Vollenhoven, 1878)

Trichomastix polita Vollenhoven, 1878
flavipes (Holmgren, 1858, *Bassus*) preocc.
tibialis (Bridgman, 1883, *Bassus*) preocc.

##### Distribution

England, Scotland

#### Campocraspedon

Uchida, 1957

#### Campocraspedon annulitarsis

(Hedwig, 1838)

Homocidus annulitarsis Hedwig, 1838
arcanus (Stelfox, 1941, *Homocidus*)

##### Distribution

England, Scotland, Wales, Ireland

#### Campocraspedon caudatus

(Thomson, 1890)

Homotropus caudatus Thomson, 1890

##### Distribution

England, Scotland, Wales, Ireland, Isle of Man

#### Diplazon

Nees, 1819

BASSUS misident.

#### Diplazon albotibialis

Dasch, 1964

alpinus (Holmgren, 1858, *Bassus*) preocc.
neoalpinus Zwakhals, 1979

##### Distribution

England, Scotland, Ireland

#### Diplazon annulatus

(Gravenhorst, 1829)

Bassus annulatus Gravenhorst, 1829
lapponicus (Zetterstedt, 1838, *Bassus*)

##### Distribution

England, Scotland, Wales, Ireland, Isle of Man

##### Notes

*Diplazon multicolor* (Gravenhorst, 1829, *Bassus*) was removed from synonymy by Klopfstein (2011).

#### Diplazon deletus

(Thomson, 1890)

Bassus deletus Thomson, 1890
rufigaster Dasch, 1964

##### Distribution

England, Scotland, Wales, Ireland

#### Diplazon laetatorius

(Fabricius, 1781)

Ichneumon laetatorius Fabricius, 1781
dichrous (Schrank, 1781, *Ichneumon*)
albovarius (Wollaston, 1858, *Bassus*)
cinctipes (Holmgren, 1868, *Bassus*)
varipes (Smith, 1878, *Scolobates*)
venustulus (Saussure, 1892, *Bassus*)
balearicus (Kriechbaumer, 1894, *Bassus*)

##### Distribution

England, Scotland, Wales, Ireland, Isle of Man

#### Diplazon pectoratorius

(Thunberg, 1824)

Ichneumon pectoratorius Thunberg, 1824
angustorius Thunberg, 1824, Ichneumon)
pectoratorius (Gravenhorst, 1829, *Bassus*) preocc.
nigrithorax (Strobl, 1902, *Homotropus*)
akaashii (Uchida, 1931, *Homocidus*)
urupensis (Uchida, 1935, *Bassus*)

##### Distribution

England, Scotland, Wales, Ireland, Isle of Man

#### Diplazon scutatorius

Teunissen, 1943

pilosus Uchida, 1957
tetragonopsis Uchida, 1957

##### Distribution

England

##### Notes

Added by Thirion (1987); *Diplazon tetragonopsis* is listed as a synonym of *tetragonus* by Yu and Horstmann (1997) but Diller (1982) and Klopfstein (2014) treat it as a synonym of *scutatorius*.

#### Diplazon tetragonus

(Thunberg, 1824)

Ichneumon tetragonus Thunberg, 1824
hortorius (Thunberg, 1824, *Ichneumon*)
ustorius (Thunberg, 1824, *Ichneumon*)
tricinctus (Gravenhorst, 1829, *Bassus*)
nemoralis (Holmgren, 1858, *Bassus*)

##### Distribution

England, Scotland, Wales, Ireland, Isle of Man

#### Diplazon tibiatorius

(Thunberg, 1824)

Ichneumon tibiatorius Thunberg, 1824
albosignatus (Gravenhorst, 1829, *Bassus*)

##### Distribution

England, Scotland, Wales, Ireland, Isle of Man

##### Notes

some distribution data from Askew (2000)

#### Diplazon varicoxa

(Thomson, 1890)

Bassus varicoxa Thomson, 1890

##### Distribution

England, Scotland, Ireland, Isle of Man

#### Enizemum

Förster, 1869

#### Enizemum nigricorne

(Thomson, 1890)

Homotropus nigricornis Thomson, 1890

##### Distribution

Ireland

##### Notes

Fitton and Rotheray (1982) doubted whether the few British specimens identified as *nigricorne* were distinct from *ornatum*. One apparently British specimen in BMNH (Capron coll., no other details) is *nigricorne* (det. Broad and Klopfstein). Beirne (1941) records *nigricorne* from Ireland.

#### Enizemum ornatum

(Gravenhorst, 1829)

Bassus ornatus Gravenhorst, 1829
deplanatum (Gravenhorst, 1829, *Bassus*)
carinulatum (Ruthe, 1859, *Bassus*)
frenator (Desvignes, 1862, *Bassus*)
sumptuosum (Schmiedeknecht, 1926, *Homocidus*)

##### Distribution

England, Scotland, Wales, Ireland, Isle of Man

#### Enizemum scutellare

(Lange, 1911)

Homotropus scutellaris Lange, 1911
albopictum (Lange, 1911, *Homotropus*) preocc.
rubiginosum (Schmiedeknecht, 1926, *Homocidus*)

##### Distribution

Wales

##### Notes

added by Klopfstein (2014)

#### Enizemum tridentatum

Dasch, 1964

##### Distribution

Ireland

##### Notes

BMNH, det. Klopfstein, Broad, added here

#### Eurytyloides

Nakanishi, 1978

#### Eurytyloides umbrinus

Klopfstein, 2014

##### Distribution

England

##### Notes

added by Klopfstein (2014)

#### Fossatyloides

Klopfstein, Quicke, Kropf & Frick, 2011

#### Fossatyloides gracilentus

(Holmgren, 1858)

Bassus gracilentus Holmgren, 1858
pulcher (Holmgren, 1858, *Bassus*) preocc.

##### Distribution

England, Scotland, Wales

#### Homotropus

Förster, 1869

HOMOCIDUS Morley, 1911

##### Notes

*Homotropus* species have generally been included in *Syrphoctonus*; generic combinations and species-level taxonomy follow Klopfstein (2014).

species incertae sedis within *Homotropus*:

[*impolitus* (Stelfox, 1941, *Homocidus*), from Scotland] Known only from males, Klopfstein (2014) was unable to place this taxon but suggested that it may be a colour variant of *pallipes*.

#### Homotropus collinus

(Stelfox, 1941)

Homocidus collinus Stelfox, 1941
simulans (Stelfox, 1941, *Homocidus*)

##### Distribution

England, Scotland, Ireland

#### Homotropus crassicornis

Thomson, 1890

brevicornis Thomson, 1890
asyntactus (Schmiedeknecht, 1926, *Homocidus*)

##### Distribution

England, Scotland, Wales, Isle of Man

#### Homotropus dimidiatus

(Schrank, 1802)

Ichneumon dimidiatus Schrank, 1802
planus (Desvignes, 1862, *Bassus*)
crassicrus Thomson, 1890

##### Distribution

England, Scotland

#### Homotropus elegans

(Gravenhorst, 1829)

Bassus elegans Gravenhorst, 1829
rufonotatus (Holmgren, 1858, *Bassus*)
affinis Szépligeti, 1898

##### Distribution

England, Scotland, Wales, Isle of Man

#### Homotropus frontorius

(Thunberg, 1824)

Ichneumon frontorius Thunberg, 1824
subopacus (Stelfox, 1941, *Homocidus*)

##### Distribution

England, Scotland, Wales, Ireland

#### Homotropus haemorrhoidalis

Szépligeti, 1898

rhenanus (Habermehl, 1930, *Homocidus*)
struvei (Hedwig, 1939, *Homocidus*)
tricolor (Stelfox, 1941, *Homocidus*)
lipothrix Momoi, 1973

##### Distribution

England, Scotland, Wales, Ireland

#### Homotropus longiventris

Thomson, 1890

##### Distribution

England, Scotland, Ireland

#### Homotropus megaspis

Thomson, 1890

megalaspis Schulz, 1906

##### Distribution

England, Scotland, Ireland

#### Homotropus melanogaster

(Holmgren, 1872)

Bassus melanogaster Holmgren, 1872

##### Distribution

England

##### Notes

added by Klopfstein (2014)

#### Homotropus nigritarsus

(Gravenhorst, 1829)

Bassus nigritarsus Gravenhorst, 1829
picitans (Desvignes, 1862, *Bassus*)
groenlandicus (Holmgren, 1872, *Bassus*)

##### Distribution

England, Scotland, Wales, Ireland, Isle of Man

#### Homotropus pallipes

(Gravenhorst, 1829)

Bassus pallipes Gravenhorst, 1829
pectoralis (Gravenhorst, 1829, *Lissonota*)
pallidipes (Marshall, 1872, *Bassus*)
pallidipennis Dalla Torre, 1901
pallidipes Dalla Torre, 1901

##### Distribution

England, Scotland, Ireland, Isle of Man

#### Homotropus pectoralis

(Provancher, 1874)

Bassus pectoralis Provancher, 1874
incisus Thomson, 1890
reflexus Morley, 1906

##### Distribution

England, Scotland, Wales

#### Homotropus pictus

(Gravenhorst, 1829)

Bassus pictus Gravenhorst, 1829
nigricornis (Zetterstedt, 1838, *Tryphon*)
pumilus (Holmgren, 1858, *Bassus*)
thoracicus (Desvignes, 1862, *Bassus*)
brevis (Hedwig, 1938, *Homocidus*)

##### Distribution

England, Scotland, Wales, Ireland, Isle of Man

#### Homotropus signatus

(Gravenhorst, 1829)

Bassus signatus Gravenhorst, 1829
hygrobius Thomson, 1890
bifoveolatus Kriechbaumer, 1894

##### Distribution

England, Scotland, Ireland, Isle of Man

#### Homotropus strigator

(Fabricius, 1793)

Ichneumon strigator Fabricius, 1793
ruficornis (Holmgren, 1858, *Bassus*) preocc.

##### Distribution

England, Wales

#### Homotropus sundevalli

(Holmgren, 1858)

Bassus sundevalli Holmgren, 1858
scabrosus (Desvignes, 1862, *Bassus*)

##### Distribution

England, Scotland, Wales

##### Notes

some distribution data from Rotheray (1986)

#### Phthorima

Förster, 1869

PHTHORIMUS Thomson, 1890

##### Notes

Distribution data from Fitton and Boston (1988).

#### Phthorima compressa

(Desvignes, 1856)

Bassus compressus Desvignes, 1856
ibalioidis (Kriechbaumer, 1878, *Bassus*)
nigra (Morley, 1906, *Homotropus*)

##### Distribution

England, Scotland, Wales, Ireland

#### Phthorima picta

(Habermehl, 1925)

Phthorimus picta Habermehl, 1925
gaullei Seyrig, 1928

##### Distribution

England, Ireland

##### Notes

added by Fitton and Boston (1988)

#### Phthorima xanthaspis

(Thomson, 1890)

Homotropus xanthaspis Thomson, 1890

##### Distribution

England

##### Notes

added by Fitton and Boston (1988)

#### Promethes

Förster, 1869

LIOPSIS Förster, 1869
PROMETHUS Thomson, 1890

#### Promethes bridgmani

Fitton, 1976

scutellaris (Bridgman, 1886, *Bassus*) preocc.

##### Distribution

England, Scotland, Wales, Ireland

#### Promethes sulcator

(Gravenhorst, 1829)

Bassus sulcator Gravenhorst, 1829
areolatus (Holmgren, 1859, *Bassus*)
anomalus (Taschenberg, 1865, *Orthopelma*)
dodsi (Morley, 1906, *Promethus*)

##### Distribution

England, Scotland, Wales, Ireland, Isle of Man

#### Sussaba

Cameron, 1909

#### Sussaba cognata

(Holmgren, 1858)

Bassus cognatus Holmgren, 1858
albicoxa (Thomson, 1890, *Promethus*)

##### Distribution

England, Scotland, Wales, Ireland, Isle of Man

#### Sussaba dorsalis

(Holmgren, 1858)

Bassus dorsalis Holmgren, 1858
maculata (Desvignes, 1862, *Bassus*)

##### Distribution

England, Scotland, Wales, Ireland

#### Sussaba erigator

(Fabricius, 1793)

Ichneumon erigator Fabricius, 1793
festiva (Fabricius, 1798, *Ichneumon*)
festivator (Fabricius, 1804, *Ophion*)

##### Distribution

England

##### Notes

The only English specimens listed by Kerrich (1949) (and possibly the same as those listed by Beirne 1941) were supposedly from the Lichfield district (Carr (1924)) and are thus inadmissable (Perkins 1953, Shaw 2003). There are specimens in BMNH recently identified by S. Klopfstein.

#### Sussaba flavipes

(Lucas, 1849)

Bassus flavipes Lucas, 1849
pulchella misident.
neopulchella Diller, 1980
coriacea Dasch, 1964

##### Distribution

England, Scotland, Wales, Ireland

##### Notes

Sometimes identified as *coriacea*, but this is now classified as the Nearctic subspecies of *flavipes* (Yu and Horstmann 1997).

#### Sussaba placita

Dasch, 1964

punctiventris misident.

##### Distribution

England

##### Notes

The only British specimens labelled as *punctiventris* in BMNH were misidentified *flavipes*, and Beirne (1941) did not mention any British or Irish specimens. According to S. Klopfstein (pers. comm.), specimens identified as *punctiventris* (Thomson, 1890, *Homotropus*) by Fraser et al. (2007) are actually *placita* and the species was subsequently recorded as *placita* by Mayhew et al. (2009).

#### Sussaba pulchella

(Holmgren, 1858)

Bassus pulchellus Holmgren, 1858
elongata (Provancher, 1874, *Bassus*)
monticola (Vollenhoven, 1880, *Bassus*)
laticarpus (Thomson, 1890, *Promethus*)
ruthei (Roman, 1931, *Promethes*)

##### Distribution

England, Scotland, Wales, Ireland, Isle of Man

##### Notes

The name *pulchella* has frequently been applied to *flavipes*.

#### Syrphoctonus

Förster, 1869

##### Notes

Klopfstein et al. (2011) reassigned several species to *Homotropus* and *Fossatyloides* with other remaining species formally transferred by Klopfstein (2014).

#### Syrphoctonus desvignesii

(Marshall, 1870)

Bassus desvignesii Marshall, 1870
pulcher misident.
pulchellus (Desvignes, 1862, *Bassus*) preocc.
neopulcher Horstmann, 1968

##### Distribution

England, Scotland, Wales, Ireland, Isle of Man

#### Syrphoctonus fissorius

(Gravenhorst, 1829)

Bassus fissorius Gravenhorst, 1829
punctatus (Bridgman, 1887, *Bassus*)
similis (Lange, 1911, *Homotropus*)

##### Distribution

England, Scotland, Wales, Ireland, Isle of Man

#### Syrphoctonus tarsatorius

(Panzer, 1809)

Bassus tarsatorius Panzer, 1809
exsultans (Gravenhorst, 1829, *Bassus*)
insignis (Gravenhorst, 1829, *Bassus*)
flavus (Desvignes, 1862, *Bassus*)
indicus (Cameron, 1909, *Bassus*)
eximius (Habermehl, 1922, *Homotropus*)
flavitrochanterus (Uchida, 1957, *Homotropus*)

##### Distribution

England, Scotland, Wales, Ireland, Isle of Man

#### Syrphophilus

Dasch, 1964

#### Syrphophilus bizonarius

(Gravenhorst, 1829)

Bassus bizonarius Gravenhorst, 1829
cingulatus (Holmgren, 1858, *Bassus*)
frontalis (Brischke, 1878, *Bassus*) preocc.
iwatensis (Uchida, 1930, *Homocidus*)
satoi (Uchida, 1930, *Homocidus*)

##### Distribution

England, Scotland, Wales, Ireland

#### Syrphophilus tricinctorius

(Thunberg, 1824)

Ichneumon tricinctorius Thunberg, 1824
cinctus (Gravenhorst, 1829, *Bassus*)
lateralis (Gravenhorst, 1829, *Bassus*)
albicinctus (Desvignes, 1862, *Bassus*)
takaozanus (Uchida, 1930, *Homocidus*)

##### Distribution

England, Scotland, Wales, Ireland, Isle of Man

##### Notes

some distribution data from Askew (2000)

#### Tymmophorus

Schmiedeknecht, 1913

ZOOTREPHES misident.

#### Tymmophorus erythrozonus

(Förster, 1850)

Tryphon erythrozonus Förster, 1850
rufiventris (Gravenhorst, 1829, *Bassus*) preocc. (Horstmann 2006c)
holmgreni (Bridgman, 1882, *Bassus*)
lacustris Schmiedeknecht, 1913

##### Distribution

England

#### Tymmophorus obscuripes

(Holmgren, 1858)

Bassus obscuripes Holmgren, 1858
graculus misident.
rufocinctus (Desvignes, 1862, *Bassus*)
arcticus (Holmgren, 1869, *Bassus*)
luctuosus (Schmiedeknecht, 1926, *Promethes*)

##### Distribution

England, Scotland, Wales, Ireland, Isle of Man

##### Notes

*Bassus graculus* Gravenhorst, 1829, is a species of *Zoophthorus* (Cryptinae).

#### Tymmophorus suspiciosus

(Brischke, 1871)

Bassus suspiciosus Brischke, 1871

##### Distribution

England, Scotland, Ireland

##### Notes

Raised from synonymy with *erythrozonus* by Klopfstein (2014); only country-level data that can be directly attributable to each of these species are included here.

#### Woldstedtius

Carlson, 1979

SYRPHOCTONUS misident.

#### Woldstedtius bauri

Klopfstein, 2014

##### Distribution

England

##### Notes

added by Klopfstein (2014)

#### Woldstedtius biguttatus

(Gravenhorst, 1829)

Bassus biguttatus Gravenhorst, 1829
rufipes (Gravenhorst, 1829, *Bassus*)
confusus (Woldstedt, 1874, *Bassus*)

##### Distribution

England, Scotland, Wales, Ireland

#### Woldstedtius citropectoralis

(Schmiedeknecht, 1926)

Homocidus citropectoralis Schmiedeknecht, 1926
abdominator (Bridgman, 1886, *Bassus*) preocc.

##### Distribution

England, Scotland, Ireland

#### Woldstedtius flavolineatus

(Gravenhorst, 1829)

Bassus flavolineatus Gravenhorst, 1829
bimaculatus (Holmgren, 1858, *Bassus*)
interruptus (Holmgren, 1858, *Bassus*)

##### Distribution

England, Scotland, Wales

#### Woldstedtius holarcticus

(Diller, 1969)

Syrphoctonus holarcticus Diller, 1969

##### Distribution

England

##### Notes

added by Klopfstein (2014)

#### Xestopelta

Dasch, 1964

#### Xestopelta gracillima

(Schmiedeknecht, 1926)

Promethes gracillimus Schmiedeknecht, 1926
amabilis (Habermehl, 1935, *Homocidus*)

##### Distribution

England

##### Notes

added by Fitton and Rotheray (1982)

### Eucerotinae

#### EUCEROTINAE

Viereck, 1919

##### Notes

Distribution data from Fitton (1984) and the collections of NMS.

#### Euceros

Gravenhorst, 1829

EUMESIUS Westwood, 1840
OMALOCEROS Giraud, 1857
TAUTOZELUS Förster, 1869

#### Euceros albitarsus

Curtis, 1837

dimidiatus Brullé, 1846

##### Distribution

England, Ireland

#### Euceros pruinosus

(Gravenhorst, 1829)

Tryphon pruinosus Gravenhorst, 1829
crassicornis Gravenhorst, 1829
morionellus Holmgren, 1857
unifasciatus Vollenhoven, 1878
castaneus (Pfankuch, 1906, *Eumesius*) unavailable

##### Distribution

England, Scotland

##### Notes

Horstmann (2006a) removed *superbus* Kriechbeumer, 1888 from synonymy.

#### Euceros serricornis

Haliday, 1839

egregius Holmgren, 1857
grandicornis Holmgren, 1857

##### Distribution

England, Scotland, Ireland

### Hybrizontinae

#### HYBRIZONTINAE

Blanchard, 1845

PAXYLLOMATINAE Förster, 1862

##### Notes

Usually referred to as Paxyllomatinae (e.g. Fitton 1978, Mason 1981, Yu and Horstmann 1997), but Hybrizontinae has priority (Wharton and Achterberg 2000). Distribution data from Donisthorpe (1927), Achterberg (1999) and the collections of BMNH and NMS.

#### Ghilaromma

Tobias, 1988

#### Ghilaromma fuliginosi

(Donisthorpe & Wilkinson, 1930)

Paxylomma fuliginosi Donisthorpe & Wilkinson, 1930

##### Distribution

England

#### Hybrizon

Fallén, 1813

PAXYLLOMA Latreille, 1817
PLANCUS Curtis, 1833
PACHYLOMMA Ratzeburg, 1848

#### Hybrizon buccatus

(de Brébisson, 1825)

Paxylomma buccata de Brébisson, 1825
apicalis (Curtis, 1833, *Plancus*)
latebricola Nees, 1834

##### Distribution

England, Wales

##### Notes

*Hybrizon pubicornis* Zetterstedt, 1838, is listed as a synonym of *buccatus* in Yu and Horstmann (1997) but is actually a species of *Anteon* (Dryinidae) (Achterberg 1999).

### Ichneumoninae

#### ICHNEUMONINAE

Latreille, 1802

##### Notes

Distribution data from Perkins (1953), Perkins (1959), Perkins (1960), Fitton (1976), Boston and Nash (1989) and the collections of BMNH and NMS, except where noted. Additional distribution references are given.

#### EURYLABINI

Heinrich, 1934

#### Eurylabus

Wesmael, 1845

MISCHOPHORUS Kriechbaumer, 1894

#### Eurylabus larvatus

(Christ, 1791)

Ichneumon larvatus Christ, 1791
vinulatorius (Thunberg, 1824, *Ichneumon*)
intrepidus Wesmael, 1855
pestrei (Berthoumieu, 1892, *Catadelphus*)
flavosignatus (Kriechbaumer, 1894, *Mischophorus*)
vinulator Thomson, 1894
dusmeti (Berthoumieu, 1904, *Catadelphus*)

##### Distribution

England

#### Eurylabus torvus

Wesmael, 1845

##### Distribution

England, Wales, Ireland

#### Eurylabus tristis

(Gravenhorst, 1829)

Ichneumon tristis Gravenhorst, 1829
corvinus Wesmael, 1845

##### Distribution

England, Wales, Ireland

#### GOEDARTIINI

Townes, 1961

#### Goedartia

Boie, 1841

AUTOMALUS Wesmael, 1845

#### Goedartia alboguttata

(Gravenhorst, 1829)

Trogus alboguttatus Gravenhorst, 1829
affinis (Boie, 1841, *Trogus*)
baltica (Ratzeburg, 1844, *Ichneumon*)
dimidiativentris (Rudow, 1888, *Amblyteles*)

##### Distribution

England, Wales, Ireland

##### Notes

Welsh specimens in World Museum Liverpool, det. T. Hunter.

#### HERESIARCHINI

Ashmead, 1900

PROTICHNEUMONINI Heinrich, 1934
CALLAJOPPINI Heinrich, 1962 synonymy by Sime and Wahl (2002)
TROGINI Förster, 1869 synonymy by Sime and Wahl (2002)

#### Amblyjoppa

Cameron, 1902

#### Amblyjoppa fuscipennis

(Wesmael, 1845)

Amblyteles fuscipennis Wesmael, 1845

##### Distribution

England, Wales, Ireland

#### Amblyjoppa proteus

(Christ, 1791)

Ichneumon proteus Christ, 1791
laminatoria (Fabricius, 1798, *Ichneumon*)
nigratoria (Fabricius, 1798, *Ichneumon*) preocc.
bilineator (Donovan, 1810, *Ichneumon*) synonymy by Horstmann (1997)
nigriculus (Walkley, 1958, *Ichneumon*)

##### Distribution

England, Scotland, Ireland

#### Callajoppa

Cameron, 1903

#### Callajoppa cirrogaster

(Schrank, 1781)

Ichneumon cirrogaster Schrank, 1781
cirrogastra misspelling
nigrocaudata (Retzius, 1783, *Ichneumon*)
crocata (Geoffroy, 1785, *Ichneumon*)
lutoria (Fabricius, 1787, *Ichneumon*)
rubricornuta (Christ, 1791, *Ichneumon*)
dessinator (Olivier, 1792, *Ichneumon*)
scutellaris (Olivier, 1792, *Ichneumon*)
imperatoria (Panzer, 1804, *Ichneumon*)
obscuratoria (Gravenhorst, 1807, *Ichneumon*)
atrocaudata (Stephens, 1835, *Trogus*)
excellens (Tischbein, 1882, *Trogus*)

##### Distribution

England, Ireland

#### Callajoppa exaltatoria

(Panzer, 1804)

Ichneumon exaltatorius Panzer, 1804
latoria (Thunberg, 1824, *Ichneumon*)
atropos (Curtis, 1828, *Ichneumon*)

##### Distribution

England

#### Coelichneumon

Thomson, 1893

##### Notes

species of *Coelichneumon* excluded from the British and Irish list

[*eximius* (Stephens, 1835, *Ichneumon*)] Listed in error by Fitton (1978), this species is North American (Perkins 1953).

#### Coelichneumon anthrax

(Dalla Torre, 1901)

Ichneumon anthrax Dalla Torre, 1901
anthracinus (Holmgren, 1864, *Ichneumon*) preocc.

##### Distribution

England

##### Notes

added by Riedel (2012)

#### Coelichneumon biannulatus

(Gravenhorst, 1820)

Ichneumon biannulatus Gravenhorst, 1820
auspex misident.
fasciatus (Gmelin, 1790, *Ichneumon*) preocc.
leucopis (Berthoumieu, 1894, *Ichneumon*) preocc., unavailable

##### Distribution

England, Wales

##### Notes

*Coelichneumon funebrator* Horstmann, 2006, (=*funebris* (Holmgren, 1864, *Ichneumon*) preocc.) was removed from synonymy by Horstmann (2006c).

#### Coelichneumon biguttorius

(Thunberg, 1789)

Ichneumon biguttorius Thunberg, 1789
microstictus misident. (Horstmann 2002c)
serenus (Gravenhorst, 1829, *Ichneumon*) synonymy by Riedel (2012)
restaurator (Fabricius, 1793, *Ichneumon*) preocc.
restritutor (Thunberg, 1824, *Ichneumon*)
laticeps (Rudow, 1888, *Amblyteles*)
rufiapicalis (Pic, 1914, *Ichneumon*)
transsylvanicus (Kiss, 1924, *Ichneumon*) synonymy by Riedel (2012)
concolor Heinrich, 1949 synonymy by Riedel (2012)

##### Distribution

England

#### Coelichneumon bilineatus

(Gmelin, 1790)

Ichneumon bilineatus Gmelin, 1790
pulsator (Panzer, 1804, *Ichneumon*)

##### Distribution

England, Ireland

#### Coelichneumon comitator

(Linnaeus, 1758)

Ichneumon comitator Linnaeus, 1758
auspex (Müller, 1776, *Ichneumon*)
biguttatus (Thunberg, 1784, *Ichneumon*)
tripunctorius (Thunberg, 1789, *Ichneumon*)
nigrator (Fabricius, 1793, *Ichneumon*) preocc.
narrator (Fabricius, 1804, *Ichneumon*)
restaurator (Gravenhorst, 1820, *Ichneumon*) preocc., synonymy by Horstmann (1998b)
fuscatorius (Thunberg, 1824, *Ichneumon*)
ferreus (Gravenhorst, 1829, *Ichneumon*)
coerulescens (Tischbein, 1879, *Ichneumon*)
purpurissatus Perkins, 1953 synonymy by Horstmann (2000b)

##### Distribution

England, Ireland

#### Coelichneumon consimilis

(Wesmael, 1845)

Ichneumon consimilis Wesmael, 1845
caelareator (Tischbein, 1881, *Ichneumon*)
nigripes (Kriechbaumer, 1894, *Ichneumon*) preocc., unavailable

##### Distribution

England, Wales, Ireland

#### Coelichneumon cyaniventris

(Wesmael, 1859)

Ichneumon cyaniventris Wesmael, 1859
biobliteratus (Pic, 1923, *Ichneumon*)
multialbonotatus (Pic, 1923, *Ichneumon*)

##### Distribution

England, Wales, Ireland

##### Notes

Irish occurrence from O'Connor (2004b)

#### Coelichneumon desinatorius

(Thunberg, 1824)

Ichneumon desinatorius Thunberg, 1824
fuscipes (Gmelin, 1790, *Ichneumon*) preocc.
subguttatus (Gravenhorst, 1829, *Ichneumon*)

##### Distribution

England, Scotland

#### Coelichneumon falsificus

(Wesmael, 1845)

Ichneumon falsificus Wesmael, 1845
specularis (Tischbein, 1881, *Ichneumon*) synonymy by Riedel (2012)
chevrieri (Pic, 1902, *Ichneumon*)

##### Distribution

England

#### Coelichneumon haemorrhoidalis

(Gravenhorst, 1820)

Ichneumon haemorrhoidalis Gravenhorst, 1820
castaniventris (Gravenhorst, 1829, *Ichneumon*) synonymy by Riedel (2012)
castanicauda (Tischbein, 1881, *Ichneumon*)
truncatulus (Thomson, 1886,)
secretus (Berthoumieu, 1894, *Ichneumon*) unavailable
subniger (Berthoumieu, 1894, *Ichneumon*) unavailable
strandi (Berthoumieu, 1910, *Ichneumon*)
binigronotatus (Pic, 1925, *Ichneumon*)
vulcanius (Pic, 1925, *Ichneumon*)
bipunctatus (Schmiedeknecht, 1928, *Ichneumon*) preocc.

##### Distribution

England, Ireland

#### Coelichneumon leucocerus

(Gravenhorst, 1820)

Ichneumon leucocerus Gravenhorst, 1820
solitarius (Thunberg, 1824, *Ichneumon*)
ligeris (Pic, 1923, *Ichneumon*)

##### Distribution

England, Ireland

#### Coelichneumon litoralis

Horstmann, 2000

purpurissatus misident. (Horstmann 2000b)

##### Distribution

England

##### Notes

added by Horstmann (2000b)

#### Coelichneumon nigerrimus

(Stephens, 1835)

Ichneumon nigerrimus Stephens, 1835
derasus (Wesmael, 1845, *Ichneumon*)
carbonator (Tischhbein, 1874, *Amblyteles*)
percussor (Tischbein, 1876, *Ichneumon*) synonymy by Riedel (2012)
minor (Kriechbaumer, 1894, *Ichneumon*) preocc., unavailable
annulatus Heinrich, 1929

##### Distribution

England, Scotland, Wales, Ireland

#### Coelichneumon oltenensis

Constantineanu, Pîrvescu & Mihalache, 1979

serenus misident.

##### Notes

Added by Horstmann (2002c)from ‘British Isles, Stephens Coll.’.

#### Coelichneumon orbitator

(Thunberg, 1824)

Ichneumon orbitator Thunberg, 1824
microstictus (Gravenhorst, 1829, *Ichneumon*) synonymy by Horstmann (2002c)
melanopyrrhus (Stephens, 1835, *Ichneumon*)
separator (Fonscolombe, 1847, *Ichneumon*) synonymy by Riedel (2012)
liocnemis (Thomson, 1888, *Ichneumon*)

##### Distribution

England

#### Coelichneumon ruficauda

(Wesmael, 1845)

Ichneumon ruficauda Wesmael, 1845

##### Distribution

England

#### Coelichneumon validus

(Berthoumieu, 1894)

Ichneumon validus Berthoumieu, 1894
nigricornis (Wesmael, 1845, *Ichneumon*) preocc.

##### Distribution

England, Scotland

#### Coelichneumonops

Heinrich, 1958

#### Coelichneumonops solutus

(Holmgren, 1864)

Ichneumon solutus Holmgren, 1864
chrysostomus (Thomson, 1896, *Ichneumon*) synonymy by Horstmann (1999a)
pictus (Roman, 1904, *Ichneumon*) preocc.

##### Distribution

Scotland

#### Heresiarches

Wesmael, 1859

#### Heresiarches eudoxius

(Wesmael, 1845)

Hepiopelmus eudoxius Wesmael, 1845

##### Distribution

England

#### Lymantrichneumon

Heinrich, 1978

#### Lymantrichneumon disparis

(Poda, 1761)

Sphex disparis Poda, 1761

##### Distribution

England

##### Notes

added by Broad and Davis (2015)

#### Protichneumon

Thomson, 1893

#### Protichneumon pisorius

(Linnaeus, 1758)

Ichneumon pisorius Linnaeus, 1758
fusorius misident.
lentorius (Panzer, 1799, *Ichneumon*)
fugatorius (Panzer, 1804, *Ichneumon*)
mediofulvus (Berthoumieu, 1894, *Ichneumon*) unavailable
dorsoniger Roman,1910

##### Distribution

England, Ireland

#### Protichneumon similatorius

(Fabricius, 1798)

Ichneumon similatorius Fabricius, 1798
Protichneumon similatorius ?*exspectorius* (Fabricius, 1794, *Ichneumon*)
erythrogaster (Stephens, 1835, *Ichneumon*) preocc.
coqueberti (Wesmael, 1848, *Ichneumon*)
dorsoniger (Berthoumieu, 1894, *Ichneumon*)

##### Distribution

England

##### Notes

*Amblyteles gigantorius* Holmgren, 1871 was removed from synonymy as it was found to be a junior synonym of *Protichneumon fusorius* (Linnaeus, 1761) (Horstmann 2008a).

#### Psilomastax

Tischbein, 1868

CERCODINOTOMUS Uchida, 1940

#### Psilomastax pyramidalis

Tischbein, 1868

pictus Kriechbaumer, 1882

##### Distribution

England

#### Syspasis

Townes, 1965

#### Syspasis carinator

(Fabricius, 1798)

Ichneumon carinator Fabricius, 1798
helleri (Holmgren, 1878, *Ichneumon*)
rufipes (Strobl, 1901, *Ichneumon*) preocc.
tenuidens (Berthoumieu, 1904, *Ichneumon*)

##### Distribution

England

##### Notes

NMS, det. Riedel, added here

#### Syspasis lineator

(Fabricius, 1781)

Ichneumon lineator Fabricius, 1781
trilineata (Gmelin, 1790, *Ichneumon*)
umbraculosa (Gravenhorst, 1829, *Ichneumon*)
binotata (Stephens, 1835, *Ichneumon*)
brischkii (Ratzeburg, 1852, *Ichneumon*)
adulator (Tischbein, 1881, *Ichneumon*)
calculosa (Berthoumieu, 1903, *Ichneumon*)

##### Distribution

England, Scotland, Ireland, Isle of Man

#### Syspasis rufina

(Gravenhorst, 1820)

Ichneumon rufinus Gravenhorst, 1820
Syspasis rufina ?*judex* (Müller, 1776, *Ichneumon*)

##### Distribution

England

#### Syspasis scutellator

(Gravenhorst, 1829)

Ichneumon scutellator Gravenhorst, 1829
rufescens (Berthoumieu, 1894, *Ichneumon*) preocc., unavailable

##### Distribution

England

#### Trogus

Panzer, 1806

DINOTOMUS Förster, 1869

#### Trogus lapidator

(Fabricius, 1787)

Ichneumon lapidator Fabricius, 1787
anthracinus (Scopoli, 1763, *Sphex*) nom. ob. (Horstmann 2001a)
coerulator (Weber, 1795, *Ichneumon*)
coerulator (Fabricius, 1804, *Ichneumon*) preocc.
saxator (Thunberg, 1824, *Ichneumon*)
fuscipennis Gravenhorst, 1829
violaceus (Mocsáry,1883, *Psilomastax*)
cyaneipennis Costa,1886
cyaneus (Kriechbaumer,1892, *Psilomastax*)
romani Uchida,1942
brevicaudae Heinrich,1975
panzeri Carlson,1975

##### Distribution

England

##### Notes

Distribution data from Shaw (1978); the English population has been treated as belonging to the subspecies *panzeri*, synonymised by Wahl and Sime (2006), along with many other names.

#### ICHNEUMONINI

Latreille, 1802

JOPPINI Kriechbaumer, 1898

#### Achaius

Cameron, 1903

#### Achaius margineguttatus

(Gravenhorst, 1829)

Ichneumon margineguttatus Gravenhorst, 1829
novitius (Wesmael, 1854, *Amblyteles*)
luteosignatus (Pic, 1914, *Amblyteles*)

##### Distribution

England, Scotland

#### Achaius oratorius

(Fabricius, 1793)

Ichneumon oratorius Fabricius, 1793
Achaius oratorius ?*dealbatus* (Gmelin, 1790, *Ichneumon*)
cingulatorius (Weber, 1801, *Ichneumon*) synonymy by Horstmann (1997)
atramentarius (Gravenhorst, 1829, *Ichneumon*)
cingulipes (Stephens, 1835, *Ichneumon*)
bipunctus (Berthoumieu, 1896, *Amblyteles*) unavailable
theresae (Pic, 1897, *Amblyteles*)
albocingulatus (Strobl, 1901, *Ichneumon*)
marginalis (Habermehl, 1903, *Amblyteles*)
bellus (Habermehl, 1917, *Spiloteles*)

##### Distribution

England, Scotland, Wales, Ireland, Isle of Man

#### Acolobus

Wesmael, 1845

#### Acolobus albimanus

(Gravenhorst, 1829)

Ichneumon albimanus Gravenhorst, 1829
buyssoni (Berthoumieu, 1892, *Ichneumon*)

#### Acolobus sericeus

Wesmael, 1845

##### Distribution

England

#### Amblyteles

Wesmael, 1845

##### Notes

Many species previously placed in *Amblyteles* (Perkins 1960) are now classified in *Achaius, Diphyus, Eutanyacra, Limerodops, Obtusodonta, Spilothyrateles* and *Triptognathus*.

#### Amblyteles armatorius

(Forster, 1771)

Ichneumon armatorius Forster, 1771
fasciatorius (Fabricius, 1775, *Ichneumon*)
notatorius (Villers, 1789, *Ichneumon*)
dimicatorius (Gmelin, 1790, *Ichneumon*)
signatorius (Olivier, 1792, *Ichneumon*)
diversorius (Stephens, 1835, *Ichneumon*)
regius Tischbein, 1868

##### Distribution

England, Scotland, Ireland, Isle of Man

#### Aoplus

Tischbein, 1874

#### Aoplus altercator

(Wesmael, 1855)

Ichneumon altercator Wesmael, 1855

##### Distribution

England, Scotland, Ireland

#### Aoplus castaneus

(Gravenhorst, 1820)

Ichneumon castaneus Gravenhorst, 1820
rufoniger (Tischbein, 1881, *Exephanes*)
mesopyrrhus (Kriechbaumer, 1893, *Ichneumon*) synonymy by Hinz and Horstmann (2000)
subniger (Berthoumieu, 1894, *Ichneumon*) unavailable
fieschensis (Pic, 1926, *Ichneumon*)
royatensis (Pic, 1926, *Ichneumon*)

##### Distribution

England, Scotland, Ireland

#### Aoplus defraudator

(Wesmael, 1845)

Ichneumon defraudator Wesmael, 1845
angustus (Tischbein, 1863, *Ichneumon*)
jemilleri (Kriechbaumer, 1893, *Ichneumon*) synonymy by Hinz and Horstmann (2000)
sabaudus (Berthoumieu, 1904, *Ichneumon*)

##### Distribution

Scotland, Ireland

#### Aoplus ochropis

(Gmelin, 1790)

Ichneumon ochropis Gmelin, 1790
ephippium (Rudow, 1886, *Cryptus*)

##### Distribution

England, Scotland, Ireland

#### Aoplus rubricosus

(Holmgren, 1864)

Ichneumon rubricosus Holmgren, 1864

##### Distribution

England

#### Aoplus ruficeps

(Gravenhorst, 1829)

Ichneumon ruficeps Gravenhorst, 1829
leucocrepis (Wesmael, 1857, *Ichneumon*)
maximorufus (Pic, 1927, *Ichneumon*)

##### Distribution

England, Scotland, Ireland

#### Baranisobas

Heinrich, 1972

#### Baranisobas ridibundus

(Gravenhorst, 1829)

Ichneumon ridibundus Gravenhorst, 1829
hassicus (Ratzeburg, 1848, *Ichneumon*)
variegator (Tischbein, 1881, *Exephanes*) synonymy by Hinz and Horstmann (2000)
polystictus (Kriechbaumer, 1887, *Ichneumon*)
instabilis (Berthoumieu, 1897, *Ichneumon*) preocc.
evianensis (Pic, 1902, *Ichneumon*)
insperatus (Dalla Torre, 1902, *Ichneumon*)
fallaciosus (Berthoumieu, 1903, *Ichneumon*)
bulsanensis (Smits$)

##### Distribution

England, Ireland

#### Barichneumon

Thomson, 1893

##### Notes

Many species previously placed in *Barichneumon* (Perkins 1960) are now classified in *Baranisobas, Stenobarichneumon, Virgichneumon* and *Vulgichneumon*.

#### Barichneumon anator

(Fabricius, 1793)

Ichneumon anator Fabricius, 1793
Barichneumon anator ?*biscutatus* (Gmelin, 1790, *Ichneumon*)
bulimorius (Thunberg, 1824, *Ichneumon*)
dealbator (Thunberg, 1824, *Ichneumon*)
femoratorius (Thunberg, 1824, *Ichneumon*)
retusorius (Thunberg, 1824, *Ichneumon*)
henschi (Schmiedeknecht, 1929, *Ichneumon*)

##### Distribution

England, Ireland

#### Barichneumon bilunulatus

(Gravenhorst, 1829)

Ichneumon bilunulatus Gravenhorst, 1829
sexlineatus (Gravenhorst, 1829, *Ichneumon*)
piniperdae (Hartig, 1838, *Phygadeuon*)
troscheli (Ratzeburg, 1844, *Ichneumon*)
imitator (Kriechbaumer, 1882, *Ichneumon*) preocc.
moraguesi (Kriechbaumer, 1894, *Ichneumon*)

##### Distribution

England, Ireland

#### Barichneumon chionomus

(Wesmael, 1845)

Ichneumon chionomus Wesmael, 1845

##### Distribution

England, Scotland, Ireland

#### Barichneumon derogator

(Wesmael, 1845)

Ichneumon derogator Wesmael, 1845

##### Distribution

England

#### Barichneumon gemellus

(Gravenhorst, 1829)

Ichneumon gemellus Gravenhorst, 1829
inversus (Kriechbaumer, 1893, *Ichneumon*) preocc.
carri Habermehl, 1923
controversus (Schmiedeknecht, 1928, *Ichneumon*)
rubricans (Schmiedeknecht, 1929, *Ichneumon*)
semirufus (Schmiedeknecht, 1929, *Ichneumon*) preocc.
constantineanui (Heinrich, 1972, *Stenobarichneumon*)

##### Distribution

England, Scotland, Ireland, Isle of Man

#### Barichneumon heracliana

(Bridgman, 1884)

Ichneumon heracliana Bridgman, 1884

##### Distribution

England, Isle of Man

#### Barichneumon peregrinator

(Linnaeus, 1758)

Ichneumon peregrinator Linnaeus, 1758
scriptorius (Thunberg, 1824, *Ichneumon*)
vacillatorius (Gravenhorst, 1829, *Ichneumon*) preocc.

##### Distribution

England, Scotland, Ireland, Isle of Man

#### Barichneumon plagiarius

(Wesmael, 1848)

Ichneumon plagiarius Wesmael, 1848
merkli (Kiss, 1915, *Plectocryptus*) synonymy by

#### Barichneumon praeceptor

(Thunberg, 1824)

Ichneumon praeceptor Thunberg, 1824
procerus (Gravenhorst, 1829, *Ichneumon*)
derivator (Wesmael, 1845, *Ichneumon*)
lunuliger (Kriechbaumer, 1890, *Ichneumon*) synonymy by Horstmann (2006a)
kervillei (Berthoumieu, 1903, *Ichneumon*)
cenisiensis (Berthoumieu, 1906, *Ichneumon*)
atricornis (Pic, 1926, *Ichneumon*)

##### Distribution

England, Scotland

#### Chasmias

Ashmead, 1900

CHASMODES Wesmael, 1845

#### Chasmias motatorius

(Fabricius, 1775)

Ichneumon motatorius Fabricius, 1775
importunus (Tischbein, 1874, *Ichneumon*)
transitorius (Berthoumieu, 1894, *Chasmodes*) unavailable
atronotatus Pic, 1917
berthoumieui Pic, 1917
bicoloripes Pic, 1917
diversipes Pic, 1917
rufonotatus Pic, 1917

##### Distribution

England, Ireland, Isle of Man

#### Chasmias paludator

(Desvignes, 1854)

Ichneumon paludator Desvignes, 1854
paludicola (Wesmael, 1857, *Chasmodes*)
dissimulator (Tischbein, 1881, *Ichneumon*)

##### Distribution

England

#### Cratichneumon

Thomson, 1893

#### Cratichneumon albifrons

(Stephens, 1835)

Ichneumon albifrons Stephens, 1835
gravenhorstii (Fonscolombe, 1847, *Ichneumon*)
grandiceps (Thomson, 1887, *Ichneumon*)

##### Distribution

England, Ireland

#### Cratichneumon coruscator

(Linnaeus, 1758)

Ichneumon coruscator Linnaeus, 1758
corruscator misspelling
ambulator (Müller, 1774, *Ichneumon*)
alacer (Gravenhorst, 1829, *Ichneumon*)
luridus (Gravenhorst, 1829, *Ichneumon*)
gasterator (Stephens, 1835, *Ichneumon*)
metaxanthus (Hartig, 1838, *Ichneumon*)
binotatus (Desvignes, 1856, *Ichneumon*) preocc.
pyrenaeus (Tischbein, 1882, *Ichneumon*)

##### Distribution

England, Wales, Ireland

#### Cratichneumon culex

(Müller, 1776)

Ichneumon culex Müller, 1776
tibialis (Geoffroy, 1785, *Ichneumon*)
clavipes (Gmelin, 1790, *Ichneumon*)
leucostoma (Gmelin, 1790, *Ichneumon*)
quadricolor (Gmelin, 1790, *Ichneumon*)
versicolor (Gmelin, 1790, *Ichneumon*)
annulator (Fabricius, 1793, *Ichneumon*) preocc.
fabricator (Fabricius, 1793, *Ichneumon*) synonymy by Horstmann (2001a)
crassator (Thunberg, 1824, *Ichneumon*)
infestor (Thunberg, 1824, *Ichneumon*)
viator (Thunberg, 1824, *Ichneumon*)
fulvipes (Stephens, 1835, *Ichneumon*)
ruficoxis Constantineanu, Andriescu & Ciochia, 1956

##### Distribution

England, Scotland, Wales, Ireland

#### Cratichneumon flavifrons

(Schrank, 1781)

Ichneumon flavifrons Schrank, 1781
fabricator misident. (Horstmann 2001a)
frontalis (Geoffroy, 1785, *Ichneumon*)
tricolor (Razoumowsky, 1789, *Ichneumon*) preocc.
generator (Olivier, 1792, *Ichneumon*)
maculifrons (Stephens, 1835, *Ichneumon*)
pyrrhopus (Stephens, 1835, *Ichneumon*)
extinctus (Ratzeburg, 1844, *Ichneumon*)
hartigii (Ratzeburg, 1844, *Ichneumon*)
impugnator (Wesmael, 1845, *Ichneumon*) preocc.
spiracularis (Tischbein, 1881, *Ichneumon*)
baudyi (Pic, 1902, *Ichneumon*)

##### Distribution

England, Scotland, Ireland

#### Cratichneumon fugitivus

(Gravenhorst, 1829)

Ichneumon fugitivus Gravenhorst, 1829
rutilus (Holmgren, 1864, *Ichneumon*) preocc.
capreolus (Berthoumieu, 1899, *Ichneumon*)

##### Distribution

England, Ireland

#### Cratichneumon infidus

(Wesmael, 1848)

Ichneumon infidus Wesmael, 1848
liostylus (Thomson, 1897, *Ichneumon*)

##### Distribution

England, Ireland

#### Cratichneumon jocularis

(Wesmael, 1848)

Ichneumon jocularis Wesmael, 1848
punctifrons (Holmgren, 1864, *Ichneumon*)
semiannulatus (Kriechbaumer, 1895, *Ichneumon*) preocc., synonymy by Horstmann (2002a)
angusteannulatus (Strobl, 1901, *Ichneumon*)

##### Distribution

England, Scotland, Ireland

##### Notes

Horstmann (2006a) removed *parvulus* (Kriechbaumer, 1887, *Ichneumon*) from synonymy.

#### Cratichneumon luteiventris

(Gravenhorst, 1820)

Ichneumon luteiventris Gravenhorst, 1820
indictus (Tischbein, 1874, *Ichneumon*)

#### Cratichneumon pallitarsis

(Thomson, 1887)

Ichneumon pallitarsis Thomson, 1887
palliditarsis (Berthoumieu, 1895, *Ichneumon*)

##### Distribution

Scotland

##### Notes

NMS, det. Riedel, added here; it is unclear whether J.F. Perkins overlooked earlier records of this species from Scotland or whether these were based on misidentifications.

#### Cratichneumon rufifrons

(Gravenhorst, 1829)

Ichneumon rufifrons Gravenhorst, 1829
frontatorius (Fabricius, 1793, *Ichneumon*) nom. ob., synonymy by Horstmann (2001a)
pallidiatorius (Gravenhorst, 1829, *Ichneumon*)

##### Distribution

England, Scotland, Ireland

#### Cratichneumon semirufus

(Gravenhorst, 1820)

Ichneumon semirufus Gravenhorst, 1820
nigroscutatus (Berthoumieu, 1895, *Ichneumon*)

##### Distribution

England, Ireland

#### Cratichneumon sicarius

(Gravenhorst, 1829)

Ichneumon sicarius Gravenhorst, 1829
nigratorius (Panzer, 1800, *Ichneumon*) preocc.
ingratorius (Gravenhorst, 1829, *Ichneumon*)
jugatus (Gravenhorst, 1829, *Ichneumon*)
alboannulatus (Strobl, 1901, *Ichneumon*)
atrocellaris (Pic, 1927, *Ichneumon*)
forticornis (Hedwig, 1956, *Hoplismenus*)

##### Distribution

England, Ireland

#### Cratichneumon versator

(Thunberg, 1824)

Ichneumon versator Thunberg, 1824
pallifrons (Gravenhorst, 1829, *Ichneumon*)
pallidifrons (Marshall, 1872, *Ichneumon*)
anotylus (Thomson, 1893, *Ichneumon*) synonymy by Riedel (2014)

##### Distribution

England, Scotland, Ireland

#### Cratichneumon viator

(Scopoli, 1763)

Ichneumon viator Scopoli, 1763
nigritarius (Gravenhorst, 1820, *Ichneumon*)
obfuscator (Thunberg, 1824, *Ichneumon*) preocc.
aethiops (Gravenhorst, 1829, *Ichneumon*)
pinetorum (Ratzeburg, 1852, *Ichneumon*)
parviscopa (Thomson, 1893, *Ichneumon*)
brischkei (Berthoumieu, 1895, *Ichneumon*) preocc., unavailable
nuperus (Berthoumieu, 1910, *Ichneumon*)
charadensis (Pic, 1924, *Ichneumon*)
atrifemur (Fahringer, 1943, *Ichneumon*)
rufipes Constantineanu, 1954

##### Distribution

England, Scotland, Wales, Ireland

#### Cratichneumon vulpecula

(Kriechbaumer, 1875)

Ichneumon vulpecula Kriechbaumer, 1875
pseudogracilentus (Strobl, 1901, *Ichneumon*)
hemerythrus Heinrich, 1949

##### Distribution

Scotland

##### Notes

NMS, det. Hilpert, added here

#### Crypteffigies

Heinrich, 1961

#### Crypteffigies albilarvatus

(Gravenhorst, 1820)

Ichneumon albilarvatus Gravenhorst, 1820
obscurior (Berthoumieu, 1895, *Ichneumon*) preocc., unavailable
deubeli (Kiss, 1924, *Megaplectes*)

##### Distribution

England, Scotland, Wales, Ireland

#### Crypteffigies lanius

(Gravenhorst, 1829)

Ichneumon lanius Gravenhorst, 1829
aberrans (Taschenberg, 1865, *Phygadeuon*)
muelleri (Kiss, 1929, *Plectocryptus*)

##### Distribution

England, Scotland, Ireland

#### Crypteffigies pseudocryptus

(Wesmael, 1857)

Ichneumon pseudocryptus Wesmael, 1857
punctulatus (Kriechbaumer, 1891, *Microcryptus*)

##### Distribution

England

#### Crytea

Cameron, 1906

#### Crytea sanguinator

(Rossi, 1794)

Ichneumon sanguinator Rossi, 1794
ruficollis (Stephens, 1835, *Ichneumon*) preocc.
discrepator (Wesmael, 1845, *Ichneumon*)
sanguinator (Desvignes, 1856, *Cryptus*) preocc.
multifarius (Berthoumieu, 1897, *Ichneumon*)

##### Distribution

England, Scotland, Ireland

#### Ctenichneumon

Thomson, 1894

DOCHYTELES Berthoumieu, 1904

#### Ctenichneumon castigator

(Fabricius, 1793)

Ichneumon castigator Fabricius, 1793
Ctenichneumon castigator ?*certator* (Müller, 1776, *Ichneumon*)
Ctenichneumon castigator ?*abrogator* (Schrank, 1781, *Ichneumon*)
Ctenichneumon castigator ?*cardui* (Schrank, 1786, *Ichneumon*)
Ctenichneumon castigator ?*adustus* (Gmelin, 1790, *Ichneumon*)
Ctenichneumon castigator ?*ruficingulus* (Schrank, 1802, *Ichneumon*)

##### Distribution

England, Ireland

#### Ctenichneumon devylderi

(Holmgren, 1871)

Amblyteles devylderi Holmgren, 1871
ineptus (Holmgren, 1871, *Amblyteles*)
tischbeini (Berthoumieu, 1896, *Amblyteles*) synonymy by Horstmann (2004c)

##### Distribution

England

#### Ctenichneumon divisorius

(Gravenhorst, 1820)

Ichneumon divisorius Gravenhorst, 1820
obsoletorius (Fabricius, 1793, *Ichneumon*) nom. ob., synonymy by Horstmann (2001a)
baeticus (Spinola, 1843, *Ichneumon*)
clipeator (Habermehl, 1917, *Dochyteles*)

##### Distribution

England, Scotland

#### Ctenichneumon edictorius

(Linnaeus, 1758)

Ichneumon edictorius Linnaeus, 1758
gladiatorius (Müller, 1776, *Ichneumon*)
fuscipes (Geoffroy, 1785, *Ichneumon*)
trichrous (Gmelin, 1790, *Ichneumon*)
erectorius (Fabricius, 1798, *Ichneumon*)
calceatorius (Panzer, 1801, *Ichneumon*)
amputatorius (Panzer, 1804, *Ichneumon*)
fossorius (Gravenhorst, 1820, *Ichneumon*) synonymy by Horstmann (2000b)
pallipes (Gravenhorst, 1820, *Ichneumon*)
depressorius (Thunberg, 1824, *Ichneumon*)
incertorius (Thunberg, 1824, *Ichneumon*)
perileucus (Gravenhorst, 1829, *Ichneumon*)
cognatus (Stephens, 1833, *Ichneumon*) synonymy by Horstmann (2000b)
nigricornis (Spinola, 1843, *Ichneumon*)
lotharingicus (Rudow, 1888, *Amblyteles*)
nigroscutellatus (Kriechbaumer, 1894, *Amblyteles*) unavailable
pallidipes (Dalla Torre, 1902, *Amblyteles*)

##### Distribution

England, Ireland

#### Ctenichneumon funereus

(Geoffroy, 1785)

Ichneumon funereus Geoffroy, 1785
funerarius (Olivier, 1792, *Ichneumon*)

##### Distribution

England, Wales

#### Ctenichneumon inspector

(Wesmael, 1845)

Amblyteles inspector Wesmael, 1845
nigriventris (Berthoumieu, 1896, *Amblyteles*)
brunnicans (Constantineanu, 1956, *Amblyteles*)

##### Distribution

England

#### Ctenichneumon melanocastanus

(Gravenhorst, 1820)

Ichneumon melanocastanus Gravenhorst, 1820
rubroater (Ratzeburg, 1852, *Ichneumon*)
erythropygus (Rudow, 1888, *Amblyteles*) preocc.

##### Distribution

England

#### Ctenichneumon messorius

(Gravenhorst, 1820)

Ichneumon messorius Gravenhorst, 1820
montivagus (Giraud, 1877, *Amblyteles*)

##### Distribution

England

#### Ctenichneumon nitens

(Christ, 1791)

Ichneumon nitens Christ, 1791
Ctenichneumon nitens ?*glabratorius* (Müller, 1776, *Ichneumon*)
vespertinus (Christ, 1791, *Ichneumon*)
mesocastanus (Gravenhorst, 1820, *Ichneumon*)
nigrocastaneus (Berthoumieu, 1896, *Amblyteles*) unavailable

##### Distribution

England

#### Ctenichneumon panzeri

(Wesmael, 1845)

Amblyteles panzeri Wesmael, 1845
flavocinctus (Desvignes, 1856, *Ichneumon*)
vexillarius (Tischbein, 1874, *Amblyteles*)
rufescens Morley, 1903
denticornis (Strobl, 1904, *Amblyteles*) synonymy by Horstmann (1999a)
styriacus (Strobl, 1904, *Amblyteles*) synonymy by Horstmann (1999a)
wormatiensis (Habermehl, 1909, *Amblyteles*)
nigrifemur (Ulbricht, 1926, *Amblyteles*) unavailable
rufifemur (Ulbricht, 1926, *Amblyteles*) unavailable

##### Distribution

England, Ireland, Isle of Man

#### Ctenochares

Förster, 1869

#### Ctenochares bicolorus

(Linnaeus, 1767)

Ichneumon bicolorus Linnaeus, 1767
instructor (Fabricius, 1793, *Ichneumon*)
deustor (Thunberg, 1824, *Ichneumon*)
rufator (Thunberg, 1824, *Ichneumon*)
apicalis (Wiedemann, 1824, *Ichneumon*)
apicalis (Brullé, 1846, *Joppa*)
xanthomelas (Brullé, 1846, *Ichneumon*)

##### Notes

Added by Jones (2001); possibly inadvertently introduced, as this is a widespread species in the Old World tropics, but it is known from as far north as Spain (M.R. Shaw, pers. comm.).

#### Deuterolabops

Heinrich, 1975

#### Deuterolabops eupitheciae

(Brischke, 1878)

Ichneumon eupitheciae Brischke, 1878
pulchellatus (Bridgman, 1889, *Ichneumon*)

##### Distribution

England, Scotland

#### Diphyus

Kriechbaumer, 1890

PHYSCOTELES Berthoumieu, 1904

#### Diphyus amatorius

(Müller, 1776)

Ichneumon amatorius Müller, 1776
laboratorius (Fabricius, 1793, *Ichneumon*) preocc.
nigronotatus (Pic, 1908, *Amblyteles*)

##### Distribution

England, Scotland, Ireland

#### Diphyus castanopyga

(Stephens, 1835)

Ichneumon castanopyga Stephens, 1835
rubriventris (Wesmael, 1845, *Amblyteles*)
bicristatus (Strobl, 1901, *Ichneumon*)

##### Distribution

England, Scotland, Ireland

#### Diphyus gradatorius

(Thunberg, 1824)

Ichneumon gradatorius Thunberg, 1824
egregius (Gravenhorst, 1829, *Ichneumon*)
sibiricus (Mocsáry, 1878, *Amblyteles*)
illustris (Kriechbaumer, 1894, *Ichneumon*) synonymy by Horstmann (2006a)
carlsbadensis (Pic, 1914, *Ichneumon*)
rufotriangularis (Pic, 1914, *Ichneumon*)

##### Distribution

Scotland

#### Diphyus longigena

(Thomson, 1888)

Amblyteles longigena Thomson, 1888
inermis (Berthoumieu, 1892, *Amblyteles*)

##### Distribution

England, Scotland, Wales

#### Diphyus luctatorius

(Linnaeus, 1758)

Ichneumon luctatorius Linnaeus, 1758
erratorius (Thunberg, 1824, *Ichneumon*)
litigiosus (Wesmael, 1854, *Amblyteles*)
oblongatus (Tischbein, 1873, *Ichneumon*)

##### Distribution

England, Scotland, Ireland

#### Diphyus mercatorius

(Fabricius, 1793)

Ichneumon mercatorius Fabricius, 1793
nugatorius (Fabricius, 1794, *Ichneumon*) synonymy by Horstmann (2001a)
nigricaudus (Berthoumieu, 1896, *Amblyteles*) preocc., unavailable

##### Distribution

England, Ireland

##### Notes

English record from P. Whitehead (pers. comm.)

#### Diphyus monitorius

(Panzer, 1801)

Ichneumon monitorius Panzer, 1801
quadrimaculatus (Schrank, 1802, *Ichneumon*) preocc.
interruptorius (Fabricius, 1804, *Ichneumon*)

#### Diphyus ochromelas

(Gmelin, 1790)

Ichneumon ochromelas Gmelin, 1790
pulchellus (Christ, 1791, *Ichneumon*)
negatorius (Fabricius, 1793, *Ichneumon*)
ornatorius (Panzer, 1800, *Ichneumon*)
umbratorius (Thunberg, 1824, *Ichneumon*)
sartorius (Gravenhorst, 1829, *Ichneumon*)
canaliculatus (Saussure, 1892, *Ichneumon*) preocc.
nigripes (Seyrig, 1928, *Spiloteles*)
trialbatus (Constantineanu, 1954, *Amblyteles*)

##### Distribution

England

#### Diphyus palliatorius

(Gravenhorst, 1829)

Ichneumon palliatorius Gravenhorst, 1829
Diphyus palliatorius ?*defensorius* (Villers, 1789, *Ichneumon*)
erythropygus (Gravenhorst, 1829, *Ichneumon*)
spoliator (Wesmael, 1845, *Amblyteles*)
ancipiterus (Desvignes, 1856, *Ichneumon*)
dubitatus (Desvignes, 1856, *Ichneumon*)
ochraceus (Tischbein, 1873, *Ichneumon*)
aequivocus (Tischbein, 1879, *Ichneumon*)
infinitus (Tischbein, 1879, *Ichneumon*)
gemmatus (Tischbein, 1881, *Ichneumon*)
laetus (Tischbein, 1881, *Ichneumon*) preocc.
brunneonotatus (Pic, 1898, *Amblyteles*)
atratus (Berthoumieu, 1901, *Amblyteles*)
rufotriangularis (Pic, 1915, *Amblyteles*) preocc.
subniger (Habermehl, 1929, *Amblyteles*) preocc.

##### Distribution

England, Scotland, Wales, Ireland

#### Diphyus quadripunctorius

(Müller, 1776)

Ichneumon quadripunctorius Müller, 1776
constellatus (Geoffroy, 1785, *Ichneumon*)
citreus (Christ, 1791, *Ichneumon*) synonymy by Horstmann (2001a)
intratorius (Fabricius, 1793, *Ichneumon*) synonymy by Horstmann (2001a)
jubilatorius (Müller, 1776, *Ichneumon*) synonymy by Horstmann (2001a)
pedatorius (Fabricius, 1793, *Ichneumon*) synonymy by Horstmann (2001a)
natatorius (Fabricius, 1798, *Ichneumon*)
mediatorius (Panzer, 1801, *Ichneumon*)
bipunctatus (Schrank, 1802, *Ichneumon*) preocc.
desertorius (Panzer, 1806, *Ichneumon*)
xanthozosmus (Gravenhorst, 1820, *Ichneumon*)
natator (Zetterstedt, 1838, *Ichneumon*)
infestorius (Fonscolombe, 1847, *Ichneumon*)
notatorius (Marshall, 1872, *Amblyteles*)
bipunctatus (Rudow, 1888, *Amblyteles*) preocc.
schrammi (Pic, 1827, *Amblyteles*) preocc.

##### Distribution

England, Wales, Ireland

#### Diphyus raptorius

(Linnaeus, 1758)

Ichneumon raptorius Linnaeus, 1758
quadriguttorius (Thunberg, 1824, *Ichneumon*)
gravenhorstii (Wesmael, 1836, *Ichneumon*) preocc.
flavaginis (Schiødte, 1839, *Ichneumon*) synonymy by Horstmann (2004a)
flavolaetus (Berthoumieu, 1896, *Amblyteles*) preocc., unavailable
quercus (Pic, 1917, *Amblyteles*)

##### Distribution

England

#### Diphyus salicatorius

(Gravenhorst, 1820)

Ichneumon salicatorius Gravenhorst, 1820
cinctorius (Stephens, 1835, *Ichneumon*) preocc.
indocilis (Wesmael, 1845, *Amblyteles*) synonymy by Horstmann (1998b)
relucens (Desvignes, 1856, *Ichneumon*)
inaciculatus (Pic, 1927, *Amblyteles*)
nigrobinotatus (Pic, 1927, *Amblyteles*)

##### Distribution

England, Scotland, Wales

#### Diphyus septemguttatus

(Gravenhorst, 1829)

Ichneumon septemguttatus Gravenhorst, 1829
wesmaeli (Tischbein, 1868, *Amblyteles*)
triplicatus (Thomson, 1894, *Amblyteles*)

##### Distribution

England, Ireland

#### Diphyus trifasciatus

(Gravenhorst, 1829)

Ichneumon trifasciatus Gravenhorst, 1829
triangulator (Stephens, 1835, *Ichneumon*)
daguini (Pic, 1920, *Amblyteles*)

##### Distribution

England, Scotland, Ireland

#### Eristicus

Wesmael, 1845

#### Eristicus clarigator

(Wesmael, 1845)

Ichneumon clarigator Wesmael, 1845
pachycephalus (Rudow, 1886, *Phygadeuon*)
cephalotes (Berthoumieu, 1906, *Amblyteles*)

##### Distribution

England

#### Eristicus clericus

(Gravenhorst, 1829)

Ichneumon clericus Gravenhorst, 1829
eucephalus (Wesmael, 1848, *Ichneumon*)

##### Distribution

England

#### Eupalamus

Wesmael, 1845

#### Eupalamus lacteator

(Gravenhorst, 1829)

Ichneumon lacteator Gravenhorst, 1829
fenestrator (Zetterstedt, 1838, *Ichneumon*)
depexus (Wesmael, 1845, *Ichneumon*)
albatus (Tischbein, 1879, *Ichneumon*)

##### Distribution

England

#### Eupalamus wesmaeli

(Thomson, 1886)

Ichneumon wesmaeli Thomson, 1886

##### Distribution

England

#### Eutanyacra

Cameron, 1903

#### Eutanyacra crispatoria

(Linnaeus, 1758)

Ichneumon crispatorius Linnaeus, 1758
limbatoria (Thunberg, 1824, *Ichneumon*)
rufatoria (Gravenhorst, 1829, *Ichneumon*) preocc.
nemoralis (Tischbein, 1876, *Ichneumon*) preocc.
laticincta (Rudow, 1888, *Amblyteles*)
bicuspis (Berthoumieu, 1892, *Amblyteles*)
pallidior (Pic, 1898, *Amblyteles*)

##### Distribution

England, Ireland

#### Eutanyacra glaucatoria

(Fabricius, 1793)

Ichneumon glaucatorius Fabricius, 1793
Eutanyacra glaucatoria ?*albiventris* (Gmelin, 1790, *Ichneumon*)
hungarica (Tischbein, 1868, *Amblyteles*)
sicula (Rudow, 1888, *Amblyteles*)
distyca (Berthoumieu, 1894, *Amblyteles*)
hispanica (Berthoumieu, 1896, *Amblyteles*)
spoliata (Berthoumieu, 1896, *Amblyteles*) unavailable
medinai (Berthoumieu, 1903, *Amblyteles*)
nigroscutellatus (Ulbricht, 1909, *Amblyteles*) preocc., unavailable
praetexta (Berthoumieu, 1910, *Amblyteles*)
bruyanti (Pic, 1927, *Amblyteles*)
viturati (Pic, 1927, *Amblyteles*)
krapinensis (Schmiedeknecht, 1930, *Amblyteles*)
bimaculata (Constantineanu, 1954, *Amblyteles*) preocc.

##### Distribution

England, Ireland

##### Notes

*Eutanyacra ruficornis* (Berthoumieu, 1894, *Eurylabus*) was removed from synonymy by Horstmann (2006c).

#### Eutanyacra pallidicornis

(Gravenhorst, 1829)

Ichneumon pallidicornis Gravenhorst, 1829
dimidiata (Stephens, 1835, *Ichneumon*) preocc.

##### Distribution

England, Scotland

#### Eutanyacra picta

(Schrank, 1776)

Ichneumon pictus Schrank, 1776
laboratoria (Müller, 1776, *Ichneumon*)
sanguinea (Christ, 1791, *Ichneumon*)
vadatoria (Illiger, 1807, *Ichneumon*)
affirmatoria (Thunberg, 1824, *Ichneumon*)
concinnus (Stephens, 1829, *Ichneumon*) synonymy by Horstmann (2000b)

##### Distribution

England

#### Exephanes

Wesmael, 1845

OCTATOMUS Tischbein, 1881

##### Notes

Distribution data and synonymy from Hinz and Horstmann (2000) and BMNH.

#### Exephanes fulvescens

Vollenhoven, 1875

ulbrichti Hinz, 1957

##### Distribution

England, Ireland

#### Exephanes ischioxanthus

(Gravenhorst, 1829)

Ichneumon ischioxanthus Gravenhorst, 1829
exulans (Gravenhorst, 1829, *Ichneumon*)
hilaris (Gravenhorst, 1829, *Ichneumon*)
subnudus Tischbein, 1881

##### Distribution

England, Ireland

#### Exephanes occupator

(Gravenhorst, 1829)

Ichneumon occupator Gravenhorst, 1829
contaminatus (Gravenhorst, 1829, *Ichneumon*)
munki (Kriechbaumer, 1893, *Ichneumon*) synonymy by Horstmann (2006a)
munki Kriechbaumer, 1895 preocc.
uniguttatus Kriechbaumer, 1895
unipunctatus Strobl, 1901

##### Distribution

England, Wales, Ireland

#### Exephanes riesei

(Habermehl, 1916)

Ichneumon riesei Habermehl, 1916
hoerhammeri Heinrich, 1949
amabilis Kreichbaumer, 1895 preocc.

##### Distribution

England, Ireland

##### Notes

Transferred to *Exephanes* (from *Ichneumon*) by Hinz and Horstmann (2000).

#### Exephanes venustus

(Tischbein, 1876)

Ichneumon venustus Tischbein, 1876
insidiator (Tischbein, 1876, *Ichneumon*)
caelebs Kreichbaumer, 1890

##### Distribution

Ireland

#### Gareila

Heinrich, 1980

#### Gareila tenebrosa

(Wesmael, 1845)

Ichneumon tenebrosus Wesmael, 1845
nigricornis (Schmiedeknecht, 1930, *Ichneumon*) preocc.

##### Distribution

Scotland

##### Notes

NMS, det. Riedel, added here

#### Hepiopelmus

Wesmael, 1845

EPIOPELMUS Dalla Torre, 1902

#### Hepiopelmus melanogaster

(Gmelin, 1790)

Ichneumon melanogaster Gmelin, 1790
leucostigmus (Gravenhorst, 1820, *Ichneumon*)
maculiventris (Desvignes, 1856, *Ichneumon*)
aureosericeus Taschenberg, 1866
incorruptus (Holmgren, 1871, *Amblyteles*)
palliventris (Rudow, 1888, *Amblyteles*)
annulitarsis (Pic, 1914, *Acolobus*)
maculipes Hellén, 1951

##### Distribution

England, Scotland, Wales, Ireland, Isle of Man

#### Hepiopelmus variegatorius

(Panzer, 1800)

Ichneumon variegatorius Panzer, 1800
notatorius (Panzer, 1801, *Ichneumon*) preocc.
flavoguttatus (Gravenhorst, 1829, *Ichneumon*)

##### Distribution

England, Wales, Ireland

#### Homotherus

Förster, 1869

#### Homotherus locutor

(Thunberg, 1824)

Ichneumon locutor Thunberg, 1824
labiatorius (Thunberg, 1824, *Ichneumon*)
albicinctus (Gravenhorst, 1829, *Ichneumon*)
albiceps (Hartig, 1838, *Phygadeuon*)
festinatorius (Zetterstedt, 1838, *Ichneumon*)
lautus (Tischbein, 1868, *Ichneumon*)
ruber (Kiss, 1924, *Proscus*)

##### Distribution

England, Scotland, Wales, Ireland

#### Homotherus magus

(Wesmael, 1855)

Ichneumon magus Wesmael, 1855
clavipes (Möller, 1883, *Ichneumon*) preocc.
nitidus (Bridgman, 1886, *Phaeogenes*)

##### Distribution

England, Ireland

#### Homotherus varipes

(Gravenhorst, 1829)

Ichneumon varipes Gravenhorst, 1829
costator (Donovan, 1810, *Ichneumon*) preocc., synonymy by Horstmann (2002b)
decimator (Gravenhorst, 1829, *Ichneumon*)
laevis (Ratzeburg, 1844, *Ichneumon*)
pictipes (Holmgren, 1864, *Ichneumon*)
fallax (Habermehl, 1923, *Cratichneumon*) invalid
anglicanus (Schmiedeknecht, 1928, *Ichneumon*)

##### Distribution

England, Scotland, Wales, Ireland

#### Hoplismenus

Gravenhorst, 1829

PERITAENIUS Förster, 1869
TAENIASPIS Clément, 1927

#### Hoplismenus albifrons

Gravenhorst, 1829

axillatorius misident.
Hoplismenus albifrons ?*armatorius* (Fabricius, 1787, *Ichneumon*) preocc.
albifrons Gravenhorst, 1829
perniciosus Gravenhorst, 1829
crassicornis (Rudow, 1883, *Cryptus*) preocc.
bellicosus (De Stefani, 1885, *Ichneumon*)

##### Distribution

England, Scotland, Ireland

##### Notes

Usually known as *H. axillatorius* (Thunberg) but *Ichneumon axillatorius* Thunberg, 1824 was found to be a senior synonym of *Cyclolabus pactor* (Wesmael) by Riedel (2014).

#### Hoplismenus bidentatus

(Gmelin, 1790)

Ichneumon bidentatus Gmelin, 1790
moestus Gravenhorst, 1829
maurus (Marshall, 1873, *Mesostenus*)
ichneumonoides (Rudow, 1883, *Cryptus*)
berthoumieui Pic, 1897
spinosus (Morley, 1903, *Dinotomus*)
alpinus (Clément, 1927, *Peritaenius*)
bavaricus (Clément, 1927, *Peritaenius*)

##### Distribution

England, Wales, Ireland

##### Notes

some distribution data from Whitehead (2003)

#### Hoplismenus bispinatorius

(Thunberg, 1824)

Ichneumon bispinatorius Thunberg, 1824
annulatus Berthoumieu, 1894
nigripes Seyrig, 1927
rufitarsis Constantineanu, Andriescu & Ciochia, 1956

##### Distribution

England, Scotland

##### Notes

NMS, det. Riedel, added here

#### Ichneumon

Linnaeus, 1758

BRACHYPTERUS Gravenhorst, 1829 preocc.
PTEROCORMUS Förster, 1850

##### Notes

Distribution data from Hilpert (1992), BMNH and NMS.

doubtfully placed species of *Ichneumon*:

[*femorator* Kirby, 1802 preocc., nom. dub.]

#### Ichneumon albiger

Wesmael, 1845

tempestivus Holmgren, 1864

##### Distribution

England, Scotland, Ireland

#### Ichneumon alius

Tischbein, 1879

eurycerus Thomson, 1890
dubiosus Habermehl, 1926
petrophilus Heinrich, 1951

##### Distribution

England, Scotland

#### Ichneumon alpestris

Holmgren, 1864

##### Notes

Added by Hilpert (1992); uncertain identification, based on female specimen.

#### Ichneumon analis

Gravenhorst, 1829

nigroscutellatus Habermehl, 1916 preocc.

#### Ichneumon aquilonius

Perkins, 1953

##### Distribution

England, Scotland, Ireland

#### Ichneumon bellipes

Wesmael, 1845

medialis Wesmael, 1855
divergens Holmgren, 1864
strangulator Tischbein, 1876
evanidus Berthoumieu, 1892
orbitalis Kriechbaumer, 1894 preocc., unavailable
rasnitsyni Heinrich, 1978

##### Distribution

Scotland

#### Ichneumon bucculentus

Wesmael, 1845

glaucus Tischbein, 1876
umbilicatus Valemberg, 1975

##### Distribution

England, Wales, Ireland

#### Ichneumon caloscelis

Wesmael, 1845

caloscelus Marshall, 1872
decens (Berthoumieu, 1910, *Amblyteles*)

##### Distribution

England, Ireland

#### Ichneumon cessator

Müller, 1776

custodiator Fabricius, 1793
compunctor Stephens, 1835 preocc.

##### Distribution

England, Ireland

#### Ichneumon computatorius

Müller, 1776

croceipes Wesmael, 1848
bicoloripes Tischbein, 1868
insolitus Berthoumieu, 1895 preocc., unavailable

##### Distribution

Ireland

#### Ichneumon confusor

Gravenhorst, 1820

confusorius Gravenhorst, 1829
crassicornis Tischbein, 1873 preocc.
retectus Tischbein, 1873
atronotatus Pic, 1917

##### Distribution

England, Scotland, Wales, Ireland

#### Ichneumon crassifemur

Thomson, 1886

sulphuratus Kriechbaumer, 1894 preocc.

#### Ichneumon deliratorius

Linnaeus, 1758

alternatus Schrank, 1776
Ichneumon deliratorius ?*fabricatorius* Müller, 1776
palmarius Geoffroy, 1785
inflictorius Rossi, 1792
multiannulatus Gravenhorst, 1829
delirator Zetterstedt, 1838
gmuendensis Pfeffer, 1913
schimitscheki Fahringer, 1943

##### Distribution

England, Scotland, Wales, Ireland

##### Notes

Heinrich transferred *deliratorius*, which is anomalous within *Ichneumon*, to *Coelichneumon*, which has been followed by most authors, but where *deliratorius* was also *anomalous*; Riedel (2012) transferred *deliratorius* back to *Ichneumon*, which is where, for example, Perkins (1960) classified the species; this result was supported by molecular phylogenetic evidence (Tschopp et al. 2013).

#### Ichneumon didymus

Gravenhorst, 1829

bisignatus Gravenhorst, 1829
dissimulator (Stephens, 1835, *Trogus*)
crassorius Desvignes, 1856

##### Distribution

England, Scotland, Wales

#### Ichneumon emancipatus

Wesmael, 1845

propinquus (Taschenberg, 1870, *Exephanes*)
rugosus Tischbein, 1873 preocc.
hostificus Tischbein, 1881
ramiformis Tischbein, 1881
alpinus Strobl, 1901 preocc.
vogesus Habermehl, 1916
circalpinus Heinrich, 1949

##### Distribution

England, Ireland

#### Ichneumon exilicornis

Wesmael, 1857

hircinus Holmgren, 1864
rufolineatus Holmgren, 1864
caproni Perkins, 1953

##### Distribution

England

#### Ichneumon extensorius

Linnaeus, 1758

compressus Geoffroy, 1785 preocc.
tripunctatus Geoffroy, 1785
auratus Gmelin, 1790
lusorius Gravenhorst, 1807
vexatorius Gravenhorst, 1807
retractus Tischbein, 1873
longareolatus Thomson, 1886
atropunctum Pic, 1917
quercus Pic, 1917
cassonensis Pic, 1919
luteorufus Pic, 1919
polonicus (Heinrich, 1929, *Euichneumon*)
clypeonigro Constantineanu, 1954
transitorius Constantineanu, Suciu, Andriescu & Ciochia, 1957

##### Distribution

England, Scotland, Wales, Ireland, Isle of Man

#### Ichneumon formosus

Gravenhorst, 1829

obsessor Wesmael, 1845
obessor misspelling
batis Holmgren, 1880
brunneosparsus Strobl, 1901
schachti Heinrich, 1980

##### Distribution

England, Scotland, Ireland

##### Notes

Two subspecies, *I. f. formosus* and *I. formosus microcephalus* Stephens, 1835, have been recorded from Britain (Hilpert 1992).

#### Ichneumon fuscatus

Gmelin, 1790

##### Distribution

England

##### Notes

Preoccupied by *Ichneumon fuscatus* Fabricius, 1781.

#### Ichneumon gracilentus

Wesmael, 1845

gratiosus Wesmael, 1845
vicinus Holmgren, 1864 preocc.
adscendens Tischbein, 1881
improbus Tischbein, 1881
quadrilineatus Tischbein, 1881
wuestneii Kriechbaumer, 1890
bioculatus Kriechbaumer, 1894 preocc., unavailable
trioculatus Habermehl, 1903
helveticus Habermehl, 1916 preocc.

##### Distribution

England, Scotland, Ireland

#### Ichneumon gracilicornis

Gravenhorst, 1829

iocerus Gravenhorst, 1829
quadrinotatus Stephens, 1835
propinquus Taschenberg, 1870 synonymy by Hinz and Horstmann (2000)
longisectus Berthoumieu, 1895
nigricaudus Berthoumieu, 1895 preocc., unavailable
nigroscutellatus Berthoumieu, 1895 unavailable
quadrimaculatus Habermehl, 1916
daphne Bauer, 1985

##### Distribution

England, Ireland

#### Ichneumon haemorrhoicus

Kriechbaumer, 1887

albicollis Wesmael, 1857 preocc.
nigrifemur Constantineanu, Andriescu & Ciochia, 1956 preocc.

##### Distribution

England, Wales, Ireland

#### Ichneumon ignobilis

Wesmael, 1855

filatus (Tischbein, 1879, *Amblyteles*) synonymy by Riedel (2014)
debilis (Kriechbaumer, 1886, *Amblyteles*) synonymy by Horstmann (2006a)
isenschmidii (Kriechbaumer, 1887, *Amblyteles*)
ambifarius Berthoumieu, 1904
baueri Habermehl, 1935

##### Distribution

Scotland

#### Ichneumon insidiosus

Wesmael, 1845

argali Kriechbaumer, 1882
corfitzi Thomson, 1890
jesperi Thomson, 1893 preocc.
gansuanus Kokujev, 1904
scanicus Schmiedeknecht, 1929 preocc.

##### Distribution

England, Scotland, Ireland

#### Ichneumon languidus

Wesmael, 1845

immisericors Tischbein, 1876
malignus Tischbein, 1881
nigrocastaneus Tischbein, 1881
luteoannulatus Pic, 1915

##### Distribution

England

##### Notes

Added by Hilpert (1992); one specimen (Chobham, Surrey) in BMNH identified by K. Horstmann as 'var. *immisericors* Tischbein, 1876'.

#### Ichneumon lautatorius

Desvignes, 1856

amabilis Giraud, 1863
bizonatus (Rudow, 1888, *Amblyteles*)
cingulatus Berthoumieu, 1895 unavailable
mutabilis Berthoumieu, 1895 preocc., unavailable
gynandra Habermehl, 1903
nigropunctatus Habermehl, 1903
trimaculatus Habermehl, 1903 preocc.

##### Distribution

England

#### Ichneumon ligatorius

Thunberg, 1824

gradarius Wesmael, 1848
refractarius Wesmael, 1855
velatus Wesmael, 1855
firmipes Wesmael, 1857
thulensis Ruthe, 1859
faroensis Schmiedeknecht, 1938
plautus Hilpert, 1992 synonymy by Riedel (2014)

##### Distribution

England, Scotland, Ireland

#### Ichneumon lugens

Gravenhorst, 1829

napaeus Holmgren, 1880

##### Distribution

England, Wales, Ireland

##### Notes

Transferred from *Chasmias* by Tschopp et al. (2013).

#### Ichneumon megapodius

Heinrich, 1949

nigroscutellatus Kriechbaumer, 1897 unavailable
alpinus Habermehl, 1913 preocc.
megapodiops Bauer, 1985

##### Distribution

England, Scotland

##### Notes

The British population is referable to the subspecies *fennicola* Heinrich, 1951 (Hilpert 1992, Horstmann 2006c).

#### Ichneumon melanotis

Holmgren, 1864

macrocerus Thomson, 1886
discolor Berthoumieu, 1895 preocc., unavailable
macrocerophorus Dalla Torre, 1901

##### Distribution

England, Scotland, Ireland

#### Ichneumon memorator

Wesmael, 1845

incomptus Holmgren, 1864

##### Distribution

England, Wales, Ireland

#### Ichneumon minutorius

Desvignes, 1856

guttatus Tischbein, 1873 synonymy by Horstmann (2003b)
captorius Thomson, 1887 preocc.
xanthognathus Thomson, 1887
flavipetiolatus Habermehl, 1903

##### Distribution

England, Scotland

#### Ichneumon molitorius

Linnaeus, 1761

molitor Zetterstedt, 1838
holsaticus Tischebin, 1873
intrudens Smith, 1874
croceiventris (Rudow, 1888, *Amblyteles*)
montanus Habermehl, 1903
corsicator Aubert, 1961

##### Distribution

England, Scotland, Ireland

#### Ichneumon mordax

Kriechbaumer, 1875

##### Notes

Added by Hilpert (1992); although Hilpert (1992) did not state the country of occurrence there is a specimen in NMS from Perthshire.

#### Ichneumon oblongus

Schrank, 1802

latrator misident. (Horstmann 2001a)
crassipes Gmelin, 1790, preocc.
geniculator Gravenhorst, 1807
elegans Gravenhorst, 1829
means (Gravenhorst, 1829, *Brachypterus*)

##### Distribution

England, Scotland, Ireland

#### Ichneumon primatorius

Forster, 1771

bicinctus Christ, 1791 preocc.
grossorius Fabricius, 1793
gemellitorius Thunberg, 1824
flavolineatus Gravenhorst, 1829
monetierensis Pic, 1914

##### Distribution

England, Scotland, Wales, Ireland, Isle of Man

##### Notes

Manx occurrence from Cowin and Williamson (1940)

#### Ichneumon rufidorsatus

Bridgman, 1887

##### Distribution

Scotland

#### Ichneumon sarcitorius

Linnaeus, 1758

vaginatorius Linnaeus, 1758
curvatorius Müller, 1776
bipartitus Geoffroy, 1785
flavatus Gmelin, 1790 preocc.
farctor Gravenhorst, 1807
zaydamensis Kokujev, 1909
funereus Schmiedeknecht, 1928 preocc.
niger Constantineanu, 1954 preocc.

##### Distribution

England, Scotland, Wales, Ireland, Isle of Man

#### Ichneumon sculpturatus

Holmgren, 1864

nereni Thomson, 1887
albicaudus Berthoumieu, 1895 unavailable
flavocingulatus Habermehl, 1916

##### Distribution

England

#### Ichneumon simulans

Tischbein, 1873

variolosus Holmgren, 1878
subquadratus Thomson, 1887
obscuratus Habermehl, 1916

##### Distribution

England, Scotland, Ireland

#### Ichneumon spurius

Wesmael, 1848

sieberti Habermehl, 1929

##### Distribution

England, Ireland

#### Ichneumon stigmatorius

Zetterstedt, 1838

cursorius Zetterstedt, 1838
walkeri Wesmael, 1848
rubedinis Desvignes, 1856
polyonomus Wesmael, 1859
kamtschaticus Roman, 1927
modestus Habermehl, 1935 preocc.

##### Distribution

Scotland, Ireland

#### Ichneumon stramentarius

Gravenhorst, 1820

clitellarius Holmgren, 1880
rhaeticus (Habermehl, 1917, *Dochyteles*)
scelestus Perkins, 1952 preocc.
atrifemur Perkins, 1953 preocc.
circumscriptor Valemberg, 1975
medianus Berthoumieu, 1910

##### Distribution

England, Scotland, Wales, Ireland, Isle of Man

##### Notes

According to Hilpert (1992) the subspecies *stramentarius* Grav. is found in England, *boreomaritimus* Hilpert, 1992 in Scotland and *septentrionalis* Holmgren, 1864 in England, Scotland, Wales and Ireland (additional data from Perkins 1952).

#### Ichneumon stramentor

Rasnitsyn, 1981

stramentarius misident.

##### Distribution

England, Wales

##### Notes

Added by Hilpert (1992) Perkins's (Perkins 1960) *stramentarius* is properly called *stramentor*, the name *stramentarius* actually being a senior synonym of Perkins's *septentrionalis* (with the latter name valid as a subspecies).

#### Ichneumon suspiciosus

Wesmael, 1845

mellinurus Wesmael, 1848
trispilus Thomson, 1888
rufonotatus Pic, 1929

##### Distribution

England, Scotland, Ireland, Isle of Man

##### Notes

Manx occurrence from Cowin and Williamson (1940)

#### Ichneumon terminatorius

Gravenhorst, 1820

concinnatorius Stephens, 1835
fulvoscutellatus Stephens, 1835

##### Distribution

England, Ireland

#### Ichneumon tuberculipes

Wesmael, 1848

cuneatus Tischbein, 1876
limbatus Tischbein, 1879
piceatus Tischbein, 1879
mediorufus Schmiedeknecht, 1930

##### Distribution

England, Ireland

#### Ichneumon vafer

Tischbein, 1876

conjugalis Holmgren, 1878
brevicornis Tischbein, 1881 preocc.
rogenhoferi Kriechbaumer, 1888
quartanus Perkins, 1953

##### Distribution

England, Scotland

#### Ichneumon validicornis

Holmgren, 1864

vivacior Tischbein, 1873
pseudoconfusor Heinrich, 1980

##### Distribution

England, Scotland, Ireland, Isle of Man

#### Ichneumon ventus

Hilpert, 1992

##### Distribution

Scotland

##### Notes

added by Hilpert (1992)

#### Ichneumon vulneratorius

Zetterstedt, 1838

dahlbomi Wesmael, 1857
versutus Holmgren, 1864

##### Distribution

Scotland, Ireland

#### Ichneumon xanthorius

Forster, 1771

flaviniger Gravenhorst, 1820
nassavicus (Habermehl, 1917, *Physcoteles*)
bimactulatus (Habermehl, 1917, *Physcoteles*)

##### Distribution

England, Scotland, Ireland

#### Limerodes

Wesmael, 1845

#### Limerodes arctiventris

(Schiødte, 1839)

Ichneumon arctiventris Schiødte, 1839
arctiventris (Boie, 1841, *Ichneumon*)
ophioniventris Wesmael, 1845

##### Distribution

England, Scotland, Ireland

#### Limerodops

Heinrich, 1949

OXYSOMA Kriechbaumer, 1875 preocc.

#### Limerodops elongatus

(Brischke, 1865)

Eurylabus elongatus Brischke, 1865
fluvipes (Matsumura, 1911, *Hoplismenus*)

##### Distribution

England, Scotland, Ireland

#### Limerodops subsericans

(Gravenhorst, 1820)

Ichneumon subsericans Gravenhorst, 1820
pedestrinus (Gravenhorst, 1820, *Ichneumon*)
cognatus (Stephens, 1835, *Ichneumon*)

##### Distribution

England, Scotland, Ireland, Isle of Man

#### Melanichneumon

Thomson, 1893

#### Melanichneumon leucocheilus

(Wesmael, 1845)

Ichneumon leucocheilus Wesmael, 1845
arieticornis (Berthoumieu, 1906, *Ichneumon*)

##### Distribution

England

#### Obtusodonta

Heinrich, 1962

#### Obtusodonta equitatoria

(Panzer, 1786)

Ichneumon equitatorius Panzer, 1786
antennatoria (Panzer, 1800, *Ichneumon*)
mediatoria (Fabricius, 1804, *Ichneumon*) preocc.
cingulatoria (Thunberg, 1824, *Ichneumon*) preocc.
haemorrhoidaria (Thunberg, 1824, *Ichneumon*)
rufa (De Stefani, 1885, *Amblyteles*)
nigricauda (Berthoumieu, 1896, *Amblyteles*) unavailable

#### Platylabops

Heinrich, 1950

#### Platylabops apricus

(Gravenhorst, 1820)

Ichneumon apricus Gravenhorst, 1820
intersector (Wesmael, 1854, *Amblyteles*)
semirufus (Desvignes, 1856, *Hoplismenus*) preocc.
delphinas (Berthoumieu, 1892, *Ichneumon*)
solitarius (Habermehl, 1929, *Ichneumon*) synonymy by Horstmann (2000b)

##### Distribution

England, Ireland

#### Platylabops humilis

(Wesmael, 1857)

Ichneumon humilis Wesmael, 1857
rufipes (Strobl, 1901, *Ichneumon*) preocc.

##### Distribution

England

#### Platylabops lariciatae

(Kreichbaumer, 1890)

Platylabus lariciatae Kreichbaumer, 1890
atrithorax (Berthoumieu, 1910, *Ischnogaster*)

##### Distribution

England, Scotland, Ireland

#### Platylabops speciosus

(Wesmael, 1845)

Amblyteles speciosus Wesmael, 1845
castaneusimilis Heinrich, 1930, Aoplus)

##### Distribution

Wales

##### Notes

Recorded from Wales by Perkins (1960) but not listed by Fitton (1978).

#### Platylabops virginalis

(Wesmael, 1845)

Ichneumon virginalis Wesmael, 1845
albicoxatus (Pfeffer, 1913, *Ichneumon*)

##### Distribution

England, Ireland

#### Probolus

Wesmael, 1845

##### Notes

synonymy from Horstmann (2000a)

#### Probolus crassulus

Horstmann, 2000

concinnus misident.
crassicornis (Stephens, 1835, *Ichneumon*)

##### Distribution

England, Ireland

#### Probolus culpatorius

(Linnaeus, 1758)

Ichneumon culpatorius Linnaeus, 1758
alticola (Gravenhorst, 1820, *Ichneumon*)
trucidator (Gravenhorst, 1829, *Ichneumon*)
femorator (Stephens, 1835, *Ichneumon*) preocc.
fossorius Wesmael, 1845

##### Distribution

England, Scotland, Wales

##### Notes

distribution data from Horstmann (2000a)

#### Rictichneumon

Heinrich, 1961

#### Rictichneumon pachymerus

(Hartig, 1838)

Phygadeuon pachymerus Hartig, 1838
trucidus (Wesmael, 1845, *Ichneumon*)
aciculator (Ratzeburg, 1852, *Ichneumon*)
steinii (Ratzeburg, 1852, *Ichneumon*)
septimus (Berthoumieu, 1910, *Ichneumon*)

##### Distribution

England

##### Notes

NMS, det. Riedel, added here

#### Spilichneumon

Thomson, 1894

SPILOTELES Berthoumieu, 1904
PSEUDICHNEUMON Kokujev, 1909

#### Spilichneumon ammonius

(Gravenhorst, 1820)

Ichneumon ammonius Gravenhorst, 1820
nonagriae (Holmgren, 1871, *Amblyteles*)
stagnicola (Thomson, 1888, *Amblyteles*)

##### Distribution

England, Scotland

#### Spilichneumon celenae

Perkins, 1953

##### Distribution

Scotland, Wales, Ireland

#### Spilichneumon johansoni

(Holmgren, 1871)

Amblyteles johansoni Holmgren, 1871
subalnotatus (Pic, 1914, *Amblyteles*)

##### Distribution

England, Ireland

#### Spilichneumon occisorius

(Fabricius, 1793)

Ichneumon occisorius Fabricius, 1793
sanguinatorius (Gravenhorst, 1829, *Ichneumon*)
nigrinus (Berthoumieu, 1896, *Ambyteles*) preocc., unavailable
rufinus (Berthoumieu, 1896, *Ambyteles*) preocc., unavailable
plicatus (Morley, 1903, *Ctenichneumon*)
morvandicus (Pic, 1925, *Amblyteles*)

##### Distribution

England, Scotland, Ireland, Isle of Man

#### Spilothyrateles

Heinrich, 1967

#### Spilothyrateles nuptatorius

(Fabricius, 1793)

Ichneumon nuptatorius Fabricius, 1793
fabricii (Schrank, 1802, *Ichneumon*) synonymy by Horstmann (2001a)
terminator (Panzer, 1804, *Ichneumon*)
insidiator (Fonscolombe, 1847, *Ichneumon*)
melanocerus (Wesmael, 1845, *Ichneumon*)
cubicularis (Desvignes, 1856, *Ichneumon*)
truncicola (Thomson, 1888, *Amblyteles*)
frustrator (Berthoumieu, 1892, *Amblyteles*)
paganus (Berthoumieu, 1892, *Ichneumon*)
australis (Habermehl, 1917, *Anisobas*) synonymy by Horstmann (1997)

##### Distribution

Ireland

#### Spilothyrateles punctus

(Gravenhorst, 1829)

Ichneumon punctus Gravenhorst, 1829
obscuripes (Holmgren, 1864, *Ichneumon*)
erraticus (Berthoumieu, 1892, *Ichneumon*)
nigriventris (Berthoumieu, 1895, *Ichneumon*) unavailable
lateobscurus (Pic, 1902, *Amblyteles*)
pillichi (Kiss, 1929, *Ichneumon*) synonymy by

##### Distribution

England

#### Stenaoplus

Heinrich, 1938

#### Stenaoplus pictus

(Gravenhorst, 1829)

Hoplismenus pictus Gravenhorst, 1829
rufescens (Stephens, 1835, *Ichneumon*) preocc.
ratzeburgii (Hartig, 1838, *Cryptus*)
exornatus (Wesmael, 1845, *Ichneumon*)
obscurior (Pic, 1898, *Ichneumon*) preocc.

##### Distribution

England, Scotland, Ireland

#### Stenichneumon

Thomson, 1893

#### Stenichneumon culpator

(Schrank, 1802)

Ichneumon culpator Schrank, 1802
Stenichneumon culpator ?*ani* (Geoffroy, 1785, *Ichneumon*)
ater (Berthoumieu, 1894, *Ichneumon*) preocc., unavailable
corsicator Aubert, 1960

##### Distribution

England, Scotland, Wales, Ireland, Isle of Man

#### Stenichneumon militarius

(Thunberg, 1824)

Ichneumon militarius Thunberg, 1824
pistorius (Gravenhorst, 1829, *Ichneumon*)
pistor (Zetterstedt, 1838, *Ichneumon*)
sexannularis (Berthoumieu, 1894, *Ichneumon*)

##### Distribution

England, Scotland

#### Stenobarichneumon

Heinrich, 1961

#### Stenobarichneumon basalis

(Perkins, 1960)

Barichneumon basalis Perkins, 1960

##### Distribution

England, Scotland

#### Stenobarichneumon basiglyptus

(Kriechbaumer, 1890)

Ichneumon basiglyptus Kriechbaumer, 1890
bifossatus (Berthoumieu, 1892, *Ichneumon*)
coxiglyptus (Heinrich, 1951, *Barichneumon*)

##### Distribution

England, Ireland

#### Stenobarichneumon citator

(Thunberg, 1824)

Ichneumon citator Thunberg, 1824
incubitor misident.

##### Distribution

England

#### Sycaonia

Cameron, 1903

#### Sycaonia foersteri

(Wesmael, 1848)

Ichneumon foersteri Wesmael, 1848
boreosicaria (Roman, 1913, *Cratichneumon*)

##### Distribution

England, Scotland, Ireland

#### Thyrateles

Perkins, 1953

#### Thyrateles camelinus

(Wesmael, 1845)

Amblyteles camelinus Wesmael, 1845
certator (Müller, 1776, *Ichneumon*) nom. ob., synonymy by Horstmann (2001a)
cardui (Schrank, 1786, *Ichneumon*) nom. ob., synonymy by Horstmann (2001a)
adustus (Gmelin, 1790, *Ichneumon*) nom. ob., synonymy by Horstmann (2001a)
malignus (Tischbein, 1868, *Amblyteles*)
brunnipes (Tischbein, 1879, *Ichneumon*)
rufomaculatus (Kriechbaumer, 1894, *Amblyteles*) unavailable
alticola (Habermehl, 1920, *Ichneumon*) preocc.
oisanensis (Pic, 1927, *Ichneumon*)

##### Distribution

England, Scotland

#### Thyrateles haereticus

(Wesmael, 1854)

Amblyteles haereticus Wesmael, 1854
urticarum (Holmgren, 1880, *Ichneumon*)
binotatus (Kriechbaumer, 1894, *Amblyteles*) preocc., unavailable
Thyrateles haereticus ?*cinctor* (Kriechbaumer, 1894, *Amblyteles*) preocc.
pyraeneus (Pic, 1914, *Ichneumon*) preocc.

#### Tricholabus

Thomson, 1894

#### Tricholabus strigatorius

(Gravenhorst, 1829)

Ichneumon strigatorius Gravenhorst, 1829
quittardi (Pic, 1904, *Amblyteles*)
berthoumieui Pic, 1927

##### Distribution

England, Scotland, Ireland, Isle of Man

#### Triptognathus

Berthoumieu, 1904

#### Triptognathus atripes

(Gravenhorst, 1820)

Ichneumon atripes Gravenhorst, 1820
goedarti (Gravenhorst, 1829, *Ichneumon*)
interruptus (Gravenhorst, 1829, *Ichneumon*)
pratensis (Gravenhorst, 1829, *Ichneumon*)
quadricingulatus (Gravenhorst, 1829, *Ichneumon*)
uniguttatus (Gravenhorst, 1829, *Ichneumon*)
ignotus (Fonscolombe, 1847, *Ichneumon*)
praedator (Fonscolombe, 1847, *Ichneumon*) preocc.
flavifemur (Tischbein, 1873, *Ichneumon*)
interjectus (Tischbein, 1879, *Amblyteles*)
subfasciatus (Tischbein, 1879, *Amblyteles*)
stephani (Pic, 1903, *Amblyteles*)
taiyudongus (Uchida, 1926, *Spilichneumon*)

##### Distribution

England

##### Notes

NMS (from Santon Downham), det. Riedel, added here; tentative identification as the genus is in need of revision, but certainly in the *atripes* group (M. Riedel, pers. comm.)

#### Triptognathus sibilans

(Gravenhorst, 1829)

Ichneumon sibilans Gravenhorst, 1829
propinquus (Perkins, 1953, *Amblyteles*)

##### Distribution

England

#### Virgichneumon

Heinrich, 1977

#### Virgichneumon albilineatus

(Gravenhorst, 1820)

Ichneumon albilineatus Gravenhorst, 1820
albolineatus misspelling
leucomelas (Gmelin, 1790, *Ichneumon*) preocc.
nigratorius (Panzer, 1804, *Ichneumon*) preocc.
Virgichneumon albilineatus ?*bilineator* (Donovan, 1810, *Ichneumon*)
bipunctorius (Stephens, 1835, *Ichneumon*) preocc.

##### Distribution

England

#### Virgichneumon albosignatus

(Gravenhorst, 1829)

Ichneumon albosignatus Gravenhorst, 1829
mesostilpnus (Thomson, 1888, *Ichneumon*)
punctus (Berthoumieu, 1895, *Ichneumon*) preocc., unavailable
nigricollis (Constantineanu, 1954, *Melanichneumon*)

##### Distribution

England

#### Virgichneumon callicerus

(Gravenhorst, 1820)

Ichneumon callicerus Gravenhorst, 1820
plurialbatus (Wesmael, 1855, *Ichneumon*)
eremita (Kokujev, 1909, *Ichneumon*)

##### Distribution

England

#### Virgichneumon digrammus

(Gravenhorst, 1820)

Ichneumon digrammus Gravenhorst, 1820
nudicoxa (Thomson, 1888, *Ichneumon*)
balearicus (Kriechbaumer, 1894, *Ichneumon*)

##### Distribution

England, Ireland

#### Virgichneumon dumeticola

(Gravenhorst, 1829)

Ichneumon dumeticola Gravenhorst, 1829

##### Distribution

England

#### Virgichneumon faunus

(Gravenhorst, 1829)

Ichneumon faunus Gravenhorst, 1829
leucopygus (Gravenhorst, 1829, *Ichneumon*)

##### Distribution

England, Ireland

#### Virgichneumon maculicauda

(Perkins, 1953)

Barichneumon maculicauda Perkins, 1953
perscrutator (Wesmael, 1845, *Ichneumon*) preocc.

##### Distribution

England, Scotland, Ireland

#### Virgichneumon monostagon

(Gravenhorst, 1820)

Ichneumon monostagon Gravenhorst, 1820
luctuosus (Gravenhorst, 1820, *Ichneumon*)
indagator (Wesmael, 1845, *Ichneumon*) preocc.
redimitus (Tischbein, 1874, *Ichneumon*)
explorator (Tischbein, 1876, *Ichneumon*)
minor (Kriechbaumer, 1894, *Ichneumon*) preocc., unavailable
hexaleucus (Kriechbaumer, 1899, *Ichneumon*) synonymy by Horstmann (2006a)
annulicornis (Schmiedeknecht, 1928, *Ichneumon*) preocc.

##### Distribution

England, Ireland

#### Virgichneumon tergenus

(Gravenhorst, 1820)

Ichneumon tergenus Gravenhorst, 1820
octoguttatus (Gravenhorst, 1829, *Ichneumon*)

##### Distribution

England, Wales

#### Vulgichneumon

Heinrich, 1961

#### Vulgichneumon bimaculatus

(Schrank, 1776)

Ichneumon bimaculatus Schrank, 1776
bimaculatorius (Panzer, 1801, *Ichneumon*)

##### Distribution

England, Ireland

#### Vulgichneumon deceptor

(Scopoli, 1763)

Ichneumon deceptor Scopoli, 1763
deceptorius (Thunberg, 1824, *Ichneumon*)
deceptorius (Zetterstedt, 1838, *Ichneumon*) preocc.
vestigator (Wesmael, 1845, *Ichneumon*) preocc.
completus (Berthoumieu, 1894, *Ichneumon*)
obscurior (Berthoumieu, 1895, *Ichneumon*) unavailable

##### Distribution

England, Ireland

#### Vulgichneumon saturatorius

(Linnaeus, 1758)

Ichneumon saturatorius Linnaeus, 1758
nigratorius (Pontoppidan, 1763, *Ichneumon*)
carnifex (Müller, 1776, *Ichneumon*)
clavatorius (Müller, 1776, *Ichneumon*)
fuscocastaneus (Gravenhorst, 1829, *Ichneumon*)
saturator (Zetterstedt, 1838, *Ichneumon*)
albotrochanteratus (Ulbricht, 1926, *Ichneumon*)

##### Distribution

England, Scotland, Wales, Ireland, Isle of Man

#### Vulgichneumon suavis

(Gravenhorst, 1820)

Ichneumon suavis Gravenhorst, 1820
fallax (Gravenhorst, 1829, *Ichneumon*)
lepidus (Gravenhorst, 1829, *Ichneumon*)

##### Distribution

England, Scotland, Wales, Ireland

#### LISTRODROMINI

Förster, 1869

#### Anisobas

Wesmael, 1845

LYCAENIPHILOS Heinrich, 1934

#### Anisobas cingulatellus

Horstmann, 1997

cingulatorius (Gravenhorst, 1820, *Ichneumon*) preocc.

##### Distribution

England, Wales

##### Notes

Replacement name for *Ichneumon cingulatorius* Gravenhorst, 1820, preoccupied by *cingulatorius* Weber, 1801. Listed as a subspecies of *australis* Habermehl, 1917, in Yu and Horstmann (1997) but the latter is a junior synonym of *Spilothyrateles nuptatorius* (Horstmann 1997).

#### Anisobas platystylus

Thomson, 1888

##### Distribution

England

#### Listrodromus

Wesmael, 1845

#### Listrodromus nycthemerus

(Gravenhorst, 1820)

Ichneumon nycthemerus Gravenhorst, 1820
quinqueguttatus (Gravenhorst, 1829, *Ichneumon*)

##### Distribution

England, Ireland

#### Neotypus

Förster, 1869

#### Neotypus nobilitator

(Gravenhorst, 1807)

Ichneumon nobilitator Gravenhorst, 1807
erythronotus (Rudow, 1882, *Cryptus*)

##### Distribution

England

#### OEDICEPHALINI

Heinrich, 1934

NOTOSEMINI Townes, 1961

#### Notosemus

Förster, 1869

ISCHNIDIUM Kriechbaumer, 1890
ISCHNOGASTER Kriechbaumer, 1890 preocc.

#### Notosemus bohemani

(Wesmael, 1855)

Phaeogenes bohemani Wesmael, 1855
dives Brischke, 1887
albibucca (Kriechbaumer, 1890, *Ischnogaster*)
gaullei (Berthoumieu, 1900, *Ischnus*)
atriventris (Pic, 1915, *Ischnogaster*)

##### Distribution

England

#### PHAEOGENINI

Förster, 1869

##### Notes

Wahl and Mason (1995) included *Alomya* and relatives in this tribe, with the tribe taking the name Alomyini. With the removal of *Alomya* to a separate subfamily (see notes under Alomyinae) the tribe takes the name Phaeogenini. Some distribution data from Diller and Shaw (2014).

#### Aethecerus

Wesmael, 1845

#### Aethecerus discolor

Wesmael, 1845

styriacus Strobl, 1901

##### Distribution

England, Scotland, Ireland

#### Aethecerus dispar

Wesmael, 1845

frontatus Wesmael, 1845
albipictus Berthoumieu, 1897 unavailable
rufipes Strobl, 1901

##### Distribution

England, Scotland

#### Aethecerus foveolatus

Gregor, 1940

exilis (Berthoumieu, 1899, *Diadromus*) preocc.

##### Distribution

England, Scotland

##### Notes

added by Diller and Shaw (2014)

#### Aethecerus horstmanni

Diller & Shaw, 2014

##### Distribution

England, Scotland

##### Notes

added by Diller and Shaw (2014)

#### Aethecerus longulus

Wesmael, 1845

formosus (Bridgman, 1881, *Phaeogenes*)

##### Distribution

England

#### Aethecerus nitidus

Wesmael, 1845

corcyriensis (Berthoumieu, 1901, *Phaeogenes*)

##### Distribution

England, Scotland

#### Aethecerus placidus

Wesmael, 1845

nigricoxatus Strobl, 1901

##### Distribution

England, Scotland, Ireland

#### Aethecerus porcellus

Holmgren, 1890

##### Distribution

England, Scotland, Wales

##### Notes

added by Diller and Shaw (2014)

#### Aethecerus ruberpedatus

Diller & Shaw, 2014E

##### Distribution

England, Scotland

##### Notes

added by Diller and Shaw (2014)

#### Aethecerus rugifrons

Holmgren, 1890

##### Distribution

England, Scotland

##### Notes

added by Diller and Shaw (2014)

#### Aethecerus subuliferus

(Holmgren, 1890)

Phaeogenes subuliferus Holmgren, 1890

##### Distribution

England

##### Notes

Added by Diller and Shaw (2014); a species of *Aethecerus*, not *Phaeogenes*, where it is listed by Yu et al. (2012) (Diller & Shaw, 2014Diller and Shaw 2014).

#### Baeosemus

Förster, 1869

#### Baeosemus mitigosus

(Gravenhorst, 1829)

Ichneumon mitigosus Gravenhorst, 1829
phaeocerus (Wesmael, 1845, *Herpestomus*)
vulpecula Holmgren, 1890

##### Distribution

England, Scotland

##### Notes

added by Diller and Shaw (2014)

#### Centeterus

Wesmael, 1845

#### Centeterus confector

(Gravenhorst, 1829)

Ichneumon confector Gravenhorst, 1829
picticollis Wesmael, 1845
nigridentis Constantineanu, 1951

##### Distribution

England, Ireland

#### Centeterus rubiginosus

(Gmelin, 1790)

Ichneumon rubiginosus Gmelin, 1790
opprimator (Gravenhorst, 1820, *Ichneumon*)
rufipes (Brischke, 1891, *Phaeogenes*)

##### Distribution

England, Scotland, Wales, Ireland, Isle of Man

#### Colpognathus

Wesmael, 1845

#### Colpognathus celerator

(Gravenhorst, 1807)

Ichneumon celerator Gravenhorst, 1807
procerus (Gravenhorst, 1829, *Phygadeuon*)
femorator (Stephens, 1835, *Ichneumon*) preocc.
celeratorius (Zetterstedt, 1838, *Ichneumon*)
armatus Thomson, 1891 synonymy by Diller and Schönitzer (2003)
atricornis Pic, 1914 synonymy by Diller and Schönitzer (2003)
femoralis Habermehl, 1917
petiolaris Constantineanu, 1954
nigroscaposus Aubert, 1959

##### Distribution

England, Scotland, Wales, Ireland, Isle of Man

##### Notes

Manx occurrence from Shaw and Bennett (2001)

#### Colpognathus divisus

Thomson, 1891

atricornis Pic, 1914
rufifemur Constantineanu, 1959

##### Distribution

England, Scotland, Wales, Ireland

#### Diadromus

Wesmael, 1845

THYRAEELLA Holmgren, 1890

#### Diadromus albinotatus

(Gravenhorst, 1829)

Ichneumon albinotatus Gravenhorst, 1829

##### Distribution

England

#### Diadromus arrisor

Wesmael, 1845

##### Distribution

England

##### Notes

BMNH, Ely coll., det. Diller, added here

#### Diadromus candidatus

(Gravenhorst, 1829)

Ichneumon candidatus Gravenhorst, 1829
guttulatus (Gravenhorst, 1829, *Ichneumon*)
aries (Brischke, 1887, *Phaegoenes*)
decolor Holmgren, 1890

#### Diadromus collaris

(Gravenhorst, 1829)

Ischnus collaris Gravenhorst, 1829
similis (Bridgman, 1881, *Phaeogenes*)
bellulus (Kriechbaumer, 1894, *Phaeogenes*)
brischkei Berthoumieu, 1897 unavailable
punicus Berthoumieu, 1898
rufiscapus Pic, 1902
cabrerai Berthoumieu, 1903
hispanicus (Berthoumieu,1904, *Heterischnus*)
brevicauda (Hellén, 1949, *Ischnopsidea*)

##### Distribution

England, Scotland

#### Diadromus heteroneurus

Holmgren, 1890

quadriguttatus misident.
prosopius Holmgren, 1890
nigroscutellatus Constantineanu, Suciu, Andriescu & Ciochia, 1957

##### Distribution

England, Scotland

#### Diadromus nitidigaster

Diller & Shaw, 2014

##### Distribution

England

##### Notes

added by Diller and Shaw (2014)

#### Diadromus pulchellus

Wesmael, 1845

##### Distribution

England

##### Notes

added by Diller and Shaw (2014)

#### Diadromus subtilicornis

(Gravenhorst, 1829)

Ichneumon subtilicornis Gravenhorst, 1829
imbellis Wesmael, 1845
dolosus Berthoumieu, 1899
nigrinus (Berthoumieu, 1901, *Phaeogenes*)

##### Distribution

England, Scotland

#### Diadromus tenax

Wesmael, 1845

##### Distribution

England

#### Diadromus troglodytes

(Gravenhorst, 1829)

Ichneumon troglodytes Gravenhorst, 1829
abdominator (Stephens, 1835, *Ichneumon*)
scobinatus Holmgren, 1890

##### Distribution

England, Scotland, Wales, Ireland

#### Diadromus varicolor

Wesmael, 1845

##### Distribution

England, Scotland, Wales, Isle of Man

#### Dicaelotus

Wesmael, 1845

DELOGLYPTUS Förster, 1869
HOLOCREPIS Förster, 1869
LEPTODEMAS Förster, 1869
CINXAELOTUS Holmgren, 1890
EURYPTILUS Holmgren, 1890

#### Dicaelotus cameroni

Bridgman, 1881

minutulus Kokujev, 1909
nigroclypeatus Constantineanu, 1959

##### Distribution

England, Scotland

#### Dicaelotus erythrostoma

Wesmael, 1845

##### Distribution

England, Ireland

#### Dicaelotus inflexus

Thomson, 1891

##### Distribution

England, Scotland, Wales, Ireland

#### Dicaelotus orbitalis

Thomson, 1891

##### Distribution

England, Ireland

#### Dicaelotus parvulus

(Gravenhorst, 1829)

Ichneumon parvulus Gravenhorst, 1829
alpigenus Strobl, 1901

##### Distribution

England

#### Dicaelotus pictus

(Schmiedeknecht, 1903)

Deloglyptus pictus Schmiedeknecht, 1903
lugens Berthoumieu, 1906

##### Distribution

England

#### Dicaelotus pudibundus

(Wesmael, 1845)

Herpestomus pudibundus Wesmael, 1845
alboscutatus Berthoumieu, 1900
gaullei Berthoumieu, 1903

##### Distribution

England

#### Dicaelotus pumilus

(Gravenhorst, 1829)

Ichneumon pumilus Gravenhorst, 1829
morosus Wesmael, 1855
analis Berthoumieu, 1901 unavailable

##### Distribution

England, Scotland, Wales, Ireland

#### Dicaelotus punctiventris

(Thomson, 1891)

Deloglyptus punctiventris Thomson, 1891
punicus Berthoumieu, 1901 unavailable

##### Distribution

England, Scotland, Ireland

#### Dicaelotus pusillator

(Gravenhorst, 1807)

Ichneumon pusillator Gravenhorst, 1807
Dicaelotus pusillator ?*pallidus* (Gmelin, 1790, *Ichneumon*)
notator (Gravenhorst, 1807, *Ichneumon*)

##### Distribution

Scotland

##### Notes

added by Diller and Shaw (2014)

#### Dicaelotus pusillus

Holmgren, 1890

##### Distribution

England

##### Notes

added by Diller and Shaw (2014)

#### Dicaelotus resplendens

Holmgren, 1890

fitchi Perkins, 1953

##### Distribution

England, Ireland

#### Dicaelotus ruficoxatus

(Gravenhorst, 1829)

Ichneumon ruficoxatus Gravenhorst, 1829
unipunctatus Wesmael, 1845
nigrescens Constantineanu, 1959

##### Distribution

England, Scotland, Ireland

#### Dicaelotus rufoniger

Berthoumieu, 1897

##### Distribution

England, Scotland, Wales, Ireland

#### Dicaelotus schmiedeknechti

Diller & Shaw, 2014

ruficornis (Schmiedeknecht, 1903, *Eparces*) preocc.

##### Distribution

England

##### Notes

added by Diller and Shaw (2014)

#### Dicaelotus suspectus

Perkins, 1953

##### Distribution

England

#### Dilleritomus

Aubert, 1979

#### Dilleritomus apertor

Aubert, 1979

##### Distribution

England

##### Notes

added by Diller and Shaw (2014)

#### Dilleritomus filiformis

(Strobl, 1901)

Herpestomus filiformis Strobl, 1901

##### Distribution

England, Wales

##### Notes

added by Diller and Shaw (2014)

#### Dirophanes

Förster, 1869

#### Dirophanes callopus

(Wesmael, 1845)

Phaeogenes callopus Wesmael, 1845
tibiator (Thunberg, 1824, *Ichneumon*) preocc.
palliditarsis (Berthoumieu, 1900, *Diadromus*)

##### Distribution

England, Scotland

#### Dirophanes foveolatus

(Perkins, 1953)

Phaeogenes foveolatus Perkins, 1953

##### Distribution

England, Scotland, Ireland

##### Notes

Transferred from *Phaeogenes* by Schönitzer et al. (2006).

#### Dirophanes fulvitarsis

(Wesmael, 1845)

Phaeogenes fulvitarsis Wesmael, 1845
limatus (Wesmael, 1845, *Phaeogenes*)
hyperboreus (Holmgren, 1890, *Phaeogenes*)
nitidiventris (Holmgren, 1890, *Phaeogenes*)
ruficoxa (Thomson, 1891, *Phaeogenes*)

##### Distribution

England, Scotland, Ireland, Isle of Man

#### Dirophanes invisor

(Thunberg, 1824)

Ichneumon invisor Thunberg, 1824
stimulator (Gravenhorst, 1829, *Ichneumon*)
homochlorus (Wesmael, 1845, *Phaeogenes*)
kabylianus (Pic, 1897, *Phaeogenes*)

##### Distribution

England, Scotland, Wales

#### Dirophanes maculicornis

(Stephens, 1835)

Ichneumon maculicornis Stephens, 1835
scutellaris (Wesmael, 1845, *Phaeogenes*)
bisignatus (Holmgren, 1890, *Phaeogenes*)
dentatus (Pic, 1923, *Phaeogenes*)
nigroscutellatus (Habermehl, 1929, *Phaeogenes*)
murinanae (Fahringer, 1936, *Microcryptus*)
gigas (Fahringer, 1943, *Phaeogenes*)
dinianae (Fahringer, 1948, *Phaeogenes*)
ruficoxis (Constantineanu, 1959, *Phaeogenes*) preocc.

##### Distribution

England, Scotland

#### Dirophanes mysticus

(Wesmael, 1855)

Phaeogenes mysticus Wesmael, 1855
tetricus Wesmael, 1855

##### Distribution

England, Scotland

##### Notes

Transferred from *Phaeogenes* by Schönitzer et al. (2006).

#### Dirophanes regenerator

(Fabricius, 1804)

Cryptus regenerator Fabricius, 1804
rusticatus (Wesmael, 1845, *Phaeogenes*) synonymy by Horstmann (2001a)

##### Distribution

England, Scotland, Wales, Ireland, Isle of Man

#### Eparces

Förster, 1869

#### Eparces grandiceps

(Thomson, 1891)

Centeterus grandiceps Thomson, 1891

##### Distribution

England

#### Epitomus

Förster, 1869

#### Epitomus infuscatus

(Gravenhorst, 1829)

Hemiteles infuscatus Gravenhorst, 1829
pygmaeus (Brischke, 1890, *Hemiteles*) preocc.
parvus Thomson, 1891
laeviareolatus Schmiedeknecht, 1904

##### Distribution

England, Scotland, Wales, Ireland, Isle of Man

#### Epitomus proximus

Perkins, 1953

##### Distribution

England, Scotland, Wales, Ireland

#### Eriplatys

Förster, 1869

ANOPIESTA Förster, 1869
MELANOMICRUS Morley, 1903

#### Eriplatys ardeicollis

(Wesmael, 1845)

Herpestomus ardeicollis Wesmael, 1845
elliotti (Morley, 1903, *Melanomicrus*)
neirae (Ceballos, 1958, *Herpestomus*)

##### Distribution

England, Scotland, Ireland

#### Eriplatys sawoniewiczi

Diller, 1993

##### Distribution

England, Scotland

##### Notes

added by Diller and Shaw (2014)

#### Hemichneumon

Wesmael, 1857

#### Hemichneumon subdolus

Wesmael, 1857

elongatus (Ratzeburg, 1852, *Hemiteles*) preocc.
suspectus Wesmael, 1857
varians (Taschenberg, 1865, *Cryptus*) synonymy by Schwarz (2005)
tineidarum (Giraud, 1872, *Ischnus*)

##### Distribution

England, Ireland

#### Herpestomus

Wesmael, 1845

#### Herpestomus arridens

(Gravenhorst, 1829)

Ichneumon arridens Gravenhorst, 1829
facialis (Gravenhorst, 1829, *Ichneumon*)
xanthops (Gravenhorst, 1829, *Ichneumon*)
erubescens (Berthoumieu, 1899, *Diadromus*)
subatriceps (Pic, 1914, *Phaeogenes*)
transsylvanicus Kiss, 1924
rufifrons (Aerts, 1957, *Rhexidermus*)
meridionator Aubert, 1960 synonymy by Diller and Tereshkin (2005)

##### Distribution

England, Scotland, Wales

#### Herpestomus brunnicornis

(Gravenhorst, 1829)

Ichneumon brunnicornis Gravenhorst, 1829
padella (Goureau, 1847, *Ichneumon*)
bruneicornis Dalla Torre, 1902
bisignatus Habermehl, 1917
nigriventris Constantineanu, 1944

##### Distribution

England, Scotland, Ireland

#### Herpestomus minimus

(Berthoumieu, 1901)

Phaeogenes minimus Berthoumieu, 1901

##### Distribution

England, Scotland

##### Notes

Added by Diller and Shaw (2014); previously recorded as British by Carr (1924) but these records are now discounted (Perkins 1953, Shaw 2003).

#### Herpestomus nasutus

Wesmael, 1845

furunculus Wesmael, 1845
intermedius Wesmael, 1845

##### Distribution

England, Scotland, Ireland

#### Herpestomus wesmaeli

Perkins, 1953

##### Distribution

England, Scotland, Ireland

#### Heterischnus

Wesmael, 1859

RHEXIDERMUS Förster, 1869
ISCHNOPSIDEA Viereck, 1914

#### Heterischnus nigricollis

(Wesmael, 1845)

Ischnus nigricollis Wesmael, 1845
rufipes (Wesmael, 1848, *Ischnus*)

##### Distribution

England, Scotland, Wales

#### Heterischnus pulex

(Müller, 1776)

Ichneumon pulex Müller, 1776
murex (Müller, 1776, *Ichneumon*)
brevicornis (Gravenhorst, 1829, *Ichneumon*)

#### Heterischnus truncator

(Fabricius, 1798)

Ichneumon truncator Fabricius, 1798
Heterischnus truncator ?*colorator* (Villers, 1789, *Ichneumon*)
filiformis (Gravenhorst, 1829, *Ischnus*)
thoracicus (Gravenhorst, 1829, *Ischnus*) synonymy by Horstmann (2001a)
elegans (Tischbein, 1868, *Ischnus*)
montanus (Berthoumieu, 1897, *Ischnus*)
moravicus (Gregor, 1939, *Ischnus*) synonymy by Horstmann (2002b)
nigrinus (Constantineanu, 1959, *Ischnus*) preocc.

##### Distribution

England, Scotland

#### Mevesia

Holmgren, 1890

#### Mevesia arguta

(Wesmael, 1845)

Phaeogenes argutus Wesmael, 1845
tenuis (Berthoumieu, 1899, *Phaeogenes*)
albifemur Constantineanu, 1959

##### Distribution

England, Scotland, Wales, Ireland

#### Mevesia guttata

Perkins, 1953

##### Distribution

England

#### Misetus

Wesmael, 1845

#### Misetus oculatus

Wesmael, 1845

obscurus Berthoumieu, 1897 unavailable

##### Distribution

England, Scotland, Ireland

#### Nematomicrus

Wesmael, 1845

#### Nematomicrus tenellus

Wesmael, 1845

##### Distribution

England, Scotland, Ireland

#### Oiorhinus

Wesmael, 1845

#### Oiorhinus pallipalpis

Wesmael, 1845

striatus (Bridgman, 1881, *Herpestomus*)
pallidipalpis Dalla Torre, 1902

##### Distribution

England, Scotland, Wales, Ireland

##### Notes

some distribution data from Shaw (1984)

#### Oronotus

Wesmael, 1845

ORONTUS misspelling

#### Oronotus binotatus

(Gravenhorst, 1829)

Phygadeuon binotatus Gravenhorst, 1829
coarctatus Wesmael, 1845

##### Distribution

Ireland

#### Orotylus

Holmgren, 1890

#### Orotylus mitis

(Wesmael, 1848)

Diadromus mitis Wesmael, 1848

##### Distribution

England

##### Notes

The only British specimens seen by Perkins (1959) were from the Bridgman collection, apparently lacking locality data. There are recent specimens in BMNH and NMS.

#### Paraethecerus

Perkins, 1953

#### Paraethecerus elongatus

Perkins, 1953

##### Distribution

England

#### Phaeogenes

Wesmael, 1845

##### Notes

Many species previously placed in the genus *Phaeogenes* (Perkins 1959, Fitton 1978) are now classified in *Dirophanes* and *Tycherus*.

doubtfully placed species of *Phaeogenes*:

[*picipes* (Stephens, 1835, *Ichneumon*) nom. dub., from England]

#### Phaeogenes curator

(Thunberg, 1824)

Ichneumon curator Thunberg, 1824
crassidens Thomson, 1891

##### Distribution

England, Wales

##### Notes

some distribution data from Ely (2002)

#### Phaeogenes distinctus

(Bridgman, 1887)

Herpestomus distinctus Bridgman, 1887

##### Distribution

England

#### Phaeogenes heterogonus

Holmgren, 1890

##### Distribution

Wales, Ireland

#### Phaeogenes melanogonos

(Gmelin, 1790)

Ichneumon melanogonos Gmelin, 1790
protervus Wesmael, 1855
nigripes Constantineanu, 1954

##### Distribution

England, Scotland

#### Phaeogenes nigridens

(Wesmael, 1845)

Phaeogenes nigridens Wesmael, 1845
major (Berthoumieu, 1901, *Phaeogenes*)

##### Distribution

England

##### Notes

Added by Ely (2002); transferred back to *Phaeogenes* (from *Tycherus*) by Diller and Shaw (2014).

#### Phaeogenes planifrons

Wesmael, 1845

compar Berthoumieu, 1904
hungaricus (Kiss, 1926, *Orotylus*) synonymy by

##### Distribution

Ireland

#### Phaeogenes semivulpinus

(Gravenhorst, 1829)

Ichneumon semivulpinus Gravenhorst, 1829
mutabilis (Gravenhorst, 1829, *Ichneumon*)
rufator (Stephens, 1835, *Ichneumon*) preocc.
primarius Wesmael, 1845

##### Distribution

England, Scotland

#### Phaeogenes trepidus

Wesmael, 1845

##### Distribution

England

#### Stenodontus

Berthoumieu, 1897

GNATHOXYS Wesmael, 1845

#### Stenodontus marginellus

(Gravenhorst, 1829)

Ichneumon marginellus Gravenhorst, 1829
albicoxis Habermehl, 1917

##### Distribution

England, Scotland, Wales, Ireland

#### Trachyarus

Thomson, 1891

##### Notes

Gokhman (2007) states that a series of males from Hampshire, in BMNH, probably belong to *T. prominulus* Diller, 1989. I have also seen a female from the site and cannot differentiate these specimens from others that V. Gokhman has identified as *corvinus*.

#### Trachyarus corvinus

Thomson, 1891

atratus (Berthoumieu, 1901, *Phaeogenes*)

##### Distribution

England

#### Tycherus

Förster, 1869

MICROPE Förster, 1869
PROSCUS Holmgren, 1890
MICROPA Schulz, 1906
GLYPTICHNEUMON Habermehl, 1917

#### Tycherus amaenus

(Wesmael, 1845)

Phaeogenes amaenus Wesmael, 1845
flavoclypeatus (Strobl, 1901, *Phaeogenes*)

##### Distribution

Wales

##### Notes

Added by Diller and Shaw (2014); transferred from *Phaeogenes* by Sebald et al. (2000).

#### Tycherus bellicornis

(Wesmael, 1845)

Phaeogenes bellicornis Wesmael, 1845
rugulosus (Constantineanu, 1959, *Phaeogenes*)

##### Distribution

England, Scotland, Wales, Ireland

#### Tycherus brunneus

(Kiss, 1924)

Eriplatys brunneus Kiss, 1924

##### Distribution

England

##### Notes

added by Diller and Shaw (2014)

#### Tycherus capitosus

(Holmgren, 1890)

Phaeogenes capitosus Holmgren, 1890

##### Distribution

Scotland

##### Notes

added by Diller and Shaw (2014)

#### Tycherus cephalotes

(Wesmael, 1845)

Phaeogenes cephalotes Wesmael, 1845

##### Distribution

England

#### Tycherus coriaceus

(Perkins, 1953)

Phaeogenes coriaceus Perkins, 1953

##### Distribution

England, Scotland

#### Tycherus dodecellae

Ranin, 1983

##### Distribution

Scotland

##### Notes

added by Diller and Shaw (2014)

#### Tycherus elongatus

(Thomson, 1891)

Phaeogenes elongatus Thomson, 1891

##### Distribution

England

#### Tycherus eques

(Wesmael, 1845)

Phaeogenes eques Wesmael, 1845

##### Distribution

England, Scotland

#### Tycherus flavidens

(Wesmael, 1845)

Phaeogenes flavidens Wesmael, 1845
flavoclypeatus (Strobl, 1901, *Herpestomus*)

##### Distribution

England

#### Tycherus fuscibucca

(Berthoumieu, 1901)

Ischnogaster fuscibucca Berthoumieu, 1901
kratochvili (Gregor, 1943, *Eriplatys*)

##### Distribution

Scotland

##### Notes

added by Diller and Shaw (2014)

#### Tycherus fuscicornis

(Wesmael, 1845)

Phaeogenes fuscicornis Wesmael, 1845
phaeogenoides (Habermehl, 1917, *Glyptichneumon*)

##### Distribution

England, Scotland, Ireland

#### Tycherus helleni

Ranin, 1983

##### Distribution

Scotland

##### Notes

added by Diller and Shaw (2014)

#### Tycherus histrio

(Wesmael, 1848)

Phaeogenes histrio Wesmael, 1848

##### Notes

Removed from synonymy with *ischiomelinus* by Diller and Shaw (2014); only British specimen lacking locality data.

#### Tycherus impiger

(Wesmael, 1845)

Phaeogenes impiger Wesmael, 1845
ruficoxis (Constantineanu, 1951, *Phaeogenes*)

##### Distribution

England, Scotland

##### Notes

Transferred from *Phaeogenes* by Bauer (2001).

#### Tycherus improcerus

Ranin, 1983

##### Distribution

England

##### Notes

added by Diller and Shaw (2014)

#### Tycherus infimus

(Wesmael, 1845)

Phaeogenes infimus Wesmael, 1845
minutus (Wesmael, 1845, *Phaeogenes*)

##### Distribution

England, Scotland, Ireland

#### Tycherus ischiomelinus

(Gravenhorst, 1829)

Ichneumon ischiomelinus Gravenhorst, 1829
eximius (Wesmael, 1845, *Phaeogenes*)

##### Distribution

England, Scotland, Ireland

#### Tycherus jucundus

(Wesmael, 1845)

Phaeogenes jucundus Wesmael, 1845

##### Distribution

England

##### Notes

BMNH, added here; transferred from *Phaeogenes* by Diller and Schönitzer (2003). Has also been treated as a species of *Colpognathus*, including by J.F. Perkins in his curation of the collections of BMNH.

#### Tycherus macilentus

(Wesmael, 1845)

Phaeogenes macilentus Wesmael, 1845

##### Distribution

Ireland

#### Tycherus modestus

(Wesmael, 1845)

Phaeogenes modestus Wesmael, 1845
grammostoma (Kriechbaumer, 1887, *Phaeogenes*)

##### Distribution

England, Scotland

#### Tycherus ophtalmicus

(Wesmael, 1845)

Phaeogenes ophtalmicus Wesmael, 1845
ophthalmicus misspelling
hybridus (Wesmael, 1845, *Phaeogenes*)
pulchricornis (Brischke, 1891, *Phaeogenes*)
palliventris (Berthoumieu, 1910, *Phaeogenes*)

##### Distribution

England, Scotland, Wales, Ireland

#### Tycherus osculator

(Thunberg, 1824)

Ichneumon osculator Thunberg, 1824
nanus (Wesmael, 1845, *Phaeogenes*)
lascivus (Wesmael, 1855, *Phaeogenes*)
socius (Holmgren, 1890, *Phaeogenes*)
inanis (Berthoumieu, 1901, *Phaeogenes*)
tristis (Berthoumieu, 1904, *Phaeogenes*)
strandi (Berthoumieu, 1910, *Dicaelotus*)
nigroclypeatus (Constantineanu, 1942, *Phaeogenes*)

##### Distribution

England, Scotland, Wales, Ireland

#### Tycherus planipectus

(Holmgren, 1890)

Phaeogenes planipectus Holmgren, 1890

##### Distribution

Scotland

##### Notes

added by Diller and Shaw (2014)

#### Tycherus socialis

(Ratzeburg, 1852)

Ichneumon socialis Ratzeburg, 1852
discoidalis (Ratzeburg, 1852, *Ichneumon*)
clypearis (Brischke, 1878, *Phaeogenes*)
martialis (Pic, 1899, *Phaeogenes*)

##### Distribution

England

##### Notes

added by Diller and Shaw (2014)

#### Tycherus stipator

(Wesmael, 1855)

Phaeogenes stipator Wesmael, 1855
cambriensis (Desvignes, 1867, *Ichneumon*)
cicutellus (Brischke, 1878, *Phaeogenes*)
basirufus (Constantineanu, 1951, *Phaeogenes*)
fuscitarsis (Constantineanu, 1951, *Phaeogenes*)

##### Distribution

England, Scotland, Wales, Ireland

#### Tycherus suspicax

(Wesmael, 1845)

Phaeogenes suspicax Wesmael, 1845
crassiceps (Habermehl, 1917, *Proscus*)

##### Distribution

England, Scotland, Wales, Ireland

#### Tycherus teres

(Berthoumieu, 1906)

Phaeogenes teres Berthoumieu, 1906

##### Distribution

England

##### Notes

added by Diller and Shaw (2014)

#### Tycherus vagus

(Berthoumieu, 1899)

Phaeogenes vagus Berthoumieu, 1899

##### Distribution

England

##### Notes

added by Diller and Shaw (2014)

#### Tycherus verecundus

Ranin, 1983

##### Distribution

England

##### Notes

added by Ranin (1983)

#### PLATYLABINI

Berthoumieu, 1904

PRISTICEROTINI Townes, 1961

#### Apaeleticus

Wesmael, 1845

#### Apaeleticus bellicosus

Wesmael, 1845

inclytus Wesmael, 1853
cautus (Berthoumieu, 1898, *Diadromus*)
rufipes Constantineanu, 1951

##### Distribution

England

#### Apaeleticus inimicus

(Gravenhorst, 1820)

Ichneumon inimicus Gravenhorst, 1820
haematodus (Gravenhorst, 1829, *Cryptus*) synonymy by Sawoniewicz and Wanat (2003)
flammeolus Wesmael, 1845
balearicus Kriechbaumer, 1894
amoenus Habermehl, 1917
muelleri (Kiss, 1929, *Microcryptus*)

##### Distribution

England

#### Asthenolabus

Heinrich, 1951

STENOLABUS Heinrich, 1936 preocc.

#### Asthenolabus latiscapus

(Thomson, 1894)

Platylabus latiscapus Thomson, 1894

##### Distribution

England

#### Asthenolabus vitratorius

(Gravenhorst, 1829)

Mesoleptus vitratorius Gravenhorst, 1829
albinus (Gravenhorst, 1829, *Hoplismenus*)
errabundus (Gravenhorst, 1829, *Hoplismenus*)
tinctorius (Gravenhorst, 1829, *Cryptus*)
coxalis (Habermehl, 1917, *Platylabus*)

##### Distribution

England, Wales

#### Cyclolabus

Heinrich, 1936

#### Cyclolabus axillatorius

(Thunberg, 1824)

Ichneumon axillatorius Thunberg, 1824
pactor (Wesmael, 1845, *Platylabus*) synonymy by Riedel (2014)
pici (Berthoumieu, 1910, *Anisobas*)
septentrionalis (Berthoumieu, 1910, *Dicaelotus*)

##### Distribution

England, Scotland, Ireland

#### Cyclolabus dubiosus

Perkins, 1953

##### Distribution

England

#### Cyclolabus nigricollis

(Wesmael, 1845)

Platylabus nigricollis Wesmael, 1845

##### Distribution

England, Wales

#### Dentilabus

Heinrich, 1974

#### Dentilabus variegatus

(Wesmael, 1845)

Platylabus variegatus Wesmael, 1845

##### Distribution

England, Scotland

#### Ectopius

Wesmael, 1859

#### Ectopius rubellus

(Gmelin, 1790)

Ichneumon rubellus Gmelin, 1790
thedenii (Holmgren, 1871, *Platylabus*)

##### Distribution

Ireland

#### Hypomecus

Wesmael, 1845

#### Hypomecus quadriannulatus

(Gravenhorst, 1829)

Mesoleptus quadriannulatus Gravenhorst, 1829
albitarsis Wesmael, 1845
submarginatus (Magretti, 1896, *Platylabus*) synonymy by Riedel (2008)
carens Berthoumieu, 1897

##### Distribution

England, Scotland

#### Linycus

Cameron, 1903

#### Linycus exhortator

(Fabricius, 1787)

Ichneumon exhortator Fabricius, 1787
dimidiatus (Gravenhorst, 1829, *Hoplismenus*)
discedens (Gravenhorst, 1829, *Phygadeuon*)
latior (Pic, 1902, *Platylabus*)
tricolor (Berthoumieu, 1904, *Platylabus*)
balearicus (Hedwig, 1939, *Platylabus*) synonymy by Riedel (2008)

##### Distribution

England, Scotland, Wales, Ireland

#### Linycus flavitarsis

(Heinrich, 1937)

Ectopius flavitarsis Heinrich, 1937
priesneri Heinrich, 1972

##### Distribution

Scotland

##### Notes

NMS, det. Riedel, added here

#### Platylabus

Wesmael, 1845

##### Notes

synonymy follows Riedel (2008)

#### Platylabus concinnus

Thomson, 1888

##### Distribution

England

#### Platylabus curtorius

(Thunberg, 1824)

Ichneumon curtorius Thunberg, 1824
eurygaster Holmgren, 1871 synonymy by Riedel (2008)
punctifrons Thomson, 1888 synonymy by Riedel (2007)

##### Distribution

England, Scotland, Wales

#### Platylabus daemon

Wesmael, 1845

##### Distribution

Wales

##### Notes

Added by Riedel (2008) and transferred from *Asthenolabus*.

#### Platylabus dolorosus

(Gravenhorst, 1829)

Ichneumon dolorosus Gravenhorst, 1829
sollicitus Wesmael, 1845
viturati (Pic, 1902, *Ichneumon*)

##### Distribution

England, Scotland

#### Platylabus fugator

(Gravenhorst, 1807)

Ichneumon fugator Gravenhorst, 1807
atricornis Pic, 1926 synonymy by Riedel (2007)

##### Distribution

Scotland

##### Notes

added by Riedel (2008)

#### Platylabus gigas

Kriechbaumer, 1886

##### Distribution

England

#### Platylabus heteromallus

(Berthoumieu, 1910)

Amblyteles heteromallus Berthoumieu, 1910
pedatorius misident. (Horstmann 2001a)
rhenana Habermehl, 1917

##### Distribution

England, Scotland, Wales, Ireland, Isle of Man

##### Notes

Removed from synonymy with *curtorius* by Riedel (2008), who found that *curtorius* is a senior synonym of *punctifrons* and *eurygaster*.

#### Platylabus histrio

Wesmael, 1855

varipedulis Wesmael, 1857
erberi Tischbein, 1868

##### Distribution

England, Scotland

#### Platylabus intermedius

Holmgren, 1871

polonicus Heinrich, 1937

##### Distribution

England, Scotland

#### Platylabus iridipennis

(Gravenhorst, 1829)

Ichneumon iridipennis Gravenhorst, 1829
helensis (Brischke, 1888, *Ichneumon*)
fornicatus Kriechbaumer, 1890 synonymy by Horstmann (2006a)
calidus Berthoumieu, 1904 synonymy by Riedel (2008)
novellus Berthoumieu, 1910

##### Distribution

England, Scotland

#### Platylabus judaicus

Berthoumieu, 1900

stolidus Perkins, 1953 synonymy by Riedel (2007)

##### Distribution

England

#### Platylabus neglectus

(Fonscolombe, 1847)

Ichneumon neglectus Fonscolombe, 1847
decipiens Wesmael, 1848 synonymy by Riedel (2007)
minai (De Stefani, 1885, *Ischnus*) synonymy by Riedel (2008)

##### Distribution

England

#### Platylabus nigrocyaneus

(Gravenhorst, 1829)

Ichneumon nigrocyaneus Gravenhorst, 1829
armatus Wesmael, 1845

#### Platylabus obator

(Desvignes, 1856)

Ichneumon obator Desvignes, 1856

##### Distribution

England, Scotland

#### Platylabus odiosus

Perkins, 1953

##### Distribution

England, Scotland

#### Platylabus opaculus

Thomson, 1888

##### Distribution

England, Ireland

#### Platylabus orbitalis

(Gravenhorst, 1829)

Ichneumon orbitalis Gravenhorst, 1829
vibratorius misident. (Riedel 2008)
persecutor (Gravenhorst, 1829, *Ichneumon*)
subalbellus (Gravenhorst, 1829, *Ichneumon*)
volubilis (Gravenhorst, 1829, *Ichneumon*) synonymy by Riedel (2008)
suborbitalis Kriechbaumer, 1894 synonymy by Horstmann (2006a)
muticus Thomson, 1894 synonymy by Riedel (2007)

##### Distribution

England, Scotland, Wales

#### Platylabus perexiguus

Heinrich, 1973

##### Distribution

England

##### Notes

NMS, det. Riedel, added here

#### Platylabus pseudopumilio

Riedel, 2008

##### Distribution

England, Scotland, Wales

##### Notes

added by Riedel (2008)

#### Platylabus pumilio

Holmgren, 1871

##### Distribution

England

#### Platylabus rufus

Wesmael, 1845

pictus Vollenhoven, 1878
rubeus Valemberg, 1976 synonymy by Valemberg (2001)

##### Distribution

England, Scotland, Wales, Ireland

#### Platylabus sternoleucus

Wesmael, 1853

##### Distribution

England, Scotland, Wales

##### Notes

Added by Riedel (2008) and transferred from *Asthenolabus*.

#### Platylabus tenuicornis

(Gravenhorst, 1829)

Ichneumon tenuicornis Gravenhorst, 1829
niger Wesmael, 1845

##### Distribution

England

#### Platylabus transversus

Bridgman, 1889

lativentris Thomson, 1894

##### Distribution

England

#### Platylabus tricingulatus

(Gravenhorst, 1820)

Ichneumon tricingulatus Gravenhorst, 1820
maurus Berthoumieu, 1900
berthoumieui Pic, 1923
zagoriensis Heinrich, 1930 synonymy by Riedel (2008)

##### Distribution

England

#### Platylabus vibratorius

(Thunberg, 1824)

Ichneumon vibratorius Thunberg, 1824
wienkeri (Ratzeburg, 1844, *Ichneumon*) synonymy by Riedel (2008)
rufiventris Wesmael, 1845 synonymy by Horstmann (2000b)

##### Distribution

England

#### Platymischos

Tischbein, 1868

RYSSOLABUS Berthoumieu, 1894

#### Platymischos atriventris

(Pic, 1914)

Ryssolabus atriventris Pic, 1914
arcticus (Hellén, 1942, *Ryssolabus*)
montanus (Heinrich, 1951, *Ryssolabus*)

##### Distribution

Scotland

#### Poecilostictus

Ratzeburg, 1852

IDIOSTOLIS Förster, 1869
NEOPLATYLABUS Heinrich, 1936

#### Poecilostictus cothurnatus

(Gravenhorst, 1829)

Hoplismenus cothurnatus Gravenhorst, 1829
orbitatus (Gravenhorst, 1829, *Hoplismenus*)
octopunctatus Ratzeburg, 1852
ratzeburgi Kawall, 1868 synonymy by Horstmann (1997)
apicalis (Brischke, 1892, *Hepiopelmus*)
geometrae (Berthoumieu, 1894, *Platylabus*)
saxonicus (Hedwig, 1939, *Platylabus*)

##### Distribution

England, Wales

#### Pristicerops

Heinrich, 1961

#### Pristicerops infractorius

(Linnaeus, 1761)

Ichneumon infractorius Linnaeus, 1761
phaleratus (Haliday, 1839, *Ichneumon*)
leucogrammus (Wesmael, 1853, *Platylabus*)

##### Distribution

England, Scotland, Wales, Ireland, Isle of Man

#### Pristiceros

Gravenhorst, 1829

#### Pristiceros serrarius

Gravenhorst, 1829

##### Distribution

England

#### ZIMMERIINI

Heinrich, 1934

#### Cotiheresiarches

Telenga, 1929

ZIMMERIA Heinrich, 1934

#### Cotiheresiarches dirus

(Wesmael, 1853)

Eurylabus dirus Wesmael, 1853
niger Telenga, 1929

##### Notes

Specimens in BMNH lack locality data but reliably recorded as British by Perkins (1959).

### Lycorininae

#### LYCORININAE

Cushman & Rohwer, 1920

LYCORINAE misspelling

#### Lycorina

Holmgren, 1859

AMYS Schiødte, 1839 nom. ob., synonymy by Horstmann (2004a)
TOXOPHOROIDES Cresson, 1873
CHLOROLYCORINA Cushman, 1920
GONIOGLYPHUS Seyrig, 1932

#### Lycorina triangulifera

Holmgren, 1859

flavilabris (Schiødte, 1839, *Amys*) nom. ob., synonymy by Horstmann (2004a)
lycorinoides (Costa, 1886, *Glypta*)
sardoa (Costa, 1886, *Glypta*)

##### Distribution

England, Scotland, Ireland

##### Notes

some distribution data from Shaw (2004)

### Mesochorinae

#### MESOCHORINAE

Förster, 1869

##### Notes

Schwenke (1999) reinstated various synonyms of *Astiphromma* and *Mesochorus* as valid genera. This treatment is not followed here as Wahl (1993) gave good reasons for treating these as synonyms. Looking at material from across the globe, there are no clear-cut differences between, for example, *Stictopisthus* and *Mesochorus*. An exception is *Dolichochorus*, q.v. Distribution data, unless noted otherwise, are taken from Schwenke (1999), NMS, BMNH and UM.

#### Astiphromma

Förster, 1869

ASTIPHROMMUS Thomson, 1886
MESOCHORELLA Szépligeti, 1911
PSEUDACOENITUS Kiss, 1924
DEMOPHORELLUS Hedwig, 1955

##### Notes

Some distribution data and much synonymy from Riedel (2015), with most identifications by M. Riedel and GRB. J.F. Perkins, A. Roman and W. Schwenke had also identified a few interesting specimens in BMNH.

species of *Astiphromma* excluded from the British and Irish list:

[*dorsale* (Holmgren, 1860, *Mesochorus*)] No British or Irish specimens have been seen (Riedel 2015). The previously synonymous name *hirsutum*, described from British specimens, is a senior synonym of *granigerum*.

[*striatum* (Brischke, 1880, *Mesochorus*); syn. *mandibulare* (Thomson, 1886, *Mesochorus*)] All British specimens under the name *mandibulare* (synonymised under *striatum* by Riedel 2015) in BMNH have proved to be misidentifications.

#### Astiphromma aggressor

(Fabricius, 1804)

Ophion aggressor Fabricius, 1804
marginellum (Holmgren, 1860, *Mesochorus*)
alpinum (Roman, 1909, *Mesochorus*)
barbatum Schwenke, 1999
caecum Schwenke, 1999

##### Distribution

England

##### Notes

added by Riedel (2015)

#### Astiphromma albitarse

(Brischke, 1880)

Mesochorus albitarsis Brischke, 1880
nigrum Pfankuch, 1921
heydeni Habermehl, 1923
transsylvanicum (Kiss, 1924, *Pseudacoenitus*)

##### Distribution

England, Scotland

##### Notes

added by Riedel (2015)

#### Astiphromma alpinum

Roman, 1909

dispersum Schwenke, 1999
laricis Schwenke, 1999

##### Distribution

England

##### Notes

added by Riedel (2015)

#### Astiphromma anale

(Holmgren, 1860)

Mesochorus analis Holmgren, 1860

##### Distribution

England, Scotland

##### Notes

added by Riedel (2015)

#### Astiphromma buccatum

(Thomson, 1886)

Mesochorus buccatus Thomson, 1886
hamulum (Thomson, 1886, *Mesochorus*)
consertum Schwenke, 1999

##### Distribution

England

#### Astiphromma hirsutum

(Bridgman, 1883)

Mesochorus hirsutus Bridgman, 1883
granigerum (Thomson, 1886, *Mesochorus*)

##### Distribution

England, Scotland, Ireland

#### Astiphromma italicum

Schwenke, 1999

contum Schwenke, 1999

##### Distribution

England

##### Notes

added by Riedel (2015)

#### Astiphromma leucogrammum

(Holmgren, 1860)

Mesochorus leucogrammus Holmgren, 1860

##### Distribution

England, Scotland

##### Notes

NMS, added here

#### Astiphromma nigrocoxatum

(Strobl, 1904)

Mesochorus nigrocoxatus Strobl, 1904
mimulum (Hedwig, 1955, *Demophorellus*)

##### Distribution

England

##### Notes

added by Riedel (2015)

#### Astiphromma pictum

(Brischke, 1880)

Mesochorus pictus Brischke, 1880
incidens (Thomson, 1886, *Mesochorus*)

##### Distribution

England

#### Astiphromma scutellatum

(Gravenhorst, 1829)

Mesochorus scutellatus Gravenhorst, 1829
festivum (Holmgren, 1860, *Mesochorus*)

##### Distribution

England

#### Astiphromma splenium

(Curtis, 1833)

Mesochorus splenium Curtis, 1833
sericans (Curtis, 1833, *Mesochorus*)
strenuum (Holmgren, 1860, *Mesochorus*)
plagiatum (Thomson, 1886, *Mesochorus*) synonymy by Schwenke (1999)

##### Distribution

England, Scotland, Ireland

##### Notes

*Astiphromma sericans* was reinstated as a valid species by Horstmann (2006b) but synonymised under *splenium* again by Riedel (2015).

#### Astiphromma tenuicorne

(Thomson, 1886)

Mesochorus tenuicornis Thomson, 1886

##### Distribution

England, Scotland

#### Astiphromma trimaculosum

Schwenke, 2004

##### Distribution

England

##### Notes

added by Schwenke (2004)

#### Astiphromma uliginosum

Schwenke, 1999

##### Distribution

England, Wales, Ireland

##### Notes

added by Riedel (2015)

#### Astiphromma varipes

(Holmgren, 1860)

Mesochorus varipes Holmgren, 1860
variipes Dalla Torre, 1901

##### Distribution

England, Scotland

##### Notes

added by Riedel (2015)

#### Cidaphus

Förster, 1869

PLESIOPHTHALMUS Förster, 1869
MATER Schluz, 1911
TETRAGONALYS Morley, 1913
OPHTHALMOCHORUS Roman, 1925

##### Notes

Distribution data for *Cidaphus* species is taken from Fitton (1985b) and NMS. Note that Schwenke (1999) used an out-of-date taxonomy for *Cidaphus* species. Fitton (1985b) and Horstmann (2002b) are followed here.

#### Cidaphus alarius

(Gravenhorst, 1829)

Mesochorus alarius Gravenhorst, 1829
thuringiacus Brauns, 1889

##### Distribution

England, Wales

#### Cidaphus areolatus

(Boie, 1850)

Paniscus areolatus Boie, 1850
gigas (Kriechbaumer, 1897, *Meoschorus*) synonymy by Horstmann (2002b)
brischkei (Szépligeti, 1911, *Plesiophthalmus*) synonymy by Horstmann (2002b)

##### Distribution

England, Scotland

##### Notes

Added by Fitton (1985b); listed as a synonym of *alarius* in Yu and Horstmann (1997), with *brischkei* as the valid name for this species.

#### Cidaphus atricillus

(Haliday, 1838)

Cryptus atricillus Haliday, 1838
potanini Kokujev, 1906
melanocephalus (Habermehl, 1909, *Plesiophthalmus*)

##### Distribution

England, Scotland, Wales

#### Dolichochorus

Strobl, 1904

##### Notes

*Mesochorus longiceps* is the type species of *Dolichochorus* Strobl, 1904. This genus was synonymised under *Astiphromma* by Townes et al. (1965) but reinstated as a valid genus by Schwenke (1999). Wahl (1993) listed *Dolichochorus* as a synonym of *Astiphromma* but did not include *longiceps* in his cladistic analysis. Broad & Watanabe (in prep.) have found that *Dolichochorus* is a rather basal member of the Mesochorinae, and distinct from *Astiphromma*.

#### Dolichochorus longiceps

(Strobl, 1904)

Mesochorus longiceps Strobl, 1904

##### Distribution

England

##### Notes

Added by Schwenke (1999); it is not known on what basis Schwenke (1999) recorded this as a British species but its occurrence in Britain is confirmed by Riedel (2015) and Broad & Watanabe (in prep.).

#### Mesochorus

Gravenhorst, 1829

STICTOPISTHUS Thomson, 1886

##### Notes

Horstmann (2006b) substantially revised the taxonomy of various species of *Mesochorus* as W. Schwenke had misinterpreted many names.

species of *Mesochorus* excluded from the British and Irish list:

[*nuncupator* (Panzer, 1800, *Ichneumon*)] Horstmann (2006b)

#### Mesochorus aggestus

Schwenke, 2002

sulcatus Schwenke, 1999

##### Distribution

England

##### Notes

added by Schwenke (1999)

#### Mesochorus albionis

Schwenke, 1999

##### Distribution

England

##### Notes

added by Schwenke (1999)

#### Mesochorus alpigenus

Strobl, 1904

compactus Schwenke, 1999 synonymy by Horstmann (2001b)

##### Distribution

Scotland

#### Mesochorus anglicus

Schwenke, 1999

##### Distribution

England

##### Notes

added by Schwenke (1999)

#### Mesochorus angustatus

Thomson, 1886

#### Mesochorus anomalus

Holmgren, 1860

#### Mesochorus arenarius

(Haliday, 1839)

Cryptus arenarius Haliday, 1839
nigripes Ratzeburg, 1852 Horstmann (2006b)
melas Fonscolombe, 1852
gibbulus Holmgren, 1856

##### Distribution

England, Scotland, Ireland

#### Mesochorus atriventris

Cresson, 1872

sylvarum (Haliday, 1839, *Cryptus*) synonymy by Horstmann (2006b)

##### Distribution

England, Scotland, Ireland, Isle of Man

##### Notes

some distribution data from Shaw (1993), Horstmann (2006b)

#### Mesochorus basalis

Curtis, 1833

##### Distribution

England

#### Mesochorus bracatus

Schwenke, 1999

##### Distribution

Ireland

##### Notes

added by Schwenke (1999)

#### Mesochorus brevipetiolatus

Ratzeburg, 1844

#### Mesochorus britannicus

Schwenke, 1999

##### Distribution

England

##### Notes

added by Schwenke (1999)

#### Mesochorus carinatus

Schwenke, 1999

##### Distribution

England

##### Notes

added by Schwenke (1999)

#### Mesochorus cimbicis

Ratzeburg, 1844

confusus Holmgren, 1860 synonymy by Horstmann (2006b)
longicauda Thomson, 1886 synonymy by Schwenke (1999)
gallicator Aubert, 1963 synonymy by Schwenke (1999)

##### Distribution

England, Scotland

#### Mesochorus dimidiator

Aubert, 1970

##### Distribution

England

##### Notes

added by Horstmann (2006b)

#### Mesochorus discitergus

(Say, 1835)

Cryptus discitergus Say, 1835
facialis Bridgman, 1884
baueri Schwenke, 1999 synonymy by Horstmann (2003b)

##### Distribution

England

##### Notes

distribution data from Horstmann (2003b)

#### Mesochorus discolor

Schwenke, 1999

##### Distribution

England

##### Notes

added by Schwenke (1999)

#### Mesochorus dispar

Brischke, 1880

##### Distribution

Ireland

##### Notes

added by Horstmann (2002c)

#### Mesochorus errabundus

Hartig, 1838

politus misident.

##### Distribution

England, Scotland

##### Notes

BMNH, added here; this species was treated as a synonym of *politus* Gravenhorst, 1829 by Schwenke (1999) but Horstmann (2003b) established *errabundus* as a distinct species. Using the characters given by Horstmann for distinguishing the two species, both occur here.

#### Mesochorus extensator

Schwenke, 2002

##### Distribution

England

##### Notes

added by Schwenke (2002)

#### Mesochorus flavescens

Fonscolombe, 1852

##### Distribution

England

##### Notes

added by Schwenke (1999)

#### Mesochorus formosus

Bridgman, 1882

convexicollis Thomson, 1886 synonymy by Schwenke (1999)

##### Distribution

England, Scotland

#### Mesochorus fulgurans

Curtis, 1833

fulgurans (Haliday, 1839, *Cryptus*) preocc.
pectinipes Thomson, 1886 synonymy by Horstmann (2006b)
fulvus Thomson, 1886 synonymy by Horstmann (2006b)
suecicus Dalla Torre, 1901 synonymy by Horstmann (2006b)

##### Distribution

England, Scotland, Ireland

##### Notes

some distribution data from Gauld (1970)

#### Mesochorus fulgurator

Horstmann, 2006

##### Distribution

England

##### Notes

BMNH, det. Broad, added here

#### Mesochorus fuscicornis

Brischke, 1880

##### Distribution

England, Scotland

#### Mesochorus fuscus

Schwenke, 1999

##### Distribution

England

##### Notes

added by Schwenke (1999), Schwenke (2004)

#### Mesochorus gemellus

Holmgren, 1860

tachypus Holmgren, 1860
brevicollis Thomson, 1886

##### Distribution

England

#### Mesochorus giberius

(Thunberg, 1824)

Ichneumon giberius Thunberg, 1824
thoracicus Gravenhorst, 1829
sylvarum Curtis, 1833 synonymy by Horstmann (2006b)
marginatus Thomson, 1886 synonymy by Schwenke (1999)

##### Distribution

England, Scotland, Ireland

#### Mesochorus globulator

(Thunberg, 1824)

Ichneumon globulator Thunberg, 1824
crassimanus Holmgren, 1860
dimidiatus Holmgren, 1860
sericeus Brischke, 1880 synonymy by Horstmann (2006b)

##### Distribution

Scotland, Isle of Man

##### Notes

some distribution data from Horstmann (2004b), Horstmann (2006b)

#### Mesochorus gracilentus

Brischke, 1880

##### Notes

reinstated by Horstmann (2006b)

#### Mesochorus iniquus

Schwenke, 1999

##### Distribution

England, Scotland

##### Notes

added by Horstmann (2006b)

#### Mesochorus insularis

Schwenke, 1999

##### Distribution

England

##### Notes

added by Schwenke (1999)

#### Mesochorus jenniferae

Schwenke, 2002

##### Distribution

England

##### Notes

added by Schwenke (2002), Schwenke (2004)

#### Mesochorus laricis

Hartig, 1838

##### Distribution

England

##### Notes

BMNH, det. Horstmann, added here

#### Mesochorus latus

Schwenke, 1999

##### Distribution

England

##### Notes

added by Schwenke (1999)

#### Mesochorus lilioceriphilus

Schwenke, 2000

##### Distribution

England

##### Notes

added by Salisbury and Broad (2011)

#### Mesochorus liquidus

Schwenke, 2002

##### Distribution

England

##### Notes

added by Schwenke (2002)

#### Mesochorus nematus

Schwenke, 2004

##### Distribution

England

##### Notes

added by Schwenke (2004)

#### Mesochorus olerum

Curtis, 1833

pectoralis Ratzeburg, 1844 synonymy by Horstmann (2006b)
rapae Schwenke, 1999 synonymy by Horstmann (2008a)

##### Distribution

England

#### Mesochorus orbitalis

Holmgren, 1860

##### Distribution

England, Scotland

#### Mesochorus owenae

Schwenke, 1999

##### Distribution

England

##### Notes

added by Schwenke (1999)

#### Mesochorus oxfordensis

Schwenke, 1999

##### Distribution

England

##### Notes

added by Schwenke (1999)

#### Mesochorus pallipes

Brischke, 1880

stigmaticus Brischke, 1880 synonymy by Horstmann (2006b)
brunneus Brischke, 1880 synonymy by Horstmann (2006b)
rufipes Brischke, 1880 synonymy by Horstmann (2006b)
albipes Thomson, 1886 synonymy by Schwenke (1999)
crassicrus Thomson, 1886 synonymy by Horstmann (2006b)

##### Distribution

England, Scotland, Wales

##### Notes

*Mesochorus pallipes* *sensu* Schwenke (1999) is not this species (Horstmann 2006b).

#### Mesochorus pectinellus

Horstmann, 2006

##### Distribution

England

##### Notes

BMNH, det. Horstmann, added here

#### Mesochorus pectinipes

Bridgman, 1883

##### Distribution

England

#### Mesochorus perticatus

Schwenke, 1999

##### Distribution

England

##### Notes

added by Schwenke (1999)

#### Mesochorus pictilis

Holmgren, 1860

#### Mesochorus politus

Gravenhorst, 1829

##### Distribution

England

#### Mesochorus pumilionis

Schwenke, 1999

##### Distribution

England

##### Notes

added by Schwenke (1999), Schwenke (2004)

#### Mesochorus punctipleuris

Thomson, 1886

nigriceps Thomson, 1886 preocc., synonymy by Horstmann (2001c)
thomsonii Dalla Torre, 1901
thomsoni Strobl, 1904 preocc.
amplitudinis Schwenke, 1999 synonymy by Horstmann (2002c)

##### Distribution

England, Ireland

##### Notes

Added by Horstmann (2002c); listed as a synonym of *M. agilis* Cresson, 1865 in Yu and Horstmann (1997).

#### Mesochorus quercus

Schwenke, 2004

##### Distribution

England

##### Notes

added by Schwenke (2004)

#### Mesochorus rufoniger

Brischke, 1880

brevigena Thomson, 1886

##### Distribution

England, Scotland

##### Notes

NMS, UM, det. Brock, added here

#### Mesochorus rutilus

Schwenke, 2002

##### Distribution

England

##### Notes

added by Schwenke (2002)

#### Mesochorus scutellaris

Schwenke, 2004

##### Distribution

England

##### Notes

added by Schwenke (2004)

#### Mesochorus semirufus

Holmgren, 1860

#### Mesochorus stigmator

(Thunberg, 1824)

Ichneumon stigmator Thunberg, 1824
splendidulus Gravenhorst, 1829 synonymy by Horstmann (2001b)
pallidus Brischke, 1880
stigmaticus Thomson, 1886 preocc.
orgyiae Dalla Torre, 1902

#### Mesochorus temporalis

Thomson, 1886

##### Distribution

England

#### Mesochorus tenuiscapus

Thomson, 1886

##### Distribution

England, Scotland

#### Mesochorus testaceus

Gravenhorst, 1829

##### Distribution

England

#### Mesochorus tetricus

Holmgren, 1860

macrurus Thomson, 1886

##### Distribution

England

#### Mesochorus trifoveatus

Schwenke, 2004

##### Distribution

England

##### Notes

added by Schwenke (2004)

#### Mesochorus unicinctor

(Thunberg, 1824)

Ichneumon unicinctor Thunberg, 1824
complanatus (Haliday, 1839, *Cryptus*) synonymy by Schwenke (1999)
aciculatus Bridgman, 1881
laticeps Thomson, 1886

##### Distribution

England, Ireland

#### Mesochorus velox

Holmgren, 1860

#### Mesochorus vittator

(Zetterstedt, 1838)

Tryphon vittator Zetterstedt, 1838

##### Distribution

Isle of Man

##### Notes

added by Horstmann (2006b)

#### Mesochorus vitticollis

Holmgren, 1860

hungaricus Szépligeti, 1914 synonymy by Schwenke (1999)

##### Distribution

England, Scotland, Wales

#### Mesochorus windsorianus

Schwenke, 2004

##### Distribution

England

##### Notes

added by Schwenke (2004)

### Metopiinae

#### METOPIINAE

Förster, 1869

#### Carria

Schmiedeknecht, 1924

#### Carria paradoxa

Schmiedeknecht, 1924

##### Distribution

England

#### Chorinaeus

Holmgren, 1858

#### Chorinaeus australis

Thomson, 1887

flavifrons Schmiedeknecht, 1925 preocc.
xanthopsis (Townes, 1946, *Trieces*)

##### Distribution

England, Wales

#### Chorinaeus brevicalcar

Thomson, 1887

##### Distribution

England, Scotland

#### Chorinaeus cristator

(Gravenhorst, 1829)

Exochus cristator Gravenhorst, 1829

##### Distribution

England, Scotland, Wales

#### Chorinaeus flavipes

Bridgman, 1881

##### Distribution

England, Scotland

#### Chorinaeus funebris

(Gravenhorst, 1829)

Exochus funebris Gravenhorst, 1829
femoratus Teunissen, 1948

##### Distribution

England, Scotland, Wales

#### Chorinaeus hastianae

Aeschlimann, 1975

##### Distribution

England, Wales

#### Chorinaeus longicornis

Thomson, 1887

##### Distribution

England, Scotland

#### Chorinaeus rhenanus

Aeschlimann, 1981

##### Distribution

England

##### Notes

NMS, det. Aeschlimann, added here

#### Chorinaeus subcarinatus

Holmgren, 1858

longicalcar Thomson, 1887

##### Distribution

England, Scotland

#### Chorinaeus talpa

(Haliday, 1839)

Exochus talpa Haliday, 1839

##### Distribution

England, Scotland, Ireland

#### Colpotrochia

Holmgren, 1856

#### Colpotrochia cincta

(Scopoli, 1763)

Sphex cincta Scopoli, 1763
elegantula (Schrank, 1781, *Ichneumon*)
mandator (Fabricius, 1787, *Ichneumon*) preocc.
mundator (Thunberg, 1824, *Ichneumon*)
affinis Vollenhoven, 1875

##### Distribution

England, Scotland

#### Exochus

Gravenhorst, 1829

Amesolytus Förster, 1869

##### Notes

doubtfully placed species of *Exochus*

[*antiquus* Haliday, 1839 nom. dub.]

#### Exochus albicinctus

Holmgren, 1873

anospilus Thomson, 1887
nigricans (Szépligeti, 1898, *Amesolytus*)

##### Distribution

England, Scotland

#### Exochus alpinus

(Zetterstedt, 1838)

Bassus alpinus Zetterstedt, 1838

##### Distribution

England

#### Exochus britannicus

Morley, 1911

##### Distribution

England

#### Exochus carri

Schmiedeknecht, 1924

##### Distribution

England

#### Exochus citripes

Thomson, 1877

##### Distribution

England

##### Notes

BMNH, added here

#### Exochus consimilis

Holmgren, 1858

parvispina Thomson, 1887
decoloratus Schmiedeknecht, 1924
subalpinus Schmiedeknecht, 1924

##### Distribution

England

##### Notes

BMNH, UM, added here

#### Exochus erythronotus

(Gravenhorst, 1820)

Ichneumon erythronotus Gravenhorst, 1820
concinnus Holmgren, 1858
pumilus Holmgren, 1873
rufidorsum (Szépligeti, 1898, *Amesolytus*)
ghigii Ferrière, 1929

##### Distribution

England

#### Exochus flavomarginatus

Holmgren, 1856

##### Distribution

England, Scotland

#### Exochus fletcheri

Bridgman, 1884

femoralis Pfankuch, 1925

##### Distribution

England, Scotland

#### Exochus frontellus

Holmgren, 1858

#### Exochus gravipes

(Gravenhorst, 1820)

Ichneumon gravipes Gravenhorst, 1820

##### Distribution

England, Scotland

#### Exochus gravis

Gravenhorst, 1829

#### Exochus intermedius

Morley, 1911

##### Distribution

England

#### Exochus lentipes

Gravenhorst, 1829

cylindricus Holmgen, 1858

#### Exochus lictor

Haliday, 1839

pectoralis Haliday, 1839
decoratus Holmgren, 1873

##### Distribution

England, Scotland, Ireland

#### Exochus mitratus

Gravenhorst, 1829

affninis Holmgren, 1858
australis Thomson, 1894
pseudaffinis Strobl, 1903
paradoxus Schmiedeknecht, 1924
punctifer Schmiedeknecht, 1924

#### Exochus nigripalpis

Thomson, 1887

##### Distribution

England

#### Exochus notatus

Holmgren, 1858

woldstedtii Holmgren, 1873

##### Distribution

England, Scotland

#### Exochus pictus

Holmgren, 1858

##### Distribution

England, Scotland, Ireland

#### Exochus prosopius

Gravenhorst, 1829

maculatus Brischke, 1871
procerus Holmgren, 1873
dioszeghyi Kiss, 1926

##### Distribution

England, Scotland

#### Exochus ratzeburgi

Holmgren, 1858

##### Notes

BMNH, added here

#### Exochus rubroater

Schmiedeknecht, 1924

##### Distribution

England, Ireland

#### Exochus semilividus

Vollenhoven, 1875

longicornis Thomson, 1887

##### Distribution

England

##### Notes

BMNH, added here

#### Exochus septentrionalis

Holmgren, 1873

#### Exochus thomsoni

Schmiedeknecht, 1924

crassicornis Thomson, 1894 preocc.

##### Distribution

England

##### Notes

BMNH, added here

#### Exochus tibialis

Holmgren, 1858

##### Distribution

England, Scotland

#### Hypsicera

Latreille, 1829

METACOELUS Förster, 1869
POLYCLISTUS Förster, 1869

#### Hypsicera britannica

Tolkanitz, 2011

anglica (Schmiedeknecht, 1925, *Metacoelus*) preocc.

##### Distribution

England

##### Notes

NMS, det. Aeschlimann, added here; despite its original and replacement names, described from the Channel Islands.

#### Hypsicera curvator

(Fabricius, 1793)

Ichneumon curvator Fabricius, 1793
mansuetor (Gravenhorst, 1807, *Ichneumon*)
affinis (Zetterstedt, 1838, *Bassus*)

##### Distribution

England, Scotland, Isle of Man

#### Hypsicera femoralis

(Geoffroy, 1785)

Ichneumon femoralis Geoffroy, 1785

##### Distribution

England

#### Hypsicera flaviceps

(Ratzeburg, 1852)

Exochus flaviceps Ratzeburg, 1852

##### Distribution

England, Scotland, Ireland

#### Hypsicera subtilitor

Aubert, 1969

##### Distribution

England

##### Notes

added by Aeschlimann (1989)

#### Ischyrocnemis

Holmgren, 1858

TEROZOA Förster, 1869
TERATOZOA Schulz, 1906

#### Ischyrocnemis goesi

Holmgren, 1858

##### Distribution

England

##### Notes

added by Broad and Shaw (2005)

#### Metopius

Panzer, 1806

#### Ceratopius

Clément, 1927

#### Metopius (Ceratopius) citratus

(Geoffroy, 1762)

Ichneumon citratus Geoffroy, 1762
dissectorius (Panzer, 1805, *Ichneumon*) synonymy by Horstmann (2006c)
sicarius Gravenhorst, 1829
zagoriensis Hensch, 1928

##### Distribution

England, Scotland, Ireland

#### Metopius

Panzer, 1806

PELTOPIUS Clément, 1927

#### Metopius (Metopius) anxius

Wesmael, 1849

intermedius Förster, 1850
peltator Marshall, 1874

##### Distribution

England, Scotland, Wales, Ireland

#### Peltastes

Illiger, 1807

TYLOPIUS Townes, 1959 synonymy by Horstmann (2001a)

#### Metopius (Peltastes) leiopygus

Förster, 1850

marchandi Dominique, 1898
krapinensis Hensch, 1928

##### Distribution

England, Ireland

#### Metopius (Peltastes) pinatorius

Brullé, 1846

meridionalis Hensch, 1928
gracilis Clément, 1930

##### Distribution

England, Scotland, Ireland

#### Peltocarus

Thomson, 1887

CLEMONTIA Michener, 1941

#### Metopius (Peltocarus) croceicornis

Thomson, 1887

chrysopus (Lewin, 1797, *Ichneumon*) preocc.

#### Metopius (Peltocarus) dentatus

(Fabricius, 1779)

Ichneumon dentatus Fabricius, 1779
Metopius (Peltocarus) dentatus ?*fasciatus* (Geoffroy, 1785, *Ichneumon*)
Metopius (Peltocarus) dentatus ?*lunulatus* (Villers, 1789, *Ichneumon*)
micratorius (Fabricius, 1804, *Ichneumon*)
denticularis (Thunberg, 1824, *Ichneumon*)
pini (Curtis, 1824, *Peltastes*)
incisus Clément, 1930

##### Distribution

England, Scotland, Ireland

#### Periope

Haliday, 1839

MONOPLECTRON Holmgren, 1856
OLIGOPLECTRON Förster, 1869
MONOPLECTROCHUS Heinrich, 1949

#### Periope auscultator

Haliday, 1839

zygaenator (Holmgren, 1856, *Monoplectron*)

##### Distribution

England, Scotland, Ireland

#### Scolomus

Townes, 1969

APOLOPHUS Townes, 1971 synonymy by Gauld and Wahl (2006)

#### Scolomus borealis

(Townes, 1971)

Apolophus borealis Townes, 1971

##### Distribution

England, Scotland, Wales, Ireland

##### Notes

added by Owen et al. (1981); Broad and Shaw (2005)

#### Stethoncus

Townes, 1959

#### Stethoncus monopicida

Broad & Shaw, 2005

sulcator misident.

##### Distribution

England, Scotland

##### Notes

Added by Gauld and Sithole (2002); recorded (in the context of a host record) as *Stethoncus sulcator* Aubert, 1963.

#### Synosis

Townes, 1959

#### Synosis caesiellae

Broad & Shaw, 2005

##### Distribution

England, Scotland

##### Notes

added by Broad and Shaw (2005)

#### Synosis fieldi

Broad & Shaw, 2005

##### Distribution

England

##### Notes

added by Broad and Shaw (2005)

#### Synosis parenthesellae

Broad & Shaw, 2005

##### Distribution

England, Scotland

##### Notes

added by Broad and Shaw (2005)

#### Triclistus

Förster, 1869

#### Triclistus aethiops

(Gravenhorst, 1829)

Exochus aethiops Gravenhorst, 1829

##### Distribution

England, Scotland

#### Triclistus albicinctus

Thomson, 1887

#### Triclistus anthophilae

Aeschlimann, 1983

##### Distribution

England, Scotland, Wales

##### Notes

added by Aeschlimann (1983); Shaw (1984)

#### Triclistus areolatus

Thomson, 1887

##### Distribution

England, Scotland

#### Triclistus congener

(Holmgren, 1858)

Exochus congener Holmgren, 1858
meridionator Aubert, 1960

##### Distribution

England, Scotland

#### Triclistus epermeniae

Shaw & Aeschlimann, 1994

##### Distribution

England, Scotland

##### Notes

added by Shaw and Aeschliman (1994)

#### Triclistus facialis

Thomson, 1887

#### Triclistus globulipes

(Desvignes, 1856)

Exochus globulipes Desvignes, 1856
holmgreni (Bohemani, 1863, *Exochus*)

##### Distribution

England, Scotland, Wales

#### Triclistus lativentris

Thomson, 1887

##### Distribution

England

#### Triclistus longicalcar

Thomson, 1887

##### Distribution

England, Scotland

#### Triclistus niger

(Bridgman, 1883)

Exochus niger Bridgman, 1883

##### Distribution

England, Scotland

#### Triclistus pallipes

Holmgren, 1873

nitifrons Thomson, 1887
pallidipes Dalla Torre, 1901

##### Distribution

England, Scotland

##### Notes

*Triclistus nitifrons* was regarded as a separate species by J.F. Perkins in his curation of the BMNH collection, with specimens from England and Scotland.

#### Triclistus podagricus

(Gravenhorst, 1829)

Exochus podagricus Gravenhorst, 1829
nigritellus Holmgren, 1873

##### Distribution

England, Scotland, Ireland

#### Triclistus pubiventris

Thomson, 1887

##### Distribution

England, Scotland

#### Triclistus pygmaeus

(Cresson, 1864)

Exochus pygmaeus Cresson, 1864

##### Distribution

England, Scotland, Wales

#### Triclistus spiracularis

Thomson, 1887

##### Distribution

England

#### Triclistus squalidus

(Holmgren, 1858)

Exochus squalidus Holmgren, 1858

##### Distribution

England, Wales

#### Triclistus yponomeutae

Aeschlimann, 1973

##### Distribution

England, Scotland

#### Trieces

Townes, 1946

#### Trieces thuringiacus

(Schmiedeknecht, 1925)

Chorinaeus thuringiacus Schmiedeknecht, 1925

##### Distribution

England

##### Notes

NMS, det. Aeschlimann, added here

#### Trieces tricarinatus

(Holmgren, 1858)

Chorinaeus tricarinatus Holmgren, 1858

##### Distribution

England, Scotland, Ireland

### Microleptinae

#### MICROLEPTINAE

Townes, 1958

##### Notes

Microleptinae as treated here includes only the genus *Microleptes* (Broad 2004), contra Humala (1997). Dasch (1992) gives some distribution data.

#### Microleptes

Gravenhorst, 1829

MIOMERIS Förster, 1869
MIONOMERIS Schulz, 1906
GNATHONIELLA Schmiedeknecht, 1924

#### Microleptes aquisgranensis

(Förster, 1871)

Miomeris aquisgranensis Förster, 1871

##### Distribution

England, Scotland, Wales

#### Microleptes rectangulus

(Thomson, 1888)

Miomeris rectangulus Thomson, 1888
exareolatus (Strobl, 1903, *Seleucus*) synonymy by Horstmann (2011b)
egregius (Schmiedeknecht, 1924, *Gnathoniella*)

##### Distribution

England, Scotland, Wales, Ireland

#### Microleptes splendidulus

Gravenhorst, 1829

glabriventris (Thomson, 1888, *Miomeris*)

##### Distribution

England, Scotland, Ireland

### Neorhacodinae

#### NEORHACODINAE

Hedicke, 1922

##### Notes

Although synonymised with Tersilochinae by Quicke et al. (2009) the Neorhacodinae are here treated as a separate subfamily again following the phylogenetic results of Bennett et al. (A.M.R. Bennett, pers. comm.) and in light of the different host associations.

#### Neorhacodes

Hedicke, 1922

RHACODES Ruschka, 1922

##### Notes

Distribution data from Fitton 1984, Boston 1986 and Notton and Shaw (1988).

#### Neorhacodes enslini

(Ruschka, 1922)

Rhacodes enslini Ruschka, 1922

##### Distribution

England, Scotland, Ireland

### Ophioninae

#### OPHIONINAE

Shuckard, 1840

##### Notes

Unless stated otherwise, all distribution data are taken from Brock (1982), Gauld (1973), Gauld (1974) and from the nocturnal Ichneumonoidea recording scheme (database maintained by GRB).

#### Enicospilus

Stephens, 1835

HENICOSPILUS Agassiz, 1846
ALLOCAMPTUS Förster, 1869
CYMATONEURA Kriechbaumer, 1901
CRYPTOCAMPTUS Brèthes, 1909
AMESOPHILUS Enderlein, 1914

##### Notes

Distribution and taxonomy follow Broad and Shaw (2016)

#### Enicospilus adustus

(Haller, 1885)

Ophion adustus Haller, 1885
merdarius misident.

##### Distribution

England, Scotland, Ireland

##### Notes

*Enicospilus* ‘*merdarius*’ *auctt*. has now been split into three species, none of which is actually *merdarius* (Broad and Shaw 2016); *E. merdarius* was regarded as a synonym of *E. ramidulus* by Gauld (1973) and listed as such in Fitton (1978) but regarded by most recent authors as a valid species, which is borne out by the lack of intermediate specimens and differences in distribution (Broad and Shaw 2016) (allowing for the fact that the name *merdarius* was misappplied).

#### Enicospilus cerebrator

Aubert, 1966

##### Distribution

England

##### Notes

added by Broad and Shaw (2016)

#### Enicospilus combustus

(Gravenhorst, 1829)

Ophion combustus Gravenhorst, 1829

##### Distribution

England, Wales

#### Enicospilus inflexus

(Ratzeburg, 1844)

Ophion inflexus Ratzeburg, 1844

##### Distribution

England, Scotland, Wales

#### Enicospilus merdarius

(Gravenhorst, 1829)

Ophion merdarius Gravenhorst, 1829
repentinus misident.
tournieri (Vollenhoven, 1879, *Ophion*) synonymy by Broad and Shaw (2016)
rossicus (Kokujev, 1907, *Henicospilus*)
contributus Shestakov, 1926

##### Distribution

England, Scotland

##### Notes

see note under *repentinus*

#### Enicospilus myricae

Broad & Shaw, 2016

##### Distribution

England, Scotland, Wales

##### Notes

added by Broad and Shaw (2016)

#### Enicospilus ramidulus

(Linnaeus, 1758)

Ichneumon ramidulus Linnaeus, 1758
truncatus (Poda, 1761, *Sphex*)
instabilis (Kokujev, 1907, *Henicospilus*)

##### Distribution

England, Scotland, Wales, Ireland

#### Enicospilus repentinus

(Holmgren, 1860)

Ophion repentinus Holmgren, 1860

##### Distribution

England

##### Notes

Added by Broad and Shaw (2016); although previously recognised as British (Gauld 1973), this was in error and the specimens are *merdarius* (=*tournieri auctt*.), however, Broad and Shaw (2016) record the true *repentinus* from Britain.

#### Enicospilus undulatus

(Gravenhorst, 1829)

Ophion undulatus Gravenhorst, 1829
arcuatus (Brullé, 1846, *Ophion*)

##### Distribution

England

#### Eremotylus

Förster, 1869

CAMPTONEURA Kriechbaumer, 1901
CAMPTONEUROIDES Strand, 1928
CLISTORAPHA Cushman, 1947

#### Eremotylus curvinervis

(Kriechbaumer, 1878)

Ophion curvinervis Kriechbaumer, 1878
hungaricus Szépligeti, 1905
dryobotae Seyrig, 1926

##### Distribution

England

##### Notes

added by Horstmann (1981b)

#### Eremotylus marginatus

(Jurine, 1807)

Anomalon marginatum Jurine, 1807

##### Distribution

England

##### Notes

a distribution record from George (1957)

#### Ophion

Fabricius, 1798

PANISCUS Schrank, 1802
STENOPHTHALMUS Szépligeti, 1905
PACHYPROTOMA Kohl, 1906
APATOPHION Shestakov, 1926
PLATOPHION Hellén, 1926

##### Notes

The species *areolaris* and *ocellaris* have often been treated as comprising a separate genus, *Platophion* (e.g. Gauld 1973, Brock 1982) but are now generally treated as a species-group of *Ophion* (see Schwarzfeld et al. 2016).

#### Ophion areolaris

Brauns, 1889

##### Distribution

Scotland

#### Ophion brevicornis

Morley, 1915

##### Distribution

England, Scotland, Wales

#### Ophion costatus

Ratzeburg, 1848

##### Distribution

England, Scotland, Wales, Ireland

#### Ophion crassicornis

Brock, 1982

##### Distribution

England, Scotland, Wales

##### Notes

added by Brock (1982)

#### Ophion forticornis

Morley, 1915

baueri Habermehl, 1930

##### Distribution

England, Ireland

#### Ophion longigena

Thomson, 1888

##### Distribution

England, Scotland

#### Ophion luteus

(Linnaeus, 1758)

Ichneumon luteus Linnaeus, 1758
distans Thomson, 1888
slaviceki Kriechbaumer, 1892
pictus Kokujev, 1906
calcaratus Morley, 1915

##### Distribution

England, Scotland, Wales, Ireland, Isle of Man

##### Notes

*slaviceki* was re-synonymised by Brock (1982)

#### Ophion minutus

Kriechbaumer, 1879

eremotyloides Ceballos, 1962

##### Distribution

England, Scotland, Wales

#### Ophion mocsaryi

Brauns, 1889

fossulatus Hedwig, 1957 unavailable

##### Distribution

England, Scotland, Wales, Ireland

#### Ophion obscuratus

Fabricius, 1798

obscurus Fabricius, 1804
polyguttator (Thunberg, 1824, *Ichneumon*)
flavolineatus Brullé, 1846
variegatus Rudow, 1883

##### Distribution

England, Scotland, Wales, Ireland

#### Ophion ocellaris

Ulbricht, 1926

##### Distribution

England, Scotland

#### Ophion parvulus

Kriechbaumer, 1879

##### Distribution

England, Scotland, Wales, Ireland

#### Ophion perkinsi

Brock, 1982

##### Distribution

England, Scotland, Wales

##### Notes

added by Brock (1982)

#### Ophion pteridis

Kriechbaumer, 1879

##### Distribution

England, Scotland, Wales, Ireland

#### Ophion scutellaris

Thomson, 1888

longicornis Brauns, 1889
stigmaticus Morley, 1915

##### Distribution

England, Scotland, Wales

#### Ophion ventricosus

Gravenhorst, 1829

impressus (Thunberg, 1824, *Ichneumon*) preocc.

##### Distribution

England, Scotland, Wales, Ireland

#### Stauropoctonus

Brauns, 1889

STAUROPODOCTONUS Morley, 1913

#### Stauropoctonus bombycivorus

(Gravenhorst, 1829)

Ophion bombycivorus Gravenhorst, 1829
infuscatus (Taschenberg, 1875, *Ophion*)

##### Distribution

England

### Orthocentrinae

#### ORTHOCENTRINAE

Förster, 1869

HELICTINAE Gupta, 1987

##### Notes

Traditionally, the Orthocentrinae comprised a tightly-knit group of genera related to *Orthocentrus* but now includes many genera of the former ‘Microleptinae’ sensu Townes (1971) (Wahl 1990). Distribution data taken from BMNH, NMS (det. J.F. Perkins and GRB), UM, Rossem (1982), Rossem (1983a), Rossem (1987), Rossem (1988) and Dasch (1992), with additional references given.

#### Aniseres

Förster, 1871

#### Aniseres lapponicus

Jussila, 1994

##### Distribution

Scotland

##### Notes

NMS, added here; synonymised *lapponicus* under *pallipes*; I do not follow this as there are two clearly distinct species in Britain and one corresponds to Jussila’s (Jussila 1994b) description of *lapponicus*. However, I have not yet examined type material.

#### Aniseres pallipes

Förster, 1871

pallidipes Dalla Torre, 1901

##### Distribution

England

##### Notes

NMS, added here

#### Aperileptus

Förster, 1869

#### Aperileptus albipalpus

(Gravenhorst, 1829)

Plectiscus albipalpus Gravenhorst, 1829
conformis Förster, 1871
custoditor Förster, 1871
euryzonus Förster, 1871
exstirpator Förster, 1871
frontalis Förster, 1871
fungicola Förster, 1871
impacatus Förster, 1871
penetrans Förster, 1871
placidus Förster, 1871
tutorius Förster, 1871
vacuus Förster, 1871
vittiger Förster, 1871
nigricarpus Strobl, 1904

##### Distribution

England, Scotland

#### Aperileptus impurus

Förster, 1871

electus Förster, 1871
filiventris Förster, 1871
immundus Förster, 1871
inamoenus Förster, 1871
inclinans Förster, 1871
labilis Förster, 1871
languidus Förster, 1871
notabilis Förster, 1871
secretus Förster, 1871
sternoxanthus Förster, 1871
trivittatus Strobl, 1904

##### Distribution

England, Scotland

#### Aperileptus infuscatus

Förster, 1871

##### Distribution

England

##### Notes

BMNH, added here

#### Aperileptus microspilus

Förster, 1871

spoliator Förster, 1871

##### Distribution

England, Scotland

##### Notes

NMS, W.A. Ely coll., added here

#### Aperileptus vanus

Förster, 1871

obliquus (Thomson, 1888, *Plectiscus*)

##### Distribution

England, Scotland, Wales, Ireland

##### Notes

added by Dasch (1992)

#### Apoclima

Förster, 1869

#### Apoclima signaticorne

Förster, 1881

##### Distribution

Scotland

##### Notes

NMS, added here

#### Batakomacrus

Kolarov, 1986

#### Batakomacrus caudatus

(Holmgren, 1858)

Orthocentrus caudatus Holmgren, 1858
crassicaudatus (Kolarov, 1986, *Batakomacrus*) synonymy by Broad (2010)

##### Distribution

England, Scotland, Wales

#### Batakomacrus flaviceps

(Gravenhorst, 1829)

Orthocentrus flaviceps Gravenhorst, 1829

##### Distribution

England

#### Batakomacrus noyesi

Broad, 2010

##### Distribution

England, Scotland

##### Notes

added by Broad (2010)

#### Catastenus

Förster, 1871

CATATENUS misspelling

#### Catastenus femoralis

Förster, 1871

##### Distribution

England, Scotland, Wales

#### Dialipsis

Förster, 1869

PARENTYPOMA Strobl, 1901

#### Dialipsis exilis

Förster, 1871

conjuncta Förster, 1871
diversa Förster, 1871
intermedia Förster, 1871
mesomelana Förster, 1871
observatrix Förster, 1871
pallida Förster, 1871
crassipes (Thomson, 1888, *Plectiscus*)
femorata (Strobl, 1901, *Parentypoma*)

##### Distribution

England, Scotland, Ireland

#### Entypoma

Förster, 1869

ENTELECHIA Förster, 1871
ENTELECHIUS Thomson, 1888
ENTYPOMUS Thomson, 1888

#### Entypoma robustator

Aubert, 1968

##### Distribution

England

##### Notes

BMNH, added here

#### Entypoma robustum

Förster, 1871

##### Distribution

England

#### Entypoma suspiciosum

(Förster, 1871)

Entelechia suspiciosa Förster, 1871
remotum (Marshall, 1896, *Bassus*)

##### Distribution

England, Scotland

#### Eusterinx

Förster, 1869

#### Divinatrix

Rossem, 1987

#### Eusterinx (Divinatrix) inaequalis

Rossem, 1981

##### Distribution

England

##### Notes

NMS, BMNH, det. Schwarz & Broad, added here

#### Eusterinx

Förster, 1869

#### Eusterinx (Eusterinx) argutula

Förster, 1871

ambigua Förster, 1871
divulgata Förster, 1871
intermedia Förster, 1871
scitula Förster, 1871
subcincta Förster, 1871

##### Distribution

England

#### Eusterinx (Eusterinx) obscurella

Förster, 1871

exigua Förster, 1871

#### Eusterinx (Eusterinx) oligomera

Förster, 1871

fulvicincta Förster, 1871
fulvicornis Förster, 1871
moesta Förster, 1871
oreophila Förster, 1871
speculifera Förster, 1871
tenuis Förster, 1871

##### Distribution

England, Ireland

##### Notes

added by Dasch (1992)

#### Holomeristus

Förster, 1869

#### Eusterinx (Holomeristus) refractaria

Rossem, 1982

##### Distribution

Isle of Man

##### Notes

added by Dasch (1992)

#### Eusterinx (Holomeristus) tenuicincta

(Förster, 1871)

Holomeristus tenuicinctus Förster, 1871

##### Distribution

England, Scotland

#### Ischyracis

Förster, 1869

#### Eusterinx (Ischyracis) bispinosa

(Strobl, 1901)

Hemiteles bispinosus Strobl, 1901
alpigena (Strobl, 1904, *Catomicrus*)

##### Distribution

England

##### Notes

added by Dasch (1992)

#### Gnathochorisis

Förster, 1869

LAEPSERUS Förster, 1869
ACROBLAPTICUS Schmiedeknecht, 1911
BLAPTICUS misident.

#### Gnathochorisis crassula

(Thomson, 1888)

Blapticus crassulus Thomson, 1888

##### Distribution

England, Scotland, Ireland

##### Notes

NMS, BMNH, added here

#### Gnathochorisis dentifer

(Thomson, 1888)

Blapticus dentifer Thomson, 1888
debilis (Schmiedeknecht, 1911, *Acroblapticus*)

##### Distribution

England, Scotland

#### Helictes

Haliday, 1837

ENCOPIUS Schiødte, 1839
MYRIARTHRUS Förster, 1869

#### Helictes borealis

(Holmgren, 1857)

Megastylus borealis Holmgren, 1857
clypeatus (Förster, 1871, *Idioxenus*)
coxalis (Förster, 1871, *Idioxenus*)
invalidus (Förster, 1871, *Idioxenus*)
polymerus (Förster, 1871, *Idioxenus*)
propinquus (Förster, 1871, *Idioxenus*)
variator (Förster, 1871, *Idioxenus*)
pilicornis (Thomson, 1888, *Megastylus*)

##### Distribution

England, Scotland, Wales

#### Helictes erythrostoma

(Gmelin, 1790)

Ichneumon erythrostoma Gmelin, 1790
mediator misident.
fulvicornis (Haliday, 1839, *Cryptus*)
conspicuus (Förster, 1871, *Idioxenus*)
inaequalis (Förster, 1871, *Idioxenus*)
inquilinus (Förster, 1871, *Idioxenus*)
intricator (Förster, 1871, *Idioxenus*)
tetraglyptus (Förster, 1871, *Idioxenus*)
nigricoxus Strobl, 1904

##### Distribution

England, Scotland, Wales, Ireland

#### Helictes varius

(Haliday, 1839)

Cryptus varius Haliday, 1839

##### Distribution

Ireland

#### Hemiphanes

Förster, 1869

#### Hemiphanes erratum

Humala, 2007

##### Distribution

Scotland, Wales, Ireland

##### Notes

Added by Humala et al. (2007) on the basis of information from GRB. According to Humala et al. (2007), *Hemiphanes flavipes* Förster, 1871 sensu Rossem is a misidentification; Humala (2007) described this species as new.

#### Hemiphanes gravator

Förster, 1871

laevithorax (Strobl, 1903, *Trematopygus*)

##### Distribution

Scotland

##### Notes

NMS, det. Brock, added here

#### Hemiphanes performidatum

Rossem, 1988

##### Distribution

England, Scotland

##### Notes

Added by Humala et al. (2007) on the basis of information from GRB.

#### Megastylus

Schiødte, 1838

DICOLUS Förster, 1869
IDIOXENUS Förster, 1869
MEGALOSTYLUS Schulz, 1906
MIOMEROIDES Kiss, 1924
MYRIARTHRUS misident.

#### Megastylus cruentator

Schiødte, 1838

mediator Schiødte, 1838
cruentatus (Haliday, 1839, *Cryptus*)
conformis Förster, 1871
fuscicornis Förster, 1871
nigriventris Förster, 1871
pectoralis (Rudow, 1886, *Hemiteles*) preocc.

##### Distribution

England, Scotland, Wales, Ireland

#### Megastylus excubitor

(Förster, 1871)

Dicolus excubitor Förster, 1871

##### Distribution

England, Scotland

#### Megastylus flavopictus

(Gravenhorst, 1829)

Plectiscus flavopictus Gravenhorst, 1829
lineator Schiødte, 1838
cingulator (Förster, 1871, *Myriarthrus*)

##### Distribution

England, Scotland

#### Megastylus impressor

Schiødte, 1838

insectator (Förster, 1871, *Dicolus*)

##### Distribution

England, Scotland, Wales

#### Megastylus orbitator

Schiødte, 1838

maderensis (Wollaston, 1858, *Mesoleptus*)
leptoderus Förster, 1871
pauxillus Förster, 1871
pumilio Förster, 1871
retroligatus Förster, 1871
rufipleuris (Förster, 1871, *Myriarthrus*)

##### Distribution

England, Scotland, Wales, Ireland

##### Notes

added by Rossem (1983b)

#### Megastylus pectoralis

(Förster, 1871)

Dicolus pectoralis Förster, 1871
subtiliventris (Förster, 1871, *Dicolus*)

##### Distribution

England, Scotland, Ireland

#### Megastylus suecicus

Rossem, 1983

##### Distribution

England, Scotland, Ireland

##### Notes

NMS, BMNH, det. Broad, added here

#### Neurateles

Ratzeburg, 1848

#### Neurateles falcatus

(Thomson, 1897)

Orthocentrus falcatus Thomson, 1897

##### Distribution

Scotland

##### Notes

BMNH, added here

#### Neurateles papyraceus

Ratzeburg, 1848

britteni (Waterson, 1929, *Stenomacrus*)

##### Distribution

England

#### Orthocentrus

Gravenhorst, 1829

ATMETUS Förster, 1869
PHAENOSEMUS Förster, 1869
TAPINOPS Förster, 1869

#### Orthocentrus asper

(Gravenhorst, 1829)

Exochus asper Gravenhorst, 1829
discolor Holngren, 1858

##### Distribution

England, Ireland

#### Orthocentrus attenuatus

Holmgren, 1858

##### Distribution

England

#### Orthocentrus corrugatus

Holmgren, 1858

##### Distribution

England

#### Orthocentrus frontator

(Zetterstedt, 1838)

Tryphon frontator Zetterstedt, 1838
repentinus Holmgren, 1858
frontalis Brischke, 1871
pirasii Costa, 1886

##### Distribution

England, Scotland, Ireland

#### Orthocentrus fulvipes

Gravenhorst, 1829

anomalus Gravenhorst, 1829

##### Distribution

England, Scotland, Ireland

#### Orthocentrus marginatus

Holmgren, 1858

##### Distribution

England

#### Orthocentrus monilicornis

Holmgren, 1858

##### Distribution

England, Ireland

#### Orthocentrus petiolaris

Thomson, 1897

##### Distribution

England

#### Orthocentrus protervus

Holmgren, 1858

##### Distribution

England

#### Orthocentrus radialis

Thomson, 1897

##### Distribution

England

#### Orthocentrus sannio

Holmgren, 1858

histrio Holmgren, 1858

##### Distribution

England, Scotland

#### Orthocentrus spurius

Gravenhorst, 1829

protuberans Holmgren, 1858

##### Distribution

England, Scotland

#### Orthocentrus winnertzii

Förster, 1850

stigmaticus Holmgren, 1858 synonymy by Horstmann (2002c)
borealis Roman, 1915
meridionator Aubert, 1960

##### Distribution

England, Scotland

#### Pantisarthrus

Förster, 1871

#### Pantisarthrus dispar

Rossem, 1981

##### Distribution

England

##### Notes

NMS, added here

#### Pantisarthrus lubricus

(Förster, 1871)

Aniseres lubricus Förster, 1871
inaequalis Förster, 1871
ochropus Förster, 1871
pseudochropus Strobl, 1904
subalpinus (Strobl, 1904, *Aniseres*)

##### Distribution

England, Scotland

#### Pantisarthrus luridus

Förster, 1871

##### Distribution

England, Scotland, Ireland

#### Picrostigeus

Förster, 1869

##### Notes

Some distribution data from Horstmann (1994b).

#### Picrostigeus brevicauda

Horstmann, 1994

##### Distribution

England

##### Notes

added by Horstmann (1994b)

#### Picrostigeus debilis

(Gravenhorst, 1829)

Orthocentrus debilis Gravenhorst, 1829

##### Distribution

England, Scotland, Ireland

#### Picrostigeus obscurus

Horstmann, 1994

##### Distribution

England, Scotland, Wales, Ireland

##### Notes

added by Horstmann (1994b)

#### Picrostigeus recticauda

(Thomson, 1897)

Orthocentrus recticauda Thomson, 1897

##### Distribution

England, Scotland, Wales, Ireland

#### Picrostigeus setiger

(Brischke, 1871)

Orthocentrus setiger Brischke, 1871

##### Distribution

England, Ireland

##### Notes

added by Horstmann (1994b)

#### Plectiscidea

Viereck, 1914

#### Fugatrix

Rossem, 1987

#### Plectiscidea (Fugatrix) communis

(Förster, 1871)

Plectiscus communis Förster, 1871
elumbis (Förster, 1871, *Plectiscus*)
gilva (Förster, 1871, *Plectiscus*)
infirma (Förster, 1871, *Plectiscus*)
nigrita (Förster, 1871, *Plectiscus*)
parviceps (Förster, 1871, *Plectiscus*)
tantilla (Förster, 1871, *Plectiscus*)

##### Distribution

England, Scotland, Ireland

#### Plectiscidea

Viereck, 1914

EPHALMATOR Rossem, 1981

##### Notes

species of *Plectiscidea* excluded from the British and Irish list:

[*amicalis* (Förster, 1871, *Plectiscus*); syn. *sodalis* (Förster, 1871, *Plectiscus*)] Listed as *sodalis* by Fitton (1978), presumaby either on the basis of specimens recorded by Carr, which are not now accepted as necessarily British (see note under *Lissonota funebris*), or on the basis of specimens in BMNH identified as *sodalis* var. *moerens*, which is now regarded as a separate species.

#### Plectiscidea (Plectiscidea) aquilonia

Humala, 2003

##### Distribution

England, Wales

##### Notes

BMNH, det. Broad, added here

#### Plectiscidea (Plectiscidea) bistriata

(Thomson, 1888)

Plectiscus bistriatus Thomson, 1888

##### Distribution

England

#### Plectiscidea (Plectiscidea) canaliculata

(Förster, 1871)

Plectiscus canaliculatus Förster, 1871
distincta (Förster, 1871, *Plectiscus*)
subcurvata (Förster, 1871, *Plectiscus*)
subtilis (Förster, 1871, *Plectiscus*)

#### Plectiscidea (Plectiscidea) collaris

(Gravenhorst, 1829)

Plectiscus collaris Gravenhorst, 1829
binodula (Förster, 1871, *Plectiscus*)

##### Distribution

England, Scotland, Wales

#### Plectiscidea (Plectiscidea) conjuncta

(Förster, 1871)

Plectiscus conjunctus Förster, 1871
flavicoxis (Förster, 1871, *Plectiscus*)

##### Distribution

England

##### Notes

NMS, added here

#### Plectiscidea (Plectiscidea) eurystigma

(Thomson, 1888)

Plectiscus eurystigma Thomson, 1888

#### Plectiscidea (Plectiscidea) humeralis

(Förster, 1871)

Plectiscus humeralis Förster, 1871
fulva (Förster, 1871, *Plectiscus*)
hostilis (Förster, 1871, *Plectiscus*)

#### Plectiscidea (Plectiscidea) hyperborea

(Holmgren, 1869)

Plectiscus hyperboreus Holmgren, 1869

##### Notes

The identification of specimens as *hyperborea* away from Svalbard is uncertain (Horstmann 2003b), the identity of specimens recorded as this species is therefore unknown.

#### Plectiscidea (Plectiscidea) melanocera

(Förster, 1871)

Plectiscus melanocerus Förster, 1871
proxima (Förster, 1871, *Plectiscus*)

##### Distribution

England

#### Plectiscidea (Plectiscidea) moerens

(Förster, 1871)

Plectiscus moerens Förster, 1871
eversoria (Förster, 1871, *Plectiscus*)
flavizona (Förster, 1871, *Plectiscus*)
xanthoneuris (Förster, 1871, *Plectiscus*)

##### Distribution

England

##### Notes

BMNH, added here; see note under *Plectiscidea*.

#### Plectiscidea (Plectiscidea) subteres

(Thomson, 1888)

Plectiscus subteres Thomson, 1888

#### Plectiscidea (Plectiscidea) tener

(Förster, 1871)

Plectiscus tener Förster, 1871
incerta (Förster, 1871, *Plectiscus*)

##### Distribution

England

##### Notes

added by Rossem (1988)

#### Plectiscidea (Plectiscidea) tenuicornis

(Förster, 1871)

Plectiscus tenuicornis Förster, 1871
brachyura (Förster, 1871, *Plectiscus*)

##### Distribution

England

#### Plectiscidea (Plectiscidea) terebrator

(Förster, 1871)

Plectiscus terebrator Förster, 1871
habilis (Förster, 1871, *Plectiscus*)
praeposita (Förster, 1871, *Plectiscus*)

##### Distribution

England

#### Plectiscidea (Plectiscidea) ventosa

Rossem, 1987

##### Distribution

Scotland

##### Notes

NMS, added here

#### Plectiscidea (Plectiscidea) zonata

(Gravenhorst, 1829)

Plectiscus zonatus Gravenhorst, 1829
abscondita (Förster, 1871, *Proclitus*)
contemptibilis (Förster, 1871, *Proclitus*)
denticulata (Förster, 1871, *Proclitus*)
displicita (Förster, 1871, *Proclitus*)
humilis (Förster, 1871, *Proclitus*)
inaestimabilis (Förster, 1871, *Proclitus*)
inferior (Förster, 1871, *Proclitus*)
marginata (Förster, 1871, *Proclitus*)
punctata (Förster, 1871, *Proclitus*)
sordida (Förster, 1871, *Proclitus*)
exareolata Aubert, 1979
sodalis (Förster, 1871, *Plectiscus*])

##### Distribution

England

##### Notes

BMNH, added here; transferred from *Proclitus* by Humala (2007). This is a typical species of *Plectiscidea* and there seems to be no reason why Rossem (1983a) transferred it to *Proclitus*.

#### Plectiscus

Gravenhorst, 1829

BREPHOCTONUS Förster, 1869
LEIPAULUS Townes, 1945

#### Plectiscus agilis

(Holmgren, 1858)

Orthocentrus agilis Holmgren, 1858
flavicornis (Thomson, 1897, *Orthocentrus*)

##### Distribution

Ireland

#### Plectiscus impurator

Gravenhorst, 1829

ventralis (Holmgren, 1858, *Orthocentrus*)
vittatus (Holmgren, 1858, *Orthocentrus*)

##### Distribution

England, Scotland, Wales, Ireland

#### Plectiscus ridibundus

(Gravenhorst, 1829)

Orthocentrus ridibundus Gravenhorst, 1829
exilis (Holmgren, 1858, *Orthocentrus*)

##### Distribution

England, Scotland, Wales, Ireland

#### Proclitus

Förster, 1869

CLEPTICUS Haliday, 1839 preocc.
ACLASTONEURA Kriechbaumer, 1896

#### Proclitus attentus

Förster, 1871

fossulatus Förster, 1871
gracilentus Förster, 1871
leptosomus Förster, 1871
melanocephalus Förster, 1871
mesoxanthus Förster, 1871
procerulus Förster, 1871
quaestorius Förster, 1871
sincerus Förster, 1871
stenogaster Förster, 1871
substriatus Förster, 1871
vallidus Förster, 1871

##### Distribution

England, Scotland

#### Proclitus comes

(Haliday, 1839)

Cryptus comes Haliday, 1839
caudiger Förster, 1871
macrurus Förster, 1871
pallens Förster, 1871
perditorius Förster, 1871

##### Distribution

England, Scotland, Ireland

#### Proclitus edwardsi

Roman, 1923

##### Distribution

England

#### Proclitus fulvicornis

Förster, 1871

cupidus Förster, 1871
evacuator Förster, 1871
inquietus Förster, 1871
periculosus Förster, 1871
ruficaudator Aubert, 1963

##### Distribution

England, Ireland

##### Notes

*Plectiscus heterocerus* Thomson, 1888 was removed from synonymy by Humala (2007) and regarded as a valid species of *Proclitus*; it is not known if it occurs in Britain or Ireland.

#### Proclitus paganus

(Haliday, 1839)

Cryptus paganus Haliday, 1839
autumnalis Förster, 1871
clypearis Förster, 1871
conturbator Förster, 1871
curiosus Förster, 1871
dimidiatus Förster, 1871
instigator Förster, 1871
providus Förster, 1871
longitarsis (Thomson, 1888, *Plectiscus*)

##### Distribution

England, Scotland, Ireland

#### Proclitus praetor

(Haliday, 1839)

Cryptus praetor Haliday, 1839
grandis Förster, 1871

##### Distribution

England, Scotland, Wales, Ireland

#### Proclitus socius

(Haliday, 1839)

Cryptus socius Haliday, 1839

##### Distribution

Ireland

##### Notes

BMNH, added here; described from Ireland so not included in the 1978 British checklist, and Fitton (1976) considered the name to be a nomen dubium.

#### Proeliator

Rossem, 1982

#### Proeliator proprius

Rossem, 1982

##### Distribution

England

##### Notes

BMNH, det. Broad, added here

#### Stenomacrus

Förster, 1869

##### Notes

species of *Stenomacrus* excluded from the British and Irish list:

[*affinis* misident.] *Stenomacrus affinis* (Zetterstedt, 1838, *Bassus*) of authors was redescribed as *S. affinitor* Aubert, 1981 as the type of *affinis* is a species of *Hypsicera* (Metopiinae), a junior synonym of *curvator* (Fabricius). Old British records of ‘*Stenomacrus affinis*’ are not valid.

[*exserens* (Thomson, 1897, *Orthocentrus*)] Morley (1911) recorded *Stenomacrus exserens* as a British species but the only two specimens purportedly of this species in BMNH, from C. Morley’s collection, are misidentified *Eusterinx*. There is no other evidence that exserens is a British or Irish species.

#### Stenomacrus binotatus

(Holmgren, 1858)

Orthocentrus binotatus Holmgren, 1858

##### Distribution

England

#### Stenomacrus carbonariae

Roman, 1939

##### Distribution

England

#### Stenomacrus celer

(Holmgren, 1858)

Orthocentrus celer Holmgren, 1858

##### Distribution

England, Scotland

##### Notes

BMNH, added here

#### Stenomacrus cognatus

(Holmgren, 1858)

Orthocentrus cognatus Holmgren, 1858
confinis (Holmgren, 1858, *Orthocentrus*)
tristis (Holmgren, 1858, *Orthocentrus*)

##### Distribution

England

#### Stenomacrus cubiceps

(Thomson, 1897)

Orthocentrus cubiceps Thomson, 1897

##### Distribution

England, Ireland

#### Stenomacrus curvicaudatus

(Brischke, 1871)

Orthocentrus curvicaudatus Brischke, 1871

##### Distribution

England

#### Stenomacrus curvulus

(Thomson, 1897)

Orthocentrus curvulus Thomson, 1897

##### Distribution

England, Wales

##### Notes

BMNH, det. Perkins, Broad, added here

#### Stenomacrus deletus

(Thomson, 1897)

Orthocentrus deletus Thomson, 1897

#### Stenomacrus holmgreni

(Kirchner, 1867)

Orthocentrus holmgreni Kirchner, 1867
lapponicus Horstmann & Yu, 1999 synonymy by Horstmann (2006a)
intermedius (Holmgren, 1858, *Orthocentrus*) preocc.

##### Distribution

England

#### Stenomacrus incisus

(Gravenhorst, 1829)

Orthocentrus incisus Gravenhorst, 1829

#### Stenomacrus innotatus

(Thomson, 1897)

Orthocentrus innotatus Thomson, 1897

#### Stenomacrus laricis

(Haliday, 1839)

Bassus laricis Haliday, 1839
concinnus (Holmgren, 1858, *Orthocentrus*)
fortipes (Thomson, 1897, *Orthocentrus*)

##### Distribution

England, Scotland, Ireland

#### Stenomacrus molestus

(Holmgren, 1858)

Orthocentrus molestus Holmgren, 1858

#### Stenomacrus ochripes

(Holmgren, 1858)

Orthocentrus ochripes Holmgren, 1858

#### Stenomacrus palustris

(Holmgren, 1858)

Orthocentrus palustris Holmgren, 1858

##### Distribution

England

#### Stenomacrus pedestris

(Holmgren, 1869)

Orthocentrus pedestris Holmgren, 1869
reptilis (Marshall, 1877, *Orthocentrus*)

##### Distribution

England

#### Stenomacrus pygmaeus

Horstmann & Yu, 1999

pusillus (Zetterstedt, 1838, *Bassus*)

##### Distribution

England

##### Notes

BMNH, added here

#### Stenomacrus silvaticus

(Holmgren, 1858)

Orthocentrus silvaticus Holmgren, 1858

##### Distribution

England, Scotland

#### Stenomacrus vafer

(Holmgren, 1858)

Orthocentrus vafer Holmgren, 1858

##### Distribution

Scotland

##### Notes

BMNH, added here

#### Symplecis

Förster, 1869

Orthocentrus vafer Holmgren, 1858

#### Symplecis bicingulata

(Gravenhorst, 1829)

Mesoleptus bicingulatus Gravenhorst, 1829
facialis Thomson, 1888
albicoxis (Kiss, 1924, *Rhaestes*) synonymy by Horstmann (2007b)

##### Distribution

England, Scotland, Ireland

##### Notes

*Symplecis leucostoma* (Förster, 1871, *Blapticus*) (with *xanthostoma* Förster, 1871 as a junior synonym) and *alpicola* Förster, 1871 (with *zonaria* Förster, 1871 and *basalis* Brischke, 1880 as junior synonyms) were removed from synonymy and treated as valid species by Humala (2007); it is not yet known whether *leucostoma* or *alpicola* occur in Britain or Ireland; a previous listing of *leucostoma* as a British species rested on a record by Carr (1924), which cannot be considered reliable (see note under *Lissonota funebris*). Irish record from a specimen in the Canadian National Collection (Ottawa), det. GRB.

#### Symplecis breviuscula

Roman, 1923

breviscula misspelling
infavorabilis Rossem, 1981

##### Distribution

England

### Orthopelmatinae

#### ORTHOPELMATINAE

Schmiedeknecht, 1910

#### Orthopelma

Taschenberg, 1865

PROEDRUS Förster, 1869

##### Notes

Distribution data taken from Gauld and Mitchell (1977b) and BMNH.

#### Orthopelma brevicorne

Morley, 1907

##### Distribution

England, Wales

#### Orthopelma mediator

(Thunberg, 1824)

Ichneumon mediator Thunberg, 1824
bedeguaris (Geoffroy, 1785, *Ichneumon*) preocc.
luteolator (Gravenhorst, 1829, *Hemiteles*)
rufinum (Gravenhorst, 1829, *Porizon*)
pavoniae (Rondani, 1877, *Hemiteles*)

##### Distribution

England, Scotland, Wales, Ireland

### Oxytorinae

#### OXYTORINAE

Thomson, 1883

##### Notes

The name Oxytorinae, prior to Wahl (1986), Wahl (1990), has been applied much more widely, encompassing the subfamilies Cylloceriinae, Microleptinae and Orthocentrinae.

#### Oxytorus

Förster, 1869

CALLIDIOTES Förster, 1869
DELOLYTUS Förster, 1869
PANTOPORTHUS Förster, 1869
MESATRACTODES Morley, 1907

#### Oxytorus armatus

Thomson, 1883

##### Distribution

England, Scotland, Wales

#### Oxytorus luridator

(Gravenhorst, 1820)

Ichneumon luridator Gravenhorst, 1820
coxator (Gravenhorst, 1829, *Mesoleptus*)
ventrator (Gravenhorst, 1829, *Mesoleptus*)
properator (Haliday, 1839, *Atractodes*)
varicornis (Holmgren, 1860, *Atractodes*)
longicornis (Habermehl, 1909, *Exolytus*)
nigricoxa (Kiss, 1924, *Callidiotes*)

##### Distribution

England, Scotland, Wales

### Pimplinae

#### PIMPLINAE

Wesmael, 1845

EPHIALTINAE Hellén, 1915

##### Notes

Distribution data from Fitton et al. (1988), O'Connor and Butler (1992) and Shaw (2006a), with further distribution data for the *Polysphincta* genus-group taken from Hudson (1988). Additional distribution references are given. Tribal classification follows Gauld et al. (2002).

#### DELOMERISTINI

Hellén, 1915

PERITHOINI Wahl & Gauld, 1998 synonymy by Gauld et al. (2002)

#### Delomerista

Förster, 1869

##### Notes

Phillips (1997) provides some Scottish records for *novita* and *pfankuchi*.

#### Delomerista borealis

Walkley, 1960

##### Distribution

England

##### Notes

BMNH, det. Broad, added here

#### Delomerista laevis

(Gravenhorst, 1829)

Pimpla laevis Gravenhorst, 1829
suborbitalis (Gravenhorst, 1829, *Lissonota*) synonymy by Horstmann (2001b)
laevifrons (Thomson, 1877, *Pimpla*)
levifrons (Dalla Torre, 1901, *Pimpla*) preocc.

##### Distribution

England

##### Notes

Added by Horstmann (2001b); Fitton et al. (1988) excluded *laevis* from the British list as specimens identified as such were found to be *novita*; Horstmann (2001b) subsequently established that the holotype of *Lissonota suborbitalis*, supposedly taken in Netley, Shropshire, is the true *laevis*.

#### Delomerista mandibularis

(Gravenhorst, 1829)

Pimpla mandibularis Gravenhorst, 1829
albicinctus (Desvignes, 1862, *Ephialtes*) preocc.
desvignesii (Marshall, 1870, *Ephialtes*)

##### Distribution

England

##### Notes

some distribution data from anon. (2001)

#### Delomerista novita

(Cresson, 1870)

Pimpla novita Cresson, 1870
laevis misident.
europa Gupta, 1982

##### Distribution

England, Scotland, Ireland

##### Notes

Gupta (1982) separated European populations as the subspecies *europa*.

#### Delomerista pfankuchi

Brauns, 1905

unicolor (Hedwig, 1959, *Troctocerus*)

##### Distribution

England, Scotland

##### Notes

added by Fitton et al. (1988)

#### Perithous

Holmgren, 1859

HYBOMISCHOS Baltazar, 1961 synonymy by Wahl and Gauld (1998)
HYBOISCHOS misspelling

#### Perithous albicinctus

(Gravenhorst, 1829)

Ephialtes albicinctus Gravenhorst, 1829

##### Distribution

England

##### Notes

added by Brock and Shaw (1997)

#### Perithous divinator

(Rossi, 1790)

Ichneumon divinator Rossi, 1790
ephippiatorius (Dufour & Perris, 1840, *Pimpla*)
amoenus (Rudow, 1881, *Pimpla*)
rubi (Habermehl, 1917, *Itoplectis*)
pimplarius Haupt, 1938

##### Distribution

England, Ireland

##### Notes

some distribution data from Field and Foster (1988)

#### Perithous scurra

(Panzer, 1804)

Ichneumon scurra Panzer, 1804
mediator (Fabricius, 1804, *Pimpla*)
asilatorius (Thunberg, 1824, *Ichneumon*)
modulator (Thunberg, 1824, *Ichneumon*)
senator (Haliday, 1839, *Pimpla*)
decoratus (Ratzeburg, 1848, *Pimpla*)
longiseta Haupt, 1954
moldavicus Constantineanu & Constantineanu, 1968

##### Distribution

England, Scotland, Wales, Ireland

##### Notes

Welsh occurrence from Formstone (1999)

#### Perithous septemcinctorius

(Thunberg, 1824)

Ichneumon septemcinctorius Thunberg, 1824
varius (Gravenhorst, 1829, *Ephialtes*)
marginellatorius (Dufour & Perris, 1840, *Pimpla*)
brunnescens Koornneef, 1951
exiguus Haupt, 1954
meridionator Aubert, 1963
rufatus Constantineanu & Constantineanu, 1968

##### Distribution

England, Ireland

#### Perithous speculator

Haupt, 1954

transsylvanicus Constantineanu & Constantineanu, 1968

##### Distribution

England

##### Notes

added by Shaw (2006a)

#### EPHIALTINI

Hellén, 1915

POLYSPHINCTINI Hellén, 1915 synonymy by Wahl and Gauld (1998)

#### Acrodactyla

Haliday, 1839

BARYPUS Haliday, 1837 preocc.
COLPOMERIA Holmgren, 1859
SYMPHYLUS Förster, 1869 preocc.
POLEMOPHTHORUS Schulz, 1911
PANTOMIMA Rossem, 1990 synonymy by Broad (2004)

##### Notes

O'Connor (2012) confirms the presence of *carinator* and *quadrisculpta* in Ireland.

#### Acrodactyla carinator

(Aubert, 1965)

Colpomeria carinator Aubert, 1965
braconiformis Kolarov, 1990 synonymy by Zwakhals (2006)

##### Distribution

England, Wales, Ireland

##### Notes

added by Shaw (2006a)

#### Acrodactyla degener

(Haliday, 1839)

Pimpla degener Haliday, 1839
hadrodactyla (Förster, 1871, *Symphylus*)
festata (Rossem, 1990, *Pantomima*) synonymy by Broad (2004)

##### Distribution

England, Scotland, Wales, Ireland, Isle of Man

#### Acrodactyla quadrisculpta

(Gravenhorst, 1820)

Ichneumon quadrisculptus Gravenhorst, 1820
laevigata (Holmgren, 1859, *Colpomeria*)

##### Distribution

England, Scotland, Wales, Ireland, Isle of Man

#### Acrodactyla similis

Horstmann, 2011

##### Distribution

England, Scotland, Wales

##### Notes

added by Horstmann (2011b)

#### Acropimpla

Townes, 1960

SELANASPIS Roman, 1910

#### Acropimpla didyma

(Gravenhorst, 1829)

Pimpla didyma Gravenhorst, 1829

##### Distribution

England, Wales, Ireland

#### Clistopyga

Gravenhorst, 1829

HYMENOMACROPYGA Uchida, 1941
ICHNEUMONOGLYPTA Blanchard, 1941

#### Clistopyga canadensis

Provancher, 1880

sauberi Brauns, 1898 synonymy by Bordera et al. (2014)
terebralis Shestakov, 1927

##### Distribution

England

#### Clistopyga incitator

(Fabricius, 1793)

Ichneumon incitator Fabricius, 1793
haemorrhoidalis Gravenhorst, 1829
elegans (Ratzeburg, 1848, *Polysphincta*)
incitatrix Schulz, 1906
excavata (Telenga, 1930, *Polysphincta*)
temporalis Hellén, 1949

##### Distribution

England, Scotland, Wales, Ireland, Isle of Man

#### Clistopyga rufator

Holmgren, 1856

rufatrix Schulz, 1906

##### Distribution

England, Wales

#### Dolichomitus

Smith, 1877

CLOSTEROCERUS Hartig, 1847 preocc.
MESOEPHIALTES Schmiedeknecht, 1906
DICLOSTEROCERUS Viereck, 1914
EXERISTOIDEA Viereck, 1924
TUBERCULEPHIALTES Ozols, 1962
PAUCDOLICHOMITUS Constantineanu & Pisica, 1970

#### Dolichomitus agnoscendus

(Roman, 1939)

Ephialtes agnoscendus Roman, 1939

##### Distribution

England, Wales, Ireland

#### Dolichomitus diversicostae

(Perkins, 1943)

Ephialtes diversicostae Perkins, 1943

##### Distribution

Scotland

#### Dolichomitus imperator

(Kriechbaumer, 1854)

Ephialtes imperator Kriechbaumer, 1854
Dolichomitus imperator ?*adulterator* (Villers, 1789, *Ichneumon*)
Dolichomitus imperator ?*gracilis* (Gmelin, 1790, *Ichneumon*)
Dolichomitus imperator ?*melanopus* (Gmelin, 1790, *Ichneumon*)

##### Distribution

England, Scotland

#### Dolichomitus mesocentrus

(Gravenhorst, 1829)

Ephialtes mesocentrus Gravenhorst, 1829
rex (Kriechbaumer, 1854, *Ephialtes*)
insignis (Habermehl, 1903, *Ephialtes*)
krapinensis (Hensch, 1930, *Ephialtes*)
gaurottii (Gregor, 1941, *Ephialtes*)

##### Distribution

England, Wales

#### Dolichomitus messor

(Gravenhorst, 1829)

Ephialtes messor Gravenhorst, 1829
continuus (Ratzeburg, 1848, *Ephialtes*)
reissigii (Ratzeburg, 1848, *Pimpla*)
pusillus (Ratzeburg, 1852, *Ephialtes*)
heteropus (Thomson, 1888, *Ephialtes*)
simillimus (Hensch, 1930, *Ephialtes*)
zagoriensis (Hensch, 1930, *Ephialtes*)

##### Distribution

England

#### Dolichomitus populneus

(Ratzeburg, 1848)

Ephialtes populneus Ratzeburg, 1848
abbreviatus (Thomson, 1877, *Ephialtes*)

##### Distribution

England

#### Dolichomitus pterelas

(Say, 1829)

Ichneumon pterelas Say, 1829
discrepans (Hensch, 1929, *Ephialtes*)

##### Distribution

England, Ireland

#### Dolichomitus terebrans

(Ratzeburg, 1844)

Pimpla terebrans Ratzeburg, 1844
planifrons (Thomson, 1877, *Ephialtes*)
borealis (Hellén, 1915, *Ephialtes*)
kangasi (Györfi, 1941, *Pimpla*)

##### Distribution

England, Scotland, Wales

#### Dolichomitus tuberculatus

(Geoffroy, 1785)

Ichneumon tuberculatus Geoffroy, 1785
hyalinus (Gmelin, 1790, *Ichneumon*)
leucopterus (Gmelin, 1790, *Ichneumon*)
crispus (Christ, 1791, *Ichneumon*)
fluctuans (Christ, 1791, *Ichneumon*)
parallelus (Thomson, 1888, *Ephialtes*)
dentiventris (Hellén, 1915, *Ephialtes*)
pfefferi (Habermehl, 1917, *Ephialtes*)

##### Distribution

England, Scotland, Wales, Ireland

#### Dreisbachia

Townes, 1962

LAUFEIA Tosquinet, 1903

##### Notes

Synonymised under *Schizopyga* by Gauld and Dubois (2006), Shaw (2006a) disagrees with this synonymy, on the basis of substantial differences in biology.

#### Dreisbachia pictifrons

(Thomson, 1877)

Pimpla pictifrons Thomson, 1877
bridgmani (Bignell, 1894, *Pimpla*)

##### Distribution

England, Scotland, Ireland

#### Endromopoda

Hellén, 1939

#### Endromopoda arundinator

(Fabricius, 1804)

Pimpla arundinator Fabricius, 1804
melanopyga (Gravenhorst, 1829, *Pimpla*)
erythrosoma (Rudown, 1883, *Pimpla*)
arundinatrix (Schulz, 1906, *Pimpla*)
nigricans (Ulbricht, 1913, *Pimpla*) preocc.
culpator (Morley, 1914, *Epiurus*)
crefeldensis (Strand, 1918, *Pimpla*)

##### Distribution

England, Ireland

#### Endromopoda detrita

(Holmgren, 1860)

Pimpla detrita Holmgren, 1860
laevidorsum (Vollenhoven, 1873, *Pimpla*)
brunnea (Brischke, 1880, *Pimpla*)
punctator (Müller, 1766, *Ichneumon*) synonymy by Horstmann (2001b)

##### Distribution

England, Scotland, Wales, Ireland, Isle of Man

#### Endromopoda nigricoxis

(Ulbricht, 1910)

Pimpla nigricoxis Ulbricht, 1910
melanopyga (Ulbricht, 1909, *Pimpla*) preocc.
ulbrichtiana (Strand, 1918, *Pimpla*)

##### Distribution

England, Scotland, Wales, Ireland, Isle of Man

##### Notes

added by Fitton et al. (1988)

#### Endromopoda nitida

(Brauns, 1898)

Pimpla nitida Brauns, 1898
deplanata (Morley, 1908, *Pimpla*)

##### Distribution

England, Wales

#### Endromopoda phragmitidis

(Perkins, 1957)

Ephialtes phragmitidis Perkins, 1957
rufipes (Aubert, 1963, *Scambus*)

##### Distribution

England, Wales

#### Ephialtes

Gravenhorst, 1829

APECHTHIS misident.

#### Ephialtes duplicauda

Heinrich, 1949

spatulata (Townes, 1960, *Pimpla*) synonymy by Horstmann (2008c)

##### Distribution

Ireland

##### Notes

added by Horstmann (2008c)

#### Ephialtes manifestator

(Linnaeus, 1758)

Ichneumon manifestator Linnaeus, 1758
extricator (Villers, 1789, *Ichneumon*)
leucopalpus (Gmelin, 1790, *Ichneumon*)
carbonarius (Christ, 1791, *Ichneumon*) preocc.
gracilis (Schrank, 1802, *Ichneumon*) preocc.
nepotor (Thunberg, 1824, *Ichneumon*)
elongator (Zetterstedt, 1838, *Pimpla*)

##### Distribution

England, Wales

##### Notes

Irish occurrence needs to be confirmed since Horstmann’s confirmation that *duplicauda* occurs there; there are no Irish specimens in BMNH or NMS.

#### Exeristes

Förster, 1869

EREMOCHILA Förster, 1869

#### Exeristes ruficollis

(Gravenhorst, 1829)

Pimpla ruficollis Gravenhorst, 1829
variegatus (Ratzeburg, 1844, *Pimpla*)

##### Distribution

England, Scotland, Wales

#### Flavopimpla

Betrem, 1932

AFREPHIALTES Benoit, 1953

##### Notes

 treated *Afrephialtes* as a junior synonym of *Flavopimpla* whilst retained two separate genera. Fitton et al. (1988) and Yu et al. (2012) have followed but Gauld et al. (2002) found ‘*Flavopimpla*’ to be nested within ‘*Afrephialtes*’ in their morphological phylogeny, which result is followed here.

#### Flavopimpla cicatricosa

(Ratzeburg, 1848)

Pimpla cicatricosa Ratzeburg, 1848

##### Distribution

England, Ireland

##### Notes

Irish occurrence from O'Connor and Shaw (2004) and Allen (2007) documents a recent English record.

#### Fredegunda

Fitton, Shaw & Gauld, 1988

#### Fredegunda diluta

(Ratzeburg, 1852)

Pimpla diluta Ratzeburg, 1852
nigriceps (Taschenberg, 1863, *Pimpla*) preocc.
media (Verhoeff, 1891, *Pimpla*)
taschenbergii (Dalla Torre, 1901, *Pimpla*)

##### Distribution

England, Wales

#### Gregopimpla

Momoi, 1965

#### Gregopimpla inquisitor

(Scopoli, 1763)

Ichneumon inquisitor Scopoli, 1763
Gregopimpla inquisitor ?*visitator* (Poda, 1761, *Ichneumon*)
Gregopimpla inquisitor ?*visitator* (Scopoli, 1763, *Ichneumon*) preocc.
scanica (Geoffroy, 1785, *Ichneumon*)
albipes (Gmelin, 1790, *Ichneumon*) preocc.
annulata (Gmelin, 1790, *Ichneumon*)
aurifrons (Gmelin, 1790, *Ichneumon*)
variegata (Gmelin, 1790, *Ichneumon*) preocc.
perquisitor (Olivier, 1792, *Ichneumon*)
pennator (Fabricius, 1793, *Ichneumon*) preocc.
pinnator (Thunberg, 1824, *Ichneumon*)
flavipes (Gravenhorst, 1829, *Pimpla*)
pini (Hartig, 1838, *Pimpla*)
pudibundae (Ratzeburg, 1848, *Pimpla*)
inquisitrix (Schulz, 1906, *Pimpla*)

##### Distribution

England, Scotland, Ireland

#### Iseropus

Förster, 1869

CNEMOPIMPLA Cameron, 1903

#### Iseropus stercorator

(Fabricius, 1793)

Ichneumon stercorator Fabricius, 1793
graminellae (Schrank, 1802, *Ichneumon*)
mussii (Hartig, 1838, *Pimpla*)
holmgreni (Schmiedeknecht, 1888, *Pimpla*)

##### Distribution

England, Scotland, Wales, Ireland

#### Liotryphon

Ashmead, 1900

LIOGASTER Kriechbaumer, 1890 preocc.
APISTES Seyrig, 1927 preocc.
APISTEPHIALTES Seyrig, 1928
NEOEPHIALTES Constantineanu & Pisica, 1970

##### Notes

species of *Liotryphon* excluded from the British and Irish list:

[*strobilellae* (Linnaeus, 1758, *Ichneumon*); syn. *resinosus* (Retzius, 1783, *Ichneumon*); *strobilator* (Thunberg, 1824, *Ichneumon*); *glabratus* (Ratzeburg, 1852, *Ephialtes*); *zhedenevensis* (Shestakov, 1927, *Ephialtes*); *discedens* (Hensch, 1930, *Ephialtes*)] As discussed in Fitton et al. (1988), only one 19th century record, and not certainly of British or Irish specimens.

#### Liotryphon ascaniae

(Rudow, 1883)

Ephialtes ascaniae Rudow, 1883
ruficollis (Desvignes, 1856, *Ephialtes*) invalid
sanguinicollis (Brauns, 1901, *Ephialtes*)
perversus (Seyrig, 1927, *Apistes*)

##### Distribution

England, Scotland

#### Liotryphon caudatus

(Ratzeburg, 1848)

Pimpla caudata Ratzeburg, 1848
brevivalvis (Hensch, 1929, *Ephialtes*)
incertus (Hensch, 1929, *Ephialtes*)
foveolatus (Constantineanu & Pisica, 1970, *Neoephialtes*) preocc.

##### Distribution

England, Scotland

#### Liotryphon crassiseta

(Thomson, 1877)

Ephialtes crassiseta Thomson, 1877
pleuralis (Thomson, 1877, *Ephialtes*)
musculus (Kriechbaumer, 1889, *Ephialtes*)
albispiculus (Morley, 1908, *Ephialtes*)
taschenbergi (Ulbricht, 1909, *Ephialtes*) preocc., unavailable
sternoleucus (Lange, 1911, *Ephialtes*)
foveolatus (Ulbricht, 1912, *Ephialtes*)
taschenbergella (Strand, 1918, *Pimpla*)
rufipes (Hensch, 1930, *Ephialtes*)

##### Distribution

England, Ireland

##### Notes

some distribution data from Godfrey and Whitehead (2001)

#### Liotryphon punctulatus

(Ratzeburg, 1848)

Pimpla punctulata Ratzeburg, 1848
discolor (Brischke, 1880, *Ephialtes*)
macrurus (Förster, 1888, *Epiurus*)
longulus (Kriechbaumer, 1890, *Liogaster*)
tener (Hensch, 1929, *Ephialtes*)
vernalis (Hensch, 1929, *Ephialtes*)
gracilentus (Hensch, 1930, *Ephialtes*)

##### Distribution

England

#### Megaetaira

Gauld & Dubois, 2006

##### Notes

The one included species was transferred from *Acrodactyla* by Gauld and Dubois (2006).

#### Megaetaira madida

(Haliday, 1839)

Pimpla madida Haliday, 1839
clypeata (Holmgren, 1860, *Polysphincta*)

##### Distribution

England, Scotland, Ireland

#### Oxyrrhexis

Förster, 1869

#### Oxyrrhexis carbonator

(Gravenhorst, 1807)

Cryptus carbonator Gravenhorst, 1807
Oxyrrhexis carbonator *velata* (Hartig, 1838, *Polysphincta*)
Oxyrrhexis carbonator *pusilla* (Fonscolombe, 1854, *Polysphincta*)
Oxyrrhexis carbonator *carbonatrix* (Schulz, 1906, *Acrodactyla*)

##### Distribution

England

##### Notes

Added by Shaw (1998); previously excluded from the British list by Fitton et al. (1988): see Shaw (2006a).

#### Paraperithous

Haupt, 1954

GNATHAULAX Townes, 1964

#### Paraperithous gnathaulax

(Thomson, 1877)

Ephialtes gnathaulax Thomson, 1877
luteipes (Thomson, 1877, *Ephialtes*)
ruficollis (Rudow, 1881, *Ephialtes*) preocc.
aterrimus (Haupt, 1954, *Perithous*)
moldavicus Constantineanu & Pisica, 1970

##### Distribution

England, Scotland

#### Piogaster

Perkins, 1958

#### Piogaster albina

Perkins, 1958

##### Distribution

England

#### Piogaster punctulata

Perkins, 1958

##### Distribution

England

#### Polysphincta

Gravenhorst, 1829

#### Polysphincta boops

Tschek, 1869

eltshaninovi Shestakov, 1927

##### Distribution

England, Scotland

#### Polysphincta longa

Kasparyan, 1976

##### Distribution

England, Wales

##### Notes

Added by Fritzén and Shaw (2014), from Wales, with an English specimen in BMNH subsequently identified by GRB.

#### Polysphincta rufipes

Gravenhorst, 1829

drewseni Holmgren, 1860

##### Distribution

England, Scotland, Wales, Ireland

#### Polysphincta tuberosa

Gravenhorst, 1829

taschenbergi Woldstedt, 1877
sculpturata Roman, 1931

##### Distribution

England, Scotland, Wales, Ireland, Isle of Man

#### Polysphincta vexator

Fitton, Shaw & Gauld, 1988

##### Distribution

England, Wales, Ireland

##### Notes

added by Fitton et al. (1988)

#### Reclinervellus

He & Ye, 1998

#### Reclinervellus nielseni

(Roman, 1923)

Polysphincta nielseni Roman, 1923

##### Distribution

England

##### Notes

Added by Fitton et al. (1988); transferred from *Polysphincta* by Gauld and Dubois (2006).

#### Scambus

Hartig, 1838

EPIURUS Förster, 1869
TROMERA Förster, 1869
TROCTOCERUS Woldstedt, 1877
ATELEOPHADNUS Cameron, 1905
PSEUDOPOEMENIA Kiss, 1924
ERYTHROSCAMBUS Walley, 1930
LISSOSCAMBUS Walley, 1930

#### Scambus brevicornis

(Gravenhorst, 1829)

Pimpla brevicornis Gravenhorst, 1829
concolor (Ratzeburg, 1848, *Pimpla*) preocc.
nigriscaposus (Thomson, 1877, *Pimpla*)
punctiventris (Thomson, 1877, *Pimpla*)
agilis (Förster, 1888, *Epiurus*)
centaureae (Förster, 1888, *Epiurus*) synonymy by Horstmann (2010b)
depositor (Förster, 1888, *Epiurus*)
infestus (Förster, 1888, *Epiurus*)
anomalus (Morley, 1906, *Phthorimus*)
tibialis (Ulbricht, 1910, *Pimpla*) unavailable
puniceus (Schmiedeknecht, 1914, *Pimpla*)
pratensis (Pfankuch, 1921, *Pimpla*) unavailable
terrestris (Pfankuch, 1921, *Pimpla*) unavailable
ribesii (Hensch, 1929, *Pimpla*)

##### Distribution

England, Scotland, Wales, Ireland, Isle of Man

##### Notes

Horstmann (2010a) split up brevicornis but none of the additional species have yet been found in Britain or Ireland.

#### Scambus buolianae

(Hartig, 1838)

Pimpla buolianae Hartig, 1838
triangularis (Verhoeff, 1890, *Pimpla*)
flavotrochanteratus (Pfeffer, 1913, *Pimpla*)

##### Distribution

England, Scotland

#### Scambus calobatus

(Gravenhorst, 1829)

Pimpla calobata Gravenhorst, 1829
planatus (Hartig, 1838, *Pimpla*) synonymy by Shaw et al. (2011)
ghilianii (Spinola, 1843, *Pimpla*)
nucum (Ratzeburg, 1844, *Pimpla*)
longiventris (Ratzeburg, 1848, *Pimpla*)
cingulatus (Ratzeburg, 1852, *Pimpla*)
ventricosus (Tschek, 1871, *Pimpla*) synonymy by Shaw et al. (2011)
gallicola (Giraud, 1872, *Pimpla*)
stramentarius (Kriechbaumer, 1890, *Pimpla*)
zonatus (Habermehl, 1903, *Pimpla*) preocc.
calobatarius (Kokujev, 1913, *Pimpla*)
zonatellus (Schmiedeknecht, 1914, *Pimpla*)
nigricoxis (Habermehl, 1918, *Epiurus*)

##### Distribution

England

##### Notes

Horstmann (2009c) recognised *ventricosus* as a separate species, occurring in Britain, but Shaw et al. (2011) subsequently demonstrated that these individuals, together with the previously recognised species *planatus*, are seasonal forms of *calobatus*.

#### Scambus cincticarpus

(Kriechbaumer, 1895)

Pimpla cincticarpus Kriechbaumer, 1895
affinis (Habermehl, 1903, *Pimpla*)
divergens (Hensch, 1929, *Pimpla*)

##### Distribution

England

##### Notes

added by Fitton et al. (1988)

#### Scambus elegans

(Woldstedt, 1877)

Troctocerus elegans Woldstedt, 1877
albicrus (Rondani, 1877, *Ephialtes*)
cingulatellus (Costa, 1885, *Pimpla*)
erythronotus (Förster, 1888, *Epiurus*)
ulicicida (Morley, 1911, *Pimpla*)
cottei (Seyrig, 1926, *Pimpla*)
dumeticola (Hensch, 1929, *Pimpla*)
zagoriensis (Hensch, 1929, *Troctocerus*)

##### Distribution

England, Wales, Ireland

#### Scambus eucosmidarum

(Perkins, 1957)

Ephialtes eucosmidarum Perkins, 1957

##### Distribution

England, Scotland, Wales, Ireland

#### Scambus foliae

(Cushman, 1938)

Epiurus foliae Cushman, 1938

##### Distribution

England, Scotland, Isle of Man

##### Notes

added by Fitton et al. (1988)

#### Scambus inanis

(Schrank, 1802)

Ichneumon inanis Schrank, 1802
agilis (Förster, 1888, *Epiurus*)
depositor (Förster, 1888, *Epiurus*)
distinctus (Förster, 1888, *Epiurus*)
annulatus (Kiss, 1924, *Pseudopoemenia*)
lativentris (Ulbricht, 1926, *Epiurus*)
trilobatus (Keler, 1937, *Pimpla*)

##### Distribution

England, Scotland, Wales, Ireland

##### Notes

Added by Fitton et al. (1988); synonymy follows Horstmann (2005b).

#### Scambus nigricans

(Thomson, 1877)

Pimpla nigricans Thomson, 1877
similis (Bridgman, 1884, *Pimpla*)
fulvus (Szépligeti, 1898, *Pimpla*)
lucens (Szépligeti, 1898, *Pimpla*)
interruptecallosus (Strobl, 1902, *Pimpla*)
kriechbaumeri (Habermehl, 1903, *Pimpla*) preocc.
habermehli (Schmiedeknecht, 1908, *Pimpla*)
robustus (Morley, 1908, *Pimpla*) preocc.
obscuripes (Hensch, 1929, *Pimpla*)
singularis (Hensch, 1929, *Pimpla*)
sparsator Aubert, 1965

##### Distribution

England, Scotland, Wales, Ireland, Isle of Man

#### Scambus pomorum

(Ratzeburg, 1848)

Pimpla pomorum Ratzeburg, 1848

##### Distribution

England, Scotland, Ireland

#### Scambus sagax

(Hartig, 1838)

Pimpla sagax Hartig, 1838
linearis (Ratzeburg, 1844, *Pimpla*)
atrocoxatus (Pfeffer, 1913, *Pimpla*)
suecicus (Roman, 1917, *Epiurus*)
sanctacrucianus (Glowacki, 1967, *Ephialtes*)

##### Distribution

England, Scotland

#### Scambus signatus

(Pfeffer, 1913)

Pimpla signata Pfeffer, 1913

##### Distribution

England, Scotland

##### Notes

Originally established as British by Perkins (1943) then excluded from the British list by Fitton et al. (1988); Horstmann (2005b) confirmed that some British specimens are indeed *signatus*; see Shaw (2006a).

#### Scambus tenthredinum

(Goeze, 1776)

Ichneumon tenthredinum Goeze, 1776

##### Distribution

England, Scotland

##### Notes

added by Horstmann (2005b); see Shaw (2006a)

#### Scambus vesicarius

(Ratzeburg, 1844)

Pimpla vesicaria Ratzeburg, 1844
cryptocampi (Boie, 1857, *Pimpla*)
gallicolus (Morley, 1908, *Pimpla*) preocc.
ruficoxis (Ulbricht, 1909, *Pimpla*) unavailable
rhenanus (Ulbricht, 1910, *Pimpla*) unavailable
salicola (Hensch, 1929, *Pimpla*)
morleyi (Schmiedeknecht, 1934, *Pimpla*)

##### Distribution

England, Scotland, Ireland

#### Schizopyga

Gravenhorst, 1829

AFROSPHINCTA Benoit, 1953
SCHIZOPYGOIDES Kasparyan, 1976

#### Schizopyga circulator

(Panzer, 1800)

Ichneumon circulator Panzer, 1800
analis Gravenhorst, 1829
circulatrix Schulz, 1906

##### Distribution

England, Scotland, Wales, Ireland

#### Schizopyga frigida

Cresson, 1870

atra Kriechbaumer, 1887

##### Distribution

England, Scotland, Wales, Ireland

#### Schizopyga podagrica

Gravenhorst, 1829

minuta Gravenhorst, 1829
silbernageli (Kiss, 1933, *Polysphincta*) preocc.

##### Distribution

England, Scotland, Ireland

#### Schizopyga varipes

Holmgren, 1856

##### Distribution

Ireland

##### Notes

Added by Shaw (2006a); incorrectly listed as a synonym of *flavifrons* Holmgren, 1856 by Fitton et al. (1988) and as a synonym of *podagrica* by Yu and Horstmann (1997) (Shaw 2006a).

#### Sinarachna

Townes, 1960

#### Sinarachna nigricornis

(Holmgren, 1860)

Polysphincta nigricornis Holmgren, 1860
caudata (Thomson, 1888, *Polysphincta*)

##### Distribution

England, Scotland

##### Notes

added by Fitton et al. (1988)

#### Sinarachna pallipes

(Holmgren, 1860)

Polysphincta pallipes Holmgren, 1860

##### Distribution

England, Ireland

##### Notes

Irish occurrence from O'Connor (2004b)

#### Townesia

Ozols, 1962

#### Townesia tenuiventris

(Holmgren, 1860)

Ephialtes tenuiventris Holmgren, 1860
geniculata (Brischke, 1865, *Ephialtes*)
antefurcalis (Thomson, 1877, *Ephialtes*)
gracilis (Hensch, 1930, *Ephialtes*)

##### Distribution

England, Scotland, Ireland

#### Tromatobia

Förster, 1869

AUSTROPIMPLA Brèthes, 1913

#### Tromatobia forsiusi

(Hellén, 1915)

Polysphincta forsiusi Hellén, 1915

##### Distribution

Scotland

##### Notes

added by Fitton et al. (1988)

#### Tromatobia lineatoria

(Villers, 1789)

Ichneumon lineatorius Villers, 1789
oculatoria misident. (Horstmann 2001a)
tipulatoria (Thunberg, 1824, *Ichneumon*)
balanini (Rudow, 1883, *Ephialtes*)
multipicta (Kiss, 1924, *Pimpla*)
sanguinolenta (Kiss, 1924, *Pimpla*)
rufiventris Hellén, 1949
amoena (Haupt, 1954, *Pimpla*) preocc.
orbitalis (Haupt, 1954, *Pimpla*) preocc.

##### Distribution

England, Scotland, Wales, Ireland, Isle of Man

##### Notes

Although the name *oculatoria* (Fabricius, 1798, *Ichneumon*) has usually been used for this species (e.g. Fitton et al. 1988), the type of *oculatoria* is actually a species of *Lissonota* (Horstmann 2001a) so this species has to take the name *lineatoria*.

#### Tromatobia ornata

(Gravenhorst, 1829)

Pimpla ornata Gravenhorst, 1829
soror (Ratzeburg, 1848, *Polysphincta*)
arachnicida Förster, 1888 synonymy by Horstmann (2000c)
concors (Kriechbaumer, 1894, *Pimpla*)
semivaria (Kriechbaumer, 1894, *Pimpla*)
tricolor (Kriechbaumer, 1894, *Pimpla*) preocc.
kriechbaumeri (Dalla Torre, 1901, *Pimpla*)

##### Distribution

England

#### Tromatobia ovivora

(Boheman, 1821)

Pimpla ovivora Boheman, 1821
armillatoria (Thunberg, 1824, *Ichneumon*)
vexatoria (Thunberg, 1824, *Ichneumon*)
angens (Gravenhorst, 1829, *Pimpla*)
parallela (Thomson, 1877, *Pimpla*)
rufipleura (Bignell, 1899, *Pimpla*)
albipes (Brischke, 1891, *Pimpla*) preocc.
contraria Förster, 1888 synonymy by Horstmann (2000c)
evacuans Förster, 1888 synonymy by Horstmann (2000c)
brischkei (Dalla Torre, 1901, *Pimpla*)
obscurata (Ulbricht, 1910, *Pimpla*) preocc., unavailable
rugulosa (Morley, 1914, *Apechtis*)
obscurascens (Strand, 1918, *Pimpla*)
simulans (Hensch, 1929, *Pimpla*)

##### Distribution

England, Scotland, Wales, Ireland, Isle of Man

#### Tromatobia variabilis

(Holmgren, 1856)

Pimpla variabilis Holmgren, 1856
abdominalis (Brullé, 1846,) preocc.
epeirae (Bignell, 1893, *Pimpla*)
hibernica (Morley, 1908, *Pimpla*)
ruficoxa (Kokujev, 1913, *Pimpla*)
inornata (Ulbricht, 1926, *Pimpla*)

##### Distribution

England, Wales, Ireland

#### Zaglyptus

Förster, 1869

#### Zaglyptus multicolor

(Gravenhorst, 1829)

Polysphincta multicolor Gravenhorst, 1829
fairmairii (Laboulbene, 1858, *Pimpla*)
ephippium (Rudow, 1883, *Pimpla*) preocc.
moldavicus (Costantineanu, 1929, *Pimpla*)
rufus Aubert, 1959

##### Distribution

England

#### Zaglyptus varipes

(Gravenhorst, 1829)

Polysphincta varipes Gravenhorst, 1829
tricingulatus (Gravenhorst, 1829, *Schizopyga*)
cingulatus (Kriechbaumer, 1894, *Pimpla*) preocc.
variipes Dalla Torre, 1901 preocc.
rufithorax (Habermehl, 1917, *Polysphincta*)
silbernageli (Kiss, 1926, *Polysphincta*)

##### Distribution

England, Scotland, Wales, Ireland

#### Zatypota

Förster, 1869

POLYSPHINCTOPSIS Habermehl, 1917
LYCORINOPSIS Haupt, 1954

##### Notes

species of *Zatypota* excluded from the British and Irish list by Fitton et al. (1988)

[*anomala* (Holmgren, 1860, *Polysphincta*); syn. *minor* (Kolarov, 1982, *Sinarachna*): Zwakhals (2006)] usually treated as a species of *Sinarachna* (e.g. Fitton et al. 1988, Yu and Horstmann 1997) but transferred to *Zatypota* by Zwakhals (2006) (and, independently, by Gauld and Dubois 2006).

#### Zatypota albicoxa

(Walker, 1874)

Glypta albicoxa Walker, 1874
colorata (Rudow, 1883, *Pimpla*)
eximia (Schmiedeknecht, 1907, *Polysphincta*)
nigriventris (Habermehl, 1917, *Polysphinctopsis*)

##### Distribution

England

##### Notes

added by Hudson (1985)

#### Zatypota bohemani

(Holmgren, 1860)

Polysphincta bohemani Holmgren, 1860

##### Distribution

England, Scotland, Wales, Isle of Man

#### Zatypota discolor

(Holmgren, 1860)

Polysphincta discolor Holmgren, 1860
thoracica (Brischke, 1864, *Polysphincta*)

##### Distribution

England, Scotland, Ireland

##### Notes

Irish occurrence from O'Connor (2004b)

#### Zatypota percontatoria

(Müller, 1776)

Ichneumon percontatorius Müller, 1776
phoenicea (Haliday, 1839, *Pimpla*)
gracilis (Holmgren, 1860, *Polysphincta*) synonymy by Horstmann (2000c)
scutellaris (Holmgren, 1860, *Polysphincta*)
pulchrator (Thomson, 1877, *Polysphincta*)
pulchratrix (Schulz, 1906, *Polysphincta*)
decorata (Haupt, 1954, *Lycorinopsis*)
rhombifer (Haupt, 1954, *Lycorinopsis*)

##### Distribution

England, Scotland, Wales

#### PIMPLINI

Wesmael, 1845

THERONIINI Cushman & Rohwer, 1920

#### Apechthis

Förster, 1869

EPHIALTES Schrank, 1802 nom. ob.
APECHTIS Thomson, 1889
PARAPECHTHIS Blanchard, 1936
TAIWATHERONIA Sonan, 1936

#### Apechthis compunctor

(Linnaeus, 1758)

Ichneumon compunctor Linnaeus, 1758
brassicariae (Poda, 1761, *Ichneumon*)
cunctator (Scopoli, 1763, *Ichneumon*)
annulosa (Gmelin, 1790, *Ichneumon*)
cylindrica (Gmelin, 1790, *Ichneumon*) preocc.
melanoxantha (Gmelin, 1790, *Ichneumon*)
imminuitor (Christ, 1791, *Ichneumon*) synonymy by Horstmann (2000c)
vigilans (Christ, 1791, *Ichneumon*) synonymy by Horstmann (2000c)
varicornis (Fabricius, 1793, *Ichneumon*)
conjunctor (Panzer, 1804, *Ichneumon*)
varicator (Thunberg, 1824, *Ichneumon*)
lativentris (Rudow, 1881, *Pimpla*)
rufipes (Rudow, 1883, *Pimpla*)

##### Distribution

England, Wales

#### Apechthis quadridentata

(Thomson, 1887)

Pimpla quadridentata Thomson, 1887
resinator misident.

##### Distribution

England, Scotland, Wales, Ireland

#### Apechthis rufata

(Gmelin, 1790)

Ichneumon rufatus Gmelin, 1790
flavonotata (Holmgren, 1860, *Pimpla*)
rufithorax (Strobl, 1902, *Pimpla*)
pectoralis (Ulbricht, 1909, *Pimpla*) unavailable

##### Distribution

England, Scotland, Wales, Ireland

#### Itoplectis

Förster, 1869

NESOPIMPLA Ashmead, 1906
EXERISTESOIDES Uchida, 1928

#### Itoplectis alternans

(Gravenhorst, 1829)

Pimpla alternans Gravenhorst, 1829
examinanda (Ratzeburg, 1852, *Pimpla*)
tricolor (Ratzeburg, 1852, *Pimpla*) preocc.
tricincta (Thomson, 1877, *Pimpla*) preocc.
spiracularis (Morley, 1908, *Pimpla*)
ruficoxis (Ulbricht, 1916, *Pimpla*) unavailable

##### Distribution

England, Scotland, Wales, Ireland

#### Itoplectis aterrima

Jussila, 1965

kolthoffi misident.
enslini (Ulbricht, 1916, *Pimpla*) preocc., unavailable
ultimator Aubert, 1966

##### Distribution

England, Scotland, Wales, Ireland

##### Notes

added by Fitton et al. (1988)

#### Itoplectis clavicornis

(Thomson, 1889)

Pimpla clavicornis Thomson, 1889
curticauda misident.

##### Distribution

England, Scotland, Ireland

#### Itoplectis enslini

(Ulbricht, 1911)

Pimpla enslini Ulbricht, 1911
insignis misident. (Shaw 2006a)
ignalinoensis (Strand, 1918, *Pimpla*)
griseanae Perkins, 1957

##### Distribution

England, Scotland

##### Notes

added by Fitton et al. (1988)

#### Itoplectis maculator

(Fabricius, 1775)

Ichneumon maculator Fabricius, 1775
arlequinata (Geoffroy, 1785, *Ichneumon*)
plaesseus (Geoffroy, 1785, *Ichneumon*)
scanica (Villers, 1789, *Ichneumon*) preocc.
laetatoria (Thunberg, 1824, *Ichneumon*)
vincta (Vollenhoven, 1873, *Pimpla*)
maculatrix (Schulz, 1906, *Pimpla*)

##### Distribution

England, Scotland, Wales, Ireland, Isle of Man

#### Itoplectis melanocephala

(Gravenhorst, 1829)

Pimpla melanocephala Gravenhorst, 1829
ephippium (Brullé, 1846, *Pimpla*)
bicolor (Boie, 1855, *Pimpla*) preocc.
ragusae (De Stefani, 1885, *Pimpla*)
cleopatra (Schmiedeknecht, 1897, *Pimpla*)
burtoni (Morley, 1946, *Pimpla*)

##### Distribution

England, Wales

#### Itoplectis viduata

(Gravenhorst, 1829)

Pimpla viduata Gravenhorst, 1829
atrocoxalis (Cresson, 1870, *Pimpla*)
ovalis (Thomson, 1877, *Pimpla*)
meridionalis (Kriechbaumer, 1887, *Pimpla*)
annulata (Ulbricht, 1911, *Pimpla*) unavailable

##### Distribution

England

##### Notes

added by Shaw (2006a)

#### Pimpla

Fabricius, 1804

COCCYGOMIMUS Saussure, 1892
HABROPIMPLA Cameron, 1900
LISSOTHERONIA Cameron, 1905
PHYTODIAETOIDES Morley, 1913
PIMPLIDEA Viereck, 1914
COELOPIMPLA Brèthes, 1916
DIHYBOPLAX Enderlein, 1919
LIOTHERONIA Enderlein, 1919
NEOGABUNIA Brèthes, 1927
OPODACTYLA Seyrig, 1932
OXYPIMPLA Noskiewicz & Chudoba, 1951
JAMAICAPIMPLA Mason, 1975

##### Notes

doubtfully placed species of Pimpla:

[*cossivora* (Curtis, 1826, *Lissonota*) nom. dub.]

#### Pimpla aethiops

Curtis, 1828

aterrima Gravenhorst, 1829
parnarae Viereck, 1912

##### Distribution

England

#### Pimpla arctica

Zetterstedt, 1838

heraclii Boie, 1855
coxator Ruthe, 1859
heraclei Dalla Torre, 1901
coxatrix Schulz, 1906

##### Distribution

Scotland

#### Pimpla contemplator

(Müller, 1776)

Ichneumon contemplator Müller, 1776
geniculata (Geoffroy, 1785, *Ichneumon*)
rufistigma Morley, 1908
rufitibia Morley, 1908

##### Distribution

England, Scotland, Wales, Ireland, Isle of Man

#### Pimpla flavicoxis

Thomson, 1877

##### Distribution

England, Scotland, Wales, Ireland, Isle of Man

##### Notes

*Pimpla aquilonia* Cresson, 1870, is a possible senior synonym of *flavicoxis* (e.g. Oehlke 1964) but is currently classified as a separate species (e.g. Kasparyan 1974, Fitton et al. 1988).

#### Pimpla insignatoria

(Gravenhorst, 1807)

Cryptus insignatorius Gravenhorst, 1807
mixta Ratzeburg, 1848
coxalis Habermehl, 1917
scutellaris Habermehl, 1917 preocc.
conmixta Kiss, 1929

##### Distribution

England, Scotland, Wales, Ireland, Isle of Man

##### Notes

Added by Horstmann (2000c), who lists synonymy; *Pimpla insignatoria* has, until recently, been confused under *flavicoxis* in Britain (Shaw 2006a), although Kasparyan (1974) differentiated the two species (using the name *conmixta* for *insignatoria*).

#### Pimpla melanacrias

Perkins, 1941

geniculata Hensch, 1929 preocc., invalid

##### Distribution

England, Scotland, Wales, Ireland, Isle of Man

#### Pimpla rufipes

(Miller, 1759)

Ichneumon rufipes Miller, 1759
hypochondriaca (Retzius, 1783, *Ichneumon*) synonymy by Horstmann (1999a)
compunctor (Geoffroy, 1785, *Ichneumon*) preocc.
inguinalis (Geoffroy, 1785, *Ichneumon*)
instigator (Fabricius, 1793, *Ichneumon*) preocc.
intermedia Holmgren, 1860
aegyptiaca Schmiedeknecht, 1897
instigatrix Schulz, 1906
scutellaris Ulbricht, 1909 preocc., unavailable
sibirica Meyer, 1926

##### Distribution

England, Scotland, Wales, Ireland, Isle of Man

#### Pimpla sodalis

Ruthe, 1859

cheloniae Giraud, 1869 synonymy by Horstmann (2001b)
nordenskioldii Holmgren, 1872
longiceps Thomson, 1877

##### Distribution

Scotland

#### Pimpla spuria

Gravenhorst, 1829

bilineata Brullé, 1846
strigipleuris Thomson, 1877 synonymy by Shaw (2006b)
dubitata Pérez, 1895
nilotica Schmiedeknecht, 1914
turionelloides Aubert, 1959

##### Distribution

England, Scotland, Wales, Ireland, Isle of Man

#### Pimpla turionellae

(Linnaeus, 1758)

Ichneumon turionellae Linnaeus, 1758
variegata (Schrank, 1785, *Ichneumon*)
leucogonos (Gmelin, 1790, *Ichneumon*)
rufescens (Gmelin, 1790, *Ichneumon*) preocc.
examinator (Fabricius, 1804, *Cryptus*)
cingulator (Thunberg, 1824, *Ichneumon*)
turionator (Thunberg, 1824, *Ichneumon*)
opacellata Desvignes, 1868
examinatrix Schulz, 1906
pubescens Hellén, 1915
padellae Torka, 1918
rufoannula Schmiedeknecht, 1934
freyi Hellén, 1949
variegata Constantineanu, 1954 preocc.

##### Distribution

England, Scotland, Wales, Ireland

#### Pimpla wilchristi

Fitton, Shaw & Gauld, 1988

##### Distribution

England, Scotland, Wales, Isle of Man

##### Notes

Added by Fitton et al. (1988); incorrectly synonymised with *strigipleuris* Thomson (a junior synonym of *spuria*) by Hedström (1990) (Shaw 2006b).

#### Theronia

Holmgren, 1859

PSEUDACOENITES Kriechbaumer, 1892
POECILOPIMPLA Cameron, 1903
ERYTHROTHERONIA Cameron, 1905
ORIENTOTHERONIA Morley, 1913

#### Theronia atalantae

(Poda, 1761)

Ichneumon atalantae Poda, 1761
speculator (Scopoli, 1763, *Ichneumon*)
acuminator (Müller, 1776, *Ichneumon*)
melanops (Schrank, 1781, *Ichneumon*)
nigroculus (Schrank, 1781, *Ichneumon*)
quadripunctata (Schrank, 1781, *Ichneumon*)
vincta (Schrank, 1781, *Ichneumon*)
scutellata (Geoffroy, 1785, *Ichneumon*)
albiscutata (Gmelin, 1790, *Ichneumon*)
crassipes (Rossi, 1790, *Ichneumon*) preocc.
incisa (Gmelin, 1790, *Ichneumon*)
superba (Christ, 1791, *Ichneumon*) preocc.
vulpes (Christ, 1791, *Ichneumon*)
varia (Olivier, 1792, *Ichneumon*) preocc.
flavicans (Fabricius, 1793, *Ichneumon*)
varia (Fabricius, 1793, *Ichneumon*) preocc.
variatoria (Fabricius, 1804, *Cryptus*)
colonator (Thunberg, 1824, *Ichneumon*)
femoralis Benoit, 1953

##### Distribution

England

### Poemeniinae

#### POEMENIINAE

Narayanan & Lal, 1953

##### Notes

Distribution data from Fitton et al. (1988), where treated as a tribe of Pimplinae, and Shaw (2006a). Further distribution references are given.

#### POEMENIINI

Narayanan & Lal, 1953

#### Deuteroxorides

Viereck, 1914

#### Deuteroxorides elevator

(Panzer, 1799)

Ichneumon elevator Panzer, 1799
albitarsus (Gravenhorst, 1829, *Xorides*)
nigricornis Clément, 1938
nigritarsus Clément, 1938

##### Distribution

England, Ireland

#### Podoschistus

Townes, 1957

#### Podoschistus scutellaris

(Desvignes, 1856)

Xorides scutellaris Desvignes, 1856
wahlbergi (Holmgren, 1860, *Xorides*)
erosus (Tschek, 1869, *Xorides*)

##### Distribution

England, Wales

#### Poemenia

Holmgren, 1859

OPHIODES Hartig, 1847
CALLICLISIS Förster, 1869
PHTHINODES Tschek, 1869
LISSONOTOPSIS Habermehl, 1917

#### Poemenia collaris

(Haupt, 1917)

Calliclisis collaris Haupt, 1917
picta (Haupt, 1938, *Calliclisis*)

##### Distribution

England

##### Notes

added by Fitton et al. (1988)

#### Poemenia hectica

(Gravenhorst, 1829)

Ephialtes hecticus Gravenhorst, 1829
montana (Hartig, 1847, *Ophiodes*)
tipularia Holmgren, 1860

##### Distribution

England, Scotland, Ireland

#### Poemenia notata

Holmgren, 1859

novakii Strobl, 1902
rufa (Habermehl, 1918, *Lissonotopsis*)
rufa (Habermehl, 1918, *Xorides*) preocc.
intermedia Constantineanu & Constantineanu, 1969
moldavica Constantineanu & Constantineanu, 1969

##### Distribution

England

##### Notes

added by Fitton et al. (1988)

#### PSEUDORHYSSINI

Wahl & Gauld, 1998

#### Pseudorhyssa

Merrill, 1915

#### Pseudorhyssa alpestris

(Holmgren, 1860)

Rhyssa alpestris Holmgren, 1860
ruficoxis (Kriechbaumer, 1887, *Rhyssa*)
hungarica (Mocsáry, 1905, *Rhyssa*)

##### Distribution

England, Wales

##### Notes

Welsh occurrence from Formstone (1999)

### Rhyssinae

#### RHYSSINAE

Morley, 1913

##### Notes

Distribution data from Fitton et al. (1988), where treated as a tribe of Pimplinae. Further distribution references are given.

#### Rhyssa

Gravenhorst, 1829

CRYPTOCENTRUM Kirby, 1837
PARARHYSSA Walsh, 1873

#### Rhyssa persuasoria

(Linnaeus, 1758)

Ichneumon persuasorius Linnaeus, 1758
marginalis Brullé, 1846
Rhyssa persuasoria *lineolata* Kriechbaumer, 1887 preocc.
Rhyssa persuasoria *gloriosa* Rudow, 1889

##### Notes

Some Scottish and Manx records from Hayes (1982) and Cowin and Williamson (1940).

#### Rhyssella

Rohwer, 1920

#### Rhyssella approximator

Fabricius, 1793

Ichneumon approximator Fabricius, 1793
Rhyssella approximator *curvipes* (Gravenhorst, 1829, *Rhyssa*)
Rhyssella approximator *rugicollis* (Zetterstedt, 1838, *Tryphon*)
bellator (Schiødte, 1839, *Rhyssa*) synonymy by Horstmann (2004c)
Rhyssella approximator *approximatrix* (Schulz, 1906, *Rhyssa*)
silbernageli (Kiss, 1926, *Rhyssa*)

### Stilbopinae

#### STILBOPINAE

Townes & Townes, 1949

#### Panteles

Förster, 1869

BRACHYPIMPLA misident.

##### Notes

*Panteles* has frequently been classified as a genus of Banchinae but we follow Wahl (1988) in classifying it in the Stilbopinae.

#### Panteles schuetzeanus

(Roman, 1925)

Brachypimpla schuetzeana Roman, 1925
schnetzeanus misspelling

##### Distribution

England, Scotland, Ireland

##### Notes

Quicke (2005) has published on the biology of *schuetzeanus* in Britain, unfortunately misspelling the name.

#### Stilbops

Förster, 1869

APHANOROPTRUM Förster, 1869
APHANOROPTRA Thomson, 1877
APHANORRHOPTRUM Dalla Torre, 1901
ERITRACHYNUS Schmiedeknecht, 1913

##### Notes

Distribution data from Fitton (1984), with additional data from Shaw (1989) and NMS.

#### Stilbops asper

(Schmiedeknecht, 1913)

Eritrachynus asper Schmiedeknecht, 1913

##### Distribution

England, Scotland

##### Notes

added by Fitton (1984)

#### Stilbops limneriaeformis

(Schmiedeknecht, 1888)

Pimpla limneriaeformis Schmiedeknecht, 1888

##### Distribution

Scotland, Ireland

#### Stilbops ruficornis

(Gravenhorst, 1829)

Lissonota ruficornis Gravenhorst, 1829
abdominalis (Gravenhorst, 1829, *Pimpla*)
nematorum (Rudow, 1881, *Pimpla*)
longiceps (Strobl, 1903, *Polyblastus*)

##### Distribution

England

#### Stilbops vetula

(Gravenhorst, 1829)

Pimpla vetula Gravenhorst, 1829
chrysostomus (Gravenhorst, 1829, *Phytodietus*)
pallipes (Gravenhorst, 1829, *Lissonota*)
pallidipes (Marshall, 1872, *Lissonota*)
varicauda (Capron, 1888, *Pimpla*)

##### Distribution

England, Scotland, Wales, Ireland

### Tersilochinae

#### TERSILOCHINAE

Schmiedeknecht, 1910

PHRUDINAE Townes & Townes, 1949

##### Notes

Following the phylogenetic results of Quicke et al. (2009), the Tersilochinae now encompasses the former subfamily Phrudinae (containing the genera *Astrenis*, *Phrudus* and *Pygmaeolus* in Britain) (they also synonymised Neorhacodinae under Tersilochinae but I don’t follow that decision). Distribution data for the ‘tersilochine’ genera from Horstmann (1971), Horstmann (1981a) and material in BMNH and NMS, mostly determined by K. Horstmann and, latterly, A. Khalaim; distribution data for the ‘phrudine’ genera from Gauld and Fitton (1980) and Vikberg and Koponen (2000); additional references are given.

#### Allophroides

Horstmann, 1971

#### Allophroides boops

(Gravenhorst, 1829)

Porizon boops Gravenhorst, 1829
italicus (Gravenhorst, 1829, *Porizon*)
breviventris (Hellén, 1958, *Allophrys*)

#### Aneuclis

Förster, 1869

#### Aneuclis melanaria

(Holmgren, 1860)

Thersilochus melanarius Holmgren, 1860
diversa (Szépligeti, 1899, *Isurgus*)
petiolaris (Szépligeti, 1899, *Isurgus*)

##### Distribution

England, Ireland

#### Astrenis

Förster, 1869

MENGERSENIA Schmiedeknecht, 1907
HAMBERGIELLA Roman, 1909

##### Notes

Until recently, *Astrenis* has usually been regarded as a synonym of *Phrudus*.

#### Astrenis brunneofacies

Vikberg, 2000

##### Distribution

England, Scotland

##### Notes

added by Vikberg and Koponen (2000)

#### Astrenis nigrifacies

Vikberg, 2000

##### Distribution

England, Scotland

##### Notes

added by Vikberg and Koponen (2000)

#### Astrenis paradoxus

(Schmiedeknecht, 1907)

Mengersenia paradoxa Schmiedeknecht, 1907

##### Distribution

England

##### Notes

added by Gauld and Fitton (1980)

#### Astrenis sinuatus

(Roman, 1909)

Hambergiella sinuata Roman, 1909

##### Distribution

England, Scotland, Ireland

#### Barycnemis

Förster, 1869

LEPTOPYGUS Förster, 1869
CRATOPHION Thomson, 1889
CYRTOPHION Thomson, 1889

#### Barycnemis agilis

(Holmgren, 1860)

Porizon agilis Holmgren, 1860

##### Distribution

England, Scotland

##### Notes

BMNH, NMS, det. Khalaim & Horstmann, added here

#### Barycnemis angustipennis

(Holmgren, 1860)

Porizon angustipennis Holmgren, 1860
added by

##### Distribution

England, Scotland, Ireland

##### Notes

added by Horstmann (1981a)

#### Barycnemis bellator

(Müller, 1776)

Ichneumon bellator Müller, 1776
laeviceps (Thomson, 1889, *Porizon*)
leviceps Dalla Torre, 1901
pfankuchi Lange, 1911

##### Distribution

England, Scotland

#### Barycnemis blediator

(Aubert, 1970)

Leptopygus blediator Aubert, 1970

##### Distribution

England, Wales

##### Notes

added by Wyatt and Foster (1989)

#### Barycnemis confusa

Horstmann, 1981

##### Distribution

Scotland

##### Notes

BMNH, NMS, det. Broad & Horstmann, added here

#### Barycnemis dissimilis

(Gravenhorst, 1829)

Porizon dissimilis Gravenhorst, 1829
erythrura (Strobl, 1904, *Porizon*)

##### Distribution

England

#### Barycnemis exhaustator

(Fabricius, 1798)

Ichneumon exhaustator Fabricius, 1798
obtusator (Panzer, 1809, *Ophion*)

##### Distribution

England

#### Barycnemis gravipes

(Gravenhorst, 1829)

Porizon gravipes Gravenhorst, 1829
hostilis (Gravenhorst, 1829, *Porizon*)

##### Distribution

England, Scotland, Ireland

#### Barycnemis guttulator

(Thunberg, 1824)

Ichneumon guttulator Thunberg, 1824
caudatula (Thomson, 1889, *Porizon*)

#### Barycnemis harpura

(Schrank, 1802)

Ichneumon harpurus Schrank, 1802
bedeguaris (Panzer, 1809, *Ophion*)

##### Distribution

England, Scotland, Wales, Ireland

#### Barycnemis punctifrons

Horstmann, 1981

##### Distribution

England, Ireland

##### Notes

added by Horstmann (1981a)

#### Diaparsis

Förster, 1869

#### Diaparsis

Förster, 1869

DIAPARSUS Thomson, 1889

#### Diaparsis (Diaparsis) carinifer

(Thomson, 1889)

Thersilochus carinifer Thomson, 1889
carinata (Bridgman, 1889, *Thersilochus*)
vernalis (Szépligeti, 1899, *Thersilochus*)

##### Distribution

England, Scotland, Wales, Ireland

#### Diaparsis (Diaparsis) multiplicator

Aubert, 1969

##### Distribution

England

#### Diaparsis (Diaparsis) nutritor

(Fabricius, 1804)

Ophion nutritor Fabricius, 1804
gemina (Holmgren, 1860, *Thersilochus*)
genalis (Thomson, 1889, *Thersilochus*)
rugosa (Szépligeti, 1905, *Temelucha*)
nutritrix Schulz, 1906

#### Diaparsis (Diaparsis) punctipleuris

Horstmann, 1981

##### Distribution

England

##### Notes

BMNH, det. Khalaim, added here

#### Ischnobatis

Förster, 1869

#### Diaparsis (Ischnobatis) stramineipes

(Brischke, 1880)

Thersilochus stramineipes Brischke, 1880
rufiventris (Brischke, 1880, *Thersilochus*)
flavicornis (Thomson, 1889, *Thersilochus*)
petiolata (Szépligeti, 1899, *Thersilochus*)

##### Distribution

England, Ireland

#### Nanodiaparsis

Horstmann, 1971

#### Diaparsis (Nanodiaparsis) aperta

(Thomson, 1889)

Thersilochus apertus Thomson, 1889

##### Distribution

England

##### Notes

NMS, BMNH, det. Horstmann and Khalaim, added here

#### Diaparsis (Nanodiaparsis) frontella

(Holmgren, 1860)

Thersilochus frontellus Holmgren, 1860

##### Distribution

England

#### Pseudaneuclis

Horstmann, 1971

#### Diaparsis (Pseudaneuclis) rara

(Horstmann, 1971)

Pseudaneuclis rarus Horstmann, 1971

##### Distribution

England

##### Notes

BMNH, det. Horstmann, added here

#### Epistathmus

Förster, 1869

#### Epistathmus crassicornis

Horstmann, 1971

##### Distribution

England, Scotland, Ireland

##### Notes

added by Horstmann (1981a)

#### Gelanes

Horstmann, 1981

#### Gelanes fusculus

(Holmgren, 1860)

Thersilochus fusculus Holmgren, 1860

##### Distribution

England, Scotland

##### Notes

NMS, det. Horstmann, BMNH, det. Khalaim, added here

#### Gelanes simillimus

Horstmann, 1981

##### Distribution

England, Scotland, Ireland

##### Notes

added by Horstmann (1981a)

#### Heterocola

Förster, 1869

#### Heterocoloides

Horstmann, 1971

#### Heterocola (Heterocoloides) linguaria

(Haliday, 1839)

Porizon linguarius Haliday, 1839
punctulata (Szépligeti, 1899, *Ischnobatis*)

##### Distribution

England, Ireland

##### Notes

Two specimens in BMNH, from Cornwall & Co. WX, have been identified as *H. rufiventris* Horstmann, 1971. There are no specimens of *linguaria*; their identity needs to be checked.

#### Phradis

Förster, 1869

EUTOMUS Förster, 1869
ISURGUS Förster, 1869

#### Phradis brevis

(Brischke, 1880)

Thersilochus brevis Brischke, 1880
temporalis (Thomson, 1889, *Thersilochus*)
styriacus (Strobl, 1904, *Thersilochus*)

##### Distribution

England, Ireland

##### Notes

added by Horstmann (1981a)

#### Phradis interstitialis

(Thomson, 1889)

Thersilochus interstitialis Thomson, 1889
brachygaster (Szépligeti, 1899, *Isurgus*)

##### Distribution

England, Scotland, Wales, Ireland

#### Phradis minutus

(Bridgman, 1889)

Thersilochus minutus Bridgman, 1889

##### Distribution

England, Wales, Ireland

#### Phradis monticola

Szépligeti, 1899

##### Distribution

England

##### Notes

BMNH, det. Ely, added here

#### Phradis morionellus

(Holmgren, 1860)

Thersilochus morionellus Holmgren, 1860
lanceolatus (Szépligeti, 1899, *Isurgus*)
oudesmani (Smits$)

##### Distribution

England, Ireland

#### Phradis nigritulus

(Gravenhorst, 1829)

Porizon nigritulus Gravenhorst, 1829
albipennis (Szépligeti, 1899, *Isurgus*)

##### Distribution

England

#### Phradis polonicus

Horstmann, 1981

##### Distribution

England, Scotland

##### Notes

BMNH, NMS, added here

#### Phradis rufiventris

Horstmann, 1981

##### Distribution

England

##### Notes

BMNH, det. Ely, added here

#### Phradis terebrator

Horstmann, 1981

##### Distribution

England

##### Notes

BMNH, det. Ely, added here

#### Phradis thyridialis

Horstmann, 1981

##### Distribution

England, Ireland

##### Notes

BMNH, det. Ely, added here

#### Phrudus

Förster, 1869

PHRUDUS Bridgman, 1886 preocc.
KTENOSTILPNUS Strobl, 1901
VENDOLUS Roman, 1914

#### Phrudus badensis

Hilpert, 1987

##### Distribution

England

##### Notes

added by Shaw (1991)

#### Phrudus defectus

Stelfox, 1966

##### Distribution

England, Scotland, Ireland, Isle of Man

#### Phrudus monilicornis

Bridgman, 1886

aequearticulatus (Strobl, 1901, *Ktenostilpnus*)
stilpninus (Roman, 1914, *Vendolus*)

##### Distribution

England, Scotland, Wales, Ireland

#### Probles

Förster, 1869

#### Euporizon

Horstmann, 1971

#### Probles (Euporizon) brevicauda

Horstmann, 1981

##### Distribution

England

##### Notes

added by Horstmann (1981a)

#### Probles (Euporizon) exilis

(Holmgren, 1860)

Thersilochus exilis Holmgren, 1860

##### Distribution

Scotland

##### Notes

NMS, det. Horstmann, added here

#### Probles (Euporizon) gilvipes

(Gravenhorst, 1829)

Porizon gilvipes Gravenhorst, 1829
pallipes (Holmgren, 1860, *Thersilochus*)
orchesiae (Morley, 1915, *Thersilochus*)

##### Distribution

England

#### Probles (Euporizon) longicaudator

Aubert, 1972

##### Distribution

England, Ireland

##### Notes

added by Horstmann (1981a)

#### Probles (Euporizon) marginatus

(Bridgman, 1886)

Thersilochus marginatus Bridgman, 1886

##### Distribution

England, Ireland

#### Probles (Euporizon) montanus

Horstmann, 1971

##### Distribution

Scotland, Ireland

##### Notes

added by Horstmann (1981a)

#### Probles (Euporizon) nigriventris

Horstmann, 1971

##### Distribution

Ireland

##### Notes

added by Horstmann (1981a)

#### Probles (Euporizon) rufipes

(Holmgren, 1860)

Thersilochus rufipes Holmgren, 1860
flavigaster (Szépligeti, 1899, *Ischnobatis*)

##### Distribution

England, Ireland

#### Probles (Euporizon) truncorum

(Holmgren, 1860)

Thersilochus truncorum Holmgren, 1860

##### Distribution

England, Ireland

#### Microdiaparsis

Horstmann, 1971

#### Probles (Microdiaparsis) caudiculatus

Khalaim, 2007

##### Distribution

England

##### Notes

added by Khalaim (2007)

#### Probles (Microdiaparsis) microcephalus

(Gravenhorst, 1829)

Porizon microcephalus Gravenhorst, 1829
quercetorum (Szépligeti, 1899, *Thersilochus*)
ruficoxis (Seyrig, 1927, *Diaparsis*)

##### Distribution

England, Scotland, Ireland

#### Probles (Microdiaparsis) neoversutus

(Horstmann, 1967)

Diaparsis neoversutus Horstmann, 1967
parviceps (Szépligeti, 1899, *Thersilochus*) preocc.

##### Distribution

England, Ireland

#### Probles (Microdiaparsis) versutus

(Holmgren, 1860)

Thersilochus versutus Holmgren, 1860
parviceps (Thomson, 1899, *Thersilochus*)

##### Distribution

England

#### Probles

Förster, 1869

#### Probles (Probles) erythrostomus

(Gravenhorst, 1829)

Porizon erythrostomus Gravenhorst, 1829
minator (Gravenhorst, 1829, *Porizon*)
melanarius Szépligeti, 1899

##### Distribution

England, Scotland, Ireland

#### Probles (Probles) flavipes

(Szépligeti, 1899)

Ischnobatis flavipes Szépligeti, 1899

##### Distribution

England, Ireland

##### Notes

added by Horstmann (1981a)

#### Rugodiaparsis

Horstmann, 1971

#### Probles (Rugodiaparsis) crassipes

(Thomson, 1889)

Thersilochus crassipes Thomson, 1889

##### Distribution

England, Scotland, Wales

##### Notes

BMNH, NMS, det. Horstmann, added here

#### Pygmaeolus

Hellén, 1958

#### Pygmaeolus nitidus

(Bridgman, 1889)

Thersilochus nitidus Bridgman, 1889

##### Distribution

England, Scotland, Ireland

#### Sathropterus

Förster, 1869

#### Sathropterus pumilus

(Holmgren, 1860)

Thersilochus pumilus Holmgren, 1860

##### Distribution

England, Ireland

##### Notes

added by Horstmann (1981a)

#### Spinolochus

Horstmann, 1971

#### Spinolochus laevifrons

(Holmgren, 1860)

Thersilochus laevifrons Holmgren, 1860
levifrons (Dalla Torre, 1901, *Cyrtophion*) preocc.

##### Distribution

England, Scotland, Ireland

##### Notes

added by Horstmann (1981a)

#### Tersilochus

Holmgren, 1859

#### Gonolochus

Förster, 1869

#### Tersilochus (Gonolochus) caudatus

(Holmgren, 1860)

Thersilochus caudatus Holmgren, 1860
pratensis (Szépligeti, 1899, *Thersilochus*)
salinus (Kiss, 1924, *Temelucha*)

##### Distribution

England, Ireland

#### Tersilochus (Gonolochus) rugulosus

Horstmann, 1981

##### Distribution

England

##### Notes

BMNH, det. Khalaim, added here

#### Pectinolochus

Aubert, 1960

POLEMOLOCHUS Aubert, 1964

#### Tersilochus (Pectinolochus) intermedius

Horstmann, 1981

##### Distribution

England

##### Notes

NMS, det. Horstmann, added here

#### Tersilochus (Pectinolochus) lapponicus

Hellén, 1958

##### Distribution

England, Wales, Ireland

##### Notes

added by Horstmann (1981a)

#### Tersilochus (Pectinolochus) spiracularis

Horstmann, 1971

##### Distribution

England

##### Notes

BMNH, det. Broad and Khalaim, added here

#### Tersilochus (Pectinolochus) striola

(Thomson, 1889)

Thersilochus striola Thomson, 1889
unguiculator (Aubert, 1960, *Thersilochus*)

##### Distribution

England, Scotland, Ireland

##### Notes

added by Horstmann (1981a)

#### Tersilochus (Pectinolochus) terebrator

(Horstmann, 1971)

Pectinolochus terebrator Horstmann, 1971

##### Distribution

England, Ireland

##### Notes

added by Horstmann (1981a)

#### Tersilochus

Holmgren, 1859

THERSILOCHUS misspelling

#### Tersilochus (Tersilochus) cognatus

(Holmgren, 1860)

Thersilochus cognatus Holmgren, 1860
jocator Holmgren, 1859 unavailable (Horstmann 2005a)

##### Distribution

England, Wales, Ireland

#### Tersilochus (Tersilochus) curvator

Horstmann, 1981

saltator misident.

##### Distribution

England, Ireland

##### Notes

added by Horstmann (1981a)

#### Tersilochus (Tersilochus) heterocerus

(Thomson, 1889)

Thersilochus heterocerus Thomson, 1889
stanionyteus Jonaitis, 1974
vicinus Jonaitis, 1974

##### Distribution

England, Ireland

#### Tersilochus (Tersilochus) liopleuris

(Thomson, 1889)

Thersilochus liopleuris Thomson, 1889

##### Distribution

England, Ireland

#### Tersilochus (Tersilochus) longicaudatus

Horstmann, 1971

##### Distribution

England, Ireland

##### Notes

added by Horstmann (1981a)

#### Tersilochus (Tersilochus) longicornis

(Thomson, 1889)

Thersilochus longicornis Thomson, 1889

##### Distribution

England, Scotland, Ireland

##### Notes

added by Horstmann (1981a)

#### Tersilochus (Tersilochus) microgaster

(Szépligeti, 1899)

Isurgus microgaster Szépligeti, 1899

##### Distribution

England

##### Notes

added by Barari et al. (2005)

#### Tersilochus (Tersilochus) nitidipleuris

Horstmann, 1971

##### Distribution

England, Ireland

##### Notes

added by Horstmann (1981a)

#### Tersilochus (Tersilochus) obliquus

(Thomson, 1889)

Thersilochus obliquus Thomson, 1889

##### Distribution

England, Ireland

##### Notes

added by Horstmann (1981a)

#### Tersilochus (Tersilochus) obscurator

(Aubert, 1959)

Thersilochus obscurator Aubert, 1959

##### Distribution

England, Scotland, Ireland

##### Notes

added by Horstmann (1981a)

#### Tersilochus (Tersilochus) ruberi

Horstmann, 1981

##### Distribution

Ireland

##### Notes

added by Horstmann (1981a)

#### Tersilochus (Tersilochus) triangularis

(Gravenhorst, 1807)

Ophion triangulare Gravenhorst, 1807
minutus (Szépligeti, 1899, *Isurgus*)

##### Distribution

England

#### Tersilochus (Tersilochus) tripartitus

(Brischke, 1880)

Thersilochus tripartitus Brischke, 1880
melanogaster (Thomson, 1889, *Thersilochus*)
nigricans (Szépligeti, 1899, *Thersilochus*)

##### Distribution

England, Ireland

##### Notes

added by Horstmann (1981a)

### Tryphoninae

#### ECLYTINI

Townes & Townes, 1945

##### Notes

Distribution data from Fitton and Ficken (1990) and Shaw and Kasparyan (2005).

#### Eclytus

Holmgren, 1857

#### Anoplectes

Kriechbaumer, 1896

#### Eclytus (Anoplectes) multicolor

(Kriechbaumer, 1896)

Anoplectes multicolor Kriechbaumer, 1896
praeclerus Schmiedeknecht, 1912

##### Distribution

England, Scotland, Ireland

##### Notes

added by Fitton and Ficken (1990)

#### Eclytus

Holmgren, 1857

ZAPEDIAS Förster, 1869 synonymy by Bennett (2015)

##### Notes

species of Eclytus (Eclytus) excluded from the British and Irish list:

[*ornatus* Holmgren, 1857] Fitton and Ficken (1990) recorded *Eclytus ornatus* from England and Scotland but their identifications were not based on egg characters, used by Kasparyan (1977) to separate species, and they suggested that their records of *ornatus* might be based on misidentifications. Shaw and Kasparyan (2005) did not find any specimens of the true *ornatus* in NMS. 

#### Eclytus (Eclytus) difficilis

Kasparyan, 1977

##### Distribution

Scotland

##### Notes

added by Shaw and Kasparyan (2005)

#### Eclytus (Eclytus) egregius

Kasparyan, 1977

##### Distribution

Scotland

##### Notes

added by Shaw and Kasparyan (2005)

#### Eclytus (Eclytus) exornatus

(Gravenhorst, 1829)

Mesoleptus exornatus Gravenhorst, 1829

##### Distribution

England, Scotland, Wales, Ireland

#### Eclytus (Eclytus) haustatorius

Kasparyan, 1977

##### Distribution

England, Scotland

##### Notes

added by Shaw and Kasparyan (2005)

#### IDIOGRAMMATINI

Cushman, 1942

##### Notes

Distribution data from Shaw and Kasparyan (2005).

#### Idiogramma

Förster, 1869

MACROCHASMUS Thomson, 1888

#### Idiogramma euryops

Förster, 1869

##### Distribution

Scotland

#### OEDEMOPSINI

Woldstedt, 1877

THYMARIDINI Schmiedeknecht, 1911

##### Notes

Distribution data from Fitton and Ficken (1990) and NMS, additional references given.

#### Cladeutes

Townes, 1969

#### Cladeutes discedens

(Woldstedt, 1874)

Perilissus discedens Woldstedt, 1874
haematothorax (Strobl, 1903, *Eclytus*)
lepidus Townes, 1969

##### Distribution

England, Ireland

##### Notes

added by Fitton and Ficken (1990)

#### Hercus

Townes, 1969

#### Hercus fontinalis

(Holmgren, 1857)

Eclytus fontinalis Holmgren, 1857
frontalis (Zetterstedt, 1838, *Bassus*) nom. ob.

##### Distribution

England, Scotland, Wales, Ireland

#### Neliopisthus

Thomson, 1883

#### Neliopisthus elegans

(Ruthe, 1855)

Phytodiaetus elegans Ruthe, 1855
ops (Morley, 1908, *Oedematopsis*)

##### Distribution

England

#### Oedemopsis

Tschek, 1869

CAMPOTHREPTUS Förster, 1869
HYBOPHANES Förster, 1869
OEDEMATOPSIS Morley, 1908
ODEMOPSIS misspelling

#### Oedemopsis scabricula

(Gravenhorst, 1829)

Tryphon scabriculus Gravenhorst, 1829
dorsata (Zetterstedt, 1838, *Bassus*)
pulchra (Zetterstedt, 1839, *Bassus*)
rogenhoferi Tschek, 1869
limbata Thomson, 1883

##### Distribution

England, Scotland, Ireland, Isle of Man

##### Notes

Irish distribution data from O'Connor (2003)

#### Thymaris

Förster, 1869

THYMARUS Thomson, 1883

#### Thymaris niger

(Taschenberg, 1865)

Hemiteles niger Taschenberg, 1865
fenestralis Morley, 1908
modestus Schmiedeknecht, 1912
simplicicornis Kiss, 1924 synonymy by Horstmann (1998b)
tristrigator Aubert, 1960

##### Distribution

England, Scotland

#### Thymaris srikem

Fitton & Ficken, 1990

##### Distribution

England, Wales, Ireland

##### Notes

added by Fitton and Ficken (1990)

#### Thymaris tener

(Gravenhorst, 1829)

Mesoleptus tener Gravenhorst, 1829
contaminatus (Gravenhorst, 1829, *Hemiteles*)
marchicus (Hartig, 1838, *Ischnoceros*)
pulchricornis Brischke, 1880
compressus (Thomson, 1883, *Thymarus*)

##### Distribution

England, Scotland

##### Notes

Fitton and Ficken (1990) synonymised *collaris* (Thomson, 1883) with *tener* but Kasparyan (1993) treated it as a valid species and gave characters for their separation; no British specimens of *collaris* have been seen, it is best separated from *tener* by the all red pronotum and partly red first metasomal tergite.

#### PHYTODIETINI

Hellén, 1915

NETELIINI Townes, 1938

#### Netelia

Gray, 1860

PANISCUS misident.

##### Notes

Distribution data from the nocturnal Ichneumonoidea recording scheme (run by G. Broad) and Shaw (2001), with additional references given.

#### Bessobates

Townes, Townes & Gupta, 1961

#### Netelia (Bessobates) cristata

(Thomson, 1888)

Parabatus cristatus Thomson, 1888
frankii (Brauns, 1889, *Parabatus*)

##### Distribution

England, Scotland, Wales, Ireland, Isle of Man

##### Notes

*Parabatus frankii* removed from synonymy by Horstmann (1998c) but resynonymised by Broad & Shaw (in prep.).

#### Netelia (Bessobates) latungula

(Thomson, 1888)

Parabatus latungulus Thomson, 1888

##### Distribution

England, Scotland, Ireland

#### Netelia (Bessobates) pallescens

(Schmiedeknecht, 1910)

Parabatus pallescens Schmiedeknecht, 1910

##### Distribution

England, Scotland, Ireland

##### Notes

added by Broad & Shaw (in prep.)

#### Netelia (Bessobates) virgata

(Geoffroy, 1785)

Ichneumon virgatus Geoffroy, 1785

##### Distribution

England, Scotland, Wales, Ireland, Isle of Man

#### Netelia (Bessobates) sp. R

##### Distribution

Scotland

##### Notes

added Broad & Shaw (in prep.)

#### Netelia

Gray, 1860

NETELIA Schulz, 1906

##### Notes

*Netelia fulvator* Delrio, 1971, was recorded as British by Horstmann (1992b), on the basis that the paralectotype specimens of *Paniscus testaceus* belong to this species; however, Broad & Shaw (in prep.) will synonymise *fulvator* under another name.

#### Netelia (Netelia) dilatata

(Thomson, 1888)

Paniscus dilatatus Thomson, 1888
brachycera (Thomson, 1888, *Paniscus*)
capito (Kokujev, 1889, *Paniscus*)
genalis (Kokujev, 1889, *Paniscus*)
schirjajewi (Kokujev, 1889, *Paniscus*)
sibiricola (Kokujev, 1889, *Paniscus*)
nigricans (Kriechbaumer, 1898, *Paniscus*)
nigridorsum (Meyer, 1929, *Paniscus*)

##### Distribution

England

#### Netelia (Netelia) fuscicarpus

(Kokujev, 1899)

Paniscus fuscicarpus Kokujev, 1899
desertus (Kokujev, 1915, *Paniscus*)
maltractus (Roman, 1938, *Paniscus*)
ambiguator Aubert, 1969

##### Distribution

England, Wales

##### Notes

added by Broad & Shaw (in prep.)

#### Netelia (Netelia) fuscicornis

(Holmgren, 1860)

Paniscus fuscicornis Holmgren, 1860
gracilipes (Thomson, 1888, *Paniscus*)
intersita (Kokujev, 1889, *Paniscus*)
montana (Kokujev, 1889, *Paniscus*)
praetermissa (Kokujev, 1889, *Paniscus*)

##### Distribution

England, Scotland

#### Netelia (Netelia) infractor

Delrio, 1971

##### Distribution

England, Scotland, Wales, Ireland

##### Notes

added by Broad & Shaw (in prep.)

#### Netelia (Netelia) melanura

(Thomson, 1888)

Paniscus melanurus Thomson, 1888

##### Distribution

England, Scotland

#### Netelia (Netelia) ocellaris

(Thomson, 1888)

Paniscus ocellaris Thomson, 1888

##### Distribution

England

#### Netelia (Netelia) opacula

(Thomson, 1888)

Paniscus opaculus Thomson, 1888

##### Distribution

England

#### Netelia (Netelia) testacea

(Gravenhorst, 1829)

Paniscus testaceus Gravenhorst, 1829

##### Distribution

England, Scotland

##### Notes

Added by Horstmann (1992b); this has been a much misunderstood name. Listed as a possible (senior) synonym of *opacula* in Fitton (1978), Horstmann (1992b) regarded it as a possible senior synonym of *valvator* Aubert, 1969. Broad & Shaw (in prep.), following a dissection of the lectotype male’s genitalia, offer a different interpretation of *testacea*. Tolkanitz (1974) and Kasparyan and Tolkanitz (2000) treated *fuscicarpus*, together with its associated synonyms, as a junior synonym of *testacea*. This is not followed here.

#### Netelia (Netelia) vinulae

(Scopoli, 1763)

Ichneumon vinulae Scopoli, 1763
inquinata (Gravenhorst, 1829, *Paniscus*) synonymy by Horstmann (1998b)
vinulae (Stephens, 1829, *Ophion*) preocc., synonymy by Horstmann (2000c)
cephalotes (Holmgren, 1860, *Paniscus*)

##### Distribution

England, Scotland

#### Netelia (Netelia) sp. W

##### Distribution

England, Wales

##### Notes

added by Broad & Shaw (in prep.)

#### Parabates

Förster, 1869

PARABATUS Thomson, 1888

#### Netelia (Parabates) nigricarpa

(Thomson, 1888)

Parabatus nigricarpus Thomson, 1888
semifusca (Strobl, 1904, *Parabatus*)

##### Distribution

England, Scotland

#### Paropheltes

Cameron, 1907

#### Netelia (Paropheltes) sp. C

##### Distribution

England

##### Notes

added by Broad & Shaw (in prep.)

#### Netelia (Paropheltes) inedita

(Kokujev, 1899)

Paniscus ineditus Kokujev, 1899
longipes misident.
ornata misident.
thomsonii misident.

##### Distribution

England, Scotland, Wales, Ireland

##### Notes

Added by Shaw (2001) who tentatively identified this widespread species as *thomsoni* (lapsus for *Netelia thomsonii* (Brauns, 1899, *Paniscus*), whilst noting that in Delrio (1975) specimens would key to *inedita*. Tolkanitz (1974) synonymised *inedita* under *thomsonii* but these two species are distinct (Broad & Shaw, in prep.), with *inedita* occurring in Britain and Ireland. This species has also been referred to in the literature as *ornata* and *longipes*.

#### Netelia (Paropheltes) millieratae

(Kriechbaumer, 1897)

Parabatus millieratae Kriechbaumer, 1897
added by

##### Distribution

England

##### Notes

added by Broad & Shaw (in prep.)

#### Netelia (Paropheltes) ornata

(Vollenhoven, 1873)

Paniscus ornatus Vollenhoven, 1873
longipes (Brauns, 1889, *Paniscus*)
catagrapha (Kokujev, 1915, *Paniscus*)
ignobilis (Kokujev, 1915, *Paniscus*)
versicolor (Kokujev, 1915, *Paniscus*)
decorator (Seyrig, 1927, *Paniscus*)

##### Distribution

England

##### Notes

Previous records of *ornata* in Britain were probably based on misidentifications of *inedita* but there are specimens of the true *ornata* in BMNH (Broad & Shaw, in prep.). *Netelia longipes* has been treated as a separate species by Tolkanitz (1974) and Kasparyan and Tolkanitz (2000) but as a synonym of *ornata* by Delrio (1975). The type of *longipes* is very similar to material corresponding to *ornata* (Broad & Shaw, in press).

#### Netelia (Paropheltes) tarsata

(Brischke, 1880)

Paniscus tarsatus Brischke, 1880

##### Distribution

England, Scotland, Wales, Ireland

#### Prosthodocis

Enderlein, 1912

#### Netelia (Prosthodocis) sp. A

##### Distribution

England, Ireland

##### Notes

added by Broad & Shaw (in prep.)

#### Netelia (Prosthodocis) sp. B

##### Distribution

Scotland, Ireland

##### Notes

added by Broad & Shaw (in prep.)

#### Phytodietus

Gravenhorst, 1829

##### Notes

Distribution data mainly from Kasparyan and Shaw (2008), also BMNH (det. A. Kostro-Ambroziak).

#### Neuchorus

Uchida, 1931

##### Notes

species of Phytodietus (Neuchhorus) excluded from the British and Irish list:

[*obscurus* (Ratzeburg, 1852, *Lissonota*); syn. *rufipes* Holmgren, 1860 (Horstmann 1998b); *orbitalis* Ulbricht, 1911 unavailable] Kasparyan and Shaw (2008) do not list any British or Irish specimens of *obscurus* and English specimens in BMNH, det. J.F. Perkins, are actually *elongator*.

#### Phytodietus (Neuchorus) elongator

Aubert, 1963

iassiensis Constantineanu, 1929 invalid

##### Distribution

England

##### Notes

added by Kasparyan and Shaw (2008)

#### Phytodietus (Neuchorus) maculator

Kasparyan & Shaw, 2008

##### Distribution

England

##### Notes

added by Kasparyan and Shaw (2008)

#### Phytodietus

Gravenhorst, 1829

PHYTODIAETUS Aggasiz, 1846
PHYTODIAETUS Morley, 1913

#### Phytodietus (Phytodietus) astutus

Gravenhorst, 1829

obscurus Desvignes, 1856 preocc.
continuus Thomson, 1887 synonymy by Horstmann (1998b)
britannicus (Habermehl, 1923, *Barytarbes*)

##### Distribution

England, Scotland, Wales

#### Phytodietus (Phytodietus) basalis

Kasparyan, 1993

##### Distribution

England, Scotland

##### Notes

added by Kasparyan and Shaw (2008)

#### Phytodietus (Phytodietus) femoralis

Holmgren, 1860

##### Distribution

Scotland

##### Notes

added by Kasparyan and Shaw (2008)

#### Phytodietus (Phytodietus) gelitorius

(Thunberg, 1824)

Ichneumon gelitorius Thunberg, 1824
polyzonius (Thunberg, 1824, *Ichneumon*) preocc.
coryphaeus Gravenhorst, 1829
carinatus Hellén, 1915
coxator (Aubert, 1963, *Lathrolestes*)

##### Distribution

England, Scotland, Wales, Ireland

#### Phytodietus (Phytodietus) geniculatus

Thomson, 1877

##### Distribution

England, Scotland, Wales, Ireland

#### Phytodietus (Phytodietus) griseanae

Kerrich, 1962

##### Distribution

England, Scotland, Wales

#### Phytodietus (Phytodietus) montanus

Tolkanitz, 1979

##### Distribution

England, Scotland, Wales, Ireland

##### Notes

added by Kasparyan and Shaw (2008)

#### Phytodietus (Phytodietus) ornatus

Desvignes, 1856

rubricosus Thomson, 1877
rufipictus Brischke, 1880
pictus (Habermehl, 1923, *Barytarbes*) synonymy by Horstmann (2004b)

##### Distribution

England, Scotland, Wales, Ireland

#### Phytodietus (Phytodietus) polyzonias

(Forster, 1771)

Ichneumon polyzonias Forster, 1771
segmentator Gravenhorst, 1829

##### Distribution

England

##### Notes

some distribution data from Fitton (1976)

#### Phytodietus (Phytodietus) variegatus

(Fonscolombe, 1854)

Lissonota variegata Fonscolombe, 1854
albipes Holmgren, 1856

##### Distribution

England, Scotland

##### Notes

added by Kasparyan and Shaw (2008)

#### SPHINCTINI

Förster, 1869

#### Sphinctus

Gravenhorst, 1829

#### Sphinctus serotinus

Gravenhorst, 1829

##### Distribution

England

##### Notes

Probably extinct in Britain (Shaw and Voogd 2016).

#### TRYPHONINI

Shuckard, 1840

EXENTERINI Förster, 1869
CTENISCINI Thomson, 1883
GRYPOCENTRINI Townes & Townes, 1949

##### Notes

Except where noted otherwise, distribution data from Kerrich (1952), Kasparyan (1973), Fitton (1976), Shaw and Kasparyan (2005) and the collections of the BMNH. Additional references are given. The Exenterini has usually been considered a separate tribe, the distinctive group of genera lacking hind tibial spurs (in Britain comprising *Acrotomus*, *Cteniscus*, *Cycasis*, *Eridolius*, *Excavarus*, *Exenterus*, *Exyston*, *Kristotomus*, *Orthomiscus* and *Smicroplectus*) were recognised as being a derived group of Tryphonini by Bennett (2015).

#### Acrotomus

Holmgren, 1857

DELOTOMUS Förster, 1869

#### Acrotomus lucidulus

(Gravenhorst, 1829)

Tryphon lucidulus Gravenhorst, 1829
sexcinctus (Gravenhorst, 1829, *Tryphon*)
auriculatus (Thomson, 1883, *Delotomus*)

##### Distribution

England, Scotland, Wales

#### Acrotomus succinctus

(Gravenhorst, 1829)

Tryphon succinctus Gravenhorst, 1829

##### Distribution

England, Scotland, Wales, Ireland

#### Cosmoconus

Förster, 1869

#### Cosmoconus ceratophorus

(Thomson, 1888)

Tryphon ceratophorus Thomson, 1888

##### Distribution

England, Scotland

#### Cosmoconus elongator

(Fabricius, 1775)

Ichneumon elongator Fabricius, 1775
elliotti (Morley, 1911, *Tryphon*)

##### Distribution

England, Scotland, Ireland, Isle of Man

#### Cosmoconus meridionator

Aubert, 1963

##### Distribution

England, Scotland

##### Notes

added by Shaw and Kasparyan (2005)

#### Cosmoconus nigriventris

Kasparyan, 1971

##### Distribution

England, Scotland

##### Notes

added by Shaw and Kasparyan (2005)

#### Cteniscus

Haliday, 1832

EUDIABORUS Kerrich, 1952

#### Cteniscus maculiventris

(Ashmead, 1896)

Diaborus maculiventris Ashmead, 1896

##### Distribution

Scotland

##### Notes

Added by Kerrich (1952); British specimens belong to the subspecies *boreoalpinus* (Kerrich, 1952, *Eudiaborus*). Overlooked by Fitton (1978).

#### Cteniscus nigrifrons

(Thomson, 1883)

Diaborus nigrifrons Thomson, 1883

##### Distribution

England

#### Cteniscus pedatorius

(Panzer, 1809)

Bassus pedatorius Panzer, 1809
sexlituratus (Gravenhorst, 1829, *Tryphon*)
filipalpis (Thomson, 1883, *Diaborus*)
moravicus (Gregor, 1937, *Diaborus*)

##### Distribution

England, Scotland, Ireland

#### Cteniscus scalaris

(Gravenhorst, 1829)

Tryphon scalaris Gravenhorst, 1829
pallitarsis (Thomson, 1883, *Diaborus*)
palliditarsis (Dalla Torre, 1901, *Diaborus*)

##### Distribution

England, Scotland

##### Notes

some distribution data from Rahoo and Luff (1988)

#### Ctenochira

Förster, 1855

CTENACME Förster, 1869
GEMOPHAGA Förster, 1869
SCOPIORUS Förster, 1869
CTENACMUS Thomson, 1883
CTENACMA Schulz, 1906
EXOCHOBLASTUS Schmiedeknecht, 1912
SCOPIMENUS Roman, 1937
COELOPROSOPON Bauer, 1958

##### Notes

species of *Ctenochira* excluded from the British and Irish list:

[?*breviseta* (Ratzeburg, 1852, *Pimpla*); syn. *aberrans* (Ruthe, 1855, *Tryphon*) (Horstmann 2002a)] Although appearing on the last British list (Fitton 1978, as *aberrans*), this may have been on the basis of misidentifications of *haemosterna* (K. Horstmann, pers. comm.). Kasparyan and Tolkanitz (2000) interpreted *aberrans* as a synonym of *haemosterna* but K. Horstmann’s interpretation is followed here, having revised the closely related species involved (Horstmann 2002a).

#### Ctenochira angulata

(Thomson, 1883)

Polyblastus angulatus Thomson, 1883

##### Distribution

Scotland, Isle of Man

#### Ctenochira angustata

(Roman, 1909)

Polyblastus angustatus Roman, 1909

#### Ctenochira arcuata

(Holmgren, 1857)

Polyblastus arcuatus Holmgren, 1857
antennator Aubert, 1965

##### Distribution

England

##### Notes

W. Ely has noted that many specimens under this name (including British?) are *marginata* sensu Kasparyan.

#### Ctenochira gelida

Kasparyan, 1973

##### Distribution

Scotland

##### Notes

added by Shaw and Kasparyan (2005), who suggest that the British specimens, which differ slightly from Continental forms, may represent a separate species.

#### Ctenochira genalis

(Thomson, 1883)

Polyblastus genalis Thomson, 1883

##### Distribution

England

##### Notes

Added by Kasparyan (1973); Kasparyan’s inclusion of British specimens (coll. J.P. Brock) was overlooked by Fitton (1978) and by Shaw and Kasparyan (2005).

#### Ctenochira gilvipes

(Holmgren, 1857)

albiventris (Brischke, 1892, *Polyblastus*)

##### Distribution

England, Scotland, Isle of Man

#### Ctenochira grossa

(Brischke, 1871)

Polyblastus grossus Brischke, 1871
annulicornis (Giraud, 1872, *Polyblastus*)

##### Distribution

England, Scotland

##### Notes

Listed in Fitton (1978) (as a doubtfully placed species of *Polyblastus*) as *annulicornis*, it was subsequently recorded as *Ctenochira grossa* by Kasparyan and Tolkanitz (2000).

#### Ctenochira haemosterna

(Haliday, 1839)

Tryphon haemosternus Haliday, 1839
senilis (Holmgren, 1857, *Polyblastus*)
nigripalpis (Thomson, 1883, *Polyblastus*)
subrufa (Bridgman, 1887, *Polysphincta*) synonymy by Kasparyan and Tolkanitz (2000)
haematosterna (Dalla Torre, 1901, *Polyblastus*)

##### Distribution

England, Scotland, Ireland

##### Notes

Some distribution data from Horstmann (2002a); Shaw and Kasparyan (2005) listed *aberrans* as a junior synonym of *haemosterna* as they were unaware of Horstmann’s (Horstmann 2002a) revised synonymy (see note under *breviseta*).

#### Ctenochira marginata

(Holmgren, 1857)

Polyblastus marginatus Holmgren, 1857
fractigena (Heinrich, 1953, *Scopiorus*)

##### Distribution

England, Scotland, Wales, Isle of Man

#### Ctenochira meridionator

Aubert, 1969

##### Distribution

England

##### Notes

Added by Shaw and Kasparyan (2005); listed as a subspecies of *genalis* in Yu and Horstmann (1997).

#### Ctenochira pastoralis

(Gravenhorst, 1829)

Tryphon pastoralis Gravenhorst, 1829
mutabilis (Holmgren, 1857, *Polyblastus*)
nitidiventris (Holmgren, 1857, *Polyblastus*)

#### Ctenochira pratensis

(Gravenhorst, 1829)

Tryphon pratensis Gravenhorst, 1829

##### Distribution

England

#### Ctenochira propinqua

(Gravenhorst, 1829)

Tryphon propinquus Gravenhorst, 1829
obscura (Stephens, 1835, *Tryphon*) synonymy by Kasparyan and Tolkanitz (2000)
caudata (Holmgren, 1856, *Poyblastus*)

##### Distribution

England, Scotland, Ireland

#### Ctenochira romani

(Pfankuch, 1925)

Poyblastus romani Pfankuch, 1925
pygobarba (Roman, 1937, *Scopimenus*)

##### Distribution

England, Isle of Man

#### Ctenochira rubranator

Aubert, 1965

##### Distribution

Scotland

##### Notes

Added by Shaw and Kasparyan (2005); listed as a subspecies of *genalis* in Yu and Horstmann (1997).

#### Ctenochira rufipes

(Gravenhorst, 1829)

Tryphon rufipes Gravenhorst, 1829
anilis (Holmgren, 1857, *Polyblastus*)
glabella (Holmgren, 1857, *Polyblastus*)
limosa (Holmgren, 1857, *Polyblastus*)
mixta (Holmgren, 1857, *Polyblastus*)
nigella (Holmgren, 1857, *Polyblastus*)
praedator (Holmgren, 1857, *Polyblastus*)
holmgreni (Woldstedt, 1874, *Polyblastus*) preocc.
woldstedtii (Dalla Torre, 1901, *Polyblastus*)

##### Distribution

England, Scotland

#### Ctenochira sanguinatoria

(Ratzeburg, 1852)

Tryphon sanguinatorius Ratzeburg, 1852

##### Distribution

England

#### Ctenochira sphaerocephala

(Gravenhorst, 1829)

Tryphon sphaerocephalus Gravenhorst, 1829
bisculpta (Gravenhorst, 1829, *Tryphon*)
trisculpta (Stephens, 1835, *Tryphon*)
bifasciata (Zetterstedt, 1838, *Tryphon*)
trisculpta (Holmgren, 1856, *Polyblastus*)

##### Distribution

England, Scotland, Isle of Man

#### Ctenochira validicornis

(Brischke, 1871)

Polyblastus validicornis Brischke, 1871
added by
fusicornis (Thomson, 1883, *Polyblastus*)
insculpta (Habermehl, 1922, *Polyblastus*)

##### Distribution

England, Scotland, Ireland

##### Notes

Added by Shaw and Kasparyan (2005); listed as a subspecies of *genalis* in Yu and Horstmann (1997).

#### Ctenochira xanthopyga

(Holmgren, 1857)

Polyblastus xanthopygus Holmgren, 1857
rivalis (Holmgren, 1857, *Polyblastus*)

##### Distribution

England, Scotland

#### Cycasis

Townes, 1965

#### Cycasis rubiginosa

(Gravenhorst, 1829)

Tryphon rubiginosus Gravenhorst, 1829
insidiator (Holmgren, 1857, *Acrotomus*)
morio (Holmgren, 1857, *Exenterus*)
binotata (Thomson, 1883, *Delotomus*)
parvula (Thomson, 1883, *Delotomus*)

##### Distribution

England, Scotland

#### Dyspetes

Förster, 1869

DYSPETUS Thomson, 1883

#### Dyspetes arrogator

Heinrich, 1949

rufatus Gregor, 1929 unavailable (Horstmann 2005a)

##### Distribution

England, Ireland

#### Dyspetes luteomarginatus

Habermehl, 1925

chrysogaster (Gmelin, 1790, *Ichneumon*) preocc., synonymy by Horstmann (2006a)
praerogator (Thomson, 1883, *Dyspetus*) unavailable (Horstmann 2005a)
fracticeps (Townes & Townes, 1950, *Dyspetus*) synonymy by Horstmann (2006a)

##### Distribution

England, Scotland, Wales, Ireland, Isle of Man

##### Notes

NMS, det. Horstmann, BMNH, det. Broad, added here; specimens of *Dyspetes* in Britain and Ireland have usually been treated as one species (named *praerogator* or *arrogator*) but Horstmann (2006a) demonstrated that the commoner species is actually *luteomarginatus*. The name *praerogator* was used by Gravenhorst and Thomson, referring to Linnaeus’s *praerogator*, which is actually a species of *Tranosemella* (Campopleginae). These usages of *praerogator* are unavailable names (Horstmann 2005a, Horstmann 2006a).

#### Eridolius

Förster, 1869

ANISOCTENION Förster, 1869

#### Eridolius alacer

(Gravenhorst, 1829)

Tryphon alacer Gravenhorst, 1829
xanthopus (Holmgren, 1857, *Acrotomus*)

##### Distribution

England, Scotland

#### Eridolius aurifluus

(Haliday, 1839)

Tryphon aurifluus Haliday, 1839
geniculosus (Schiødte, 1839, *Exenterus*)
approximatus (Holmgren, 1857, *Exenterus*)

##### Distribution

England, Ireland

#### Eridolius basalis

(Stephens, 1835)

Tryphon basalis Stephens, 1835
connatus (Holmgren, 1857, *Exenterus*)
flavilabris (Holmgren, 1857, *Exenterus*)
gracilis (Holmgren, 1857, *Exenterus*)
hostilis (Holmgren, 1857, *Exenterus*)
limbatellus (Holmgren, 1857, *Exenterus*)
umbellatarum (Woldstedt, 1874, *Exenterus*)
rufofasciatus (Strobl, 1903, *Polyblastus*) synonymy by Horstmann (2012a)
minutulus (Pfankuch, 1907, *Cteniscus*)

##### Distribution

England, Scotland, Wales, Ireland, Isle of Man

#### Eridolius bimaculatus

(Holmgren, 1856)

Exenterus bimaculatus Holmgren, 1856
zonellus (Holmgren, 1857, *Exenterus*)
alpinus (Roman, 1909, *Cteniscus*)

##### Distribution

England, Scotland, Wales, Ireland

#### Eridolius consobrinus

(Holmgren, 1857)

Exenterus consobrinus Holmgren, 1857

##### Distribution

Ireland

#### Eridolius curtisii

(Haliday, 1839)

Tryphon curtisii Haliday, 1839

##### Distribution

England, Scotland, Ireland

#### Eridolius dorsator

(Thunberg, 1824)

Ichneumon dorsator Thunberg, 1824
mitigosus (Gravenhorst, 1829, *Tryphon*)
lineola (Stephens, 1835, *Tryphon*)
similatorius (Schiødte, 1839, *Exenterus*)
limbatus (Holmgren, 1856, *Exenterus*)
alpicola (Holmgren, 1857, *Exenterus*)
borealis (Holmgren, 1857, *Exenterus*)
frigidus (Holmgren, 1857, *Exenterus*)
brevigena (Thomson, 1883, *Cteniscus*)
punctipes (Thomson, 1883, *Cteniscus*)
punctipleuris (Thomson, 1883, *Cteniscus*)
signifer (Thomson, 1883, *Cteniscus*)
albicollis (Habermehl, 1925, *Cteniscus*)

##### Distribution

England, Ireland

#### Eridolius elegans

(Stephens, 1835)

Tryphon elegans Stephens, 1835
aulicus (Roman, 1914, *Cteniscus*)

##### Distribution

England, Scotland

#### Eridolius ermolenkoi

Kasparyan, 1990

##### Distribution

England

##### Notes

added by Shaw and Kasparyan (2005)

#### Eridolius flavomaculatus

(Gravenhorst, 1829)

Tryphon flavomaculatus Gravenhorst, 1829
praeustus (Holmgren, 1857, *Exenterus*)
pumilus (Holmgren, 1857, *Exenterus*)
ustulatus (Holmgren, 1857, *Exenterus*)
quadrinotatus (Thomson, 1883, *Cteniscus*)
t-nigrum (Thomson, 1883, *Cteniscus*)
facialis (Roman, 1913, *Cteniscus*)

##### Distribution

England, Scotland

#### Eridolius gnathoxanthus

(Gravenhorst, 1829)

Tryphon gnathoxanthus Gravenhorst, 1829
hachfeldi (Ulbricht, 1926, *Polyblastus*)

##### Distribution

England, Scotland, Ireland

#### Eridolius hofferi

(Gregor, 1937)

Cteniscus hofferi Gregor, 1937

##### Distribution

England, Ireland

#### Eridolius lineiger

(Thomson, 1883)

Cteniscus lineiger Thomson, 1883
nordstromi (Kerrich, 1952, *Cteniscus*)

##### Distribution

Scotland

##### Notes

added by Shaw and Kasparyan (2005)

#### Eridolius pachysoma

(Stephens, 1835)

Tryphon pachysoma Stephens, 1835
colorator (Zetterstedt, 1838, *Tryphon*)

##### Distribution

England, Ireland

#### Eridolius pictus

(Gravenhorst, 1829)

Tryphon pictus Gravenhorst, 1829
marginatus (Thomson, 1833, *Cteniscus*)
crassiceps (Szépligeti, 1901, *Diaborus*)

##### Distribution

England, Scotland, Wales, Ireland, Isle of Man

#### Eridolius romani

(Kerrich, 1952)

Cteniscus romani Kerrich, 1952

##### Distribution

Ireland

#### Eridolius rufilabris

(Holmgren, 1857)

Exenterus rufilabris Holmgren, 1857
genalis (Thomson, 1883, *Cteniscus*)

##### Distribution

England, Ireland

##### Notes

some distribution data from Kerrich (1962)

#### Eridolius rufonotatus

(Holmgren, 1857)

Exenterus rufonotatus Holmgren, 1857
breviventris (Thomson, 1883, *Cteniscus*)
fulvipes (Kriechbaumer, 1896, *Exenterus*)

##### Distribution

Scotland, Ireland

#### Eridolius similis

(Holmgren, 1857)

Exenterus similis Holmgren, 1857

##### Distribution

England

##### Notes

added by Ely (2010)

#### Eridolius taigensis

Kasparyan, 1985

##### Distribution

Scotland

##### Notes

added by Shaw and Kasparyan (2005)

#### Erromenus

Holmgren, 1857

ANIAROPHRON Förster, 1869
TRICHOCALYMMA Förster, 1869
TRICHOCALYMMUS Thomson, 1887

#### Erromenus analis

Brischke, 1871

##### Distribution

England, Scotland

#### Erromenus bibulus

Kasparyan, 1973

##### Distribution

England, Scotland, Wales

#### Erromenus brunnicans

(Gravenhorst, 1829)

Tryphon brunnicans Gravenhorst, 1829
brunicans Dalla Torre, 1901 preocc.

##### Distribution

England, Scotland

#### Erromenus calcator

(Müller, 1776)

Ichneumon calcator Müller, 1776
erythropus (Gmelin, 1790, *Ichneumon*)
carinatus (Holmgren, 1857, *Polyblastus*)
oelandicus (Holmgren, 1857, *Polyblastus*)
scutellaris (Holmgren, 1857, *Polyblastus*)

##### Distribution

England, Scotland

#### Erromenus junior

(Thunberg, 1824)

Ichneumon junior Thunberg, 1824
frenator (Gravenhorst, 1829, *Exochus*)
arenicola Thomson, 1883

##### Distribution

England, Scotland

#### Erromenus plebejus

(Woldstedt, 1878)

Trichocalymma plebejus Woldstedt, 1878
bipunctatus (Woldstedt, 1878, *Trichocalymma*)
brevitarsis Thomson, 1883

##### Distribution

England

#### Erromenus punctatus

(Woldstedt, 1878)

Trichocalymma punctatus Woldstedt, 1878
added by
simplex Thomson, 1883
defectivus Strobl, 1903

##### Distribution

Scotland, Wales

##### Notes

added by Shaw and Kasparyan (2005)

#### Erromenus punctulatus

Holmgren, 1857

niger (Szépligeti, 1901, *Aniarophron*)

##### Distribution

England, Scotland, Ireland

#### Erromenus zonarius

(Gravenhorst, 1820)

Ichneumon zonarius Gravenhorst, 1820
obscuratus Habermehl, 1925

##### Distribution

England, Scotland, Wales

#### Excavarus

Davis, 1897

#### Excavarus apiarius

(Gravenhorst, 1829)

Tryphon apiarius Gravenhorst, 1829
obscuratorius (Panzer, 1809, *Ichneumon*) preocc.

##### Distribution

England, Wales

#### Exenterus

Hartig, 1837

ACTENONYX Förster, 1869
PICROSCOPUS Förster, 1869

##### Notes

species of *Exenterus* excluded from the British and Irish list:

[*confusus* Kerrich, 1952]

[*tricolor* Roman, 1913] 

[*vellicatus* Cushman, 1940] 

Released into Wales from Austrian stock, no evidence of successful establishment (Billany et al. 1983).

Doubtfully placed species of *Exenterus*:

[*anceps* (Stephens, 1835, *Tryphon*) nom. dub., from England] Listed as a doubtfully placed species of *Tryphon* by Fitton (1978), placed here in *Exenterus* following Yu and Horstmann (1997).

#### Exenterus abruptorius

(Thunberg, 1824)

Ichneumon abruptorius Thunberg, 1824
cingulatorius Holmgren, 1857

##### Distribution

England, Scotland

#### Exenterus adspersus

Hartig, 1838

lepidus Holmgren, 1857
laricinus Thomson, 1888

##### Distribution

England

#### Exenterus amictorius

(Panzer, 1801)

Ichneumon amictorius Panzer, 1801
marginatorius (Fabricius, 1793, *Ichneumon*) preocc.
sulcatorius (Thunberg, 1824, *Ichneumon*)
claripennis Thomson, 1883

##### Distribution

England

#### Exenterus ictericus

(Gravenhorst, 1829)

Tryphon ictericus Gravenhorst, 1829

##### Distribution

Scotland

##### Notes

added by Shaw and Kasparyan (2005)

#### Exenterus oriolus

Hartig, 1838

flavellus Thomson, 1883
brunnescens Fahringer, 1941

##### Distribution

England

#### Exyston

Schiødte, 1839

ANECPHYSIS Förster, 1869
DIABORUS Förster, 1869
TRICAMPTUS Förster, 1869
PAREXYSTON Kerrich, 1952

#### Exyston calcaratus

Thomson, 1883

##### Distribution

England, Ireland

#### Exyston pratorum

(Woldstedt, 1874)

Exenterus pratorum Woldstedt, 1874
sedulus (Woldstedt, 1878, *Cteniscus*)
brevipetiolatus Thomson, 1883
melanurus Ulbricht, 1926 unavailable

##### Distribution

England, Scotland, Ireland

#### Exyston sponsorius

(Fabricius, 1781)

Ichneumon sponsorius Fabricius, 1781
cinctulus (Gravenhorst, 1820, *Ichneumon*)
conopsator (Thunberg, 1824, *Ichneumon*)
carinatus Thomson, 1883

##### Distribution

England, Scotland, Wales, Ireland

#### Exyston subnitidus

(Gravenhorst, 1829)

Tryphon subnitidus Gravenhorst, 1829
phaeorrhaeus (Haliday, 1839, *Tryphon*)

##### Distribution

England, Ireland

#### Grypocentrus

Ruthe, 1855

APIMELES Förster, 1869

#### Grypocentrus albipes

Ruthe, 1855

##### Distribution

England, Scotland

#### Grypocentrus apicalis

Thomson, 1883

#### Grypocentrus basalis

Ruthe, 1855

##### Distribution

England, Scotland

#### Grypocentrus bilobus

Kasparyan, 1976

##### Distribution

Scotland

##### Notes

added by Shaw and Kasparyan (2005)

#### Grypocentrus cinctellus

Ruthe, 1855

##### Distribution

England

#### Grypocentrus incisulus

Ruthe, 1855

erythrurus Ulbricht, 1926

#### Kristotomus

Mason, 1962

#### Kristotomus laetus

(Gravenhorst, 1829)

Mesoleptus laetus Gravenhorst, 1829
cephalotes (Gravenhorst, 1829, *Tryphon*)
orbitatorius (Schiødte, 1839, *Exenterus*)
calcaratus (Thomson, 1883, *Delotomus*)
marginatus (Thomson, 1883, *Delotomus*)
dioszeghyi (Kiss, 1924, *Cteniscus*) synonymy by Horstmann (2009d)

##### Distribution

England, Ireland

#### Kristotomus laticeps

(Gravenhorst, 1829)

Tryphon laticeps Gravenhorst, 1829

##### Distribution

England

#### Kristotomus pumilio

(Holmgren, 1857)

Exenterus pumilio Holmgren, 1857

##### Distribution

England

#### Kristotomus ridibundus

(Gravenhorst, 1829)

Tryphon ridibundus Gravenhorst, 1829

##### Distribution

England

#### Kristotomus triangulatorius

(Gravenhorst, 1829)

Tryphon triangulatorius Gravenhorst, 1829
mesoleptoides (Stephens, 1835, *Tryphon*)
coarctatus (Holmgren, 1857, *Acrotomus*)

##### Distribution

England, Scotland, Ireland

#### Monoblastus

Hartig, 1837

COELOCONUS Förster, 1869
XIPHURUS Kriechbaumer, 1896 preocc., synonymy by Horstmann (2002a)
IDOTHRICHUS Schmiedeknecht, 1907
PSEUDOPSILOSAGE Gregor, 1929

#### Monoblastus brachyacanthus

(Gmelin, 1790)

Ichneumon brachyacanthus Gmelin, 1790
testaceus (Gmelin, 1790, *Ichneumon*)
oraniensis (Schmiedeknecht, 1912, *Psilosage*)

##### Distribution

England, Scotland, Ireland

#### Monoblastus caudatus

(Hartig, 1837)

Tryphon caudatus Hartig, 1837
lateralis (Giraud, 1872, *Tryphon*) preocc.
sericeus (Brischke, 1892, *Phaestus*)
lateralis Kriechbaumer, 1896 preocc.

##### Distribution

England

##### Notes

BMNH, det. Broad, added here

#### Monoblastus marginellus

(Gravenhorst, 1829)

Lissonota marginella Gravenhorst, 1829
compunctor misident.

##### Distribution

England

#### Neleges

Förster, 1869

ANELPISTUS Brauns, 1898 preocc.
BRAUNSIANUS Berg, 1898

#### Neleges proditor

(Gravenhorst, 1829)

Tryphon proditor Gravenhorst, 1829
bidentatus (Brauns, 1898, *Anelpistus*)
bidentatus (Strobl, 1903, *Tryphon*) preocc.
bimucronatus (Strobl, 1903, *Erromenus*)

##### Distribution

England

#### Orthomiscus

Mason, 1955

#### Orthomiscus unicinctus

(Holmgren, 1857)

Exenterus unicinctus Holmgren, 1857
macrocephalus (Holmgren, 1857, *Exenterus*)

##### Distribution

England

#### Otoblastus

Förster, 1869

#### Otoblastus luteomarginatus

(Gravenhorst, 1829)

Tryphon luteomarginatus Gravenhorst, 1829

##### Distribution

England

#### Polyblastus

Hartig, 1837

##### Notes

doubtfully placed species of *Polyblastus*: 

[*bridgmani* Parfitt, 1882 nom. dub., from England] Fitton (1976)

[*parvulus* (Gravenhorst, 1829, *Tryphon*) nom. dub.]

#### Cophencus

Townes & Townes, 1949

#### Polyblastus (Cophencus) macrocentrus

Thomson, 1888

##### Distribution

England, Scotland, Wales

#### Labroctonus

Förster, 1869

NEMIOBLASTUS Thomson, 1883

#### Polyblastus (Labroctonus) alternans

Schiødte, 1838

albicoxa Thomson, 1883

##### Notes

British specimens without locality data in BMNH.

#### Polyblastus (Labroctonus) melanostigmus

Holmgren, 1857

grammicus Holmgren, 1857
lucidus Brischke, 1892

##### Distribution

England, Scotland, Isle of Man

#### Polyblastus (Labroctonus) nanus

Kasparyan, 1973

##### Distribution

England

##### Notes

added by Shaw and Kasparyan (2005)

#### Polyblastus (Labroctonus) pallicoxa

Thomson, 1888

pallidicoxa Dalla Torre, 1901

##### Distribution

England, Ireland, Isle of Man

#### Polyblastus (Labroctonus) stenocentrus

Holmgren, 1857

albicoxa Thomson, 1883

##### Distribution

England, Scotland, Isle of Man

##### Notes

Shaw and Kasparyan (2005) treat this as species separate from *alternans* Schiødte, 1838, of which it is listed as a subspecies by Yu and Horstmann (1997).

#### Polyblastus (Labroctonus) westringi

Holmgren, 1857

##### Distribution

England, Scotland

#### Polyblastus

Hartig, 1837

#### Polyblastus (Polyblastus) cancer

(Hartig, 1837)

Tryphon cancer Hartig, 1837
palaemon Schiødte, 1838
holosericeus (Ratzeburg, 1848, *Tryphon*)
pyramidatus Holmgren, 1857

##### Distribution

England, Scotland, Isle of Man

#### Polyblastus (Polyblastus) cothurnatus

(Gravenhorst, 1829)

Tryphon cothurnatus Gravenhorst, 1829
drewseni Schiødte, 1838
unicinctus Bridgman, 1889
intermedius Ulbricht, 1916 unavailable
hungaricus (Kiss, 1926, *Trematopygus*)

##### Distribution

England, Scotland

#### Polyblastus (Polyblastus) pedalis

(Cresson, 1864)

Tryphon pedalis Cresson, 1864
carbonarius misident.
rhenanus Ulbricht, 1926 unavailable
rufifemur Hedwig, 1943 unavailable

##### Distribution

England, Scotland

##### Notes

British specimens belong to the subspecies *carbonator* Kasparyan, 1970.

#### Polyblastus (Polyblastus) pinguis

(Gravenhorst, 1920)

Ichneumon pinguis Gravenhorst, 1920
petryi Schmiedeknecht, 1912

#### Polyblastus (Polyblastus) subalpinus

Holmgren, 1857

##### Distribution

Scotland

#### Polyblastus (Polyblastus) tener

Habermehl, 1909

##### Distribution

England, Scotland, Wales

#### Polyblastus (Polyblastus) tuberculatus

Teunissen, 1953

##### Distribution

England

##### Notes

added by Shaw and Kasparyan (2005)

#### Polyblastus (Polyblastus) varitarsus

(Gravenhorst, 1829)

Tryphon varitarsus Gravenhorst, 1829
strobilator misident.
albovinctus (Gravenhorst, 1829, *Tryphon*)
affinis Woldstedt, 1874
subtilis Thomson, 1883
variitarsus Dalla Torre, 1901 preocc.
rufus Kiss, 1926

##### Distribution

England, Scotland, Wales, Ireland, Isle of Man

#### Polyblastus (Polyblastus) wahlbergi

Holmgren, 1857

wesmaeli Holmgren, 1857

##### Distribution

England, Scotland, Ireland

#### Smicroplectrus

Thomson, 1883

MICROPLECTRON Förster, 1869 preocc.

#### Smicroplectrus bohemani

(Holmgren,1857)

Exenterus bohemani Holmgren,1857

##### Distribution

England

#### Smicroplectrus erosus

(Holmgren,1857)

Exenterus erosus Holmgren,1857

##### Distribution

England

#### Smicroplectrus excisus

Kerrich, 1952

##### Distribution

England

##### Notes

some distribution data from Kerrich (1962)

#### Smicroplectrus heinrichi

Kerrich, 1952

##### Distribution

Scotland, Ireland

#### Smicroplectrus jucundus

(Holmgren, 1857)

Exenterus jucundus Holmgren, 1857

##### Distribution

England

#### Smicroplectrus nigricornis

Kasparyan, 1976

##### Distribution

England, Scotland

##### Notes

added by Shaw and Kasparyan (2005)

#### Smicroplectrus perkinsorum

Kerrich, 1952

##### Distribution

England

#### Smicroplectrus quinquecinctus

(Gravenhorst, 1820)

Ichneumon quinquecinctus Gravenhorst, 1820
trianguligena Kerrich, 1952

##### Distribution

England, Scotland

#### Tryphon

Fallén, 1813

##### Notes

Distribution data from Fitton (1975) and Shaw and Kasparyan (2005).

Doubtfully placed species of *Tryphon* (Fitton 1976):

[*flavilabris* Stephens, 1835 nom. dub., from England]

[*thoracicus* Stephens, 1835 nom. dub., from England]

[*zonatus* Stephens, 1835 nom. dub., from England]

#### Stenocrotaphon

Kasparyan, 1969

#### Tryphon (Stenocrotaphon) obtusator

(Thunberg, 1824)

Ichneumon obtusator Thunberg, 1824
Tryphon (Stenocrotaphon) obtusator ?*subrufus* (Gmelin, 1790, *Ichneumon*)
consobrinus Holmgren, 1857

##### Distribution

England

#### Tryphon (Stenocrotaphon) subsulcatus

Holmgren, 1857

##### Distribution

England

#### Symboethus

Förster, 1869

#### Tryphon (Symboethus) bidentatus

Stephens, 1835

incestus Holmgren, 1857
incertus Brischke, 1871
tricolor Rudow, 1910 preocc.
abnormis Habermehl, 1925
rufescens (Kiss, 1926, *Erromenus*)

##### Distribution

England, Scotland, Wales

##### Notes

Shaw and Kasparyan (2005) list the vice counties separately for the two forms of *bidentatus*, the typical form and f. *rufifemur* Kasparyan, the latter only recorded from Scotland and possibly belonging to a separate species, *T. hinzi* (Heinrich, 1953, *Symboethus*) (but see the note regarding *Tryphon duplicatus*).

#### Tryphon (Symboethus) brunniventris

Gravenhorst, 1829

##### Distribution

England, Scotland

#### Tryphon (Symboethus) duplicatus

(Heinrich, 1953)

Symboethus duplicatus Heinrich, 1953
discedens (Heinrich, 1953, *Symboethus*)

##### Distribution

England, Scotland

##### Notes

H.K. Townes (pers. comm. to M.G. Fitton) regarded *duplicatus* as a junior synonym of *exclamationis*, the correct name for the species here called *duplicatus* being *Tryphon hinzi* (Heinrich, 1953). Townes also regarded ‘*duplicatus*’ (=*hinzi* sensu Townes) as comprising two species, one with red hind femora and one with black hind femora, also differing in characteristics of the egg. However, Shaw and Kasparyan (2005) regard a form of *bidentatus* with red hind femora as possibly being synonymous with *hinzi*. Further clarification is needed.

#### Tryphon (Symboethus) exclamationis

Gravenhorst, 1829

connectens Roman, 1909

##### Distribution

England

#### Tryphon (Symboethus) fulviventris

Holmgren, 1857

##### Distribution

England, Scotland

##### Notes

Added by Kasparyan (1973), whose inclusion of British specimens was overlooked by Fitton (Fitton 1975, Fitton 1978).

#### Tryphon (Symboethus) heliophilus

Gravenhorst, 1829

bicornutus Holmgren, 1856
confinis Holmgren, 1856
maculatus (Pfankuch, 1924, *Symboethus*) unavailable

##### Distribution

England

#### Tryphon

Fallén, 1813

OTITOCHILUS Förster, 1869
PSILOSAGE Förster, 1869

#### Tryphon (Tryphon) abditus

Kasparyan, 1969

pleuralis Thomson, 1883 preocc.

##### Distribution

England, Scotland

#### Tryphon (Tryphon) atriceps

Stephens, 1835

ephippium Holmgren, 1857

##### Distribution

England, Wales

#### Tryphon (Tryphon) bidentulus

Thomson, 1883

separandus Schmiedeknecht, 1912

##### Distribution

England

#### Tryphon (Tryphon) latrator

(Fabricius, 1781)

Ichneumon latrator Fabricius, 1781
auricularis Thomson, 1883 synonymy by Horstmann (2001a)

##### Distribution

England, Scotland, Wales, Ireland, Isle of Man

#### Tryphon (Tryphon) nigripes

Holmgren, 1857

##### Distribution

England

#### Tryphon (Tryphon) relator

(Thunberg, 1824)

Ichneumon relator Thunberg, 1824
vulgaris Holmgren, 1857
erythrogaster Thomson, 1883

##### Distribution

England, Scotland

#### Tryphon (Tryphon) rutilator

(Linnaeus, 1761)

Ichneumon rutilator Linnaeus, 1761
impraegnator (Schrank, 1781, *Ichneumon*)
cepae (Geoffroy, 1785, *Ichneumon*)
anodon (Schrank, 1802, *Ichneumon*)
ceparum (Schrank, 1802, *Ichneumon*)
insultator (Gravenhorst, 1807, *Ichneumon*)
quadratus Stephens, 1835

##### Distribution

England, Scotland, Wales

#### Tryphon (Tryphon) signator

Gravenhorst, 1829

facialis Stephens, 1835
nigrifacies Ulbricht, 1926 unavailable

##### Distribution

England, Scotland, Wales, Isle of Man

#### Tryphon (Tryphon) thomsoni

Roman, 1939

##### Distribution

England, Scotland, Wales

#### Tryphon (Tryphon) trochanteratus

Holmgren, 1857

##### Distribution

England, Scotland, Wales, Isle of Man

### Xoridinae

#### XORIDINAE

Shuckard, 1840

##### Notes

Some distribution data from Gauld and Fitton (1981).

#### Ischnoceros

Gravenhorst, 1829

MITROBORIS Holmgren, 1859

#### Ischnoceros caligatus

(Gravenhorst, 1829)

Xylonomus caligatus Gravenhorst, 1829
seticornis Kriechbaumer, 1879

##### Distribution

England, Scotland, Ireland

#### Ischnoceros rusticus

(Geoffroy, 1785)

Ichneumon rusticus Geoffroy, 1785
striatus (Brullé, 1846, *Odontomerus*)
cornutus (Ratzeburg, 1848, *Xorides*)
filicornis Kriechbaumer, 1879
caper (Hedwig, 1957, *Eclytus*)

##### Distribution

England, Scotland, Wales, Ireland

#### Odontocolon

Cushman, 1942

ODONTOMERUS Gravenhorst, 1829 preocc.

#### Odontocolon dentipes

(Gmelin, 1790)

Ichneumon dentipes Gmelin, 1790
femoratum (Olivier, 1811, *Ophion*)
pinetorum (Thomson, 1877, *Odontomerus*)

##### Distribution

England, Scotland, Wales, Ireland

#### Odontocolon quercinum

(Thomson, 1877)

Odontomerus quercinus Thomson, 1877
liogaster (Szépligeti, 1914, *Odontomerus*)
simile (Habermehl, 1920, *Odontomerus*)
brunneiventre (Telenga, 1930, *Odontomerus*)

##### Distribution

England

#### Xorides

Latreille, 1809

XYLONOMUS Gravenhorst, 1829
GONOPHONUS Förster, 1869
MOEROPHORA Förster, 1869
RHADINA Förster, 1869 preocc.
SICHELIA Förster, 1869
STEROTRICHUS Förster, 1869
RHADINOPIMPLA Schulz, 1911
NEOXYLONOMUS Clément, 1938
XYLONOMINUS Clément, 1938

##### Notes

The British species have usually been separated into the subgenera *Moerophora* (containing *rufipes* and *rusticus*) and *Xorides* s. str. (containing the others) but Wahl (1997) has placed the subgenera of Xorides in synonymy.

#### Xorides brachylabis

(Kriechbaumer, 1889)

Xylonomus brachylabis Kriechbaumer, 1889
brachylabris misspelling

##### Distribution

England

#### Xorides csikii

Clément, 1938

##### Distribution

England

#### Xorides fuligator

(Thunberg, 1824)

Ichneumon fuligator Thunberg, 1824
sordator (Thunberg, 1824, *Ichneumon*)
pilicornis (Gravenhorst, 1829, *Xylonomus*)

##### Distribution

England, Wales

#### Xorides gravenhorstii

(Curtis, 1831)

Xylonomus gravenhorstii Curtis, 1831
securicornis (Holmgren, 1860, *Xylonomus*)
glyptus (Thomson, 1877, *Xylonomus*)
clavicornis (Kriechbaumer, 1879, *Xylonomus*)
distinguendus (Magretti, 1884, *Xylonomus*)
hungaricus (Szépligeti, 1899, *Sichelia*)
seticornis (Strobl, 1902, *Xylonomus*)
rufoscutellatus (Habermehl, 1918, *Xylonomus*)
kokujevi (Meyer, 1922, *Xylonomus*)
rufus (Kiss, 1924, *Xylonomus*)
caucasicus (Shestakov, 1925, *Xylonomus*)
romani Clément, 1938

##### Distribution

England, Wales, Ireland

#### Xorides irrigator

(Fabricius, 1793)

Ichneumon irrigator Fabricius, 1793
planus Šedivý, 1958

##### Distribution

England

#### Xorides niger

(Pfeffer, 1913)

Xylonomus niger Pfeffer, 1913
bicolor Clément, 1938

##### Distribution

England

#### Xorides praecatorius

(Fabricius, 1793)

Ichneumon praecatorius Fabricius, 1793
Xorides praecatorius ?*articulatus* (Geoffroy, 1785, *Ichneumon*)
Xorides praecatorius ?*falsatorius* (Olivier, 1792, *Ichneumon*)
parvulus (Gravenhorst, 1829, *Xylonomus*)
praecatorius (Marshall, 1872, *Xylonomus*) preocc.
rufopictus (Kiss, 1926, *Xylonomus*)
ruficoxis (Kiss, 1929, *Xylonomus*) preocc.
temporalis (Kiss, 1929, *Xylonomus*)
holsaticus Heinrich, 1951

##### Distribution

England, Scotland

#### Xorides rufipes

(Gravenhorst, 1829)

Xylonomus rufipes Gravenhorst, 1829

##### Distribution

England

#### Xorides rusticus

(Desvignes, 1856)

Xylonomus rusticus Desvignes, 1856

##### Distribution

England

## Supplementary Material

Supplementary material 1 Checklist of British and Irish Ichneumonidae Data type: Excel spreadsheet
Brief description: Excel spreadsheet version of the British and Irish Ichneumonidae checklist
File: oo_92300.xlsx Broad, G.R.

Supplementary material 2 Checklist of British and Irish Ichneumonidae Data type: Word document
Brief description: text version of the British and Irish Ichneumonidae checklist
File: oo_92298.docx Broad, G.R.

## Figures and Tables

**Figure 1a.**
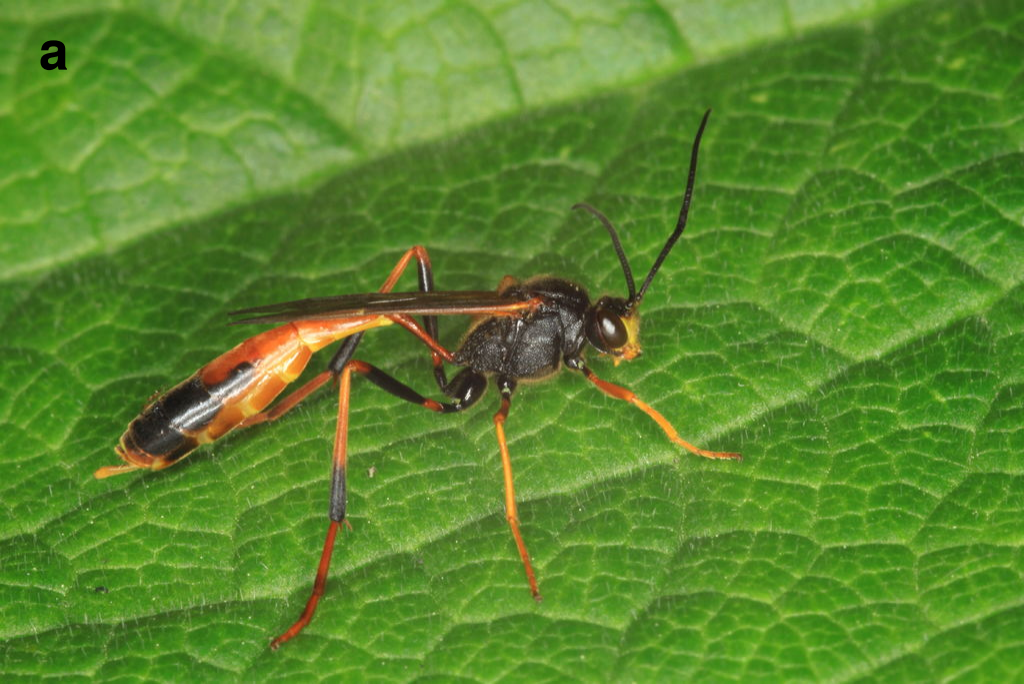
Anomaloninae: *Erigorgus cerinops* (Gravenhorst) female (courtesy of B. Formstone)

**Figure 1b.**
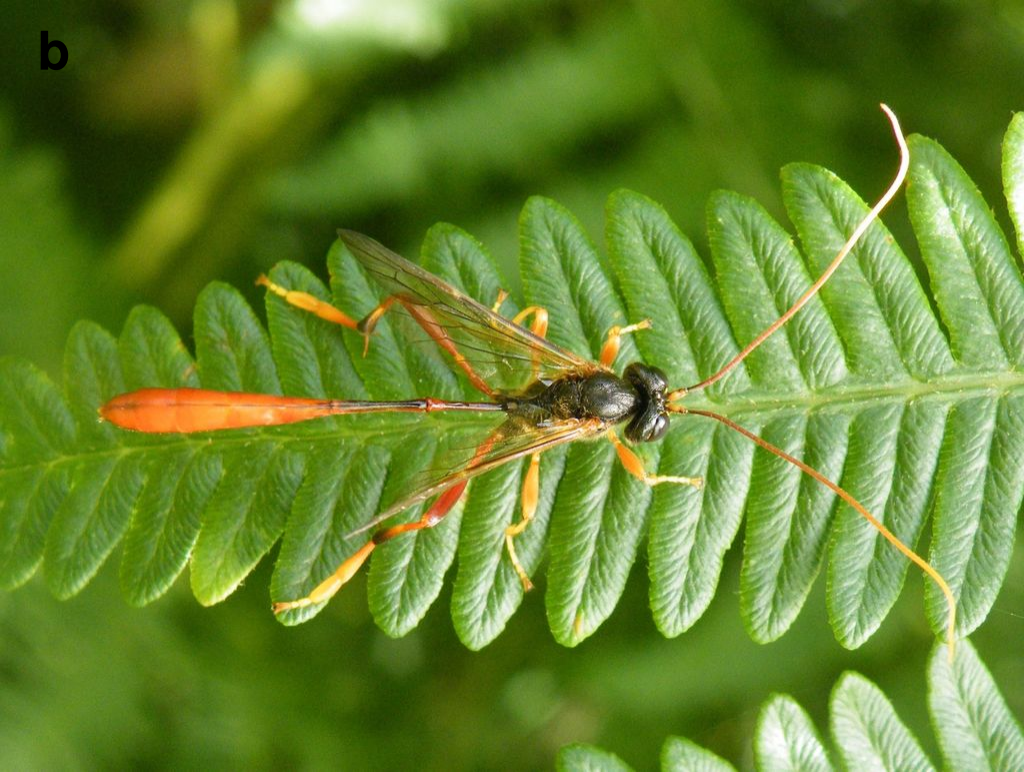
Anomaloninae: *Heteropelma amictum* (Fabricius) male (courtesy of I. Middlebrook)

**Figure 1c.**
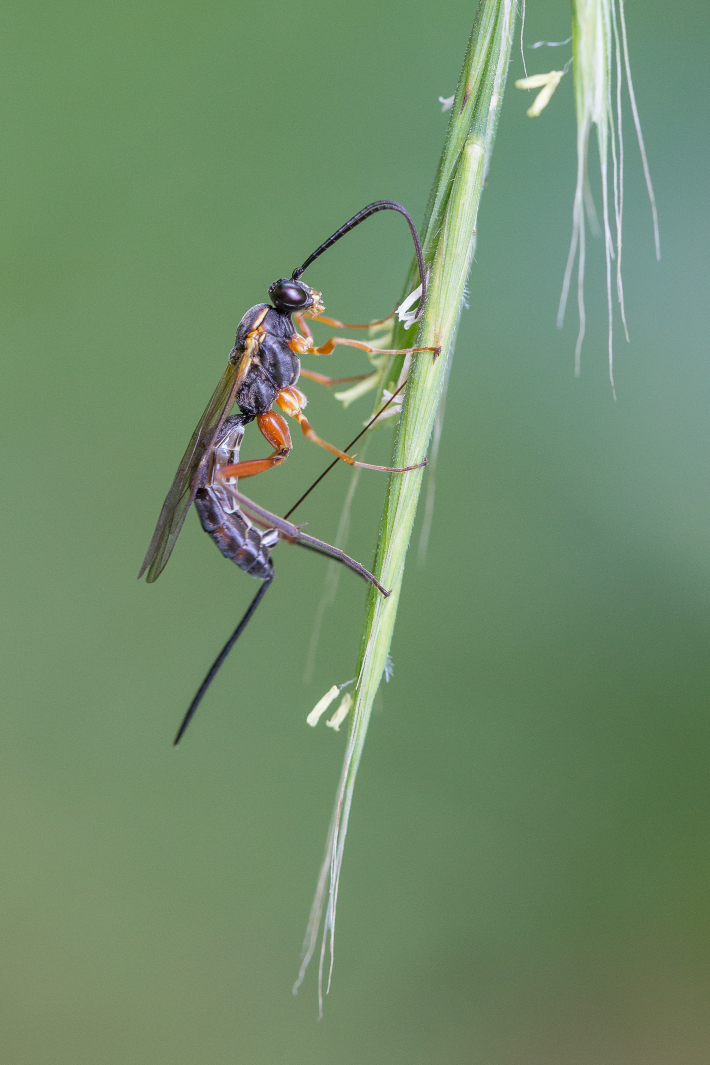
Banchinae: *Lissonota lineolaris* (Gmelin) female probing for *Apamea* (Lepidoptera: Noctuidae) larva (courtesy of P. Adams)

**Figure 1d.**
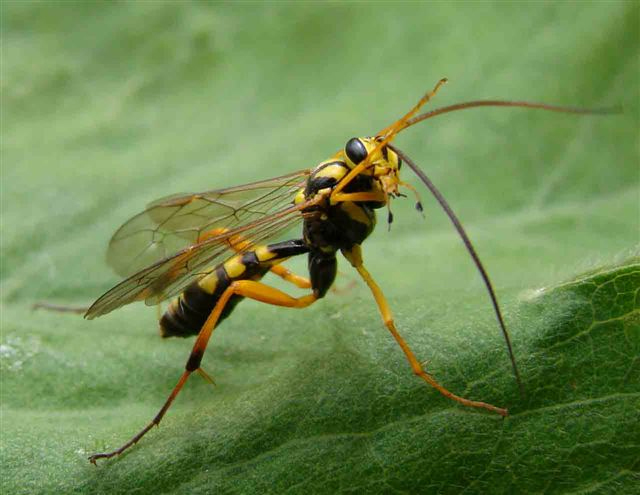
Banchinae: *Banchus volutatorius* (Linnaeus) male (courtesy of A. Watson Featherstone)

**Figure 2a.**
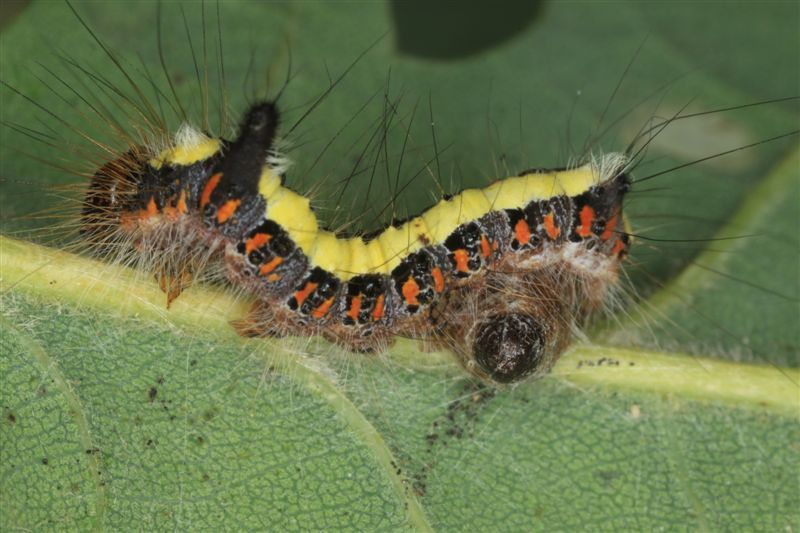
Campopleginae: cocoon of *Hyposoter* sp. ex *Acronicta psi* (Linnaeus) (Lepidoptera: Noctuidae) (courtesy of B. Formstone)

**Figure 2b.**
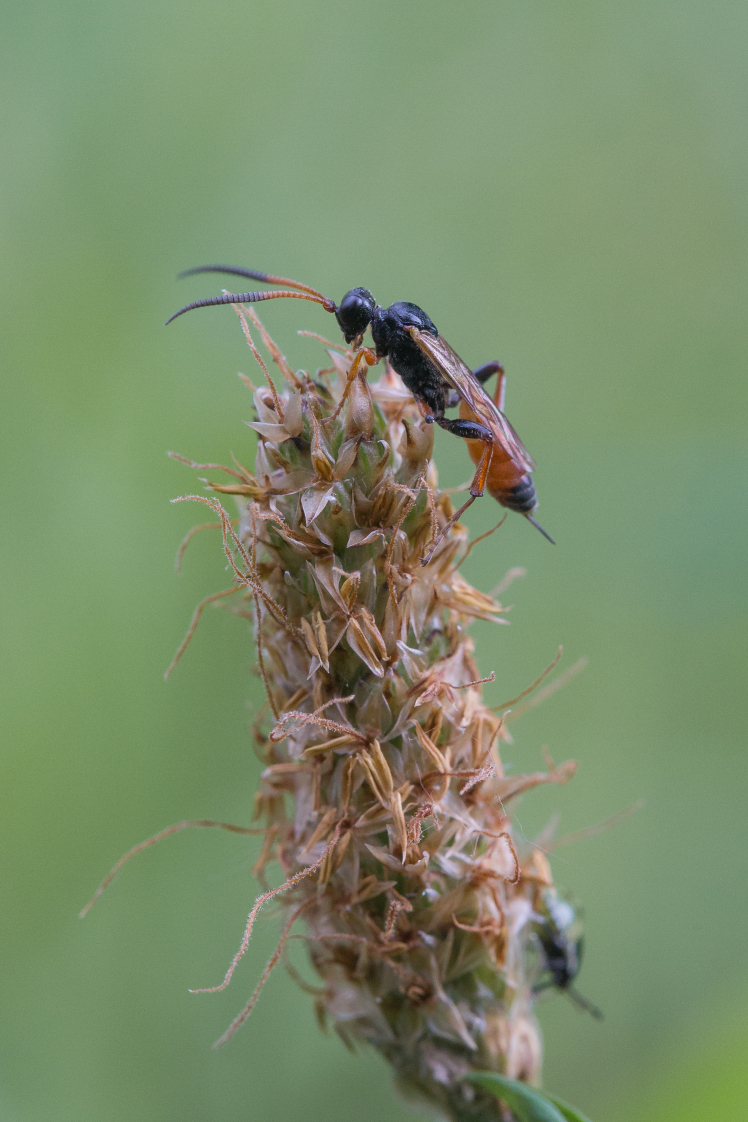
Cryptinae: *Colocnema rufina* (Gravenhorst) female

**Figure 2c.**
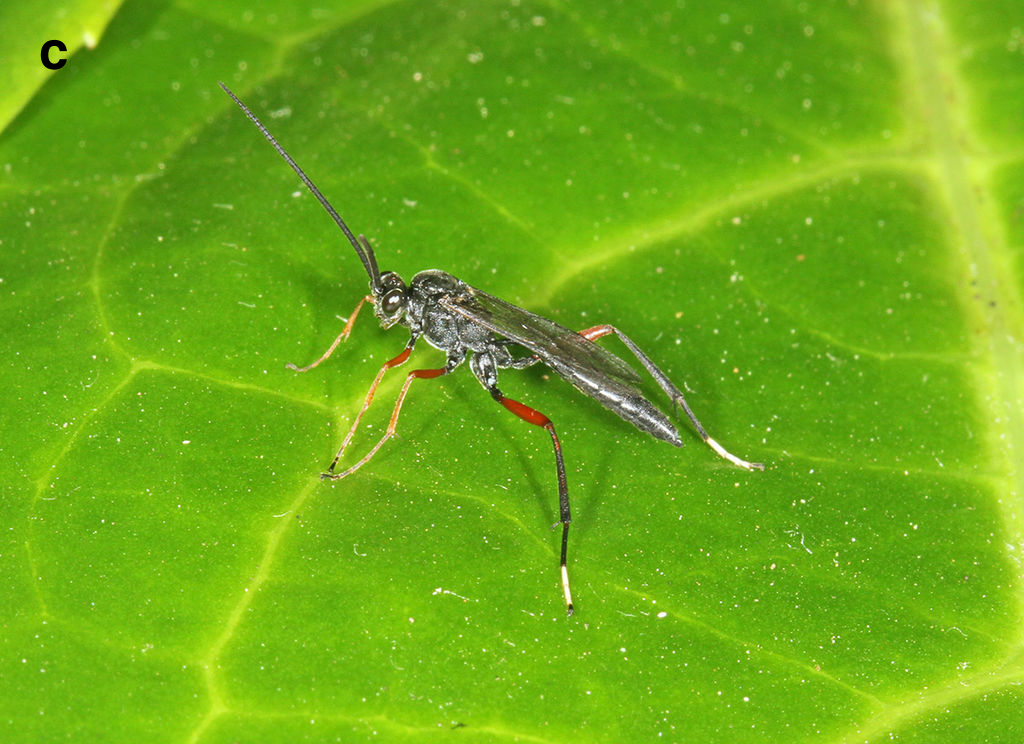
Cryptinae: *Polytribax perspicillator* (Gravenhorst) male (courtesy of J. Early)

**Figure 2d.**
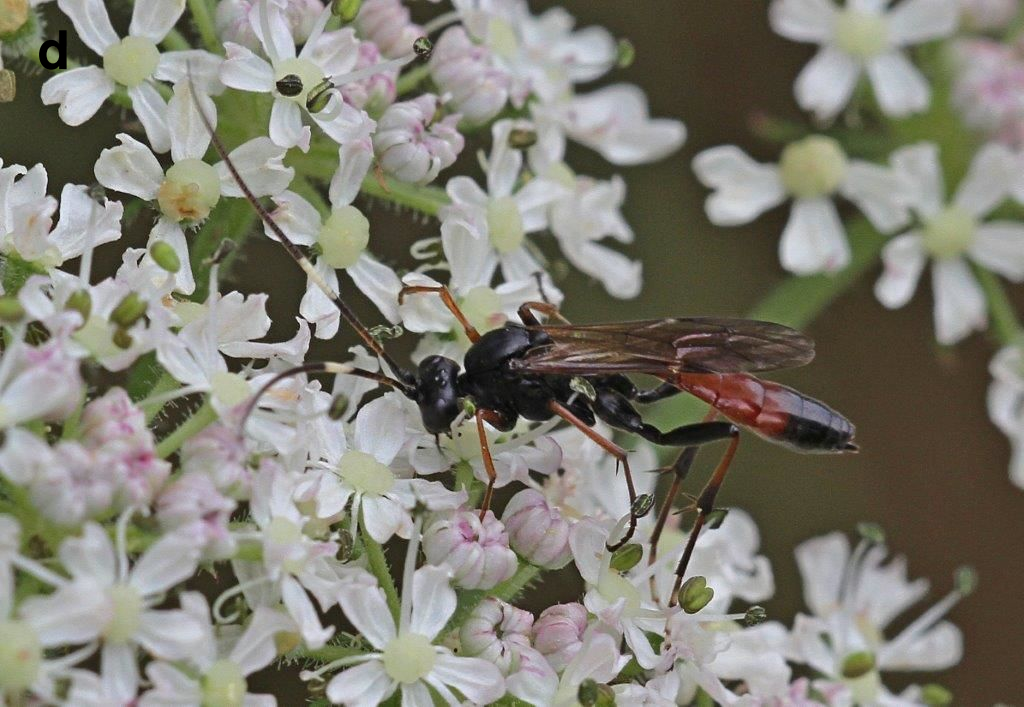
Ctenopelmatinae: *Euryproctus* sp. female (courtesy of D. Bateson)

**Figure 3a.**
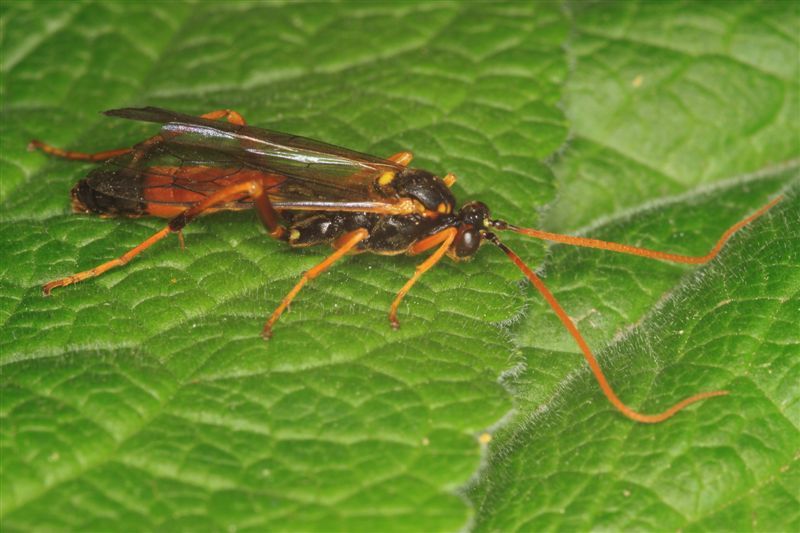
Ctenopelmatinae: *Protarchus melanurus* (Thomson) female (courtesy of B. Formstone)

**Figure 3b.**
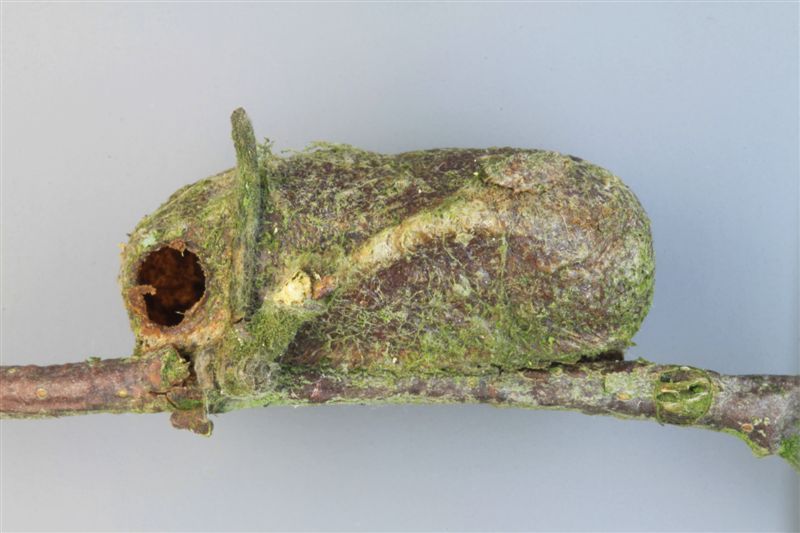
Ctenopelmatinae: *Cimbex* (Hymenoptera: Cimbicidae) cocoon with *Protarchus melanurus* (Thomson) emergence hole (courtesy of B. Formstone)

**Figure 3c.**
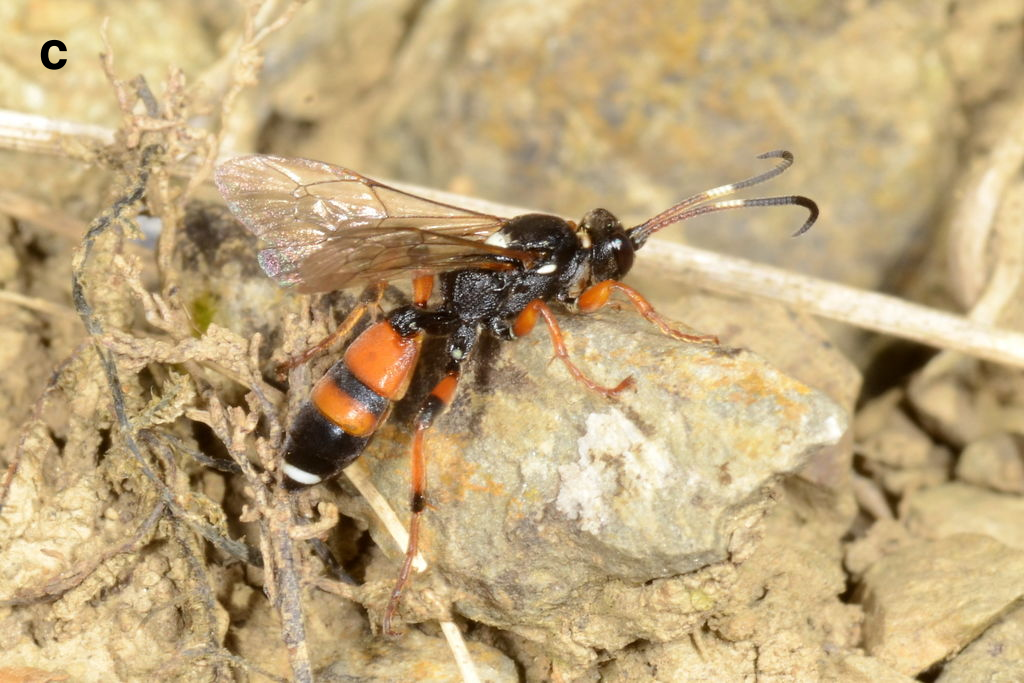
Ichneumoninae: *Ichneumon sarcitorius* Linnaeus female (courtesy of J. Bingham)

**Figure 3d.**
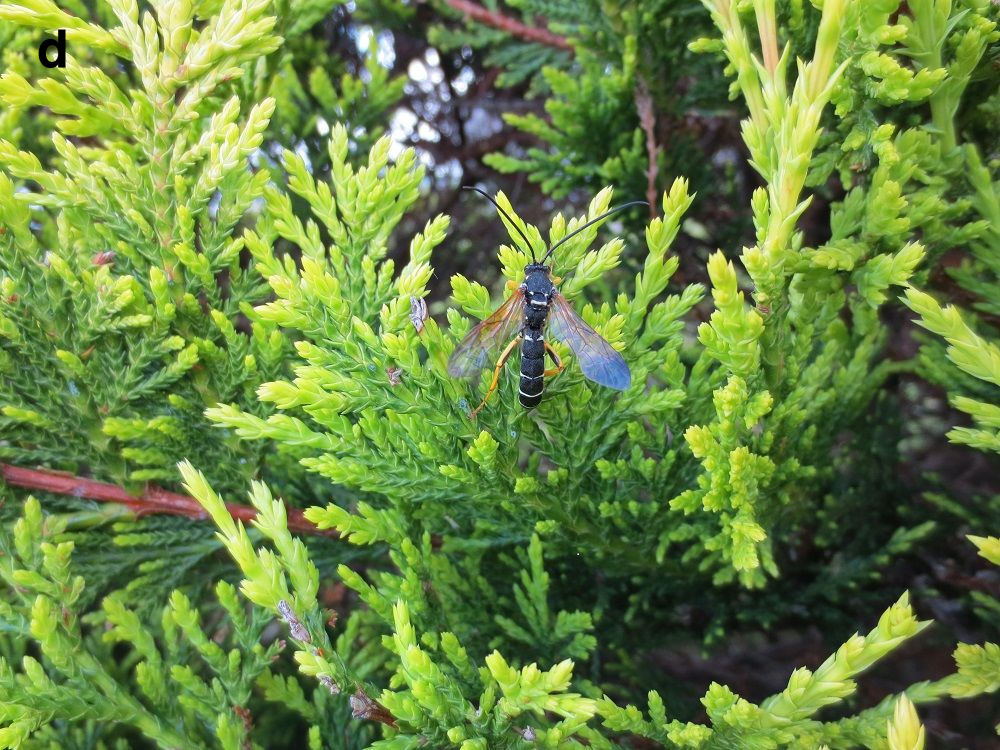
Metopiinae: *Metopius dentatus* (Fabricius) ex *Lasiocampa quercus callunae* Palmer (Lepidoptera: Lasiocampidae) pupa (courtesy of F. Stark)

**Figure 4a.**
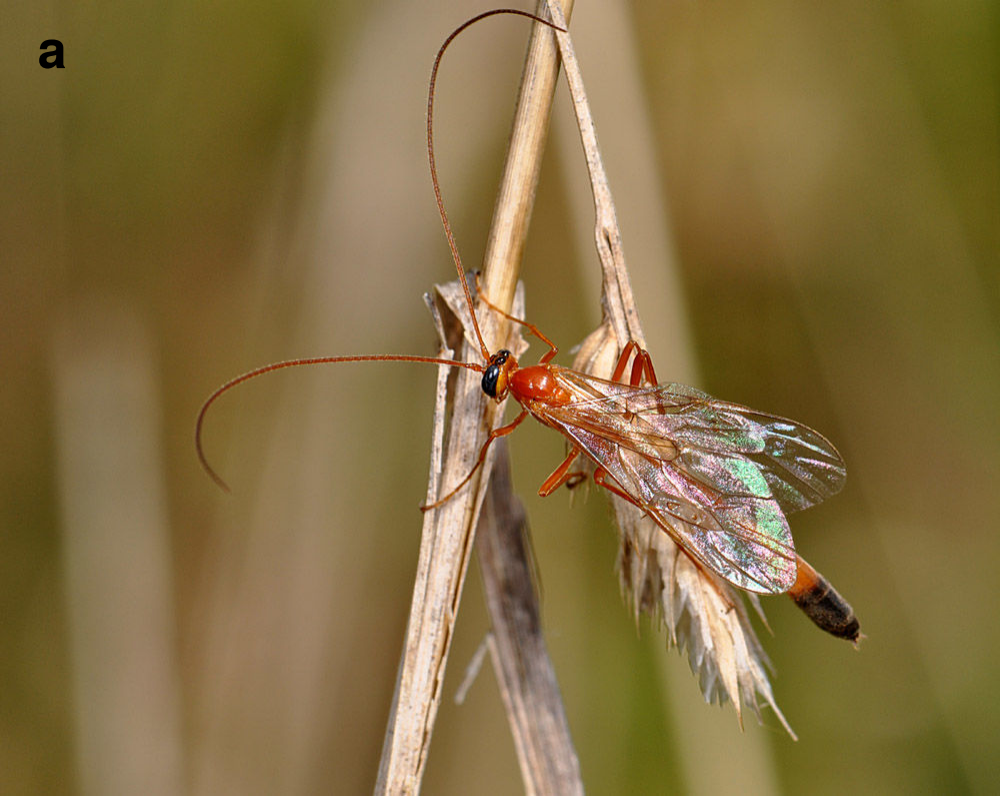
Ophioninae: *Enicospilus ramidulus* (Linnaeus) (courtesy of P. Brock)

**Figure 4b.**
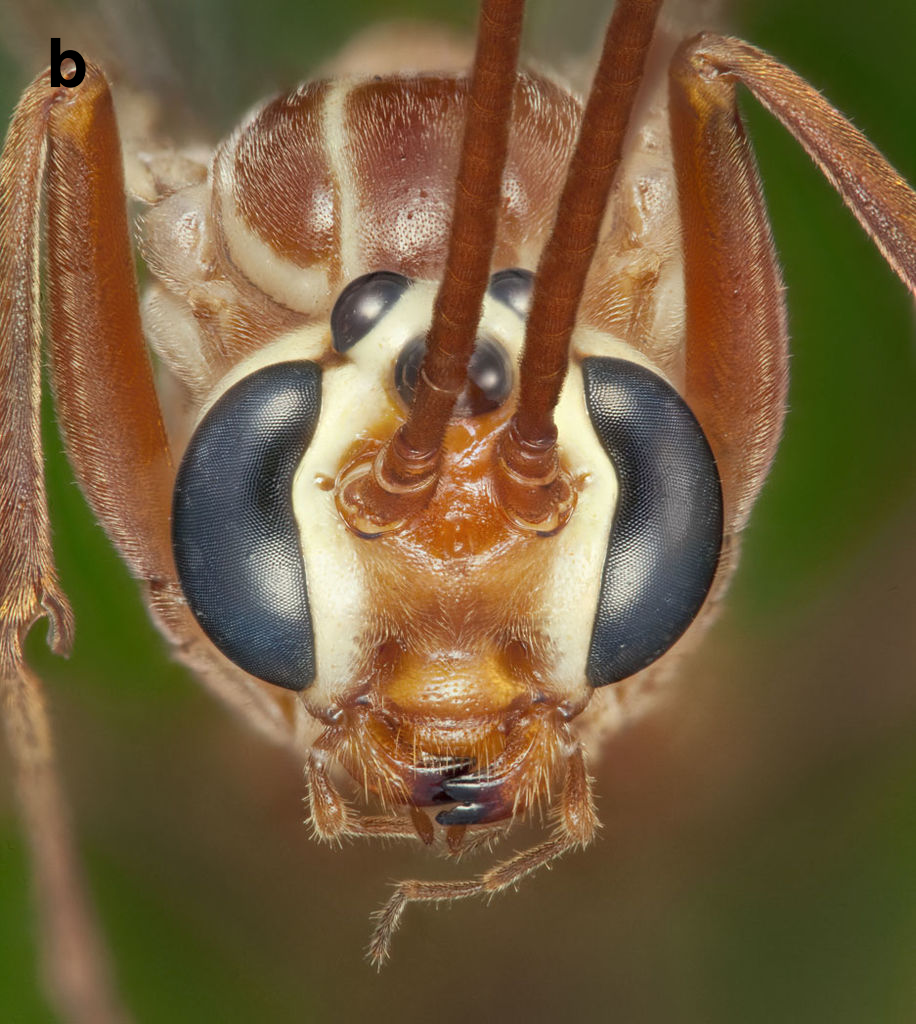
Ophioninae: *Ophion obscuratus* Fabricius (courtesy of H. Taylor, BMNH)

**Figure 4c.**
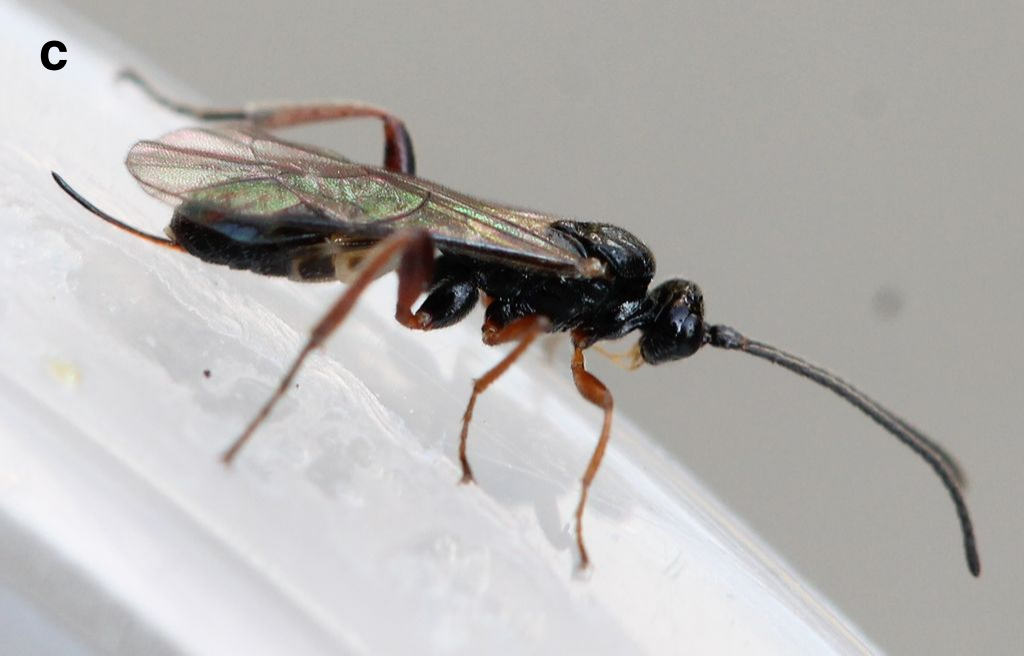
Orthocentrinae: *Entypoma* sp. (courtesy of T. LeGrand)

**Figure 4d.**
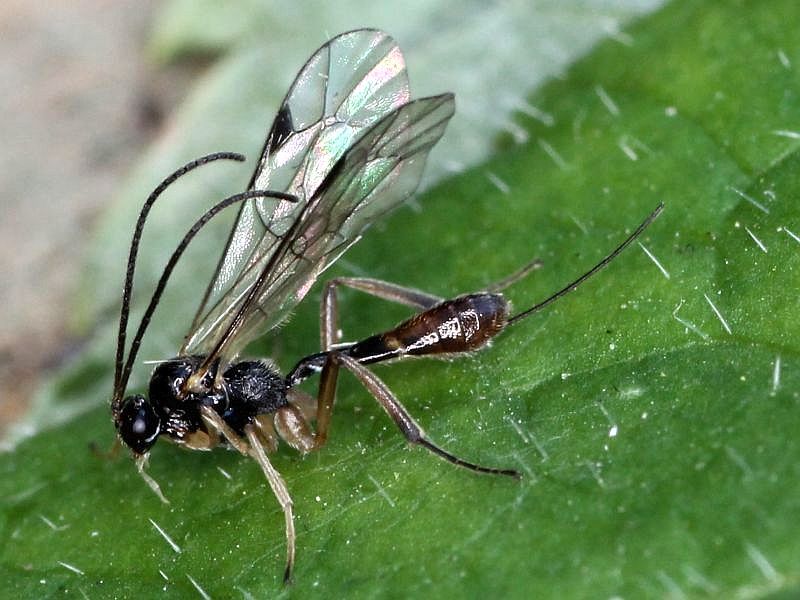
Orthocentrinae: *Plectiscidea* sp. (courtesy of M. Stemmer)

**Figure 5a.**
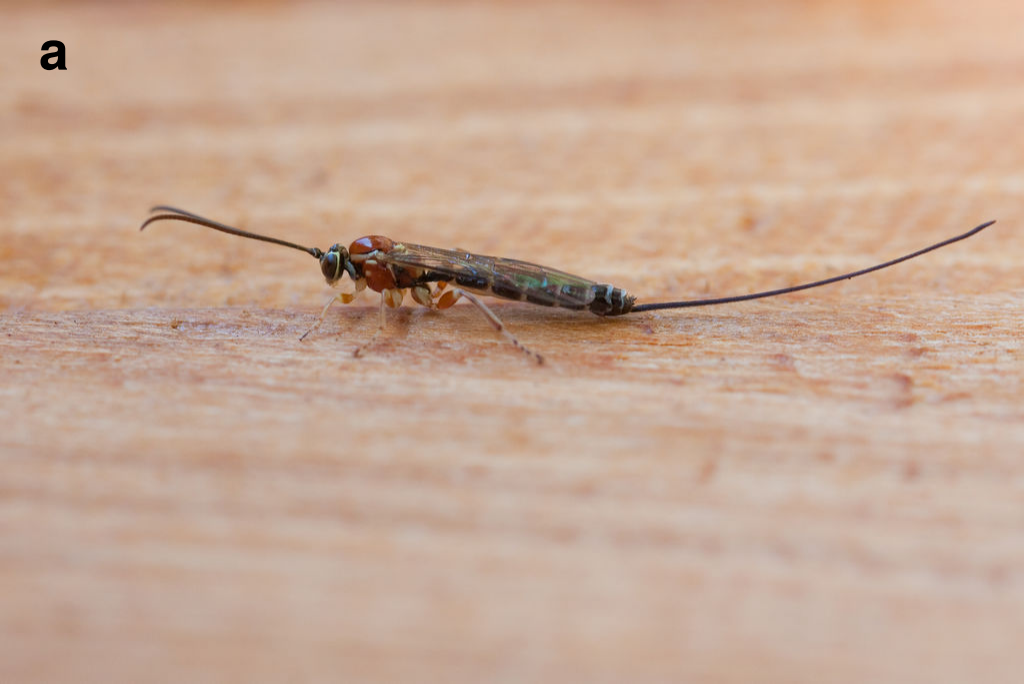
Pimplinae: *Perithous scurra* (Panzer) female (courtesy of P. Adams)

**Figure 5b.**
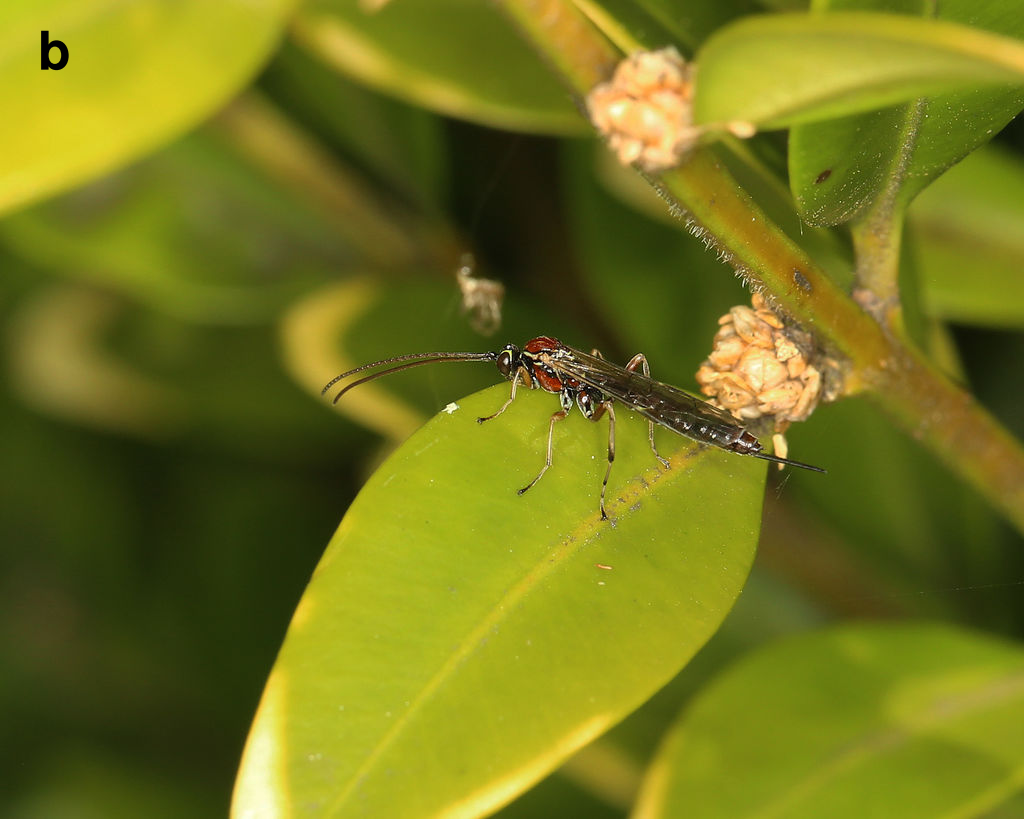
Pimplinae: Tromatobia lineatoria (Villers) female (courtesy of J. Davison)

**Figure 5c.**
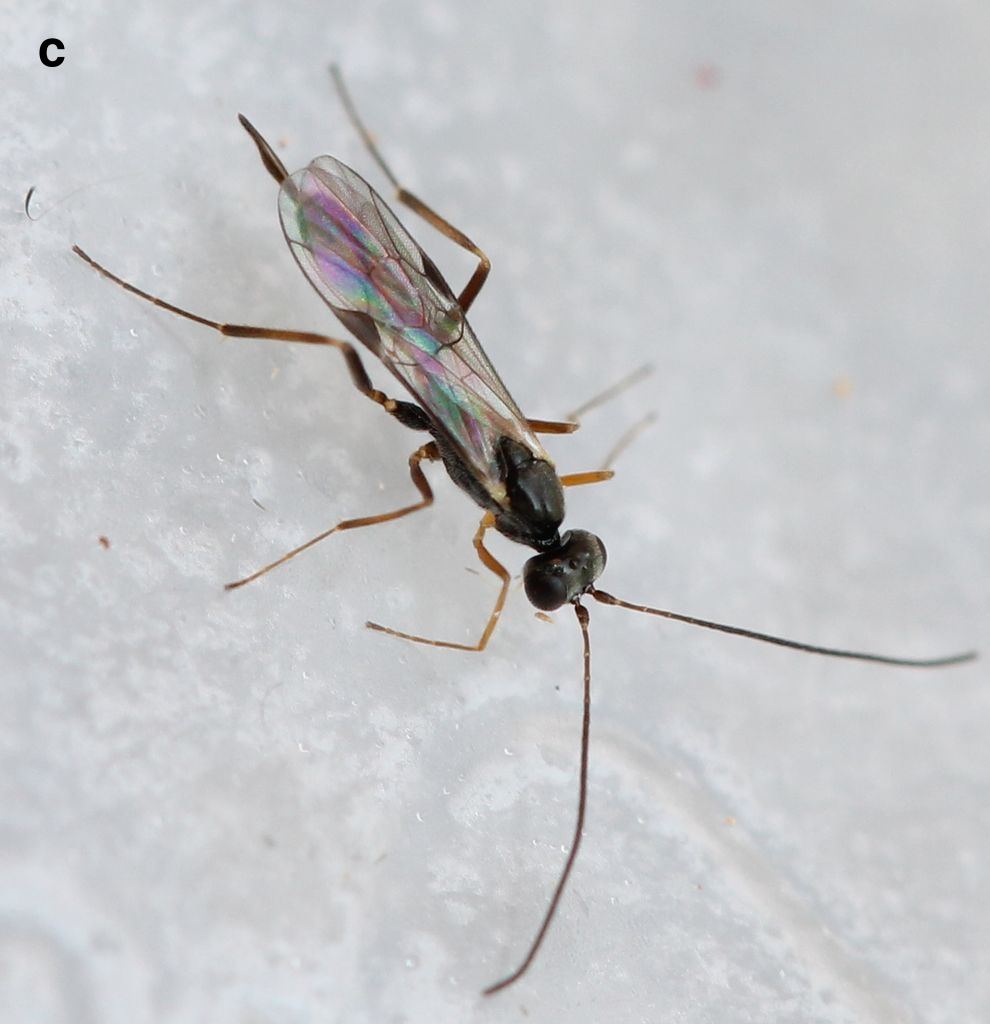
Tryphoninae: *Thymaris niger* (Taschenberg) female (courtesy of T. LeGrand)

**Figure 5d.**
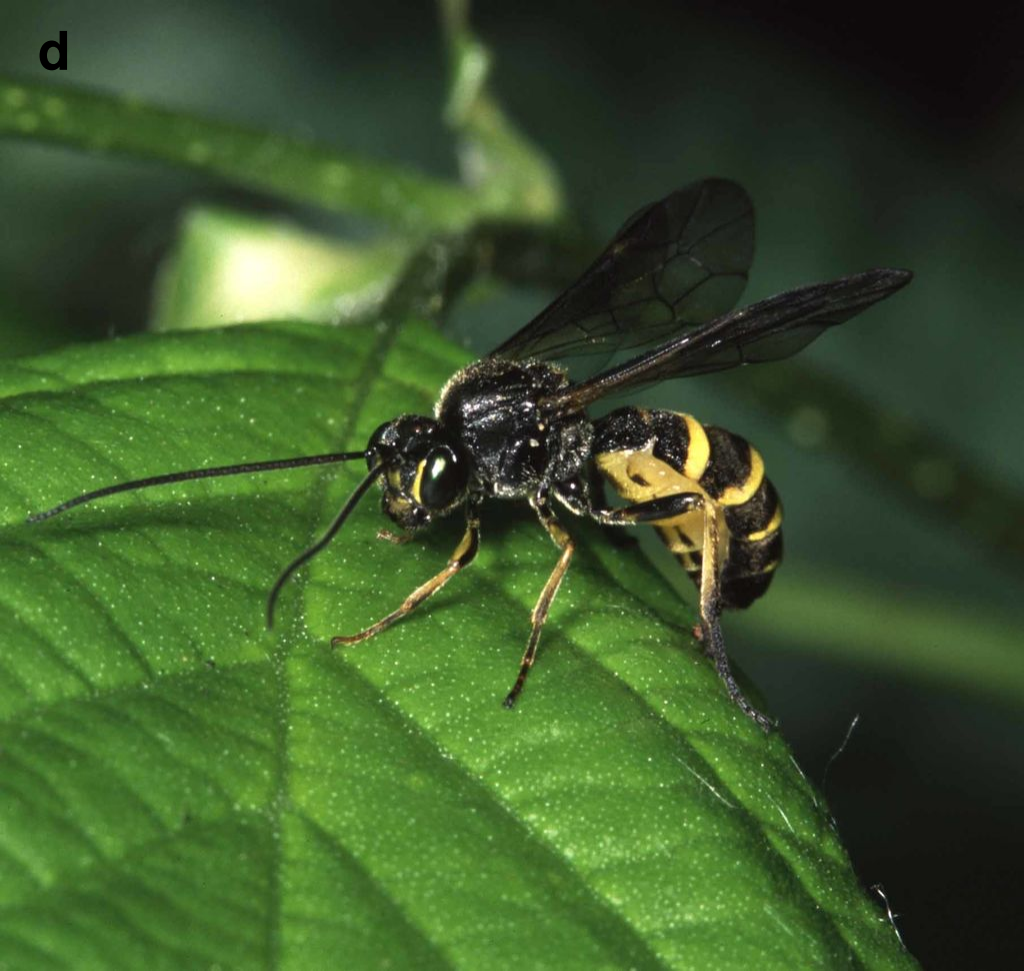
Tryphoninae: *Excavarus* sp. (details unfortunately lost)

**Table 1.** Numbers of confirmed British and Irish Ichneumonidae broken down by subfamily and country. Totals do not include manuscript names or uncertain identifications.

**subfamily**	**total valid species**	**England**	**Scotland**	**Wales**	**Ireland**	**Isle of Man**
Acaenitinae	6	3	2			
Adelognathinae	19	18	8	4	11	2
Agriotypinae	1	1	1	1		
Alomyinae	2	2	1		1	1
Anomaloninae	38	36	20	14	13	3
Banchinae	132	122	65	21	55	5
Campopleginae	361	284	169	41	122	22
Collyriinae	2	2				
Cremastinae	15	15	1	2	7	
Cryptinae	529	404	245	123	140	40
Ctenopelmatinae	295	244	147	35	30	19
Cylloceriinae	5	5	3		4	
Diacritinae	1	1	1		1	
Diplazontinae	60	57	45	35	40	22
Eucerotinae	3	3	2		2	
Hybrizontinae	2	2		1		
Ichneumoninae	383	334	181	81	176	28
Lycorininae	1	1	1		1	
Mesochorinae	86	73	25	4	11	3
Metopiinae	76	67	42	10	14	1
Microleptinae	3	3	3	2	2	
Neorhacodinae	1	1	1		1	
Ophioninae	27	26	19	15	10	1
Orthopelmatinae	2	2	1	2	1	
Oxytorinae	2	2	2	2		
Pimplinae	109	103	64	52	56	20
Poemeniinae	6	6	1	2	2	
Rhyssinae	2	2	2	1	1	1
Stilbopinae	5	4	4	1	3	
Tersilochinae	81	73	27	9	46	1
Tryphoninae	179	153	108	37	53	20
Xoridinae	13	13	4	3	4	
	**2447**	**2062**	**1195**	**498**	**807**	**189**
