# Supplementary material for: Checklist of British and Irish Hymenoptera - Ichneumonidae
Source: Biodivers Data J. 2016 Jul 5;(4):e9042. doi: 10.3897/BDJ.4.e9042 (PMC5039340; doi:10.3897/BDJ.4.e9042)
Supplement: Supplementary material 2 — Checklist of British and Irish Ichneumonidae [file biodiversity_data_journal-4-e9042-s002.docx]

Family **Ichneumonidae** Latreille, 1802

Gavin R. Broad

The basis of the classification of Ichneumonidae used here is the catalogue of Yu & Horstmann (1997). Deviations from this work, including more recent changes, are referenced in the checklist. Full synonymy for the Western Palaearctic is given, and for much of the Eastern Palaearctic, but synonyms described extralimitally have not been included. Synonymies published since Yu & Horstmann (1997) have been included and referenced.

Subfamily ACAENITINAE Förster, 1869[[1]](#footnote-2)

***ACAENITUS*** Latreille, 1809

*ACOENITES* Latreille, 1810

*ACOENITUS* Griffith, 1832

***dubitator*** (Panzer, 1800, *Ichneumon*) S

***AROTES*** Gravenhorst, 1829

*ASTHENOMERIS* Förster, 1869

*SPHALERUS* Kriechbaumer, 1878

***albicinctus*** Gravenhorst, 1829 E

*bifasciatus* (Kriechbaumer, 1878, *Sphalerus*)

*annulicornis* Kriechbaumer, 1894 Varga (2013)

***COLEOCENTRUS*** Gravenhorst, 1829

*MACROCOLEUS* Desvignes, 1850

***croceicornis*** (Gravenhorst, 1829, *Macrus*)[[2]](#footnote-3) E

***excitator*** (Poda, 1761, *Ichneumon*) S added by Shaw (1986)

*segmentator* (Fabricius, 1793, *Ichneumon*)

*gigantor* (Thunberg, 1824, *Ichneumon*)

*longiventris* (Gravenhorst, 1829, *Macrus*)

*segmentatrix* (Schulz, 1906, *Lissonota*)

***LEPTACOENITES*** Strobl, 1902

***notabilis*** (Desvignes, 1856, *Lampronota*)

*frauenfeldi* (Tschek, 1869, *Lissonota*)

*marginatus* (Kriechbaumer, 1899, *Heterolabis*)

*petiolaris* (Kriechbaumer, 1899, *Heterolabis*)

*tscheki* (Strobl, 1902, *Procinetus*)

***PHAENOLOBUS*** Förster, 1869

*CHORISCHIZUS* Förster, 1869

*MOLDACOENITUS* Constantineanu & Constantineanu, 1968

***terebrator*** (Scopoli, 1763, *Ichneumon*) E

*arator* (Rossi, 1790, *Ichneumon*)

Subfamily ADELOGNATHINAE Thomson, 1888[[3]](#footnote-4)

***ADELOGNATHUS*** Holmgren, 1857

*PAMMICRA* Förster, 1869

*NOTOMERIS* Förster, 1869

*CNEMISCHYS* Förster, 1869

*EPITROPUS* Rossem, 1990 Broad (2004)

***acantholydae*** Kasparyan, 1986 S NMS, det. Shaw, added here

***brevicornis*** Holmgren, 1857 E S I

*limbatus* Thomson, 1888

*montivagator* Aubert, 1976

***britannicus*** Perkins, 1943 E

***chrysopygus*** (Gravenhorst, 1829, *Hemiteles*) E S I M[[4]](#footnote-5)

Bennett *et al.* (2002)

*granulatus* Perkins, 1943

***difformis*** Holmgren, 1857 E NMS, det. Shaw, added here

***dorsalis*** (Gravenhorst, 1829, *Hemiteles*) E S W I M

*melanius* Roman, 1918

*insolitus* (Rossem, 1990, *Epitropus*) Broad (2004)

***laevicollis*** Thomson, 1883 E I

***leucotrochi*** Shaw & Wahl, 2014 E S added by Shaw & Wahl (2014)

***nigriceps*** Thomson, 1888 E W Askew (2000)

***nigrifrons*** Holmgren, 1857 E I added by Fitton *et al.* (1982)

***obscurus*** Kasparyan, 1986 E S added by Kasparyan (1990)

***pallipes*** (Gravenhorst, 1829, *Plectiscus*) E S I

*ruthei* Holmgren, 1857

*pallidipes* (Marshall, 1872, *Plectiscus*)

***pilosus*** Thomson, 1888 E

***punctulatus*** Thomson, 1883 E I

*chrysopygus* misident.

*pallipes* Holmgren, 1857 preocc.

*dimidiatus* Thomson, 1888

*pallidipes* Dalla Torre, 1901

***pusillus*** Holmgren, 1857 E W I

***stelfoxi*** Fitton, Gauld & Shaw, 1982 E S W I added by Fitton *et al*. (1982); Ely (2001)

***tenthredinarum*** (Giraud, 1872, *Plectiscus*) E

*nigricornis* Thomson, 1888

***tetratinctorius*** (Thunberg, 1824, *Ichneumon*) E I

*fasciatus* Thomson, 1883

*scabriculus* Thomson, 1883

***thomsoni*** Schmiedeknecht, 1911 E I

*thuringiacus* Schmiedeknecht, 1911

Subfamily AGRIOTYPINAE Haliday, 1838[[5]](#footnote-6)

***AGRIOTYPUS*** Curtis, 1832

*CROTOPUS* Holmgren, 1859

*ATOPOTYPUS* Chao, 1992 Bennett (2001)

***armatus*** Curtis, 1832 E S W

*abnormis* (Holmgren, 1859, *Crotopus*)

Subfamily ALOMYINAE Förster, 1869[[6]](#footnote-7)

***ALOMYA*** Panzer, 1806[[7]](#footnote-8)

*ALOMYIA* misspelling

*HALOMYA* Billberg, 1820

***debellator*** (Fabricius, 1775, *Ichneumon*) E S I M

*fischeri* (Schrank, 1776, *Ichneumon*)

*trituberculata* (Gmelin, 1790, *Ichneumon*)

*ovator* (Fabricius, 1793, *Ichneumon*)

*victor* Curtis, 1826

*nigra* Gravenhorst, 1829

*debellatrix* Schulz, 1906

*victrix* Schulz, 1906

*silvicola* Ulbricht, 1909 unavailable

***semiflava*** Stephens, 1835 E

*minor* Ulbricht, 1909 unavailable

*minor* Ulbricht, 1911 preocc.

Subfamily ANOMALONINAE Viereck, 1918[[8]](#footnote-9)

ANOMALINAE misspelling

Tribe ANOMALONINI Viereck, 1918

ANOMALINI misspelling

***ANOMALON*** Panzer, 1804

*TRACHYNOTUS* Gravenhorst, 1829 preocc.

*OCHLERUS* Gistel, 1848 preocc.

*NOTOTRACHYS* Marshall, 1872

*ANOMALUM* Schulz, 1906

*TRACHYOPTERUS* Morley, 1912

*PSEUDONOTOTRACHYS* Meyer, 1930

*MICROCREMASTUS* Hedwig, 1961

***cruentatum*** (Geoffroy, 1785, *Ichneumon*) E

*petiolatum* (Geoffroy, 1785, *Ichneumon*)

*foliator* (Fabricius, 1798, *Ophion*)

*cruentatum* Panzer, 1804 preocc.

*humerale* (Brullé, 1832, *Trachynotus*)

*epiphanii* Izquierdo, 1977

Tribe GRAVENHORSTIINI Enderlein, 1912

THERIONINI Viereck, 1918

***AGRYPON*** Förster, 1860

*AGRYPUM* Schulz, 1906

***anomelas*** (Gravenhorst, 1829, *Anomalon*) E[[9]](#footnote-10)

*anomalas* misspelling

*furtivum* Förster, 1860 Schnee (2008)

*trochanteratum* (Holmgren, 1860, *Anomalon*)

*rufipes* Kiss, 1926

***anxium*** (Wesmael, 1849, *Anomalon*) E S W I

*pictum* Kiss, 1924

***batis*** (Ratzeburg, 1855, *Anomalon*) E S I NMS, NHM, det. Schnee, added here

*serpentinum* Förster, 1860 Schnee (2008)

*stenostigma* (Thomson, 1892, *Anomalon*)

*segne* (Tosquinet, 1896, *Anomalon*)

***brachycerum*** Hellén, 1950[[10]](#footnote-11) E S NMS, NHM, det. Schnee, added here

***canaliculatum*** (Ratzeburg, 1844, *Anomalon*)[[11]](#footnote-12) E S

NMS, NHM, det. Schnee, added here

***clandestinum*** (Gravenhorst, 1829, *Anomalon*) E S I M

*delarvatum* misident.[[12]](#footnote-13)

*capillosum* (Hartig, 1838, *Anomalon*)

*affine* (Holmgren, 1857, *Anomalon*) preocc.

*brachypterum* Förster, 1860 Schnee (2008)

*clandestinum* Förster, 1860 preocc. Schnee (2008)

*ruficoxis* (Szépligeti, 1899, *Labrorychus*)

*flavopunctatum* (Kiss, 1933, *Blaptocampus*)

***flaveolatum*** (Gravenhorst, 1807, *Ophion*) E S W I

*cribrator* (Thunberg, 1824, *Ichneumon*)

*laedator* (Thunberg, 1824, *Ichneumon*)

*arquatum* (Gravenhorst, 1829, *Anomalon*)

*septentrionale* (Holmgren, 1857, *Anomalon*)

*aggressorium* Förster, 1860 Schnee (2008)

*confusum* Förster, 1860 Schnee (2008)

*elegantulum* Förster, 1860 Schnee (2008)

*rubricatum* Förster, 1860 Schnee (2008)

*rubricatum* (Förster, 1878, *Atrometus*) preocc.

***flexorium*** (Thunberg, 1824, *Ichneumon*)[[13]](#footnote-14) E S W I

*tenuicorne* (Gravenhorst, 1829, *Anomalon*)

*subclavatum* Förster, 1860 Schnee (2008)

*anaitidis* (Szépligeti, 1899, *Labrorychus*)

*sibiricum* (Shestakov, 1923, *Labrorychus*)

***gracilipes*** (Curtis, 1839, *Therion*) E W

*debile* (Wesmael, 1849, *Anomalon*)

*meridionator* Aubert, 1964 preocc.

***interstitiale*** Schnee, 1989 E S NMS, NHM, det. Schnee, added here

***minutum*** (Bridgman & Fitch, 1884, *Anomalon*)[[14]](#footnote-15) E S W

*minutum* (Bridgman, 1884, *Anomalon*) preocc.

***rugifer*** (Thomson, 1894, *Anomalon*) W NMS, det. Schnee, added here

***varitarsum*** (Wesmael, 1849, *Anomalon*) E S I

*cognatum* Förster, 1860 Schnee (2008)

*nigripes* (Bridgman, 1887, *Anomalon*)

*variitarsum* Dalla Torre, 1901 preocc.

**sp. H** Schnee, in prep. E W NMS, NHM, det. Schnee, added here

species excluded from the British and Irish list

[***interruptus*** (Desvignes, 1856, *Anomalon*[[15]](#footnote-16)]

***APHANISTES*** Förster, 1869

*ANOCHILACRUM* Enderlein, 1921

***bellicosus*** (Wesmael, 1849, *Anomalon*) E NMS, det. Schnee, added here

***gliscens*** (Hartig, 1838, *Anomalon*) E S W

*bellicosus* misident.[[16]](#footnote-17)

*xanthopus* misident.

*armatus* (Wesmael, 1849, *Anomalon*)

***ruficornis*** (Gravenhorst, 1829, *Anomalon*) E S W I

*excavatus* (Ratzeburg, 1848, *Anomalon*)

*wesmaeli* (Holmgren, 1856, *Anomalon*) E

***ATROMETUS*** Förster, 1869

**?*insignis*** Förster, 1878 E[[17]](#footnote-18)

*rubricatus* Förster, 1878 Schnee (2008)

*trachynotus* (Brauns, 1895, *Anomalon*)

*melanosoma* Szépligeti, 1899

*pulchellator* Aubert, 1971

***BARYLYPA*** Förster, 1869

*LAPHYCTES* Förster, 1869

*SARNTHEINIA* Dalla Torre, 1901

*HADROMANUS* Szépligeti, 1905

*MAGNIBUCCA* Morley, 1913

*TROCHISCOMERUS* Meyer, 1931

***delictor*** (Thunberg, 1824, *Ichneumon*) E S W

*perspicillator* (Gravenhorst, 1829, *Anomalon*)

*affinis* (Lucas, 1849, *Anomalon*)

*menyanthidis* (Boie, 1855, *Anomalon*)

*mesozona* (Förster, 1878, *Laphyctes*)

*genalis* (Thomson, 1892, *Anomalon*)

*frisiaca* Habermehl, 1922

*temporalis* Meyer, 1935

***propugnator*** (Förster, 1855, *Anomalon*)[[18]](#footnote-19) E W I

*insidiator* (Förster, 1878, *Laphyctes*) Schnee (2008)

*carinata* (Brischke, 1880, *Anomalon*) Schnee (2008)

*cylindrica* (Bridgman & Fitch, 1884, *Anomalon*)

*cylindrica* (Bridgman, 1884, *Anomalon*) preocc.

*rufa* (Habermehl, 1920, *Anomalon*) preocc.

Schnee (2008)

***rubricator*** (Szépligeti, 1899, *Laphyctes*) E NHM, Hunterian, det. Schnee, added here

*rubricatrix* (Schulz, 1906, *Sarntheinia*)

*rossica* Meyer, 1935

**?*uniguttata*** (Gravenhorst, 1829, *Anomalon*)[[19]](#footnote-20) E

***ERIGORGUS*** Förster, 1869[[20]](#footnote-21)

*SYMPRATIS* Förster, 1869

***cerinops*** (Gravenhorst, 1829, *Anomalon*) E S W I

*flavifrons* (Gravenhorst, 1807, *Ophion*) preocc.

*xantha* (Boie, 1855, *Anomalon*)

*facialis* (Boie, 1857, *Campoplex*)

*lapponicus* (Thomson, 1892, *Anomalon*)

*rufofemoralis* (Schmiedeknecht, 1936, *Anomalon*)

***fibulator*** (Gravenhorst, 1829, *Anomalon*) E W

NHM, det. Schnee, added here

*claripennis* (Thomson, 1892, *Anomalon*)

***foersteri*** (Mocsáry, 1897, *Anomalon*) E[[21]](#footnote-22) NMS, det. Schnee, added here

*melanops* misident.

*brevicorne* (Förster, 1855, *Anomalon*) preocc.

Schnee (2008)

***melanops*** (Förster, 1855, *Anomalon*) E S

*melanobata* misident.[[22]](#footnote-23)

*varians* (Brauns, 1895, *Anomalon*)

*flavimana* (Szépligeti, 1899, *Erigorgus*)

*interstitialis* (Szépligeti, 1899, *Erigorgus*)

*similis* (Szépligeti, 1899, *Erigorgus*)

*purpuratae* (Kriechbaumer, 1900, *Erigorgus*)

***procerus*** (Gravenhorst, 1829, *Anomalon*) E NHM, det. Schnee, added here[[23]](#footnote-24)

***varicornis*** (Thomson, 1894, *Anomalon*)[[24]](#footnote-25) E S

NHM, det. Schnee, added here

***GRAVENHORSTIA*** Boie, 1836

*ODONTOPSIS* Förster, 1869

***picta*** Boie, 1836 E

*fasciata* (Giraud, 1857, *Anomalon*)

*fasciata* (Marshall, 1873, *Anomalon*) preocc.

*septemfasciata* (Taschenberg, 1875, *Ophion*)

*picta* (Rudow, 1882, *Anomalon*) preocc.

***HABROCAMPULUM*** Gauld, 1976

***biguttatum*** (Gravenhorst, 1829, *Anomalon*) E

***HABRONYX*** Förster, 1869

subgenus ***CAMPOSCOPUS*** Förster, 1869

*LABRORYCHUS* Förster, 1869

*BLAPTOCAMPUS* Thomson, 1892

***nigricornis*** (Wesmael, 1849, *Anomalon*) E S W I

*canaliculatus* misident.

*melanomerus* (Förster, 1860, *Agrypon*) Schnee (2008)

*maidan* (Shestakov, 1923, *Blaptocampus*)

species of *Habronyx* (*Camposcopus*) deleted from the British and Irish list:

[***perspicuus*** (Wesmael, 1849, *Anomalon*)[[25]](#footnote-26)]

subgenus ***HABRONYX*** Förster, 1869

*ACANTHOSTOMA* Kriechbaumer, 1895

*MACROSTEMMA* Shestakov, 1923

***heros*** (Wesmael, 1849, *Anomalon*) E

*mirabilis* (Desvignes, 1856, *Anomalon*)

*gravenhorstii* Förster, 1860

*gigas* (Kriechbaumer, 1880, *Anomalon*)

*oti* (Kriechbaumer, 1895, *Anomalon*)

***HETEROPELMA*** Wesmael, 1849

*SCHIZOLOMA* Wesmael, 1849

*SCHIZOPOMA* Förster, 1869

***amictum*** (Fabricius, 1775, *Ichneumon*) E S I M

Gauld (1976*b*)

*xanthopus* (Schrank, 1781, *Ichneumon*)

*amictor* (Thunberg, 1824, *Ichneumon*)

*xanthopor* (Thunberg, 1824, *Ichneumon*)

*capitatum* (Desvignes, 1856, *Anomalon*)

*bucephalum* (Vollenhoven, 1858, *Anomalon*)

*bucephalum* (Brauns, 1898, *Schizoloma*) preocc.

***megarthrum*** (Ratzeburg, 1848, *Anomalon*) E S I

*calcator* Wesmael, 1849

*scabridum* (Boie, 1855, *Anomalon*)

*megalarthrum* (Schulz, 1906, *Anomalon*)

*nigriscutum* (Fahringer, 1941, *Anomalon*)

***PARANIA*** Morley, 1913

***geniculata*** (Holmgren, 1857, *Anomalon*) E

species excluded from the British and Irish list

[***PERISPHINCTER*** Townes, 1961

***brevicollis*** (Wesmael, 1849, *Anomalon*) S[[26]](#footnote-27)]

***THERION*** Curtis, 1829

*THERIUM* Agassiz, 1846

*EXOCHILUM* Wesmael, 1849

***circumflexum*** (Linnaeus, 1758, *Ichneumon*) E S W I

*brevicorne* misident.[[27]](#footnote-28)

*ramidulum* (Christ, 1791, *Ichneumon*)

*unicolor* (Ratzeburg, 1844, *Anomalon*)

*callosum* (Shestakov, 1923, *Exochilum*)

*curticorne* Bauer, 1967

***TRICHOMMA*** Wesmael, 1849

*TRICHOMELLA* Szépligeti, 1910

***enecator*** (Rossi, 1790, *Ichneumon*) E M

*ruficoxis* Förster, 1860

***fulvidens*** Wesmael, 1849 E

*bituberculatum* Schmiedeknecht, 1902

***intermedium*** Krieger, 1904 E

***occisor*** Habermehl, 1909 E

Subfamily BANCHINAE Wesmael, 1845[[28]](#footnote-29)

LISSONOTINAE Förster, 1869 preocc.

Tribe ATROPHINI Seyrig, 1932

LISSONOTINI Förster, 1869 preocc.

***ALLOPLASTA*** Förster, 1869

*ASYMMICTUS* Förster, 1869

*TRYSICAMPE* Förster, 1869

***piceator*** (Thunberg, 1824, *Ichneumon*) E S I

*creditor* (Thunberg, 1824, *Ichneumon*)

*albitarsus* (Gravenhorst, 1829, *Exetastes*)

*lata* (Gravenhorst, 1829, *Exetastes*)

*murina* (Gravenhorst, 1829, *Lissonota*)

*murina* (Gravenhorst, 1829, *Tryphon*)

*albitarsoria* (Zetterstedt, 1838, *Tryphon*)

*genucincta* (Rudow, 1886, *Cryptus*)

*variipes* (Szépligeti, 1899, *Meniscus*)

***plantaria*** (Gravenhorst, 1829, *Phytodietus*) E W

***ARENETRA*** Holmgren, 1859

*LASIOPS* Holmgren, 1856 preocc.

***pilosella*** (Gravenhorst, 1829, *Tryphon*) E S

***CRYPTOPIMPLA*** Taschenberg, 1863

*APHANODON* Förster, 1869

*XENACIS* Förster, 1869

*XENOCORNIA* Schmiedeknecht, 1900

***altipes*** (Holmgren, 1860, *Lissonota*)[[29]](#footnote-30) E S added by Brock (in prep.)

***anomala*** (Holmgren, 1860, *Lissonota*) E S W added by Brock (in prep.)

***arvicola*** (Gravenhorst, 1829, *Lissonota*) E

*brachycentra* (Gravenhorst, 1829, *Lissonota*)

*kaisdii* (Kiss, 1929, *Arenetra*)

***calceolata*** (Gravenhorst, 1829, *Phytodietus*) E S

*leptogaster* (Holmgren, 1860, *Lissonota*)

***caligata*** (Gravenhorst, 1829, *Lissonota*) E I

***errabunda*** (Gravenhorst, 1829, *Phytodietus*) E I

***hertrichi*** Heinrich, 1952 E S added by Brock (in prep.)

***quadrilineata*** (Gravenhorst, 1829, *Tryphon*) E W

*blanda* (Gravenhorst, 1829, *Phytodietus*)

*hungarica* (Szépligeti, 1899, *Xenacis*)

*vaga* (Szépligeti, 1899, *Lissonota*)

***LISSONOTA*** Gravenhorst, 1829[[30]](#footnote-31)

*LAMPRONOTA* Curtis, 1832

*STILBONOTA* Stephens, 1835

*MENISCUS* Schiødte, 1839

*ASYNIDA* Gistel, 1848

*AMERSIBIA* Förster, 1869

*BATHYCETES* Förster, 1869

*BOTHYNOPHRYS* Förster, 1869

*ENSIMUS* Förster, 1869

*OPISORHYSSA* Kriechbaumer, 1890

*ANARTHRONOTA* Schmiedeknecht, 1900

*CAMPOCINETA* Schmiedeknecht, 1900

*ECHTHRODOCA* Schmiedeknecht, 1900

*PIMPLOPTERUS* Ashmead, 1900

*ADELOPIMPLA* Schulz, 1906

*LOPHANTIUM* Clément, 1925

*GIBBONOTA* Heinrich, 1937

*LOXONOTA* Aubert, 1993

***accusator*** (Fabricius, 1793, *Ichneumon*)[[31]](#footnote-32) E I

*segmentator* misident.

*rusticator* (Thunberg, 1824, *Ichneumon*) Horstmann (2001*b*)

*humeralis* (Zetterstedt, 1838, *Tryphon*) preocc.

*unicincta* Holmgren, 1860

*thomsoni* Schmiedeknecht, 1900

*nigricoxa* Strobl, 1902

*accusatrix* Schulz, 1906

*segmentellator* Aubert, 1967

***admontensis*** Strobl, 1902 E S added by Brock (in prep.)

*alpina* Strobl, 1902

*praebellator* Aubert, 1967

***anomala*** Holmgren, 1860 I

***antennalis*** Thomson, 1877 E W added by Brock (in prep.)

***argiola*** Gravenhorst, 1829 E S W I

*eximia* Habermehl, 1918

***biguttata*** Holmgren, 1860 E I

*femorata* Holmgren, 1860

*crassipes* Thomson, 1877

***buccator*** (Thunberg, 1824, *Ichneumon*)[[32]](#footnote-33) E S[[33]](#footnote-34)

Horstmann (2003*a*)

*varicoxa* Thomson, 1887

*iridipennis* Kriechbaumer, 1900

***canaliculata*** (Szépligeti, 1899, *Meniscus*) E added by Brock (in prep.)

*pimplator* misident.

*flavipes* Lucas, 1849

***carbonaria*** Holmgren, 1860 E S[[34]](#footnote-35)

*melania* Holmgren, 1860

*artemisiae* Tschek, 1871

***clypealis*** Thomson, 1877 E S I

*albobarbata* Strobl, 1902

***clypeator*** (Gravenhorst, 1820, *Ichneumon*) E S I

*cylindrator* misident.

?*coccinea* (Christ, 1791, *Ichneumon*) preocc.

*unicornis* Strobl, 1902

*nigrescens* Constantineanu, 1929

*spectabilis* Schmiedeknecht, 1935

*magna* Heinrich, 1952

***coracina*** (Gmelin, 1790, *Ichneumon*) E S I

*bellator* (Gravenhorst, 1807, *Ichneumon*) preocc.

*tricoloria* (Thunberg, 1824, *Ichneumon*)

*irrigua* Thomson, 1888

*bellatrix* Schulz, 1906

*meridionalis* Seyrig, 1928

***cruentator*** (Panzer, 1809, *Alomya*) E I

*insignita* Gravenhorst, 1829

*verberans* Gravenhorst, 1829

*cruentatrix* (Schulz, 1906, *Alomya*)

*rufifemur* Kiss, 1926

*szepligeti* Kiss, 1926 preocc.

***culiciformis*** Gravenhorst, 1829 E W added by Brock (in prep.)

*lateralis* Gravenhorst, 1829

*cruenta* Vollenhoven, 1858

*assimilis* Brischke, 1880

*sziladyi* Kiss, 1926

***deversor*** Gravenhorst, 1829 E

***digestor*** (Thunberg, 1824, *Ichneumon*) E S

*vocator* (Thunberg, 1824, *Ichneumon*)

*hians* Thomson, 1877

***distincta*** Bridgman, 1889 E

***dubia*** Holmgren, 1856 E S[[35]](#footnote-36)

*jugorum* (Strobl, 1903, *Mesoleius*)

*duplanae* (Heinrich, 1937, *Gibbonota*)

***erythrina*** Holmgren, 1860 S added by Brock (in prep.)

*pusilla* Habermehl, 1918

***fletcheri*** Bridgman, 1882 E[[36]](#footnote-37)

***folii*** Thomson, 1877 E I

*transversa* Bridgman, 1889

*areolata* (Kiss, 1924, *Clistopyga*) preocc.

***freyi*** (Hellén, 1915, *Meniscus*) E added by Brock (in prep.)

*tuberculata* (Hellén, 1915, *Meniscus*)

*sesiae* Habermehl, 1918

***frontalis*** (Desvignes, 1856, *Lampronota*) E S

*canaliculata* misident.

*sulcator* (Morley, 1908, *Meniscus*)

***fulvipes*** (Desvignes, 1856, *Lampronota*) E

*piffardi* (Morley, 1908, *Meniscus*)

***fundator*** (Thunberg, 1824, *Ichneumon*) E S I

*sulphurifera* Gravenhorst, 1829

*rimator* Thomson, 1877

*affinis* (Szépligeti, 1899, *Meniscus*) preocc.

*caudata* (Szépligeti, 1899, *Meniscus*)

*ruficoxis* Schmiedeknecht, 1900

*nigricoxis* Pfankuch, 1920 preocc., unavailable

***genator*** Aubert, 1972 S added by Brock (in prep.)

***gracilenta*** Holmgren, 1860 E S added by Brock (in prep.)

***gracilipes*** Thomson, 1877 E added by Brock (in prep.)

***halidayi*** Holmgren, 1860 E

***histrio*** (Fabricius, 1798, *Banchus*) E S I

*marginator* (Fabricius, 1804, *Bassus*) Horstmann (2001*b*)

*parallela* Gravenhorst, 1829

*dioszeghyi* (Kiss, 1924, *Syzeuctus*)

*nigrobasalis* Constantineanu & Pisica, 1960

***impressor*** Gravenhorst, 1829 E added by Brock (in prep.)

*basalis* Brischke, 1865

*signata* (Szépligeti, 1899, *Meniscus*)

*nigricoxis* Ulbricht, 1913 preocc.

*humerella* Habermehl, 1918 preocc.

***linearis*** Gravenhorst, 1829 E[[37]](#footnote-38)

*varicornis* (Schmiedeknecht, 1900, *Campocineta*)

*incerta* Habermehl, 1918

***lineata*** Gravenhorst, 1829 E W

*flavovariegatus* (Lucas, 1849, *Mesoleptus*)

Horstmann (1997)

***lineolaris*** (Gmelin, 1790, *Ichneumon*) E S I M

*catenator* (Panzer, 1804, *Ichneumon*)

*gladiator* (Thunberg, 1824, *Ichneumon*) preocc.

*mammillator* (Thunberg, 1824, *Ichneumon*)

*signator* (Thunberg, 1824, *Ichneumon*)

*excavator* (Zetterstedt, 1838, *Tryphon*)

*facialis* (Desvignes, 1862, *Ephialtes*)

***luffiator*** Aubert, 1969 E added by Brock (in prep.)

***maculata*** Brischke, 1865 E[[38]](#footnote-39)

*affinis* Brischke, 1865

***magdalenae*** Pfankuch, 1921 E S I[[39]](#footnote-40) added by Stelfox (1932)

*vernalis* Roman, 1925

***mutator*** Aubert, 1969 E Shaw (1999*a*)

***nigridens*** Thomson, 1889 E S I

***nitida*** Gravenhorst, 1829 E

*agnata* Gravenhorst, 1829

*rhenana* Ulbricht, 1916 unavailable

*lissonotoides* (Habermehl, 1917, *Meniscus*)

***obsoleta*** Bridgman, 1889 E

***palpalis*** Thomson, 1889 E I

*oudemansi* Smits van Burgst, 1912

*exareolata* (Habermehl, 1923, *Meniscus*)

*inareolata* (Kiss, 1824, *Meniscus*) preocc.

***palpator*** Aubert, 1969 E S added by Horstmann (2003*a*)

*parasitellae* Horstmann, 2003 Brock (in prep.)

*errabunda* misident. Horstmann (2003*a*)

***picticoxis*** Schmiedeknecht, 1900 E S added by Brock (in prep.)

***pimplator*** (Zetterstedt, 1838, *Tryphon*) S added by Aubert (1978)[[40]](#footnote-41)

***pleuralis*** Brischke, 1880 E added by Aubert (1978)

*strigifrons* Schmiedeknecht, 1900

***proxima*** Fonscolombe, 1854 E I

*varipes* (Desvignes, 1856, *Lampronota*)

*commixta* Holmgren, 1860

*lapponica* Holmgren, 1860

*opacula* Szépligeti, 1899

*variipes* Dalla Torre, 1901

***punctiventrator*** Aubert, 1977 E S W added by Aubert (1978)

*punctiventris* misident.

***punctiventris*** Thomson, 1877 E S added by Brock (in prep.)

?*errabunda* Holmgren 1860

***quadrinotata*** Gravenhorst, 1829 E W

*leucogona* Gravenhorst, 1829

*carinifrons* Thomson, 1877

***saturator*** (Thunberg, 1824, *Ichneumon*) E S W[[41]](#footnote-42)

*pubescens* (Zetterstedt, 1838, *Bassus*)

*vicina* Holmgren, 1860

*basalis* Thomson, 1889 preocc.

*mutanda* Schmiedeknecht, 1900

***semirufa*** (Desvignes, 1856, *Lampronota*) E S Horstmann (2004*c*)

***setosa*** (Geoffroy, 1785, *Ichneumon*) E

*enervator* (Fabricius, 1793, *Ichneumon*) preocc.

*cryptator* (Thunberg, 1824, *Ichneumon*)

*renovator* (Thunberg, 1824, *Ichneumon*)

*nigra* (Szépligeti, 1914, *Odinophora*) preocc.

***silvatica*** Habermehl, 1918 E added by Aubert (1978)

*palpator* Aubert, 1969

***stigmator*** Aubert, 1972 E S

***subaciculata*** Bridgman, 1886 E[[42]](#footnote-43)

*nitida* Bridgman, 1886 preocc.

***tenerrima*** Thomson, 1877 E S I

*variabilis* Holmgren, 1860 Brock (in prep.)

*fracta* Taschenberg, 1863

*rufomedia* Bridgman, 1886

*trochanterata* Bridgman, 1889 preocc.

*trochanteralis* Dalla Torre, 1901

*procera* Pfeffer, 1913

*bimaculata* Constantineanu & Ciochia, 1968 preocc.

***trochanterator*** Aubert, 1972 E

***versicolor*** Holmgren, 1860 E I

*formosa* Bridgman, 1888

*coxata* Smits van Burgst, 1914

*rufithorax* Habermehl, 1918

**sp. A** E added by Brock (in prep.)

**sp. C** E added by Brock (in prep.)

**sp. D** E S W added by Brock (in prep.)

**sp. P** S added by Brock (in prep.)

*impressor* misident.

**sp. S** E added by Brock (in prep.)

**sp. SI** E added by Brock (in prep.)

**sp. V** E added by Brock (in prep.)

species excluded from the British and Irish list

[***bilineata*** Gravenhorst, 1829[[43]](#footnote-44)]

[***funebris*** Habermehl, 1923[[44]](#footnote-45)]

[***impressor*** Gravenhorst, 1829[[45]](#footnote-46) E I Aubert (1978)

*basalis* Brischke, 1865]

***SYZEUCTUS*** Förster, 1869

*DICERATOPS* Förster, 1869

*SYZEUCTA* Thomson, 1889

***bicornis*** (Gravenhorst, 1829, *Lissonta*) E

***fuscator*** (Panzer, 1809, *Ophion*) E W

*maculatorius* (Fabricius, 1787, *Ichneumon*) preocc.

*bicolor* Szépligeti, 1899

*rufipes* Kiss, 1933

species excluded from the British and Irish list

[***irrisorius*** (Rossius, 1794, *Ichneumon*)[[46]](#footnote-47)]

Tribe BANCHINI Wesmael, 1845

***BANCHUS*** Fabricius, 1798[[47]](#footnote-48)

***crefeldensis*** Ulbricht, 1916 S I

*croaticus* Hensch, 1928

***dilatatorius*** (Thunberg, 1824, *Ichneumon*) E S

*variegator* misident.

*acuminator* (Fabricius, 1787, *Ichneumon*)

*compressus* (Fabricius, 1787, *Ichneumon*)

*sibiricus* Meyer, 1927

***falcatorius*** (Fabricius, 1775, *Ichneumon*) E

*variegator* (Fabricius, 1775, *Ichneumon*)

*tricolor* (Schrank, 1776, *Ichneumon*)

*intersectus* (Geoffroy, 1785, *Ichneumon*)

*aries* (Christ, 1791, *Ichneumon*)

*notatorius* (Olivier, 1792, *Ichneumon*) preocc.

*histrio* (Schrank, 1802, *Ichneumon*) preocc.

*labiatus* (Schrank, 1802, *Ichneumon*)

*falcator* Fabricius, 1804

*luteofasciatus* Ulbricht, 1911 unavailable

*nobilitator* Morley, 1915

*sanguinator* Meyer, 1922

*lavrovi* Meyer, 1927

*nigromarginatus* Constantineanu & Pisica, 1960

*propitius* Kuslitzky, 1979

***hastator*** (Fabricius, 1793, *Ichneumon*) E S I

*pungitor* (Thunberg, 1824, *Ichneumon*)

*reticulator* (Thunberg, 1824, *Ichneumon*)

*femoralis* Thomson, 1897

*kolosovi* Meyer, 1925

***moppiti*** Fitton, 1985[[48]](#footnote-49) added by Fitton (1985)

***palpalis*** Ruthe, 1859 E S I added by Fitton (1985)

*monileatus* misident.

*groenlandicus* Aurivilius, 1890

***pictus*** Fabricius, 1798 E W

*cultratus* (Gmelin, 1790, *Ichneumon*) preocc.

*mutillatus* (Christ, 1791, *Ichneumon*) preocc.

*bipunctatus* Hensch, 1928

*zagoriensis* Hensch, 1928

***volutatorius*** (Linnaeus, 1758, *Ichneumon*) E S W I

*venator* (Linnaeus, 1758, *Ichneumon*)

*umbellatarum* (Schrank, 1786, *Ichneumon*)

*certator* (Thunberg, 1824, *Ichneumon*)

*monileatus* Gravenhorst, 1829

*farrani* Curtis, 1836

*moniliatus* Marshall, 1872

*alticola* Schmiedeknecht, 1910

*calcaratus* Szépligeti, 1910

*obscurus* Meyer, 1926

***EXETASTES*** Gravenhorst, 1829

*LEPTOBATUS* Gravenhorst, 1829

*RHIMPHALEA* Förster, 1869

*SEMNOPHRYS* Förster, 1869

*ALLEXETASTES* Kokujev, 1904

***adpressorius*** (Thunberg, 1824, *Ichneumon*) E S I

*guttatorius* Gravenhorst, 1829

*tristis* Gravenhorst, 1829

*procera* Kriechbaumer, 1894 unavailable

*guttifer* Thomson, 1897

*medianus* Szépligeti, 1898

*albopictus* Aubert, 1959

*albopictor* Aubert, 1972

***atrator*** (Forster, 1771, *Ichneumon*) E S I

*cinctipes* (Retzius, 1783, *Ichneumon*)

*junci* (Geoffroy, 1785, *Ichneumon*)

*osculatorius* (Fabricius, 1787, *Ichneumon*)

*obscurator* (Gmelin, 1790, *Ichneumon*)

*clavator* (Fabricius, 1793, *Ichneumon*) preocc.

*tarsator* (Fabricius, 1804, *Ophion*)

*sinuatorius* (Thunberg, 1824, *Ichneumon*)

***calobatus*** Gravenhorst, 1829 E

*calobates* Dalla Torre, 1901

***femorator*** Desvignes, 1856 E[[49]](#footnote-50)

***fornicator*** (Fabricius, 1781, *Ichneumon*) E I

*exapansor* (Thunberg, 1824, *Ichneumon*)

*punctulatus* Kokujev, 1905

***illusor*** Gravenhorst, 1829 E S I M

*minor* Szépligeti, 1901

*annulatus* Habermehl, 1927

?*geniculosus* Holmgren, 1860

***illyricus*** Strobl, 1904 E added by Brock (in prep.)

***laevigator*** (Villers, 1789, *Ichneumon*) E

*cothurnatus* (Gravenhorst, 1807, *Ichneumon*) preocc.

*incurvator* (Thunberg, 1824, *Ichneumon*)

*alpinus* Kriechbaumer, 1888

*puberulus* (Szépligeti, 1898)

*levigator* Dalla Torre, 1901

*similis* Kokujev, 1905

*nigriventris* Meyer, 1927

***maurus*** Desvignes, 1856 E

*facialis* Desvignes, 1856

*benoisti* Seyrig, 1926

*melanopus* Meyer, 1927

*croaticus* Hensch, 1928

***nigripes*** Gravenhorst, 1829 E S W[[50]](#footnote-51)

***tibialis*** Pfankuch, 1921 E added by Brock (in prep.)

***RYNCHOBANCHUS*** Kriechbaumer, 1894

***flavopictus*** Heinrich, 1937 E added by Fitton (1987)

Tribe GLYPTINI Cushman & Rohwer, 1920

***APOPHUA*** Morley, 1913

***bipunctoria*** (Thunberg, 1824, *Ichneumon*) E S W I M

*cubitoria* (Thunberg, 1824, *Ichneumon*)

*flavolineata* (Gravenhorst, 1829, *Glypta*)

*baltica* (Habermehl, 1926, *Glypta*)

***cicatricosa*** (Ratzeburg, 1848, *Glypta*)

*crenulata* (Thomson, 1889, *Glypta*)

***evanescens*** (Ratzeburg, 1848, *Glypta*) E S I

*albifrons* (Holmgren, 1856, *Glypta*)

***genalis*** (Möller, 1883, *Glypta*) I

*superba* (Hellén, 1915, *Glypta*)

***DIBLASTOMORPHA*** Förster, 1869[[51]](#footnote-52)

***cylindrator*** (Fabricius, 1787, *Ichneumon*) E S W I

*erythrogaster* Lucas, 1849

*bicornis* Boie, 1850

*bicornis* Desvignes, 1856 preocc.

*corniculata* Brischke, 1865

*elegans* Vollenhoven, 1873

*ephippigera* Kriechbaumer, 1895

*ruficornis* Szépligeti, 1898 preocc.

*paleanae* Kriechbaumer, 1900

*szepligetii* Dalla Torre, 1901

*cylindatrix* (Schulz, 1906, *Lissonota*)

*abundans* (Schmiedeknecht, 1934, *Diblastomorpha*)

***rostrata*** Holmgren, 1860[[52]](#footnote-53) E I M

***GLYPTA*** Gravenhorst, 1829

*CONOBLASTA* Förster, 1869

*FOVEOGLYPTA* Hellén, 1915

***bifoveolata*** Gravenhorst, 1829 E S W I

*setosa* Roman, 1909

***ceratites*** Gravenhorst, 1829 E S I M

***consimilis*** Holmgren, 1860 E S I

*brevicornis* Rudow, 1883

*parvicornuta* Bridgman, 1886

*xanthognatha* Thomson, 1889

*berolinae* (Strand, 1918, *Conoblasta*)

***elongata*** Holmgren, 1860 E S W I

***extincta*** Ratzeburg, 1852 E S

*nigriventris* Thomson, 1889

***femorator*** Desvignes, 1856 E S I

*filicornis* Thomson, 1889

*femoratrix* Schulz, 1906

*elegantula* Hellén, 1915

*obscurata* Kiss, 1929

*pellucida* Schmiedeknecht, 1935

*triangularis* Schmiedeknecht, 1935

*curvicoxa* Kuslitzky, 1977

***fronticornis*** Gravenhorst, 1829 E S I

*dispar* Schiødte, 1839 Horstmann (2004*b*)

***haesitator*** Gravenhorst, 1829 E S I

*haesitatrix* Schulz, 1906

*australis* (Hedwig, 1959, *Lycorina*)

***incisa*** Gravenhorst, 1829 E W

***lapponica*** Holmgren, 1860 E S I

*annulata* Bridgman, 1890

*areolaris* Hellén, 1915

*nigricoxa* (Kokujev, 1927, *Conoblasta*)

*alpina* (Heinrich, 1949, *Conoblasta*)

***lineata*** Desvignes, 1856 E

***longicauda*** Hartig, 1838 I

*nigrotrochanterata* Strobl, 1902

***longispinis*** (Gmelin, 1790, *Ichneumon*) E

*provincialis* Fonscolombe, 1854

*rubicunda* Bridgman, 1890

*algerica* Habemehl, 1917

*zangezurica* Kuslitzky, 1974

***mensurator*** (Fabricius, 1775, *Ichneumon*) E S W I

*lugubrina* Holmgren, 1860

*macropyga* Hellén, 1915

*heydeni* Habemehl, 1917

*jaroslavensis* Shestakov, 1927

***microcera*** Thomson, 1899 E added by Aubert (1978)

*segrex* Kokujev, 1913

***monoceros*** Gravenhorst, 1829 E S W I

***nigricornis*** Thomson, 1899 E S I added by Aubert (1978)

*rufipes* Brischke, 1865 preocc.

*brischkei* Dalla Torre, 1901 preocc.

*papyri* Speiser, 1908

***nigrina*** Desvignes, 1856 E S I

*flavipes* Desvignes, 1856

*ruficeps* Desvignes, 1856

*fractigena* Thomson, 1889

*obscura* Pfankuch, 1924 unavailable

*clypeodentata* Bauer, 1959

*habermani* Ozols, 1959

***nigrotrochanterator*** Strobl, 1902 E S I added by Brock (in prep.)

*mensurator* misident.

*longicauda* misident.

***parvicaudata*** Bridgman, 1889 E S I

***pedata*** Desvignes, 1856 E

***pictipes*** Taschenberg, 1863 E S I added by Aubert (1978)

***punctifrons*** Bridgman, 1890[[53]](#footnote-54) S

***resinanae*** Hartig, 1838 E

*arreptans* Hellén, 1915

*summimontis* Heinrich, 1953

***rufata*** Bridgman, 1887 E I

***sculpturata*** Gravenhorst, 1829 E I

*macrura* Habermehl, 1918

*rufoclypeata* Kiss, 1924

***scutellaris*** Thomson, 1899 E S added by Brock (in prep.)

***similis*** Bridgman, 1886 E I

*rufipes* Thomson, 1889 preocc.

*thomsonii* Dalla Torre, 1901

*thomsoni* Strobl, 1902 preocc.

***tenuicornis*** Thomson, 1889 E

*pygmaea* Shestakov, 1927

***trochanterata*** Bridgman, 1886 E S I

***ulbrichti*** Habermehl, 1926 E added by Brock (in prep.)

***vulnerator*** Gravenhorst, 1829 E S I

*vulneratrix* Schulz, 1906

*monstrosa* Hellén, 1915

***woerzi*** (Hedwig, 1952, *Conoblasta*) E added by Brock (in prep.)

**sp. PA** E added by Brock (in prep.)

**sp. PU** E I added by Brock (in prep.)

*scalaris* misident.

Species excluded from the British and Irish list

[***schneideri*** Krieger, 1897[[54]](#footnote-55)]

[***scalaris*** Gravenhorst, 1829[[55]](#footnote-56)]

[***teres*** Gravenhorst, 1829[[56]](#footnote-57)]

***TELEUTAEA*** Förster, 1869

*HOPLITOPHRYS* Förster, 1869

*TELEUTEA* Thomson, 1889

***brischkei*** (Holmgren, 1860, *Glypta*) E added by Aubert (1978); NMS; Horniman

Subfamily CAMPOPLEGINAE Förster, 1869[[57]](#footnote-58)

***ALCIMA*** Förster, 1869

***orbitale*** (Gravenhorst, 1829, *Campoplex*)[[58]](#footnote-59) E W

*alboscutellare* (Thomson, 1887, *Casinaria*)

*carinata* (Kriechbaumer, 1898, *Casinaria*) Horstmann (2006*b*)

***BATHYPLECTES*** Förster, 1869

*CANIDIA* Holmgren, 1860 preocc.

*RHEXINEURA* Förster, 1869

*BIOLYSIA* Schmiedeknecht, 1907

*BATHYPIESTA* Aubert, 1979

***anura*** (Thomson, 1887, *Canidia*) E I

***balteatus*** (Thomson, 1887, *Canidia*) E NMS, det. Horstmann, added here

*trisculptus* (Habermehl, 1926, *Canidia*)

***curculionis*** (Thomson, 1887, *Canidia*) E I added by Horstmann (1974)[[59]](#footnote-60)

*carthaginiensis* (Smits van Burgst, 1913, *Canidia*)

Horstmann (2009*d*)

***exiguus*** (Gravenhorst, 1829, *Campoplex*) E S I

*subcinctus* (Gravenhorst, 1829, *Campoplex*)

***immolator*** (Gravenhorst, 1829, *Campoplex*) I

*marginellus* (Thomson, 1887, *Nepiesta*)

***infernalis*** (Gravenhorst, 1820, *Ichneumon*) E I

*infernalis* (Gravenhorst, 1829, *Mesoleptus*) preocc.

*tristis* (Gravenhorst, 1829, *Campoplex*)

*trochantellus* (Thomson, 1887, *Canidia*)

***quinqueangularis*** (Ratzeburg, 1852, *Campoplex*) I

added by Horstmann (1974)[[60]](#footnote-61)

***rostratus*** (Thomson, 1887, *Canidia*) S I

***rufipes*** Horstmann, 1974 E NMS, det. Horstmann, added here

***tibiator*** (Gravenhorst, 1820, *Ichneumon*) E NHM, added here

*corvinus* (Thomson, 1887, *Canidia*)

***CALLIDORA*** Förster, 1869

*PANTROPA* Förster, 1869

*NEOCALLIDORA* Ozols, 1966

***analis*** (Gravenhorst, 1829, *Campoplex*) E NMS, det. Horstmann, added here

***CAMPOLETIS*** Förster, 1869

*ANILASTUS* Förster, 1869

*ECPHORA* Förster, 1869 preocc.

*ANILASTA* Thomson, 1877

*ECPHOROPSIS* Ashmead, 1900

***agilis*** (Holmgren, 1860, *Sagaritis*) I

***annulata*** (Gravenhorst, 1829, *Campoplex*) E S I

*maculipes* (Tschek, 1871, *Sagaritis*)

*trochanterata* (Kriechbaumer, 1894, *Sagaritis*)

*nigripes* (Seyrig, 1928, *Sagaritis*) preocc.

***cognata*** (Tschek, 1871, *Sagaritis*)

***crassicornis*** (Tschek, 1871, *Sagaritis*) E S I

*brachycera* (Thomson, 1877, *Sagaritis*)

***dilatator*** (Thunberg, 1824, *Ichneumon*) I

*mediator* (Zetterstedt, 1838, *Porizon*)

***ensator*** (Gravenhorst, 1829, *Campoplex*) E S I

*holmgreni* (Tschek, 1871, *Sagaritis*) Horstmann (2000*d*)

***fasciata*** (Bridgman, 1888, *Sagaritis*) E

***femoralis*** (Gravenhorst, 1829, *Campoplex*) I

*laticollis* (Holmgren, 1860, *Sagaritis*)

***fuscipes*** (Holmgren, 1856, *Campoplex*) E I

*semirufa* (Szépligeti, 1916, *Omorgus*)

***incisa*** (Bridgman, 1883, *Sagaritis*) I

***latrator*** (Gravenhorst, 1829, *Campoplex*)[[61]](#footnote-62) E S I M

*assimilis* (Gravenhorst, 1829, *Campoplex*) Horstmann (2000*d*)

*mitis* (Holmgren, 1860, *Sagaritis*)

*latratrix* (Schulz, 1906, *Sagaritis*)

*bicingulata* (Szépligeti, 1916, *Omorgus*)

***postica*** (Bridgman & Fitch, 1885, *Sagaritis*) E S I

*postica* (Bridgman, 1886, *Sagaritis*) preocc.

***punctata*** (Bridgman, 1886, *Sagaritis*) E S I

***rapax*** (Gravenhorst, 1829, *Campoplex*)[[62]](#footnote-63) E S W I

*erythropus* (Thomson, 1887, *Sagaritis*) Horstmann (2000*d*)

*curticaudis* (Szépligeti, 1916, *Omorgus*)

***raptor*** (Zetterstedt, 1838, *Porizon*)

*raptrix* (Schulz, 1906, *Sagaritis*)

*dubiosa* (Szépligeti, 1916, *Omorgus*)

*rufator* Aubert, 1960

***thomsoni*** (Roman, 1915, *Sagaritis*) E S NMS, det. Horstmann, added here

***trichoptili*** (Bauer, 1936, *Sagaritis*) E NMS, det. Horstmann, added here

***varians*** (Thomson, 1887, *Sagaritis*) E S W I added by Johnson (1929)[[63]](#footnote-64)

*completa* (Szépligeti, 1916, *Omorgus*)

***viennensis*** (Gravenhorst, 1829, *Campoplex*) E I

*annulator* (Zetterstedt, 1838, *Porizon*)

*vexans* (Holmgren, 1860, *Limneria*)

*maculipes* (Strobl, 1904, *Anilasta*)

*subdentata* (Hellén, 1949, *Sagaritopsis*)

***vimmeri*** (Gregor, 1935, *Sagaritis*) S NMS, det. Riedel, added here

***zonata*** (Gravenhorst, 1829, *Campoplex*) E S I

***CAMPOPLEX*** Gravenhorst, 1829[[64]](#footnote-65)

*DIORATICA* Förster, 1869

*OMORGUS* Förster, 1869

*OMORGA* Förster, 1869

***abbreviatus*** (Brischke, 1880, *Limneria*)

***alticolellae*** Horstmann, 1980 E S W NMS, det. Horstmann, added here

***bilobus*** (Thomson, 1887, *Omorga*) E

***brevicornis*** (Szépligeti, 1916, *Omorgus*) E NMS, det. Horstmann, added here

*flavocinctus* (Seyrig, 1928, *Sagaritis*)

***caloptiliae*** Horstmann, 2013 E S added by Horstmann (2013)

***cingulatus*** (Brischke, 1880, *Limneria*)

***continuus*** (Thomson, 1887, *Omorga*) E[[65]](#footnote-66)

***coracinus*** (Thomson, 1887, *Omorga*) E

*submarginatus* (Bridgman, 1899, *Limneria*)

***crassus*** Horstmann, 1980 E NMS, det. Horstmann, added here

***cursitans*** (Holmgren, 1860, *Limneria*) E I

***deficiens*** Gravenhorst, 1829[[66]](#footnote-67) E

*algerica* (Habermehl, 1922, *Omorga*)

***difformis*** (Gmelin, 1790, *Ichneumon*) E I

*lineolatus* Ratzeburg, 1844

*mutabilis* (Holmgren, 1860, *Limneria*)

***eudoniae*** Horstmann & Yu, 1999 E S I Horstmann & Yu (1999)

*rufipes* (Bridgman, 1883, *Nemeritis*) preocc.

*ruficoxa* (Thomson, 1887, *Omorga*) preocc.

***faunus*** Gravenhorst, 1829 E I

*xanthocarpus* (Szépligeti, 1916, *Omorgus*)

***formosanae*** Horstmann, 2012 E NMS, det. Horstmann, added here

***fusciplica*** (Thomson, 1887, *Omorga*)

***hadrocerus*** (Thomson, 1887, *Omorga*) E I

*fasciatus* (Bridgman, 1889, *Limneria*)

***interruptus*** Horstmann, 1993 E S NMS, det. Horstmann, added here

***investigator*** (Habermehl, 1923, *Omorga*) E NMS, det. Horstmann, added here

***jaeckhi*** (Bauer, 1936, *Dioctes*) E added by Horstmann (2012*b*)

***lugubrinus*** (Holmgren, 1860, *Limneria*) E S I

*pusillus* (Szépligeti, 1916, *Angitia*)

***lyratus*** (Thomson, 1887, *Omorga*) E S W I M added by Shaw (1984)

***melanostictus*** Gravenhorst, 1829 E S

***molestus*** Gravenhorst, 1829 E

***multicinctus*** Gravenhorst, 1829 E I

*excentricus* (Bauer, 1937, *Omorgus*) Horstmann (2000*d*)

***ovatus*** (Brischke, 1880, *Limneria*) E S

***procerus*** (Brischke, 1880, *Limneria*)

***psammae*** (Morley, 1915, *Omorga*) E S I[[67]](#footnote-68)

***punctipleuris*** Horstmann, 1980 E S W added by Horstmann (1980)

*alhpictus* (Pfankuch, 1924, *Omorga*) unavailable

*albipictus* Horstmann 1986 unavailable

***punctulatus*** (Szépligeti, 1916, *Omorgus*) E S I

NHM, NMS, det. Horstmann, added here

***pyraustae*** Smith, 1931 E S W I M added by Shaw & Aeschlimann (1994)

*continuus* misident.[[68]](#footnote-69)

***ramidulus*** (Brischke, 1880, *Limneria*) E

***raschkiellae*** Horstmann, 1980 E S added by Horstmann (1980)

***restrictor*** Aubert, 1960 E added by Shaw (1981*a*)

***rothii*** (Holmgren, 1860, *Limneria*) E S

***rufipes*** Gravenhorst, 1829 E S I

*angulatus* (Thomson, 1887, *Omorga*) Horstmann (2000*d*)

***striatus*** Horstmann, 1985 E NHM, det. Horstmann, added here

***sulcatus*** Horstmann, 1985 E S M added by Horstmann (1985)

***tibialis*** (Szépligeti, 1916, *Nemeritis*) E W NHM, det. Horstmann, added here[[69]](#footnote-70)

*dioszeghyi* (Kiss, 1929, *Omorgus*)

*corsicator* Aubert, 1960

***tumidulus*** Gravenhorst, 1829 E S W I M[[70]](#footnote-71)

*ensator* misident.

*nigrifemur* (Seyrig, 1928, *Omorgus*)

*rufinator* Aubert, 1971 Horstmann (2000*d*)

***tussilaginis*** Horstmann, 2013 E added by Horstmann (2013)

***unicingulatus*** (Schmiedeknecht, 1909, *Omorgus*) E S M

added by Horstmann (1985)

***variabilis*** (Bridgman, 1886, *Limneria*) E

***volubilis*** (Holmgren, 1860, *Limneria*) S NMS, det. Horstmann, added here

doubtfully placed species of *Campoplex*

[***arvensis*** Gravenhorst, 1829 nom. dub. [[71]](#footnote-72)]

species excluded from the British and Irish list

[***borealis*** (Zetterstedt, 1838, *Porizon*)[[72]](#footnote-73)]

[***melanostoma*** (Strobl, 1904, *Limneria*)[[73]](#footnote-74)

*anterior* Aubert, 1960]

***CASINARIA*** Holmgren, 1859

*AMORPHOTA* Förster, 1869

*ANEMPHERES* Förster, 1869

*CAMPOTREPHUS* Förster, 1869

*HOROGENES* Förster, 1869

*NOTHANOMALON* Szépligeti, 1905

*TROPHOCAMPA* Schmiedeknecht, 1907

*CASINARIODES* Aubert, 1960

***affinis*** Tschek, 1871 I

***albipalpis*** (Gravenhorst, 1829, *Campoplex*) I

***ischnogaster*** Thomson, 1887 E I

***moesta*** (Gravenhorst, 1829, *Campoplex*) E added by Horstmann (2000*d*)

*maesta* Dalla Torre, 1901 preocc.

***morionella*** Holmgren, 1860 E

***pallipes*** Brischke, 1880 E I

*pallidipes* Dalla Torre, 1901

***petiolaris*** (Gravenhorst, 1829, *Mesoleptus*) E

*claviventris* Holmgren, 1860

***tenuiventris*** (Gravenhorst, 1829, *Campoplex*) E

*conica* (Ratzeburg, 1844, *Campoplex*)

*latifrons* Holmgren, 1860

*protensa* Thomson, 1887

***CHAROPS*** Holmgren, 1859

***cantator*** (DeGeer, 1778, *Ichneumon*) E W

*decipiens* (Gravenhorst, 1829, *Campoplex*)

*tenuitarsus* (Gravenhorst, 1829, *Anomalon*)

*nigropetiolatus* Strobl, 1904

*nigrifacies* (Kiss, 1924, *Anomalon*)

***CLYPEOPLEX*** Horstmann, 1987

***cerophagus*** (Gravenhorst, 1829, *Campoplex*) E S

NHM, NMS, UM, added here

*picticrus* (Thomson, 1887, *Omorga*)

***CYMODUSA*** Holmgren, 1859

*SAGARITIS* Holmgren, 1859 preocc.

*THERSITIA* Schmiedeknecht, 1907

*SAGARITOPSIS* Hincks, 1944

***antennator*** Holmgren, 1860 E S I

*flavipes* Brischke, 1880

*anntenatrix* Schulz, 1906

***cruentata*** (Gravenhorst, 1829, *Campoplex*) S I

*marginella* (Zetterstedt, 1838, *Porizon*)

*longicalcar* Thomson, 1887

***declinator*** (Gravenhorst, 1829, *Campoplex*)[[74]](#footnote-75) E S

*fasciata* (Bridgman & Fitch, 1885, *Thymaris*)

*fasciata* (Bridgman, 1886, *Thymaris*) preocc.

*declinatrix* (Schulz, 1906, *Sagaritis*)

***exilis*** Holmgren, 1860 E I

*petulans* Holmgren, 1860

*convergens* (Thomson, 1887, *Nemeritis*)

***leucocera*** Holmgren, 1859 E S I

*pulchricornis* Szépligeti, 1901

*egregia* (Schmiedeknecht, 1907, *Thersitia*)

***DIADEGMA*** Förster, 1869[[75]](#footnote-76)

*ANGITIA* Holmgren, 1859

*NYTHOBIA* Förster, 1869

*PECTINELLA* Morley, 1915

*NEOANGITIA* Horstmann, 1969

*AUMA* Dbar, 1984[[76]](#footnote-77)

***aculeatum*** (Bridgman, 1889, *Limneria*) E W

*atrum* (Kokujev, 1915, *Angitia*)

*politor* (Aubert, 1960, *Horogenes*)

***agile*** (Brischke, 1880, *Limneria*)

***angitiaeforma*** Horstmann, 1969 E S NMS, det. Horstmann, added here

***angulator*** (Aubert, 1963, *Horogenes*) E I NMS, det. Horstmann, added here

***annulicrus*** (Thomson, 1887, *Angitia*)

***anurum*** (Thomson, 1887, *Angitia*) E S added by Shaw & Horstmann (1997)

***areolare*** (Holmgren, 1860, *Limneria*) I added by O’Connor *et al.* (2007)[[77]](#footnote-78)

***argentellae*** Horstmann, 2004 S[[78]](#footnote-79) added by Horstmann (2004*a*)

***armillatum*** (Gravenhorst, 1829, *Campoplex*)[[79]](#footnote-80) E S W I

*tibiale* (Gravenhorst, 1829, *Campoplex*)

*pseudocombinatum* (Szépligeti, 1916, *Angitia*)

***berberatae*** Horstmann, 2013 E added by Horstmann (2013)

***brevipetiolatum*** Horstmann, 1969 E NMS, det. Horstmann, added here

***brevivalve*** (Thomson, 1887, *Angitia*) E NMS, det. Horstmann, added here

***callisto*** Horstmann, 1993 S NMS, det. Horstmann, added here

***chrysostictos*** (Gmelin, 1790, *Ichneumon*) E S I

Horstmann & Shaw (1984)

*corsicator* (Aubert, 1961, *Horogenes*)

*orientator* Aubert, 1965

***claripenne*** (Thomson, 1887, *Angitia*) I added by O’Connor *et al.* (2007)[[80]](#footnote-81)

***clavicorne*** (Brischke, 1880, *Limneria*)

***coleophorarum*** (Ratzeburg, 1852, *Campoplex*) E S

***combinatum*** (Holmgren, 1860, *Limneria*) E S I

*alpinator* Aubert, 1970

***compunctellae*** Horstmann, 2013 S added by Horstmann (2013)

***consumtor*** (Gravenhorst, 1829, *Campoplex*)

*varians* (Brischke, 1880, *Limneria*)

***crassicorne*** (Gravenhorst, 1829, *Campoplex*)[[81]](#footnote-82) E S W I

Horstmann (2000*d*)

*carnifex* (Gravenhorst, 1829, *Campoplex*)

*brevicorne* (Holmgren, 1860, *Limneria*)

*normannicum* (Rudow, 1883, *Limneria*)

***crassiseta*** (Thomson, 1887, *Angitia*) E NHM, det. Perkins, added here

***crassum*** (Bridgman, 1889, *Limneria*) E S

***crataegi*** Horstmann, 1980 E S added by Horstmann (1980)

***cylindricum*** (Brischke, 1880, *Limneria*) E I[[82]](#footnote-83)

***duplicatum*** Horstmann, 1980 E S added by Shaw & Horstmann (1997)

***elishae*** (Bridgman, 1884, *Limneria*) E S I

***ericinellae*** Horstmann, 2013 E added by Horstmann (2013)

***erucator*** (Zetterstedt, 1838, *Porizon*) E S I

*rufipes* misident.

*fumipennis* (Holmgren, 1856, *Campoplex*)

***exareolator*** Aubert, 1964 E S I added by Shaw & Horstmann (1997)

***fabricianae*** Horstmann & Shaw, 1984 E S W added by Horstmann & Shaw (1984)

***fenestrale*** (Holmgren, 1860, *Limneria*) E S W I M

Azidah *et al*. (2000); NMS

?*gracile* (Gravenhorst, 1829, *Campoplex*)[[83]](#footnote-84)

***flexum*** Horstmann, 1973 E NMS, det. Horstmann, added here

***fungicola*** Horstmann, 2008 E added by Horstmann (2008*a*)

***grisescens*** (Gravenhorst, 1829, *Mesoleptus*) E S I

*rufiventris* (Gravenhorst, 1829, *Campoplex*)

*hydropota* (Holmgren, 1860, *Limneria*)

***holopygum*** (Thomson, 1887, *Angitia*) E S I

***hygrobium*** (Thomson, 1887, *Meloboris*) E S W I

*ischnocerum* (Thomson, 1887, *Meloboris*)

*pechlaneri* (Hedwig, 1957, *Angitia*) unavailable

***incompletum*** Horstmann, 1973 E S NMS, det. Horstmann, added here

***insectator*** (Schrank, 1781, *Ichneumon*) E I

*insectatrix* (Schulz, 1906, *Angitia*)

**?*kyffhusanae*** Horstmann, 1973 E[[84]](#footnote-85)

***laricinellum*** (Strobl, 1904, *Angitia*) E S added by Shaw & Horstmann (1997)

**?*laterale*** (Gravenhorst, 1829, *Campoplex*) E I[[85]](#footnote-86)

***latungulum*** (Thomson, 1887, *Angitia*) E S W I

*deletum* (Morley, 1915, *Pectinella*)

***ledicola*** Horstmann, 1969 E W added by Shaw & Horstmann (1997)

***lithocolletis*** Horstmann, 1969 E S added by Shaw & Horstmann (1997)

***litorale*** (Holmgren, 1856, *Campoplex*) E I

***majale*** (Gravenhorst, 1829, *Campoplex*) E S I

***melanium*** (Thomson, 1887, *Angitia*) E

***monospilum*** (Thomson, 1887, *Angitia*) E S W

NMS, det. Horstmann, added here

***nanus*** (Gravenhorst, 1829, *Campoplex*) E S

***naryciae*** Horstmann, 2008[[86]](#footnote-87) E S NMS, det. Horstmann, added here

*narcyiae* misspelling

***neocerophagum*** Horstmann, 1969

*cerophaga* misident.

***neomajale*** Horstmann, 1969 E NHM, det. Horstmann, added here

***pusio*** (Holmgren, 1860, *Meloboris*) E S

*annulipes* (Bridgman, 1889, *Limneria*)

***rufatum*** (Bridgman, 1884, *Limneria*) E S

***ruficeps*** (Holmgren, 1860, *Limneria*) E S NHM, NMS, det. Horstmann, added here[[87]](#footnote-88)

*gracile* (Ratzeburg, 1848, *Campoplex*) preocc.

*rimator* (Thomson, 1887, *Angitia*)

*rimatrix* (Schulz, 1906, *Angitia*)

***scotiae*** (Bridgman, 1889, *Limneria*) S

***semiclausum*** (Hellén, 1949, *Limneria*) E S I M

Azidah *et al*. (2000)

*tibialis* misident.

*cerophaga* misident.

*eucerophagum* Horstmann, 1969

***sordipes*** (Thomson, 1887, *Angitia*) E S W

***stagnale*** (Holmgren, 1856, *Campoplex*) E I

***stigmatellae*** Horstmann, 1980 E S added by Horstmann (1980)

***tenuipes*** (Thomson, 1887, *Angitia*) E S I

***tripunctatum*** (Bridgman, 1886, *Limneria*) E I

***trochanteratum*** (Thomson, 1887, *Angitia*) E I

***truncatum*** (Thomson, 1887, *Campoplex*) E W I

*subbuccatum* (Thomson, 1887, *Angitia*)

***DOLOPHRON*** Förster, 1869

***pedellum*** (Holmgren, 1860, *Limneria*) E S M

*albicoxis* (Schmiedeknecht, 1909, *Synetaeris*)

***DUSONA*** Cameron, 1901[[88]](#footnote-89)

*DELOPIA* Cameron, 1903

***admontina*** (Speiser, 1908, *Campoplex*) E S added by Horstmann (2011*b*)

*rufiventris* (Strobl, 1904, *Campoplex*) preocc.

***aemula*** (Förster, 1868, *Campoplex*) E S W added by Horstmann (2011*b*)

*discrepans* (Förster, 1868, *Campoplex*)

*dissepta* (Förster, 1868, *Campoplex*)

*parvula* (Förster, 1868, *Campoplex*) preocc.

*filicornis* (Holmgren, 1872, *Campoplex*)

**?*alpigena*** Hinz, 1972 S[[89]](#footnote-90)

***anceps*** (Holmgren, 1860, *Campoplex*) E

*auriculata* (Förster, 1868, *Campoplex*)

*disparilis* (Förster, 1868, *Campoplex*)

*costulata* (Bridgman & Fitch, 1885, *Campoplex*)

*costulata* (Bridgman 1886, *Campoplex*)

*libauensis* (Strand, 1918, *Campoplex*) Horstmann (2009*a*)

***angustata*** (Thomson, 1887, *Campoplex*) E S

***angustifrons*** (Förster, 1868, *Campoplex*) E S W

?*obreptans* (Förster, 1868, *Campoplex*)

*zonella* (Förster, 1868, *Campoplex*)

*cornella* (Teunissen, 1947, *Campoplex*)

***annexa*** (Förster, 1868, *Campoplex*) E S I M

*limnobia* (Thomson, 1887, *Campoplex*) Hinz & Horstmann (2004)

*facialis* (Holmgren, 1872, *Campoplex*) preocc.

*americana* (Ashmead, 1890, *Casinaria*) Horstmann (2009*a*)

*mariae* (Dalla Torre, 1901, *Campoplex*)

*oyamadai* Hinz, 1994 Horstmann (2009*a*)

***aurita*** (Kriechbaumer, 1883, *Campoplex*) E added by Horstmann (2011*b*)

***aversa*** (Förster, 1868, *Campoplex*) E S

*dubiosa* (Förster, 1868, *Campoplex*)

*tschekii* (Holmgren, 1872, *Campoplex*)

*crassipes* (Thomson, 1887, *Campoplex*)

***bellipes*** (Holmgren, 1872, *Campoplex*) E added by Horstmann (2011*b*)

*vernalis* Hinz, 1957

***bicoloripes*** (Ashmead, 1906, *Campoplex*) E S

*pugillator* misident.

*foersteri* (Roman, 1942, *Campoplex*) Hinz & Horstmann (2004)

***blanda*** (Förster, 1868, *Campoplex*) E S M

*remota* (Förster, 1868, *Campoplex*)

*forsselli* (Holmgren, 1872, *Campoplex*)

*punctiventris* (Woldstedt, 1877, *Casinaria*)

***bucculenta*** (Holmgren, 1860, *Campoplex*) E S

*melampus* (Förster, 1868, *Campoplex*)

***carinifrons*** (Holmgren, 1860, *Campoplex*) E

*minax* (Förster, 1868, *Campoplex*)

*geometrae* (Rudow, 1883, *Campoplex*) Horstmann (1999*a*)

***carpathica*** (Szépligeti, 1916, *Casinaria*) E S added by Horstmann (2011*b*)

*zonella* misident.

*adriaansei* (Teunissen, 1947, *Campoplex*)

Horstmann (2009*a*)

***circumcinctus*** (Förster, 1868, *Campoplex*) E added by Horstmann (2011*b*)

*subcinctus* (Förster, 1868, *Campoplex*) preocc.

***circumspectans*** (Förster, 1868, *Campoplex*) E S

added by Horstmann (2011*b*)

*vagula* (Förster, 1868, *Campoplex*)

*subsulcata* (Holmgren, 1872, *Campoplex*)

***confusa*** (Förster, 1868, *Campoplex*) E S W M

*lacunosa* (Kriechbaumer, 1883, *Campoplex*)

*consimilis* (Schmiedeknecht, 1908, *Campoplex*)

***cultrator*** (Gravenhorst, 1829, *Campoplex*) E

***disclusa*** (Förster, 1868, *Campoplex*) E S added by Horstmann (2011*b*)

***dubitor*** Hinz, 1977 E S added by Horstmann (2011*b*)

*oxyacanthae* misident.[[90]](#footnote-91)

***erythrogaster*** (Förster, 1868, *Campoplex*) E S

*indefessa* (Förster, 1868, *Campoplex*)

***falcator*** (Fabricius, 1775, *Ichneumon*) E

***flagellator*** (Fabricius, 1793, *Ichneumon*) E added by Horstmann (2011*b*)

*debilis* (Förster, 1868, *Campoplex*) Horstmann (2009*a*)

*heterocera* (Förster, 1868, *Campoplex*) Horstmann (2001*b*)

**?*holmgrenii*** (Dalla Torre, 1901, *Campoplex*)

*unicincta* (Holmgren, 1872, *Campoplex*) preocc.

***humilis*** (Förster, 1868, *Campoplex*) E S added by Horstmann (2011*b*)

*eurynotus* (Holmgren, 1872, *Campoplex*)

***incompleta*** (Bridgman, 1889, *Campoplex*) E S

***inermis*** (Förster, 1868, *Campoplex*) E S added by Horstmann (2011*b*)

***infesta*** (Förster, 1868, *Campoplex*) S W

*terrifica* (Förster, 1868, *Campoplex*)

***insignita*** (Förster, 1868, *Campoplex*) E S

*bistrigosa* (Holmgren, 1872, *Campoplex*)

***juvenilis*** (Förster, 1868, *Campoplex*) E W

*victor* (Thunberg, 1824, *Ichneumon*) preocc.

*monozona* (Förster, 1868, *Campoplex*)

***leptogaster*** (Holmgren, 1860, *Campoplex*) E S

*macrostylus* (Förster, 1868, *Campoplex*)

*sylvicola* (Habermehl, 1922, *Campoplex*)

***libertatis*** (Teunissen, 1947, *Campoplex*) E added by Horstmann (2011*b*)

***mercator*** (Fabricius, 1793, *Ichneumon*) E S

*venditor* (Thunberg, 1824, *Ichneumon*)

*oxyacanthae* (Boie, 1855) Hinz & Horstmann (2004)

*mesoxantha* (Förster, 1868, *Campoplex*)

***minor*** (Provancher, 1879, *Campoplex*) E added by Horstmann (2011*b*)

***montana*** (Roman, 1929, *Campoplex*) E added by Horstmann (2011*b*)

*carinifer* (Teunissen, 1947, *Campoplex*) Horstmann (2009*a*)

***myrtilla*** (Desvignes, 1856, *Campoplex*) E

*tenthredinum* (Tschek, 1871, *Campoplex*)

*nobilitata* (Holmgren, 1872, *Campoplex*)

***nidulator*** (Fabricius, 1804, *Ophion*) E S

*nitidulator* (Holmgren, 1856, *Campoplex*)

*circumscripta* (Förster, 1868, *Campoplex*)

*martialis* (Förster, 1868, *Campoplex*)

*vindex* (Förster, 1868, *Campoplex*)

*bifida* (Thomson, 1887, *Campoplex*)

*obscura* (Kiss, 1926, *Campoplex*)

***notabilis*** (Förster, 1868, *Campoplex*) E S

*scolator* misident.[[91]](#footnote-92)

***obliterata*** (Holmgren, 1872, *Campoplex*) E added by Horstmann (2011*b*)

*limniventris* (Kriechbaumer, 1883, *Campoplex*)

**?*opaca*** (Thomson, 1887, *Campoplex*)

***petiolator*** (Fabricius, 1804, *Ophion*) E S

*lapponica* (Holmgren, 1860, *Campoplex*)

*callizona* (Förster, 1868, *Campoplex*)

*punctata* (Bridgman & Fitch, 1885, *Campoplex*)

*punctata* (Bridgman, 1886, *Campoplex*) preocc.

*nigra* (Kiss, 1924, *Campoplex*)

***pineticola*** (Holmgren, 1872, *Campoplex*) E S added by Horstmann (2011*b*)

*litigiosa* (Habermehl, 1922, *Campoplex*)

*sibirica* Hinz, 1985 Horstmann (2009*a*)

***polita*** (Förster, 1868, *Campoplex*) E S

*flavipalpis* (Förster, 1868, *Campoplex*)

*mediana* (Förster, 1868, *Campoplex*)

*spoliator* (Förster, 1868, *Campoplex*)

*trisculpta* (Holmgren, 1872, *Campoplex*)

*femorator* (Bridgman & Fitch, 1885, *Campoplex*)

*femorator* (Bridgman, 1886, *Campoplex*) preocc.

*latungula* (Thomson, 1887, *Campoplex*)

*splendens* (Thomson, 1887, *Campoplex*)

***prominula*** (Förster, 1868, *Campoplex*) E S

*contumax* (Förster, 1868, *Campoplex*)

*foveolata* (Förster, 1868, *Campoplex*)

***pugillator*** (Linnaeus, 1758, *Ichneumon*) E S

*canaliculata* (Förster, 1868, *Campoplex*) preocc.

***pulchripes*** (Holmgren, 1872, *Campoplex*) E I added by Horstmann (2011*b*)

*praecox* (Teunissen, 1947, *Campoplex*) Horstmann (2009*a*)

***recta*** (Thomson, 1887, *Campoplex*) E added by Horstmann (2011*b*)

***rubidatae*** Horstmann, 2009 S added by Horstmann (2011*b*)

***rugifer*** (Förster, 1868, *Campoplex*) E[[92]](#footnote-93) S

*subaequalis* (Förster, 1868, *Campoplex*) Horstmann (2009*a*)

*puncta* (Kriechbaumer, 1883, *Campoplex*)

**?*rugulosa*** (Förster, 1868, *Campoplex*)

***semiflava*** (Costa, 1883, *Campoplex*) E added by Horstmann (2011*b*)

*flaviscapus* (Thomson, 1887, *Campoplex*)

***sobolicida*** (Förster, 1868, *Campoplex*) E S W

*ulcerata* (Holmgren, 1872, *Campoplex*)

***spinipes*** (Thomson, 1887, *Campoplex*) E added by Horstmann (2011*b*)

***stenogaster*** (Förster, 1868, *Campoplex*) E added by Horstmann (2011*b*)

*monticola* (Habermehl, 1922, *Campoplex*)

***stragifex*** (Förster, 1868, *Campoplex*) E S M

*delusor* misident.

*adjuncta* (Förster, 1868, *Campoplex*)

*areolata* (Brauns, 1895, *Campoplex*)

***stygia*** (Förster, 1868, *Campoplex*) E added by Horstmann (2011*b*)

***subimpressa*** (Förster, 1868, *Campoplex*) E added by Horstmann (2011*b*)

*transitoria* (Kiss, 1924, *Campoplex*)

***tenuis*** (Förster, 1868, *Campoplex*) E S

*agnata* (Förster, 1868, *Campoplex*)

*anxia* (Förster, 1868, *Campoplex*)

*peraffinis* (Förster, 1868, *Campoplex*)

*proxima* (Förster, 1868, *Campoplex*)

***terebrator*** (Förster, 1868, *Campoplex*) E S W I M

added by Horstmann (2011*b*)

***thomsoni*** Hinz, 1966 E added by Horstmann (2011*b*)

***vigilator*** (Förster, 1868, *Campoplex*) E S

***xenocampta*** (Förster, 1868, *Campoplex*) S

*polyxantha* (Strobl, 1904, *Campoplex*)

*baltica* (Habermehl, 1926, *Campoplex*)

Species excluded from the British and Irish list

[***limnobia*** (Thomson, 1887, *Campoplex*)[[93]](#footnote-94)]

[***vidua*** (Gravenhorst, 1829, *Campoplex*)[[94]](#footnote-95)]

***ECHTHRONOMAS*** Förster, 1869

***facialis*** (Thomson, 1887, *Anilasta*) E Horniman, det. Shaw, added here

***ochrostoma*** (Holmgren, 1860, *Casinaria*) NHM, added here

***tricincta*** (Gravenhorst, 1829, *Campoplex*)

***ENYTUS*** Cameron, 1905

*IOCTES* Förster, 1869

*NAREOLATA* Ellinger & Sachtleben, 1928

***apostata*** (Gravenhorst, 1829, *Campoplex*) E S I Shaw (1981*a*)

*exareolata* (Ratzeburg, 1852, *Campoplex*)

*reticulata* (Bridgman, 1884, *Limneria*)

***appositor*** (Aubert, 1970, *Diadegma*) E S W M

NMS, NHM, det. Horstmann, added here

***crataegellae*** (Thomson, 1887, *Angitia*) E NMS, det. Horstmann, added here

***neoapostata*** (Horstmann, 1969, *Diadegma*) E S I

*neapostatus* misspelling

***parvicanda*** (Thomson, 1887, *Angitia*)

*parvicauda* misspelling

***styriacus*** (Horstmann, 1980, *Diadegma*) S NMS, det. Horstmann, added here

***ERIBORUS*** Förster, 1869[[95]](#footnote-96)

*ZAPORUS* Förster, 1869

***braccatus*** (Gmelin, 1790, *Ichneumon*) E

*jocator* (Fabricius, 1793, *Ichneumon*)

*nigriventris* (Habermehl, 1922, *Anilasta*)

***dorsalis*** (Gravenhorst, 1829, *Campoplex*)[[96]](#footnote-97) E S

*micorocephalus* (Gravenhorst, 1829, *Ischnoceros*)

***perfidus*** (Gravenhorst, 1829, *Campoplex*) E

*aberrans* (Gravenhorst, 1829, *Campoplex*

*obscuriventris* Kiss, 1926

***GONOTYPUS*** Förster, 1869

*GONOTYPA* Thomson, 1887

***melanostoma*** (Thomson, 1887, *Gonotypa*) E W I M

***HYPOSOTER*** Förster, 1869

*AMELOCTONUS* Förster, 1869

*ISCHNOSCOPUS* Förster, 1869

*RHYTHMONOTUS* Förster, 1869

***albonotatus*** (Bridgman, 1889, *Limneria*) E S

*melaleucus* (Schmiedeknecht, 1909, *Anilastus*)

***alienus*** (Brischke, 1880, *Limneria*) I

***barrettii*** (Bridgman, 1881, *Limneria*) E

*teucrii* (Bridgman, 1889, *Limneria*)

***boops*** (Thomson, 1887, *Anilasta*)[[97]](#footnote-98) E added by Horstmann (2013)

***brischkei*** (Bridgman, 1882, *Limneria*) E S I

***caedator*** (Gravenhorst, 1829, *Campoplex*) E

*henscheli* (Smits van Burgst, 1910, *Anilastus*)

*persimilis* (Szépligeti, 1916, *Anilastus*)

*parvulus* (Kiss, 1926, *Anilastus*)

***carbonarius*** (Ratzeburg, 1844, *Campoplex*) E S

***clausus*** (Brischke, 1880, *Limneria*)[[98]](#footnote-99) E S

***coxator*** (Thomson, 1887, *Anilasta*) E NHM, NMS, det. Horstmann, added here

***didymator*** (Thunberg, 1824, *Ichneumon*) E S W I

*rotundator* (Thunberg, 1824, *Ichneumon*)

*ruficinctus* (Gravenhorst, 1829, *Campoplex*)

*schmiedeknechti* (Smits van Burgst, 1913, *Anilastus*)

***discedens*** (Schmiedeknecht, 1909, *Anilastus*) I

***dolosus*** (Gravenhorst, 1829, *Campoplex*) E S

*rufimanus* (Gravenhorst, 1829, *Campoplex*)

Horstmann (2000*d*)

*oculatus* (Tschek, 1871, *Limneria*)

***fitchii*** (Bridgman, 1881, *Limneria*)

***inquinatus*** (Holmgren, 1860, *Limneria*)[[99]](#footnote-100) S

***leucomerus*** (Thomson, 1887, *Anilasta*) E I NMS, Horstmann, added here

*tricinctus* (Holmgren, 1858, *Limneria*) unavailable

***longulus*** (Thomson, 1887, *Anilasta*) E S W

***neglectus*** (Holmgren, 1860, *Limneria*) S NMS, det. Horstmann, added here

*varicoxa* (Thomson, 1887, *Anilasta*)

*variicoxus* (Dalla Torre, 1901, *Anilastus*)

***notatus*** (Gravenhorst, 1829, *Campoplex*) E S W I

***obscurellus*** (Holmgren, 1860, *Limneria*)

***orbator*** (Gravenhorst, 1829, *Campoplex*)

*rufus* (Bridgman, 1882, *Limneria*)

***placidus*** (Desvignes, 1856, *Campoplex*) E

***rhodocerae*** (Rondani, 1877, *Tryphon*) E I

*ebeninus* misident.

***ruficrus*** (Thomson, 1887, *Anilasta*) E NMS, det. Horstmann, added here

***thuringiacus*** (Schmiedeknecht, 1909, *Anilastus*) E I

***tricolor*** (Ratzeburg, 1844, *Campoplex*) E S

*vidua* misident.

*henaultii* (Desvignes, 1856, *Campoplex*)

***virginalis*** (Gravenhorst, 1829, *Campoplex*) E Horstmann (2000*d*)

***vividus*** (Holmgren, 1860, *Limneria*) E NHM, added here

*albicrus* (Thomson, 1877, *Anilasta*)

Species of *Hyposoter* excluded from the British and Irish list:

[***anglicanus*** (Habermehl, 1923, *Anilasta*)[[100]](#footnote-101)]

***Lathroplex*** Förster, 1869

***clypearis*** Thomson, 1887 E NMS, det. Horstmann, added here

***LATHROSTIZUS*** Förster, 1869

*LATHROSTIZA* Thomson, 1887

***clypeatus*** (Brischke, 1880, *Limneria*) S

*sternocerus* (Thomson, 1887, *Lathrostiza*) Horstmann (2004*a*)

***lugens*** (Gravenhorst, 1829, *Campoplex*) E I M

*vestigialis* (Ratzeburg, 1852, *Campoplex*)

***LEMOPHAGUS*** Townes, 1965

*HOLOCREMNODES* Aubert, 1986 Horstmann (2004*a*)

***curtus*** Townes, 1965 E W added by Cox (2007); NHM; NMS

***errabundus*** (Gravenhorst, 1829, *Campoplex*) E

added by Salisbury (2003)

***LEPTOCAMPOPLEX*** Horstmann, 1970

***cremastoides*** (Holmgren, 1860, *Nemeritis*)[[101]](#footnote-102) E S

NMS, NHM, added here

***MACRUS*** Gravenhorst, 1829

***parvulus*** (Gravenhorst, 1829, *Campoplex*) E S

*fusicornis* (Roman, 1923, *Lathroplex*)

***MELANOPLEX*** Horstmann, 1987

***bucculentus*** (Holmgren, 1860, *Limneria*) E NHM, det. Horstmann, added here

***MELOBORIS*** Holmgren, 1859

*ASINAMORA* Förster, 1869

*NEPIERA* Förster, 1869

*PSEUDOCYMODUSA* Habermehl, 1922

*ANOIXIS* Townes, 1970

***alternans*** (Gravenhorst, 1829, *Campoplex*) E S I

*elachistae* (Brischke, 1880, *Cymodusa*)

*ruficornis* (Bridgman, 1884, *Limneria*) preocc.

***cingulata*** Horstmann, 2004 S added by Horstmann (2004*a*)

***collector*** (Thunberg, 1824, *Ichneumon*) E S W I M

*concinna* (Holmgren, 1860, *Limneria*)

*signata* (Szépligeti, 1916, *Omorgus*)

*foersteri* (Kiss, 1924, *Idechthis*)

*albicincta* (Seyrig, 1927, *Angitia*)

***gracilis*** Holmgren, 1859 E S I

*monticolana* (Bridgman, 1881, *Limneria*)

***neglecta*** (Habermehl, 1923, *Pseudocymodusa*)

***proxima*** (Perkins, 1942, *Nepiera*) E S I NHM, NMS, det. Horstmann, added here

***NEMERITIS*** Holmgren, 1860[[102]](#footnote-103)

*PSEUDONEMERITIS* Szépligeti, 1916

***breviventris*** Horstmann, 1975 E NHM, det. Horstmann, added here

***caudatula*** Thomson, 1887 E

*rhaphidiae* Kriechbaumer, 1892

*raphidiae* Dalla Torre, 1901

*monticola* Habermehl, 1922

***cingulata*** Horstmann, 1980 E

***fallax*** (Gravenhorst, 1829, *Campoplex*) E NMS, NHM, det. Horstmann, added here

*crassiceps* Habermehl, 1922

***lativentris*** Thomson, 1887 E

***macrocentra*** (Gravenhorst, 1829, *Campoplex*) E I

*sordida* (Gravenhorst, 1829, *Campoplex*)

*varipes* (Gravenhorst, 1829, *Campoplex*)

*antennalis* (Szépligeti, 1916, *Angitia*)

*caudata* (Szépligeti, 1916, *Omorgus*) preocc.

*transsylvanica* (Szépligeti, 1916, *Canidia*)

*caudata* (Gregor, 1940, *Idechthis*) preocc.

***silvicola*** Horstmann, 1973 E

***stenura*** Thomson, 1887[[103]](#footnote-104)

***NEPIESTA*** Förster, 1869

***mandibularis*** (Holmgren, 1860, *Limneria*) E S I

*aberrans* misident.

*umbrata* (Brischke, 1880, *Canidia*)

*nigra* Szépligeti, 1901

***subclavata*** Thomson, 1887 E NMS, det Horstmann, added here

***tricingulata*** Horstmann, 1973 E NHM, det. Horstmann, added here

***OLESICAMPE*** Förster, 1869

*LIMNERIA* Förster, 1859

*HOLOCREMNUS* Förster, 1869

*OMOBORUS* Förster, 1869

*HOLOCREMNA* Thomson, 1887

*OLESICAMPA* Thomson, 1887

***alboplica*** (Thomson, 1887, *Olesicampa*) E I

*simplex* (Thomson, 1887, *Olesicampa*)

***argentata*** (Gravenhorst, 1829, *Campoplex*) S

***auctor*** (Gravenhorst, 1829, *Campoplex*) E

*limbata* (Gravenhorst, 1829, *Campoplex*)

*auctrix* (Schulz, 1906, *Olesicampa*)

***binotata*** (Thomson, 1887, *Olesicampa*) E S NHM, det. Perkins, added here

***buccata*** (Thomson, 1887, *Holocremna*) I

***canaliculata*** (Gravenhorst, 1829, *Campoplex*) M

NMS, det. Horstmann, added here

***cavigena*** (Thomson, 1887, *Olesicampa*)

***clandestina*** (Holmgren, 1860, *Limneria*) E I Shaw (1999*a*)

***crassitarsis*** (Thomson, 1887, *Olesicampa*) S I

***erythropyga*** (Holmgren, 1860, *Limneria*) I

***femorella*** (Thomson, 1887, *Olesicampa*) E NHM, det. Perkins, added here

***forticostata*** (Schmiedeknecht, 1909, *Anilastus*) E

***fulcrans*** (Thomson, 1887, *Olesicampa*) E S I

***fulviventris*** (Gmelin, 1790, *Ichneumon*) E I

***geniculella*** (Thomson, 1887, *Olesicampa*) S I

***gracilipes*** (Thomson, 1887, *Olesicampa*) I

***longipes*** (Müller, 1776, *Ichneumon*) I

*canescens* (Gmelin, 1790, *Ichneumon*)

***macellator*** (Thunberg, 1824, *Ichneumon*) E S

*retecta* (Hartig, 1838, *Campoplex*)

*cothurnata* (Holmgren, 1860, *Limneria*)

*frutetorum* (Thomson, 1887, *Holocremna*)

[***monticola*** (Hedwig, 1938, *Holocremna*) W[[104]](#footnote-105)]

***nigroplica*** (Thomson, 1887, *Olesicampa*) E S

***pagana*** (Holmgren, 1860, *Limneria*)

***paludicola*** (Holmgren, 1860, *Limneria*) E I

*inculcator* misident.

?*sagittaria* (Müller, 1776, *Ichneumon*)

***patellana*** (Thomson, 1887, *Olesicampa*) S I NHM, det. Perkins, added here

***praecox*** (Holmgren, 1860, *Limneria*) I

***proterva*** (Brischke, 1880, *Limneria*) I

*luteipes* (Thomson, 1887, *Olesicampa*)

*subcallosa* (Thomson, 1887, *Olesicampa*)

***pubescens*** (Ratzeburg, 1844, *Campoplex*) I

*hyalinata* (Holmgren, 1860, *Limneria*)

[***ratzeburgi*** (Tschek, 1871, *Limneria*) W[[105]](#footnote-106)]

***retusa*** (Thomson, 1887, *Olesicampa*)

***sericea*** (Holmgren, 1856, *Campoplex*) E I

***sinuata*** (Thomson, 1887, *Holocremna*) E S NHM, det. Perkins, UM, added here

***transiens*** (Ratzeburg, 1848, *Campoplex*) E S W I

*incrassator* (Holmgren, 1856, *Campoplex*) Horstmann (2007*b*)

***vexata*** (Holmgren, 1860, *Limneria*)

***vitripennis*** (Holmgren, 1860, *Limneria*)

doubtfully placed species of *Olesicampe*

[***affinis*** (Parfitt, 1882, *Limneria*) nom. dub.]

[***alienata*** (Gravenhorst, 1829, *Campoplex*) nom. dub.]

***PHOBOCAMPE*** Förster, 1869

*HYPOTHEREUTES* Förster, 1869

*PHOBOCAMPA* Thomson, 1887

***alticollis*** (Thomson, 1887, *Phobocampa*) E added by Horstmann (2006*d*)

***bicingulata*** (Gravenhorst, 1829, *Campoplex*) E S W

***brumatae*** Horstmann, 2009 E S added by Horstmann (2009*d*)

***confusa*** (Thomson, 1887, *Phobocampa*) E S I[[106]](#footnote-107)

***coniferella*** (Roman, 1914, *Phobocampa*) E[[107]](#footnote-108) added by Šedivý (2004)

*facialis* (Szépligeti, 1916, *Holocremnus*)

***crassiuscula*** (Gravenhorst, 1829, *Campoplex*) E S

***croceipes*** (Marshall, 1876, *Limneria*) E S

*albitarsis* Szépligeti, 1916

***flavicincta*** (Thomson, 1887, *Phobocampa*) S NHM, det. Perkins, added here

***horstmanni*** Šedivý, 2004 E S NMS, det. Horstmann, added here

***lymantriae*** Gupta, 1983 E NMS, det. Horstmann, added here

***neglecta*** (Holmgren, 1860, *Limneria*) E S

*varicoxa* (Thomson, 1887, *Anilasta*)

*variicoxa* (Dalla Torre, 1901, *Anilastus*)

***nigra*** Šedivý, 2004 E NHM, det. Horstmann, added here

***pulchella*** (Thomson, 1887, *Phobocampa*) E S

NMS, det. Horstmann, added here

***quercus*** Horstmann, 2008 E added by Horstmann (2008*a*)

***tempestiva*** (Holmgren, 1860, *Limneria*) E S NMS, det. Horstmann, added here

***unicincta*** (Gravenhorst, 1829, *Campoplex*) E

*disparis* (Viereck, 1911, *Hyoposoter*)

***variabilis*** Šedivý, 2004 E NMS, det. Horstmann, added here

***PORIZON*** Fallén, 1813

*PHAEDROCTONUS* Förster, 1869 Horstmann (2004*a*)

***humuli*** (Horstmann, 1987, *Phaedroctonus*) E NMS, det. Horstmann, added here

***moderator*** (Linnaeus, 1758, *Ichneumon*) E S

*strobinellae* (Christ, 1791, *Cynipsichneumon*)

*flaviventris* (Ratzeburg, 1844, *Campoplex*)

*punctulatus* (Ratzeburg, 1844, *Cremastus*) Horstmann (1999*a*)

*ensifer* (Brischke, 1880, *Limneria*)

***transfuga*** (Gravenhorst, 1829, *Campoplex*) E S W I M

*syringellae* Hedwig, 1944

***PYRACMON*** Holmgren, 1859

***fumipennis*** (Zetterstedt, 1838, *Porizon*) I

***sepiellus*** (Holmgren, 1860, *Limneria*) E NHM, det. Horstmann, added here

***RHIMPHOCTONA*** Förster, 1869

*PARAPYRACMON* Clément, 1924

subgenus ***XYLOPHYLAX*** Kriechbaumer, 1878

***megacephalus*** (Gravenhorst, 1829, *Campoplex*) E

*corvina* (Gravenhorst, 1829, *Phytodietus*)

*austriaca* (Tschek, 1871, *Pyracmon*)

*megalocephalus* (Schulz, 1906, *Campoplex*)

*rufipes* (Lange, 1911, *Pyracmon*) preocc.

*hungarica* (Kiss, 1926, *Pyracmon*)

***melanura*** (Holmgren, 1860, *Pyracmon*)

*signata* (Habermehl, 1922, *Pyracmon*)

***obscuripes*** (Holmgren, 1860, *Pyracmon*) I

*alpina* (Strobl, 1904, *Pyracmon*)

***xoridiformis*** (Holmgren, 1860, *Pyracmon*)

*nigerrima* (Kiss, 1924, *Eclytus*)

***SCIRTETES*** Hartig, 1838

*SPUDASTICA* Förster, 1869

***robustus*** (Woldstedt, 1874, *Limneria*) E S I

*kriechbaumeri* (Bridgman, 1882, *Limneria*)

*petiolaris* (Thomson, 1887, *Spudastica*)

***SINOPHORUS*** Förster, 1869

*EULIMNERIA* Schmiedeknecht, 1907

***albidus*** (Gmelin, 1790, *Ichneumon*) E S

*hungaricus* (Szépligeti, 1916, *Omorgus*)

***bridgmanii*** (Dalla Torre, 1901, *Limnerium*) E

*distinctus* (Bridgman, 1887, *Limneria*) preocc.

*renominatus* (Morley, 1915, *Limnerium*)

***costalis*** (Thomson, 1887, *Limneria*) I

***crassifemur*** (Thomson, 1887, *Limneria*) I

***fuscicarpus*** (Thomson, 1887, *Limneria*) E added by Sanborne (1984)

***geniculatus*** (Gravenhorst, 1829, *Campoplex*) I

*nigritellus* (Thomson, 1887, *Limneria*)

*argentator* (Aubert, 1960, *Campoplex*)

***juniperinus*** (Holmgren, 1856, *Campoplex*) E I

added by Sanborne (1984)

*ornatus* (Gregor, 1941, *Omorgus*)

***pleuralis*** (Thomson, 1887, *Limneria*) E added by Sanborne (1984)

***turionum*** (Ratzeburg, 1844, *Campoplex*) E S I

Sanborne (1984)

*spectabilis* (Rudow, 1883, *Limneria*)

*planiscapus* (Thomson, 1887, *Limneria*)

*rufifemur* (Thomson, 1887, *Limneria*)

*nigrotibialis* (Kiss, 1926, *Eulimneria*)

*alkae* (Ellinger & Sachtleben, 1928, *Limnerium*) I

***xanthostomus*** (Gravenhorst, 1829, *Campoplex*)

*pineticola* (Thomson, 1887, *Limneria*)

*deserticola* (Tosquinet, 1896, *Campoplex*)

doubtfully placed species of *Sinophorus*

[***paniscus*** (Gravenhorst, 1829, *Campoplex*)[[108]](#footnote-109) nom. dub.]

***SYNETAERIS*** Förster, 1869

***heteropus*** Thomson, 1887 S

***TRANOSEMA*** Förster, 1869

***carbonellum*** (Thomson, 1887, *Synetaeris*) E S

NHM, det. Broad, Perkins, added here

*aterrimum* (Strobl, 1904, *Pyracmon*)

*rossicum* (Szépligeti, 1916, *Canidia*)

***exoletum*** (Thomson, 1887, *Omorga*) E NHM, det. Perkins, UM, added here

*geniculatum* (Ulbricht, 1910, *Omorgus*) unavailable

***hyperboreum*** (Thomson, 1887, *Limneria*) E NMS, det. Horstmann, added here

***intermedium*** (Szépligeti, 1916, *Gonotypus*) E NMS, det. Horstmann, added here

*majus* (Szépligeti, 1916, *Gonotypus*)

*minus* (Szépligeti, 1916, *Gonotypus*)

***latiusculum*** (Thomson, 1887, *Omorga*) E S NMS, NHM, det. Horstmann, added here

***nigridens*** (Thomson, 1887, *Omorga*) E added by Horstmann (1978*a*)

*striolatum* (Thomson, 1887, *Omorga*)

*alpinator* Aubert, 1966

***rostrale*** (Brischke, 1880, *Limneria*) E S W

*arenicola* Thomson, 1887

*thuringiacum* (Schmiedeknecht, 1907, *Sinophorus*)

***TRANOSEMELLA*** Horstmann, 1978

***citrofrontalis*** (Hedwig, 1939, *Anilasta*) E S W I

NHM, NMS, det. Horstmann, Shaw, Broad, added here

***coxalis*** (Brischke, 1880, *Limneria*)[[109]](#footnote-110) E

***praerogator*** (Linnaeus, 1758, *Ichneumon*) E S W I M

Shaw (1981*a*)

*chrysogaster* (Gmelin, 1790, *Ichneumon*) preocc.

*mandibulator* (Thunberg, 1824, *Ichneumon*)

*interrupta* (Holmgren, 1858, *Limneria*)

*laticrus* (Thomson, 1887, *Angitia*)

***VENTURIA*** Schrottky, 1902

*IDECHTHIS* Förster, 1869

*DEVORGILLA* Cameron, 1907

*NEMERITIS* misident.

***canescens*** (Gravenhorst, 1829, *Campoplex*) E S I

*frumentaria* (Rondani, 1874, *Campoplex*)

*orientalis* (Schmiedeknecht, 1909, *Omorgus*)

*compressa* (Hedwig, 1962, *Angitia*)

Subfamily COLLYRIINAE Cushman, 1924[[110]](#footnote-111)

***COLLYRIA*** Schiødte, 1839

*PACHYMERUS* Gravenhorst, 1829

***coxator*** (Villers, 1789, *Ichneumon*) E

?*falcata* (Geoffroy, 1785, *Ichneumon*)

?*arcuata* (Olivier, 1792, *Ichneumon*)

*calcitrator* (Gravenhorst, 1807, *Bassus*)

*puncticeps* (Thomson, 1877, *Pachymerus*)

*calcitratrix* Schulz, 1906

***trichophthalma*** (Thomson, 1877, *Pachymerus*) E

Subfamily CREMASTINAE Förster, 1869[[111]](#footnote-112)

***CREMASTUS*** Gravenhorst, 1829

***bellicosus*** Gravenhorst, 1829 E

*partitus* Szépligeti, 1899

*meridionator* Aubert, 1960

***cephalotes*** Šedivý, 1970 E

*ponticus* Kolarov, 1982

***geminus*** Gravenhorst, 1829 E S W I

*areolaris* Strand, 1918

***infirmus*** Gravenhorst, 1829 E I

*filicaudis* Szépligeti, 1905

***kratochvili*** Šedivý, 1970 E added by Fitton & Gauld (1980)

***pungens*** Gravenhorst, 1829 E I

*laeviusculus* Thomson, 1890

***spectator*** Gravenhorst, 1829 E W I

*binotatus* Gravenhorst, 1829

*melanarius* Szépligeti, 1901

species excluded from the British and Irish list by Fitton & Gauld (1980)

[***crassicornis*** Thomson, 1890]

***DIMOPHORA*** Förster, 1869

*DIMOPHORUS* Thomson, 1889

***nitens*** (Gravenhorst, 1829, *Campoplex*) E

*robusta* Brischke, 1880

*similis* Brischke, 1880

*arenicola* (Thomson, 1890, *Dimophorus*)

***PRISTOMERUS*** Curtis, 1836

***armatus*** (Lucas, 1849, *Collyria*) E NMS, det. Shaw & Narolsky, added here

*glandarius* (Rondani, 1877, *Odontomerus*)

*gratiosus* Tosquinet, 1896

*cingulatus* Szépligeti, 1905

***horribilis*** Narolsky, 1987 E NHM, det. Broad, added here

***vulnerator*** (Panzer, 1799, *Ichneumon*) E I

*marginalis* Habermehl, 1923

*stigmaticus* Hellén, 1949

***TEMELUCHA*** Förster, 1869

*PARACREMASTUS* Szépligeti, 1899

***arenosa*** (Szépligeti, 1899, *Cremastus*) E I added by Fitton & Gauld (1980)

***interruptor*** (Gravenhorst, 1829, *Cremastus*) E

*buoliana* (Curtis, 1854, *Cremastus*)

***ophthalmica*** (Holmgren, 1860, *Cremastus*) E added by Fitton & Gauld (1980)

***signata*** (Holmgren, 1860, *Cremastus*) E I added by Fitton & Gauld (1980)

species excluded from the British and Irish list by Fitton & Gauld (1980)

[***decorata*** (Gravenhorst, 1829, *Cremastus*)]

[***subnasuta*** (Thomson, 1890, *Cremastus*)]

Subfamily CRYPTINAE Kirby, 1837

PHYGADEUONTINAE Förster, 1869

GELINAE Viereck, 1918

HEMITELINAE Förster, 1869

Tribe CRYPTINI Kirby, 1837[[112]](#footnote-113)

MESOSTENINI Ashmead, 1900

ECHTHRINI Narayanan & Kundanlal, 1958

***ACRORICNUS*** Ratzeburg, 1852

*XENODOCON* Förster, 1855

*MACROBATUS* Holmgren, 1856

*LINOCERAS* Taschenberg, 1865

*LEPTOBATIDES* Buysson, 1896

***stylator*** (Thunberg, 1824, *Ichneumon*) E I

*macrobatus* (Gravenhorst, 1829, *Cryptus*)

*schaumii* Ratzeburg, 1852

*clavator* (Holmgren, 1856, *Macrobatus*)

*exannulatus* (Kriechbaumer, 1894, *Linoceras*) unavailable

***AGROTHEREUTES*** Förster, 1850

*SPILOCRYPTUS* Thomson, 1873

***abbreviatus*** (Fabricius, 1794, *Ichneumon*) E W I M[[113]](#footnote-114)

*abbreviator* (Fabricius, 1793, *Ichneumon*) misident.

*abbreviator* (Fabricius, 1798, *Ichneumon*) preocc.

*breviator* (Thunberg, 1824, *Ichneumon*)

*marginellus* (Gravenhorst, 1829, *Cryptus*) Horstmann (2001*d*)

*pygoleucus* (Gravenhorst, 1829, *Cryptus*)

*tibiator* (Gravenhorst, 1829, *Cryptus*)

*ocellator* (Zetterstedt, 1838, *Cryptus*)

*evanescens* (Ratzeburg, 1852, *Cryptus*)

*leucomerus* (Ratzeburg, 1852, *Cryptus*)

*dispar* (Thomson, 1873, *Spilocryptus*)

*destitutus* Vollenhoven, 1879

*spectabilis* (Rudow, 1886, *Aptesis*)

*tricolor* (Rudow, 1886, *Aptesis*)

*brevipennis* (Kriechbaumer, 1893, *Spilocryptus*) preocc.

*spectabilis* (Rudow, 1914, *Aptesis*) preocc.

*atratus* (Rudow, 1917, *Stibeutes*) preocc.

*livonensis* (Rudow, 1917, *Aptesis*)

*spectabilis* (Rudow, 1917, *Aptesis*) preocc.

*tricolor* (Rudow, 1917, *Aptesis*)

*cingulatus* (Kiss, 1924, *Gambrus*)

*variegatus* (Kiss, 1924, *Gambrus*) preocc.

*alpium* Heinrich, 1951 Schwarz (2005)

***adustus*** (Gravenhorst, 1829, *Cryptus*)

*albolineatus* (Gravenhorst, 1829, *Cryptus*)

*nubeculatus* (Gravenhorst, 1829, *Cryptus*)

*opisoleucus* (Gravenhorst, 1829, *Cryptus*)

*leucostictus* (Hartig, 1838, *Cryptus*)

*melanocerus* (Ulbricht, 1916, *Spilocryptus*) unavailable

***aterrimus*** (Gravenhorst, 1829, *Cryptus*)

*bicingulatus* (Gravenhorst, 1829, *Cryptus*)

***fumipennis*** (Gravenhorst, 1829, *Cryptus*) E W

*zygaenarum* (Thomson, 1873, *Spilocryptus*)

*hymotomadum* (Rudow, 1883, *Cryptus*)

*nigricans* (Kiss, 1915, *Spilocryptus*)

*gracilentus* (Habermehl, 1929, *Spilocryptus*)

***hospes*** (Tschek, 1871, *Cryptus*)

*solitarius* (Tschek, 1871, *Cryptus*)

*intermedius* (Verhoeff, 1890, *Cryptus*) preocc.

***leucorhaeus*** (Donovan, 1810, *Ichneumon*) E S

*migrator* misident.

*bombycis* (Boudier, 1836, *Cryptus*)

*?tibialis* (Thomson, 1873, *Spilocryptus*)[[114]](#footnote-115)

*temporalis* (Szépligeti, 1916, *Gambrus*) Schwarz (2005)

***mandator*** (Linnaeus, 1758, *Ichneumon*) E S

*ischioleucus* (Gravenhorst, 1829, *Cryptus*) Schwarz (2005)

*cimbicis* (Tschek, 1871, *Cryptus*)

***mansuetor*** (Tschek, 1871, *Cryptus*) S added by Schwarz & Shaw (1998)

*nasutus* (Thomson, 1873, *Spilocryptus*)

*curiosus* (Szépligeti, 1916, *Gambrus*) Schwarz (2005)

***saturniae*** (Boie, 1855, *Cryptus*) E S I

*pavoniae* (Bauer, 1937, *Spilocryptus*)

***APSILOPS*** Förster, 1869

*DAPANUS* Förster, 1869

*HETEROTYPUS* Förster, 1869

*SOBAS* Förster, 1869

*TRICHOCRYPTUS* Thomson, 1873

***aquaticus*** (Thomson, 1874, *Trichocrytus*) E

*napiformis* (Rudow, 1882, *Cryptus*)

***cinctorius*** (Fabricius, 1775, *Ichneumon*) E W

*scirpi* (Geoffroy, 1785, *Ichneumon*)

*spinuosus* (Rudow, 1886, *Phygadeuon*)

***ARITRANIS*** Förster, 1869[[115]](#footnote-116)

*PYCNOCRYPTUS* Thomson, 1873

***director*** (Thunberg, 1824, *Ichneumon*) E S W I M

*peregrinator* misident.

***nigripes*** (Gravenhorst, 1829, *Cryptus*) E

*fuscomarginatus* (Gravenhorst, 1829, *Cryptus*)

*insectator* (Tschek, 1871, *Cryptus*)

*jonicus* (Tschek, 1872, *Cryptus*)

***occisor*** (Gravenhorst, 1829, *Cryptus*)[[116]](#footnote-117)

*gracilis* (Taschenberg, 1865, *Cryptus*) preocc.

Schwarz (2005)

*fuscicornis* (Tschek, 1871, *Cryptus*) Schwarz (2005)

*notabilis* (Habermehl, 1926, *Hoplocryptus*)

Schwarz (2005)

*punguri* (Kiss, 1915, *Spilocryptus*) Schwarz (2005)

***ATELEUTE*** Förster, 1869

*ATELEUTA* Schulz, 1906

***linearis*** Förster, 1871 E added by Schwarz & Shaw (1998)

*lissonotoides* (Thomson, 1885, *Hemiteles*)

*egregia* (Schmiedeknecht, 1933, *Hemiteles*)

***BUATHRA*** Cameron, 1903

***laborator*** (Thunberg, 1824, *Ichneumon*) E S W

*fabricii* (Schiødte, 1839, *Cryptus*)

*fulvipes* (Magretti, 1884, *Cryptus*) Horstmann (2004*c*)

*fulvipes* (Habermehl, 1902, *Cryptus*)

***tarsoleuca*** (Schrank, 1781, *Ichneumon*) E S

*leucopus* (Gmelin, 1790, *Ichneumon*)

*leucotarsos* (Gmelin, 1790, *Ichneumon*)

*curvicauda* (Thomson, 1896, *Cryptus*)

***CAENOCRYPTUS*** Thomson, 1873

***rufiventris*** (Gravenhorst, 1829, *Cryptus*)[[117]](#footnote-118) E S

*eborinus* (Ratzeburg, 1852, *Cryptus*)

*collaris* (Rudow, 1883, *Cryptus*) preocc.

***CRYPTUS*** Fabricius, 1804

*EUCRYPTUS* Haldeman, 1842

*ITAMOPLEX* Förster, 1869

***apparitorius*** (Villers, 1789, *Ichneumon*)

*pungens* Gravenhorst, 1829

*gratiosus* Tschek, 1871

*histrionicus* Rudow, 1882

***arenicola*** Thomson, 1873 E added by Schwarz (2005)[[118]](#footnote-119)

***armator*** Fabricius, 1804 E S W

*albatorius* misident.

*cunctator* (Fabricius, 1793, *Ichneumon*) Horstmann (2001*b*)

*rusticator* Zetterstedt, 1838

*filicornis* Rudow, 1886 preocc.

***dianae*** Gravenhorst, 1829 E

*gracilicornis* Gravenhorst, 1829

*leucostomus* Gravenhorst, 1829

*stenogaster* Gravenhorst, 1829

*seticornis* (Ratzeburg, 1844, *Ichneumon*)

*bolivari* Kriechbaumer, 1898

*solitarius* Habermehl, 1909 preocc.

*solitarius* Habermehl, 1918 preocc.

***fibulatus*** Gravenhorst, 1829 S W I added by Schwarz & Shaw (1998)

*rhenanus* Ulbricht, 1911

*antennalis* Szépligeti, 1916

***inculcator*** (Linnaeus, 1758, *Ichneumon*)

*sponsor* (Fabricius, 1793, *Ichneumon*)

*regenerator* (Panzer, 1804, *Ichneumon*)

*quadrilineatus* Gravenhorst, 1829

*filicornis* Ratzeburg, 1844

*bicolor* Rudow, 1882 preocc.

*erythrostoma* Rudow, 1882

*lippensis* Rudow, 1883

*albopictus* Seyrig, 1928 preocc.

***minator*** Gravenhorst, 1829

***moschator*** (Fabricius, 1787, *Ichneumon*) E

*polytropus* Heinrich, 1951 Schwarz (2005)

***obscuripes*** Zetterstedt, 1838 E NHM, det. Schwarz, added here

*borealis* Thomson, 1873 preocc.

*carpathicus* Szépligeti, 1916

***spinosus*** Gravenhorst, 1829 E

*armatorius* misident.[[119]](#footnote-120)

*leucostictus* Gravenhorst, 1829 Horstmann (2001*d*)

***spiralis*** (Geoffroy, 1785, *Ichneumon*)

*inconspicuus* Gravenhorst, 1829

*hispanicus* Habermehl, 1918

***titubator*** (Thunberg, 1824, *Ichneumon*) E S W I M

*difficilis* Tschek, 1871

*infumatus* Thomson, 1873

***tuberculatus*** Gravenhorst, 1829 E

*investigator* Tschek, 1871

*solivagus* Rossem, 1989

***viduatorius*** Fabricius, 1804 E S W I

*germari* Taschenberg, 1865

***ECHTHRUS*** Gravenhorst, 1829[[120]](#footnote-121)

*SPHAETES* Bremi, 1849

***reluctator*** (Linnaeus, 1758, *Ichneumon*) E

*usurpator* (Scopoli, 1763, *Ichneumon*)

*obex* (Müller, 1776, *Ichneumon*)

*rubiginosus* (Christ, 1791, *Ichneumon*)

*carbonator* (Thunberg, 1824, *Ichneumon*)

*chirothecator* (Thunberg, 1824, *Ichneumon*)

*ternator* (Thunberg, 1824, *Ichneumon*)

*crassicrus* (Bremi, 1849, *Sphaetes*)

*corsicus* (Marshall, 1901, *Nyxeophilus*)

*nigerrimus* Strobl, 1902

***ENCLISIS*** Townes, 1970

***alpicola*** (Habermehl, 1926, *Caenocryptus*) E added by Schwarz (1989)[[121]](#footnote-122)

***macilenta*** (Gravenhorst, 1829, *Cryptus*) E W

*remex* (Tschek, 1871, *Cryptus*)

*inflata* (Thomson, 1873, *Caenocryptus*)

*gracilipes* (Gravenhorst, 1829, *Cryptus*) Sawoniewicz (2003)

*antennata* (Bridgman, 1881, *Cryptus*)

*laticrus* (Thomson, 1896, *Caenocryptus*)

*exareolata* (Strobl, 1901, *Chaeretymma*)

*rubi* (Habermehl, 1921, *Microcryptus*)

*alboclypeata* (Kiss, 1924, *Hoplocryptus*)

***ruficeps*** (Desvignes, 1856, *Cryptus*)

*pulchella* Schwarz, 1989

***vindex*** (Tschek, 1871, *Cryptus*) E S

*pubiventris* (Thomson, 1873, *Caenocryptus*)

*tener* (Thomson, 1873, *Caenocryptus*)

*nubifer* (Thomson, 1896, *Caenocryptus*)

*striolata* (Thomson, 1896, *Caenocryptus*)

*nigriventris* (Habermehl, 1919, *Caenocryptus*)

***GAMBRUS*** Förster, 1869

*KALTENBACHIA* Förster, 1869

*HYGROCRYPTUS* Thomson, 1873

***amoenus*** (Gravenhorst, 1829, *Cryptus*)

***bipunctatus*** (Tschek, 1872, *Cryptus*) E S W added by Schwarz & Shaw (1998)[[122]](#footnote-123)

*ornatus* misident. Schwarz & Shaw (1998)

*maculatus* Brischke, 1888 Schwarz (2005)

***carnifex*** (Gravenhorst, 1829, *Cryptus*) E S W

*varicoxis* (Taschenberg, 1865, *Cryptus*)

***incubitor*** (Linnaeus, 1758, *Ichneumon*) E S[[123]](#footnote-124)

*vibex* (Müller, 1776, *Ichneumon*)

*upsaliensis* (Geoffroy, 1785, *Ichneumon*)

*superus* Thomson, 1896 Schwarz & Shaw (1998)

*quadricinctus* (Strobl, 1901, *Spilocryptus*) Schwarz & Shaw (1998)

*incertus* Habermehl, 1935 preocc. Schwarz (2005)

***ornatus*** (Gravenhorst, 1829, *Cryptus*) E S W I[[124]](#footnote-125)

added by Schwarz (2005)

*ornatulus* (Thomson, 1873, *Spilocryptus*) Schwarz & Shaw (1998)

*inferus* Thomson, 1896

*quadricinctus* (Strobl, 1901, *Spilocryptus*) Schwarz & Shaw (1998)

*ruficoxis* Habermehl, 1919

*meridionator* (Aubert, 1965, *Agrothereutes*) preocc.

Schwarz (2005)

***tricolor*** (Gravenhorst, 1829, *Cryptus*)[[125]](#footnote-126) E S W

*subcinctus* (Gravenhorst, 1829, *Cryptus*)

*opacus* Szépligeti, 1916 Schwarz (2005)

***HELCOSTIZUS*** Förster, 1869[[126]](#footnote-127)

*BRACHYCENTRUS* Taschenberg, 1865 preocc.

*CYRTOCRYPTUS* Marshall, 1872

*MESOCRYPTUS* Thomson, 1873

*HETEROCRYPTUS* Woldstedt, 1874

*CHENBERGUS* Navás, 1930

***restaurator*** (Fabricius, 1775, *Ichneumon*) E I

*albator* (Thunberg, 1824, *Ichneumon*)

*brachycentrus* (Gravenhorst, 1829, *Cryptus*)

*crassipes* (Hartig, 1847, *Echthrus*)

*hercynianus* (Hartig, 1847, *Echthrus*)

*pimplarius* (Taschenberg, 1865, *Brachycentrus*)

*fuscitarsis* (Haupt, 1917, *Perosis*)

*turcicus* (Fahringer, 1944, *Brachycentrus*)

*serraticornis* (Haupt, 1954, *Perosis*)

***HIDRYTA*** Förster, 1869

*BRACHYCRYPTUS* Thomson, 1873

*EUTHYCRYPTUS* Jussel, 1907

***nigricoxa*** (Provancher, 1888, *Cryptus*) W added by Schwarz & Shaw (1998)

*scrobiculifer* (Jussel, 1907, *Euthycryptus*)

***sordida*** (Tschek, 1871, *Cryptus*) E S W I added by Edgar (1971)[[127]](#footnote-128)

*melanopus* (Taschenberg, 1865, *Cryptus*) preocc.

Schwarz (2005)

*erythrocera* (Thomson, 1873, *Brachycryptus*)

*sordidula* (Thomson, 1873, *Brachycryptus*)

*hueberi* (Dalla Torre, 1901, *Cryptus*) Schwarz (2005)

*nigritarsis* (Habermehl, 1918, *Idiolispa*)

*pygmaea* (Habermehl, 1918, *Idiolispa*)

*henrichi* (Kiss, 1924, *Habrocryptus*)

?*atlantica* Horstmann, 1990 tentative synonymy by Schwarz (2005)

***HOPLOCRYPTUS*** Thomson, 1873

***bellosus*** (Curtis, 1837, *Cryptus*) E I

*signatorius* (Fabricius, 1793, *Ichneumon*) preocc.

*pulcher* Thomson, 1873

*fuscipes* (Tschek, 1871, *Cryptus*)

*thoracicus* (Brischke, 1881, *Hygrocryptus*)

*macrophyiae* (Rudow, 1911, *Cryptus*)

***bohemani*** (Holmgren, 1856, *Cryptus*)

*rufoniger* (Desvignes, 1856, *Cryptus*) Horstmann (2000*b*)

*mesoxanthus* Thomson, 1873

***confector*** (Gravenhorst, 1829, *Cryptus*) E

*albus* (Taschenberg, 1865, *Cryptus*)

*brachysoma* (Taschenberg, 1865, *Cryptus*)

Schwarz (2005)

*dubius* (Taschenberg, 1865, *Cryptus*)[[128]](#footnote-129)

*elegans* Thomson, 1873

*thomsoni* (Bridgman, 1881, *Cryptus*)

*gladiator* Kriechbaumer, 1899

*caudatus* Szépligeti, 1916

*quadratus* (Szépligeti, 1916, *Gambrus*) preocc.

*gallicus* (Habermehl, 1923, *Gambrus*)

*enslini* (Habermehl, 1923, *Spilocryptus*) preocc.

*exannulatus* Habermehl, 1926

*hungaricus* Habermehl, 1926 preocc.

***melanocephalus*** (Gravenhorst, 1829, *Cryptus*) E

added by Schwarz (2007)

***murarius*** (Börner, 1782, *Ichneumon*)[[129]](#footnote-130)

*olitorius* (Fabricius, 1793, *Ichneumon*) Horstmann (2001*b*)

*fugitivus* (Gravenhorst, 1829, *Cryptus*) Schwarz (2007)

*gracilis* (Gravenhorst, 1829, *Cryptus*)

*binotatulus* Thomson, 1873

*pseudocryptus* (Szépligeti, 1916, *Gambrus*)

*tegularis* (Szépligeti, 1916, *Gambrus*)

*ignalinoensis* Strand, 1918

*ratzeburgi* (Habermehl, 1919, *Spilocryptus*)

*obscurata* (Kiss, 1929, *Habrocryptus*) preocc.

***quadriguttatus*** (Gravenhorst, 1829, *Cryptus*) E

*cognatus* (Fonscolombe, 1850, *Cryptus*) Schwarz (2005)

*mallorcanus* Kriechbaumer, 1894 Schwarz (2007)

*enslini* Habermehl, 1921 Schwarz (2007)

*hebraicator* (Aubert, 1970, *Aritranis*) Schwarz (2005)

*tiloidalis* (Kolarov & Beyarslan, 1994, *Agrothereutes*)

Schwarz (2005)

***IDIOLISPA*** Förster, 1869

*LIOCRYPTUS* Thomson, 1873

*PARACRYPTUS* Szépligeti, 1916

***analis*** (Gravenhorst, 1807, *Bassus*) E S W I

*cursor* (Thunberg, 1824, *Ichneumon*) preocc.

*elevata* (Zetterstedt, 1838, *Cryptus*)

*dubiosa* (Kiss, 1924, *Spilocryptus*)

**?*hungarica*** (Szépligeti, 1916, *Paracryptus*)[[130]](#footnote-131)

?*grossa* misident. Schwarz & Shaw (1998)

*meyeri* (Habermehl, 1926, *Spilocryptus*)

***subalpina*** (Schmiedeknecht, 1904, *Spilocryptus*) S

added by Schwarz & Shaw (1998)

*heydeni* (Habermehl, 1919, *Spilocryptus*)

species of *Idiolispa* deleted from the British and Irish list by Schwarz & Shaw (1998)

[***obfuscator*** (Villers, 1789, *Ichneumon*)[[131]](#footnote-132)]

***ISCHNUS*** Gravenhorst, 1829

*HABROCRYPTUS* Thomson, 1873

***agitator*** (Olivier, 1792, *Ichneumon*)

*destructorius* (Fabricius, 1793, *Ichneumon*)

Horstmann (2001*b*)

*rubricator* (Panzer, 1801, *Ichneumon*)

*constrictor* (Fabricius, 1804, *Cryptus*)

*minutorius* (Fabricius, 1804, *Cryptus*)

*minor* (Thunberg, 1824, *Ichneumon*)

*pictor* (Thunberg, 1824, *Ichneumon*) preocc.

*dineurae* (Rudow, 1882, *Cryptus*)

*oriicus* (De Stefani, 1886, *Cryptus*) Horstmann (2000*b*)

***alternator*** (Gravenhorst, 1829, *Cryptus*) E S W

*unicinctus* (Gravenhorst, 1829, *Cryptus*)

*striatellus* (Zetterstedt, 1838, *Cryptus*)

*annulipes* (Taschenberg, 1865, *Cryptus*)

*annulitarsis* (Rudow, 1882, *Cryptus*)

***inquisitorius*** (Müller, 1776, *Ichneumon*)[[132]](#footnote-133) E

*migrator* misident.

*dictator* (Geoffroy, 1785, *Ichneumon*)

*porrectorius* (Fabricius, 1787, *Ichneumon*)

*leucostictos* (Gmelin, 1790, *Ichneumon*)

*sanguinolentus* (Gmelin, 1790, *Ichneumon*)

*assertorius* (Fabricius, 1793, *Ichneumon*)

*zonator* (Fabricius, 1793, *Ichneumon*)

*triplicatorius* (Thunberg, 1824, *Ichneumon*)

*brachyurus* (Gravenhorst, 1829, *Cryptus*)

*geminus* (Gravenhorst, 1829, *Cryptus*) Sawoniewicz (2003)

*sannio* (Gravenhorst, 1829, *Cryptus*)

*sedulus* (Gravenhorst, 1829, *Cryptus*) Sawoniewicz (2003)

*flavopictus* (Rudow, 1883, *Cryptus*)

*alpinus* (Strobl, 1901, *Habrocryptus*)

*obscuratus* (Kiss, 1924, *Habrocryptus*)

***migrator*** (Fabricius, 1775, *Ichneumon*) E S

*collaris* (Tschek, 1872, *Cryptus*) Horstmann (2001*b*)

*punctiger* (Thomson, 1896, *Habrocryptus*) Schwarz & Shaw (1998)

*insulanus* (Krieger, 1897, *Habrocryptus*) Schwarz & Shaw (1998)

*helveticator* Aubert, 1968 Schwarz (2005)

***LISTROGNATHUS*** Tschek, 1871

*MESOSTENIDEA* Viereck, 1914

*MESOSTENUS* misident.

***firmator*** (Fabricius, 1798, *Ichneumon*)[[133]](#footnote-134) E Horstmann (1990*a*)

*ligator* (Gravenhorst, 1829, *Mesostenus*) Horstmann (2001*b*)

*senilis* (Rudow, 1882, *Cryptus*)

*aculeatus* (Rudow, 1883, *Cryptus*)

***mactator*** (Thunberg, 1824, *Ichneumon*)

*niveatus* (Gravenhorst, 1829, *Mesostenus*)

*pygostolus* (Gravenhorst, 1829, *Mesostenus*)

*tricolor* Tschek, 1872

*intermedius* (Szépligeti, 1916, *Mesostenus*)

***mengersseni*** Schmiedeknecht, 1905 E added by Horstmann (1990*a*)

***obnoxius*** (Gravenhorst, 1829, *Mesostenus*) E W

*zygaenarum* (Ratzeburg, 1847, *Cryptus*) Horstmann (1997)

*subovalis* (Thomson, 1873, *Mesostenus*)

*robustus* (Rudow, 1882, *Cryptus*) preocc.

*subcircularis* (Thomson, 1896, *Mesostenus*)

***MERINGOPUS*** Förster, 1869

*GONIOCRYPTUS* Thomson, 1873

***attentorius*** (Panzer, 1804, *Ichneumon*)

*confiscator* (Fabricius, 1804, *Cryptus*) Horstmann (2001*b*)

*alboannulatus* (Szépligeti, 1916, *Cryptus*)

***cyanator*** (Gravenhorst, 1829, *Cryptus*)

?*fuscescens* (Gmelin, 1790, *Ichneumon*)

?*roeselii* (Bechstein & Scharfenberg, 1805, *Ichneumon*)

***titillator*** (Linnaeus, 1758, *Ichneumon*) E

*recreator* (Fabricius, 1804, *Cryptus*)

*tornator* (Panzer, 1804, *Ichneumon*)

*pupurator* (Thunberg, 1824, *Ichneumon*)

*australis* (Tschek, 1871, *Cryptus*) preocc.

*latitarsis* (Thomson, 1873, *Cryptus*)

*pectinitarsis* (Rudow, 1882, *Cryptus*)

*titillatrix* (Schulz, 1906, *Trychosis*)

*meridionalis* (Szépligeti, 1916, *Cryptus*)

*orientalis* (Szépligeti, 1916, *Cryptus*)

*nigripes* (Seyrig, 1927, *Cryptus*)

***MESOSTENUS*** Gravenhorst, 1829

*STENARAEUS* Thomson, 1896

***transfuga*** Gravenhorst, 1829 E

*gallarum* (Rudow, 1881, *Hemiteles*)

*gallarum* (Rudow, 1882, *Hemiteles*) preocc.

*ingenuus* Tosquinet, 1896

*niger* Kiss, 1929 preocc. Schwarz (2005)

***NEMATOPODIUS*** Gravenhorst, 1829

*PSEUDOPIMPLA* Fahringer, 1935 preocc.

***debilis*** (Ratzeburg, 1852, *Mesostenus*) E

*formosus* misident.

*tricolor* (Haupt, 1954, *Mesostenus*) preocc.

*homonymator* (Aubert, 1959, *Mesostenus*)

***PICARDIELLA*** Lichtenstein, 1920

*BORCIELLA* Constantineanu, 1929

*NIPPORICNUS* Uchida, 1931

*PARETHA* Seyrig, 1952

***melanoleuca*** (Gravenhorst, 1829, *Cryptus*) E NHM, det. Broad, added here

*argiola* (Rudow, 1882, *Mesostenus*)

*peregrina* (Schmiedeknecht, 1905, *Mesostenus*)

*crenulata* (Constantineanu, 1929, *Borciella*)

*tarsoleuca* (Kiss, 1929, *Habrocryptus*)

***POLYTRIBAX*** Förster, 1869[[134]](#footnote-135)

*EPIPHOBUS* Förster, 1869

*NELEOPHRON* Förster, 1869

*PLESIGNATHUS* Förster, 1869

***arrogans*** (Gravenhorst, 1829, *Cryptus*) E S W I M

*sectator* (Gravenhorst, 1829, *Phygadeuon*)

*longipes* (Hartig, 1838, *Cryptus*)

*halensis* (Taschenberg, 1865, *Phygadeuon*)

*nigriventris* (Habermehl, 1917, *Microcryptus*) preocc.

*tricolor* (Fahringer, 1935, *Plectocryptus*)

***perspicillator*** (Gravenhorst, 1807, *Ichneumon*) E S I

*desertor* (Gravenhorst, 1829, *Phygadeuon*)

*obscuripes* (Taschenberg, 1865, *Phygadeuon*)

*errator* (Marshall, 1868, *Phygadeuon*)

*rufofemoratus* (Strobl, 1901, *Plectocryptus*)

*nigrifemur* (Kiss, 1929, *Cryptus*)

*mocsari* (Györfi, 1944, *Megaplectes*)

***picticornis*** (Ruthe, 1859, *Cryptus*) E S W NMS, NHM, added here

*gravenhorstii* (Thomson, 1883, *Microcryptus*)

***rufipes*** (Gravenhorst, 1829, *Cryptus*) E I

*curvus* (Schrank, 1802, *Ichneumon*) preocc.

*rufipes* (Schrank, 1835, *Ichneumon*) preocc.

*vexator* (Pfankuch, 1921, *Microcryptus*)

***SPHECOPHAGA*** Westwood, 1840

*CHRYONOMON* Desvignes, 1856

*CACOTROPA* Förster, 1869

***vesparum*** (Curtis, 1828, *Anomalon*) E S I

*striata* (Zetterstedt, 1838, *Bassus*)

*vesparum* (Ratzeburg, 1852, *Tryphon*) preocc.

*sericea* (Thomson, 1888, *Cacotropa*)

*thuringiaca* Schmiedeknecht, 1914

***THRYBIUS*** Townes, 1965

***brevispina*** (Thomson, 1896, *Hygrocryptus*)[[135]](#footnote-136) E

*puhlmanni* (Ulbricht, 1909, *Hygrocryptus*)

***praedator*** (Rossi, 1792, *Ichneumon*) E W

*leucopygus* (Gravenhorst, 1829, *Hoplismenus*)

*praedator* (Gravenhorst, 1829, *Cryptus*) preocc.

*sanguinolentus* (Gravenhorst, 1829, *Cryptus*)

*elegans* (Desvignes, 1856, *Cryptus*)

*drewseni* (Thomson, 1873, *Hygrocryptus*)

*picticornis* (Rudow, 1882, *Cryptus*) preocc.

*praedatrix* (Schulz, 1906, *Aritranis*)

*puhlmanni* (Ulbricht, 1909, *Hygrocryptus*)

*continuus* (Ulbricht, 1910, *Hygrocryptus*) unavailable

*atrocoxatus* (Ulbricht, 1916, *Hygrocryptus*) unavailable

***TRYCHOSIS*** Förster, 1869

***ambigua*** (Tschek, 1871, *Cryptus*) E NHM, det. Schwarz, added here

*mesocastana* (Tschek, 1871, *Cryptus*) Horstmann (2005*b*)

*molesta* (Tschek, 1871, *Cryptus*)

*annulicornis* (Thomson, 1896, *Goniocryptus*)

*trisculpta* (Habermehl, 1929, *Goniocryptus*)

Schwarz (2005)

***atripes*** (Gravenhorst, 1829, *Cryptus*) E NHM, det. Schwarz, added here

*castaniventris* (Tschek, 1871, *Cryptus*)

*curvipes* (Tschek, 1871, *Cryptus*)

*jugorum* (Strobl, 1901, *Idiolispa*)

**?*ingrata*** (Tschek, 1871, *Cryptus*) E[[136]](#footnote-137) added by Schwarz & Shaw (1998)

*macroura* (Thomson, 1873, *Goniocryptus*)

***insularis*** Rossem, 1990 E added by Schwarz & Shaw (1998)

***legator*** (Thunberg, 1824, *Ichneumon*) E S W I M

*mesocastana* misident.

*titillator* misident.

*bicolor* (Lucas, 1849, *Cryptus*)

*abnormis* (Tschek, 1871, *Cryptus*)

*inimica* (Tschek, 1871, *Cryptus*)

*plebeja* (Tschek, 1871, *Cryptus*)

*rustica* (Tschek, 1871, *Cryptus*)

*simulator* (Tschek, 1871, *Cryptus*)

*clypearis* (Thomson, 1873, *Goniocryptus*)

*parvula* (Kriechbaumer, 1894, *Goniocryptus*)

*simulatrix* Schulz, 1906

*timenda* Rossem, 1990

***neglecta*** (Tschek, 1871, *Cryptus*) E NHM, det. Schwarz, added here

***picta*** (Thomson, 1873, *Goniocryptus*) E S[[137]](#footnote-138) added by Schwarz & Shaw (1998)

***tristator*** (Tschek, 1871, *Cryptus*) E added by Schwarz & Shaw (1998); UM

*glabricula* (Thomson, 1873, *Goniocryptus*)

*pleuralis* (Thomson, 1896, *Goniocryptus*)

*tristatrix* Schulz, 1906

***XYLOPHRURUS*** Förster, 1869

*NYXEOPHILUS* Förster, 1869

*MACROCRYPTUS* Thomson, 1873

*NYXEOPHILUS* Thomson, 1885 preocc.

***lancifer*** (Gravenhorst, 1829, *Echthrus*) E S added by Schwarz & Shaw (1998)

*dispar* (Thunberg, 1824, *Ichneumon*) preocc.

?*nubeculatus* (Gravenhorst, 1829, *Echthrus*)[[138]](#footnote-139)

?*dentifer* (Thomson, 1896, *Caenocryptus*)

?*castaniventris* (Habermehl, 1909, *Kaltenbachia*)

?*rufescens* (Ozolz, 1942, *Kaltenbachia*)

***tumidus*** (Desvignes, 1856, *Cryptus*)[[139]](#footnote-140) E

*longiseta* (Rudow, 1882, *Cryptus*) preocc.

Species excluded from the British and Irish list by Schwarz & Shaw (1998)

[***STENARELLA*** Szépligeti, 1916

***domator*** (Poda, 1761, *Ichneumon*)

*gladiator* (Scopoli, 1763, *Ichneumon*)]

Tribe HEMIGASTRINI Ashmead, 1900[[140]](#footnote-141)

APTESINI Smith & Shenefelt, 1955

ECHTHRINI Narayanan & Kundanlal, 1958

***ACONIAS*** Cameron, 1904

***tarsatus*** (Bridgman, 1881, *Phygadeuon*) E S I

*pectoralis* (Thomson, 1896, *Plectocryptus*)

*lateannulatus* (Strobl, 1901, *Chaeretymma*)

*nigrofemoratus* (Strobl, 1901, *Plectocryptus*)

***APTESIS*** Förster, 1850

*PEZOPORUS* Förster, 1869

*CLYPEODIODON* Aubert, 1968

***assimilis*** (Gravenhorst, 1829, *Phygadeuon*) I M

*distans* (Thomson, 1883, *Microcryptus*)

***cretata*** (Gravenhorst, 1829, *Phygadeuon*) E

***femoralis*** (Thomson, 1883, *Microcryptus*) E I

*zonata* (Kriechbaumer, 1893, *Microcryptus*)

*alpina* (Strobl, 1901, *Microcryptus*)

***flagitator*** (Rossius, 1794, *Icheumon*) E I

*pumilio* (Gravenhorst, 1829, *Phygadeuon*)

*tyranna* (Gravenhorst, 1829, *Phygadeuon*) preocc.

*hopei* (Desvignes, 1856, *Cryptus*)

*proximator* (Costa, 1886, *Phygadeuon*)

*tricolor* (Kriechbaumer, 1894, *Microcryptus*)

*hopei* (Morley, 1907, *Acanthocryptus*) preocc.

*feketei* (Kiss, 1915, *Acanthocryptus*)

*rufipes* (Obrtel, 1953, *Acanthocryptus*)

***improba*** (Gravenhorst, 1829, *Phygadeuon*) I

*exigua* (Habermehl, 1909, *Microcryptus*)

*bisignata* (Habermehl, 1919, *Microcryptus*)

***jejunator*** (Gravenhorst, 1807, *Ichneumon*) E I

*abdominator* (Gravenhorst, 1829, *Phygadeuon*)

*nematorum* (Rudow, 1886, *Phygadeuon*)

*genalis* (Kriechbaumer, 1895, *Microcryptus*)

*albilarva* (Speiser, 1908, *Microcryptus*)

*brumatae* (Silvestri, 1941, *Microcryptus*)

***nigricollis*** (Thomson, 1883, *Acanthocryptus*) E I[[141]](#footnote-142)

***nigritula*** (Thomson, 1885, *Microcryptus*) E I

*nigripes* (Strobl, 1901, *Stenocryptus*)

***nigrocincta*** (Gravenhorst, 1815, *Ichneumon*) E S W I

?*bimaculata* (Christ, 1791, *Ichneumon*) preocc.

*sudetica* (Gravenhorst, 1815, *Ichneumon*)

*duplicatoria* (Thunberg, 1824, *Ichneumon*)

*flaveolata* (Gravenhorst, 1829, *Phygadeuon*)

*hostilis* (Gravenhorst, 1829, *Cryptus*)

*jucunda* (Gravenhorst, 1829, *Phygadeuon*)

*ephippia* (Rudow, 1914, *Agrothereutes*)

*fulvipes* (Rudow, 1914, *Agrothereutes*)

*haemorrhoidalis* (Rudow, 1914, *Theroscopus*)

*analis* (Rudow, 1917, *Theroscopus*)

*borealis* Rudow, 1917

*clythrae* (Rudow, 1917, *Pezomachus*)

*ephippia* (Rudow, 1917, *Agrothereutes*) preocc.

*fulvipes* (Rudow, 1917, *Agrothereutes*) preocc.

*haemorhoidalis* (Rudow, 1917, *Theroscopus*) preocc.

*nigrocincta* (Rudow, 1917, *Pezomachus*) preocc.

***orbitalis*** (Thomson, 1883, *Microcryptus*)[[142]](#footnote-143) NHM, added here

***scotica*** (Marshall, 1868, *Phygadeuon*) S

***terminata*** (Gravenhorst, 1829, *Phygadeuon*) E

*gilvipes* (Gravenhorst, 1829, *Phygadeuon*)

*ceilonota* (Taschenberg, 1865, *Phygadeuon*)

doubtfully placed species of *Aptesis*

[***leucosticta*** (Gravenhorst, 1829, *Cryptus*) nom. dub.]

***COLOCNEMA*** Förster, 1869

*COELOCRYPTUS* Thomson, 1873

***rufina*** (Gravenhorst, 1829, *Phygadeuon*) E

*romani* (Pfankuch, 1914, *Plectocryptus*)

***CRATOCRYPTUS*** Thomson, 1873

**?*furcator*** (Gravenhorst, 1829, *Cryptus*) S[[143]](#footnote-144)

***subpetiolatus*** (Gravenhorst, 1829, *Cryptus*)[[144]](#footnote-145) E

***CUBOCEPHALUS*** Ratzeburg, 1848

*CHAERETYMMA* Förster, 1869

*ECPORTHETOR* Förster, 1869

*PAMMACHUS* Förster, 1869

*MICROCRYPTUS* Thomson, 1873

*STENOCRYPTUS* Thomson, 1873

*PLANOCRYPTUS* Heinrich, 1949

***anatorius*** (Gravenhorst, 1829, *Cryptus*) E W I

?*dumetorum* (Geoffroy, 1785, *Ichneumon*)

*stomaticus* (Gravenhorst, 1829, *Cryptus*) Sawoniewicz (2003)

*exareolatus* (Habermehl, 1917, *Cratocryptus*)

*albopictus* (Kiss, 1924, *Plectocryptus*)

***associator*** (Thunberg, 1824, *Ichneumon*) E

*ruficoxis* (Thomson, 1873, *Cratocryptus*)

***brevicornis*** (Taschenberg, 1865, *Phygadeuon*) E I

*oviventris* misident.

***distinctor*** (Thunberg, 1824, *Ichneumon*) E S W I[[145]](#footnote-146)

*fortipes* misident.

***femoralis*** (Thomson, 1873, *Cratocryptus*)

*kriegeri* (Habermehl, 1911, *Cratocryptus*)

***nigriventris*** (Thomson, 1874, *Stenocryptus*) E S W I

***sperator*** (Müller, 1776, *Ichneumon*)[[146]](#footnote-147) E S I

*bilineatus* (Gravenhorst, 1829, *Cryptus*)

*erythrinus* (Gravenhorst, 1829, *Cryptus*) Sawoniewicz (2003)

*lacteator* (Gravenhorst, 1829, *Cryptus*)

*semiorbitalis* (Gravenhorst, 1829, *Phygadeuon*)

*cruentus* (Kriechbaumer, 1891, *Microcryptus*)

***DEMOPHELES*** Förster, 1869

***corruptor*** (Taschenberg, 1865, *Phygadeuon*)[[147]](#footnote-148) E S I

***GIRAUDIA*** Förster, 1869

*CALOCRYPTUS* Thomson, 1873

*PSEUDOCRYPTUS* Kriechbaumer, 1893

***grisescens*** (Gravenhorst, 1829, *Cryptus*) I

*scansor* (Thomson, 1890, *Plectocryptus*)

*nigritarsis* (Ulbricht, 1910, *Plectocryptus*) unavailable

***gyratoria*** (Thunberg, 1824, *Ichneumon*)

*congruens* (Gravenhorst, 1829, *Cryptus*)

*dimimilis* (Kiss, 1924, *Megaplectes*)

***JAVRA*** Cameron, 1903

*MONOCRYPTUS* Hellén, 1957

***anomala*** (Morley, 1908, *Diadegma*) E S W

*hedwigi* (Habermehl, 1929, *Microcryptus*)

***opaca*** (Thomson, 1873, *Cratocryptus*) S

*gracilicornis* (Kriechbaumer, 1891, *Microcryptus*)

*jenneri* (Heinrich, 1949, *Microcryptus*)

***tricincta*** (Gravenhorst, 1829, *Cryptus*) E W

*areolaris* (Thomson, 1883, *Microcryptus*)

*prominens* (Schmiedeknecht, 1931, *Microcryptus*)

***LISTROCRYPTUS*** Brauns, 1905

***spatulatus*** Brauns, 1905 E NMS (det. Schwarz), NHM, added here

***MEGAPLECTES*** Förster, 1869

*IOCRYPTUS* Thomson, 1873

*MEGALOPLECTES* Schulz, 1906

***monticola*** (Gravenhorst, 1829, *Ichneumon*)

*regius* (Taschenberg, 1865, *Phygadeuon*)

*andrei* (Berthoumieu, 1897, *Eurylabus*)

*lucens* Torka, 1935

***ORESBIUS*** Marshall, 1867

*OPIDNUS* Förster, 1869

***arridens*** (Gravenhorst, 1829, *Phygadeuon*) E I

*niveatus* (Desvignes, 1856, *Ichneumon*)

*rhombifer* (Kriechbaumer, 1893, *Microcryptus*)

***castaneus*** Marshall, 1867 S

*terrestris* (Roman, 1909, *Microcryptus*)

***funereus*** (Schmiedeknecht, 1905, *Microcryptus*)

***galactinus*** (Gravenhorst, 1829, *Phygadeuon*) I

*fulgens* (Taschenberg, 1865, *Phygadeuon*)

*nigricans* (Pfankuch, 1923, *Microcryptus*) unavailable

***leucopsis*** (Gravenhorst, 1829, *Cryptus*) E I

*nycthemerus* (Gravenhorst, 1829, *Phygadeuon*)

*brumatae* (Rudow, 1886, *Phygadeuon*)

*nigriventris* (Thomson, 1896, *Mesocryptus*)

*victorovi* Jonaitis, 1981

***nivalis*** (Zetterstedt, 1838, *Cryptus*)

*opacus* (Taschenberg, 1865, *Cryptus*) preocc.

*borealis* (Thomson, 1883, *Microcryptus*)

*exannulatus* (Roman, 1909, *Microcryptus*) preocc.

***subguttatus*** (Gravenhorst, 1829, *Cryptus*)[[148]](#footnote-149) E I

*contracta* (Gravenhorst, 1829, *Cryptus*)

*punctata* (Ratzeburg, 1844, *Cryptus*)

*abscissa* (Ratzeburg, 1852, *Cryptus*)

*incerta* (Ratzeburg, 1852, *Cryptus*)

*silesiacus* (Habermehl, 1920, *Platylabus*) Horstmann (2006*a*)

*discedens* (Habermehl, 1929, *Platylabus*) Horstmann (2006*a*)

***PARMORTHA*** Townes, 1962

***parvula*** (Gravenhorst, 1829, *Cryptus*) E S I

*erythropus* (Gravenhorst, 1829, *Cryptus*)

***pleuralis*** (Thomson, 1873, *Cratocryptus*) E S I M

***PLECTOCRYPTUS*** Thomson, 1873

***albulatorius*** (Gravenhorst, 1829, *Cryptus*)

*hilarulus* Schmiedeknecht, 1905

***digitatus*** (Gmelin, 1790, *Ichneumon*) E S

*bivinctus* (Gravenhorst, 1829, *Cryptus*)

*poecilopus* (Rudow, 1883, *Cryptus*)

*niger* (Kiss, 1926, *Habrocryptus*)

***effeminatus*** (Gravenhorst, 1829, *Cryptus*) E

*flavopunctatus* (Bridgman, 1889, *Phygadeuon*)

*armatus* (Kriechbaumer, 1893, *Microcryptus*)

*clavatus* (Kriechbaumer, 1893, *Microcryptus*)

*sellatus* Ulbricht, 1911 unavailable

*lancifer* (Roman, 1925, *Acanthocryptus*)

***periculosus*** (Schmiedeknecht, 1905, *Microcryptus*) E

NMS, det. Schwarz, added here

***PLEOLOPHUS*** Townes, 1962

***basizonus*** (Gravenhorst, 1829, *Phygadeuon*) E S I

*larvincola* (Scharfenberg, 1805, *Ichneumon*) *nom. ob.*[[149]](#footnote-150)

*varicolor* (Gravenhorst, 1829, *Cryptus*)

*pteronum* (Hartig, 1838, *Phygadeuon*)

*commutatus* (Ratzeburg, 1848, *Phygadeuon*)

*obscurus* (Ulbricht, 1913, *Microcryptus*) unavailable

*nigrinus* (Fahringer, 1941, *Spilocryptus*)

***brachypterus*** (Gravenhorst, 1815, *Ichneumon*) E S W I M

*micropterus* misident.

*assimilis* (Förster, 1850, *Aptesis*)

*curtulus* (Kriechbaumer, 1891, *Microcryptus*)

*antennalis* (Kiss, 1924, *Habrocryptus*)

*hungaricus* (Kis, 1924, *Hemichneumon*

***sericans*** (Gravenhorst, 1829, *Phygadeuon*) E I

*pictus* (Gmelin, 1790, *Ichneumon*) preocc.

*eximius* (Habermehl, 1935, *Microcryptus*)

***vestigialis*** (Förster, 1850, *Aptesis*)

*aphyopterus* (Förster, 1850, *Aptesis*)

*formosus* (Förster, 1850, *Aptesis*)

*unifasciatus* (Schmiedeknecht, 1905, *Microcryptus*)

*alpinus* (Rudow, 1917, *Aptesis*)

*triangularis* (Kiss, 1924, *Microcryptus*)

*angustipetiolatus* (Ozols, 1934, *Microcryptus*)

*piceus* (Fahringer, 1935, *Stibeutes*)

***RHEMBOBIUS*** Förster, 1869[[150]](#footnote-151)

*ULOTHYMUS* Förster, 1869

*ACANTHOCRYPTUS* Thomson, 1873

***bifrons*** (Gmelin, 1790, *Ichneumon*)[[151]](#footnote-152) E S W I

*rufoniger* (Bridgman, 1889, *Phygadeuon*)

*minimus* (Lange, 1911, *Microcryptus*)

***perscrutator*** (Thunberg, 1824, *Ichneumon*) E S W I

Godfrey & Whitehead (2001)

*basalis* (Smith, 1874, *Cryptus*)

***quadrispinus*** (Gravenhorst, 1829, *Phygadeuon*) E S W I M

*ambiguus* (Berthoumieu, 1914, *Platylabus*)

*albicoxis* (Kiss, 1915, *Ryssolabus*)

*limnophilus* (Smits van Burgst, 1920, *Acanthocryptus*)

Horstmann (2000*a*)

*nigrobasicus* (Kiss, 1924, *Acanthocryptus*)

***SCHENKIA*** Förster, 1869

*ECPAGLUS* Förster, 1869

*SCHENCKIA* Dalla Torre, 1901

***graminicola*** (Gravenhorst, 1829, *Phygadeuon*) E S I

*brevicornis* (Gravenhorst, 1829, *Cryptus*)

*humilis* (Gravenhorst, 1829, *Cryptus*)

*alta* Jonaitis, 1981

***labralis*** (Gravenhorst, 1829, *Phygadeuon*) I

***spinolae*** (Gravenhorst, 1829, *Phygadeuon*)

Tribe PHYGADEUONTINI Förster, 1869[[152]](#footnote-153)

HEMITELINI Förster, 1869

GELINI Viereck, 1918

***ACLASTUS*** Förster, 1869

*DAETORA* Förster, 1869

*MICROPLEX* Förster, 1869

*OPISTHOSTENUS* Förster, 1869

*FETIALIS* Rossem, 1990 Broad (2004)

***borealis*** (Boheman, 1866, *Hemiteles*) S added by Horstmann (1980*b*)

*septentrionalis* (Holmgren, 1869, *Hemiteles*)

***eugracilis*** Horstmann, 1980 E S W added by Schwarz & Shaw (2000)

***flavipes*** Horstmann, 1980 E added by Horstmann (1980*b*)

***gracilis*** (Thomson, 1884, *Hemiteles*) E S W I M

*furcifer* Hellén, 1967

***micator*** (Gravenhorst, 1807, *Ichneumon*)[[153]](#footnote-154) E S W

*necator* misident.

*caudator* Hellén, 1967

***minutus*** (Bridgman, 1886, *Hemiteles*) E S I Anderson *et al.* (2006)

***pilosus*** Horstmann, 1980 E S W I added by Horstmann (1980*b*)

***solutus*** (Thomson, 1884, *Hemiteles*) E S W I

***transversalis*** Horstmann, 1980 E W added by Horstmann (1980*b*)

**sp. A** Horstmann, in prep. E S

**sp. B** Horstmann, in prep. E S I

***ACROLYTA*** Förster, 1869

*RHADINOCERA* Förster, 1869

***flagellator*** Schwarz & Shaw, 2000 S added by Schwarz & Shaw (2000)

***marginata*** (Bridgman, 1883, *Hemiteles*) E S

***nens*** (Hartig, 1838, *Hemiteles*) E S W I

*submarginata* (Bridgman, 1883, *Hemiteles*)

*rufizonata* (Schmiedeknecht, 1905, *Hemiteles*)

***okadai*** (Uchida, 1942, *Adiastola*) S[[154]](#footnote-155) added by Schwarz & Shaw (2000)

***pseudonens*** Schwarz & Shaw, 2000 E S added by Schwarz & Shaw (2000)

***rufocincta*** (Gravenhorst, 1829, *Hemiteles*)[[155]](#footnote-156) E S W

*distincta* (Bridgman, 1883, *Hemiteles*)

*capreolus* (Thomson, 1884, *Hemiteles*)

*quadrimaculata* (Lange, 1911, *Hemiteles*)

*obscurata* (Kiss, 1924, *Hemiteles*)

*unifasciata* (Kiss, 1924, *Hemiteles*)

***semistrigosa*** (Schmiedeknecht, 1897, *Hemiteles*) E

added by Schwarz & Shaw (2000)

***AGASTHENES*** Förster, 1869[[156]](#footnote-157)

*ASTHENOPTERA* Förster, 1869

***subarcticus*** (Jussila, 1965, *Hemiteles*) S I added by Horstmann (1998*a*)

***varitarsus*** (Gravenhorst, 1829, *Hemiteles*) E W I

*stagnalis* (Thomson, 1884, *Hemiteles*)

***AMPHIBULUS*** Kriechbaumer, 1893[[157]](#footnote-158)

***gracilis*** Kriechbaumer, 1893 E S I

*bispinus* (Thomson, 1894, *Cratocryptus*)

*aertsi* (Habermehl, 1926, *Stylocryptus*)

***AROTREPHES*** Townes, 1970[[158]](#footnote-159)

***laeviscutum*** Horstmann, 1993 E added by Horstmann (1993*b*)

***parvipennis*** (Thomson, 1884, *Phygadeuon*) E S

added by Horstmann (1993*b*)

***perfusor*** (Gravenhorst, 1829, *Cryptus*) E S I[[159]](#footnote-160)

*nitidus* (Bridgman, 1889, *Hemiteles*)

***speculator*** (Gravenhorst, 1829, *Phygadeuon*) E S I

***ATRACTODES*** Gravenhorst, 1829[[160]](#footnote-161)

subgenus ***ASYNCRITA*** Förster, 1876

***acuminator*** Roman, 1909 E added by Jussila (2001)

***albovinctus*** Haliday, 1837[[161]](#footnote-162) I

*mediatus* (Förster, 1876, *Asyncrita*)

***ambiguus*** Ruthe, 1859 E

*truncator* Roman, 1909

***angustipennis*** Förster, 1876 S I

*adversarius* Förster, 1876

*affinis* Förster, 1876

*cryptonastes* Förster, 1876

*gracilentus* Förster, 1876

*invalidus* Förster, 1876

*subdentatus* Förster, 1876

*vilis* Förster, 1876

*flavicoxa* Thomson, 1884

*thomsonii* Dalla Torre, 1902 preocc.

***assimilis*** Förster, 1876 S added by Jussila (2001)

*minusculus* Förster, 1876

*sordidus* Förster, 1876

*sponsus* Förster, 1876

***croceicornis*** Haliday, 1839 E S I M

*acceptus* Förster, 1876

*aemulator* Förster, 1876

*alticola* Förster, 1876

*atricornis* Förster, 1876

*contrarius* Förster, 1876

*designatus* Förster, 1876 preocc.

*difficilis* Förster, 1876

*distinctus* Förster, 1876

*engadinus* Förster, 1876

*exosus* Förster, 1876

*expertus* Förster, 1876

*fatalis* Förster, 1876

*inclinans* Förster, 1876

*infestus* Förster, 1876

*intemperans* Förster, 1876

*laboriosus* Förster, 1876

*minax* Förster, 1876

*modestus* Förster, 1876

*nodifer* Förster, 1876

*obsoletus* Förster, 1876

*placidus* Förster, 1876

*praepotens* Förster, 1876

*progenitus* Förster, 1876

*quaerulosus* Förster, 1876

*rapinatorius* Förster, 1876

*reconditus* Förster, 1876

*ruficinctus* Förster, 1876

*singularis* Förster, 1876

*solivagus* Förster, 1876

*sollicitator* Förster, 1876

*sulcatulus* Förster, 1876

*ultorius* Förster, 1876

*vanus* Förster, 1876

*vorax* Förster, 1876

*ruficornis* Brischke, 1880

*compressus* Thomson, 1884 preocc.

***cryptobius*** Förster, 1876 I added by Jussila (1979)

*eryptobius* misspelling

*carinatus* Förster, 1876

*conspicuus* Förster, 1876

*custoditor* Förster, 1876

*fulvicornis* Förster, 1876

*parallelus* Thomson, 1884

***cultellator*** Haliday, 1839 I

***exilis*** Haliday, 1839 E S I

*alpicola* (Förster, 1876, *Asyncrita*)

*angustulus* Förster, 1876

*delicatulus* Förster, 1876

*dispar* Förster, 1876

*flavicoxis* Förster, 1876

*longiventris* (Förster, 1876, *Asyncrita*)

*suspicax* Förster, 1876

*xanthocarpus* Förster, 1876

*alpicola* Strobl, 1901 preocc.

***exitialis*** Förster, 1876 S added by Jussila (2001)

*breviusculus* Förster, 1876

*callidus* Förster, 1876

*debilis* Förster, 1876

*difformis* Förster, 1876

*infimus* Förster, 1876

*parilis* Förster, 1876

*particeps* Förster, 1876

*perpusillus* Förster, 1876

***foveolatus*** Gravenhorst, 1829 E

*canaliculatus* (Hellén, 1944, *Asyncrita*)

***picipes*** Holmgren, 1860

*nigripes* Förster, 1876

***spiraculator*** Roman, 1918 S added by Jussila (2001)

subgenus ***ATRACTODES*** Gravenhorst, 1829

*ZETESIMA* Förster, 1876

***alpestris*** Roman, 1918 I added by Jussila (1979)

***arator*** Haliday, 1839 S I

*rufiventris* Strobl, 1901 preocc.

***bicolor*** Gravenhorst, 1829 S

*pygmaeator* (Zetterstedt, 1838, *Ichneumon*)

*alpigradus* Förster, 1876

*analogus* Förster, 1876

*cultrarius* Förster, 1876 preocc.

*destructor* Förster, 1876

*incommodus* Förster, 1876

*indigena* Förster, 1876

*lepidus* Förster, 1876

*mesoxanthus* Förster, 1876

*montivagus* Förster, 1876

*tenax* Förster, 1876

***citator*** Haliday, 1839 I

***fumatus*** Haliday, 1839 E S

*abnormis* Förster, 1876

*ambifarius* Förster, 1876

*avidus* Förster, 1876

*castus* Förster, 1876

*discolor* Förster, 1876

*dissidens* Förster, 1876

*ecarinatus* Förster, 1876

*homologus* Förster, 1876

*incongruens* Förster, 1876

*isomorphus* Förster, 1876

*melanocerus* Förster, 1876

*melanostomus* Förster, 1876

*nigrocoxis* Förster, 1876

*proprius* Förster, 1876

*separatus* Förster, 1876

*subdolus* Förster, 1876

*tenuicinctus* Förster, 1876

*tenuis* Förster, 1876

*unicinctus* Förster, 1876

***gilvipes*** Holmgren, 1860

***gravidus*** Gravenhorst, 1829 E

*fraternus* Förster, 1876

*areolaris* (Habermehl, 1909, *Exolytus*)

*archangelicae* Roman, 1913

*brevicornis* Bauer, 1958

***magnus*** Jussila, 2001 E Ely coll., det Jussila, added here

***obsoletor*** (Zetterstedt, 1838, *Porizon*) E W added by Jussila (1979)

*agilis* Förster, 1876

*declinis* Förster, 1876

*neophytus* Förster, 1876

*niger* Förster, 1876 preocc.

*foersteri* Dalla Torre, 1901

***pauxillus*** Förster, 1876 E S I

*montanus* Förster, 1876

*breviscapus* Thomson, 1884

***pusillus*** Förster, 1876 E S I

*calceatus* Förster, 1876

*linearis* Förster, 1876

*tenellus* Förster, 1876

*liogaster* Förster, 1876

*pernitens* Kokujev, 1909

***tenuipes*** Thomson, 1884 E S added by Bass & Cooling (1983)

***townesi*** Jussila, 1983 S added by Jussila (2001)

*thomsoni* Jussila, 1979 preocc.

subgenus ***CYCLAULATRACTODES*** Jussila, 1979

***helveticus*** Förster, 1876 I

*aequilongus* Förster, 1876

*oreophilus* Förster, 1876 Jussila (2001)

***punctator*** Roman, 1909 E S W added by Jussila (2001)

subgenus ***Hadratractodes*** Jussila, 1979

***vicinus*** Förster, 1876 S NMS, det. Jussila, added here

*absconditus* Förster, 1876

*cautior* Förster, 1876

*inquilinus* Förster, 1876

*intersectus* Förster, 1876 preocc.

*lentus* Förster, 1876

*rufipes* Förster, 1876 preocc.

*sectator* Förster, 1876

*venustulus* Förster, 1876

*crassicornis* Förster, 1876

*sarntheinii* Dalla Torre, 1901

subgenus ***RUGRATRACTODES*** Jussila, 1979

***alpinus*** Förster, 1876 S added by Jussila (2001)

*inimicus* Förster, 1876

***incrassator*** Roman, 1926 S added by Jussila (2001)

doubtfully placed species of *Atractodes*

[***dionaeus*** Haliday, 1837 nom. nud. E I Fitton (1976)]

[***piceicornis*** Haliday, 1837 nom. nud. I Fitton (1976)]

[***salius*** Haliday, 1837 nom. nud. I Fitton (1976)]

[***vestalis*** Haliday, 1837 nom. nud.]

***BATHYTHRIX*** Förster, 1869[[162]](#footnote-163)

*GAUSOCENTRUS* Förster, 1869

*ISCHNURGOPS* Förster, 1869

*PANARGYROPS* Förster, 1869

*STEGANOPS* Förster, 1869

*LEPTOCRYPTUS* Thomson, 1873

***aerea*** (Gravenhorst, 1829, *Cryptus*) E S W I

*brevis* (Thomson, 1884, *Leptocryptus*)

***alter*** (Kerrich, 1942, *Panargyrops*) E W

***argentata*** (Gravenhorst, 1829, *Hemiteles*) E

*lacustris* (Schmiedeknecht, 1905, *Leptocryptus*)

***claviger*** (Taschenberg, 1865, *Cryptus*) E S I

*atra* (Brischke, 1881, *Cryptus*)

***collaris*** (Thomson, 1896, *Leptocryptus*) E S

***decipiens*** (Gravenhorst, 1829, *Hemiteles*)[[163]](#footnote-164) E I M

*gyrini* (Parfitt, 1881, *Hemiteles*)

*signata* (Habermehl, 1919, *Leptocryptus*)

*meridionator* (Aubert, 1960, *Panargyrops*)

***formosa*** (Desvignes, 1860, *Hemiteles*)[[164]](#footnote-165) E

*albomarginata* (Kriechbaumer, 1892*, Leptocryptus*)

?*grandimacula* (Kriechbaumer, 1892*, Leptocryptus*)[[165]](#footnote-166) unavailable

*geniculosa* (Thomson, 1884, *Leptocryptus*)

***fragilis*** (Gravenhorst, 1829, *Hemiteles*) E S W

*bellula* (Kriechbaumer, 1892*, Leptocryptus*)

*urticarum* (Habermehl, 1920, *Leptocryptus*)

***lamina*** (Thomson, 1884, *Leptocryptus*) E S W I M

Kerrich (1942)

***linearis*** (Gravenhorst, 1829, *Nematopodius*) E

*heteropus* (Thomson, 1886, *Leptocryptus*)

***margaretae*** Sawoniewicz, 1980 E added by Schwarz & Shaw (2010)

***pellucidator*** (Gravenhorst, 1829, *Cryptus*) E S W I M

*ruficaudata* (Bridgman, 1883, *Hemiteles*)

***prominens*** (Strobl, 1901, *Leptocryptus*) E S W I M

added by Sawoniewicz (1980)

***rugulosa*** (Thomson, 1884, *Leptocryptus*) E S I

added by Sawoniewicz (1980)

***spheginus*** (Gravenhorst, 1829, *Mesoleptus*) E

added by Sawoniewicz (1980)

*sphecinus* (Schulz, 1906, *Mesoleptus*)

***strigosa*** (Thomson, 1884, *Leptocryptus*) E S added by Schwarz & Shaw (2010)

*ruficollis* (Habermehl, 1919, *Leptocryptus*)

***tenuis*** (Gravenhorst, 1829, *Cryptus*)

*rubens* (Kriechbaumer, 1892, *Leptocryptus*)

***thomsoni*** (Kerrich, 1942, *Thysiotorus*) E S W I M

*corsicator* (Aubert, 1961, *Panargyrops*)

***BLAPSIDOTES*** Förster, 1869

***vicinus*** (Gravenhorst, 1829, *Hemiteles*)[[166]](#footnote-167) E S I

*melanarius* (Gravenhorst, 1829, *Hemiteles*)

*pimplarius* (Berthoumieu, 1904, *Platylabus*)

***CEPHALOBARIS*** Kryger, 1915

***eskelundi*** Kryger, 1915 E added by Schwarz & Shaw (2011)

***CERATOPHYGADEUON*** Viereck, 1924

*EUROMONZIA* Aubert, 1965

***bellus*** (Gravenhorst, 1829, *Ichneumon*)[[167]](#footnote-168)

*longiceps* (Thomson, 1884, *Phygadeuon*)

***gracilicornis*** Horstmann, 1979 E added by Schwarz & Shaw (2011)

**?*parvicaudator*** (Aubert, 1965, *Remonzia*) E added by Schwarz & Shaw (2011)[[168]](#footnote-169)

***varicornis*** (Thomson, 1885, *Phygadeuon*) E added by Horstmann (1993*a*)

*maritimus* Horstmann, 1979 Horstmann (2001*d*)

***CHARITOPES*** Förster, 1869[[169]](#footnote-170)

*ADIASTOLA* Förster, 1869

***areolaris*** (Thomson, 1884, *Hemiteles*) E S

*brunneus* (Morley, 1907, *Hemiteles*)[[170]](#footnote-171)

***carri*** (Roman, 1923, *Cecidonomus*)[[171]](#footnote-172) E S M

*londinensis* (Morley, 1947, *Phygadeuon*)

*hemerobii* (Pfankuch, 1914, *Hemiteles*)

*pusillus* (Habermehl, 1920, *Hemiteles*) preocc.

***clausus*** (Thomson, 1888, *Hemiteles*) E S I M added by Townes (1983)

***gastricus*** (Holmgren, 1868, *Hemiteles*) E S W I

*chrysopae* (Brischke, 1890, *Hemiteles*)

*flavigaster* (Schmiedekecht, 1897, *Hemiteles*)

*flavocinctus* (Strobl, 1901, *Hemiteles*)

*brunnescens* (Schmiedekecht, 1905, *Hemiteles*)

*sylvicola* (Habermehl, 1920, *Hemiteles*)

***wesmaeliicida*** (Roman, 1934, *Hemiteles*) E S

***CHIROTICA*** Förster, 1869

*ALLOCOTA* Förster, 1869 preocc.

*DIAGLYPTA* Förster, 1869

*SPINOLIA* Förster, 1869 preocc.

*SYNECHES* Förster, 1869 preocc.

*DEUTEROSPINOLIA* Dalla Torre, 1902

***maculipennis*** (Gravenhorst, 1829, *Hemiteles*)[[172]](#footnote-173) E

*excellens* (Imhoff, 1850, *Hemiteles*)

*mulsantii* (Fonscolombe, 1852, *Hemiteles*)

*glyptonota* (Thomson, 1885, *Hemiteles*)

*schiefereri* (Strobl, 1904, *Hemiteles*)

***CLYPEOTELES*** Horstmann, 1974

***distans*** (Thomson, 1884, *Hemiteles*) E S[[173]](#footnote-174)

*rugifrons* (Thomson, 1884, *Hemiteles*)

*pseudorubiginosus* (Strobl, 1901, *Hemiteles*)

*xylonomoides* (Morley, 1907, *Cecidonomus*)

*fennicus* (Hellén, 1967, *Catalytus*)

***CREMNODES*** Förster, 1850

*CAENOMERIS* Förster, 1869

*STYGERA* Förster, 1869

*CREMNIAS* Roman, 1939

***atricapillus*** (Gravenhorst, 1815, *Icheumon*) E S W I

Horstmann (1993*b*)

*combustus* Förster, 1850

*nanodes* Förster, 1850

***costalis*** Horstmann, 1992 E S added by Horstmann (1992*a*)

***rufipes*** (Perkins, 1962, *Stygera*) E W Perkins (1962)

***DIAGLYPTIDEA*** Viereck, 1913

***conformis*** (Gmelin, 1790, *Ichneumon*) E S I M

*secernenda* (Schmiedeknecht, 1897, *Hemiteles*)

***DICHROGASTER*** Doumerc, 1855[[174]](#footnote-175)

*BRACHYCEPHALUS* Förster, 1869

*MICROTORUS* Förster, 1869

*OTACUSTES* Förster, 1869

*XENOBRACHYS* Förster, 1869

***aestivalis*** (Gravenhorst, 1829, *Hemiteles*) E S W I

*ruficollis* (Gravenhorst, 1829, *Hemiteles*)

*geniculata* (Thomson, 1884, *Hemiteles*)

***bischoffi*** (Schmiedeknecht, 1905, *Phygadeuon*) E

added by Schwarz & Shaw (2000)

*rufovaria* (Schmiedeknecht, 1905, *Phygadeuon*)

***genalis*** (Habermehl, 1925, *Phygadeuon*) E S I

added by Townes (1983)

*varsoviensis* (Sawoniewicz, 1978, *Ethelurgus*)

***heteropus*** (Thomson, 1896, *Phygadeuon*) E S

added by Horstmann (1992*a*)

*rufithorax* (Schmiedeknecht, 1932, *Phygadeuon*)

***liostylus*** (Thomson, 1885, *Hemiteles*) E S I

*schaffneri* (Schmiedeknecht, 1897, *Hemiteles*)

***longicaudata*** (Thomson, 1884, *Hemiteles*) E S

NMS, det. Schwarz, added here

*diatropus* Townes, 1983

***mandibularis*** Horstmann, 1973 E added by Horstmann (1992*a*)

***modesta*** (Gravenhorst, 1829, *Hemiteles*) E S W

added by Townes (1983)

*brunnea* (Kiss, 1924, *Herpestomus*)

***perlae*** (Doumerc, 1855, *Microgaster*) E added by Townes (1983)

***schimitscheki*** (Fahringer, 1935, *Phygadeuon*) E

added by Townes (1983)

*nigrithorax* Horstmann, 1976

***ENCRATEOLA*** Strand, 1917

*ENCRATES* Förster, 1869 preocc.

***glabra*** Horstmann, 1998 E added by Schwarz & Shaw (2000)

***laevigata*** (Ratzeburg, 1848, *Hemiteles*)[[175]](#footnote-176) E S W I M

*furcata* (Taschenberg, 1865, *Hemiteles*)

*subimpressa* (Brischke, 1892, *Hemiteles*)

***ENDASYS*** Förster, 1869[[176]](#footnote-177)

*BACHIA* Förster, 1869 preocc.

*SCINACOPUS* Förster, 1869

*STYLOCRYPTUS* Thomson, 1873

*BACHIANA* Strand, 1929

***alutaceus*** (Habermehl, 1912, *Stylocryptus*) E NHM, det. Sawoniewicz & Luhman, added here

*nigriventris* (Aerts, 1953, *Stylocryptus*)

***analis*** (Thomson, 1883, *Stylocryptus*) E NHM, det. Sawoniewicz & Luhman, added here

***anglianus*** Sawoniewicz & Luhman, 1992 E S W

added by Sawoniewicz & Luhman (1992)

***brevis*** (Gravenhorst, 1829, *Phygadeuon*) E W

***brunnulus*** Sawoniewicz & Luhman, 1992 E added by Sawoniewicz & Luhman (1992)

***erythrogaster*** (Gravenhorst, 1829, *Phygadeuon*)

*nigricoxis* (Habermehl, 1912, *Stylocryptus*)

***minutulus*** (Thomson, 1883, *Stylocryptus*) E W I

added by Sawoniewicz & Luhman (1992)

*nigripes* (Strobl, 1904, *Stylocryptus*)

*fusciventris* (Habermehl, 1916, *Stylocryptus*)

***parviventris*** (Gravenhorst, 1829, *Phygadeuon*) E

*pictipes* (Rudow, 1886, *Phygadeuon*)

*tyrolensis* (Schmiedeknecht, 1905, *Stylocryptus*)

***petiolus*** Sawoniewicz & Luhman, 1992 E added by Sawoniewicz & Luhman (1992)

***plagiator*** (Gravenhorst, 1829, *Phygadeuon*) E S M

added by Sawoniewicz & Luhman (1992)

*braunsi* (Lange, 1911, *Acanthocryptus*)

*laetus* (Habermehl, 1929, *Stylocryptus*)

***proteuryopsis*** Sawoniewicz & Luhman, 1992 E

added by Sawoniewicz & Luhman (1992)

***rusticus*** (Habermehl, 1912, *Stylocryptus*) E

***senilis*** (Gmelin, 1790, *Ichneumon*) E

***striatus*** (Kiss, 1924, *Acanthocryptus*) E added by Sawoniewicz & Luhman (1992)

***talitzkii*** (Telenga, 1961, *Phygadaeuon*) E NHM, det. Sawoniewicz & Luhman, added here

***testaceipes*** (Brischke, 1881, *Phygadeuon*) E added by Sawoniewicz & Luhman (1992)

*coxalis* (Schmiedeknecht, 1905, *Stylocryptus*)

***thunbergi*** Sawoniewicz & Luhman, 1992 E

*rubricator* (Thunberg, 1824, *Ichneumon*) preocc.

***transverseareolatus*** (Strobl, 1901, *Stylocryptus*)

***triannulatus*** Sawoniewicz & Luhman, 1992 E added by Sawoniewicz & Luhman (1992)

***varipes*** (Gravenhorst, 1829, *Phygadeuon*) E M

***ETHELURGUS*** Förster, 1869[[177]](#footnote-178)

*NUNECHES* Förster, 1869

*TOLMERUS* Förster, 1869 preocc.

*PLATYCRYPTUS* Kriechbaumer, 1893

***sodalis*** (Taschenberg, 1865, *Phygadeuon*) E S W I M

*pseudovulnerator* (Strobl, 1901, *Phygadeuon*)

*pici* (Berthoumieu, 1908, *Platylabus*)

*flavocinctus* (Habermehl, 1909, *Phygadeuon*)

*inermis* (Habermehl, 1919, *Phygadeuon*)

*niger* (Pfankuch, 1824, *Phygadeuon*) preocc. unavailable

***vulnerator*** (Gravenhorst, 1829, *Phygadeuon*) E S[[178]](#footnote-179)

***EUDELUS*** Förster, 1869

*CALLIPHRURUS* Förster, 1869

*IDEMUM* Förster, 1869

***pallicarpus*** (Thomson, 1884, *Hemiteles*)[[179]](#footnote-180) E

*pallidicarpus* (Dalla Torre, 1902, *Hemiteles*)

*crassiformis* (Viereck, 1917, *Hemiteles*) Schwarz & Shaw (2000)

***scabriculus*** (Thomson, 1884, *Hemiteles*)[[180]](#footnote-181) E

***simillimus*** (Taschenberg, 1865, *Hemiteles*)[[181]](#footnote-182) E S I

?*sericeus* (Rudow, 1886, *Hemiteles*)

?*albidus* (Pfankuch, 1925, *Hemiteles*)

?*meridionator* (Aubert, 1960, *Astomaspis*)

doubtfully placed species of *Eudelus*

***mediovittatus*** (Schmiedeknecht, 1897, *Hemiteles*)[[182]](#footnote-183) E

***FIANONIELLA*** Horstmann, 1992

***punctiscutum*** (Horstmann, 1990, *Odontoneura*) E

added by Horstmann (1990*b*)

***GELIS*** Thunberg, 1827

*PEZOMACHUS* Gravenhorst, 1829

*PEZOLOCHUS* Förster, 1850

*CATALYTUS* Förster, 1851

*HEMIMACHUS* Ratzeburg, 1852

*ALEGINA* Förster, 1869

*ASCHISTUS* Förster, 1869

*BARYDOTIRA* Förster, 1869

*ILAPINASTES* Förster, 1869

*PHILONYGMUS* Förster, 1869

*PLESIOMMA* Förster, 1869 preocc.

*RHADIURGUS* Förster, 1869 preocc.

*TERPIPHORA* Förster, 1869

*URITHEPTUS* Förster, 1869

*LEPTOGELIS* Ceballos, 1925

*FIANONIA* Seyrig, 1952

*HOLCOGELIS* Aubert, 1957

*ARCTODEUON* Hellén, 1967

*RHADIURGINUS* Hellén, 1967

***acarorum*** (Linnaeus, 1758, *Ichneumon*)

*nigricornis* (Retzius, 1783, *Ichneumon*)

*audax* (Förster, 1850, *Pezomachus*)

*cautus* (Förster, 1850, *Pezomachus*)

*circumcinctus* (Förster, 1850, *Pezomachus*)

*fraudulentus* (Förster, 1850, *Pezomachus*)

*integer* (Förster, 1850, *Pezomachus*)

*providus* (Förster, 1850, *Pezomachus*)

*sericeus* (Förster, 1850, *Pezomachus*)

*cruentatus* (Rudow, 1917, *Pezomachus*)

*fulvicornis* (Rudow, 1917, *Pezomachus*)

*unicinctus* (Rudow, 1917, *Pezomachus*)

*muscae* Pisica & Fabritius, 1986

***agilis*** (Fabricius, 1775, *Ichneumon*) E S W M

*cursor* (Schrank, 1780, *Ichneumon*)

*fuscicornis* (Retzius, 1783, *Ichneumon*)

*ruficornis* (Retzius, 1783, *Ichneumon*) Schwarz (2002)

*apterus* (Geoffroy, 1785, *Ichneumon*) preocc.

*celer* (Olivier, 1792, *Ichneumon*)

*instabilis* (Förster, 1850, *Pezomachus*)

*mediocris* (Förster, 1850, *Pezomachus*)

*thoracicus* (Brischke, 1878, *Pezomachus*)

*breviceps* (Thomson, 1884, *Pezomachus*)

*alpigena* (Strobl, 1901, *Pezomachus*)

*rossicus* (Szépligeti, 1901, *Pezomachus*)

*albulae* (Rudow, 1917, *Pezomachus*)

*cuculliae* (Rudow, 1917, *Pezomachus*)

*eupitheciae* (Rudow, 1917, *Pezomachus*)

*intrans* (Rudow, 1917, *Pezomachus*)

*lineatus* (Rudow, 1917, *Pezomachus*)

*microrum* (Rudow, 1917, *Pezomachus*)

*monozonius* (Rudow, 1917, *Pezomachus*) preocc.

*nigerrimus* (Rudow, 1917, *Pezomachus*) preocc.

*rosarum* (Rudow, 1917, *Pezomachus*)

*rufostictus* (Rudow, 1917, *Pezomachus*)

*vanessae* (Rudow, 1917, *Pezomachus*)

*leucurus* Ulbricht, 1926

*laricellae* (Fahringer, 1937, *Pezomachus*)

*cephalotes* Hellén, 1970

***albicinctoides*** Schwarz, 1998 E added by Schwarz (1998)

***albipalpus*** (Thomson, 1884, *Hemiteles*) E S I

*austriacus* (Fahringer, 1937, *Hemiteles*)

***albopilosus*** Schwarz, 2002 E added by Schwarz (2002)

***anthracinus*** (Förster, 1850, *Pezomachus*) E S I

*linearis* (Förster, 1851, *Pezomachus*)

*gonatopinus* (Thomson, 1884, *Pezomachus*)

***areator*** (Panzer, 1804, *Icneumon*) E S W I

*aberrans* (Gravenhorst, 1829, *Pezomachus*)

*orbiculatus* (Gravenhorst, 1829, *Hemiteles*)

*pulchellus* (Gravenhorst, 1829, *Hemiteles*)

*coelebs* (Ratzeburg, 1852, *Hemiteles*)

*variabilis* (Ratzeburg, 1852, *Hemiteles*) Horstmann (2001*d*)

*ephippium* (Rudow, 1886, *Hemimachus*)

*microgastri* (Rudow, 1886, *Hemiteles*)

*ruficollis* (Rudow, 1886, *Hemiteles*) preocc.

*cognatus* (Brischke, 1891, *Hemiteles*) Horstmann (2001*d*)

*minimus* (Glowacki, 1967, *Hemiteles*)

***avarus*** (Förster, 1850, *Pezomachus*) E S W I added by Schwarz & Shaw (1999)

***balteatus*** (Thomson, 1885, *Hemiteles*)[[183]](#footnote-184) E W

*brevistylus* (Hellén, 1967, *Charitopes*) preocc.

***bicolor*** (Villers, 1789, *Ichneumon*) E S

*alacer* (Förster, 1850, *Pezomachus*)

*brachyurus* (Förster, 1850, *Pezomachus*)

*distinctus* (Förster, 1850, *Pezomachus*)

*furtivus* (Förster, 1850, *Pezomachus*)

*incertus* (Förster, 1850, *Pezomachus*)

*molestus* (Förster, 1850, *Pezomachus*)

*muelleri* (Förster, 1850, *Pezomachus*)

*petulans* (Förster, 1850, *Pezomachus*)

*sordidus* (Förster, 1850, *Pezomachus*)

*spadiceus* (Förster, 1850, *Pezomachus*)

*timidus* (Förster, 1850, *Pezomachus*)

*vicinus* (Förster, 1850, *Pezomachus*)

*fusculus* (Förster, 1851, *Pezomachus*)

*rigii* (De Stefani, 1884, *Pezomachus*)

*facialis* (Brishcke, 1891, *Pezomachus*)

*riggioi* (Schmiedeknecht, 1906, *Pezomachus*)

*aphidicola* (Rudow, 1917, *Pezomachus*)

*formicarius* (Rudow, 1917, *Pezomachus*) preocc.

?*latus* Jonaitis, 1981

***brevis*** (Bridgman, 1883, *Pezomachus*)

***caudatulus*** Horstmann, 1997 S added by Schwarz & Shaw (1999)

*caudator* Horstmann, 1986 preocc.

***cayennator*** (Thunberg, 1824, *Ichneumon*) E S W

added by Schwarz & Shaw (1999)

*brassicae* Horstmann, 1986 Schwarz (2009)

*sulcatus* (Blunck, 1951, *Hemiteles*) preocc.

***cinctus*** (Linnaeus, 1758, *Ichneumon*) E S

*cinctor* (Thunberg, 1824, *Ichneumon*)

*bicolorinus* (Gravenhorst, 1829, *Hemiteles*)

***cursitans*** (Fabricius, 1775, *Ichneumon*)

*tuberculatus* (Hartig, 1838, *Pezomachus*)

*decipiens* (Förster, 1850, *Pezomachus*)

*peregrinator* (Förster, 1850, *Pezomachus*)

*alpinus* (Rudow, 1917, *Pezomachus*) preocc.

*braconidum* (Rudow, 1917, *Pezomachus*)

*helicis* (Rudow, 1917, *Pezomachus*)

*psychivorus* (Rudow, 1917, *Pezomachus*)

***curvicauda*** Horstmann, 1993 E added by Schwarz (1994)

***discedens*** (Förster, 1850, *Pezomachus*) E S W

*vagans* misident.

*calvus* (Förster, 1850, *Pezomachus*)

*quaesitorius* (Förster, 1850, *Pezomachus*)

*collaris* (Rudow, 1917, *Pezomachus*)

*exareolatus* (Rudow, 1917, *Pezomachus*) preocc.

*potentillae* (Rudow, 1917, *Pezomachus*)

*psychidum* (Rudow, 1917, *Pezomachus*)

*nigrithorax* (Habermehl, 1920, *Pezomachus*)

***divaricatus*** Horstmann, 1993 E W added by Horstmann (1993*a*)

***edentatus*** (Förster, 1850, *Pezomachus*) E

*imbellis* (Förster, 1850, *Pezomachus*)

*modestus* (Förster, 1850, *Pezomachus*)

*vagantiformis* (Bridgman, 1886, *Pezomachus*)

*dusmeti* Ceballos, 1925

***exareolatus*** (Förster, 1850, *Pezomachus*) E S

*nigritus* (Förster, 1850, *Pezomachus*)

*simulans* (Förster, 1850, *Pezomachus*)

*micromelas* (Kriechbaumer, 1894, *Phygadeuon*)

*lapponicus* Hellén, 1970

***falcatus*** Horstmann, 1986 S added by Horstmann (1986)

***fallax*** (Förster, 1850, *Pezomachus*) W I

*nigricornis* (Förster, 1850, *Pezomachus*) preocc.

*iglesiasi* Ceballos, 1925

***fasciitinctus*** (Dalla Torre, 1901, *Hemiteles*) E S

added by Schwarz & Shaw (1999)

*fasciipennis* (Brischke, 1881, *Hemiteles*) preocc.

***festinans*** (Fabricius, 1798, *Ichneumon*) E S W I

*nanus* (Förster, 1850, *Pezomachus*)

*pothumus* (Förster, 1850, *Pezomachus*)

*pumilus* (Förster, 1850, *Pezomachus*)

*tener* (Förster, 1850, *Pezomachus*)

*anguinus* (Förster, 1851, *Pezomachus*)

*ocissimus* (Förster, 1851, *Pezomachus*)

*brunneus* (Brischke, 1890, *Pezomachus*)

***formicarius*** (Linnaeus, 1758, *Mutilla*) E

*ratzeburgi* (Förster, 1850, *Pezomachus*)

*confusus* (Bridgman, 1883, *Hemimachus*) Schwarz & Shaw (1999)

*verrucosus* (Rudow, 1917, *Pezomachus*)

***forticornis*** (Förster, 1850, *Pezomachus*) E added by Schwarz (1998)

*manevali* Seyrig, 1927

***fuscicornis*** (Retzius, 1783, *Ichneumon*) E S added by Schwarz & Boriani (1994)

*longulus* (Zetterstedt, 1838, *Cryptus*) Schwarz (2002)

***hortensis*** (Christ, 1791, *Ichneumon*) E S W I

*acarorum* misident.

*callidus* (Förster, 1850, *Pezomachus*)

*canaliculatus* (Förster, 1850, *Pezomachus*)

*gentilis* (Förster, 1850, *Pezomachus*)

*impotens* (Förster, 1850, *Pezomachus*)

*inermis* (Förster, 1850, *Pezomachus*)

*latrator* (Förster, 1850, *Pezomachus*)

*lepidus* (Förster, 1850, *Pezomachus*)

*xylochophilus* (Förster, 1850, *Pezomachus*)

*avidus* (Förster, 1851, *Pezomachus*)

*filicornis* (Förster, 1851, *Pezomachus*)

*nomas* (Förster, 1851, *Pezomachus*)

*subtilis* (Förster, 1851, *Pezomachus*)

*albipennis* (Ratzeburg, 1852, *Hemiteles*)

***intermedius*** (Förster, 1850, *Pezomachus*) E reinstated by Schwarz & Shaw (1999)

*furax* (Förster, 1850, *Pezomachus*)

***kiesenwetteri*** (Förster, 1850, *Pezomachus*) E S I

*bellicosus* (Förster, 1850, *Pezomachus*)

*debeyii* (Förster, 1850, *Pezomachus*)

*egregius* (Förster, 1850, *Pezomachus*)

*costatus* (Bridgmanm, 1886, *Pezomachus*)

***limbatus*** (Gravenhorst, 1829, *Hemiteles*)[[184]](#footnote-185)

***liparae*** (Giraud, 1863, *Hemiteles*) E added by Horstmann (1986)

*ilicicola* (Seyrig, 1927, *Hemiteles*) Schwarz & Shaw (1999)

*ilicicolator* Aubert, 1966 Schwarz & Shaw (1999)

***longicauda*** (Thomson, 1884, *Hemiteles*) E S I

***lucidulus*** (Förster, 1850, *Pezomachus*)

*inquilinus* (Förster, 1850, *Pezomachus*)

*microstylus* (Förster, 1851, *Pezomachus*)

***mangeri*** (Gravenhorst, 1815, *Ichneumon*) E W

*fulveolatus* (Gravenhorst, 1829, *Pezomachus*)

*longipennis* (Gravenhorst, 1829, *Pezomachus*)

*foersteri* (Bridgman, 1882, *Aptesis*)

***meigenii*** (Förster, 1850, *Pezomachus*) E W S

*denudatus* (Förster, 1850, *Pezomachus*)

*geochares* (Förster, 1850, *Pezomachus*)

*insolens* (Förster, 1850, *Pezomachus*)

*?rufotinctus* (Bridgman, 1883, *Hemimachus*)[[185]](#footnote-186)

*noricus* (Strobl, 1901, *Pezomachus*)

*ephippium* (Rudow, 1914, *Pezomachus*) preocc.

*ephippium* (Rudow, 1917, *Pezomachus*) preocc.

***melanocephalus*** (Schrank, 1781, *Mutilla*) E S W I M

*fasciatus* (Fabricius, 1793, *Ichneumon*) preocc.

*fasciatus* (Ratzeburg, 1852, *Hemiteles*) preocc.

*hercyniae* (Rudow, 1917, *Pezomachus*)

***melanogaster*** (Thomson, 1884, *Hemiteles*)[[186]](#footnote-187) W

***melanophorus*** (Förster, 1851, *Pezomachus*) E S I

added by Schwarz & Shaw (1999)

*fuscicornis* (Förster, 1850, *Pezomachus*) preocc.

Schwarz & Shaw (1999)

*foersteri* (Bridgman 1886, *Pezomachus*) preocc.

Schwarz & Shaw (1999)

***micrurus*** (Förster, 1850, *Pezomachus*) E S W I

*pardosae* (Giard, 1895, *Hemiteles*)

***mitis*** Schwarz, 1994 E added by Schwarz (1994)

***mutillatus*** (Gmelin, 1790, *Ichneumon*)

*mutillarius* (Fabricius, 1787, *Ichneumon*) preocc.

*vagans* (Olivier, 1792, *Ichneumon*)

*pedicularius* (Fabricius, 1793, *Ichneumon*)

***nigritulus*** (Zetterstedt, 1838, *Cryptus*) E S

*terebrator* (Ratzeburg, 1848, *Pezomachus*)

***nitidus*** Horstmann, 1986 E added by Horstmann (1986)

***obscuripes*** Horstmann, 1986 E S W added by Horstmann (1986)

***papaveris*** (Förster, 1856 *Pezomachus*)

*hieracii* (Bridgman, 1883, *Pezomachus*)

*grandiceps* (Thomson, 1884, *Pezomachus*)

***problemator*** Aubert, 1989 E S added by Schwarz (1994)

***proximus*** (Förster, 1850, *Pezomachus*) E S W I M

*analis* (Förster, 1850, *Pezomachus*)

*attentus* (Förster, 1850, *Pezomachus*)

*celer* (Förster, 1850, *Pezomachus*)

*consociatus* (Förster, 1850, *Pezomachus*)

*corruptor* (Förster, 1850, *Pezomachus*)

*derasus* (Förster, 1850, *Pezomachus*)

*dubitator* (Förster, 1850, *Pezomachus*)

*ephippiger* (Förster, 1850, *Pezomachus*)

*faunus* (Förster, 1850, *Pezomachus*)

*hostilis* (Förster, 1850, *Pezomachus*)

*incubitor* (Förster, 1850, *Pezomachus*)

*latro* (Förster, 1850, *Pezomachus*)

*ochraceus* (Förster, 1850, *Pezomachus*)

*parvulus* (Förster, 1850, *Pezomachus*)

*sedulus* (Förster, 1850, *Pezomachus*)

*tonsus* (Förster, 1850, *Pezomachus*)

*vigil* (Förster, 1850, *Pezomachus*)

*vorax* (Förster, 1850, *Pezomachus*)

*xenoctonus* (Förster, 1850, *Pezomachus*)

*ageletes* (Förster, 1851, *Pezomachus*)

*ambulans* (Förster, 1851, *Pezomachus*)

*conveniens* (Förster, 1851, *Pezomachus*)

*decurtatus* (Förster, 1851, *Pezomachus*)

*dysalotus* (Förster, 1851, *Pezomachus*)

*elaphrus* (Förster, 1851, *Pezomachus*)

*erythropus* (Förster, 1851, *Pezomachus*)

*fugitivus* (Förster, 1851, *Pezomachus*)

*heydeni* (Förster, 1851, *Pezomachus*)

*histrio* (Förster, 1851, *Pezomachus*)

*imbecillus* (Förster, 1851, *Pezomachus*)

*indagator* (Förster, 1851, *Pezomachus*)

*indigator* misspelling

*insidiosus* (Förster, 1851, *Pezomachus*)

*inspector* (Förster, 1851, *Pezomachus*)

*lustrator* (Förster, 1851, *Pezomachus*)

*migrator* (Förster, 1851, *Pezomachus*)

*navus* (Förster, 1851, *Pezomachus*)

*procursorius* (Förster, 1851, *Pezomachus*)

*prudens* (Förster, 1851, *Pezomachus*)

*secretus* (Förster, 1851, *Pezomachus*)

*tentator* (Förster, 1851, *Pezomachus*)

*versatilis* (Förster, 1851, *Pezomachus*)

*violentus* (Förster, 1851, *Pezomachus*)

*hyponomeutae* (Bridgman, 1883, *Hemimachus*)

*ovatus* (Bridgman, 1883, *Hemimachus*) Schwarz & Shaw (1999)

*rufipes* (Bridgman, 1883, *Hemimachus*) preocc.

Schwarz & Shaw (1999)

*tricinctus* (Brischke, 1891, *Pezomachus*)

*evanescens* (Kriechbaumer, 1891, *Pezomachus*) unavailable

*rufiventris* (Kriechbaumer, 1891, *Pezomachus*) unavailable

*sesquifasciatus* (Kriechbaumer, 1891, *Pezomachus*)

?*alpinus* (Strobl, 1901, *Pezomachus*)

*transsylvanicus* (Kiss, 1915, *Pezomachus*)

*borealis* (Rudow, 1917, *Pezomachus*)

*retiniae* (Rudow, 1917, *Pezomachus*)

*versicolor* (Rudow, 1917, *Pezomachus*)

*parisiensis* Aubert, 1957

*inflatipes* Hellén, 1970

***pulicarius*** (Fabricius, 1793, *Ichneumon*)

*hoffmannseggii* (Gravenhorst, 1815, *Ichneumon*)

***recens*** Schwarz, 2002 E added by Schwarz (2002)

***rufipes*** (Förster, 1850, *Pezolochus*) E I added by Schwarz & Shaw (1999)

*aries* (Förster, 1850, *Pezomachus*)

*ecarinatus* (Förster, 1850, *Pezomachus*)

***rufogaster*** Thunberg, 1827 E S W I

*aemulus* (Förster, 1850, *Pezomachus*)

*alienus* (Förster, 1850, *Pezomachus*)

*anceps* (Förster, 1850, *Pezomachus*)

*astutus* (Förster, 1850, *Pezomachus*)

*bicinctus* (Förster, 1850, *Pezomachus*)

*carnifex* (Förster, 1850, *Pezomachus*)

*consobrinus* (Förster, 1850, *Pezomachus*)

*currens* (Förster, 1850, *Pezomachus*)

*debilis* (Förster, 1850, *Pezomachus*)

*detritus* (Förster, 1850, *Pezomachus*)

*emarcidus* (Förster, 1850, *Pezomachus*)

*flavipes* (Förster, 1850, *Pezomachus*)

*gracilis* (Förster, 1850, *Pezomachus*)

*helvolus* (Förster, 1850, *Pezomachus*)

*immaturus* (Förster, 1850, *Pezomachus*)

*insectator* (Förster, 1850, *Pezomachus*)

*juvenilis* (Förster, 1850, *Pezomachus*)

*languidus* (Förster, 1850, *Pezomachus*)

*lividus* (Förster, 1850, *Pezomachus*)

*lugubris* (Förster, 1850, *Pezomachus*)

*lutescens* (Förster, 1850, *Pezomachus*)

*puberulus* (Förster, 1850, *Pezomachus*)

*puerilis* (Förster, 1850, *Pezomachus*)

*pulcher* (Förster, 1850, *Pezomachus*)

*pulex* (Förster, 1850, *Pezomachus*)

*rufulus* (Förster, 1850, *Pezomachus*)

*scitulus* (Förster, 1850, *Pezomachus*)

*squalidus* (Förster, 1850, *Pezomachus*)

*unicolor* (Förster, 1850, *Pezomachus*)

*venustus* (Förster, 1850, *Pezomachus*)

*annulicornis* (Bridgman, 1883, *Hemimachus*)[[187]](#footnote-188)

*areneicola* (Rudow, 1914, *Pezomachus*)

*aphidum* (Rudow, 1917, *Pezomachus*)

*areneicolus* (Rudow, 1917, *Pezomachus*) preocc.

*balteatus* (Rudow, 1917, *Pezomachus*) preocc.

*isabellinus* (Rudow, 1917, *Pezomachus*)

*pemphigicola* (Rudow, 1917, *Pezomachus*)

*pieridis* (Rudow, 1917, *Pezomachus*)

*ulmicola* (Rudow, 1917, *Pezomachus*)

***rugifer*** (Thomson, 1884, *Hemiteles*) E S W

***seyrigi*** Ceballos, 1925 E added by Schwarz (1998)

***spinula*** (Thomson, 1884, *Pezomachus*) E S I

***spurius*** (Förster, 1850, *Pezomachus*) E S

*ruficornis* misident.[[188]](#footnote-189)

***terribilis*** Schwarz, 2002 E S added by Schwarz (2002)

***thomsoni*** (Schmiedeknecht, 1933, *Hemiteles*)[[189]](#footnote-190) E

*dispar* (Thomson, 1885, *Hemiteles*) preocc.

***trux*** (Förster, 1850, *Pezomachus*) E W

*ruficornis* Thunberg, 1827 preocc.

*blandus* (Förster, 1850, *Pezomachus*)

*comes* (Förster, 1850, *Pezomachus*)

*transfuga* (Förster, 1850, *Pezomachus*)

***viduus*** (Förster, 1850, *Pezomachus*) E S W I

*congruus* (Förster, 1850, *Pezomachus*)

*doliopus* (Förster, 1851, *Pezomachus*)

*mandibularis* (Thomson, 1884, *Pezomachus*)

***vulnerans*** (Förster, 1850, *Pezomachus*)

*affinis* (Magretti, 1884, *Pezomachus*)

***zeirapherator*** (Aubert, 1966, *Alegina*) S added by Schwarz & Shaw (1999)

species of *Gelis* excluded from the British list by Schwarz & Shaw (1999)

[***alpivagus*** (Strobl, 1901, *Hemiteles*) misident.]

[***stevenii*** (Gravenhorst, 1829, *Pezomachus*) misident.]

[***taschenbergii*** (Schmiedeknecht, 1897, *Hemiteles*) misident.[[190]](#footnote-191)]

***GLYPHICNEMIS*** Förster, 1869[[191]](#footnote-192)

*GNATHOCRYPTUS* Thomson, 1873

***atrata*** (Strobl, 1901, *Stylocryptus*) E S W

*alpina* (Strobl, 1901, *Stylocryptus*)

*suffolciensis* Morley, 1907

***clypealis*** (Thomson, 1883, *Stylocryptus*) E

***profligator*** (Fabricius, 1775, *Ichneumon*) E S W I M

*abdominalis* (Geoffroy, 1785, *Ichneumon*)

*nigricornis* (Gmelin, 1790, *Ichneumon*) preocc.

*textor* (Thunberg, 1824, *Ichneumon*)

*frequentoria* (Zetterstedt, 1838, *Ichneumon*)

*pygmaea* (Habermehl, 1916, *Stylocryptus*)

*ruficoxis* (Habermehl, 1916, *Stylocryptus*) preocc.

***vagabunda*** (Gravenhorst, 1829, *Phygadeuon*) E

*podagrica* (Gravenhorst, 1829, *Phygadeuon*)

*exannulata* (Hedwig, 1956, *Stylocryptus*)

***GNOTUS*** Förster, 1869

***chionops*** (Gravenhorst, 1829, *Hemiteles*) E S

*scutellator* (Lange, 1911, *Hemiteles*)

***macrurus*** (Thomson, 1884, *Hemiteles*)[[192]](#footnote-193) S

***rugipectus*** (Thomson, 1886, *Phygadeuon*)[[193]](#footnote-194)

***tenuipes*** (Gravenhorst, 1829, *Phygadeuon*) E

*tenuicornis* (Gravenhorst, 1829, *Hemiteles*)

*nebulosus* (Rudow, 1886, *Hemiteles*)

*cryptiformis* (Kiss, 1924, *Hemiteles*)

*nigripes* (Bauer, 1958, *Panargyrops*)

***GNYPETOMORPHA*** Förster, 1869

*TRISACRA* Förster, 1869

*VICTOROVIA* Tobias, 1963

***obscura*** (Bridgman, 1883, *Hemiteles*) E S

*aperta* (Thomson, 1884, *Hemiteles*)

***tubertae*** Horstmann, 2012 E added by Horstmann (2012*a*)

***GRASSEITELES*** Aubert, 1965

*DIAGLYPTELLANA* Horstmann, 1976 Schwarz (2005)

***opaculus*** (Thomson, 1884, *Hemiteles*) E S[[194]](#footnote-195)

***punctus*** (Holmgren, 1857, *Adelognathus*) E S added by Schwarz & Shaw (2000)

*sisyphii* (Verhoeff, 1891, *Hemiteles*)

*punctata* Horstmann, 1986 Schwarz & Shaw (2000)

***HEMITELES*** Gravenhorst, 1829[[195]](#footnote-196)

*OCYMORUS* Förster, 1869

***bipunctator*** (Thunberg, 1824, *Ichneumon*) E S I

*cingulator* Gravenhorst, 1829

*tristator* Gravenhorst, 1829

*rufipleuris* Szépligeti, 1901

***maricesca*** Schwarz & Shaw, 2000 E S W added by Schwarz & Shaw (2000)

***rubropleuralis*** Kiss, 1929 S added by Schwarz & Shaw (2000)

***similis*** (Gmelin, 1790, *Ichneumon*) E S W I M

*?debellator* (Schrank, 1781, *Ichneumon*) preocc.

*meridionalis* Gravenhorst, 1829

*unicolor* Thomson, 1884

doubtfully placed species of *Hemiteles*

[***liambus*** Thomson, 1885 nom. dub.]

[***piceus*** (Bridgman, 1883, *Hemimachus*) nom. dub. E

Fitton (1976)]

***HOLCOMASTRUS*** Horstmann, 2012

***bituberculatus*** (Schmiedeknecht, 1905, *Hemiteles*) E

NHM, det. Broad, added here

***ISADELPHUS*** Förster, 1869[[196]](#footnote-197)

*PEROSIS* Förster, 1869

*CECIDONOMUS* Bridgman, 1880

***armatus*** (Gravenhorst, 1829, *Echthrus*) E W

*mandibulator* (Dufour & Perris, 1840, *Anomalon*)

*bidentulus* (Thomson, 1844, *Hemiteles*)

***coriarius*** (Taschenberg, 1865, *Hemiteles*)

*rixator* (Woldstedt, 1877, *Polyblastus*)

*trochanteratus* (Strobl, 1903, *Erromenus*) Horstmann (1999*a*)

*carbonarius* (Schmiedeknecht, 1905, *Hemiteles*)

***gallicola*** (Bridgman, 1880, *Cecidonomus*) E S

*nigriventris* (Thomson, 1884, *Hemiteles*)

*carpathicus* (Kiss, 1924, *Lissonota*)

***inimicus*** (Gravenhorst, 1829, *Hemiteles*) E S W I M

*rufus* (Bridgman, 1880, *Cecidonomus*)

*obscuripes* (Thomson, 1884, *Hemiteles*)

***longisetosus*** (Schmiedeknecht, 1897, Hemiteles)[[197]](#footnote-198) E S

added by Horstmann (2009*c*)

***minutus*** Horstmann, 2009 E S added by Horstmann (2009*c*)

***LEPTOCRYPTOIDES*** Horstmann, 1976

***clavipes*** (Thomson, 1888, *Leptocryptus*) E I added by Schwarz & Shaw (2011)

***LOCHETICA*** Kriechbaumer, 1892[[198]](#footnote-199)

***westoni*** (Bridgman, 1880, *Cecidonomus*) E S

*pimplaria* (Thomson, 1884, *Phygadeuon*) Townes (1983)

***LYSIBIA*** Förster, 1869[[199]](#footnote-200)

*PEMON* Förster, 1869

*STIBOSCOPUS* Förster, 1869

*HAPLASPIS* Townes, 1944

***ceylonensis*** (Kerrich, 1956, *Haplaspis*) E

*proxima* (Perkins, 1962, *Pemon*)

***nanus*** (Gravenhorst, 1829, *Tryphon*) E S W I M

*fulvipes* (Gravenhorst, 1829, *Hemiteles*)

*socialis* (Ratzeburg, 1844, *Hemiteles*)

*populnea* (Boie, 1855, *Hemiteles*)

***tenax*** Townes, 1983 S I added by Townes (1983)

***MASTRULUS*** Horstmann, 1978

***marshalli*** (Bridgman & Fitch, 1882, *Phygadeuon*)[[200]](#footnote-201) E S

*marshalli* (Bridgman, 1883, *Phygadeuon*) preocc.

*capra* (Thomson, 1884, *Hemiteles*)

*disputabilis* (Schmiedeknecht, 1897, *Hemiteles*)

***MASTRUS*** Förster, 1869[[201]](#footnote-202)

*AENOPLEX* Förster, 1869

*DAICTES* Förster, 1869

***albobasalis*** (Schmiedeknecht, 1933, *Hemiteles*) E

added by Schwarz & Shaw (2010)

***boreaphilus*** (Roman, 1939, *Cecidonomus*) S added by Schwarz & Shaw (2010)

***costalis*** (Thomson, 1884, *Hemiteles*) E S added by Schwarz & Shaw (2010)

***deminuens*** (Hartig, 1838, *Hemiteles*) E S W I

*castaneus* (Taschenberg, 1865, *Hemiteles*)

*bredensis* (Smits van Burgst, 1913, *Hemiteles*)

*ripicola* (Habermehl, 1920, *Hemiteles*)

***fumipennis*** (Thomson, 1884, *Hemiteles*)[[202]](#footnote-203) I

***longicauda*** Horstmann, 1990 E NHM, det. Broad & Horstmann, added here

***longulus*** Horstmann, 1990 E added by Horstmann (1990*b*)

***mandibularis*** Horstmann, 1990 E added by Horstmann (1990*b*)

***parviceps*** (Hellén, 1967, *Isadelphus*) S added by Schwarz & Shaw (2010)

***ridibundus*** (Gravenhorst, 1829, *Hemiteles*)[[203]](#footnote-204) E I

**?*rufobasalis*** (Habermehl, 1920, *Hemiteles*) M

added by Schwarz & Shaw (2010)[[204]](#footnote-205)

***rufulus*** (Thomson, 1884, *Hemiteles*)[[205]](#footnote-206) E I

*nigrobasalis* (Schmiedeknecht, 1905, *Hemiteles*)

*rusticus* (Habermehl, 1920, *Hemiteles*)

*nigricoxis* (Hedwig, 1959, *Phygadeuon*) unavailable

***silbernageli*** (Kiss, 1929, *Hemiteles*) E S W added by Schwarz & Shaw (2010)

*leptocryptoides* (Schmiedeknecht, 1933, *Hemiteles*)

***sordipes*** (Gravenhorst, 1829, *Hemiteles*)[[206]](#footnote-207) E S

*karpinskii* (Glowacki, 1967, *Hemiteles*)

***tenuicosta*** (Thomson, 1884, *Phygadeuon*) E S

added by Schwarz & Shaw (2010)

***varicoxis*** (Taschenberg, 1865, *Hemiteles*)[[207]](#footnote-208) E S I

?*coactus* (Ratzeburg, 1852, *Hemiteles*)

*tricoloripes* (Schmiedeknecht, 1932, *Hemiteles*)

Species excluded from the British and Irish list

[***pictipes*** (Gravenhorst, 1829, *Hemiteles*)[[208]](#footnote-209)]

***MEDOPHRON*** Förster, 1869[[209]](#footnote-210)

*BARYNTICA* Förster, 1869

*HEDYLUS* Förster, 1869[[210]](#footnote-211)

*SUBHEMITELES* Horstmann, 1976 Horstmann (1998*a*)

***afflictor*** (Gravenhorst, 1829, *Phygadeuon*) W

*nigritus* (Gravenhorst, 1829, *Phygadeuon*)

*niger* Brischke, 1881

***armatulus*** (Thomson, 1888, *Phygadeuon*) E S M

added by Schwarz & Shaw (2010)

***crassicornis*** (Gravenhorst, 1829, *Hemiteles*)

***mixtus*** (Bridgman, 1883, *Hemiteles*) E I

*flavipes* (Thomson, 1888, *Phygadeuon*) invalid

*mandibularis* (Brischke, 1891, *Phygadeuon*) preocc.

*flavitarsis* (Dalla Torre, 1901, *Phygadeuon*)

***nigriceps*** (Thomson, 1883, *Acanthocryptus*) E

*elegans* (Schmiedeknecht, 1932, *Stylocryptus*)

***nitidus*** (Horstmann, 1976, *Subhemiteles*) E added by Horstmann (1998*a*)

***recurvus*** (Thomson, 1884, *Phygadeuon*) E S added by Schwarz & Shaw (2010)

***setosus*** (Hellén, 1967, *Aclastus*) E S added by Townes (1983)

*crassicornis* Ashmead, 1899 preocc.

***MEGACARA*** Townes, 1970[[211]](#footnote-212)

***hortulana*** (Gravenhorst, 1829, *Cryptus*) E S W I

*postica* (Wollaston, 1858, *Hemiteles*)

*rusticellae* (Bridgman, 1886, *Phygadeuon*)

*hispanator* (Aubert, 1968, *Phygadeuon*)

***vagans*** (Gravenhorst, 1829, *Phygadeuon*) E S W I

*apicalis* (Gravenhorst, 1829, *Cryptus*)

*alteareolata* (Schmiedeknecht, 1905, *Phygadeuon*)

*nova* (Kiss, 1929, *Acanthocryptus*)

***MESOLEPTUS*** Gravenhorst, 1829[[212]](#footnote-213)

*EXOLYTUS* Holmgren, 1859

***congener*** (Förster, 1876, *Exolytus*) E S W I added by Jussila *et al.* (2010)

***devotus*** (Förster, 1876, *Exolytus*) E

*fulvipes* (Förster, 1876, *Exolytus*)

*secretus* (Förster, 1876, *Exolytus*)

*sollicitus* (Förster, 1876, *Exolytus*)

***distinctus*** (Förster, 1876, *Exolytus*) E S W added by Jussila *et al.* (2010)

*flavipes* (Thomson, 1884, *Atractodes*)

***incessor*** (Haliday, 1839, *Atractodes*) E S I

*scrutator* (Haliday, 1839, *Atractodes*)

*ambiguus* (Förster, 1876, *Exolytus*) Jussila *et al.* (2010)

*incertus* (Förster, 1876, *Exolytus*) Jussila *et al.* (2010)

*speculum* (Förster, 1876, *Exolytus*)

*marginatus* (Thomson, 1884, *Atractodes*)

*petiolaris* (Thomson, 1884, *Atractodes*) Jussila *et al.* (2010)

***laevigatus*** (Gravenhorst, 1820, *Ichneumon*) E

*transversor* (Thunberg, 1824, *Ichneumon*)

*aequalis* (Förster, 1876, *Exolytus*)

*transsylvanicus* (Kiss, 1924, *Exolytus*)

***laticinctus*** (Walker, 1874, *Mesostenus*) E S W

added by Jussila *et al.* (2010)

*angustulus* (Förster, 1876, *Exolytus*) Jussila *et al.* (2010)

*ruficoxatus* (Förster, 1876, *Exolytus*)[[213]](#footnote-214)

*filicornis* (Thomson, 1884, *Atractodes*) Jussila *et al.* (2010)

***pronus*** (Förster, 1876, *Exolytus*) E S W added by Jussila *et al.* (2010)

*consortius* (Förster, 1876, *Exolytus*)

***vigilatorius*** (Förster, 1876, *Exolytus*) E S W

*ripicola* (Thomson, 1884, *Atractodes*) Jussila *et al.* (2010)

doubtfully placed species of *Mesoleptus*

[***coarctatus*** (Gravenhorst, 1829, *Cryptus*) nom. dub.[[214]](#footnote-215)]

[***mirabilis*** Stephens, 1835 nom. dub. E Fitton (1976)]

[***speciosus*** Curtis, 1837 nom. dub.]

[***splendens*** Gravenhorst, 1829 nom. dub. Jussila *et al.* (2010)]

[***subcompressus*** Stephens, 1835 nom. dub. E

Fitton (1976)]

***MICROMONODON*** Förster, 1869[[215]](#footnote-216)

*HEMICRYPTUS* Kriechbaumer, 1893

***tener*** (Kriechbaumer, 1893, *Hemicryptus*) E added by Laurenne *et al.* (2006)

***NEOPIMPLA*** Ashmead, 1900

***aleiodesi*** Schwarz & Shaw, 2000 E added by Schwarz & Shaw (2000)

***OBISIPHAGA*** Morley, 1907

***stenoptera*** (Marshall, 1868, *Aptesis*) S W I

*longicauda* (Vollenhoven, 1873, *Aptesis*)

*similis* (Brischke, 1891, *Thysiotorus*) invalid

*dimidiatipennis* (Schmiedeknecht, 1905, *Hemiteles*)

*ineptipennis* (Speiser, 1908, *Hemiteles*)

***ODONTONEURA*** Förster, 1869[[216]](#footnote-217)

***annulicornis*** (Thomson, 1884, *Phygadeuon*)[[217]](#footnote-218) E S

*csikii* (Szépligeti, 1901, *Phygadeuon*)

*formosa* (Pfankuch, 1921, *Phygadeuon*)

**sp. A**[[218]](#footnote-219) S added by Schwarz & Shaw (2010)

***OECOTELMA*** Townes, 1970[[219]](#footnote-220)

**sp. indet** I

***ORTHIZEMA*** Förster, 1869[[220]](#footnote-221)

*NAETES* Förster, 1869

*PHYZELUS* Förster, 1869

***amabile*** (Hedwig, 1939, *Hemiteles*) E added by Horstmann (1993*b*)

***francescae*** Schwarz & Shaw, 2011 E S W added by Schwarz & Shaw (2011)

***graviceps*** (Marshall, 1868, *Aptesis*) E W

***hadrocerum*** (Thomson, 1884, *Hemiteles*) E S

*fasciatum* (Brischke, 1888, *Phyzelus*)

*rufum* (Brischke, 1892, *Hemiteles*)

***obscurum*** Horstmann, 1993 added by Horstmann (1993*b*)

***subannulatum*** (Bridgman, 1883, *Hemiteles*) E S

*maculipennis* (Rudow, 1886, *Hemiteles*) preocc.

*ornatum* (Brischke, 1890, *Hemiteles*)

***triannulatum*** (Thomson, 1884, *Hemiteles*)[[221]](#footnote-222) E S

***PHYGADEUON*** Gravenhorst, 1829[[222]](#footnote-223)

*APTEROPHYGAS* Förster, 1869

*BATHYMETIS* Förster, 1869

*ERNOCTONA* Förster, 1869

*GUNOPACHES* Förster, 1869

*HABROMMA* Förster, 1869

*HOMELYS* Förster, 1869

*ISELIX* Förster, 1869

*ISOCHRESTA* Förster, 1869a

*PANTOLISPA* Förster, 1869

*ZAPHLEGES* Förster, 1869

*ISCHNOCRYPTUS* Kriechbaumer, 1892

***acutipennis*** Thomson, 1884 E S

***atropos*** Kriechbaumer, 1892[[223]](#footnote-224) E added by Horstmann (2001*a*)

***brachyurus*** Thomson, 1884

***brevitarsis*** Thomson, 1884[[224]](#footnote-225) E added by Horstmann (2001*a*)

***canaliculatus*** Thomson, 1889

***cephalotes*** Gravenhorst, 1829

*transfuga* (Gravenhorst, 1829, *Ichneumon*)

***clotho*** Kriechbaumer, 1892 E S W added by Horstmann (1981*c*)

*grossae* Horstmann, 1981 Horstmann (2001*a*)

***clypearis*** Strobl, 1901 E added by Horstmann (2012*b*)

***cubiceps*** Thomson, 1884

***cylindraceus*** Ruthe, 1859 E S

*sudvoldensis* Morley, 1947

***detestator*** (Thunberg, 1824, *Ichneumon*)

***devonensis*** Morley, 1947[[225]](#footnote-226) E

***dimidiatus*** Thomson, 1884

*cylindricus* Brischke, 1891

**?*dromicus*** (Gravenhorst, 1815, *Ichneumon*)[[226]](#footnote-227)

***dubius*** (Gravenhorst, 1829, *Hemiteles*)

*scaposus* Thomson, 1884

***elegans*** (Förster, 1850, *Theroscopus*) E S W I

added by Horstmann (1993*b*)

*cingulatus* (Förster, 1850, *Theroscopus*)

*confusus* Hedwig, 1959 preocc.

***elliotti*** Morley, 1947 S

***exiguus*** Gravenhorst, 1829 E

*gallevensis* Morley, 1947

***flavimanus*** Gravenhorst, 1829 E

***forticornis*** Kriechbaumer, 1892 E

***fraternae*** Horstmann, 2001 S added by Horstmann (2001*a*)

***fumator*** Gravenhorst, 1829 E S

*lycaenae* Rudow, 1886

*britannicus* Habermehl, 1923 Horstmann (2000*c*)

*ragensis* Morley, 1947

***geniculatus*** Kriechbaumer, 1892

***gracilentus*** Horstmann, 1997 E S added by Horstmann (1993*b*)

*gracilicornis* Horstmann, 1993 preocc.

***hercynicus*** Gravenhorst, 1829 E

***infelix*** Dalla Torre, 1901

*inflatus* Thomson, 1884 invalid

***laeviventris*** Thomson, 1884 E

*compactus* Morley, 1947

***leucostigmus*** Gravenhorst, 1829 E

*punctigena* Thomson, 1884

***liosternus*** Thomson, 1886

***magnicornis*** (Thomson, 1884, *Hemiteles*)[[227]](#footnote-228)

***melanopygus*** (Gravenhorst, 1829, *Hemiteles*)[[228]](#footnote-229) E S M

*validicornis* (Thomson, 1884, *Hemiteles*)

*semicroceus* (Schmiedeknecht, 1897, *Hemiteles*)

***nanus*** (Gravenhorst, 1829, *Cryptus*)

***nigrifemur*** Horstmann, 2001 added by Horstmann (2001*a*)

***nitidus*** Gravenhorst, 1829

***ovaliformis*** Dalla Torre, 1901

*ovalis* Thomson, 1884 preocc.

***ovatus*** Gravenhorst, 1829

*caliginosus* Gravenhorst, 1829

*montanus* (Lange, 1911, *Stylocryptus*)

***pallicarpus*** Thomson, 1884 S I

*pallidicarpus* Dalla Torre, 1902

***palus*** Schwarz & Shaw, 2011 E W added by Schwarz & Shaw (2011)

***paradoxus*** (Bridgman, 1889, *Apterophygas*) E

*hungaricus* (Kiss, 1915, *Phygadeuon*)

*insulanus* Hedwig, 1939

***pegomyiae*** Habermehl, 1928

***punctiventris*** Thomson, 1884

***rotundipennis*** Thomson, 1884 E S W M

*differens* Hedwig, 1938

***rubricaudus*** Morley, 1947 E

***rugulosus*** Gravenhorst, 1829

*semipolitus* Taschenberg, 1865

***subtilis*** Gravenhorst, 1829 E

*flavicans* Thomson, 1884

*oppositus* Thomson, 1884

*subalpinus* Roman, 1909

*lincolniae* Morley, 1947

***surriensis*** Morley, 1947 E

***tenuiscapus*** Thomson, 1884

***thomsoni*** Roman, 1925 E added by Horstmann (2001*a*)

***trichops*** Thomson, 1884 S

*ocularis* Thomson, 1889

***troglodytes*** Gravenhorst, 1829

*anthracinus* Kriechbaumer, 1894

***variabilis*** Gravenhorst, 1829

*confinis* Smits van Burgst, 1912

***varicornis*** (Gravenhorst, 1829, *Hemiteles*)[[229]](#footnote-230)

***vexator*** (Thunberg, 1824, *Ichneumon*)[[230]](#footnote-231) E

*patellator* (Thunberg, 1824, *Ichneumon*)

*diaphanus* Gravenhorst, 1829

*minor* Fonscolombe, 1851

*nigripes* Aubert, 1959

***PLATYRHABDUS*** Townes, 1970[[231]](#footnote-232)

***clypeatus*** Horstmann, 1998 E S M added by Horstmann (1998*a*)

***inflatus*** (Thomson, 1884, *Hemiteles*) E S I M

*rufus* (Morley, 1907, *Aritranis*)

*elongatus* (Smits van Burgst, 1913, *Hemiteles*) preocc.

*tunisiae* (Morley, 1926, *Hemiteles*)

*tunetanus* (Schmiedeknecht, 1932, *Hemiteles*)

***monodon*** (Thomson, 1884, *Hemiteles*) E

*graciliventris* (Schmiedeknecht, 1933, *Hemiteles*)

***nervellator*** Horstmann, 1998 E added by Horstmann (1998*a*)

***PLEUROGYRUS*** Townes, 1970

***persector*** (Parfitt, 1882, *Hemiteles*) E Fitton (1976)

***pumilus*** (Hellén, 1967, *Uchidella*) E added by Horstmann (1995)

***POLYAULON*** Förster, 1869

*THAUMATOTYPUS* Förster, 1869 Horstmann (1998*a*)

*THAUMATOTYPIDEA* Viereck, 1912

*RHACODOPTERON* Čapek, 1956

***paradoxus*** (Zetterstedt, 1838, *Cryptus*) E S W

*billupsi* (Bridgman, 1882, *Thaumatotypus*)

*evertsi* (Smits van Burgst, 1912, *Thaumatotypus*)

***stiavnicensis*** (Čapek, 1956, *Rhacodopteron*) E

added by Schwarz & Shaw (2000)

***PYGOCRYPTUS*** Roman, 1925

***brevicornis*** (Brischke, 1881, *Macrocryptus*) E added by Townes (1983)

*grandis* (Thomson, 1884, *Phygadeuon*) Sawoniewicz (2003)

***STIBEUTES*** Förster, 1850[[232]](#footnote-233)

*CHAMAEZELUS* Förster, 1869

*SCHIZOPLEURON* Aubert, 1968

***blandi*** Schwarz & Shaw, 2011 S added by Shaw & Schwarz (2011)

***breviareolatus*** (Thomson, 1884, *Hemiteles*)[[233]](#footnote-234)

*rugiventris* (Strobl, 1901, *Acanthocryptus*) Horstmann (2000*a*)

***brevicornis*** (Lange, 1911, *Stilpnus*) E added by Horstmann (2010*b*)

***calderonae*** Bordera & Hernández-Rodríguez, 2004 E

added by Horstmann (2010*b*)

***curvispina*** (Thomson, 1884, *Phygadeuon*) E S W

***gravenhorstii*** Förster, 1850 E

***heinemanni*** Förster, 1850 E S

***heterogaster*** (Thomson, 1885, *Phygadeuon*) E S W M

added by Horstmann (2010*b*)

***intermedius*** Horstmann, 2010 E added by Horstmann (2010*b*)

***nigrinus*** Horstmann, 2010 E added by Horstmann (2010*b*)

***rozsypali*** (Gregor, 1941, *Phygadeuon*) E added by Horstmann (2010*b*)

***STILPNUS*** Gravenhorst, 1829[[234]](#footnote-235)

subgenus ***POLYRHEMBIA*** Förster, 1869

***tenebricosus*** (Gravenhorst, 1829, *Hemiteles*) E S W I

*nitidulator* (Zetterstedt, 1838, *Ichneumon*)

*vestalis* (Haliday, 1839, *Atractodes*)

*albicinctus* (Förster, 1876, *Polyrhembia*)

*anthracinus* (Förster, 1876, *Polyrhembia*)

*canaliculatus* (Förster, 1876, *Polyrhembia*) preocc.

*carbonarius* (Förster, 1876, *Polyrhembia*)

*corvinus* (Förster, 1876, *Polyrhembia*)

*discoloripes* (Förster, 1876, *Atractodes*)

*nigratus* (Förster, 1876, *Polyrhembia*)

*nigripes* (Förster, 1876, *Polyrhembia*)

*procerulus* (Förster, 1876, *Polyrhembia*)

*splendidus* (Förster, 1876, *Polyrhembia*)

*stygius* (Förster, 1876, *Polyrhembia*)

*rodnensis* Kiss, 1924 Horstmann (2011*a*)

subgenus ***STILPNUS*** Gravenhorst, 1829

***blandus*** Gravenhorst, 1829 E S W I

*assimilis* Förster, 1876

*callens* Förster, 1876

*cyclogaster* Förster, 1876

*fuscicornis* Förster, 1876

*pellucens* Förster, 1876

***crassicornis*** Thomson, 1884 E S I

***deplanatus*** Gravenhorst, 1829 E UM

***gagates*** (Gravenhorst, 1807, *Ichneumon*) E S W I

*aequilongus* Förster, 1876

*cyclodes* Förster, 1876

*denticulatus* Förster, 1876

*diffinis* Förster, 1876

*dimidiatus* Förster, 1876

*elimatus* Förster, 1876

*eurygaster* Förster, 1876

*fulvicornis* Förster, 1876

*gallicus* Förster, 1876

*robinsoni* Roman, 1920

***parvulus*** Förster, 1876 E added by Jussila (1987)

*inaequalis* Förster, 1876

***pavoniae*** (Scopoli, 1763, *Ichneumon*) E S W I

*agilis* Förster, 1876

*ambulatorius* Förster, 1876

*arridens* Förster, 1876

*conformatus* Förster, 1876

*declinis* Förster, 1876

*morionellus* Förster, 1876

*neglectus* Förster, 1876

*nigricoxis* Förster, 1876

*politus* Förster, 1876 preocc.

*retritus* Förster, 1876

*subtilis* Förster, 1876

*tersus* Förster, 1876

*trivialis* Förster, 1876

*unctus* Förster, 1876

*xanthopus* Förster, 1876

*angustatus* Thomson, 1884

***subzonulus*** Förster, 1876 E S W I

*canaliculatus* Förster, 1876

*diversus* Förster, 1876

*latens* Förster, 1876

*placitus* Förster, 1876

*tenuipes* Thomson, 1884

subgenus ***XESTOPHYES*** Förster, 1869

*XESTOPHYA* Förster, 1876

***dryadum*** Curtis, 1832 E I[[235]](#footnote-236)

***fallax*** (Förster, 1876, *Xestophya*) E NMS, det. Jussila, added here

***SULCARIUS*** Townes, 1970[[236]](#footnote-237)

***biannulatus*** (Gravenhorst, 1829, *Hemiteles*) S

***bispinosus*** (Rudow, 1886, *Phygadeuon*) E added by Townes (1983)

*hellbachi* (Schmiedeknecht, 1905, *Hemiteles*)

***fontinalis*** (Ruschka, 1926, *Hemiteles*) E S added by Schwarz & Shaw (2011)

***laevipleuris*** Horstmann, 1992 E added by Horstmann (1992*a*)

***nigricornis*** (Thomson, 1884, *Hemiteles*)[[237]](#footnote-238) E W S I

*homocerus* (Thomson, 1885, *Hemiteles*)

***nigridens*** Horstmann, 1992 E S added by Horstmann (1992*a*)

**sp. A**[[238]](#footnote-239) E S added by Schwarz & Shaw (2011)

***THAUMATOGELIS*** Schwarz, 1995

*THAUMATOGELIS* Schmiedeknecht, 1933 unavailable

*CRYPTOGELIS* Hellén, 1944 nom. nud.

***audax*** (Olivier, 1792, *Ichneumon*) E W

*arnearum* (Geoffroy, 1785, *Ichneumon*) preocc.

*zonatus* (Förster, 1850, *Pezomachus*)

***innoxius*** Schwarz, 2001 E added by Schwarz (2001)

*mingetshauricus* misident.[[239]](#footnote-240)

***lichtensteini*** (Pfankuch, 1913, *Thaumatotypidea*) E

added by Schwarz & Shaw (2000)

*cabrerai* (Duchaussoy, 1915, *Thaumatotypidea*)

*graecus* (Rudow, 1917, *Pezomachus*)

*siculus* (Rudow, 1917, *Pezomachus*)

?*maroccanus* (Ceballos, 1925, *Gelis*) Schwarz (2001)

*dentatus* (Seyrig, 1926, *Thaumatotypidea*)

*longicornis* (Seyrig, 1926, *Thaumatotypidea*)

*medianus* (Seyrig, 1926, *Thaumatotypidea*)

*micariae* (Seyrig, 1926, *Thaumatotypidea*) preocc.

*muticus* (Seyrig, 1926, *Thaumatotypidea*)

*nigripes* (Seyrig, 1926, *Thaumatotypidea*)

***neesii*** (Förster, 1850, *Pezomachus*) E S added by Schwarz & Shaw (2000)

*quadrifasciatus* (Kriechbaumer, 1899, *Pezomachus*) unavailable

***sylvicola*** (Förster, 1850, *Pezomachus*) E

*luceus* (Seyrig, 1928, *Gelis*)

***vulpinus*** (Gravenhorst, 1815, *Ichneumon*) E S W

*aquisgranensis* (Förster, 1850, *Pezomachus*)

species of *Thaumatogelis* excluded from the British list by Schwarz (1995)

[***pilosus*** (Capron, 1888, *Pezomachus*)[[240]](#footnote-241)]

***THEROSCOPUS*** Förster, 1850[[241]](#footnote-242)

*CHAMERPES* Förster, 1869

*ERIPLANUS* Förster, 1869

*PHYRTUS* Förster, 1869

*THYSIOTORUS* Förster, 1869

***bonelli*** (Gravenhorst, 1815, *Ichneumon*) E W added by Horstmann (1993*b*)

*ingrediens* Förster, 1850

*rufiventris* (Rudow, 1917, *Agrothereutes*)

***coriaceus*** Horstmann, 1993 E added by Horstmann (1993*b*)

***esenbeckii*** (Gravenhorst, 1815, *Ichneumon*)[[242]](#footnote-243) E S W I

*subzonatus* (Gravenhorst, 1815, *Ichneumon*)

*gravenhorstii* (Ratzeburg, 1844, *Pezomachus*)

*inaequalis* Förster, 1850

*transsylvanicus* (Kiss, 1929, *Hemiteles*)

***fasciatulus*** Horstmann, 1979[[243]](#footnote-244) E

*fasciatus* (Thomson, 1884, *Hemiteles*) preocc.

***hemipteron*** (Riche, 1791, *Ichneumon*) E S W I

*hemipterus* (Fabricius, 1793, *Ichneumon*)

*hemipterator* (Thunberg, 1824, *Ichneumon*)

*dissimilis* (Gravenhorst, 1829, *Hemiteles*)

*scrupulosus* (Gravenhorst, 1829, *Hemiteles*)

*brevipennis* (Brischke, 1891, *Thysiotorus*)

*nanopterus* (Kieffer, 1903, *Phygadeuon*)

*insignipennis* (Schmiedeknecht, 1905, *Hemiteles*)

*kandaviensis* (Ozols, 1934, *Hemiteles*)

***horsfieldi*** Schwarz & Shaw, 2011 S added by Schwarz & Shaw (2011)

***mariae*** Schwarz & Shaw, 2011 E S added by Schwarz & Shaw (2011)

***megacentrus*** (Schiødte, 1839, *Cryptus*) E S

*ornaticornis* (Schmiedeknecht, 1897, *Hemiteles*)

Horstmann (2004*b*)

*occisor* (Habermehl, 1923, *Phygadeuon*) Horstmann (2000*c*)

***naninae*** Schwarz & Shaw, 2011 S added by Schwarz & Shaw (2011)

***ochrogaster*** (Thomson, 1888, *Phygadeuon*) E S M

added by Schwarz & Shaw (2011)

*rotundator* Aubert, 1989 Schwarz & Shaw (2011)

***opacinotum*** (Hellén, 1967, *Aclastus*)[[244]](#footnote-245) added by Schwarz & Shaw (2011)

***pedestris*** (Fabricius, 1775, *Ichneumon*)

*pilosellus* (Rudow, 1917, *Pezomachus*)

***pullator*** (Gravenhorst, 1829, *Cryptus*)[[245]](#footnote-246) E S I

*notaulium* (Morley, 1947, *Phygadeuon*)

***rufulus*** (Gmelin, 1790, *Ichneumon*) E S W I

*micator* misident.

*luteiventris* (Gravenhorst, 1829, *Hemiteles*)

*oxyphymus* (Gravenhorst, 1829, *Hemiteles*)

*rufulus* (Gravenhorst, 1829, *Hemiteles*) preocc.

*litoreus* (Parfitt, 1882, *Hemiteles*)

*politus* (Bridgman, 1883, *Hemiteles*)

*silesiacus* (Habermehl, 1919, *Phygadeuon*)

***ungularis*** (Thomson, 1884, *Phygadeuon*)[[246]](#footnote-247) E

*ungularis* (Thomson, 1884, *Hemiteles*)

*heteroneurus* (Schmiedeknecht, 1933, *Hemiteles*)

***TRICHOLINUM*** Förster, 1869

*STIBOSCOPELLUS* Roman, 1930

***ischnocerum*** (Thomson, 1888, *Hemiteles*) E S W

added by Schwarz & Shaw (2011)

*pimploides* (Roman, 1930, *Stiboscopellus*)

***TROPISTES*** Gravenhorst, 1829

*PSEUDOLIMERODES* Strobl, 1902

*Boleslawia* Sawoniewicz, 1996 Schwarz & Shaw (2011)

***falcatus*** (Thomson, 1884, *Hemiteles*) E added by Schwarz & Shaw (2011)

*nigriventris* Kriechbaumer, 1894 preocc.

*rufipes* Kriechbaumer, 1894

*compressiventris* (Strobl, 1902, *Pseudolimerodes*)

***nitidipennis*** Gravenhorst, 1829 E S

*fuscipes* Kriechbaumer, 1894 unavailable

*nigriventris* Kriechbaumer, 1894 unavailable

***scoticus*** Schwarz & Shaw, 2011 E[[247]](#footnote-248) S added by Schwarz & Shaw (2011)

***Uchidella*** Townes, 1957

*ITAMUS* Förster, 1869 preocc.

***brevicauda*** Horstmann, 1993 E S added by Horstmann (1993*c*)

***flavilabris*** Horstmann, 1993 E S added by Schwarz & Shaw (2011)

***longicaudata*** Horstmann, 1997 E S I added by Schwarz & Shaw (2011)

*longicauda* Horstmann, 1993 preocc. Horstmann (1997)

***XENOLYTUS*** Förster, 1869[[248]](#footnote-249)

*STERNOCRYPTUS* Roman, 1925

***bitinctus*** (Gmelin, 1790, *Ichneumon*) E S

*expulsor* (Thunberg, 1824, *Ichneumon*)

***substriatus*** Townes, 1983 E added by Townes (1983)

***XIPHULCUS*** Townes, 1970

*NOTOSTILBUS* Townes, 1983

***floricolator*** (Gravenhorst, 1807, *Ichneumon*) E S[[249]](#footnote-250)

*imbecillus* (Gravenhorst, 1829, *Hemiteles*)

*longiventris* (Schiødte, 1839, *Hemiteles*) Horstmann (2004*b*)

*longulus* (Thomson, 1884, *Hemiteles*)

*lucidus* (Szépligeti, 1901, *Phygadeuon*)

*muelleri* (Kiss, 1924, *Hemiteles*)

*ramellosus* (Kiss, 1924, *Leptocryptus*)

*longicauda* Hellén, 1967, *Uchidella*)

***ZOOPHTHORUS*** Förster, 1869[[250]](#footnote-251)

*CHAETOMASTRUS* Hellén, 1967

***anglicanus*** (Morley, 1907, *Hemiteles*) E M

***bridgmani*** (Schmiedeknecht, 1897, *Hemiteles*) E S

*niger* (Bridgman, 1883, *Theroscopus*) invalid

*pfankuchi* (Smits van Burgst, 1913, *Hemiteles*)

***cynipinus*** (Thomson, 1884, *Hemiteles*) E S

***dodecellae*** (Obrtel & Šedivý, 1960, *Hemiteles*) E S

added by Schwarz & Shaw (2010)

***graculus*** (Gravenhorst, 1829, *Bassus*) E M

*auriculatus* (Thomson, 1884, *Hemiteles*)

*albomarginatus* (Bridgman, 1887, *Hemiteles*)

***infirmus*** (Gravenhorst, 1829, *Hemiteles*)[[251]](#footnote-252)

*tenerrimus* (Gravenhorst, 1829, *Hemiteles*)

***notaticrus*** (Thomson, 1888, *Hemiteles*) E S added by Schwarz & Shaw (2010)

***palpator*** (Müller, 1776, *Ichneumon*) E S W M

*incisus* (Bridgman, 1883, *Hemiteles*)

*hilarellus* (Schmiedeknecht, 1905, *Hemiteles*)

***plumbeus*** (Thomson, 1884, *Hemiteles*) E added by Schwarz & Shaw (2010)

**sp. A**[[252]](#footnote-253) E added by Schwarz & Shaw (2010)

Subfamily CTENOPELMATINAE Förster, 1869[[253]](#footnote-254)

SCOLOBATINAE Schmiedeknecht, 1911

Tribe CHRIONOTINI Uchida, 1957[[254]](#footnote-255)

OLETHRODOTINI Townes, 1970

***OLETHRODOTIS*** Förster, 1869

*TASCHENBERGIA* Schmiedeknecht, 1888

***modestus*** (Gravenhorst, 1829, *Mesoleptus*) E S

*evolans* (Gravenhorst, 1829, *Tryphon*)

*microtamia* (Gravenhorst, 1829, *Phytodietus*)

Tribe CTENOPELMATINI Förster, 1869[[255]](#footnote-256)

***CTENOPELMA*** Holmgren, 1857

*DIEDRUS* Förster, 1869

*ERYMA* Förster, 1869 preocc.

*XANIOPELMA* Tschek, 1869

*ZACHRESTA* Förster, 1869

*HOLMGRENIA* Kriechbaumer, 1877 preocc.

*KRIECHBAUMERIA* Dalla Torre, 1885

*POLYOMORUS* Kriechbaumer, 1894

*POLYHOMORUS* Schulz, 1906

*PSEUDOBANCHUS* Szépligeti, 1911

***ruficorne*** Holmgren, 1857 S added by Shaw *et al.* (2003)

***tomentosum*** (Desvignes, 1856, *Campoplex*) E

*nigrum* misident.

*lucifer* misident.

*luteum* Holmgren, 1857 Aubert (2000)[[256]](#footnote-257)

*xanthostigma* Holmgren, 1857 Aubert (2000); Kasparyan (2004*a*)

*variabile* Tschek, 1869

*gagatinum* (Kriechbaumer, 1894, *Polyomorus*)

*athimi* Kriechbaumer, 1896 Kasparyan (2004*a*)

*pulchrum* (Kriechbaumer, 1877, *Holmgrenia*)

*braunsii* Pfankuch, 1904

*dispar* Ulbricht, 1916 unavailable

***HOMASPIS*** Förster, 1869

*NEOHOMASPSIS* Heinrich, 1949 unavailable

***analis*** (Holmgren, 1857, *Notopygus*)[[257]](#footnote-258) E

*subalpina* misident.

*defectivus* (Tschek, 1869, *Ctenopelma*) Kasparyan (2004*a*)

*pectator* Aubert, 1989 Kasparyan (2004*a*)

***NOTOPYGUS*** Holmgren, 1857

*ANTIPYGUS* Tschek, 1869

***emarginatus*** Holmgren, 1857 E S

*sinifer* Ulbricht, 1922

***XENOSCHESIS*** Förster, 1869

subgenus ***XENOSCHESIS*** Förster, 1869

*HOMOBIA* Förster, 1869

*GLYPTOCENTRUS* Kriechbaumer, 1894

***fulvipes*** (Gravenhorst, 1829, *Exetastes*) E S

*ruficornis* (Rudow, 1883, *Exetastes*)

*ruficornis* (Rudow, 1886, *Exetastes*) preocc.

*varicoxa* Heinrich, 1949 Aubert (2000)

subgenus ***POLYCINETIS*** Förster, 1869

*ERIGLOEA* Förster, 1869

*PROSMORUS* Förster, 1869

*POLYCINETUS* Thomson, 1893

***ustulata*** (Desvignes, 1856, *Tryphon*)[[258]](#footnote-259) E S

*resplendens* (Holmgren, 1857, *Notopygus*)

Shaw *et al.* (2003)

*polita* (Kriechbaumer, 1891, *Erigloea*)

*montana* (Habermehl, 1922, *Hadrodactylus*)

Tribe EURYPROCTINI Thomson, 1883

***ANISOTACRUS*** Schmiedeknecht, 1913

***bipunctatus*** (Gravenhorst, 1829, *Mesoleptus*) E

***tenellus*** (Holmgren, 1857, *Mesoleius*) E S[[259]](#footnote-260) NHM, NMS added here

***xanthostigma*** (Gravenhorst, 1829, *Mesoleptus*) E

*vividus* (Woldstedt, 1874, *Mesoleptus*)

***EURYPROCTUS*** Holmgren, 1857

*HYPOCRYPTUS* Förster, 1869

*SYCHNOLETER* Förster, 1869

*XENONASTES* Förster, 1869

***alpinus*** Holmgren, 1857 E

*exareolatus* Thomson, 1889[[260]](#footnote-261)

***annulatus*** (Gravenhorst, 1829, *Mesoleptus*) E S

*annulator* (Stephens, 1835, *Mesoleptus*)

***bivinctus*** Holmgren, 1857 E W S added by Aubert (2000)

***crassicornis*** Thomson, 1889 E

***geniculosus*** (Gravenhorst, 1829, *Mesoleptus*) E S W M

***holmgreni*** Kerrich, 1942 E

***inferus*** Thomson, 1889 E S

***luteicornis*** (Gravenhorst, 1829, *Tryphon*) E added by Aubert (2000)

***mundus*** (Gravenhorst, 1820, *Ichneumon*) E S

*aberrans* Woldstedt, 1877

*testaceicornis* (Brischke, 1892, *Mesoleptus*)

*strandi* (Gregor, 1937, *Mesoleptus*) Horstmann (2002*b*)

***nemoralis*** (Geoffroy, 1785, *Ichneumon*) E S M

*digitator* (Thunberg, 1824, *Ichneumon*)

?*suborbitalis* (Stephens, 1835, *Mesoleptus*)

*affinis* (Holmgren, 1856, *Mesoleptus*)

*vafer* Woldstedt, 1874

*foersteri* Kriechbaumer, 1897 Horstmann (2002*c*)

***plantator*** (Thunberg, 1824, *Ichneumon*) E added by Aubert (2000)

*albipes* Holmgren, 1857

*tuberculatus* Holmgren, 1857

*exareolatus* Thomson, 1889

***ratzeburgi*** (Gorski, 1852, *Tryphon*) E

*sinister* Brischke, 1871 Horstmann (1998*b*)

*nitidulus* Thomson, 1889

*phygadeuontoides* (Kriechbaumer, 1896, *Polyblastus*)

*pictus* Habermehl, 1925

***GUNOMERIA*** Schmiedeknecht, 1907

***macrodactylus*** (Holmgren, 1856, *Mesoleptus*)[[261]](#footnote-262) E S W I

*scutellata* (Bridgman, 1886, *Mesoleptus*)

***sordida*** (Gravenhorst, 1829, *Mesoleptus*) E S W M

***HADRODACTYLUS*** Förster, 1869[[262]](#footnote-263)

*DIZEMON* Förster, 1869

*NARCOPOEA* Förster, 1869

*ZEMIODES* Förster, 1869

*MEROPACHES* Schmiedeknecht, 1913

***confusus*** (Holmgren, 1858, *Mesoleptus*) E[[263]](#footnote-264)

*albicoxa* Thomson, 1883

***faciator*** (Thunberg, 1824, *Ichneumon*) E S M

*gracilis* (Holmgren, 1856, *Mesoleptus*) preocc.

*curtus* (Holmgren, 1857, *Mesoleptus*)

***femoralis*** (Holmgren, 1857, *Mesoleptus*) E S I

added by Idar (1975)[[264]](#footnote-265)

*intrepidus* Kriechbaumer, 1891 Horstmann (2000*b*)

*nigricoxa* (Thomson, 1893, *Mesoleptus*)

*thomsoni* Schmiedeknecht, 1913

***flavofacialis*** Horstmann, 2000 E S[[265]](#footnote-266) added by Idar (1981); Horstmann (2000*b*); UM

*flavifrontator* misident.

***fugax*** (Gravenhorst, 1829, *Mesoleptus*) E S W I

*ventralis* (Curtis, 1837, *Mesoleptus*)

*marginatus* (Bridgman, 1886, *Mesoleptus*)

*alticola* (Strobl, 1903, *Mesoleptus*)

*branderi* Jussila, 1967

***genalis*** Thomson, 1883 E NHM, det. Broad, added here

*pygmaeus* Habermehl 1925

***gracilipes*** Thomson, 1883 E S

*meridionator* Villemant, 1982 Kasparyan (2011)

***gracilis*** (Stephens, 1835, *Mesoleptus*) E

***graminicola*** Idar, 1979 E S added by Kasparyan & Shaw (2009)

***idari*** Kasparyan & Shaw, 2009 E S added by Kasparyan & Shaw (2009)

*gracilipes* misident.

***indefessus*** (Gravenhorst, 1820, *Ichneumon*) E S I

added by Kasparyan & Shaw (2009)

*tarsator* Thomson, 1883

***insignis*** Kriechbaumer, 1891 E S

*varicoxa* (Thomson, 1893, *Mesoleptus*)

*variicoxa* Dalla Torre, 1901 preocc.

***nigrifemur*** Thomson, 1883 E W S I

***paludicola*** (Holmgren, 1856, *Mesoleptus*) E S I M

*subalpinus* Schmiedeknecht, 1913

***semirufus*** (Holmgren, 1858, *Mesoleptus*) E S W

added by Aubert (2000)

*erythropus* Kriechbaumer, 1891 Horstmann (2000*b*)

*pubescens* Ulbricht, 1922

***spiraculator*** Idar, 1979 E S added by Idar (1981)

***tiphae*** (Geoffroy, 1785, *Ichneumon*) E S I

*luteolus* (Gmelin, 1790, *Ichneumon*) preocc.

*laticeps* Thomson, 1883

*erythropus* Kriechbaumer, 1891

***villosulus*** Thomson, 1883 E M

***vulneratus*** (Zetterstedt, 1838, *Tryphon*) E S added by Aubert (2000)

Species excluded from the British and Irish list

[***bidentulus*** Thomson, 1883[[266]](#footnote-267)]

[***larvatus*** Kriechbaumer, 1891[[267]](#footnote-268)]

***HYPAMBLYS*** Förster, 1869

*APYSTUS* Förster, 1869

*LATHROPHAGUS* Förster, 1869

***albopictus*** (Gravenhorst, 1829, *Tryphon*) E S

*transfuga* (Holmgren, 1857, *Mesoleius*)

*instabilis* (Ruthe, 1859, *Tryphon*)

***HYPSANTYX*** Pfankuch, 1906

***lituratorius*** (Linnaeus, 1761, *Ichneumon*)

*tenthredinum* (Scharfenberg, 1805, *Ichneumon*) preocc.

*impressus* (Gravenhorst, 1829, *Tryphon*)

*crassicornis* (Zetterstedt, 1838, *Pimpla*)

***MESOLEPTIDEA*** Viereck, 1912

*GNATHONOPHORUS* Schmiedeknecht, 1912

***cingulata*** (Gravenhorst, 1829, *Mesoleptus*) E S

*bidens* (Fabricius, 1798, *Ichneumon*) *nom. ob.*

Horstmann (2001*b*)

*bidentor* (Thunberg, 1824, *Ichneumon*) *nom. ob.*

Horstmann (2006*d*)

*pectoralis* (Gravenhorst, 1829, *Mesoleptus*)

*submarginata* (Stephens, 1835, *Mesoleptus*)

*undecimnotata* (Desvignes, 1856, *Mesoleptus*)

***hilaris*** (Gravenhorst, 1829, *Mesoleptus*) E

***prosoleuca*** (Gravenhorst, 1820, *Ichneumon*) E S I

*tricolor* (Fabricius, 1793, *Ophion*) *nom. ob.*

Horstmann (2001*b*)

*neglecta* (Holmgren, 1857, *Mesoleptus*)

*glacialis* (Woldstedt, 1874, *Mesoleptus*)

*similis* (Brischke, 1878, *Mesoleptus*)

*holmgreni* (Thomson, 1893, *Mesoleptus*)

*nigriventris* (Habermehl, 1925, *Mesoleptus*)

***stallii*** (Holmgren, 1858, *Mesoleptus*)

***OCCAPES*** Townes, 1970

***selandriae*** (Brischke, 1878, *Polyblastus*) E W NHM, det. Broad, added here

***PANTORHAESTES*** Förster, 1869

*TROPHOCTONUS* Förster, 1869

***xanthostomus*** (Gravenhorst, 1829, *Tryphon*) E S W M

*ochrostomus* (Gravenhorst, 1829, *Tryphon*)

*rufocinctus* (Gravenhorst, 1829, *Mesoleptus*)

*hilarellus* (Holmgren, 1858, *Euryproctus*)

*intensicolor* (Heinrich, 1953, *Dialges*)

***PHOBETES*** Förster, 1869

*IPOCTONUS* Förster, 1869 preocc.

*PHILOTYMMA* Förster, 1869

*PHOBETUS* Thomson, 1889 preocc.

*GRIPHODES* Kriechbaumer, 1894

*IPOCTONINUS* Hincks, 1944

*PHOBETELLUS* Hincks, 1944

***atomator*** (Müller, 1776, *Ichneumon*) E W M

*croatica* (Kiss, 1926, *Brischkea*) Horstmann (2007*a*)

***cerinostomus*** (Gravenhorst, 1829, *Mesoleptus*) E

NMS, det. Kasparyan, added here

***chrysostomus*** (Gravenhorst, 1820, *Ichneumon*) E

***femorator*** (Thomson, 1893, *Phobetus*) E

*subalpinus* (Strobl, 1903, *Euryproctus*)

***fuscicornis*** (Holmgren, 1856, *Tryphon*)

*fulviventris* (Thomson, 1893, *Phobetus*)

***leptocerus*** (Gravenhorst, 1820, *Ichneumon*) E S W

*stigmaticus* (Brischke, 1878, *Mesoleptus*)

*schmiedeknechti* (Lange, 1911, *Hadrodactylus*)

*aigneri* (Kiss, 1926, *Brischkea*) Horstmann (2007*a*)

***liopleuris*** (Thomson, 1889, *Euryproctus*) E[[268]](#footnote-269) added by Aubert (2000)

***nigriceps*** (Gravenhorst, 1829, *Tryphon*)[[269]](#footnote-270) E S

*praetermissus* (Woldstedt, 1874, *Mesoleptus*)

***SYNDIPNUS*** Förster, 1869

*POLYPYSTIS* Förster, 1869

*TLEMON* Förster, 1869

*DICKSONIA* Holmgren, 1880

*NEASTUS* Holmgren, 1883

***alutaceus*** (Holmgren, 1857, *Trematopygus*) S

added by Aubert (2000)

*alutaceus* (Woldstedt, 1874, *Mesoleius*) preocc.

***decipiens*** (Woldstedt, 1877, *Mesoleius*) E NMS, added here

*subscaber* Thomson, 1893

***lateralis*** (Gravenhorst, 1829, *Tryphon*) E S

*punctiscuta* Thomson, 1894

***macrocerus*** (Thomson, 1883, *Euryproctus*) E added by Aubert (2000)

***SYNODITES*** Förster, 1869

*CAMPONASTES* Förster, 1869

*LISTROTA* Förster, 1869

*POLYTERUS* Förster, 1869

*SARCORYCHUS* Förster, 1869

*SYCHNOPORTUS* Förster, 1869

*ZOOTREPHES* Förster, 1869

*ZOOTREPHUS* Thomson, 1890

*SYNODYTES* Thomson, 1893

*ANAGLYMMUS* Roman, 1914

***breviusculus*** (Fonscolombe, 1849, *Tryphon*) E[[270]](#footnote-271)

*delicatus* (Fonscolombe, 1849, *Tryphon*)

*buccatus* (Holmgren, 1857, *Mesoleius*)

***carinatus*** (Holmgren, 1857, *Mesoleius*) E added by Aubert (2000)

***erosus*** (Holmgren, 1857, *Trematopygus*) S[[271]](#footnote-272) added by Aubert (2000)

***facialis*** (Thomson, 1893, *Spudaeus*) E[[272]](#footnote-273)

***hilaris*** (Woldstedt, 1880, *Bassus*) S NMS, added here

***lineiger*** (Thomson, 1893, *Syndipnus*) E added by Aubert (2000)

***notatus*** (Gravenhorst, 1829, *Tryphon*) E S W

*bimaculatus* (Desvignes, 1856, *Tryphon*)

*assimilis* (Holmgren, 1858, *Tryphon*)

*aberrans* (Brischke, 1871, *Polyblastus*)

*hungaricus* (Kiss, 1924, *Ipoctonus*) Horstmann (2007*a*)

***parviceps*** (Thomson, 1894, *Syndipnus*) S NMS, det. Kasparyan, added here

species excluded from the British list

[***breviventris*** (Gravenhorst, 1829, *Hemiteles*)[[273]](#footnote-274)]

***SYNOMELIX*** Förster, 1869[[274]](#footnote-275)

***albipes*** (Gravenhorst, 1829, *Tryphon*) E S

*sieboldii* Kriechbaumer, 1897

*kriechbaumeri* Schmiedeknecht, 1913

***faciator*** Idar, 1983 E S added by Idar (1983)

***perfida*** (Woldstedt, 1874, *Tryphon*) S

*curvula* (Thomson, 1895, *Syndipnus*)

***ZEMIOPHORA*** Förster, 1869

*ZEMIOPHORUS* Thomson, 1893

***scutulata*** (Hartig, 1838, *Tryphon*) E

*brischkei* (Holmgren, 1871, *Mesoleius*)

*nobilis* (Habermehl, 1909, *Otlophorus*)

Tribe MESOLEIINI Thomson, 1883

***ALEXETER*** Förster, 1869[[275]](#footnote-276)

*ADRANES* Förster, 1869

*ZEMIOPHRON* Förster, 1869

***clavator*** (Müller, 1776, *Ichneumon*) E

*testaceator* misident.

*testaceus* misident.

*venosus* (Gmelin, 1790, *Ichneumon*)

***coxalis*** (Brischke, 1871, *Mesoleptus*) S I NHM, det. Aubert and Broad, added here

*inconspicuus* Schiedeknecht, 1914

*rufus* Kiss, 1926

***fallax*** (Holmgren, 1857, *Mesoleius*) E S

***multicolor*** (Gravenhorst, 1829, *Tryphon*) E S W M

*dives* (Holmgren, 1857, *Mesoleius*)

*napaeus* (Holmgren, 1857, *Mesoleius*)

***nebulator*** (Thunberg, 1824, *Ichneumon*) E S

*melanocephalus* (Gravenhorst, 1829, *Mesoleptus*)

*gracilipes* (Curtis, 1837, *Mesoleptus*)

*paludicola* Habermehl, 1922

***niger*** (Gravenhorst, 1829, *Tryphon*) E I

***rapinator*** (Gravenhorst, 1829, *Tryphon*) E

*laevissimus* (Strobl, 1903, *Mesoleius*)

***segmentarius*** (Fabricius, 1787, *Ichneumon*) E S

*sectator* (Thunberg, 1824, *Ichneumon*) Horstmann (2001*b*)

*fraternarius* (Thunberg, 1824, *Ichneumon*)

*maxillarius* (Thunberg, 1824, *Ichneumon*)

*ruficornis* (Gravenhorst, 1829, *Mesoleptus*)

*lugubris* (Woldstedt, 1874, *Mesoleptus*)

*sibiricus* Kiss, 1926

*rufopetiolaris* Kiss, 1933

species of *Alexeter* excluded from the British and Irish list

[***attenuatus*** (Bridgman, 1887, *Mesoleius*)[[276]](#footnote-277)]

***ANONCUS*** Townes, 1970[[277]](#footnote-278)

***femorator*** (Thomson, 1893, *Mesoleius*) S added by Shaw & Kasparyan (2003)

***gracilicornis*** (Holmgren, 1857, *Mesoleius*) E

species of *Anoncus* excluded from the British and Irish list

[***linitus*** (Holmgren, 1857, *Mesoleius*)[[278]](#footnote-279)]

***ARBELUS*** Townes, 1970

***athaliaeperda*** (Curtis, 1860, *Bassus*) E I

*athaliiperdus* (Marshall, 1872, *Bassus*)

***AZELUS*** Förster, 1869[[279]](#footnote-280)

***erythropalpus*** (Gmelin, 1790, *Ichneumon*) E W S

*laterator* (Thunberg, 1824, *Ichneumon*)

*triangulatus* (Bridgman, 1886, *Perilissus*)

*bipunctatus* (Szépligeti, 1901, *Mesoleius*) preocc.

*csikii* (Kiss, 1926, *Barytarbes*) Horstmann (2007*a*)

***BARYTARBES*** Förster, 1869

*HYBRISTES* Förster, 1869

*ISODIAETA* Förster, 1869

*POLYTRERA* Förster, 1869

*BARYTARBUS* Thomson, 1883

*POLYTRERES* Thomson, 1892

*APHOLIUM* Townes, 1970 Aubert (2000)

***colon*** (Gravenhorst, 1829, *Tryphon*) E

*ventosus* (Holmgren, 1876, *Mesoleius*)

***flavicornis*** (Thomson, 1892, *Mesoleius*)[[280]](#footnote-281) E

*segmentarius* (Perkins, 1962, *Isodiaeta*) unavailable

Horstmann (2004*c*, 2005*b*)

***flavoscutellatus*** (Thomson, 1892, *Mesoleius*) E

***laeviusculus*** (Thomson, 1883, *Mesoleius*) E

***CAMPODORUS*** Förster, 1869[[281]](#footnote-282)

*PHAGESORUS* Förster, 1869

*CUBOSCOPESIS* Heinrich, 1952

***alticola*** (Holmgren, 1857, *Mesoleius*) S added by Shaw & Kasparyan (2003)

***amictus*** (Holmgren, 1857, *Mesoleius*) E S

***astutus*** (Holmgren, 1876, *Mesoleius*) E S

***caligatus*** (Gravenhorst, 1829, *Tryphon*) E S

*nemati* (Ratzeburg, 1852, *Tryphon*)

***ciliatus*** (Holmgren, 1857, *Mesoleius*) S added by Shaw & Kasparyan (2003)

***commotus*** (Holmgren, 1876, *Mesoleius*) S added by Shaw & Kasparyan (2003)

*perturbatus* (Holmgren, 1876, *Mesoleius*)

***corrugatus*** (Holmgren, 1876, *Mesoleius*)

***difformis*** (Holmgren, 1876, *Mesoleius*) E W S added by Shaw & Kasparyan (2003)

***dorsalis*** (Gravenhorst, 1829, *Tryphon*) E W S M

***efferus*** (Holmgren, 1876, *Mesoleius*) E S added by Shaw & Kasparyan (2003)

***elegans*** (Parfitt, 1882, *Mesoleius*)[[282]](#footnote-283) E

***flavescens*** Kasparyan, 2003 S NMS, det. Kasparyan, added here

***gallicus*** (Thomson, 1893, *Mesoleius*) S added by Shaw & Kasparyan (2003)

***haematodes*** (Gravenhorst, 1829, *Tryphon*) E I

*alni* (Woldstedt, 1874, *Mesoleius*)

***hamulus*** (Gravenhorst, 1829, *Tryphon*) E

*nobilis* (Holmgren, 1857, *Mesoleius*)

***ignavus*** (Holmgren, 1857, *Mesoleius*)

***immarginatus*** (Thomson, 1893, *Mesoleius*)[[283]](#footnote-284) S

***incidens*** (Thomson, 1893, *Mesoleius*) E S

***liosternus*** (Thomson, 1893, *Mesoleius*)

***luctuosus*** (Holmgren, 1857, *Mesoleius*)

***maculicollis*** (Stephens, 1835, *Tryphon*) E S

*vigens* (Holmgren, 1857, *Mesoleius*)

***marginalis*** (Geoffroy, 1785, *Ichneumon*) E

*limbarius* (Olivier, 1792, *Ichneumon*)

*histrio* (Fabricius, 1793, *Ichneumon*) preocc.

*formosus* (Gravenhorst, 1829, *Tryphon*)

*limbarius* (Fonscolombe, 1854, *Lissonota*) preocc.

*lepidus* (Giraud, 1872, *Mesoleius*) Horstmann (2008*d*)

*ornatus* (Habermehl, 1925, *Mesoleius*) Horstmann (2008*d*)

***mediosanguineus*** (Heinrich, 1950, *Mesoleius*) I

NHM, det. Broad, added here

***melanogaster*** (Holmgren, 1857, *Mesoleius*) E S

added by Shaw & Kasparyan (2003)

***mixtus*** (Holmgren, 1857, *Mesoleius*)

***molestus*** (Holmgren, 1857, *Mesoleius*)

***nigridens*** (Thomson, 1893, *Spudaeus*) S

***patagiatus*** (Holmgren, 1876, *Mesoleius*)

*modestus* (Holmgren, 1876, *Mesoleius*)

***pectinator*** Kasparyan, 2003 E added by Kasparyan (2003)

**?*pictipes*** (Habermehl, 1923, *Mesoleius*)[[284]](#footnote-285)

***scapularis*** (Stephens, 1835, *Tryphon*) E S

*humerellus* (Thomson, 1893, *Mesoleius*) Kasparyan (2003)

***tristis*** (Holmgren, 1857, *Mesoleius*)

***variegatus*** (Jurine, 1807, *Anomalon*) E S

*sanguinicollis* (Gravenhorst, 1829, *Tryphon*)

***viduus*** (Holmgren, 1857, *Mesoleius*) E UM

*annulatus* (Brischke, 1878, *Trematopygus*)

***vitosaensis*** (Gregor, 1933, *Mesoleius*)

*trochanteratus* (Kriechbaumer, 1896, *Mesoleius*)[[285]](#footnote-286) preocc.

***HIMERTA*** Förster, 1869[[286]](#footnote-287)

*CLEPSIPORTHUS* Förster, 1869

*DOLIOCTONUS* Förster, 1869

*ENOECETIS* Förster, 1869

*ITHAGENES* Förster, 1869

*HIMERTUS* Thomson, 1883

***bisannulata*** (Thomson, 1883, *Euryproctus*)[[287]](#footnote-288) added by Horstmann (2002*c*)

*pfeifferi* (Bauer, 1939, *Himertus*) Horstmann (2002*c*)

***defectiva*** (Gravenhorst, 1820, *Ichneumon*) E S

*varicornis* (Gravenhorst, 1829, *Tryphon*) Horstmann (2002*c*)

*biannulata* (Ulbricht, 1922, *Barytarbes*)

*ihsseni* (Bauer, 1939, *Himertus*) Horstmann (2002*c*)

***scutellaris*** (Kriechbaumer, 1897, *Enoecetis*) E

NHM, det. Broad, added here

***sepulchralis*** (Holmgren, 1876, *Mesoleius*) E S

*sexannulatus* Kriechbaumer, 1891 Horstmann (2002*c*)

*alboannulata* (Strobl, 1903, *Euryproctus*)

***HYPERBATUS*** Förster, 1869[[288]](#footnote-289)

***orbitalis*** (Thomson, 1893, *Mesoleius*)[[289]](#footnote-290) E S added by Shaw & Kasparyan (2003)

***segmentator*** (Holmgren, 1857, *Mesoleius*) S

*solitarius* (Holmgren, 1876, *Mesoleius*)

***sternoxanthus*** (Gravenhorst, 1829, *Tryphon*) E

*pulchellus* (Holmgren, 1857, *Mesoleius*)

***LAGAROTIS*** Förster, 1869

*DASPLETIS* Förster, 1869

*DYSANTES* Förster, 1869

*NYTHOPHONA* Förster, 1869

*ONEISTA* Förster, 1869

*LAGAROTUS* Thomson, 1892

***debitor*** (Thunberg, 1824, *Ichneumon*) E

*insolens* (Gravenhorst, 1829, *Tryphon*)

***erythrocera*** (Gravenhorst, 1829, *Tryphon*)[[290]](#footnote-291) E

***semicaligata*** (Gravenhorst, 1820, *Ichneumon*) E S

*longicornis* (Woldstedt, 1874, *Mesoleius*)

***LAMACHUS*** Förster, 1869

*ADEXIOMA* Förster, 1869

*ZAPHTHORA* Förster, 1869

*BATHYGLYPTUS* Schmiedeknecht, 1913

*TOROCAMPUS* Schmiedeknecht, 1913

***coalitorius*** (Thunberg, 1824, *Ichneumon*) E W[[291]](#footnote-292)

*variabilis* (Ratzeburg, 1844, *Tryphon*) Horstmann (2004*c*)

*ophthalmicus* (Holmgren, 1857, *Mesoleius*)

*marginatus* (Brischke, 1871, *Mesoleius*)

*spectabilis* (Holmgren, 1876, *Mesoleius*)

***eques*** (Hartig, 1838, *Tryphon*) E S Shaw & Kasparyan (2005)

*silvarum* (Holmgren, 1876, *Mesoleius*)

*aterrimus* (Scönwiese, 1934, *Torocampus*)

***frutetorum*** (Hartig, 1838, *Tryphon*) S added by Aubert (2000)

*lophyrum* (Hartig, 1838, *Tryphon*)

*nigrescens* Kiss, 1926

*altipeta* Heinrich, 1953

***pini*** (Bridgman, 1882, *Mesoleius*) S

*caledonicus* Laidlaw, 1933

***virgultorum*** (Gravenhorst, 1829, *Tryphon*) E

*flavoscutellatus* (Strobl, 1903, *Notopygus*)

***MESOLEIUS*** Holmgren, 1856[[292]](#footnote-293)

*ALLOCRITUS* Förster, 1869

*ALFKENIA* Pfankuch, 1906

*MESOLIUS* Pfankuch, 1906

*HABRODEMUS* Schmiedeknecht, 1913

***aceris*** Kasparyan & Shaw, 2003 E S M added by Shaw & Kasparyan (2003)

***armillatorius*** (Gravenhorst, 1807, *Ichneumon*) E W S M

*luteifrons* (Gravenhorst, 1829, *Tryphon*)

*flavipes* Brischke, 1871 Horstmann (2012*c*)

*similis* Brischke, 1892 preocc.

*bilineolatus* Strobl, 1903 Horstmann (2012*c*)

*chyzeri* (Kiss, 1926, *Perilissus*) Horstmann (2007*a*)

***aulicus*** (Gravenhorst, 1829, *Tryphon*) E S

***axillaris*** (Stephens, 1835, *Tryphon*)[[293]](#footnote-294) E W S M

*amabilis* Holmgren, 1857 Kasparyan (2000)

*leptogaster* Holmgren, 1857 Kasparyan (2000)

*tenuiventris* Holmgren, 1858 Kasparyan (2000)

*erythrogaster* Holmgren, 1876 Kasparyan (2000)

***brevipalpis*** Thomson, 1893 E added by Shaw & Kasparyan (2003)

***caninae*** Bridgman, 1886[[294]](#footnote-295) E

***dubius*** Holmgren, 1857 E

***filicornis*** Holmgren, 1876 E S W M

***flavopictus*** (Gravenhorst, 1829, *Mesoleptus*) E S

*trimaculatus* (Stephens, 1835, *Mesoleptus*)

***frenalis*** Thomson, 1893

***furax*** Holmgren, 1857

***fuscipes*** Holmgren, 1857[[295]](#footnote-296) S M

***geniculatus*** Holmgren, 1857 W S added by Shaw & Kasparyan (2003)

***intermedius*** (Gravenhorst, 1829, *Tryphon*)[[296]](#footnote-297) E W

added by Shaw & Kasparyan (2003)

*sinuatus* Thomson, 1893 Kasparyan (2000)

***laricis*** Teunissen, 1953 S added by Shaw & Kasparyan (2003)

***lindemansi*** Teunissen, 1953 E S added by Shaw & Kasparyan (2003)

*antennator* Kasparyan, 2000 Kasparyan (2004*b*)

***melanoleucus*** (Gravenhorst, 1829, *Tryphon*) E S

***nivalis*** Holmgren, 1857 E W S M

*aemulus* (Ruthe, 1859, *Tryphon*)

***opticus*** (Gravenhorst, 1829, *Tryphon*) E S I

***peronatus*** (Marshall, 1876, *Bassus*)[[297]](#footnote-298) I

***phyllotomae*** Cushman, 1933 S added by Shaw & Kasparyan (2003)

***placidus*** Holmgren, 1857

***pyriformis*** (Ratzeburg, 1852, *Tryphon*) E S

*unifasciatus* Holmgren, 1857

***ribesii*** Bauer, 1961 E S added by Shaw & Kasparyan (2003)

***roepkii*** Teunissen, 1945 W S added by Shaw & Kasparyan (2003)

***tenthredinis*** Morley, 1912 E UM

*romani* Teunissen, 1945

***varicoxa*** Thomson, 1893

doubtfully placed species of *Mesoleius*

[***brachyacanthus*** Parfitt, 1881 nom. dub. E]

***OTLOPHORUS*** Förster, 1869

*AEOLOMETIS* Förster, 1869

*DIALGES* Förster, 1869

*HOLMGRENIA* Förster, 1869

*NEALES* Förster, 1869

*TACHYPORTHUS* Förster, 1869

*AELOMETIS* Thomson, 1893

*OTLOPHORINUS* Hincks, 1944

***anceps*** (Holmgren, 1857, *Mesoleius*) E added by Aubert (2000)

***congruens*** (Holmgren, 1858, *Mesoleius*) E added by Aubert (2000)

***italicus*** (Gravenhorst, 1829, *Scolobates*) E

*corallinus* (Vollenhoven, 1873, *Scolobates*)

***pulverulentus*** (Holmgren, 1857, *Mesoleius*) E

*minutus* (Rudow, 1881, *Meniscus*)

***senilis*** (Holmgren, 1876, *Mesoleius*) E NHM, det. Aubert, added here

***vepretorum*** (Gravenhorst, 1829, *Tryphon*) E

*verpetorum* misspelling

***PERISPUDA*** Förster, 1869

*GENARCHES* Förster, 1869

*ZAPLETHIS* Förster, 1869

*PERISPUDUS* Thomson, 1888

***bignellii*** (Bridgman, 1881, *Mesoleius*)[[298]](#footnote-299) E S I

*flavitarsis* (Thomson, 1893, *Mesoleius*)

*sulphuripes* (Strobl, 1902, *Procinetus*)

***facialis*** (Gravenhorst, 1829, *Mesoleptus*) E

***sulphurata*** (Gravenhorst, 1807, *Ichneumon*) E I

***PROTARCHUS*** Förster, 1869

*ZACALLES* Förster, 1869

***melanurus*** (Thomson, 1893, *Mesoleius*) E S[[299]](#footnote-300)

added by Aubert (2000); UM

*rufus* misident.

***testatorius*** (Thunberg, 1824, *Ichneumon*) E S

*binarius* (Thunberg, 1824, *Ichneumon*) preocc.

*decorius* (Thunberg, 1824, *Ichneumon*)

*rufus* (Gravenhorst, 1829, *Tryphon*)

*conspicuus* (Stephens, 1835, *Tryphon*)

*rufulus* (Stephens, 1835, *Tryphon*)

***RHINOTORUS*** Förster, 1869

*SPUDAEA* Förster, 1869 preocc.

*SPUDAEUS* Thomson, 1883 preocc.

*PROSPUDAEA* Hincks, 1944

***compactor*** (Thunberg, 1824, *Ichneumon*) E

*atratus* (Holmgren, 1857, *Trematopygus*)

*quadriguttatus* (Vollenhoven, 1873, *Bassus*)

*albotrochanteratus* (Strobl, 1913, *Polyblastus*)

***leucostomus*** (Gravenhorst, 1829, *Tryphon*) E

*impressus* (Brischke, 1871, *Mesoleius*)

*subimpressus* (Thomson, 1873, *Spudaeus*)

***longicornis*** (Schmiedeknecht, 1914, *Spudaea*) E

***mesocastanus*** (Thomson, 1892, *Spudaeus*) E

NHM, det. Reshchikov, added here

***nasutus*** (Gravenhorst, 1829, *Tryphon*) E NHM, det. Perkins, added here

*confusus* (Thomson, 1883, *Mesoleius*)

***similis*** (Brischke, 1892, *Mesoleius*)

***SAOTIS*** Förster, 1869[[300]](#footnote-301)

*SAOTUS* Thomson, 1883

*ISKARUS* Kolarov, 1987 Kasparyan & Shaw (2003)

***albionis*** Kasparyan, 2007 E added by Kasparyan & Khalaim (2007*a*)

***albiventris*** Kasparyan, 2007 E S added by Kasparyan & Khalaim (2007*a*)

***compressiuscula*** (Thomson, 1883, *Mesoleius*) E

***morleyi*** Fitton, 1976[[301]](#footnote-302) E S

*emarginata* (Morley, 1911, *Homocidus*) preocc.

***nigriscuta*** (Thomson, 1888, *Mesoleius*) E added by Kasparyan & Shaw (2003)

***renovata*** (Morley, 1911, *Mesoleius*) E[[302]](#footnote-303)

***varicoxa*** (Thomson, 1893, *Saotus*) E S

***SCOPESIS*** Förster, 1869

*SCOPARCHES* Förster, 1869

*SCOPESUS* Thomson, 1893

***bicolor*** (Gravenhorst, 1829, *Tryphon*) E

*praecatoria* (Holmgren, 1876, *Mesoleius*)

*longigena* (Thomson, 1893, *Mesoleius*) Horstmann (2006*a*)

***depressa*** (Thomson, 1893, *Mesoleius*) E

***fraterna*** (Holmgren, 1857, *Mesoleius*) E

***frontator*** (Thunberg, 1824, *Ichneumon*)[[303]](#footnote-304) E S

*rufolabris* (Zetterstedt, 1838, *Bassus*)

***gesticulator*** (Thunberg, 1824, *Ichneumon*) E

*longipes* (Gravenhorst, 1829, *Tryphon*)

*nigricollis* (Gravenhorst, 1829, *Tryphon*)

***macropus*** (Thomson, 1893, *Mesoleius*) E

***obscura*** (Holmgren, 1857, *Mesoleius*)

***rufonotata*** (Holmgren, 1876, *Mesoleius*) E

*thomsoni* (Habermehl, 1925, *Scopesus*)

***tegularis*** (Thomson, 1893, *Mesoleius*) E UM

***SEMIMESOLEIUS*** Ozols, 1963

***exophthalmicus*** Ozols, 1963 S added by Shaw & Kasparyan (2003)

***SMICROLIUS*** Thomson, 1893

***parvicalcar*** (Thomson, 1895, *Syndipnus*) E S added by Shaw & Kasparyan (2003)

*parumpictus* (Roman, 1909, *Mesoleius*) Kasparyan (1998)

Tribe PERILISSINI Thomson, 1883

***ABSYRTUS*** Holmgren, 1859

*ECZETESIS* Förster, 1869

***vernalis*** Bauer, 1961 E added by Aubert (2000)

***vicinator*** (Thunberg, 1824, *Ichneumon*) E S W I

*luteus* Holmgren, 1859

*exareolatus* Ulbricht, 1926 unavailable

***Lathiponus*** Förster, 1869

*Polyselasmus* Schmiedeknecht, 1912

*Ceratosaotis* Gregor, 1939

***semiluctuosus*** (Vollenhoven, 1878, *Eclytus*) E

NHM, NMS, det. Perkins, Kasparyan, Broad, added here

*frigidus* (Woldstedt, 1874, *Perilissus*) preocc.

*bicolor* (Brischke, 1878, *Perilissus*)

*pulcherrimus* (Thomson, 1888, *Mesoleius*)

*ornatus* (Gregor, 1939, *Ceratosaotis*)

***LATHROLESTES*** Förster, 1869

*CAMPORYCHUS* Förster, 1869

*ECCLINOPS* Förster, 1869

*HOMALOMMA* Förster, 1869

*LAPHYROSCOPUS* Förster, 1869

*POLYONCUS* Förster, 1869

*LATHROLESTUS* Thomson, 1883

*LUPHYROSCOPUS* Thomson, 1883

*TRYPHONOPSIS* Brauns, 1898

*RITZEMABOSIA* Smits van Burgst, 1912

*CULMINA* Benoit, 1955

***bipunctatus*** (Bridgman, 1886, *Grypocentrus*) E S

***buccinator*** (Holmgren, 1857, *Perilissus*)[[304]](#footnote-305) E

*vollenhoveni* (Gribodo, 1880, *Perilissus*) Horstmann (2003*a*)

***caudatus*** (Thomson, 1883, *Lathrolestus*) E S NHM, NMS, added here

***citreus*** (Brischke, 1878, Perilissus) E NHM, added here

***clypeatus*** (Zetterstedt, 1838, *Tryphon*) E S added by Heath (1961)[[305]](#footnote-306)

***ensator*** (Brauns, 1898, *Tryphonopsis*) E

*dilatatus* (Nordenström, 1905, *Lathrolestus*)

*ensatrix* (Schulz, 1906, *Tryphonopsis*)

***erythrocephalus*** (Gravenhorst, 1829, *Tryphon*)[[306]](#footnote-307) E

***lucidulus*** (Holmgren, 1857, *Perilissus*) E AEI[[307]](#footnote-308), det. Reshchikov, added here

***luteolator*** (Gravenhorst, 1829, *Mesoleptus*) E

*gorskii* (Ratzeburg, 1852, *Tryphon*)

***macropygus*** (Holmgren, 1857, *Perilissus*) E S

***moravicus*** (Habermehl, 1923, *Perilissus*) E NHM, NMS, added here

***nigricollis*** (Thomson, 1883, *Perilissus*) E

*minutus* (Bridgman, 1887, *Perilissus*)

***orbitalis*** (Gravenhorst, 1829, *Tryphon*) E S W

*bucculentus* (Holmgren, 1857, *Perilissus*)

***pictilis*** (Holmgren, 1857, *Perilissus*) E S I[[308]](#footnote-309)

***pleuralis*** (Thomson, 1883, *Lathrolestus*) E

***soperi*** Reshchikov, 2010 E added by Reshchikov (2015)

***tripunctor*** (Thunberg, 1824, *Ichneumon*)[[309]](#footnote-310) E NHM, added here

*distichor* (Thunberg, 1824, *Ichneumon*)

*longicornis* (Brischke, 1871, *Perilissus*)

*luteocephalus* (Giraud, 1872, *Perilissus*)

*singularis* (Vollenhoven, 1878, *Perilissus*)

*grandiceps* (Thomson, 1883, *Perilissus*)

***ungularis*** (Thomson, 1883, *Lathrolestus*) E

*citrofrontalis* Schmiedeknecht, 1912 Reshchikov (2011)

***verticalis*** (Brischke, 1871, *Perilissus*) E S I

*abdominalis* (Brischke, 1878, *Perilissus*) Horstmann (2006*a*)

*marginatus* (Thomson, 1883, *Lathrolestus*)

***LOPHYROPLECTUS*** Thomson, 1883[[310]](#footnote-311)

***oblongopunctatus*** (Hartig, 1838, *Paniscus*) E

*luteator* (Thunberg, 1824, *Ichneumon*) preocc.

***OETOPHORUS*** Förster, 1869

*SYMPHOBUS* Förster, 1869

***naevius*** (Gmelin, 1790, *Ichneumon*) E S I M

*dilector* (Thunberg, 1824, *Ichneumon*)

*limitaris* (Gravenhorst, 1829, *Mesoleptus*)

***OPHELTES*** Holmgren, 1859[[311]](#footnote-312)

***glaucopterus*** (Linnaeus, 1758, *Ichneumon*) E S I

*pteromelas* (Villers, 1789, *Ichneumon*)

***PERILISSUS*** Holmgren, 1857

*EXACRODUS* Förster, 1869

*ICHNAEOPS* Förster, 1869

*SPANOTECNUS* Förster, 1869

*UDENIA* Förster, 1869

*DAUGNA* Seyrig, 1935

*PSEUDOCHORUS* Rao, 1953

***albitarsis*** Thomson, 1883 E S NHM, NMS, UM, added here

*emarginatus* Thomson, 1883

***compressus*** Thomson, 1883 E NHM, added here

***coxalis*** Thomson, 1883 E NHM, det. Broad, added here

***lutescens*** Holmgren, 1857 E

***pallidus*** (Gravenhorst, 1829, *Mesoleptus*) E

*holmgreni* Habermehl, 1925

***rufoniger*** (Gravenhorst, 1820, *Ichneumon*) E

*vernalis* (Gravenhorst, 1820, *Ichneumon*)

*petulans* (Gravenhorst, 1829, *Tryphon*)

*herrichii* Kriechbaumer, 1892

***sericeus*** (Gravenhorst, 1829, *Mesoleptus*)

*spiniger* Thomson, 1883

***spilonotus*** (Stephens, 1835, *Mesoleptus*) E S W

*subcinctus* Holmgren, 1857

*stigmaticus* Woldstedt, 1874

*dissimilis* Woldstedt, 1878

*thuringiacus* Schmiedeknecht, 1912

*alpinus* Habermehl, 1935

***variator*** (Müller, 1776, *Ichneumon*)[[312]](#footnote-313) E S W I M

*filicornis* (Gravenhorst, 1820, *Ichneumon*)

*interruptor* (Thunberg, 1824, *Ichneumon*)

*seminiger* (Gravenhorst, 1829, *Mesoleptus*)

***PRIOPODA*** Holmgren, 1856

*PRIONOPODA* misspelling

***apicaria*** (Geoffroy, 1785, *Ichneumon*) E S Gauld (1970)

*stictica* misident.

*luteolus* (Thunberg, 1789, *Ichneumon*) Horstmann (1999*b*)

*glabrator* (Thunberg, 1824, *Ichneumon*)

***xanthopsana*** (Gravenhorst, 1829, *Mesoleptus*) E

*xanthospana* misspelling

***SYNOECETES*** Förster, 1869

*POLYRHYSIA* Förster, 1869

*SYNAGRYPNUS* Förster, 1869

*POLYRHYSIUS* Thomson, 1893

***anterior*** (Thomson, 1893, *Syndipnus*) E S I NHM, NMS, UM, added here

***TREMATOPYGODES*** Aubert, 1968

***aprilinus*** (Giraud, 1872, *Trematopygus*) E added by Hinz & Horstmann (1998)

*blancoburgensis* (Schmiedeknecht, 1912, *Lathrolestes*)

***rarus*** Horstmann, 1990 E NMS, det. Kasparyan, added here

***ZAPLETHOCORNIA*** Schmiedeknecht, 1912

***exstinctor*** Aubert, 1985 E added by Aubert (1985)

Tribe PIONINI Smith & Shenefelt, 1955

***ASTHENARA*** Förster, 1869

*ASTHENARUS* Thomson, 1889

***scabricula*** (Thomson, 1893, *Catoglyptus*) E W S I

NHM, NMS, UM, added here

***socia*** (Holmgren, 1857, *Euryproctus*) E S I NHM, NMS, added here

*crassifemur* (Thomson, 1889, *Asthenarus*)

***GLYPTORHAESTUS*** Thomson, 1894

*LOXONEURUS* Schmiedeknecht, 1913

***boschmai*** Teunissen, 1953 E NHM, NMS, added here

***periclistor*** Hinz, 1975 E NMS, added here

***punctatus*** (Thomson, 1890, *Rhaestus*) E NMS, NHM, added here

***punctulatus*** (Woldstedt, 1877, *Mesoleius*) E

*wuestneii* (Thomson, 1893, *Rhaestus*)

*thuringiacus* (Schmiedeknecht, 1913, *Loxoneurus*)

***selandrivorus*** (Giraud, 1872, *Trematopygus*) E

NHM, added here

***LABROSSYTA*** Förster, 1869

*LABROSSYTUS* Thomson, 1893

*LIOTRYPHON* Strobl, 1903 preocc.

***scotoptera*** (Gravenhorst, 1820, *Ichneumon*) E

*fumata* (Bridgman, 1880, *Perilissus*)

***LETHADES*** Davis, 1897

***cingulator*** Hinz, 1976 E S NHM, NMS, added here

***curvispina*** (Thomson, 1883, *Trematopygus*) S

NHM, NMS, added here

*alpinus* (Zetterstedt, 1838, *Tryphon*) *nom. ob.* [[313]](#footnote-314)

*flavifrons* (Zetterstedt, 1838, *Tryphon*) *nom. ob.*

***facialis*** (Brischke, 1871, *Trematopygus*)[[314]](#footnote-315) E S W

NHM, det. Hinz & Horstmann, added here

***imperfecti*** Hinz, 1996 E added by Hinz (1996)

***subcoriaceus*** (Strobl, 1903, *Mesoleius*) E S NMS, det. Kasparyan, added here

*laricis* Hinz, 1976 Horstmann (2011*a*)

***PHAESTUS*** Förster, 1869

***anomalus*** (Brischke, 1871, *Grypocentrus*) E

*heterocerus* (Thomson, 1893, *Rhaestus*)

***PION*** Schiødte, 1839

*CATOGLYPTUS* Förster, 1855

***nigripes*** Schiødte, 1839 E S NHM, NMS, added here

*crassipes* (Holmgren, 1857, *Catoglyptus*) Horstmann (2004*b*)

***fortipes*** (Gravenhorst, 1829, *Mesoleptus*) E S I M

*pictus* (Pfankuch, 1924, *Catoglyptus*) unavailable

*transsylvanicus* (Kiss, 1924, *Mesoleptus*) Horstmann (2007*a*)

*clarus* (Kiss, 1933, *Brischkea*) Horstmann (2007*a*)

***RHAESTUS*** Thomson, 1883

*RHAESTES* Förster, 1869 preocc.

***lativentris*** (Holmgren, 1858, *Grypocentrus*) E S

***rufipes*** (Holmgren, 1857, *Grypocentrus*) E S I added by Aubert (2000)

*assimilis* (Holmgren, 1858, *Trematopygus*)

*femoralis* Thomson, 1893

***RHORUS*** Förster, 1869

*DOLICHOBLASTUS* Strobl, 1903

***anglicator*** Aubert, 1988 E W S added by Aubert (1988)

***binotatus*** (Kriechbaumer, 1897, *Polyblastus*) E

added by Aubert (2000)

***brunnifemur*** Kasparyan, 2015 E S added by Kasparyan (2015)

***chrysopus*** (Gmelin, 1790, *Ichneumon*) E S

*caproni* (Bridgman, 1882, *Monoblastus*)

*capronii* misspelling

***chrysopygus*** (Roman, 1909, *Monoblastus*) E

***exstirpatorius*** (Gravenhorst, 1829, *Tryphon*) E S

added by Aubert (2000)

*laevigatus* (Holmgren,1856, *Polyblastus*)

*levigatus* (Dalla Torre, 1901, *Monoblastus*)

***fasciatus*** (Gravenhorst, 1829, *Tryphon*) E

***flavopictus*** (Strobl, 1903, *Monoblastus*) E NHM, added here

*braunsi* Habermehl, 1903

***gauldi*** Kasparyan, 2014 E added by Kasparyan (2014)

***lapponicus*** (Roman, 1909, *Monoblastus*) E S

***laricis*** Kasparyan, 2014 S added by Kasparyan (2014)

***longicornis*** (Holmgren, 1858, *Monoblastus*) E S

*glaber* (Bridgman, 1886, *Prionopoda*)

*flavomaculatus* (Strobl, 1903, *Ischyrocnemis*)

Horstmann (2012*c*)

*vitosaensis* (Gregor, 1933, *Monoblastus*)

***longigena*** (Thomson, 1883, *Monoblastus*) S

***neuter*** Aubert, 1988 E added by Aubert (1988)

***nigrifrons*** (Holmgren, 1883, *Polyblastus*) S added by Kasparyan (2014)

***palustris*** (Holmgren, 1857, *Polyblastus*) E S

***punctus*** (Gravenhorst, 1829, *Tryphon*)[[315]](#footnote-316) E

*mesoxanthus* (Gravenhorst, 1829, *Tryphon*)

*scoticus* (Desvignes, 1856, *Tryphon*)

*conspicuus* Kriechbaumer, 1891 Horstmann (2001*d*)

*spectabilis* Kriechbaumer, 1891 Horstmann (2001*d*)

***romani*** Kasparyan, 2014 E added by Kasparyan (2014)

***subfasciatus*** (Stephens, 1835, *Tryphon*) E

***versator*** Aubert, 1994 E S added by Aubert (1994)

Species excluded from the British and Irish list

[***neustriae*** (Schrank, 1802, *Ichneumon*)[[316]](#footnote-317)]

***SYMPHERTA*** Förster, 1869

*ATRESTES* Förster, 1869

*CAMPOGENES* Förster, 1869

*STIPHROSOMUS* Förster, 1869 preocc.

*TRAPEZOCORA* Förster, 1869

*EUSTIPHROSOMUS* Hincks, 1944

***antilope*** (Gravenhorst, 1829, *Mesoleptus*) E W

*irata* (Gravenhorst, 1829, *Tryphon*)

*pulchricornis* (Holmgren, 1857, *Catoglyptus*)

*scabra* (Brischke, 1871, *Catoglyptus*)

***foveolator*** (Holmgren, 1856, *Mesoleptus*) E S NHM, Ely coll., det. Broad & Ely, added here

***obligator*** (Thunberg, 1824, *Ichneumon*) E S

*fuscicornis* (Gmelin, 1790, *Ichneumon*) preocc.

*waltoni* (Curtis, 1837, *Mesoleptus*)

***splendens*** (Strobl, 1903, *Catoglyptus*) E NHM, added here

**?*sulcata*** (Thomson, 1893, *Catoglyptus*) E[[317]](#footnote-318) added by Aubert (2000)

***tenthredinarum*** Horstmann, 1999[[318]](#footnote-319) E S

*ambulator* (Thunberg, 1824, *Ichneumon*) preocc.

***ullrichi*** (Tschek, 1869, *Catoglyptus*) E W I NHM, added here

***SYNTACTUS*** Förster, 1869

*TROMOPOEA* Förster, 1869

*BRISCHKEA* Kriechbaumer, 1897

***delusor*** (Linnaeus, 1758, *Ichneumon*) E S

*trochantericus* (Geoffroy, 1785, *Ichneumon*)

*parvulus* (Kriechbaumer, 1897, *Brischkea*)

***minor*** (Holmgren, 1857, *Catoglyptus*) E

***minutus*** (Bridgman, 1886, *Euryproctus*) E

***TREMATOPYGUS*** Holmgren, 1857

*AMORPHOGNATHON* Förster, 1869

*ASELASMA* Förster, 1869

*CAMPOPORUS* Förster, 1869

*RHIGELUS* Förster, 1869

***horvathi*** (Kiss, 1926, *Polyblastus*)[[319]](#footnote-320) S NHM, det. Horstmann, added here

***melanocerus*** (Gravenhorst, 1829, *Tryphon*) E W S

NHM, added here

*kriechbaumeri* Thomson, 1893

*thalhammeri* Strobl, 1901

*romani* Heinrich, 1929

***nigricornis*** Holmgren, 1857 E

*dictator* (Thunberg, 1824, *Ichneumon*) preocc.

**?*rufator*** Hinz, 1986 E S NHM, det. Horstmann[[320]](#footnote-321), added here

***spiniger*** Hinz, 1976 E

***vellicans*** (Gravenhorst, 1829, *Tryphon*) E S

*bicolor* (Zetterstedt, 1838, *Bassus*)

*ruficornis* Holmgren, 1857 preocc.

Tribe SCOLOBATINI Schmiedeknecht, 1911

***SCOLOBATES*** Gravenhorst, 1829

*AGLYPHUS* Giraud, 1872

*PARABRACONIA* Schmiedeknecht, 1914

***auriculatus*** (Fabricius, 1804, *Ichneumon*) E S W I

*auriculator* (Thunberg, 1824, *Ichneumon*)

*elevator* (Thunberg, 1824, *Ichneumon*) preocc.

*crassitarsus* Gravenhorst, 1829

*hylotomae* Kriechbaumer, 1897

*niger* Roman, 1917

*nigrifacies* Teunissen, 1953

Subfamily CYLLOCERIINAE Wahl, 1990

***ALLOMACRUS*** Förster, 1869

*SIBIRIAKOFFIA* Holmgren, 1880

*KENTROTRYPHON* Strobl, 1903 Schwarz (2003)

***arcticus*** (Holmgren, 1880, *Sibiriakoffia*) E S I NHM, NMS, UM, added here

*pimplarius* Thomson, 1888

***CYLLOCERIA*** Schiødte, 1838[[321]](#footnote-322)

*CHALINOCERUS* Ratzeburg, 1852

*ASPHRAGIS* Förster, 1869

***caligata*** (Gravenhorst, 1829, *Phytodietus*)[[322]](#footnote-323) E I

*nunciator* misident.

*nigra* (Gravenhorst, 1829, *Phytodietus*)

*crenicornis* (Curtis, 1832, *Lampronota*)

*nuntiator* (Zetterstedt, 1838, *Bassus*)

*manca* (Ruthe, 1855, *Chalinocerus*)

***melancholica*** (Gravenhorst, 1820, *Ichneumon*) E S I

*accusator* misidentification

*defectiva* (Gravenhorst, 1829, *Lissonota*)

*affinis* (Zetterstedt, 1838, *Bassus*)

*marginator* Schiødte, 1838

*denticornis* (Haliday, 1839, *Lampronota*)

*fracticornis* (Haliday, 1839, *Lampronota*)

*longicornis* (Ratzeburg, 1852, *Chalinocerus*)

*marginatrix* (Schulz, 1906, *Lampronota*)

*rugulosa* (Haupt, 1917, *Tropistes*)

*altior* (Heinrich, 1953, *Chalinocerus*)

***sylvestris*** (Gravenhorst, 1829, *Tryphon*) E NHM, det. Broad, added here

*striolata* (Hellén, 1915, *Lampronota*)

***HYPERACMUS*** Holmgren, 1858[[323]](#footnote-324)

*CUSHMANIA* Dasch, 1992 Wahl & Gauld (1998)

***crassicornis*** (Gravenhorst, 1829, *Exochus*) E S I

*brunniventris* (Rudow, 1883, *Exochus*)

*suerinensis* (Brauns, 1905, *Lampronota*) Humala (2002)

Subfamily DIACRITINAE Townes, 1965[[324]](#footnote-325)

***DIACRITUS*** Förster, 1869

*PHIDIAS* Vollenhoven, 1878 preocc.

*STENOLABIS* Kriechbaumer, 1894

*PHOSPHORIANA* Rossem, 1987 Humala (2007)

*PHOSPHORUS* Rossem, 1981 preocc.

***aciculatus*** (Vollenhoven, 1878, *Phidias*) E S I

*cingulatus* (Kriechbaumer, 1894, *Stenolabis*)

*rugosissima* (Strobl, 1904, *Entypoma*) Humala (2007)

Subfamily DIPLAZONTINAE Viereck, 1918[[325]](#footnote-326)

***BIOBLAPSIS*** Förster, 1869

*TRICHOMASTIX* Vollenhoven, 1878

***cultiformis*** (Davis, 1897, *Otoblastus*) E S added by Rotheray (1990)

*mallochi* Rotheray, 1990 Klopfstein (2014)

***polita*** (Vollenhoven, 1878, *Trichomastix*) E S

*flavipes* (Holmgren, 1858, *Bassus*) preocc.

*tibialis* (Bridgman, 1883, *Bassus*) preocc.

***CAMPOCRASPEDON*** Uchida, 1957

***annulitarsis*** (Hedwig, 1838, *Homocidus*) E S W I

*arcanus* (Stelfox, 1941, *Homocidus*)

***caudatus*** (Thomson, 1890, *Homotropus*) E S W I M

***DIPLAZON*** Nees, 1819

*BASSUS* misident.

***albotibialis*** Dasch, 1964 E S I

*alpinus* (Holmgren, 1858, *Bassus*) preocc.

Klopfstein (2014)

*neoalpinus* Zwakhals, 1979 Klopfstein (2014)

***annulatus*** (Gravenhorst, 1829, *Bassus*)[[326]](#footnote-327) E S W I M

*lapponicus* (Zetterstedt, 1838, *Bassus*)

***deletus*** (Thomson, 1890, *Bassus*) E S W I

*rufigaster* Dasch, 1964

***laetatorius*** (Fabricius, 1781, *Ichneumon*) E S W I M

*dichrous* (Schrank, 1781, *Ichneumon*)

*albovarius* (Wollaston, 1858, *Bassus*)

*cinctipes* (Holmgren, 1868, *Bassus*)

*varipes* (Smith, 1878, *Scolobates*)

*venustulus* (Saussure, 1892, *Bassus*)

*balearicus* (Kriechbaumer, 1894, *Bassus*)

***pectoratorius*** (Thunberg, 1824, *Ichneumon*) E S W I M

*angustorius* Thunberg, 1824, *Ichneumon*)

*pectoratorius* (Gravenhorst, 1829, *Bassus*) preocc.

*nigrithorax* (Strobl, 1902, *Homotropus*)

*akaashii* (Uchida, 1931, *Homocidus*)

*urupensis* (Uchida, 1935, *Bassus*)

***scutatorius*** Teunissen, 1943 E added by Thirion (1987)

*pilosus* Uchida, 1957

*tetragonopsis* Uchida, 1957[[327]](#footnote-328)

***tetragonus*** (Thunberg, 1824, *Ichneumon*) E S W I M

*hortorius* (Thunberg, 1824, *Ichneumon*)

*ustorius* (Thunberg, 1824, *Ichneumon*)

*tricinctus* (Gravenhorst, 1829, *Bassus*)

*nemoralis* (Holmgren, 1858, *Bassus*)

***tibiatorius*** (Thunberg, 1824, *Ichneumon*) E S W I M

Askew (2000)

*albosignatus* (Gravenhorst, 1829, *Bassus*)

***varicoxa*** (Thomson, 1890, *Bassus*) E S I M

***ENIZEMUM*** Förster, 1869

***nigricorne*** (Thomson, 1890, *Homotropus*) I[[328]](#footnote-329)

***ornatum*** (Gravenhorst, 1829, *Bassus*) E S W I M

*deplanatum* (Gravenhorst, 1829, *Bassus*)

*carinulatum* (Ruthe, 1859, *Bassus*)

*frenator* (Desvignes, 1862, *Bassus*)

*sumptuosum* (Schmiedeknecht, 1926, *Homocidus*)

***scutellare*** (Lange, 1911, *Homotropus*) W added by Klopfstein (2014)

*albopictum* (Lange, 1911, *Homotropus*) preocc.

*rubiginosum* (Schmiedeknecht, 1926, *Homocidus*)

Klopfstein (2014)

***tridentatum*** Dasch, 1964 E I NHM, det. Klopfstein, Broad, added here

***EURYTYLOIDES*** Nakanishi, 1978

***umbrinus*** Klopfstein, 2014 E added by Klopfstein (2014)

***FOSSATYLOIDES*** Klopfstein, Quicke, Kropf & Frick, 2011

***gracilentus*** (Holmgren, 1858, *Bassus*) E S W

*pulcher* (Holmgren, 1858, *Bassus*) preocc.

***HOMOTROPUS*** Förster, 1869[[329]](#footnote-330)

*HOMOCIDUS* Morley, 1911

***collinus*** (Stelfox, 1941, *Homocidus*) E S I

*simulans* (Stelfox, 1941, *Homocidus*)

***crassicornis*** Thomson, 1890 E S W M

*brevicornis* Thomson, 1890

*asyntactus* (Schmiedeknecht, 1926, *Homocidus*)

***dimidiatus*** (Schrank, 1802, *Ichneumon*) E S

*planus* (Desvignes, 1862, *Bassus*)

*crassicrus* Thomson, 1890 E S W Klopfstein (2014)

***elegans*** (Gravenhorst, 1829, *Bassus*) E S W M

*rufonotatus* (Holmgren, 1858, *Bassus*)

*affinis* Szépligeti, 1898

***frontorius*** (Thunberg, 1824, *Ichneumon*) E S W I

*subopacus* (Stelfox, 1941, *Homocidus*) Klopfstein (2014)

***haemorrhoidalis*** Szépligeti, 1898 E S W I

*rhenanus* (Habermehl, 1930, *Homocidus*)

*struvei* (Hedwig, 1939, *Homocidus*)

*tricolor* (Stelfox, 1941, *Homocidus*)

*lipothrix* Momoi, 1973

***longiventris*** Thomson, 1890 E S I

***megaspis*** Thomson, 1890 E S I

*megalaspis* Schulz, 1906

***melanogaster*** (Holmgren, 1872, *Bassus*) E added by Klopfstein (2014)

***nigritarsus*** (Gravenhorst, 1829, *Bassus*) E S W I M

*picitans* (Desvignes, 1862, *Bassus*)

*groenlandicus* (Holmgren, 1872, *Bassus*)

***pallipes*** (Gravenhorst, 1829, *Bassus*) E S I M

*pectoralis* (Gravenhorst, 1829, *Lissonota*)

*pallidipes* (Marshall, 1872, *Bassus*)

*pallidipennis* Dalla Torre, 1901

*pallidipes* Dalla Torre, 1901

***pectoralis*** (Provancher, 1874, *Bassus*) E S W

*incisus* Thomson, 1890 Klopfstein (2014)

*reflexus* Morley, 1906 Klopfstein (2014)

***pictus*** (Gravenhorst, 1829, *Bassus*) E S W I M

*nigricornis* (Zetterstedt, 1838, *Tryphon*) Klopfstein (2014)

*pumilus* (Holmgren, 1858, *Bassus*)

*thoracicus* (Desvignes, 1862, *Bassus*)

*brevis* (Hedwig, 1938, *Homocidus*)

***signatus*** (Gravenhorst, 1829, *Bassus*) E S I M

*hygrobius* Thomson, 1890

*bifoveolatus* Kriechbaumer, 1894

***strigator*** (Fabricius, 1793, *Ichneumon*) E W

*ruficornis* (Holmgren, 1858, *Bassus*) preocc.

***sundevalli*** (Holmgren, 1858, *Bassus*) E S W Rotheray (1986)

*scabrosus* (Desvignes, 1862, *Bassus*)

species *incertae sedis* within *Homotropus*

[***impolitus*** (Stelfox, 1941, *Homocidus*)[[330]](#footnote-331) S]

***PHTHORIMA*** Förster, 1869[[331]](#footnote-332)

*PHTHORIMUS* Thomson, 1890

***compressa*** (Desvignes, 1856, *Bassus*) E S W I

*ibalioidis* (Kriechbaumer, 1878, *Bassus*)

*nigra* (Morley, 1906, *Homotropus*)

***picta*** (Habermehl, 1925, *Phthorimus*) E I added by Fitton & Boston (1988)

*gaullei* Seyrig, 1928

***xanthaspis*** (Thomson, 1890, *Homotropus*) E added by Fitton & Boston (1988)

***PROMETHES*** Förster, 1869

*LIOPSIS* Förster, 1869

*PROMETHUS* Thomson, 1890

***bridgmani*** Fitton, 1976 E S W I

*scutellaris* (Bridgman, 1886, *Bassus*) preocc.

***sulcator*** (Gravenhorst, 1829, *Bassus*) E S W I M

*areolatus* (Holmgren, 1859, *Bassus*)

*anomalus* (Taschenberg, 1865, *Orthopelma*)

*dodsi* (Morley, 1906, *Promethus*)

***SUSSABA*** Cameron, 1909

***cognata*** (Holmgren, 1858, *Bassus*) E S W I M

*albicoxa* (Thomson, 1890, *Promethus*)

***dorsalis*** (Holmgren, 1858, *Bassus*) E S W I

*maculata* (Desvignes, 1862, *Bassus*)

***erigator*** (Fabricius, 1793, *Ichneumon*) E[[332]](#footnote-333)

*festiva* (Fabricius, 1798, *Ichneumon*)

*festivator* (Fabricius, 1804, *Ophion*)

***flavipes*** (Lucas, 1849, *Bassus*)[[333]](#footnote-334) E S W I

*pulchella* misident.

*neopulchella* Diller, 1980

*coriacea* Dasch, 1964

***placita*** Dasch, 1964[[334]](#footnote-335) E

*punctiventris* misident. Klopfstein (2014)

***pulchella*** (Holmgren, 1858, *Bassus*)[[335]](#footnote-336) E S W I M

*elongata* (Provancher, 1874, *Bassus*)

*monticola* (Vollenhoven, 1880, *Bassus*)

*laticarpus* (Thomson, 1890, *Promethus*)

*ruthei* (Roman, 1931, *Promethes*)

***SYRPHOCTONUS*** Förster, 1869[[336]](#footnote-337)

***desvignesii*** (Marshall, 1870, *Bassus*) E S W I M

*pulcher* misident.

*pulchellus* (Desvignes, 1862, *Bassus*) preocc.

*neopulcher* Horstmann, 1968 Klopfstein (2014)

***fissorius*** (Gravenhorst, 1829, *Bassus*) E S W I M

*punctatus* (Bridgman, 1887, *Bassus*)

*similis* (Lange, 1911, *Homotropus*)

***tarsatorius*** (Panzer, 1809, *Bassus*) E S W I M

*exsultans* (Gravenhorst, 1829, *Bassus*)

*insignis* (Gravenhorst, 1829, *Bassus*)

*flavus* (Desvignes, 1862, *Bassus*)

*indicus* (Cameron, 1909, *Bassus*)

*eximius* (Habermehl, 1922, *Homotropus*) Klopfstein (2014)

*flavitrochanterus* (Uchida, 1957, *Homotropus*)

***SYRPHOPHILUS*** Dasch, 1964

***bizonarius*** (Gravenhorst, 1829, *Bassus*) E S W I

*cingulatus* (Holmgren, 1858, *Bassus*)

*frontalis* (Brischke, 1878, *Bassus*) preocc.

*iwatensis* (Uchida, 1930, *Homocidus*)

*satoi* (Uchida, 1930, *Homocidus*)

***tricinctorius*** (Thunberg, 1824, *Ichneumon*) E S W I M

Askew (2000)

*cinctus* (Gravenhorst, 1829, *Bassus*)

*lateralis* (Gravenhorst, 1829, *Bassus*)

*albicinctus* (Desvignes, 1862, *Bassus*)

*takaozanus* (Uchida, 1930, *Homocidus*)

***TYMMOPHORUS*** Schmiedeknecht, 1913

*ZOOTREPHES* misident.

***erythrozonus*** (Förster, 1850, *Tryphon*) E

*rufiventris* (Gravenhorst, 1829, *Bassus*) preocc.

Horstmann (2006*d*)

*holmgreni* (Bridgman, 1882, *Bassus*)

*lacustris* Schmiedeknecht, 1913

***obscuripes*** (Holmgren, 1858, *Bassus*) E S W I M

*graculus* misident.[[337]](#footnote-338)

*rufocinctus* (Desvignes, 1862, *Bassus*)

*arcticus* (Holmgren, 1869, *Bassus*)

*luctuosus* (Schmiedeknecht, 1926, *Promethes*)

***suspiciosus*** (Brischke, 1871, *Bassus*)[[338]](#footnote-339) E S I

***WOLDSTEDTIUS*** Carlson, 1979

*SYRPHOCTONUS* misident.

***bauri*** Klopfstein, 2014 E added by Klopfstein (2014)

***biguttatus*** (Gravenhorst, 1829, *Bassus*) E S W I

*rufipes* (Gravenhorst, 1829, *Bassus*)

*confusus* (Woldstedt, 1874, *Bassus*)

***citropectoralis*** (Schmiedeknecht, 1926, *Homocidus*) E S I

*abdominator* (Bridgman, 1886, *Bassus*) preocc.

***flavolineatus*** (Gravenhorst, 1829, *Bassus*) E S W

*bimaculatus* (Holmgren, 1858, *Bassus*)

*interruptus* (Holmgren, 1858, *Bassus*)

***holarcticus*** (Diller, 1969, *Syrphoctonus*) E added by Klopfstein (2014)

***XESTOPELTA*** Dasch, 1964

***gracillima*** (Schmiedeknecht, 1926, *Promethes*) E

added by Fitton & Rotheray (1982)

*amabilis* (Habermehl, 1935, *Homocidus*)

Subfamily EUCEROTINAE Viereck, 1919[[339]](#footnote-340)

***EUCEROS*** Gravenhorst, 1829

*EUMESIUS* Westwood, 1840

*OMALOCEROS* Giraud, 1857

*TAUTOZELUS* Förster, 1869

***albitarsus*** Curtis, 1837 E I

*dimidiatus* Brullé, 1846

***pruinosus*** (Gravenhorst, 1829, *Tryphon*)[[340]](#footnote-341) E S

*crassicornis* Gravenhorst, 1829

*morionellus* Holmgren, 1857

*unifasciatus* Vollenhoven, 1878

*castaneus* (Pfankuch, 1906, *Eumesius*) unavailable

***serricornis*** Haliday, 1839 E S I

*egregius* Holmgren, 1857

*grandicornis* Holmgren, 1857

Subfamily HYBRIZONTINAE Blanchard, 1845[[341]](#footnote-342)

PAXYLLOMATINAE Förster, 1862

***GHILAROMMA*** Tobias, 1988

***fuliginosi*** (Donisthorpe & Wilkinson, 1930, *Paxylomma*) E

***HYBRIZON*** Fallén, 1813

*PAXYLLOMA* Latreille, 1817

*PLANCUS* Curtis, 1833

*PACHYLOMMA* Ratzeburg, 1848

***buccatus*** (de Brébisson, 1825, *Paxylomma*)[[342]](#footnote-343) E W

*apicalis* (Curtis, 1833, *Plancus*)

*latebricola* Nees, 1834

Subfamily ICHNEUMONINAE Latreille, 1802[[343]](#footnote-344)

Tribe EURYLABINI Heinrich, 1934

***EURYLABUS*** Wesmael, 1845

*MISCHOPHORUS* Kriechbaumer, 1894

***larvatus*** (Christ, 1791, *Ichneumon*) E

*vinulatorius* (Thunberg, 1824, *Ichneumon*)

*intrepidus* Wesmael, 1855

*pestrei* (Berthoumieu, 1892, *Catadelphus*)

*flavosignatus* (Kriechbaumer, 1894, *Mischophorus*)

*vinulator* Thomson, 1894

*dusmeti* (Berthoumieu, 1904, *Catadelphus*)

***torvus*** Wesmael, 1845 E W I

***tristis*** (Gravenhorst, 1829, *Ichneumon*) E W I

*corvinus* Wesmael, 1845

Tribe GOEDARTIINI Townes, 1961

***GOEDARTIA*** Boie, 1841

*AUTOMALUS* Wesmael, 1845

***alboguttata*** (Gravenhorst, 1829, *Trogus*) E W[[344]](#footnote-345) I

*affinis* (Boie, 1841, *Trogus*)

*baltica* (Ratzeburg, 1844, *Ichneumon*)

*dimidiativentris* (Rudow, 1888, *Amblyteles*)

Tribe HERESIARCHINI Ashmead, 1900

PROTICHNEUMONINI Heinrich, 1934

CALLAJOPPINI Heinrich, 1962 Sime & Wahl (2002)

TROGINI Förster, 1869 preocc. Sime & Wahl (2002)

***AMBLYJOPPA*** Cameron, 1902

***fuscipennis*** (Wesmael, 1845, *Amblyteles*) E W I

***proteus*** (Christ, 1791, *Ichneumon*) E S I

*laminatoria* (Fabricius, 1798, *Ichneumon*)

*nigratoria* (Fabricius, 1798, *Ichneumon*) preocc.

*bilineator* (Donovan, 1810, *Ichneumon*) Horstmann (1997)

*nigriculus* (Walkley, 1958, *Ichneumon*)

***CALLAJOPPA*** Cameron, 1903

***cirrogaster*** (Schrank, 1781, *Ichneumon*) E I

*cirrogastra* misspelling

*nigrocaudata* (Retzius, 1783, *Ichneumon*)

*crocata* (Geoffroy, 1785, *Ichneumon*)

*lutoria* (Fabricius, 1787, *Ichneumon*)

*rubricornuta* (Christ, 1791, *Ichneumon*)

*dessinator* (Olivier, 1792, *Ichneumon*)

*scutellaris* (Olivier, 1792, *Ichneumon*)

*imperatoria* (Panzer, 1804, *Ichneumon*)

*obscuratoria* (Gravenhorst, 1807, *Ichneumon*)

*atrocaudata* (Stephens, 1835, *Trogus*)

*excellens* (Tischbein, 1882, *Trogus*)

***exaltatoria*** (Panzer, 1804, *Ichneumon*) E

*latoria* (Thunberg, 1824, *Ichneumon*)

*atropos* (Curtis, 1828, *Ichneumon*)

***COELICHNEUMON*** Thomson, 1893

***anthrax*** (Dalla Torre, 1901, *Ichneumon*) E added by Riedel (2012)

*anthracinus* (Holmgren, 1864, *Ichneumon*) preocc.

***biannulatus*** (Gravenhorst, 1820, *Ichneumon*)[[345]](#footnote-346) E W

*auspex* misident.

*fasciatus* (Gmelin, 1790, *Ichneumon*) preocc.

*leucopis* (Berthoumieu, 1894, *Ichneumon*) preocc., unavailable

***biguttorius*** (Thunberg, 1789, *Ichneumon*) E

*microstictus* misident. Horstmann (2002*a*)

*serenus* (Gravenhorst, 1829, *Ichneumon*) Riedel (2012)

*restaurator* (Fabricius, 1793, *Ichneumon*) preocc.

*restritutor* (Thunberg, 1824, *Ichneumon*)

*laticeps* (Rudow, 1888, *Amblyteles*)

*rufiapicalis* (Pic, 1914, *Ichneumon*)

*transsylvanicus* (Kiss, 1924, *Ichneumon*) Riedel (2012)

*concolor* Heinrich, 1949 Riedel (2012)

***bilineatus*** (Gmelin, 1790, *Ichneumon*) E I

*pulsator* (Panzer, 1804, *Ichneumon*)

***comitator*** (Linnaeus, 1758, *Ichneumon*) E I

*auspex* (Müller, 1776, *Ichneumon*)

*biguttatus* (Thunberg, 1784, *Ichneumon*)

*tripunctorius* (Thunberg, 1789, *Ichneumon*)

*nigrator* (Fabricius, 1793, *Ichneumon*) preocc.

*narrator* (Fabricius, 1804, *Ichneumon*)

*restaurator* (Gravenhorst, 1820, *Ichneumon*) preocc.

Horstmann (1998*b*)

*fuscatorius* (Thunberg, 1824, *Ichneumon*)

*ferreus* (Gravenhorst, 1829, *Ichneumon*)

*coerulescens* (Tischbein, 1879, *Ichneumon*)

*purpurissatus* Perkins, 1953 Horstmann (2000*b*)

***consimilis*** (Wesmael, 1845, *Ichneumon*) E W I

*caelareator* (Tischbein, 1881, *Ichneumon*)

*nigripes* (Kriechbaumer, 1894, *Ichneumon*) preocc., unavailable

***cyaniventris*** (Wesmael, 1859, *Ichneumon*) E W I

O'Connor (2004*a*)

*biobliteratus* (Pic, 1923, *Ichneumon*)

*multialbonotatus* (Pic, 1923, *Ichneumon*)

***desinatorius*** (Thunberg, 1824, *Ichneumon*) E S

*fuscipes* (Gmelin, 1790, *Ichneumon*) preocc.

*subguttatus* (Gravenhorst, 1829, *Ichneumon*)

***falsificus*** (Wesmael, 1845, *Ichneumon*) E

*specularis* (Tischbein, 1881, *Ichneumon*) Riedel (2012)

*chevrieri* (Pic, 1902, *Ichneumon*)

***haemorrhoidalis*** (Gravenhorst, 1820, *Ichneumon*) E I

*castaniventris* (Gravenhorst, 1829, *Ichneumon*)

Riedel (2012)

*castanicauda* (Tischbein, 1881, *Ichneumon*)

*truncatulus* (Thomson, 1886)

*secretus* (Berthoumieu, 1894, *Ichneumon*) unavailable

*subniger* (Berthoumieu, 1894, *Ichneumon*) unavailable

*strandi* (Berthoumieu, 1910, *Ichneumon*)

*binigronotatus* (Pic, 1925, *Ichneumon*)

*vulcanius* (Pic, 1925, *Ichneumon*)

*bipunctatus* (Schmiedeknecht, 1928, *Ichneumon*) preocc.

***leucocerus*** (Gravenhorst, 1820, *Ichneumon*) E I

*solitarius* (Thunberg, 1824, *Ichneumon*)

*ligeris* (Pic, 1923, *Ichneumon*)

***litoralis*** Horstmann, 2000 E Horstmann (2000*b*)

*purpurissatus* misident. Horstmann (2000*b*)

***nigerrimus*** (Stephens, 1835, *Ichneumon*) E S W I

*derasus* (Wesmael, 1845, *Ichneumon*)

*carbonator* (Tischhbein, 1874, *Amblyteles*)

*percussor* (Tischbein, 1876, *Ichneumon*) Riedel (2012)

*minor* (Kriechbaumer, 1894, *Ichneumon*) preocc., unavailable

*annulatus* Heinrich, 1929

***oltenensis*** Constantineanu, Pîrvescu & Mihalache, 1979[[346]](#footnote-347)

added by Horstmann (2002*a*)

*serenus* misident. Horstmann (2002*a*)

***orbitator*** (Thunberg, 1824, *Ichneumon*) E

*microstictus* (Gravenhorst, 1829, *Ichneumon*)

Horstmann (2002*a*)

*melanopyrrhus* (Stephens, 1835, *Ichneumon*)

*separator* (Fonscolombe, 1847, *Ichneumon*)

Riedel (2012)

*liocnemis* (Thomson, 1888, *Ichneumon*)

***ruficauda*** (Wesmael, 1845, *Ichneumon*) E

***validus*** (Berthoumieu, 1894, *Ichneumon*) E S

*nigricornis* (Wesmael, 1845, *Ichneumon*) preocc.

species excluded from the British and Irish list

[***eximius*** (Stephens, 1835, *Ichneumon*)[[347]](#footnote-348)]

***COELICHNEUMONOPS*** Heinrich, 1958

***solutus*** (Holmgren, 1864, *Ichneumon*) S

*chrysostomus* (Thomson, 1896, *Ichneumon*)

Horstmann (1999*a*)

*pictus* (Roman, 1904, *Ichneumon*) preocc.

***HERESIARCHES*** Wesmael, 1859

***eudoxius*** (Wesmael, 1845, *Hepiopelmus*) E

***LYMANTRICHNEUMON*** Heinrich, 1978

***disparis*** (Poda, 1761, *Sphex*) E added by Broad & Davis (2015)

***PROTICHNEUMON*** Thomson, 1893

***pisorius*** (Linnaeus, 1758, *Ichneumon*) E I

*fusorius* misident.

*lentorius* (Panzer, 1799, *Ichneumon*)

*fugatorius* (Panzer, 1804, *Ichneumon*)

*mediofulvus* (Berthoumieu, 1894, *Ichneumon*) unavailable

*dorsoniger* Roman,1910

***similatorius*** (Fabricius, 1798, *Ichneumon*)[[348]](#footnote-349) E

?*exspectorius* (Fabricius, 1794, *Ichneumon*)

*erythrogaster* (Stephens, 1835, *Ichneumon*) preocc.

*coqueberti* (Wesmael, 1848, *Ichneumon*)

*dorsoniger* (Berthoumieu, 1894, *Ichneumon*)

***PSILOMASTAX*** Tischbein, 1868

*CERCODINOTOMUS* Uchida, 1940

***pyramidalis*** Tischbein, 1868 E

*pictus* Kriechbaumer, 1882

***SYSPASIS*** Townes, 1965

***carinator*** (Fabricius, 1798, *Ichneumon*) E NMS, det. Riedel, added here

*helleri* (Holmgren, 1878, *Ichneumon*)

*rufipes* (Strobl, 1901, *Ichneumon*) preocc.

*tenuidens* (Berthoumieu, 1904, *Ichneumon*)

***lineator*** (Fabricius, 1781, *Ichneumon*) E S I M

*trilineata* (Gmelin, 1790, *Ichneumon*)

*umbraculosa* (Gravenhorst, 1829, *Ichneumon*)

*binotata* (Stephens, 1835, *Ichneumon*)

*brischkii* (Ratzeburg, 1852, *Ichneumon*)

*adulator* (Tischbein, 1881, *Ichneumon*)

*calculosa* (Berthoumieu, 1903, *Ichneumon*)

***rufina*** (Gravenhorst, 1820, *Ichneumon*) E

?*judex* (Müller, 1776, *Ichneumon*)

***scutellator*** (Gravenhorst, 1829, *Ichneumon*) E

*rufescens* (Berthoumieu, 1894, *Ichneumon*) preocc., unavailable

***TROGUS*** Panzer, 1806

*DINOTOMUS* Förster, 1869

***lapidator*** (Fabricius, 1787, *Ichneumon*)[[349]](#footnote-350) E Shaw (1978)

*anthracinus* (Scopoli, 1763, *Sphex*) *nom. ob.*

(Horstmann, 2001*b*)

*coerulator* (Weber, 1795, *Ichneumon*)

*coerulator* (Fabricius, 1804, *Ichneumon*) preocc.

*saxator* (Thunberg, 1824, *Ichneumon*)

*fuscipennis* Gravenhorst, 1829 Wahl & Sime (2006)

*violaceus* (Mocsáry,1883, *Psilomastax*) Wahl & Sime (2006)

*cyaneipennis* Costa,1886 Wahl & Sime (2006)

*cyaneus* (Kriechbaumer,1892, *Psilomastax*)

Wahl & Sime (2006)

*romani* Uchida,1942 Wahl & Sime (2006)

*brevicaudae* Heinrich,1975 Wahl & Sime (2006)

*panzeri* Carlson,1975 Wahl & Sime (2006)

Tribe ICHNEUMONINI Latreille, 1802

JOPPINI Kriechbaumer, 1898

***ACHAIUS*** Cameron, 1903

***margineguttatus*** (Gravenhorst, 1829, *Ichneumon*) E S

*novitius* (Wesmael, 1854, *Amblyteles*)

*luteosignatus* (Pic, 1914, *Amblyteles*)

***oratorius*** (Fabricius, 1793, *Ichneumon*) E S W I M

?*dealbatus* (Gmelin, 1790, *Ichneumon*)

*cingulatorius* (Weber, 1801, *Ichneumon*) Horstmann (1997)

*atramentarius* (Gravenhorst, 1829, *Ichneumon*)

*cingulipes* (Stephens, 1835, *Ichneumon*)

*bipunctus* (Berthoumieu, 1896, *Amblyteles*) unavailable

*theresae* (Pic, 1897, *Amblyteles*)

*albocingulatus* (Strobl, 1901, *Ichneumon*)

*marginalis* (Habermehl, 1903, *Amblyteles*)

*bellus* (Habermehl, 1917, *Spiloteles*)

***ACOLOBUS*** Wesmael, 1845

***albimanus*** (Gravenhorst, 1829, *Ichneumon*)

*buyssoni* (Berthoumieu, 1892, *Ichneumon*)

***sericeus*** Wesmael, 1845 E

***AMBLYTELES*** Wesmael, 1845[[350]](#footnote-351)

***armatorius*** (Forster, 1771, *Ichneumon*) E S I M

*fasciatorius* (Fabricius, 1775, *Ichneumon*)

*notatorius* (Villers, 1789, *Ichneumon*)

*dimicatorius* (Gmelin, 1790, *Ichneumon*)

*signatorius* (Olivier, 1792, *Ichneumon*)

*diversorius* (Stephens, 1835, *Ichneumon*)

*regius* Tischbein, 1868

***AOPLUS*** Tischbein, 1874

***altercator*** (Wesmael, 1855, *Ichneumon*) E S I

***castaneus*** (Gravenhorst, 1820, *Ichneumon*) E S I

*rufoniger* (Tischbein, 1881, *Exephanes*)

*mesopyrrhus* (Kriechbaumer, 1893, *Ichneumon*)

Hinz & Horstmann (2000)

*subniger* (Berthoumieu, 1894, *Ichneumon*) unavailable

*fieschensis* (Pic, 1926, *Ichneumon*)

*royatensis* (Pic, 1926, *Ichneumon*)

***defraudator*** (Wesmael, 1845, *Ichneumon*) S I

*angustus* (Tischbein, 1863, *Ichneumon*)

*jemilleri* (Kriechbaumer, 1893, *Ichneumon*)

Hinz & Horstmann (2000)

*sabaudus* (Berthoumieu, 1904, *Ichneumon*)

***ochropis*** (Gmelin, 1790, *Ichneumon*) E S I

*ephippium* (Rudow, 1886, *Cryptus*)

***rubricosus*** (Holmgren, 1864, *Ichneumon*) E

***ruficeps*** (Gravenhorst, 1829, *Ichneumon*) E S I

*leucocrepis* (Wesmael, 1857, *Ichneumon*)

*maximorufus* (Pic, 1927, *Ichneumon*)

***BARANISOBAS*** Heinrich, 1972

***ridibundus*** (Gravenhorst, 1829, *Ichneumon*) E I

*hassicus* (Ratzeburg, 1848, *Ichneumon*)

*variegator* (Tischbein, 1881, *Exephanes*) Hinz & Horstmann (2000)

*polystictus* (Kriechbaumer, 1887, *Ichneumon*)

*instabilis* (Berthoumieu, 1897, *Ichneumon*) preocc.

*evianensis* (Pic, 1902, *Ichneumon*)

*insperatus* (Dalla Torre, 1902, *Ichneumon*)

*fallaciosus* (Berthoumieu, 1903, *Ichneumon*)

*bulsanensis* (Smits van Burgst, 1914, *Anisobas*)

***BARICHNEUMON*** Thomson, 1893[[351]](#footnote-352)

***anator*** (Fabricius, 1793, *Ichneumon*) E I

?*biscutatus* (Gmelin, 1790, *Ichneumon*)

*bulimorius* (Thunberg, 1824, *Ichneumon*)

*dealbator* (Thunberg, 1824, *Ichneumon*)

*femoratorius* (Thunberg, 1824, *Ichneumon*)

*retusorius* (Thunberg, 1824, *Ichneumon*)

*henschi* (Schmiedeknecht, 1929, *Ichneumon*)

***bilunulatus*** (Gravenhorst, 1829, *Ichneumon*) E I

*sexlineatus* (Gravenhorst, 1829, *Ichneumon*)

*piniperdae* (Hartig, 1838, *Phygadeuon*)

*troscheli* (Ratzeburg, 1844, *Ichneumon*)

*imitator* (Kriechbaumer, 1882, *Ichneumon*) preocc.

*moraguesi* (Kriechbaumer, 1894, *Ichneumon*)

***chionomus*** (Wesmael, 1845, *Ichneumon*) E S I

***derogator*** (Wesmael, 1845, *Ichneumon*) E

***gemellus*** (Gravenhorst, 1829, *Ichneumon*) E S I M

*inversus* (Kriechbaumer, 1893, *Ichneumon*) preocc.

*carri* Habermehl, 1923

*controversus* (Schmiedeknecht, 1928, *Ichneumon*)

*rubricans* (Schmiedeknecht, 1929, *Ichneumon*)

*semirufus* (Schmiedeknecht, 1929, *Ichneumon*) preocc.

*constantineanui* (Heinrich, 1972, *Stenobarichneumon*)

***heracliana*** (Bridgman, 1884, *Ichneumon*) E M

***peregrinator*** (Linnaeus, 1758, *Ichneumon*) E S I M

*scriptorius* (Thunberg, 1824, *Ichneumon*)

*vacillatorius* (Gravenhorst, 1829, *Ichneumon*) preocc.

***plagiarius*** (Wesmael, 1848, *Ichneumon*)

*merkli* (Kiss, 1915, *Plectocryptus*) Horstmann (2008*c*)

***praeceptor*** (Thunberg, 1824, *Ichneumon*) E S

*procerus* (Gravenhorst, 1829, *Ichneumon*)

*derivator* (Wesmael, 1845, *Ichneumon*)

*lunuliger* (Kriechbaumer, 1890, *Ichneumon*)

Horstmann (2006*b*)

*kervillei* (Berthoumieu, 1903, *Ichneumon*)

*cenisiensis* (Berthoumieu, 1906, *Ichneumon*)

*atricornis* (Pic, 1926, *Ichneumon*)

***CHASMIAS*** Ashmead, 1900

*CHASMODES* Wesmael, 1845

***motatorius*** (Fabricius, 1775, *Ichneumon*) E I M

*importunus* (Tischbein, 1874, *Ichneumon*)

*transitorius* (Berthoumieu, 1894, *Chasmodes*) unavailable

*atronotatus* Pic, 1917

*berthoumieui* Pic, 1917

*bicoloripes* Pic, 1917

*diversipes* Pic, 1917

*rufonotatus* Pic, 1917

***paludator*** (Desvignes, 1854, *Ichneumon*) E

*paludicola* (Wesmael, 1857, *Chasmodes*)

*dissimulator* (Tischbein, 1881, *Ichneumon*)

***CRATICHNEUMON*** Thomson, 1893

***albifrons*** (Stephens, 1835, *Ichneumon*) E I

*gravenhorstii* (Fonscolombe, 1847, *Ichneumon*)

*grandiceps* (Thomson, 1887, *Ichneumon*)

***coruscator*** (Linnaeus, 1758, *Ichneumon*) E W I

*corruscator* misspelling

*ambulator* (Müller, 1774, *Ichneumon*)

*alacer* (Gravenhorst, 1829, *Ichneumon*)

*luridus* (Gravenhorst, 1829, *Ichneumon*)

*gasterator* (Stephens, 1835, *Ichneumon*)

*metaxanthus* (Hartig, 1838, *Ichneumon*)

*binotatus* (Desvignes, 1856, *Ichneumon*) preocc.

*pyrenaeus* (Tischbein, 1882, *Ichneumon*)

***culex*** (Müller, 1776, *Ichneumon*) E S W I

*tibialis* (Geoffroy, 1785, *Ichneumon*)

*clavipes* (Gmelin, 1790, *Ichneumon*)

*leucostoma* (Gmelin, 1790, *Ichneumon*)

*quadricolor* (Gmelin, 1790, *Ichneumon*)

*versicolor* (Gmelin, 1790, *Ichneumon*)

*annulator* (Fabricius, 1793, *Ichneumon*) preocc.

*fabricator* (Fabricius, 1793, *Ichneumon*) Horstmann (2001*b*)

*crassator* (Thunberg, 1824, *Ichneumon*)

*infestor* (Thunberg, 1824, *Ichneumon*)

*viator* (Thunberg, 1824, *Ichneumon*)

*fulvipes* (Stephens, 1835, *Ichneumon*)

*ruficoxis* Constantineanu, Andriescu & Ciochia, 1956

***flavifrons*** (Schrank, 1781, *Ichneumon*) E S I

*fabricator* misident. Horstmann (2001*b*)

*frontalis* (Geoffroy, 1785, *Ichneumon*)

*tricolor* (Razoumowsky, 1789, *Ichneumon*) preocc.

*generator* (Olivier, 1792, *Ichneumon*)

*maculifrons* (Stephens, 1835, *Ichneumon*)

*pyrrhopus* (Stephens, 1835, *Ichneumon*)

*extinctus* (Ratzeburg, 1844, *Ichneumon*)

*hartigii* (Ratzeburg, 1844, *Ichneumon*)

*impugnator* (Wesmael, 1845, *Ichneumon*) preocc.

*spiracularis* (Tischbein, 1881, *Ichneumon*)

*baudyi* (Pic, 1902, *Ichneumon*)

***fugitivus*** (Gravenhorst, 1829, *Ichneumon*) E I

*rutilus* (Holmgren, 1864, *Ichneumon*) preocc.

*capreolus* (Berthoumieu, 1899, *Ichneumon*)

***infidus*** (Wesmael, 1848, *Ichneumon*) E I

*liostylus* (Thomson, 1897, *Ichneumon*)

***jocularis*** (Wesmael, 1848, *Ichneumon*)[[352]](#footnote-353) E S I

*punctifrons* (Holmgren, 1864, *Ichneumon*)

*semiannulatus* (Kriechbaumer, 1895, *Ichneumon*) preocc.

Horstmann (2002*c*)

*angusteannulatus* (Strobl, 1901, *Ichneumon*)

***luteiventris*** (Gravenhorst, 1820, *Ichneumon*)

*indictus* (Tischbein, 1874, *Ichneumon*)

***pallitarsis*** (Thomson, 1887, *Ichneumon*) S NMS, det. Riedel, added here[[353]](#footnote-354)

*palliditarsis* (Berthoumieu, 1895, *Ichneumon*)

***rufifrons*** (Gravenhorst, 1829, *Ichneumon*) E S I

*frontatorius* (Fabricius, 1793, *Ichneumon*) *nom. ob.*

Horstmann (2001*b*)

*pallidiatorius* (Gravenhorst, 1829, *Ichneumon*)

***semirufus*** (Gravenhorst, 1820, *Ichneumon*) E I

*nigroscutatus* (Berthoumieu, 1895, *Ichneumon*)

***sicarius*** (Gravenhorst, 1829, *Ichneumon*) E I

*nigratorius* (Panzer, 1800, *Ichneumon*) preocc.

*ingratorius* (Gravenhorst, 1829, *Ichneumon*)

*jugatus* (Gravenhorst, 1829, *Ichneumon*)

*alboannulatus* (Strobl, 1901, *Ichneumon*)

*atrocellaris* (Pic, 1927, *Ichneumon*)

*forticornis* (Hedwig, 1956, *Hoplismenus*)

***versator*** (Thunberg, 1824, *Ichneumon*) E S I

*pallifrons* (Gravenhorst, 1829, *Ichneumon*)

*pallidifrons* (Marshall, 1872, *Ichneumon*)

*anotylus* (Thomson, 1893, *Ichneumon*) Riedel (2014)

***viator*** (Scopoli, 1763, *Ichneumon*) E S W I

*nigritarius* (Gravenhorst, 1820, *Ichneumon*)

*obfuscator* (Thunberg, 1824, *Ichneumon*) preocc.

*aethiops* (Gravenhorst, 1829, *Ichneumon*)

*pinetorum* (Ratzeburg, 1852, *Ichneumon*)

*parviscopa* (Thomson, 1893, *Ichneumon*)

*brischkei* (Berthoumieu, 1895, *Ichneumon*) preocc., unavailable

*nuperus* (Berthoumieu, 1910, *Ichneumon*)

*charadensis* (Pic, 1924, *Ichneumon*)

*atrifemur* (Fahringer, 1943, *Ichneumon*)

*rufipes* Constantineanu, 1954 preocc.

***vulpecula*** (Kriechbaumer, 1875, *Ichneumon*) S

NMS, det. Hilpert, added here

*pseudogracilentus* (Strobl, 1901, *Ichneumon*)

*hemerythrus* Heinrich, 1949

***CRYPTEFFIGIES*** Heinrich, 1961

***albilarvatus*** (Gravenhorst, 1820, *Ichneumon*) E S W I

*obscurior* (Berthoumieu, 1895, *Ichneumon*) preocc., unavailable

*deubeli* (Kiss, 1924, *Megaplectes*)

***lanius*** (Gravenhorst, 1829, *Ichneumon*) E S I

*aberrans* (Taschenberg, 1865, *Phygadeuon*)

*muelleri* (Kiss, 1929, *Plectocryptus*)

***pseudocryptus*** (Wesmael, 1857, *Ichneumon*) E

*punctulatus* (Kriechbaumer, 1891, *Microcryptus*)

***CRYTEA*** Cameron, 1906

***sanguinator*** (Rossi, 1794, *Ichneumon*) E S I

*ruficollis* (Stephens, 1835, *Ichneumon*) preocc.

*discrepator* (Wesmael, 1845, *Ichneumon*)

*sanguinator* (Desvignes, 1856, *Cryptus*) preocc.

*multifarius* (Berthoumieu, 1897, *Ichneumon*)

***CTENICHNEUMON*** Thomson, 1894

*DOCHYTELES* Berthoumieu, 1904

***castigator*** (Fabricius, 1793, *Ichneumon*) E I

?*certator* (Müller, 1776, *Ichneumon*)

?*abrogator* (Schrank, 1781, *Ichneumon*)

?*cardui* (Schrank, 1786, *Ichneumon*)

?*adustus* (Gmelin, 1790, *Ichneumon*)

?*ruficingulus* (Schrank, 1802, *Ichneumon*)

***devylderi*** (Holmgren, 1871, *Amblyteles*) E

*ineptus* (Holmgren, 1871, *Amblyteles*)

*tischbeini* (Berthoumieu, 1896, *Amblyteles*)

Horstmann (2004*c*)

***divisorius*** (Gravenhorst, 1820, *Ichneumon*) E S

*obsoletorius* (Fabricius, 1793, *Ichneumon*) *nom. ob.*

Horstmann (2001*b*)

*baeticus* (Spinola, 1843, *Ichneumon*)

*clipeator* (Habermehl, 1917, *Dochyteles*)

***edictorius*** (Linnaeus, 1758, *Ichneumon*) E I

*gladiatorius* (Müller, 1776, *Ichneumon*)

*fuscipes* (Geoffroy, 1785, *Ichneumon*)

*trichrous* (Gmelin, 1790, *Ichneumon*)

*erectorius* (Fabricius, 1798, *Ichneumon*)

*calceatorius* (Panzer, 1801, *Ichneumon*)

*amputatorius* (Panzer, 1804, *Ichneumon*)

*fossorius* (Gravenhorst, 1820, *Ichneumon*) Horstmann (2000*b*)

*pallipes* (Gravenhorst, 1820, *Ichneumon*)

*depressorius* (Thunberg, 1824, *Ichneumon*)

*incertorius* (Thunberg, 1824, *Ichneumon*)

*perileucus* (Gravenhorst, 1829, *Ichneumon*)

*cognatus* (Stephens, 1833, *Ichneumon*) Horstmann (2000*b*)

*nigricornis* (Spinola, 1843, *Ichneumon*) preocc.

*lotharingicus* (Rudow, 1888, *Amblyteles*)

*nigroscutellatus* (Kriechbaumer, 1894, *Amblyteles*) unavailable

*pallidipes* (Dalla Torre, 1902, *Amblyteles*)

***funereus*** (Geoffroy, 1785, *Ichneumon*) E W

*funerarius* (Olivier, 1792, *Ichneumon*)

***inspector*** (Wesmael, 1845, *Amblyteles*) E

*nigriventris* (Berthoumieu, 1896, *Amblyteles*)

*brunnicans* (Constantineanu, 1956, *Amblyteles*)

***melanocastanus*** (Gravenhorst, 1820, *Ichneumon*) E

*rubroater* (Ratzeburg, 1852, *Ichneumon*)

*erythropygus* (Rudow, 1888, *Amblyteles*) preocc.

***messorius*** (Gravenhorst, 1820, *Ichneumon*) E

*montivagus* (Giraud, 1877, *Amblyteles*)

***nitens*** (Christ, 1791, *Ichneumon*) E

?*glabratorius* (Müller, 1776, *Ichneumon*)

*vespertinus* (Christ, 1791, *Ichneumon*)

*mesocastanus* (Gravenhorst, 1820, *Ichneumon*)

*nigrocastaneus* (Berthoumieu, 1896, *Amblyteles*) unavailable

***panzeri*** (Wesmael, 1845, *Amblyteles*) E I M

*flavocinctus* (Desvignes, 1856, *Ichneumon*)

*vexillarius* (Tischbein, 1874, *Amblyteles*)

*rufescens* Morley, 1903

*denticornis* (Strobl, 1904, *Amblyteles*) Horstmann (1999*a*)

*styriacus* (Strobl, 1904, *Amblyteles*) Horstmann (1999*a*)

*wormatiensis* (Habermehl, 1909, *Amblyteles*)

*nigrifemur* (Ulbricht, 1926, *Amblyteles*) unavailable

*rufifemur* (Ulbricht, 1926, *Amblyteles*) unavailable

***CTENOCHARES*** Förster, 1869

***bicolorus*** (Linnaeus, 1767, *Ichneumon*) E[[354]](#footnote-355) added by Jones (2001)

*instructor* (Fabricius, 1793, *Ichneumon*)

*deustor* (Thunberg, 1824, *Ichneumon*)

*rufator* (Thunberg, 1824, *Ichneumon*)

*apicalis* (Wiedemann, 1824, *Ichneumon*)

*apicalis* (Brullé, 1846, *Joppa*) preocc.

*xanthomelas* (Brullé, 1846, *Ichneumon*) preocc.

***DEUTEROLABOPS*** Heinrich, 1975

***eupitheciae*** (Brischke, 1878, *Ichneumon*) E S

*pulchellatus* (Bridgman, 1889, *Ichneumon*)

***DIPHYUS*** Kriechbaumer, 1890

*PHYSCOTELES* Berthoumieu, 1904

***amatorius*** (Müller, 1776, *Ichneumon*) E S I

*laboratorius* (Fabricius, 1793, *Ichneumon*) preocc.

*nigronotatus* (Pic, 1908, *Amblyteles*)

***castanopyga*** (Stephens, 1835, *Ichneumon*) E S I

*rubriventris* (Wesmael, 1845, *Amblyteles*)

*bicristatus* (Strobl, 1901, *Ichneumon*)

***gradatorius*** (Thunberg, 1824, *Ichneumon*) S

*egregius* (Gravenhorst, 1829, *Ichneumon*)

*sibiricus* (Mocsáry, 1878, *Amblyteles*)

*illustris* (Kriechbaumer, 1894, *Ichneumon*) Horstmann (2006*b*)

*carlsbadensis* (Pic, 1914, *Ichneumon*)

*rufotriangularis* (Pic, 1914, *Ichneumon*)

***longigena*** (Thomson, 1888, *Amblyteles*) E S W

*inermis* (Berthoumieu, 1892, *Amblyteles*)

***luctatorius*** (Linnaeus, 1758, *Ichneumon*) E S I

*erratorius* (Thunberg, 1824, *Ichneumon*)

*litigiosus* (Wesmael, 1854, *Amblyteles*)

*oblongatus* (Tischbein, 1873, *Ichneumon*)

***mercatorius*** (Fabricius, 1793, *Ichneumon*) E[[355]](#footnote-356) I

*nugatorius* (Fabricius, 1794, *Ichneumon*) Horstmann (2001*b*)

*nigricaudus* (Berthoumieu, 1896, *Amblyteles*) preocc., unavailable

***monitorius*** (Panzer, 1801, *Ichneumon*)

*quadrimaculatus* (Schrank, 1802, *Ichneumon*) preocc.

*interruptorius* (Fabricius, 1804, *Ichneumon*)

***ochromelas*** (Gmelin, 1790, *Ichneumon*) E

*pulchellus* (Christ, 1791, *Ichneumon*)

*negatorius* (Fabricius, 1793, *Ichneumon*)

*ornatorius* (Panzer, 1800, *Ichneumon*)

*umbratorius* (Thunberg, 1824, *Ichneumon*)

*sartorius* (Gravenhorst, 1829, *Ichneumon*)

*canaliculatus* (Saussure, 1892, *Ichneumon*) preocc.

*nigripes* (Seyrig, 1928, *Spiloteles*)

*trialbatus* (Constantineanu, 1954, *Amblyteles*)

***palliatorius*** (Gravenhorst, 1829, *Ichneumon*) E S W I

?*defensorius* (Villers, 1789, *Ichneumon*)

*erythropygus* (Gravenhorst, 1829, *Ichneumon*)

*spoliator* (Wesmael, 1845, *Amblyteles*)

*ancipiterus* (Desvignes, 1856, *Ichneumon*)

*dubitatus* (Desvignes, 1856, *Ichneumon*)

*ochraceus* (Tischbein, 1873, *Ichneumon*)

*aequivocus* (Tischbein, 1879, *Ichneumon*)

*infinitus* (Tischbein, 1879, *Ichneumon*)

*gemmatus* (Tischbein, 1881, *Ichneumon*)

*laetus* (Tischbein, 1881, *Ichneumon*) preocc.

*brunneonotatus* (Pic, 1898, *Amblyteles*)

*atratus* (Berthoumieu, 1901, *Amblyteles*)

*rufotriangularis* (Pic, 1915, *Amblyteles*) preocc.

*subniger* (Habermehl, 1929, *Amblyteles*) preocc.

***quadripunctorius*** (Müller, 1776, *Ichneumon*) E W I

*constellatus* (Geoffroy, 1785, *Ichneumon*)

*citreus* (Christ, 1791, *Ichneumon*) Horstmann (2001*b*)

*intratorius* (Fabricius, 1793, *Ichneumon*) Horstmann (2001*b*)

*jubilatorius* (Müller, 1776, *Ichneumon*) Horstmann (2001*b*)

*pedatorius* (Fabricius, 1793, *Ichneumon*) Horstmann (2001*b*)

*natatorius* (Fabricius, 1798, *Ichneumon*)

*mediatorius* (Panzer, 1801, *Ichneumon*)

*bipunctatus* (Schrank, 1802, *Ichneumon*) preocc.

*desertorius* (Panzer, 1806, *Ichneumon*)

*xanthozosmus* (Gravenhorst, 1820, *Ichneumon*)

*natator* (Zetterstedt, 1838, *Ichneumon*)

*infestorius* (Fonscolombe, 1847, *Ichneumon*)

*notatorius* (Marshall, 1872, *Amblyteles*) preocc.

*bipunctatus* (Rudow, 1888, *Amblyteles*) preocc.

*schrammi* (Pic, 1827, *Amblyteles*)

***raptorius*** (Linnaeus, 1758, *Ichneumon*) E

*quadriguttorius* (Thunberg, 1824, *Ichneumon*)

*gravenhorstii* (Wesmael, 1836, *Ichneumon*) preocc.

*flavaginis* (Schiødte, 1839, *Ichneumon*) Horstmann (2004*b*)

*flavolaetus* (Berthoumieu, 1896, *Amblyteles*) preocc., unavailable

*quercus* (Pic, 1917, *Amblyteles*)

***salicatorius*** (Gravenhorst, 1820, *Ichneumon*) E S W

*cinctorius* (Stephens, 1835, *Ichneumon*) preocc.

*indocilis* (Wesmael, 1845, *Amblyteles*) Horstmann (1998*b*)

*relucens* (Desvignes, 1856, *Ichneumon*)

*inaciculatus* (Pic, 1927, *Amblyteles*)

*nigrobinotatus* (Pic, 1927, *Amblyteles*)

***septemguttatus*** (Gravenhorst, 1829, *Ichneumon*) E I

*wesmaeli* (Tischbein, 1868, *Amblyteles*)

*triplicatus* (Thomson, 1894, *Amblyteles*)

***trifasciatus*** (Gravenhorst, 1829, *Ichneumon*) E S I

*triangulator* (Stephens, 1835, *Ichneumon*)

*daguini* (Pic, 1920, *Amblyteles*)

***ERISTICUS*** Wesmael, 1845

***clarigator*** (Wesmael, 1845, *Ichneumon*) E

*pachycephalus* (Rudow, 1886, *Phygadeuon*)

*cephalotes* (Berthoumieu, 1906, *Amblyteles*)

***clericus*** (Gravenhorst, 1829, *Ichneumon*) E

*eucephalus* (Wesmael, 1848, *Ichneumon*)

***EUPALAMUS*** Wesmael, 1845

***lacteator*** (Gravenhorst, 1829, *Ichneumon*) E

*fenestrator* (Zetterstedt, 1838, *Ichneumon*)

*depexus* (Wesmael, 1845, *Ichneumon*)

*albatus* (Tischbein, 1879, *Ichneumon*)

***wesmaeli*** (Thomson, 1886, *Ichneumon*) E

***EUTANYACRA*** Cameron, 1903

***crispatoria*** (Linnaeus, 1758, *Ichneumon*) E I

*limbatoria* (Thunberg, 1824, *Ichneumon*)

*rufatoria* (Gravenhorst, 1829, *Ichneumon*) preocc.

*nemoralis* (Tischbein, 1876, *Ichneumon*) preocc.

*laticincta* (Rudow, 1888, *Amblyteles*)

*bicuspis* (Berthoumieu, 1892, *Amblyteles*)

*pallidior* (Pic, 1898, *Amblyteles*)

***glaucatoria*** (Fabricius, 1793, *Ichneumon*)[[356]](#footnote-357) E I

?*albiventris* (Gmelin, 1790, *Ichneumon*)

*hungarica* (Tischbein, 1868, *Amblyteles*)

*sicula* (Rudow, 1888, *Amblyteles*)

*distyca* (Berthoumieu, 1894, *Amblyteles*)

*hispanica* (Berthoumieu, 1896, *Amblyteles*)

*spoliata* (Berthoumieu, 1896, *Amblyteles*) unavailable

*medinai* (Berthoumieu, 1903, *Amblyteles*)

*nigroscutellatus* (Ulbricht 1909, *Amblyteles*) preocc., unavailable

*praetexta* (Berthoumieu, 1910, *Amblyteles*)

*bruyanti* (Pic, 1927, *Amblyteles*)

*viturati* (Pic, 1927, *Amblyteles*)

*krapinensis* (Schmiedeknecht, 1930, *Amblyteles*)

*bimaculata* (Constantineanu, 1954, *Amblyteles*) preocc.

***pallidicornis*** (Gravenhorst, 1829, *Ichneumon*) E S

*dimidiata* (Stephens, 1835, *Ichneumon*) preocc.

***picta*** (Schrank, 1776, *Ichneumon*) E

*laboratoria* (Müller, 1776, *Ichneumon*)

*sanguinea* (Christ, 1791, *Ichneumon*)

*vadatoria* (Illiger, 1807, *Ichneumon*)

*affirmatoria* (Thunberg, 1824, *Ichneumon*)

*concinnus* (Stephens, 1829, *Ichneumon*) Horstmann (2000*b*)

***EXEPHANES*** Wesmael, 1845[[357]](#footnote-358)

*OCTATOMUS* Tischbein, 1881

***fulvescens*** Vollenhoven, 1875 E I

*ulbrichti* Hinz, 1957 Hinz & Horstmann (2000)

***ischioxanthus*** (Gravenhorst, 1829, *Ichneumon*) E I

*exulans* (Gravenhorst, 1829, *Ichneumon*)

*hilaris* (Gravenhorst, 1829, *Ichneumon*)

*subnudus* Tischbein, 1881

***occupator*** (Gravenhorst, 1829, *Ichneumon*) E W I

*contaminatus* (Gravenhorst, 1829, *Ichneumon*)

*munki* (Kriechbaumer, 1893, *Ichneumon*) Horstmann (2006*b*)

*munki* Kriechbaumer, 1895 preocc Hinz & Horstmann (2000)

*uniguttatus* Kriechbaumer, 1895

*unipunctatus* Strobl, 1901

***riesei*** (Habermehl, 1916, *Ichneumon*)[[358]](#footnote-359) E I

*hoerhammeri* Heinrich, 1949 Hinz & Horstmann (2000)

*amabilis* Kreichbaumer, 1895 preocc.

***venustus*** (Tischbein, 1876, *Ichneumon*) I

*insidiator* (Tischbein, 1876, *Ichneumon*) Hinz & Horstmann (2000)

*caelebs* Kreichbaumer, 1890 Hinz & Horstmann (2000)

***GAREILA*** Heinrich, 1980

***tenebrosa*** (Wesmael, 1845, *Ichneumon*) S NMS, det. Riedel, added here

*nigricornis* (Schmiedeknecht, 1930, *Ichneumon*) preocc.

***HEPIOPELMUS*** Wesmael, 1845

*EPIOPELMUS* Dalla Torre, 1902

***melanogaster*** (Gmelin, 1790, *Ichneumon*) E S W I M

*leucostigmus* (Gravenhorst, 1820, *Ichneumon*)

*maculiventris* (Desvignes, 1856, *Ichneumon*)

*aureosericeus* Taschenberg, 1866

*incorruptus* (Holmgren, 1871, *Amblyteles*)

*palliventris* (Rudow, 1888, *Amblyteles*)

*annulitarsis* (Pic, 1914, *Acolobus*)

*maculipes* Hellén, 1951

***variegatorius*** (Panzer, 1800, *Ichneumon*) E W I

*notatorius* (Panzer, 1801, *Ichneumon*) preocc.

*flavoguttatus* (Gravenhorst, 1829, *Ichneumon*)

***HOMOTHERUS*** Förster, 1869

***locutor*** (Thunberg, 1824, *Ichneumon*) E S W I

*labiatorius* (Thunberg, 1824, *Ichneumon*)

*albicinctus* (Gravenhorst, 1829, *Ichneumon*)

*albiceps* (Hartig, 1838, *Phygadeuon*)

*festinatorius* (Zetterstedt, 1838, *Ichneumon*)

*lautus* (Tischbein, 1868, *Ichneumon*)

*ruber* (Kiss, 1924, *Proscus*)

***magus*** (Wesmael, 1855, *Ichneumon*) E I

*clavipes* (Möller, 1883, *Ichneumon*) preocc.

*nitidus* (Bridgman, 1886, *Phaeogenes*)

***varipes*** (Gravenhorst, 1829, *Ichneumon*) E S W I

*costator* (Donovan, 1810, *Ichneumon*) preocc.

Horstmann (2002*b*)

*decimator* (Gravenhorst, 1829, *Ichneumon*)

*laevis* (Ratzeburg, 1844, *Ichneumon*)

*pictipes* (Holmgren, 1864, *Ichneumon*)

*fallax* (Habermehl, 1923, *Cratichneumon*) invalid

*anglicanus* (Schmiedeknecht, 1928, *Ichneumon*)

***HOPLISMENUS*** Gravenhorst, 1829

*PERITAENIUS* Förster, 1869

*TAENIASPIS* Clément, 1927

***albifrons*** Gravenhorst, 1829[[359]](#footnote-360) E S I

*axillatorius* misident.

?*armatorius* (Fabricius, 1787, *Ichneumon*) preocc.

*albifrons* Gravenhorst, 1829

*perniciosus* Gravenhorst, 1829

*crassicornis* (Rudow, 1883, *Cryptus*) preocc.

*bellicosus* (De Stefani, 1885, *Ichneumon*)

***bidentatus*** (Gmelin, 1790, *Ichneumon*) E W I Whitehead (2003)

*moestus* Gravenhorst, 1829

*maurus* (Marshall, 1873, *Mesostenus*)

*ichneumonoides* (Rudow, 1883, *Cryptus*)

*berthoumieui* Pic, 1897

*spinosus* (Morley, 1903, *Dinotomus*)

*alpinus* (Clément, 1927, *Peritaenius*)

*bavaricus* (Clément, 1927, *Peritaenius*)

***bispinatorius*** (Thunberg, 1824, *Ichneumon*) E S

NMS, det. Riedel, added here

*annulatus* Berthoumieu, 1894

*nigripes* Seyrig, 1927

*rufitarsis* Constantineanu, Andriescu & Ciochia, 1956

***ICHNEUMON*** Linnaeus, 1758[[360]](#footnote-361)

*BRACHYPTERUS* Gravenhorst, 1829 preocc.

*PTEROCORMUS* Förster, 1850

***albiger*** Wesmael, 1845 E S I

*tempestivus* Holmgren, 1864

***alius*** Tischbein, 1879 E S

*eurycerus* Thomson, 1890

*dubiosus* Habermehl, 1926

*petrophilus* Heinrich, 1951

**?*alpestris*** Holmgren, 1864 S[[361]](#footnote-362) added by Hilpert (1992)

***analis*** Gravenhorst, 1829

*nigroscutellatus* Habermehl, 1916 preocc.

***aquilonius*** Perkins, 1953 E S I

***bellipes*** Wesmael, 1845 S

*medialis* Wesmael, 1855

*divergens* Holmgren, 1864

*strangulator* Tischbein, 1876

*evanidus* Berthoumieu, 1892

*orbitalis* Kriechbaumer, 1894 preocc., unavailable

*rasnitsyni* Heinrich, 1978

***bucculentus*** Wesmael, 1845 E W I

*glaucus* Tischbein, 1876

*umbilicatus* Valemberg, 1975

***caloscelis*** Wesmael, 1845 E I

*caloscelus* Marshall, 1872

*decens* (Berthoumieu, 1910, *Amblyteles*)

***cessator*** Müller, 1776 E I

*custodiator* Fabricius, 1793

*compunctor* Stephens, 1835 preocc.

***computatorius*** Müller, 1776 I

*croceipes* Wesmael, 1848

*bicoloripes* Tischbein, 1868

*insolitus* Berthoumieu, 1895 preocc., unavailable

***confusor*** Gravenhorst, 1820 E S W I

*confusorius* Gravenhorst, 1829

*crassicornis* Tischbein, 1873 preocc.

*retectus* Tischbein, 1873

*atronotatus* Pic, 1917

***crassifemur*** Thomson, 1886

*sulphuratus* Kriechbaumer, 1894 preocc.

***deliratorius*** Linnaeus, 1758[[362]](#footnote-363) E S W I

*alternatus* Schrank, 1776

?*fabricatorius* Müller, 1776

*palmarius* Geoffroy, 1785

*inflictorius* Rossi, 1792

*multiannulatus* Gravenhorst, 1829

*delirator* Zetterstedt, 1838

*gmuendensis* Pfeffer, 1913

*schimitscheki* Fahringer, 1943

***didymus*** Gravenhorst, 1829 E S W

*bisignatus* Gravenhorst, 1829

*dissimulator* (Stephens, 1835, *Trogus*)

*crassorius* Desvignes, 1856

***emancipatus*** Wesmael, 1845 E I

*propinquus* (Taschenberg, 1870, *Exephanes*)

*rugosus* Tischbein, 1873 preocc.

*hostificus* Tischbein, 1881

*ramiformis* Tischbein, 1881

*alpinus* Strobl, 1901 preocc.

*vogesus* Habermehl, 1916

*circalpinus* Heinrich, 1949

***exilicornis*** Wesmael, 1857 E

*hircinus* Holmgren, 1864

*rufolineatus* Holmgren, 1864

*caproni* Perkins, 1953

***extensorius*** Linnaeus, 1758 E S W I M

*compressus* Geoffroy, 1785 preocc.

*tripunctatus* Geoffroy, 1785

*auratus* Gmelin, 1790

*lusorius* Gravenhorst, 1807

*vexatorius* Gravenhorst, 1807

*retractus* Tischbein, 1873

*longareolatus* Thomson, 1886

*atropunctum* Pic, 1917

*quercus* Pic, 1917

*cassonensis* Pic, 1919

*luteorufus* Pic, 1919

*polonicus* (Heinrich, 1929, *Euichneumon*)

*clypeonigro* Constantineanu, 1954

*transitorius* Constantineanu, Suciu, Andriescu & Ciochia, 1957

***formosus*** Gravenhorst, 1829[[363]](#footnote-364) E S I

*obsessor* Wesmael, 1845

*obessor* misspelling

*batis* Holmgren, 1880

*brunneosparsus* Strobl, 1901

*schachti* Heinrich, 1980

***fuscatus*** Gmelin, 1790[[364]](#footnote-365) E

***gracilentus*** Wesmael, 1845 E S I

*gratiosus* Wesmael, 1845

*vicinus* Holmgren, 1864 preocc.

*adscendens* Tischbein, 1881

*improbus* Tischbein, 1881

*quadrilineatus* Tischbein, 1881

*wuestneii* Kriechbaumer, 1890

*bioculatus* Kriechbaumer, 1894 preocc., unavailable

*trioculatus* Habermehl, 1903

*helveticus* Habermehl, 1916 preocc.

***gracilicornis*** Gravenhorst, 1829 E I

*iocerus* Gravenhorst, 1829

*quadrinotatus* Stephens, 1835

*propinquus* Taschenberg, 1870 Hinz & Horstmann (2000)

*longisectus* Berthoumieu, 1895

*nigricaudus* Berthoumieu, 1895 preocc., unavailable

*nigroscutellatus* Berthoumieu, 1895 unavailable

*quadrimaculatus* Habermehl, 1916

*daphne* Bauer, 1985

***haemorrhoicus*** Kriechbaumer, 1887 E W I

*albicollis* Wesmael, 1857 preocc.

*nigrifemur* Constantineanu, Andriescu & Ciochia, 1956 preocc.

***ignobilis*** Wesmael, 1855 S

*filatus* (Tischbein, 1879, *Amblyteles*) Riedel (2014)

*debilis* (Kriechbaumer, 1886, *Amblyteles*) Horstmann (2006*b*)

*isenschmidii* (Kriechbaumer, 1887, *Amblyteles*)

*ambifarius* Berthoumieu, 1904

*baueri* Habermehl, 1935

***insidiosus*** Wesmael, 1845 E S I

*argali* Kriechbaumer, 1882

*corfitzi* Thomson, 1890

*jesperi* Thomson, 1893 preocc.

*gansuanus* Kokujev, 1904

*scanicus* Schmiedeknecht, 1929 preocc.

***languidus*** Wesmael, 1845 E added by Hilpert (1992)[[365]](#footnote-366)

*immisericors* Tischbein, 1876

*malignus* Tischbein, 1881

*nigrocastaneus* Tischbein, 1881

*luteoannulatus* Pic, 1915

***lautatorius*** Desvignes, 1856 E

*amabilis* Giraud, 1863

*bizonatus* (Rudow, 1888, *Amblyteles*)

*cingulatus* Berthoumieu, 1895 unavailable

*mutabilis* Berthoumieu, 1895 preocc., unavailable

*gynandra* Habermehl, 1903

*nigropunctatus* Habermehl, 1903

*trimaculatus* Habermehl, 1903 preocc.

***ligatorius*** Thunberg, 1824 E S I

*gradarius* Wesmael, 1848

*refractarius* Wesmael, 1855

*velatus* Wesmael, 1855

*firmipes* Wesmael, 1857

*thulensis* Ruthe, 1859

*faroensis* Schmiedeknecht, 1938

*plautus* Hilpert, 1992 Riedel (2014)

***lugens*** Gravenhorst, 1829[[366]](#footnote-367) E W I

*napaeus* Holmgren, 1880

***megapodius*** Heinrich, 1949[[367]](#footnote-368) E S

*nigroscutellatus* Kriechbaumer, 1897 unavailable

*alpinus* Habermehl, 1913 preocc.

*megapodiops* Bauer, 1985

***melanotis*** Holmgren, 1864 E S I

*macrocerus* Thomson, 1886

*discolor* Berthoumieu, 1895 preocc., unavailable

*macrocerophorus* Dalla Torre, 1901

***memorator*** Wesmael, 1845 E W I

*incomptus* Holmgren, 1864

***minutorius*** Desvignes, 1856 E S

*guttatus* Tischbein, 1873 Horstmann (2003*a*)

*captorius* Thomson, 1887 preocc.

*xanthognathus* Thomson, 1887

*flavipetiolatus* Habermehl, 1903

***molitorius*** Linnaeus, 1761 E S I

*molitor* Zetterstedt, 1838

*holsaticus* Tischebin, 1873

*intrudens* Smith, 1874

*croceiventris* (Rudow, 1888, *Amblyteles*)

*montanus* Habermehl, 1903

*corsicator* Aubert, 1961

***mordax*** Kriechbaumer, 1875 S[[368]](#footnote-369) added by Hilpert (1992)

***oblongus*** Schrank, 1802 E S I

*latrator* misident. Horstmann (2001*b*)

*crassipes* Gmelin, 1790, preocc.

*geniculator* Gravenhorst, 1807

*elegans* Gravenhorst, 1829

*means* (Gravenhorst, 1829, *Brachypterus*)

***primatorius*** Forster, 1771 E S W I M Cowin & Williamson (1940)

*bicinctus* Christ, 1791 preocc.

*grossorius* Fabricius, 1793

*gemellitorius* Thunberg, 1824

*flavolineatus* Gravenhorst, 1829

*monetierensis* Pic, 1914

***rufidorsatus*** Bridgman, 1887 S

***sarcitorius*** Linnaeus, 1758 E S W I M

*vaginatorius* Linnaeus, 1758

*curvatorius* Müller, 1776

*bipartitus* Geoffroy, 1785

*flavatus* Gmelin, 1790 preocc.

*farctor* Gravenhorst, 1807

*zaydamensis* Kokujev, 1909

*funereus* Schmiedeknecht, 1928 preocc.

*niger* Constantineanu, 1954 preocc.

***sculpturatus*** Holmgren, 1864 E

*nereni* Thomson, 1887

*albicaudus* Berthoumieu, 1895 unavailable

*flavocingulatus* Habermehl, 1916

***simulans*** Tischbein, 1873 E S I

*variolosus* Holmgren, 1878

*subquadratus* Thomson, 1887

*obscuratus* Habermehl, 1916

***spurius*** Wesmael, 1848 E I

*sieberti* Habermehl, 1929

***stigmatorius*** Zetterstedt, 1838 S I

*cursorius* Zetterstedt, 1838

*walkeri* Wesmael, 1848

*rubedinis* Desvignes, 1856

*polyonomus* Wesmael, 1859

*kamtschaticus* Roman, 1927

*modestus* Habermehl, 1935 preocc.

***stramentarius*** Gravenhorst, 1820[[369]](#footnote-370) E S W I M

*clitellarius* Holmgren, 1880

*rhaeticus* (Habermehl, 1917, *Dochyteles*)

*scelestus* Perkins, 1952 preocc.

*atrifemur* Perkins, 1953 preocc.

*circumscriptor* Valemberg, 1975

*medianus* Berthoumieu, 1910

***stramentor*** Rasnitsyn, 1981 E W added by Hilpert (1992)

*stramentarius* misident.[[370]](#footnote-371)

***suspiciosus*** Wesmael, 1845 E S I M Cowin & Williamson (1940)

*mellinurus* Wesmael, 1848

*trispilus* Thomson, 1888

*rufonotatus* Pic, 1929

***terminatorius*** Gravenhorst, 1820 E I

*concinnatorius* Stephens, 1835

*fulvoscutellatus* Stephens, 1835

***tuberculipes*** Wesmael, 1848 E I

*cuneatus* Tischbein, 1876

*limbatus* Tischbein, 1879

*piceatus* Tischbein, 1879

*mediorufus* Schmiedeknecht, 1930

***vafer*** Tischbein, 1876 E S

*conjugalis* Holmgren, 1878

*brevicornis* Tischbein, 1881 preocc.

*rogenhoferi* Kriechbaumer, 1888

*quartanus* Perkins, 1953

***validicornis*** Holmgren, 1864 E S I M

*vivacior* Tischbein, 1873

*pseudoconfusor* Heinrich, 1980

***ventus*** Hilpert, 1992 S added by Hilpert (1992)

***vulneratorius*** Zetterstedt, 1838 S I

*dahlbomi* Wesmael, 1857

*versutus* Holmgren, 1864

***xanthorius*** Forster, 1771 E S I

*flaviniger* Gravenhorst, 1820

*nassavicus* (Habermehl, 1917, *Physcoteles*)

*bimactulatus* (Habermehl, 1917, *Physcoteles*)

doubtfully placed species of *Ichneumon*

[***femorator*** Kirby, 1802 preocc., nom. dub.]

***LIMERODES*** Wesmael, 1845

***arctiventris*** (Schiødte, 1839, *Ichneumon*) E S I

*arctiventris* (Boie, 1841, *Ichneumon*) preocc.

*ophioniventris* Wesmael, 1845

***LIMERODOPS*** Heinrich, 1949

*OXYSOMA* Kriechbaumer, 1875 preocc.

***elongatus*** (Brischke, 1865, *Eurylabus*) E S I

*fluvipes* (Matsumura, 1911, *Hoplismenus*)

***subsericans*** (Gravenhorst, 1820, *Ichneumon*) E S I M

*pedestrinus* (Gravenhorst, 1820, *Ichneumon*)

*cognatus* (Stephens, 1835, *Ichneumon*)

***MELANICHNEUMON*** Thomson, 1893

***leucocheilus*** (Wesmael, 1845, *Ichneumon*) E

*arieticornis* (Berthoumieu, 1906, *Ichneumon*)

***OBTUSODONTA*** Heinrich, 1962

***equitatoria*** (Panzer, 1786, *Ichneumon*)

*antennatoria* (Panzer, 1800, *Ichneumon*)

*mediatoria* (Fabricius, 1804, *Ichneumon*) preocc.

*cingulatoria* (Thunberg, 1824, *Ichneumon*) preocc.

*haemorrhoidaria* (Thunberg, 1824, *Ichneumon*)

*rufa* (De Stefani, 1885, *Amblyteles*)

*nigricauda* (Berthoumieu, 1896, *Amblyteles*) unavailable

***PLATYLABOPS*** Heinrich, 1950

***apricus*** (Gravenhorst, 1820, *Ichneumon*) E I

*intersector* (Wesmael, 1854, *Amblyteles*)

*semirufus* (Desvignes, 1856, *Hoplismenus*) preocc.

*delphinas* (Berthoumieu, 1892, *Ichneumon*)

*solitarius* (Habermehl, 1929, *Ichneumon*) Horstmann (2000*b*)

***humilis*** (Wesmael, 1857, *Ichneumon*) E

*rufipes* (Strobl, 1901, *Ichneumon*) preocc.

***lariciatae*** (Kreichbaumer, 1890, *Platylabus*) E S I

*atrithorax* (Berthoumieu, 1910, *Ischnogaster*)

***speciosus*** (Wesmael, 1845, *Amblyteles*) W[[371]](#footnote-372)

*castaneusimilis* Heinrich, 1930, *Aoplus*)

***virginalis*** (Wesmael, 1845, *Ichneumon*) E I

*albicoxatus* (Pfeffer, 1913, *Ichneumon*)

***PROBOLUS*** Wesmael, 1845

***crassulus*** Horstmann, 2000 E I

*concinnus* misident.

*crassicornis* (Stephens, 1835, *Ichneumon*) preocc.

Horstmann (2000*e*)

***culpatorius*** (Linnaeus, 1758, *Ichneumon*) E S W

Horstmann (2000*e*)

*alticola* (Gravenhorst, 1820, *Ichneumon*) Horstmann (2000*e*)

*trucidator* (Gravenhorst, 1829, *Ichneumon*)

*femorator* (Stephens, 1835, *Ichneumon*) preocc.

*fossorius* Wesmael, 1845

***RICTICHNEUMON*** Heinrich, 1961

***pachymerus*** (Hartig, 1838, *Phygadeuon*) E NMS, det. Riedel, added here

*trucidus* (Wesmael, 1845, *Ichneumon*)

*aciculator* (Ratzeburg, 1852, *Ichneumon*)

*steinii* (Ratzeburg, 1852, *Ichneumon*)

*septimus* (Berthoumieu, 1910, *Ichneumon*)

***SPILICHNEUMON*** Thomson, 1894

*SPILOTELES* Berthoumieu, 1904

*PSEUDICHNEUMON* Kokujev, 1909

***ammonius*** (Gravenhorst, 1820, *Ichneumon*) E S

*nonagriae* (Holmgren, 1871, *Amblyteles*)

*stagnicola* (Thomson, 1888, *Amblyteles*)

***celenae*** Perkins, 1953 S W I

***johansoni*** (Holmgren, 1871, *Amblyteles*) E I

*subalnotatus* (Pic, 1914, *Amblyteles*)

***occisorius*** (Fabricius, 1793, *Ichneumon*) E S I M

*sanguinatorius* (Gravenhorst, 1829, *Ichneumon*)

*nigrinus* (Berthoumieu, 1896, *Ambyteles*) preocc., unavailable

*rufinus* (Berthoumieu, 1896, *Ambyteles*) preocc., unavailable

*plicatus* (Morley, 1903, *Ctenichneumon*)

*morvandicus* (Pic, 1925, *Amblyteles*)

***SPILOTHYRATELES*** Heinrich, 1967

***nuptatorius*** (Fabricius, 1793, *Ichneumon*) I

*fabricii* (Schrank, 1802, *Ichneumon*) Horstmann (2001*b*)

*terminator* (Panzer, 1804, *Ichneumon*)

*insidiator* (Fonscolombe, 1847, *Ichneumon*)

*melanocerus* (Wesmael, 1845, *Ichneumon*)

*cubicularis* (Desvignes, 1856, *Ichneumon*)

*truncicola* (Thomson, 1888, *Amblyteles*)

*frustrator* (Berthoumieu, 1892, *Amblyteles*)

*paganus* (Berthoumieu, 1892, *Ichneumon*)

*australis* (Habermehl, 1917, *Anisobas*) Horstmann (1997)

***punctus*** (Gravenhorst, 1829, *Ichneumon*) E

*obscuripes* (Holmgren, 1864, *Ichneumon*)

*erraticus* (Berthoumieu, 1892, *Ichneumon*)

*nigriventris* (Berthoumieu, 1895, *Ichneumon*) unavailable

*lateobscurus* (Pic, 1902, *Amblyteles*)

*pillichi* (Kiss, 1929, *Ichneumon*) Horstmann (2008*c*)

***STENAOPLUS*** Heinrich, 1938

***pictus*** (Gravenhorst, 1829, *Hoplismenus*) E S I

*rufescens* (Stephens, 1835, *Ichneumon*) preocc.

*ratzeburgii* (Hartig, 1838, *Cryptus*)

*exornatus* (Wesmael, 1845, *Ichneumon*)

*obscurior* (Pic, 1898, *Ichneumon*) preocc.

***STENICHNEUMON*** Thomson, 1893

***culpator*** (Schrank, 1802, *Ichneumon*) E S W I M

?*ani* (Geoffroy, 1785, *Ichneumon*)

*ater* (Berthoumieu, 1894, *Ichneumon*) preocc., unavailable

*corsicator* Aubert, 1960

***militarius*** (Thunberg, 1824, *Ichneumon*) E S

*pistorius* (Gravenhorst, 1829, *Ichneumon*)

*pistor* (Zetterstedt, 1838, *Ichneumon*)

*sexannularis* (Berthoumieu, 1894, *Ichneumon*)

***STENOBARICHNEUMON*** Heinrich, 1961

***basalis*** (Perkins, 1960, *Barichneumon*) E S

***basiglyptus*** (Kriechbaumer, 1890, *Ichneumon*) E I

*bifossatus* (Berthoumieu, 1892, *Ichneumon*)

*coxiglyptus* (Heinrich, 1951, *Barichneumon*)

***citator*** (Thunberg, 1824, *Ichneumon*) E

*incubitor* misident.

***SYCAONIA*** Cameron, 1903

***foersteri*** (Wesmael, 1848, *Ichneumon*) E S I

*boreosicaria* (Roman, 1913, *Cratichneumon*)

***THYRATELES*** Perkins, 1953

***camelinus*** (Wesmael, 1845, *Amblyteles*) E S

*certator* (Müller, 1776, *Ichneumon*) *nom. ob.*

Horstmann (2001*b*)

*cardui* (Schrank, 1786, *Ichneumon*) *nom. ob.*

Horstmann (2001*b*)

*adustus* (Gmelin, 1790, *Ichneumon*) *nom. ob.*

Horstmann (2001*b*)

*malignus* (Tischbein, 1868, *Amblyteles*)

*brunnipes* (Tischbein, 1879, *Ichneumon*)

*rufomaculatus* (Kriechbaumer, 1894, *Amblyteles*) unavailable

*alticola* (Habermehl, 1920, *Ichneumon*) preocc.

*oisanensis* (Pic, 1927, *Ichneumon*)

***haereticus*** (Wesmael, 1854, *Amblyteles*)

*urticarum* (Holmgren, 1880, *Ichneumon*)

*binotatus* (Kriechbaumer, 1894, *Amblyteles*) preocc., unavailable

?*cinctor* (Kriechbaumer, 1894, *Amblyteles*) preocc.

*pyraeneus* (Pic, 1914, *Ichneumon*) preocc.

***TRICHOLABUS*** Thomson, 1894

***strigatorius*** (Gravenhorst, 1829, *Ichneumon*) E S I M

*quittardi* (Pic, 1904, *Amblyteles*)

*berthoumieui* Pic, 1927

***TRIPTOGNATHUS*** Berthoumieu, 1904

***atripes*** (Gravenhorst, 1820, *Ichneumon*) E NMS, det. Riedel, added here[[372]](#footnote-373)

*goedarti* (Gravenhorst, 1829, *Ichneumon*)

*interruptus* (Gravenhorst, 1829, *Ichneumon*)

*pratensis* (Gravenhorst, 1829, *Ichneumon*)

*quadricingulatus* (Gravenhorst, 1829, *Ichneumon*)

*uniguttatus* (Gravenhorst, 1829, *Ichneumon*)

*ignotus* (Fonscolombe, 1847, *Ichneumon*)

*praedator* (Fonscolombe, 1847, *Ichneumon*) preocc.

*flavifemur* (Tischbein, 1873, *Ichneumon*)

*interjectus* (Tischbein, 1879, *Amblyteles*)

*subfasciatus* (Tischbein, 1879, *Amblyteles*)

*stephani* (Pic, 1903, *Amblyteles*)

*taiyudongus* (Uchida, 1926, *Spilichneumon*)

***sibilans*** (Gravenhorst, 1829, *Ichneumon*) E

*propinquus* (Perkins, 1953, *Amblyteles*)

***VIRGICHNEUMON*** Heinrich, 1977

***albilineatus*** (Gravenhorst, 1820, *Ichneumon*) E

*albolineatus* misspelling

*leucomelas* (Gmelin, 1790, *Ichneumon*) preocc.

*nigratorius* (Panzer, 1804, *Ichneumon*) preocc.

?*bilineator* (Donovan, 1810, *Ichneumon*)

*bipunctorius* (Stephens, 1835, *Ichneumon*) preocc.

***albosignatus*** (Gravenhorst, 1829, *Ichneumon*) E

*mesostilpnus* (Thomson, 1888, *Ichneumon*)

*punctus* (Berthoumieu, 1895, *Ichneumon*) preocc., unavailable

*nigricollis* (Constantineanu, 1954, *Melanichneumon*)

***callicerus*** (Gravenhorst, 1820, *Ichneumon*) E

*plurialbatus* (Wesmael, 1855, *Ichneumon*)

*eremita* (Kokujev, 1909, *Ichneumon*)

***digrammus*** (Gravenhorst, 1820, *Ichneumon*) E I

*nudicoxa* (Thomson, 1888, *Ichneumon*)

*balearicus* (Kriechbaumer, 1894, *Ichneumon*)

***dumeticola*** (Gravenhorst, 1829, *Ichneumon*) E

***faunus*** (Gravenhorst, 1829, *Ichneumon*) E I

*leucopygus* (Gravenhorst, 1829, *Ichneumon*)

***maculicauda*** (Perkins, 1953, *Barichneumon*) E S I

*perscrutator* (Wesmael, 1845, *Ichneumon*) preocc.

***monostagon*** (Gravenhorst, 1820, *Ichneumon*) E I

*luctuosus* (Gravenhorst, 1820, *Ichneumon*)

*indagator* (Wesmael, 1845, *Ichneumon*) preocc.

*redimitus* (Tischbein, 1874, *Ichneumon*)

*explorator* (Tischbein, 1876, *Ichneumon*)

*minor* (Kriechbaumer, 1894, *Ichneumon*) preocc., unavailable

*hexaleucus* (Kriechbaumer, 1899, *Ichneumon*)

Horstmann (2006*b*)

*annulicornis* (Schmiedeknecht, 1928, *Ichneumon*) preocc.

***tergenus*** (Gravenhorst, 1820, *Ichneumon*) E W

*octoguttatus* (Gravenhorst, 1829, *Ichneumon*)

***VULGICHNEUMON*** Heinrich, 1961

***bimaculatus*** (Schrank, 1776, *Ichneumon*) E I

*bimaculatorius* (Panzer, 1801, *Ichneumon*)

***deceptor*** (Scopoli, 1763, *Ichneumon*) E I

*deceptorius* (Thunberg, 1824, *Ichneumon*)

*deceptorius* (Zetterstedt, 1838, *Ichneumon*) preocc.

*vestigator* (Wesmael, 1845, *Ichneumon*) preocc.

*completus* (Berthoumieu, 1894, *Ichneumon*)

*obscurior* (Berthoumieu, 1895, *Ichneumon*) unavailable

***saturatorius*** (Linnaeus, 1758, *Ichneumon*) E S W I M

*nigratorius* (Pontoppidan, 1763, *Ichneumon*)

*carnifex* (Müller, 1776, *Ichneumon*)

*clavatorius* (Müller, 1776, *Ichneumon*)

*fuscocastaneus* (Gravenhorst, 1829, *Ichneumon*)

*saturator* (Zetterstedt, 1838, *Ichneumon*) preocc.

*albotrochanteratus* (Ulbricht, 1926, *Ichneumon*) unavailable

***suavis*** (Gravenhorst, 1820, *Ichneumon*) E S W I

*fallax* (Gravenhorst, 1829, *Ichneumon*)

*lepidus* (Gravenhorst, 1829, *Ichneumon*)

Tribe LISTRODROMINI Förster, 1869

***ANISOBAS*** Wesmael, 1845

*LYCAENIPHILOS* Heinrich, 1934

***cingulatellus*** Horstmann, 1997[[373]](#footnote-374) E W

*cingulatorius* (Gravenhorst, 1820, *Ichneumon*) preocc.

***platystylus*** Thomson, 1888 E

***LISTRODROMUS*** Wesmael, 1845

***nycthemerus*** (Gravenhorst, 1820, *Ichneumon*) E I

*quinqueguttatus* (Gravenhorst, 1829, *Ichneumon*)

***NEOTYPUS*** Förster, 1869

***nobilitator*** (Gravenhorst, 1807, *Ichneumon*) E

*erythronotus* (Rudow, 1882, *Cryptus*)

Tribe OEDICEPHALINI Heinrich, 1934

NOTOSEMINI Townes, 1961

***NOTOSEMUS*** Förster, 1869

*ISCHNIDIUM* Kriechbaumer, 1890

*ISCHNOGASTER* Kriechbaumer, 1890 preocc.

***bohemani*** (Wesmael, 1855, *Phaeogenes*) E

*dives* Brischke, 1887

*albibucca* (Kriechbaumer, 1890, *Ischnogaster*)

*gaullei* (Berthoumieu, 1900, *Ischnus*)

*atriventris* (Pic, 1915, *Ischnogaster*)

Tribe PHAEOGENINI Förster, 1869[[374]](#footnote-375)

***AETHECERUS*** Wesmael, 1845

***discolor*** Wesmael, 1845 E S I

*styriacus* Strobl, 1901

***dispar*** Wesmael, 1845 E S I

*frontatus* Wesmael, 1845

*albipictus* Berthoumieu, 1897 unavailable

*rufipes* Strobl, 1901

***foveolatus*** Gregor, 1940 E S added by Diller & Shaw (2014)

*exilis* (Berthoumieu, 1899, *Diadromus*) preocc.

***horstmanni*** Diller & Shaw, 2014 E S added by Diller & Shaw (2014)

***longulus*** Wesmael, 1845 E

*formosus* (Bridgman, 1881, *Phaeogenes*)

***nitidus*** Wesmael, 1845 E S

*corcyriensis* (Berthoumieu, 1901, *Phaeogenes*)

***placidus*** Wesmael, 1845 E S I

*nigricoxatus* Strobl, 1901

***porcellus*** Holmgren, 1890 E S W added by Diller & Shaw (2014)

***ruberpedatus*** Diller & Shaw, 2014E S added by Diller & Shaw (2014)

***rugifrons*** Holmgren, 1890 E S added by Diller & Shaw (2014)

***subuliferus*** (Holmgren, 1890, *Phaeogenes*)[[375]](#footnote-376) E

added by Diller & Shaw (2014)

***Baeosemus*** Förster, 1869

***mitigosus*** (Gravenhorst, 1829, *Ichneumon*) E S

added by Diller & Shaw (2014)

*phaeocerus* (Wesmael, 1845, *Herpestomus*)

*vulpecula* Holmgren, 1890

***CENTETERUS*** Wesmael, 1845

***confector*** (Gravenhorst, 1829, *Ichneumon*) E I

*picticollis* Wesmael, 1845

*nigridentis* Constantineanu, 1951

***rubiginosus*** (Gmelin, 1790, *Ichneumon*) E S W I M

*opprimator* (Gravenhorst, 1820, *Ichneumon*)

*rufipes* (Brischke, 1891, *Phaeogenes*)

***COLPOGNATHUS*** Wesmael, 1845

***celerator*** (Gravenhorst, 1807, *Ichneumon*) E S W I M

Shaw & Bennett (2001)

*procerus* (Gravenhorst, 1829, *Phygadeuon*)

*femorator* (Stephens, 1835, *Ichneumon*) preocc.

*celeratorius* (Zetterstedt, 1838, *Ichneumon*)

*armatus* Thomson, 1891 Diller & Schönitzer (2003)

*atricornis* Pic, 1914 Diller & Schönitzer (2003)

*femoralis* Habermehl, 1917

*petiolaris* Constantineanu, 1954

*nigroscaposus* Aubert, 1959

***divisus*** Thomson, 1891 E S W I

*atricornis* Pic, 1914

*rufifemur* Constantineanu, 1959

***DIADROMUS*** Wesmael, 1845

*THYRAEELLA* Holmgren, 1890

***albinotatus*** (Gravenhorst, 1829, *Ichneumon*) E

***arrisor*** Wesmael, 1845 E NHM, Ely coll., det. Diller, added here

***candidatus*** (Gravenhorst, 1829, *Ichneumon*)

*guttulatus* (Gravenhorst, 1829, *Ichneumon*)

*aries* (Brischke, 1887, *Phaegoenes*)

*decolor* Holmgren, 1890

***collaris*** (Gravenhorst, 1829, *Ischnus*) E S

*similis* (Bridgman, 1881, *Phaeogenes*)

*bellulus* (Kriechbaumer, 1894, *Phaeogenes*)

*brischkei* Berthoumieu, 1897 unavailable

*punicus* Berthoumieu, 1898

*rufiscapus* Pic, 1902

*cabrerai* Berthoumieu, 1903

*hispanicus* (Berthoumieu,1904, *Heterischnus*)

*brevicauda* (Hellén, 1949, *Ischnopsidea*)

***heteroneurus*** Holmgren, 1890 E S

*quadriguttatus* misident.

*prosopius* Holmgren, 1890

*nigroscutellatus* Constantineanu, Suciu, Andriescu & Ciochia, 1957

***nitidigaster*** Diller & Shaw, 2014 E added by Diller & Shaw (2014)

***pulchellus*** Wesmael, 1845 E added by Diller & Shaw (2014)

***subtilicornis*** (Gravenhorst, 1829, *Ichneumon*) E S

*imbellis* Wesmael, 1845

*dolosus* Berthoumieu, 1899

*nigrinus* (Berthoumieu, 1901, *Phaeogenes*)

***tenax*** Wesmael, 1845 E

***troglodytes*** (Gravenhorst, 1829, *Ichneumon*) E S W I

*abdominator* (Stephens, 1835, *Ichneumon*)

*scobinatus* Holmgren, 1890

***varicolor*** Wesmael, 1845 E S W M

***DICAELOTUS*** Wesmael, 1845

*DELOGLYPTUS* Förster, 1869

*HOLOCREPIS* Förster, 1869

*LEPTODEMAS* Förster, 1869

*CINXAELOTUS* Holmgren, 1890

*EURYPTILUS* Holmgren, 1890

***cameroni*** Bridgman, 1881 E S

*minutulus* Kokujev, 1909

*nigroclypeatus* Constantineanu, 1959

***erythrostoma*** Wesmael, 1845 E I

***inflexus*** Thomson, 1891 E S W I

***orbitalis*** Thomson, 1891 E I

***parvulus*** (Gravenhorst, 1829, *Ichneumon*) E

*alpigenus* Strobl, 1901

***pictus*** (Schmiedeknecht, 1903, *Deloglyptus*) E

*lugens* Berthoumieu, 1906

***pudibundus*** (Wesmael, 1845, *Herpestomus*) E

*alboscutatus* Berthoumieu, 1900

*gaullei* Berthoumieu, 1903 preocc.

***pumilus*** (Gravenhorst, 1829, *Ichneumon*) E S W I

*morosus* Wesmael, 1855

*analis* Berthoumieu, 1901 unavailable

***punctiventris*** (Thomson, 1891, *Deloglyptus*) E S I

*punicus* Berthoumieu, 1901 unavailable

***pusillator*** (Gravenhorst, 1807, *Ichneumon*) S added by Diller & Shaw (2014)

?*pallidus* (Gmelin, 1790, *Ichneumon*)

*notator* (Gravenhorst, 1807, *Ichneumon*)

***pusillus*** Holmgren, 1890 E added by Diller & Shaw (2014)

***resplendens*** Holmgren, 1890 E I

*fitchi* Perkins, 1953

***ruficoxatus*** (Gravenhorst, 1829, *Ichneumon*) E S I

*unipunctatus* Wesmael, 1845

*nigrescens* Constantineanu, 1959

***rufoniger*** Berthoumieu, 1897 E S W I

***schmiedeknechti*** Diller & Shaw, 2014 E added by Diller & Shaw (2014)

*ruficornis* (Schmiedeknecht, 1903, *Eparces*) preocc.

***suspectus*** Perkins, 1953 E

***Dilleritomus*** Aubert, 1979

***apertor*** Aubert, 1979 E added by Diller & Shaw (2014)

***filiformis*** (Strobl, 1901, *Herpestomus*) E W added by Diller & Shaw (2014)

***DIROPHANES*** Förster, 1869

***callopus*** (Wesmael, 1845, *Phaeogenes*) E S

*tibiator* (Thunberg, 1824, *Ichneumon*) preocc.

*palliditarsis* (Berthoumieu, 1900, *Diadromus*)

***foveolatus*** (Perkins, 1953, *Phaeogenes*)[[376]](#footnote-377) E S I

***fulvitarsis*** (Wesmael, 1845, *Phaeogenes*) E S I M

*limatus* (Wesmael, 1845, *Phaeogenes*)

*hyperboreus* (Holmgren, 1890, *Phaeogenes*)

*nitidiventris* (Holmgren, 1890, *Phaeogenes*)

*ruficoxa* (Thomson, 1891, *Phaeogenes*)

***invisor*** (Thunberg, 1824, *Ichneumon*) E S W

*stimulator* (Gravenhorst, 1829, *Ichneumon*)

*homochlorus* (Wesmael, 1845, *Phaeogenes*)

*kabylianus* (Pic, 1897, *Phaeogenes*)

***maculicornis*** (Stephens, 1835, *Ichneumon*) E S

*scutellaris* (Wesmael, 1845, *Phaeogenes*)

*bisignatus* (Holmgren, 1890, *Phaeogenes*)

*dentatus* (Pic, 1923, *Phaeogenes*)

*nigroscutellatus* (Habermehl, 1929, *Phaeogenes*)

*murinanae* (Fahringer, 1936, *Microcryptus*)

*gigas* (Fahringer, 1943, *Phaeogenes*)

*dinianae* (Fahringer, 1948, *Phaeogenes*)

*ruficoxis* (Constantineanu, 1959, *Phaeogenes*) preocc.

***mysticus*** (Wesmael, 1855, *Phaeogenes*)[[377]](#footnote-378) E S

*tetricus* Wesmael, 1855

***regenerator*** (Fabricius, 1804, *Cryptus*) E S W I M

*rusticatus* (Wesmael, 1845, *Phaeogenes*) Horstmann (2001*b*)

***EPARCES*** Förster, 1869

***grandiceps*** (Thomson, 1891, *Centeterus*) E

***EPITOMUS*** Förster, 1869

***infuscatus*** (Gravenhorst, 1829, *Hemiteles*) E S W I M

*pygmaeus* (Brischke, 1890, *Hemiteles*) preocc.

*parvus* Thomson, 1891

*laeviareolatus* Schmiedeknecht, 1904

***proximus*** Perkins, 1953 E S W I

***ERIPLATYS*** Förster, 1869

*ANOPIESTA* Förster, 1869

*MELANOMICRUS* Morley, 1903

***ardeicollis*** (Wesmael, 1845, *Herpestomus*) E S I

*elliotti* (Morley, 1903, *Melanomicrus*)

*neirae* (Ceballos, 1958, *Herpestomus*)

***sawoniewiczi*** Diller, 1993 E S added by Diller & Shaw (2014)

***HEMICHNEUMON*** Wesmael, 1857

***subdolus*** Wesmael, 1857 E I

*elongatus* (Ratzeburg, 1852, *Hemiteles*) preocc.

*suspectus* Wesmael, 1857

*varians* (Taschenberg, 1865, *Cryptus*) Schwarz (2005)

*tineidarum* (Giraud, 1872, *Ischnus*)

***HERPESTOMUS*** Wesmael, 1845

***arridens*** (Gravenhorst, 1829, *Ichneumon*) E S W

*facialis* (Gravenhorst, 1829, *Ichneumon*)

*xanthops* (Gravenhorst, 1829, *Ichneumon*)

*erubescens* (Berthoumieu, 1899, *Diadromus*)

*subatriceps* (Pic, 1914, *Phaeogenes*)

*transsylvanicus* Kiss, 1924

*rufifrons* (Aerts, 1957, *Rhexidermus*)

*meridionator* Aubert, 1960 Diller & Tereshkin (2005)

***brunnicornis*** (Gravenhorst, 1829, *Ichneumon*) E S I

*padella* (Goureau, 1847, *Ichneumon*)

*bruneicornis* Dalla Torre, 1902

*bisignatus* Habermehl, 1917

*nigriventris* Constantineanu, 1944

***minimus*** (Berthoumieu, 1901, *Phaeogenes*) E S

added by Diller & Shaw (2014)[[378]](#footnote-379)

***nasutus*** Wesmael, 1845 E S I

*furunculus* Wesmael, 1845

*intermedius* Wesmael, 1845

***wesmaeli*** Perkins, 1953 E S I

***HETERISCHNUS*** Wesmael, 1859

*RHEXIDERMUS* Förster, 1869

*ISCHNOPSIDEA* Viereck, 1914

***nigricollis*** (Wesmael, 1845, *Ischnus*) E S W

*rufipes* (Wesmael, 1848, *Ischnus*)

***pulex*** (Müller, 1776, *Ichneumon*)

*murex* (Müller, 1776, *Ichneumon*)

*brevicornis* (Gravenhorst, 1829, *Ichneumon*)

***truncator*** (Fabricius, 1798, *Ichneumon*) E S

?*colorator* (Villers, 1789, *Ichneumon*)

*filiformis* (Gravenhorst, 1829, *Ischnus*)

*thoracicus* (Gravenhorst, 1829, *Ischnus*) Horstmann (2001*b*)

*elegans* (Tischbein, 1868, *Ischnus*)

*montanus* (Berthoumieu, 1897, *Ischnus*)

*moravicus* (Gregor, 1939, *Ischnus*) Horstmann (2002*b*)

*nigrinus* (Constantineanu, 1959, *Ischnus*) preocc.

***MEVESIA*** Holmgren, 1890

***arguta*** (Wesmael, 1845, *Phaeogenes*) E S W I

*tenuis* (Berthoumieu, 1899, *Phaeogenes*)

*albifemur* Constantineanu, 1959

***guttata*** Perkins, 1953 E

***MISETUS*** Wesmael, 1845

***oculatus*** Wesmael, 1845 E S I

*obscurus* Berthoumieu, 1897 unavailable

***NEMATOMICRUS*** Wesmael, 1845

***tenellus*** Wesmael, 1845 E S I

***OIORHINUS*** Wesmael, 1845

***pallipalpis*** Wesmael, 1845 E S W I Shaw (1984)

*striatus* (Bridgman, 1881, *Herpestomus*)

*pallidipalpis* Dalla Torre, 1902

***ORONOTUS*** Wesmael, 1845

*ORONTUS* misspelling

***binotatus*** (Gravenhorst, 1829, *Phygadeuon*) I

*coarctatus* Wesmael, 1845

***OROTYLUS*** Holmgren, 1890

***mitis*** (Wesmael, 1848, *Diadromus*) E[[379]](#footnote-380)

***PARAETHECERUS*** Perkins, 1953

***elongatus*** Perkins, 1953 E

***PHAEOGENES*** Wesmael, 1845[[380]](#footnote-381)

***curator*** (Thunberg, 1824, *Ichneumon*) E W Ely (2002)

*crassidens* Thomson, 1891

***distinctus*** (Bridgman, 1887, *Herpestomus*) E

***heterogonus*** Holmgren, 1890 W I

***melanogonos*** (Gmelin, 1790, *Ichneumon*) E S

*protervus* Wesmael, 1855

*nigripes* Constantineanu, 1954

***nigridens*** (Wesmael, 1845, *Phaeogenes*)[[381]](#footnote-382) E

added by Ely (2002)

*major* (Berthoumieu, 1901, *Phaeogenes*)

***planifrons*** Wesmael, 1845 I

*compar* Berthoumieu, 1904

*hungaricus* (Kiss, 1926, *Orotylus*) Horstmann (2008*c*)

***semivulpinus*** (Gravenhorst, 1829, *Ichneumon*) E S

*mutabilis* (Gravenhorst, 1829, *Ichneumon*)

*rufator* (Stephens, 1835, *Ichneumon*) preocc.

*primarius* Wesmael, 1845

***trepidus*** Wesmael, 1845 E

doubtfully placed species of *Phaeogenes*

[***picipes*** (Stephens, 1835, *Ichneumon*) nom. dub. E]

***STENODONTUS*** Berthoumieu, 1897

*GNATHOXYS* Wesmael, 1845 preocc.

***marginellus*** (Gravenhorst, 1829, *Ichneumon*) E S W I

*albicoxis* Habermehl, 1917

***TRACHYARUS*** Thomson, 1891[[382]](#footnote-383)

***corvinus*** Thomson, 1891 E

*atratus* (Berthoumieu, 1901, *Phaeogenes*)

***TYCHERUS*** Förster, 1869

*MICROPE* Förster, 1869

*PROSCUS* Holmgren, 1890

*MICROPA* Schulz, 1906

*GLYPTICHNEUMON* Habermehl, 1917

***amaenus*** (Wesmael, 1845, *Phaeogenes*)[[383]](#footnote-384) W

added by Diller & Shaw (2014)

*flavoclypeatus* (Strobl, 1901, *Phaeogenes*)

***bellicornis*** (Wesmael, 1845, *Phaeogenes*) E S W I

*rugulosus* (Constantineanu, 1959, *Phaeogenes*)

***brunneus*** (Kiss, 1924, *Eriplatys*) E added by Diller & Shaw (2014)

***capitosus*** (Holmgren, 1890, *Phaeogenes*) S added by Diller & Shaw (2014)

***cephalotes*** (Wesmael, 1845, *Phaeogenes*) E

***coriaceus*** (Perkins, 1953, *Phaeogenes*) E S

***dodecellae*** Ranin, 1983 S added by Diller & Shaw (2014)

***elongatus*** (Thomson, 1891, *Phaeogenes*) E

***eques*** (Wesmael, 1845, *Phaeogenes*) E S

***flavidens*** (Wesmael, 1845, *Phaeogenes*) E

*flavoclypeatus* (Strobl, 1901, *Herpestomus*)

***fuscibucca*** (Berthoumieu, 1901, *Ischnogaster*) S

added by Diller & Shaw (2014)

*kratochvili* (Gregor, 1943, *Eriplatys*)

***fuscicornis*** (Wesmael, 1845, *Phaeogenes*) E S I

*phaeogenoides* (Habermehl, 1917, *Glyptichneumon*)

***helleni*** Ranin, 1983 S added by Diller & Shaw (2014)

***histrio*** (Wesmael, 1848, *Phaeogenes*)[[384]](#footnote-385)

***impiger*** (Wesmael, 1845, *Phaeogenes*)[[385]](#footnote-386) E S

*ruficoxis* (Constantineanu, 1951, *Phaeogenes*)

***improcerus*** Ranin, 1983 E added by Diller & Shaw (2014)

***infimus*** (Wesmael, 1845, *Phaeogenes*) E S I

*minutus* (Wesmael, 1845, *Phaeogenes*)

***ischiomelinus*** (Gravenhorst, 1829, *Ichneumon*) E S I

*eximius* (Wesmael, 1845, *Phaeogenes*)

***jucundus*** (Wesmael, 1845, *Phaeogenes*)[[386]](#footnote-387) E

NHM, added here

***macilentus*** (Wesmael, 1845, *Phaeogenes*) I

***modestus*** (Wesmael, 1845, *Phaeogenes*) E S

*grammostoma* (Kriechbaumer, 1887, *Phaeogenes*)

***ophtalmicus*** (Wesmael, 1845, *Phaeogenes*) E S W I

*ophthalmicus* misspelling

*hybridus* (Wesmael, 1845, *Phaeogenes*)

*pulchricornis* (Brischke, 1891, *Phaeogenes*)

*palliventris* (Berthoumieu, 1910, *Phaeogenes*)

***osculator*** (Thunberg, 1824, *Ichneumon*) E S W I

*nanus* (Wesmael, 1845, *Phaeogenes*)

*lascivus* (Wesmael, 1855, *Phaeogenes*)

*socius* (Holmgren, 1890, *Phaeogenes*)

*inanis* (Berthoumieu, 1901, *Phaeogenes*)

*tristis* (Berthoumieu, 1904, *Phaeogenes*)

*strandi* (Berthoumieu, 1910, *Dicaelotus*)

*nigroclypeatus* (Constantineanu, 1942, *Phaeogenes*)

***planipectus*** (Holmgren, 1890, *Phaeogenes*) S

added by Diller & Shaw (2014)

***socialis*** (Ratzeburg, 1852, *Ichneumon*) E added by Diller & Shaw (2014)

*discoidalis* (Ratzeburg, 1852, *Ichneumon*)

*clypearis* (Brischke, 1878, *Phaeogenes*)

*martialis* (Pic, 1899, *Phaeogenes*)

***stipator*** (Wesmael, 1855, *Phaeogenes*) E S W I

*cambriensis* (Desvignes, 1867, *Ichneumon*)

*cicutellus* (Brischke, 1878, *Phaeogenes*)

*basirufus* (Constantineanu, 1951, *Phaeogenes*)

*fuscitarsis* (Constantineanu, 1951, *Phaeogenes*)

***suspicax*** (Wesmael, 1845, *Phaeogenes*) E S W I

*crassiceps* (Habermehl, 1917, *Proscus*)

***teres*** (Berthoumieu, 1906, *Phaeogenes*) E added by Diller & Shaw (2014)

***vagus*** (Berthoumieu, 1899, *Phaeogenes*) E added by Diller & Shaw (2014)

***verecundus*** Ranin, 1983 E added by Ranin (1983)

Tribe PLATYLABINI Berthoumieu, 1904

PRISTICEROTINI Townes, 1961

***APAELETICUS*** Wesmael, 1845

***bellicosus*** Wesmael, 1845 E

*inclytus* Wesmael, 1853

*cautus* (Berthoumieu, 1898, *Diadromus*)

*rufipes* Constantineanu, 1951

***inimicus*** (Gravenhorst, 1820, *Ichneumon*) E

*haematodus* (Gravenhorst, 1829, *Cryptus*)

Sawoniewicz & Wanat (2003)

*flammeolus* Wesmael, 1845

*balearicus* Kriechbaumer, 1894

*amoenus* Habermehl, 1917

*muelleri* (Kiss, 1929, *Microcryptus*)

***ASTHENOLABUS*** Heinrich, 1951

*STENOLABUS* Heinrich, 1936 preocc.

***latiscapus*** (Thomson, 1894, *Platylabus*) E

***vitratorius*** (Gravenhorst, 1829, *Mesoleptus*) E W

*albinus* (Gravenhorst, 1829, *Hoplismenus*)

*errabundus* (Gravenhorst, 1829, *Hoplismenus*)

*tinctorius* (Gravenhorst, 1829, *Cryptus*)

*coxalis* (Habermehl, 1917, *Platylabus*)

***CYCLOLABUS*** Heinrich, 1936

***axillatorius*** (Thunberg, 1824, *Ichneumon*) E S I

*pactor* (Wesmael, 1845, *Platylabus*) Riedel (2014)

*pici* (Berthoumieu, 1910, *Anisobas*)

*septentrionalis* (Berthoumieu, 1910, *Dicaelotus*)

***dubiosus*** Perkins, 1953 E

***nigricollis*** (Wesmael, 1845, *Platylabus*) E W

***DENTILABUS*** Heinrich, 1974

***variegatus*** (Wesmael, 1845, *Platylabus*) E S

***ECTOPIUS*** Wesmael, 1859

***rubellus*** (Gmelin, 1790, *Ichneumon*) I

*thedenii* (Holmgren, 1871, *Platylabus*)

***HYPOMECUS*** Wesmael, 1845

***quadriannulatus*** (Gravenhorst, 1829, *Mesoleptus*) E S

*albitarsis* Wesmael, 1845

*submarginatus* (Magretti, 1896, *Platylabus*)

Riedel (2008)

*carens* Berthoumieu, 1897 unavailable

***LINYCUS*** Cameron, 1903

***exhortator*** (Fabricius, 1787, *Ichneumon*) E S W I

*dimidiatus* (Gravenhorst, 1829, *Hoplismenus*)

*discedens* (Gravenhorst, 1829, *Phygadeuon*)

*latior* (Pic, 1902, *Platylabus*)

*tricolor* (Berthoumieu, 1904, *Platylabus*)

*balearicus* (Hedwig, 1939, *Platylabus*) Riedel (2008)

***flavitarsis*** (Heinrich, 1937, *Ectopius*) S NMS, det. Riedel, added here

*priesneri* Heinrich, 1972

***PLATYLABUS*** Wesmael, 1845

***concinnus*** Thomson, 1888 E

***curtorius*** (Thunberg, 1824, *Ichneumon*) E S W

*eurygaster* Holmgren, 1871 Riedel (2008)

*punctifrons* Thomson, 1888 Riedel (2007)

***daemon*** Wesmael, 1845[[387]](#footnote-388) E W added by Riedel (2008)

***dolorosus*** (Gravenhorst, 1829, *Ichneumon*) E S

*sollicitus* Wesmael, 1845

*viturati* (Pic, 1902, *Ichneumon*)

***fugator*** (Gravenhorst, 1807, *Ichneumon*) S added by Riedel (2008)

*atricornis* Pic, 1926 Riedel (2007)

***gigas*** Kriechbaumer, 1886 E

***heteromallus*** (Berthoumieu, 1910, *Amblyteles*)[[388]](#footnote-389) E S W I M

*pedatorius* misident. Horstmann (2001*b*)

*rhenana* Habermehl, 1917

***histrio*** Wesmael, 1855 E S

*varipedulis* Wesmael, 1857

*erberi* Tischbein, 1868

***intermedius*** Holmgren, 1871 E S

*polonicus* Heinrich, 1937

***iridipennis*** (Gravenhorst, 1829, *Ichneumon*) E S

*helensis* (Brischke, 1888, *Ichneumon*)

*fornicatus* Kriechbaumer, 1890 Horstmann (2006*b*)

*calidus* Berthoumieu, 1904 Riedel (2008)

*novellus* Berthoumieu, 1910

***judaicus*** Berthoumieu, 1900 E

*stolidus* Perkins, 1953 Riedel (2007)

***neglectus*** (Fonscolombe, 1847, *Ichneumon*) E

*decipiens* Wesmael, 1848 Riedel (2007)

*minai* (De Stefani, 1885, *Ischnus*) Riedel (2008)

***nigrocyaneus*** (Gravenhorst, 1829, *Ichneumon*)

*armatus* Wesmael, 1845

***obator*** (Desvignes, 1856, *Ichneumon*) E S

***odiosus*** Perkins, 1953 E S

***opaculus*** Thomson, 1888 E I

***orbitalis*** (Gravenhorst, 1829, *Ichneumon*) E S W

*vibratorius* misident. Riedel (2008)

*persecutor* (Gravenhorst, 1829, *Ichneumon*)

*subalbellus* (Gravenhorst, 1829, *Ichneumon*)

*volubilis* (Gravenhorst, 1829, *Ichneumon*) Riedel (2008)

*suborbitalis* Kriechbaumer, 1894 Horstmann (2006*b*)

*muticus* Thomson, 1894 Riedel (2007)

***perexiguus*** Heinrich, 1973 E NMS, det. Riedel, added here

***pseudopumilio*** Riedel, 2008 E S W added by Riedel (2008)

***pumilio*** Holmgren, 1871 E

***rufus*** Wesmael, 1845 E S W I

*pictus* Vollenhoven, 1878

*rubeus* Valemberg, 1976 Valemberg (2001)

***sternoleucus*** Wesmael, 1853[[389]](#footnote-390) E S W added by Riedel (2008)

***tenuicornis*** (Gravenhorst, 1829, *Ichneumon*) E

*niger* Wesmael, 1845

***transversus*** Bridgman, 1889 E

*lativentris* Thomson, 1894

***tricingulatus*** (Gravenhorst, 1820, *Ichneumon*) E

*maurus* Berthoumieu, 1900

*berthoumieui* Pic, 1923

*zagoriensis* Heinrich, 1930 Riedel (2008)

***vibratorius*** (Thunberg, 1824, *Ichneumon*) E

*wienkeri* (Ratzeburg, 1844, *Ichneumon*) Riedel (2008)

*rufiventris* Wesmael, 1845 Horstmann (2000*b*)

***PLATYMISCHOS*** Tischbein, 1868

*RYSSOLABUS* Berthoumieu, 1894

***atriventris*** (Pic, 1914, *Ryssolabus*) S

*arcticus* (Hellén, 1942, *Ryssolabus*)

*montanus* (Heinrich, 1951, *Ryssolabus*)

***POECILOSTICTUS*** Ratzeburg, 1852

*IDIOSTOLIS* Förster, 1869

*NEOPLATYLABUS* Heinrich, 1936

***cothurnatus*** (Gravenhorst, 1829, *Hoplismenus*) E W

*orbitatus* (Gravenhorst, 1829, *Hoplismenus*)

*octopunctatus* Ratzeburg, 1852

*ratzeburgi* Kawall, 1868 Horstmann (1997)

*apicalis* (Brischke, 1892, *Hepiopelmus*)

*geometrae* (Berthoumieu, 1894, *Platylabus*)

*saxonicus* (Hedwig, 1939, *Platylabus*)

***PRISTICEROPS*** Heinrich, 1961

***infractorius*** (Linnaeus, 1761, *Ichneumon*) E S W I M

*phaleratus* (Haliday, 1839, *Ichneumon*)

*leucogrammus* (Wesmael, 1853, *Platylabus*)

***PRISTICEROS*** Gravenhorst, 1829

***serrarius*** Gravenhorst, 1829 E

Tribe ZIMMERIINI Heinrich, 1934

***COTIHERESIARCHES*** Telenga, 1929

*ZIMMERIA* Heinrich, 1934

***dirus*** (Wesmael, 1853, *Eurylabus*)[[390]](#footnote-391)

*niger* Telenga, 1929

Subfamily LYCORININAE Cushman & Rohwer, 1920

LYCORINAE misspelling

***LYCORINA*** Holmgren, 1859

*AMYS* Schiødte, 1839 *nom. ob.* Horstmann (2004*b*)

*Toxophoroides* Cresson, 1873

*Chlorolycorina* Cushman, 1920

*Gonioglyphus* Seyrig, 1932

***triangulifera*** Holmgren, 1859 E S I Shaw (2004*b*)

*flavilabris* (Schiødte, 1839, *Amys*) *nom. ob.*

Horstmann (2004*b*)

*lycorinoides* (Costa, 1886, *Glypta*)

*sardoa* (Costa, 1886, *Glypta*)

Subfamily MESOCHORINAE Förster, 1869[[391]](#footnote-392),[[392]](#footnote-393)

***ASTIPHROMMA*** Förster, 1869[[393]](#footnote-394)

*ASTIPHROMMUS* Thomson, 1886

*MESOCHORELLA* Szépligeti, 1911

*PSEUDACOENITUS* Kiss, 1924

*DEMOPHORELLUS* Hedwig, 1955

***aggressor*** (Fabricius, 1804, *Ophion*) E added by Riedel (2015)

*marginellum* (Holmgren, 1860, *Mesochorus*)

*alpinum* (Roman, 1909, *Mesochorus*)

*barbatum* Schwenke, 1999 Riedel (2015)

*caecum* Schwenke, 1999 Riedel (2015)

***albitarse*** (Brischke, 1880, *Mesochorus*) E S added by Riedel (2015)

*nigrum* Pfankuch, 1921

*heydeni* Habermehl, 1923

*transsylvanicum* (Kiss, 1924, *Pseudacoenitus*)

***alpinum*** (Roman, 1909, *Mesochorus*) E added by Riedel (2015)

*dispersum* Schwenke, 1999 Riedel (2015)

*laricis* Schwenke, 1999 Riedel (2015)

***anale*** (Holmgren, 1860, *Mesochorus*) E S added by Riedel (2015)

***buccatum*** (Thomson, 1886, *Mesochorus*) E

*hamulum* (Thomson, 1886, *Mesochorus*) Riedel (2015)

*consertum* Schwenke, 1999 Riedel (2015)

***hirsutum*** (Bridgman, 1883, *Mesochorus*) E S I

*granigerum* (Thomson, 1886, *Mesochorus*)

Riedel (2015)

***italicum*** Schwenke, 1999 E added by Riedel (2015)

*contum* Schwenke, 1999 Riedel (2015)

***leucogrammum*** (Holmgren, 1860, *Mesochorus*) E S

NMS, added here

***nigrocoxatum*** (Strobl, 1904, *Mesochorus*) E added by Riedel (2015)

*mimulum* (Hedwig, 1955, *Demophorellus*)

***pictum*** (Brischke, 1880, *Mesochorus*) E

*incidens* (Thomson, 1886, *Mesochorus*)

***scutellatum*** (Gravenhorst, 1829, *Mesochorus*) E

*festivum* (Holmgren, 1860, *Mesochorus*)

***splenium*** (Curtis, 1833, *Mesochorus*) E S I

*sericans* (Curtis, 1833, *Mesochorus*)[[394]](#footnote-395)

*strenuum* (Holmgren, 1860, *Mesochorus*)

*plagiatum* (Thomson, 1886, *Mesochorus*) Schwenke (1999)

***tenuicorne*** (Thomson, 1886, *Mesochorus*) E S

***trimaculosum*** Schwenke, 2004 E added by Schwenke (2004)

***uliginosum*** Schwenke, 1999 E W I added by Riedel (2015)

***varipes*** (Holmgren, 1860, *Mesochorus*) E S added by Riedel (2015)

*variipes* Dalla Torre, 1901 preocc.

Species of *Astiphromma* excluded from the British and Irish list

[***dorsale*** (Holmgren, 1860, *Mesochorus*)[[395]](#footnote-396)]

[***striatum*** (Brischke, 1880, *Mesochorus*

*mandibulare* (Thomson, 1886, *Mesochorus*)[[396]](#footnote-397)]

***CIDAPHUS*** Förster, 1869[[397]](#footnote-398)

*PLESIOPHTHALMUS* Förster, 1869

*MATER* Schluz, 1911

*TETRAGONALYS* Morley, 1913

*OPHTHALMOCHORUS* Roman, 1925

***alarius*** (Gravenhorst, 1829, *Mesochorus*) E W

*thuringiacus* Brauns, 1889

***areolatus*** (Boie, 1850, *Paniscus*)[[398]](#footnote-399) E S added by Fitton (1985)

*gigas* (Kriechbaumer, 1897, *Meoschorus*) Horstmann (2002*b*)

*brischkei* (Szépligeti, 1911, *Plesiophthalmus*)

Horstmann (2002*b*)

***atricillus*** (Haliday, 1838, *Cryptus*) E S W

*potanini* Kokujev, 1906

*melanocephalus* (Habermehl, 1909, *Plesiophthalmus*)

***DOLICHOCHORUS*** Strobl, 1904[[399]](#footnote-400)

***longiceps*** (Strobl, 1904, *Mesochorus*) E added by Schwenke (1999)[[400]](#footnote-401)

***MESOCHORUS*** Gravenhorst, 1829[[401]](#footnote-402)

*STICTOPISTHUS* Thomson, 1886

***aggestus*** Schwenke, 2002 E added by Schwenke (1999)

*sulcatus* Schwenke, 1999 preocc.

***albionis*** Schwenke, 1999 E added by Schwenke (1999)

***alpigenus*** Strobl, 1904 S

*compactus* Schwenke, 1999 Horstmann (2001*d*)

***anglicus*** Schwenke, 1999 E added by Schwenke (1999)

***angustatus*** Thomson, 1886

***anomalus*** Holmgren, 1860

***arenarius*** (Haliday, 1839, *Cryptus*) E S I

*nigripes* Ratzeburg, 1852 Horstmann (2006*c*)

*melas* Fonscolombe, 1852

*gibbulus* Holmgren, 1856

***atriventris*** Cresson, 1872 E S I M Shaw (1993); Horstmann (2006*c*)

*sylvarum* (Haliday, 1839, *Cryptus*) preocc. Horstmann (2006*c*)

***basalis*** Curtis, 1833 E

***bracatus*** Schwenke, 1999 I added by Schwenke (1999)

***brevipetiolatus*** Ratzeburg, 1844

***britannicus*** Schwenke, 1999 E added by Schwenke (1999)

***carinatus*** Schwenke, 1999 E added by Schwenke (1999)

***cimbicis*** Ratzeburg, 1844 E S

*confusus* Holmgren, 1860 Horstmann (2006*c*)

*longicauda* Thomson, 1886 Schwenke (1999)

*gallicator* Aubert, 1963 Schwenke (1999)

***dimidiator*** Aubert, 1970 E added by Horstmann (2006*c*)

***discitergus*** (Say, 1835, *Cryptus*) E Horstmann (2003*a*)

*facialis* Bridgman, 1884

*baueri* Schwenke, 1999 Horstmann (2003*a*)

***discolor*** Schwenke, 1999 E added by Schwenke (1999)

***dispar*** Brischke, 1880 I added by Horstmann (2002*c*)

***errabundus*** Hartig, 1838 E S NHM, added here[[402]](#footnote-403)

*politus* misident.

***extensator*** Schwenke, 2002 E added by Schwenke (2002)

***flavescens*** Fonscolombe, 1852 E added by Schwenke (1999)

***formosus*** Bridgman, 1882 E S

*convexicollis* Thomson, 1886 Schwenke (1999)

***fulgurans*** Curtis, 1833 E S I Gauld (1970)

*fulgurans* (Haliday, 1839, *Cryptus*) preocc.

*pectinipes* Thomson, 1886 preocc. Horstmann (2006*c*)

*fulvus* Thomson, 1886 Horstmann (2006*c*)

*suecicus* Dalla Torre, 1901 Horstmann (2006*c*)

***fulgurator*** Horstmann, 2006 E NHM, det. Broad, added here

***fuscicornis*** Brischke, 1880 E S

***fuscus*** Schwenke, 1999 E added by Schwenke (1999); Schwenke (2004)

***gemellus*** Holmgren, 1860 E

*tachypus* Holmgren, 1860

*brevicollis* Thomson, 1886

***giberius*** (Thunberg, 1824, *Ichneumon*) E S I

*thoracicus* Gravenhorst, 1829

*sylvarum* Curtis, 1833 Horstmann (2006*c*)

*marginatus* Thomson, 1886 Schwenke (1999)

***globulator*** (Thunberg, 1824, *Ichneumon*) S M

Horstmann (2004*c*; 2006*c*)

*crassimanus* Holmgren, 1860

*dimidiatus* Holmgren, 1860

*sericeus* Brischke, 1880 Horstmann (2006*c*)

***gracilentus*** Brischke, 1880 reinstated by Horstmann (2006*c*)

***iniquus*** Schwenke, 1999 E S added by Horstmann (2006*c*)

***insularis*** Schwenke, 1999 E added by Schwenke (1999)

***jenniferae*** Schwenke, 2002 E added by Schwenke (2002); Schwenke (2004)

***laricis*** Hartig, 1838 E NHM, det. Horstmann, added here

***latus*** Schwenke, 1999 E added by Schwenke (1999)

***lilioceriphilus*** Schwenke, 2000 E added by Salisbury & Broad (2011)

***liquidus*** Schwenke, 2002 E added by Schwenke (2002)

***nematus*** Schwenke, 2004 E added by Schwenke (2004)

***olerum*** Curtis, 1833 E

*pectoralis* Ratzeburg, 1844 Horstmann (2006*c*)

*rapae* Schwenke, 1999 Horstmann (2008*d*)

***orbitalis*** Holmgren, 1860 E S

***owenae*** Schwenke, 1999 E added by Schwenke (1999)

***oxfordensis*** Schwenke, 1999 E added by Schwenke (1999)

***pallipes*** Brischke, 1880[[403]](#footnote-404) E S W

*stigmaticus* Brischke, 1880 Horstmann (2006*c*)

*brunneus* Brischke, 1880 Horstmann (2006*c*)

*rufipes* Brischke, 1880 Horstmann (2006*c*)

*albipes* Thomson, 1886 Schwenke (1999)

*crassicrus* Thomson, 1886 Horstmann (2006*c*)

***pectinellus*** Horstmann, 2006 E NHM, det. Horstmann, added here

***pectinipes*** Bridgman, 1883 E

***perticatus*** Schwenke, 1999 E added by Schwenke (1999)

***pictilis*** Holmgren, 1860

***politus*** Gravenhorst, 1829 E

***pumilionis*** Schwenke, 1999 E added by Schwenke (1999); Schwenke (2004)

***punctipleuris*** Thomson, 1886[[404]](#footnote-405) E I added by Horstmann (2002*c*)

*nigriceps* Thomson, 1886 preocc. Horstmann (2001*e*)

*thomsonii* Dalla Torre, 1901

*thomsoni* Strobl, 1904 preocc.

*amplitudinis* Schwenke, 1999 Horstmann (2002*c*)

***quercus*** Schwenke, 2004 E added by Schwenke (2004)

***rufoniger*** Brischke, 1880 E S NMS, UM, det. Brock, added here

*brevigena* Thomson, 1886

***rutilus*** Schwenke, 2002 E added by Schwenke (2002)

***scutellaris*** Schwenke, 2004 E added by Schwenke (2004)

***semirufus*** Holmgren, 1860

***stigmator*** (Thunberg, 1824, *Ichneumon*)

*splendidulus* Gravenhorst, 1829 Horstmann (2001*c*)

*pallidus* Brischke, 1880

*stigmaticus* Thomson, 1886 preocc.

*orgyiae* Dalla Torre, 1902

***temporalis*** Thomson, 1886 E

***tenuiscapus*** Thomson, 1886 E S

***testaceus*** Gravenhorst, 1829 E

***tetricus*** Holmgren, 1860 E

*macrurus* Thomson, 1886

***trifoveatus*** Schwenke, 2004 E added by Schwenke (2004)

***unicinctor*** (Thunberg, 1824, *Ichneumon*) E I

*complanatus* (Haliday, 1839, *Cryptus*) Schwenke (1999)

*aciculatus* Bridgman, 1881

*laticeps* Thomson, 1886

***velox*** Holmgren, 1860

***vittator*** (Zetterstedt, 1838, *Tryphon*) M added by Horstmann (2006*c*)

***vitticollis*** Holmgren, 1860 E S W

*hungaricus* Szépligeti, 1914 Schwenke (1999)

***windsorianus*** Schwenke, 2004 E added by Schwenke (2004)

Species excluded from the British and Irish list

[***nuncupator*** (Panzer, 1800, *Ichneumon*) Horstmann (2006*c*)]

Subfamily METOPIINAE Förster, 1869

***CARRIA*** Schmiedeknecht, 1924

***paradoxa*** Schmiedeknecht, 1924 E

***CHORINAEUS*** Holmgren, 1858

***australis*** Thomson, 1887 E W

*flavifrons* Schmiedeknecht, 1925 preocc.

*xanthopsis* (Townes, 1946, *Trieces*)

***brevicalcar*** Thomson, 1887 E S

***cristator*** (Gravenhorst, 1829, *Exochus*) E S W

***flavipes*** Bridgman, 1881 E S

***funebris*** (Gravenhorst, 1829, *Exochus*) E S W

*femoratus* Teunissen, 1948

***hastianae*** Aeschlimann, 1975 E W

***longicornis*** Thomson, 1887 E S

***rhenanus*** Aeschlimann, 1981 E NMS, det. Aeschlimann, added here

***subcarinatus*** Holmgren, 1858 E S

*longicalcar* Thomson, 1887

***talpa*** (Haliday, 1839, *Exochus*) E S I

***COLPOTROCHIA*** Holmgren, 1856

***cincta*** (Scopoli, 1763, *Sphex*) E S

*elegantula* (Schrank, 1781, *Ichneumon*)

*mandator* (Fabricius, 1787, *Ichneumon*) preocc.

*mundator* (Thunberg, 1824, *Ichneumon*)

*affinis* Vollenhoven, 1875

***EXOCHUS*** Gravenhorst, 1829

*Amesolytus* Förster, 1869

***albicinctus*** Holmgren, 1873 E S

*anospilus* Thomson, 1887

*nigricans* (Szépligeti, 1898, *Amesolytus*)

***alpinus*** (Zetterstedt, 1838, *Bassus*) E

***britannicus*** Morley, 1911 E

***carri*** Schmiedeknecht, 1924 E

***citripes*** Thomson, 1877 E NHM, added here

***consimilis*** Holmgren, 1858 E NHM, UM, added here

*parvispina* Thomson, 1887

*decoloratus* Schmiedeknecht, 1924

*subalpinus* Schmiedeknecht, 1924

***erythronotus*** (Gravenhorst, 1820, *Ichneumon*) E

*concinnus* Holmgren, 1858

*pumilus* Holmgren, 1873

*rufidorsum* (Szépligeti, 1898, *Amesolytus*)

*ghigii* Ferrière, 1929

***flavomarginatus*** Holmgren, 1856 E S

***fletcheri*** Bridgman, 1884 E S

*femoralis* Pfankuch, 1925 unavailable

***frontellus*** Holmgren, 1858

***gravipes*** (Gravenhorst, 1820, *Ichneumon*) E S

***gravis*** Gravenhorst, 1829

***intermedius*** Morley, 1911 E

***lentipes*** Gravenhorst, 1829

*cylindricus* Holmgen, 1858

***lictor*** Haliday, 1839 E S I

*pectoralis* Haliday, 1839

*decoratus* Holmgren, 1873

***mitratus*** Gravenhorst, 1829

*affninis* Holmgren, 1858

*australis* Thomson, 1894

*pseudaffinis* Strobl, 1903

*paradoxus* Schmiedeknecht, 1924

*punctifer* Schmiedeknecht, 1924

***nigripalpis*** Thomson, 1887 E

***notatus*** Holmgren, 1858 E S

*woldstedtii* Holmgren, 1873

***pictus*** Holmgren, 1858 E S I

***prosopius*** Gravenhorst, 1829 E S

*maculatus* Brischke, 1871

*procerus* Holmgren, 1873

*dioszeghyi* Kiss, 1926

***ratzeburgi*** Holmgren, 1858 NHM, added here

***rubroater*** Schmiedeknecht, 1924 E I

***semilividus*** Vollenhoven, 1875 E NHM, added here

*longicornis* Thomson, 1887

***septentrionalis*** Holmgren, 1873

***thomsoni*** Schmiedeknecht, 1924 E NHM, added here

*crassicornis* Thomson, 1894 preocc.

***tibialis*** Holmgren, 1858 E S

doubtfully placed species of *Exochus*

[***antiquus*** Haliday, 1839 nom. dub.]

***HYPSICERA*** Latreille, 1829

*METACOELUS* Förster, 1869

*POLYCLISTUS* Förster, 1869

***britannica*** Tolkanitz, 2011 E NMS, det. Aeschlimann, added here[[405]](#footnote-406)

*anglica* (Schmiedeknecht, 1925, *Metacoelus*) preocc.

***curvator*** (Fabricius, 1793, *Ichneumon*) E S M

*mansuetor* (Gravenhorst, 1807, *Ichneumon*)

*affinis* (Zetterstedt, 1838, *Bassus*)

***femoralis*** (Geoffroy, 1785, *Ichneumon*) E

***flaviceps*** (Ratzeburg, 1852, *Exochus*) E S I

***subtilitor*** Aubert, 1969 E added by Aeschlimann (1989)

***ISCHYROCNEMIS*** Holmgren, 1858

*TEROZOA* Förster, 1869

*TERATOZOA* Schulz, 1906

***goesi*** Holmgren, 1858 E added by Broad & Shaw (2005)

***METOPIUS*** Panzer, 1806

subgenus ***CERATOPIUS*** Clément, 1927

***citratus*** (Geoffroy, 1762, *Ichneumon*) E S I

*dissectorius* (Panzer, 1805, *Ichneumon*) Horstmann (2006*a*)

*sicarius* Gravenhorst, 1829

*zagoriensis* Hensch, 1928

subgenus ***METOPIUS*** Panzer, 1806

*PELTOPIUS* Clément, 1927

***anxius*** Wesmael, 1849 E S W I

*intermedius* Förster, 1850

*peltator* Marshall, 1874

subgenus ***PELTASTES*** Illiger, 1807

*TYLOPIUS* Townes, 1959 Horstmann (2001*b*)

***leiopygus*** Förster, 1850 E I

*marchandi* Dominique, 1898

*krapinensis* Hensch, 1928

***pinatorius*** Brullé, 1846 E S I

*meridionalis* Hensch, 1928

*gracilis* Clément, 1930

subgenus ***PELTOCARUS*** Thomson, 1887

*CLEMONTIA* Michener, 1941

***croceicornis*** Thomson, 1887

*chrysopus* (Lewin, 1797, *Ichneumon*) preocc.

***dentatus*** (Fabricius, 1779, *Ichneumon*) E S I

?*fasciatus* (Geoffroy 1785, *Ichneumon*)

?*lunulatus* (Villers, 1789, *Ichneumon*)

*micratorius* (Fabricius, 1804, *Ichneumon*)

*denticularis* (Thunberg, 1824, *Ichneumon*)

*pini* (Curtis, 1824, *Peltastes*)

*incisus* Clément, 1930

***PERIOPE*** Haliday, 1839

*MONOPLECTRON* Holmgren, 1856

*OLIGOPLECTRON* Förster, 1869

*MONOPLECTROCHUS* Heinrich, 1949

***auscultator*** Haliday, 1839 E S I

*zygaenator* (Holmgren, 1856, *Monoplectron*)

***SCOLOMUS*** Townes, 1969

*APOLOPHUS* Townes, 1971 Gauld & Wahl (2006)

***borealis*** (Townes, 1971, *Apolophus*) E S W I added by Owen *et al*. (1981); Broad & Shaw (2005)

***STETHONCUS*** Townes, 1959

***monopicida*** Broad & Shaw, 2005 E S added by Gauld & Sithole (2002)[[406]](#footnote-407)

*sulcator* misident.

***SYNOSIS*** Townes, 1959

***caesiellae*** Broad & Shaw, 2005 E S added by Broad & Shaw (2005)

***fieldi*** Broad & Shaw, 2005 E added by Broad & Shaw (2005)

***parenthesellae*** Broad & Shaw, 2005 E S added by Broad & Shaw (2005)

***TRICLISTUS*** Förster, 1869

***aethiops*** (Gravenhorst, 1829, *Exochus*) E S

***albicinctus*** Thomson, 1887

***anthophilae*** Aeschlimann, 1983 E S W added by Aeschlimann (1983); Shaw (1984)

***areolatus*** Thomson, 1887 E S

***congener*** (Holmgren, 1858, *Exochus*) E S

*meridionator* Aubert, 1960

***epermeniae*** Shaw & Aeschlimann, 1994 E S added by Shaw & Aeschlimann (1994)

***facialis*** Thomson, 1887

***globulipes*** (Desvignes, 1856, *Exochus*) E S W

*holmgreni* (Bohemani, 1863, *Exochus*)

***lativentris*** Thomson, 1887 E

***longicalcar*** Thomson, 1887 E S

***niger*** (Bridgman, 1883, *Exochus*) E S

***pallipes*** Holmgren, 1873 E S

*nitifrons* Thomson, 1887[[407]](#footnote-408)

*pallidipes* Dalla Torre, 1901

***podagricus*** (Gravenhorst, 1829, *Exochus*) E S I

*nigritellus* Holmgren, 1873

***pubiventris*** Thomson, 1887 E S

***pygmaeus*** (Cresson, 1864, *Exochus*) E S W

***spiracularis*** Thomson, 1887 E

***squalidus*** (Holmgren, 1858, *Exochus*) E W

***yponomeutae*** Aeschlimann, 1973 E S

***TRIECES*** Townes, 1946

***thuringiacus*** (Schmiedeknecht, 1925, *Chorinaeus*) E

NMS, det. Aeschlimann, added here

***tricarinatus*** (Holmgren, 1858, *Chorinaeus*) E S I

Subfamily MICROLEPTINAE[[408]](#footnote-409) Townes, 1958

***MICROLEPTES*** Gravenhorst, 1829

*MIOMERIS* Förster, 1869

*MIONOMERIS* Schulz, 1906

*GNATHONIELLA* Schmiedeknecht, 1924

***aquisgranensis*** (Förster, 1871, *Miomeris*) E S W

***rectangulus*** (Thomson, 1888, *Miomeris*) E S W I

*exareolatus* (Strobl, 1903, *Seleucus*) Horstmann (2011*a*)

*egregius* (Schmiedeknecht, 1924, *Gnathoniella*)

***splendidulus*** Gravenhorst, 1829 E S I

*glabriventris* (Thomson, 1888, *Miomeris*)

Subfamily NEORHACODINAE Hedicke, 1922[[409]](#footnote-410)

***NEORHACODES*** Hedicke, 1922[[410]](#footnote-411)

*RHACODES* Ruschka, 1922 preocc.

***enslini*** (Ruschka, 1922, *Rhacodes*) E S I

Subfamily OPHIONINAE Shuckard, 1840[[411]](#footnote-412)

***ENICOSPILUS*** Stephens, 1835[[412]](#footnote-413)

*HENICOSPILUS* Agassiz, 1846

*ALLOCAMPTUS* Förster, 1869

*CYMATONEURA* Kriechbaumer, 1901

*CRYPTOCAMPTUS* Brèthes, 1909

*Amesophilus* Enderlein, 1914

***adustus*** (Haller, 1885, *Ophion*)[[413]](#footnote-414) E S I

*merdarius* misident.

***cerebrator*** Aubert, 1966 E added by Broad & Shaw (2016)

***combustus*** (Gravenhorst, 1829, *Ophion*) E W

***inflexus*** (Ratzeburg, 1844, *Ophion*) E S W

***merdarius*** (Gravenhorst, 1829, *Ophion*) E S

*repentinus* misident.

*tournieri* (Vollenhoven, 1879, *Ophion*)

*rossicus* (Kokujev, 1907, *Henicospilus*)

*contributus* Shestakov, 1926

***myricae*** Broad & Shaw, 2016 E S W added by Broad & Shaw (2016)

***ramidulus*** (Linnaeus, 1758, *Ichneumon*) E S W I

*truncatus* (Poda, 1761, *Sphex*)

*instabilis* (Kokujev, 1907, *Henicospilus*)

***repentinus*** (Holmgren, 1860, *Ophion*) E added by Broad & Shaw (2016)[[414]](#footnote-415)

***undulatus*** (Gravenhorst, 1829, *Ophion*) E

*arcuatus* (Brullé, 1846, *Ophion*)

***EREMOTYLUS*** Förster, 1869

*CAMPTONEURA* Kriechbaumer, 1901 preocc.

*CAMPTONEUROIDES* Strand, 1928

*CLISTORAPHA* Cushman, 1947

***curvinervis*** (Kriechbaumer, 1878, *Ophion*) E added by Horstmann (1981*b*)

*hungaricus* Szépligeti, 1905

*dryobotae* Seyrig, 1926

***marginatus*** (Jurine, 1807, *Anomalon*) E George (1957)

***OPHION*** Fabricius, 1798

*PANISCUS* Schrank, 1802

*STENOPHTHALMUS* Szépligeti, 1905

*PACHYPROTOMA* Kohl, 1906

*APATOPHION* Shestakov, 1926

*PLATOPHION* Hellén, 1926[[415]](#footnote-416)

***areolaris*** Brauns, 1889 S

***brevicornis*** Morley, 1915 E S W

***costatus*** Ratzeburg, 1848 E S W I

***crassicornis*** Brock, 1982 E S W added by Brock (1982)

***forticornis*** Morley, 1915 E I

*baueri* Habermehl, 1930

***longigena*** Thomson, 1888 E S

***luteus*** (Linnaeus, 1758, *Ichneumon*) E S W I M

*distans* Thomson, 1888

*slaviceki* Kriechbaumer, 1892[[416]](#footnote-417)

*pictus* Kokujev, 1906

*calcaratus* Morley, 1915

***minutus*** Kriechbaumer, 1879 E S W

*eremotyloides* Ceballos, 1962

***mocsaryi*** Brauns, 1889 E S W I

*fossulatus* Hedwig, 1957 unavailable

***obscuratus*** Fabricius, 1798 E S W I

*obscurus* Fabricius, 1804

*polyguttator* (Thunberg, 1824, *Ichneumon*)

*flavolineatus* Brullé, 1846

*variegatus* Rudow, 1883

***ocellaris*** Ulbricht, 1926 E S

***parvulus*** Kriechbaumer, 1879 E S W I

***perkinsi*** Brock, 1982 E S W added by Brock (1982)

***pteridis*** Kriechbaumer, 1879 E S W I

***scutellaris*** Thomson, 1888 E S W

*longicornis* Brauns, 1889

*stigmaticus* Morley, 1915

***ventricosus*** Gravenhorst, 1829 E S W I

*impressus* (Thunberg, 1824, *Ichneumon*) preocc.

***STAUROPOCTONUS*** Brauns, 1889

*STAUROPODOCTONUS* Morley, 1913

***bombycivorus*** (Gravenhorst, 1829, *Ophion*) E

*infuscatus* (Taschenberg, 1875, *Ophion*)

Subfamily ORTHOCENTRINAE Förster, 1869[[417]](#footnote-418)

HELICTINAE Gupta, 1987

***ANISERES*** Förster, 1871

***lapponicus*** Jussila, 1994[[418]](#footnote-419) S NMS, added here

***pallipes*** Förster, 1871 E NMS, added here

*pallidipes* Dalla Torre, 1901

***APERILEPTUS*** Förster, 1869

***albipalpus*** (Gravenhorst, 1829, *Plectiscus*) E S

*conformis* Förster, 1871

*custoditor* Förster, 1871

*euryzonus* Förster, 1871

*exstirpator* Förster, 1871

*frontalis* Förster, 1871

*fungicola* Förster, 1871

*impacatus* Förster, 1871

*penetrans* Förster, 1871

*placidus* Förster, 1871

*tutorius* Förster, 1871

*vacuus* Förster, 1871

*vittiger* Förster, 1871

*nigricarpus* Strobl, 1904

***impurus*** Förster, 1871 E S

*electus* Förster, 1871

*filiventris* Förster, 1871

*immundus* Förster, 1871

*inamoenus* Förster, 1871

*inclinans* Förster, 1871

*labilis* Förster, 1871

*languidus* Förster, 1871

*notabilis* Förster, 1871

*secretus* Förster, 1871

*sternoxanthus* Förster, 1871

*trivittatus* Strobl, 1904

***infuscatus*** Förster, 1871 E NHM, added here

***microspilus*** Förster, 1871 E S NMS, W.A. Ely coll., added here

*spoliator* Förster, 1871

***vanus*** Förster, 1871 E S W I added by Dasch (1992)

*obliquus* (Thomson, 1888, *Plectiscus*)

***APOCLIMA*** Förster, 1869

***signaticorne*** Förster, 1881 S NMS, added here

***BATAKOMACRUS*** Kolarov, 1986

***caudatus*** (Holmgren, 1858, *Orthocentrus*) E S W

*crassicaudatus* (Kolarov, 1986, *Batakomacrus*)

Broad (2010)

***flaviceps*** (Gravenhorst, 1829, *Orthocentrus*) E

***noyesi*** Broad, 2010 E S added by Broad (2010)

***CATASTENUS*** Förster, 1871

*CATATENUS* misspelling

***femoralis*** Förster, 1871 E S W

***DIALIPSIS*** Förster, 1869

*PARENTYPOMA* Strobl, 1901

***exilis*** Förster, 1871 E S I

*conjuncta* Förster, 1871

*diversa* Förster, 1871

*intermedia* Förster, 1871

*mesomelana* Förster, 1871

*observatrix* Förster, 1871

*pallida* Förster, 1871

*crassipes* (Thomson, 1888, *Plectiscus*)

*femorata* (Strobl, 1901, *Parentypoma*)

***ENTYPOMA*** Förster, 1869

*ENTELECHIA* Förster, 1871

*ENTELECHIUS* Thomson, 1888

*ENTYPOMUS* Thomson, 1888

***robustator*** Aubert, 1968 E NHM, added here

***robustum*** Förster, 1871 E

***suspiciosum*** (Förster, 1871, *Entelechia*) E S

*remotum* (Marshall, 1896, *Bassus*)

***EUSTERINX*** Förster, 1869

subgenus ***Divinatrix*** Rossem, 1987

***inaequalis*** Rossem, 1981 E NMS, NHM, det. Schwarz & Broad, added here

subgenus ***EUSTERINX*** Förster, 1869

***argutula*** Förster, 1871 E

*ambigua* Förster, 1871

*divulgata* Förster, 1871

*intermedia* Förster, 1871

*scitula* Förster, 1871

*subcincta* Förster, 1871

***obscurella*** Förster, 1871

*exigua* Förster, 1871

***oligomera*** Förster, 1871 E I added by Dasch (1992)

*fulvicincta* Förster, 1871

*fulvicornis* Förster, 1871

*moesta* Förster, 1871

*oreophila* Förster, 1871

*speculifera* Förster, 1871

*tenuis* Förster, 1871

subgenus ***HOLOMERISTUS*** Förster, 1869

***refractaria*** Rossem, 1982 E M added by Dasch (1992)

***tenuicincta*** (Förster, 1871, *Holomeristus*) E S

subgenus ***Ischyracis*** Förster, 1869

***bispinosa*** (Strobl, 1901, *Hemiteles*) E added by Dasch (1992)

*alpigena* (Strobl, 1904, *Catomicrus*)

***GNATHOCHORISIS*** Förster, 1869

*LAEPSERUS* Förster, 1869

*ACROBLAPTICUS* Schmiedeknecht, 1911

*BLAPTICUS* misident.

***crassula*** (Thomson, 1888, *Blapticus*) E S I NMS, NHM, added here

***dentifer*** (Thomson, 1888, *Blapticus*) E S

*debilis* (Schmiedeknecht, 1911, *Acroblapticus*)

***HELICTES*** Haliday, 1837

*ENCOPIUS* Schiødte, 1839

*MYRIARTHRUS* Förster, 1869

***borealis*** (Holmgren, 1857, *Megastylus*) E S W

*clypeatus* (Förster, 1871, *Idioxenus*)

*coxalis* (Förster, 1871, *Idioxenus*)

*invalidus* (Förster, 1871, *Idioxenus*)

*polymerus* (Förster, 1871, *Idioxenus*)

*propinquus* (Förster, 1871, *Idioxenus*)

*variator* (Förster, 1871, *Idioxenus*)

*pilicornis* (Thomson, 1888, *Megastylus*)

***erythrostoma*** (Gmelin, 1790, *Ichneumon*) E S W I

*mediator* misident.

*fulvicornis* (Haliday, 1839, *Cryptus*)

*conspicuus* (Förster, 1871, *Idioxenus*)

*inaequalis* (Förster, 1871, *Idioxenus*)

*inquilinus* (Förster, 1871, *Idioxenus*)

*intricator* (Förster, 1871, *Idioxenus*)

*tetraglyptus* (Förster, 1871, *Idioxenus*)

*nigricoxus* Strobl, 1904

***varius*** (Haliday, 1839, *Cryptus*) I

***HEMIPHANES*** Förster, 1869

***erratum*** Humala, 2007 S W I added by Humala *et al.* (2007)[[419]](#footnote-420)

*flavipes* misident.[[420]](#footnote-421)

***gravator*** Förster, 1871 S NMS, det. Brock, added here

*laevithorax* (Strobl, 1903, *Trematopygus*) Horstmann (2012*c*)

***performidatum*** Rossem, 1988 E S added by Humala *et al.* (2007)[[421]](#footnote-422)

***MEGASTYLUS*** Schiødte, 1838

*DICOLUS* Förster, 1869

*IDIOXENUS* Förster, 1869

*MEGALOSTYLUS* Schulz, 1906

*MIOMEROIDES* Kiss, 1924

*MYRIARTHRUS* misident.

***cruentator*** Schiødte, 1838 E S W I

*mediator* Schiødte, 1838

*cruentatus* (Haliday, 1839, *Cryptus*)

*conformis* Förster, 1871

*fuscicornis* Förster, 1871

*nigriventris* Förster, 1871

*pectoralis* (Rudow, 1886, *Hemiteles*) preocc.

***excubitor*** (Förster, 1871, *Dicolus*) E S

***flavopictus*** (Gravenhorst, 1829, *Plectiscus*) E S

*lineator* Schiødte, 1838

*cingulator* (Förster, 1871, *Myriarthrus*)

***impressor*** Schiødte, 1838 E S W

*insectator* (Förster, 1871, *Dicolus*)

***orbitator*** Schiødte, 1838 E S W I added by Rossem (1983*b*)

*maderensis* (Wollaston, 1858, *Mesoleptus*)

*leptoderus* Förster, 1871

*pauxillus* Förster, 1871

*pumilio* Förster, 1871

*retroligatus* Förster, 1871

*rufipleuris* (Förster, 1871, *Myriarthrus*)

***pectoralis*** (Förster, 1871, *Dicolus*) E S I

*subtiliventris* (Förster, 1871, *Dicolus*)

***suecicus*** Rossem, 1983 E S I NMS, NHM, det. Broad, added here

***NEURATELES*** Ratzeburg, 1848

***falcatus*** (Thomson, 1897, *Orthocentrus*) S NHM, added here

***papyraceus*** Ratzeburg, 1848 E

*britteni* (Waterson, 1929, *Stenomacrus*)

***ORTHOCENTRUS*** Gravenhorst, 1829

*ATMETUS* Förster, 1869

*PHAENOSEMUS* Förster, 1869

*TAPINOPS* Förster, 1869

***asper*** (Gravenhorst, 1829, *Exochus*) E I

*discolor* Holngren, 1858

***attenuatus*** Holmgren, 1858 E

***corrugatus*** Holmgren, 1858 E

***frontator*** (Zetterstedt, 1838, *Tryphon*) E S I

*repentinus* Holmgren, 1858

*frontalis* Brischke, 1871

*pirasii* Costa, 1886

***fulvipes*** Gravenhorst, 1829 E S I

*anomalus* Gravenhorst, 1829

***marginatus*** Holmgren, 1858 E

***monilicornis*** Holmgren, 1858 E I

***petiolaris*** Thomson, 1897 E

***protervus*** Holmgren, 1858 E

***radialis*** Thomson, 1897 E

***sannio*** Holmgren, 1858 E S

*histrio* Holmgren, 1858

***spurius*** Gravenhorst, 1829 E S

*protuberans* Holmgren, 1858

***winnertzii*** Förster, 1850 E S

*stigmaticus* Holmgren, 1858 Horstmann (2002*c*)

*borealis* Roman, 1915

*meridionator* Aubert, 1960

***PANTISARTHRUS*** Förster, 1871

***dispar*** Rossem, 1981 E NMS, added here

***lubricus*** (Förster, 1871, *Aniseres*) E S

*inaequalis* Förster, 1871

*ochropus* Förster, 1871

*pseudochropus* Strobl, 1904

*subalpinus* (Strobl, 1904, *Aniseres*)

***luridus*** Förster, 1871 E S I

***PICROSTIGEUS*** Förster, 1869[[422]](#footnote-423)

***brevicauda*** Horstmann, 1994 E added by Horstmann (1994*b*)

***debilis*** (Gravenhorst, 1829, *Orthocentrus*) E S I

***obscurus*** Horstmann, 1994 E S W I added by Horstmann (1994*b*)

***recticauda*** (Thomson, 1897, *Orthocentrus*) E S W I

***setiger*** (Brischke, 1871, *Orthocentrus*) E I added by Horstmann (1994*b*)

***PLECTISCIDEA*** Viereck, 1914

subgenus ***FUGATRIX*** Rossem, 1987

***communis*** (Förster, 1871, *Plectiscus*) E S I

*elumbis* (Förster, 1871, *Plectiscus*)

*gilva* (Förster, 1871, *Plectiscus*)

*infirma* (Förster, 1871, *Plectiscus*)

*nigrita* (Förster, 1871, *Plectiscus*)

*parviceps* (Förster, 1871, *Plectiscus*)

*tantilla* (Förster, 1871, *Plectiscus*)

subgenus ***PLECTISCIDEA*** Viereck, 1914

*EPHALMATOR* Rossem, 1981

***aquilonia*** Humala, 2003 E W NHM, det. Broad, added here

***bistriata*** (Thomson, 1888, *Plectiscus*) E

***canaliculata*** (Förster, 1871, *Plectiscus*)

*distincta* (Förster, 1871, *Plectiscus*)

*subcurvata* (Förster, 1871, *Plectiscus*)

*subtilis* (Förster, 1871, *Plectiscus*)

***collaris*** (Gravenhorst, 1829, *Plectiscus*) E S W

*binodula* (Förster, 1871, *Plectiscus*)

***conjuncta*** (Förster, 1871, *Plectiscus*) E NMS, added here

*flavicoxis* (Förster, 1871, *Plectiscus*)

***eurystigma*** (Thomson, 1888, *Plectiscus*)

***humeralis*** (Förster, 1871, *Plectiscus*)

*fulva* (Förster, 1871, *Plectiscus*)

*hostilis* (Förster, 1871, *Plectiscus*)

**?*hyperborea*** (Holmgren, 1869, *Plectiscus*)[[423]](#footnote-424)

***melanocera*** (Förster, 1871, *Plectiscus*) E

*proxima* (Förster, 1871, *Plectiscus*)

***moerens*** (Förster, 1871, *Plectiscus*) E NHM, added here[[424]](#footnote-425)

*eversoria* (Förster, 1871, *Plectiscus*)

*flavizona* (Förster, 1871, *Plectiscus*)

*xanthoneuris* (Förster, 1871, *Plectiscus*)

***subteres*** (Thomson, 1888, *Plectiscus*)

***tener*** (Förster, 1871, *Plectiscus*) E added by Rossem (1988)

*incerta* (Förster, 1871, *Plectiscus*)

***tenuicornis*** (Förster, 1871, *Plectiscus*) E

*brachyura* (Förster, 1871, *Plectiscus*)

***terebrator*** (Förster, 1871, *Plectiscus*) E

*habilis* (Förster, 1871, *Plectiscus*)

*praeposita* (Förster, 1871, *Plectiscus*)

***ventosa*** Rossem, 1987 S NMS, added here

***zonata*** (Gravenhorst, 1829, *Plectiscus*)[[425]](#footnote-426) E NHM, added here

*abscondita* (Förster, 1871, *Proclitus*)

*contemptibilis* (Förster, 1871, *Proclitus*)

*denticulata* (Förster, 1871, *Proclitus*)

*displicita* (Förster, 1871, *Proclitus*)

*humilis* (Förster, 1871, *Proclitus*)

*inaestimabilis* (Förster, 1871, *Proclitus*)

*inferior* (Förster, 1871, *Proclitus*)

*marginata* (Förster, 1871, *Proclitus*)

*punctata* (Förster, 1871, *Proclitus*)

*sordida* (Förster, 1871, *Proclitus*)

*exareolata* Aubert, 1979

species excluded from the British and Irish list

[***amicalis*** (Förster, 1871, *Plectiscus*)[[426]](#footnote-427)

*sodalis* (Förster, 1871, *Plectiscus*)]

***PLECTISCUS*** Gravenhorst, 1829

*BREPHOCTONUS* Förster, 1869

*LEIPAULUS* Townes, 1945

***agilis*** (Holmgren, 1858, *Orthocentrus*) I

*flavicornis* (Thomson, 1897, *Orthocentrus*)

***impurator*** Gravenhorst, 1829 E S W I

*ventralis* (Holmgren, 1858, *Orthocentrus*)

*vittatus* (Holmgren, 1858, *Orthocentrus*)

***ridibundus*** (Gravenhorst, 1829, *Orthocentrus*) E S W I

*exilis* (Holmgren, 1858, *Orthocentrus*)

***PROCLITUS*** Förster, 1869

*CLEPTICUS* Haliday, 1839 preocc.

*ACLASTONEURA* Kriechbaumer, 1896

***attentus*** Förster, 1871 E S

*fossulatus* Förster, 1871

*gracilentus* Förster, 1871

*leptosomus* Förster, 1871

*melanocephalus* Förster, 1871

*mesoxanthus* Förster, 1871

*procerulus* Förster, 1871

*quaestorius* Förster, 1871

*sincerus* Förster, 1871

*stenogaster* Förster, 1871

*substriatus* Förster, 1871

*vallidus* Förster, 1871

***comes*** (Haliday, 1839, *Cryptus*) E S I

*caudiger* Förster, 1871

*macrurus* Förster, 1871

*pallens* Förster, 1871

*perditorius* Förster, 1871

***edwardsi*** Roman, 1923 E

***fulvicornis*** Förster, 1871[[427]](#footnote-428) E I

*cupidus* Förster, 1871

*evacuator* Förster, 1871

*inquietus* Förster, 1871

*periculosus* Förster, 1871

*ruficaudator* Aubert, 1963

***paganus*** (Haliday, 1839, *Cryptus*) E S I

*autumnalis* Förster, 1871

*clypearis* Förster, 1871

*conturbator* Förster, 1871

*curiosus* Förster, 1871

*dimidiatus* Förster, 1871

*instigator* Förster, 1871

*providus* Förster, 1871

*longitarsis* (Thomson, 1888, *Plectiscus*)

***praetor*** (Haliday, 1839, *Cryptus*) E S W I

*grandis* Förster, 1871

***socius*** (Haliday, 1839, *Cryptus*) I NHM, added here[[428]](#footnote-429)

***PROELIATOR*** Rossem, 1982

***proprius*** Rossem, 1982 E NHM, det. Broad, added here

***STENOMACRUS*** Förster, 1869

***binotatus*** (Holmgren, 1858, *Orthocentrus*) E

***carbonariae*** Roman, 1939 E

***celer*** (Holmgren, 1858, *Orthocentrus*) E S NHM, added here

***cognatus*** (Holmgren, 1858, *Orthocentrus*) E

*confinis* (Holmgren, 1858, *Orthocentrus*)

*tristis* (Holmgren, 1858, *Orthocentrus*)

***cubiceps*** (Thomson, 1897, *Orthocentrus*) E I

***curvicaudatus*** (Brischke, 1871, *Orthocentrus*) E

***curvulus*** (Thomson, 1897, *Orthocentrus*) E W

NHM, det. Perkins, Broad, added here

***deletus*** (Thomson, 1897, *Orthocentrus*)

***holmgreni*** (Kirchner, 1867, *Orthocentrus*) E

*lapponicus* Horstmann & Yu, 1999 Horstmann (2006*d*)

*intermedius* (Holmgren, 1858, *Orthocentrus*) preocc.

***incisus*** (Gravenhorst, 1829, *Orthocentrus*)

***innotatus*** (Thomson, 1897, *Orthocentrus*)

***laricis*** (Haliday, 1839, *Bassus*) E S I

*concinnus* (Holmgren, 1858, *Orthocentrus*)

*fortipes* (Thomson, 1897, *Orthocentrus*)

***molestus*** (Holmgren, 1858, *Orthocentrus*)

***ochripes*** (Holmgren, 1858, *Orthocentrus*)

***palustris*** (Holmgren, 1858, *Orthocentrus*) E

***pedestris*** (Holmgren, 1869, *Orthocentrus*) E

*reptilis* (Marshall, 1877, *Orthocentrus*)

***pygmaeus*** Horstmann & Yu, 1999 E NHM, added here

*pusillus* (Zetterstedt, 1838, *Bassus*) preocc.

***silvaticus*** (Holmgren, 1858, *Orthocentrus*) E S

***vafer*** (Holmgren, 1858, *Orthocentrus*) S NHM, added here

[species excluded from the British and Irish list]

[***affinis*** misident.[[429]](#footnote-430)]

[***exserens*** (Thomson, 1897, *Orthocentrus*)[[430]](#footnote-431)]

***SYMPLECIS*** Förster, 1869

*BLAPTICUS* Förster, 1869

***bicingulata*** (Gravenhorst, 1829, *Mesoleptus*)[[431]](#footnote-432) E S I[[432]](#footnote-433)

*facialis* Thomson, 1888

*albicoxis* (Kiss, 1924, *Rhaestes*) Horstmann (2007*a*)

***breviuscula*** Roman, 1923 E

*breviscula* misspelling

*infavorabilis* Rossem, 1981

Subfamily ORTHOPELMATINAE Schmiedeknecht, 1910

***ORTHOPELMA*** Taschenberg, 1865[[433]](#footnote-434)

*PROEDRUS* Förster, 1869

***brevicorne*** Morley, 1907 E W

***mediator*** (Thunberg, 1824, *Ichneumon*) E S W I

*bedeguaris* (Geoffroy, 1785, *Ichneumon*) preocc.

*luteolator* (Gravenhorst, 1829, *Hemiteles*)

*rufinum* (Gravenhorst, 1829, *Porizon*)

*pavoniae* (Rondani, 1877, *Hemiteles*)

Subfamily OXYTORINAE Thomson, 1883[[434]](#footnote-435)

***OXYTORUS*** Förster, 1869

*CALLIDIOTES* Förster, 1869

*DELOLYTUS* Förster, 1869

*PANTOPORTHUS* Förster, 1869

*MESATRACTODES* Morley, 1907

***armatus*** Thomson, 1883 E S W

***luridator*** (Gravenhorst, 1820, *Ichneumon*) E S W

*coxator* (Gravenhorst, 1829, *Mesoleptus*)

*ventrator* (Gravenhorst, 1829, *Mesoleptus*)

*properator* (Haliday, 1839, *Atractodes*)

*varicornis* (Holmgren, 1860, *Atractodes*)

*longicornis* (Habermehl, 1909, *Exolytus*)

*nigricoxa* (Kiss, 1924, *Callidiotes*)

Subfamily PIMPLINAE Wesmael, 1845[[435]](#footnote-436)

EPHIALTINAE Hellén, 1915

Tribe DELOMERISTINI Hellén, 1915

PERITHOINI Wahl & Gauld, 1998 Gauld *et al.* (2002)

***DELOMERISTA*** Förster, 1869[[436]](#footnote-437)

***borealis*** Walkley, 1960 E NHM, det. Broad, added here

***laevis*** (Gravenhorst, 1829, *Pimpla*) E added by Horstmann (2001*d*)[[437]](#footnote-438)

*suborbitalis* (Gravenhorst, 1829, *Lissonota*)

Horstmann (2001*d*)

*laevifrons* (Thomson, 1877, *Pimpla*)

*levifrons* (Dalla Torre, 1901, *Pimpla*) preocc.

***mandibularis*** (Gravenhorst, 1829, *Pimpla*) E anon. (2001)

*albicinctus* (Desvignes, 1862, *Ephialtes*) preocc.

*desvignesii* (Marshall, 1870, *Ephialtes*)

***novita*** (Cresson, 1870, *Pimpla*) E S I

*laevis* misident.

*europa* Gupta, 1982[[438]](#footnote-439)

***pfankuchi*** Brauns, 1905 E S added by Fitton *et al.* (1988)

*unicolor* (Hedwig, 1959, *Troctocerus*)

***PERITHOUS*** Holmgren, 1859

*HYBOMISCHOS* Baltazar, 1961 Wahl & Gauld (1998)

*HYBOISCHOS* misspelling

***albicinctus*** (Gravenhorst, 1829, *Ephialtes*) E added by Brock & Shaw (1997)

***divinator*** (Rossi, 1790, *Ichneumon*) E I Field & Foster (1988)

*ephippiatorius* (Dufour & Perris, 1840, *Pimpla*)

*amoenus* (Rudow, 1881, *Pimpla*)

*rubi* (Habermehl, 1917, *Itoplectis*)

*pimplarius* Haupt, 1938

***scurra*** (Panzer, 1804, *Ichneumon*) E S W I Formstone (1999)

*mediator* (Fabricius, 1804, *Pimpla*)

*asilatorius* (Thunberg, 1824, *Ichneumon*)

*modulator* (Thunberg, 1824, *Ichneumon*)

*senator* (Haliday, 1839, *Pimpla*)

*decoratus* (Ratzeburg, 1848, *Pimpla*)

*longiseta* Haupt, 1954

*moldavicus* Constantineanu & Constantineanu, 1968

***septemcinctorius*** (Thunberg, 1824, *Ichneumon*) E I

*varius* (Gravenhorst, 1829, *Ephialtes*)

*marginellatorius* (Dufour & Perris, 1840, *Pimpla*)

*brunnescens* Koornneef, 1951

*exiguus* Haupt, 1954

*meridionator* Aubert, 1963

*rufatus* Constantineanu & Constantineanu, 1968

***speculator*** Haupt, 1954 E added by Shaw (2006*b*)

*transsylvanicus* Constantineanu & Constantineanu, 1968

Tribe EPHIALTINI Hellén, 1915

POLYSPHINCTINI Hellén, 1915 Wahl & Gauld, 1998

***ACRODACTYLA*** Haliday, 1839[[439]](#footnote-440)

*BARYPUS* Haliday, 1837 preocc.

*COLPOMERIA* Holmgren, 1859

*SYMPHYLUS* Förster, 1869 preocc.

*POLEMOPHTHORUS* Schulz, 1911

*PANTOMIMA* Rossem, 1990 Broad (2004)

***carinator*** (Aubert, 1965, *Colpomeria*) E W I added by Shaw (2006*b*)

*braconiformis* Kolarov, 1990 Zwakhals (2006)

***degener*** (Haliday, 1839, *Pimpla*) E S W I M

*hadrodactyla* (Förster, 1871, *Symphylus*)

*festata* (Rossem, 1990, *Pantomima*) Broad (2004)

***quadrisculpta*** (Gravenhorst, 1820, *Ichneumon*) E S W I M

*laevigata* (Holmgren, 1859, *Colpomeria*)

***similis*** Horstmann, 2011 E S W added by Horstmann (2011*a*)

***ACROPIMPLA*** Townes, 1960

*SELANASPIS* Roman, 1910 preocc.

***didyma*** (Gravenhorst, 1829, *Pimpla*) E W I

***CLISTOPYGA*** Gravenhorst, 1829

*HYMENOMACROPYGA* Uchida, 1941

*ICHNEUMONOGLYPTA* Blanchard, 1941

***canadensis*** Provancher, 1880 E

*sauberi* Brauns, 1898 Bordera *et al.* (2014)

*terebralis* Shestakov, 1927

***incitator*** (Fabricius, 1793, *Ichneumon*) E S W I M

*haemorrhoidalis* Gravenhorst, 1829

*elegans* (Ratzeburg, 1848, *Polysphincta*)

*incitatrix* Schulz, 1906

*excavata* (Telenga, 1930, *Polysphincta*)

*temporalis* Hellén, 1949

***rufator*** Holmgren, 1856 E W

*rufatrix* Schulz, 1906

***DOLICHOMITUS*** Smith, 1877

*CLOSTEROCERUS* Hartig, 1847 preocc.

*MESOEPHIALTES* Schmiedeknecht, 1906

*DICLOSTEROCERUS* Viereck, 1914

*EXERISTOIDEA* Viereck, 1924

*TUBERCULEPHIALTES* Ozols, 1962

*PAUCDOLICHOMITUS* Constantineanu & Pisica, 1970

***agnoscendus*** (Roman, 1939, *Ephialtes*) E W I

***diversicostae*** (Perkins, 1943, *Ephialtes*) S

***imperator*** (Kriechbaumer, 1854, *Ephialtes*) E S

?*adulterator* (Villers, 1789, *Ichneumon*)

?*gracilis* (Gmelin, 1790, *Ichneumon*)

?*melanopus* (Gmelin, 1790, *Ichneumon*)

***mesocentrus*** (Gravenhorst, 1829, *Ephialtes*) E W

*rex* (Kriechbaumer, 1854, *Ephialtes*)

*insignis* (Habermehl, 1903, *Ephialtes*)

*krapinensis* (Hensch, 1930, *Ephialtes*)

*gaurottii* (Gregor, 1941, *Ephialtes*)

***messor*** (Gravenhorst, 1829, *Ephialtes*) E

*continuus* (Ratzeburg, 1848, *Ephialtes*)

*reissigii* (Ratzeburg, 1848, *Pimpla*)

*pusillus* (Ratzeburg, 1852, *Ephialtes*)

*heteropus* (Thomson, 1888, *Ephialtes*)

*simillimus* (Hensch, 1930, *Ephialtes*)

*zagoriensis* (Hensch, 1930, *Ephialtes*)

***populneus*** (Ratzeburg, 1848, *Ephialtes*) E

*abbreviatus* (Thomson, 1877, *Ephialtes*)

***pterelas*** (Say, 1829, *Ichneumon*) E I

*discrepans* (Hensch, 1929, *Ephialtes*)

***terebrans*** (Ratzeburg, 1844, *Pimpla*) E S W

*planifrons* (Thomson, 1877, *Ephialtes*)

*borealis* (Hellén, 1915, *Ephialtes*)

*kangasi* (Györfi, 1941, *Pimpla*)

***tuberculatus*** (Geoffroy, 1785, *Ichneumon*) E S W I

*hyalinus* (Gmelin, 1790, *Ichneumon*)

*leucopterus* (Gmelin, 1790, *Ichneumon*)

*crispus* (Christ, 1791, *Ichneumon*)

*fluctuans* (Christ, 1791, *Ichneumon*)

*parallelus* (Thomson, 1888, *Ephialtes*)

*dentiventris* (Hellén, 1915, *Ephialtes*)

*pfefferi* (Habermehl, 1917, *Ephialtes*)

***DREISBACHIA*** Townes, 1962[[440]](#footnote-441)

*LAUFEIA* Tosquinet, 1903 preocc.

***pictifrons*** (Thomson, 1877, *Pimpla*) E S I

*bridgmani* (Bignell, 1894, *Pimpla*)

***ENDROMOPODA*** Hellén, 1939

***arundinator*** (Fabricius, 1804, *Pimpla*) E I

*melanopyga* (Gravenhorst, 1829, *Pimpla*)

*erythrosoma* (Rudown, 1883, *Pimpla*)

*arundinatrix* (Schulz, 1906, *Pimpla*)

*nigricans* (Ulbricht, 1913, *Pimpla*) preocc.

*culpator* (Morley, 1914, *Epiurus*)

*crefeldensis* (Strand, 1918, *Pimpla*)

***detrita*** (Holmgren, 1860, *Pimpla*) E S W I M

*laevidorsum* (Vollenhoven, 1873, *Pimpla*)

*brunnea* (Brischke, 1880, *Pimpla*)

*punctator* (Müller, 1766, *Ichneumon*) Horstmann (2001*c*)

***nigricoxis*** (Ulbricht, 1910, *Pimpla*) E S W I M added by Fitton *et al.* (1988)

*melanopyga* (Ulbricht, 1909, *Pimpla*) preocc.

*ulbrichtiana* (Strand, 1918, *Pimpla*)

***nitida*** (Brauns, 1898, *Pimpla*) E W

*deplanata* (Morley, 1908, *Pimpla*)

***phragmitidis*** (Perkins, 1957, *Ephialtes*) E W

*rufipes* (Aubert, 1963, *Scambus*)

***EPHIALTES*** Gravenhorst, 1829

*APECHTHIS* misident.

***duplicauda*** Heinrich, 1949 I added by Horstmann (2008*b*)

*spatulata* (Townes, 1960, *Pimpla*) Horstmann (2008*b*)

***manifestator*** (Linnaeus, 1758, *Ichneumon*) E W[[441]](#footnote-442)

*extricator* (Villers, 1789, *Ichneumon*)

*leucopalpus* (Gmelin, 1790, *Ichneumon*)

*carbonarius* (Christ, 1791, *Ichneumon*) preocc.

*gracilis* (Schrank, 1802, *Ichneumon*) preocc.

*nepotor* (Thunberg, 1824, *Ichneumon*)

*elongator* (Zetterstedt, 1838, *Pimpla*)

***EXERISTES*** Förster, 1869

*EREMOCHILA* Förster, 1869

***ruficollis*** (Gravenhorst, 1829, *Pimpla*) E S W

*variegatus* (Ratzeburg, 1844, *Pimpla*)

***FLAVOPIMPLA*** Betrem, 1932

*AFREPHIALTES* Benoit, 1953[[442]](#footnote-443)

***cicatricosa*** (Ratzeburg, 1848, *Pimpla*) E I O'Connor & Shaw (2004)[[443]](#footnote-444)

***FREDEGUNDA*** Fitton, Shaw & Gauld, 1988

***diluta*** (Ratzeburg, 1852, *Pimpla*) E W

*nigriceps* (Taschenberg, 1863, *Pimpla*) preocc.

*media* (Verhoeff, 1891, *Pimpla*)

*taschenbergii* (Dalla Torre, 1901, *Pimpla*)

***GREGOPIMPLA*** Momoi, 1965

***inquisitor*** (Scopoli, 1763, *Ichneumon*) E S I

?*visitator* (Poda, 1761, *Ichneumon*)

?*visitator* (Scopoli, 1763, *Ichneumon*) preocc.

*scanica* (Geoffroy, 1785, *Ichneumon*)

*albipes* (Gmelin, 1790, *Ichneumon*) preocc.

*annulata* (Gmelin, 1790, *Ichneumon*)

*aurifrons* (Gmelin, 1790, *Ichneumon*)

*variegata* (Gmelin, 1790, *Ichneumon*) preocc.

*perquisitor* (Olivier, 1792, *Ichneumon*)

*pennator* (Fabricius, 1793, *Ichneumon*) preocc.

*pinnator* (Thunberg, 1824, *Ichneumon*)

*flavipes* (Gravenhorst, 1829, *Pimpla*)

*pini* (Hartig, 1838, *Pimpla*)

*pudibundae* (Ratzeburg, 1848, *Pimpla*)

*inquisitrix* (Schulz, 1906, *Pimpla*)

***ISEROPUS*** Förster, 1869

*CNEMOPIMPLA* Cameron, 1903

***stercorator*** (Fabricius, 1793, *Ichneumon*) E S W I

*graminellae* (Schrank, 1802, *Ichneumon*)

*mussii* (Hartig, 1838, *Pimpla*)

*holmgreni* (Schmiedeknecht, 1888, *Pimpla*)

***LIOTRYPHON*** Ashmead, 1900

*LIOGASTER* Kriechbaumer, 1890 preocc.

*APISTES* Seyrig, 1927 preocc.

*APISTEPHIALTES* Seyrig, 1928

*NEOEPHIALTES* Constantineanu & Pisica, 1970

***ascaniae*** (Rudow, 1883, *Ephialtes*) E S

*ruficollis* (Desvignes, 1856, *Ephialtes*) invalid

*sanguinicollis* (Brauns, 1901, *Ephialtes*)

*perversus* (Seyrig, 1927, *Apistes*)

***caudatus*** (Ratzeburg, 1848, *Pimpla*) E S

*brevivalvis* (Hensch, 1929, *Ephialtes*)

*incertus* (Hensch, 1929, *Ephialtes*)

*foveolatus* (Constantineanu & Pisica, 1970, *Neoephialtes*) preocc.

***crassiseta*** (Thomson, 1877, *Ephialtes*) E I Godfrey & Whitehead (2001)

*pleuralis* (Thomson, 1877, *Ephialtes*)

*musculus* (Kriechbaumer, 1889, *Ephialtes*)

*albispiculus* (Morley, 1908, *Ephialtes*)

*taschenbergi* (Ulbricht, 1909, *Ephialtes*) preocc., unavailable

*sternoleucus* (Lange, 1911, *Ephialtes*)

*foveolatus* (Ulbricht, 1912, *Ephialtes*)

*taschenbergella* (Strand, 1918, *Pimpla*)

*rufipes* (Hensch, 1930, *Ephialtes*)

***punctulatus*** (Ratzeburg, 1848, *Pimpla*) E

*discolor* (Brischke, 1880, *Ephialtes*)

*macrurus* (Förster, 1888, *Epiurus*)

*longulus* (Kriechbaumer, 1890, *Liogaster*)

*tener* (Hensch, 1929, *Ephialtes*)

*vernalis* (Hensch, 1929, *Ephialtes*)

*gracilentus* (Hensch, 1930, *Ephialtes*)

[***strobilellae*** (Linnaeus, 1758, *Ichneumon*)[[444]](#footnote-445)

*resinosus* (Retzius, 1783, *Ichneumon*) Horstmann (2003*b*)

*strobilator* (Thunberg, 1824, *Ichneumon*)

*glabratus* (Ratzeburg, 1852, *Ephialtes*)

*zhedenevensis* (Shestakov, 1927, *Ephialtes*)

*discedens* (Hensch, 1930, *Ephialtes*)]

***MEGAETAIRA*** Gauld & Dubois, 2006[[445]](#footnote-446)

***madida*** (Haliday, 1839, *Pimpla*) E S I

*clypeata* (Holmgren, 1860, *Polysphincta*)

***OXYRRHEXIS*** Förster, 1869

***carbonator*** (Gravenhorst, 1807, *Cryptus*) E added by Shaw (1998*b*)[[446]](#footnote-447)

*velata* (Hartig, 1838, *Polysphincta*)

*pusilla* (Fonscolombe, 1854, *Polysphincta*)

*carbonatrix* (Schulz, 1906, *Acrodactyla*)

***PARAPERITHOUS*** Haupt, 1954

*GNATHAULAX* Townes, 1964

***gnathaulax*** (Thomson, 1877, *Ephialtes*) E S

*luteipes* (Thomson, 1877, *Ephialtes*)

*ruficollis* (Rudow, 1881, *Ephialtes*) preocc.

*aterrimus* (Haupt, 1954, *Perithous*)

*moldavicus* Constantineanu & Pisica, 1970

***PIOGASTER*** Perkins, 1958

***albina*** Perkins, 1958 E

***punctulata*** Perkins, 1958 E

***POLYSPHINCTA*** Gravenhorst, 1829

***boops*** Tschek, 1869 E S

*eltshaninovi* Shestakov, 1927

***longa*** Kasparyan, 1976 E W added by Fritzén & Shaw (2014)[[447]](#footnote-448)

***rufipes*** Gravenhorst, 1829 E S W I

*drewseni* Holmgren, 1860

***tuberosa*** Gravenhorst, 1829 E S W I M

*taschenbergi* Woldstedt, 1877

*sculpturata* Roman, 1931

***vexator*** Fitton, Shaw & Gauld, 1988 E W I added by Fitton *et al.* (1988)

***RECLINERVELLUS*** He & Ye, 1998

***nielseni*** (Roman, 1923, *Polysphincta*)[[448]](#footnote-449) E added by Fitton *et al.* (1988)

***SCAMBUS*** Hartig, 1838

*EPIURUS* Förster, 1869

*TROMERA* Förster, 1869

*TROCTOCERUS* Woldstedt, 1877

*ATELEOPHADNUS* Cameron, 1905

*PSEUDOPOEMENIA* Kiss, 1924

*ERYTHROSCAMBUS* Walley, 1930

*LISSOSCAMBUS* Walley, 1930

***brevicornis*** (Gravenhorst, 1829, *Pimpla*)[[449]](#footnote-450) E S W I M

*concolor* (Ratzeburg, 1848, *Pimpla*) preocc.

*nigriscaposus* (Thomson, 1877, *Pimpla*)

*punctiventris* (Thomson, 1877, *Pimpla*)

*agilis* (Förster, 1888, *Epiurus*)

*centaureae* (Förster, 1888, *Epiurus*) Horstmann (2010*a*)

*depositor* (Förster, 1888, *Epiurus*)

*infestus* (Förster, 1888, *Epiurus*)

*anomalus* (Morley, 1906, *Phthorimus*)

*tibialis* (Ulbricht, 1910, *Pimpla*) unavailable

*puniceus* (Schmiedeknecht, 1914, *Pimpla*)

*pratensis* (Pfankuch, 1921, *Pimpla*) unavailable

*terrestris* (Pfankuch, 1921, *Pimpla*) unavailable

*ribesii* (Hensch, 1929, *Pimpla*)

***buolianae*** (Hartig, 1838, *Pimpla*) E S

*triangularis* (Verhoeff, 1890, *Pimpla*)

*flavotrochanteratus* (Pfeffer, 1913, *Pimpla*)

***calobatus*** (Gravenhorst, 1829, *Pimpla*) E

*planatus* (Hartig, 1838, *Pimpla*) Shaw *et al.* (2011)

*ghilianii* (Spinola, 1843, *Pimpla*)

*nucum* (Ratzeburg, 1844, *Pimpla*)

*longiventris* (Ratzeburg, 1848, *Pimpla*)

*cingulatus* (Ratzeburg, 1852, *Pimpla*)

*ventricosus* (Tschek, 1871, *Pimpla*)[[450]](#footnote-451) Shaw *et al.* (2011)

*gallicola* (Giraud, 1872, *Pimpla*)

*stramentarius* (Kriechbaumer, 1890, *Pimpla*)

*zonatus* (Habermehl, 1903, *Pimpla*) preocc.

*calobatarius* (Kokujev, 1913, *Pimpla*)

*zonatellus* (Schmiedeknecht, 1914, *Pimpla*)

*nigricoxis* (Habermehl, 1918, *Epiurus*)

***cincticarpus*** (Kriechbaumer, 1895, *Pimpla*) E added by Fitton *et al.* (1988)

*affinis* (Habermehl, 1903, *Pimpla*)

*divergens* (Hensch, 1929, *Pimpla*)

***elegans*** (Woldstedt, 1877, *Troctocerus*) E W I

*albicrus* (Rondani, 1877, *Ephialtes*)

*cingulatellus* (Costa, 1885, *Pimpla*)

*erythronotus* (Förster, 1888, *Epiurus*)

*ulicicida* (Morley, 1911, *Pimpla*)

*cottei* (Seyrig, 1926, *Pimpla*)

*dumeticola* (Hensch, 1929, *Pimpla*)

*zagoriensis* (Hensch, 1929, *Troctocerus*)

***eucosmidarum*** (Perkins, 1957, *Ephialtes*) E S W I

***foliae*** (Cushman, 1938, *Epiurus*) E S M added by Fitton *et al.* (1988)

***inanis*** (Schrank, 1802, *Ichneumon*) E S W I added by Fitton *et al.* (1988)

*agilis* (Förster, 1888, *Epiurus*) Horstmann (2005*a*)

*depositor* (Förster, 1888, *Epiurus*) Horstmann (2005*a*)

*distinctus* (Förster, 1888, *Epiurus*) Horstmann (2005*a*)

*annulatus* (Kiss, 1924, *Pseudopoemenia*) Horstmann (2005*a*)

*lativentris* (Ulbricht, 1926, *Epiurus*)

*trilobatus* (Keler, 1937, *Pimpla*)

***nigricans*** (Thomson, 1877, *Pimpla*) E S W I M

*similis* (Bridgman, 1884, *Pimpla*)

*fulvus* (Szépligeti, 1898, *Pimpla*)

*lucens* (Szépligeti, 1898, *Pimpla*)

*interruptecallosus* (Strobl, 1902, *Pimpla*)

*kriechbaumeri* (Habermehl, 1903, *Pimpla*) preocc.

*habermehli* (Schmiedeknecht, 1908, *Pimpla*)

*robustus* (Morley, 1908, *Pimpla*) preocc.

*obscuripes* (Hensch, 1929, *Pimpla*)

*singularis* (Hensch, 1929, *Pimpla*)

*sparsator* Aubert, 1965

***pomorum*** (Ratzeburg, 1848, *Pimpla*) E S I

***sagax*** (Hartig, 1838, *Pimpla*) E S

*linearis* (Ratzeburg, 1844, *Pimpla*)

*atrocoxatus* (Pfeffer, 1913, *Pimpla*)

*suecicus* (Roman, 1917, *Epiurus*)

*sanctacrucianus* (Glowacki, 1967, *Ephialtes*)

***signatus*** (Pfeffer, 1913, *Pimpla*) E S[[451]](#footnote-452)

***tenthredinum*** (Goeze, 1776, *Ichneumon*) E S added by Horstmann (2005*a*)[[452]](#footnote-453)

***vesicarius*** (Ratzeburg, 1844, *Pimpla*) E S I

*cryptocampi* (Boie, 1857, *Pimpla*)

*gallicolus* (Morley, 1908, *Pimpla*) preocc.

*ruficoxis* (Ulbricht, 1909, *Pimpla*) unavailable

*rhenanus* (Ulbricht, 1910, *Pimpla*) unavailable

*salicola* (Hensch, 1929, *Pimpla*)

*morleyi* (Schmiedeknecht, 1934, *Pimpla*)

***SCHIZOPYGA*** Gravenhorst, 1829

*AFROSPHINCTA* Benoit, 1953

*SCHIZOPYGOIDES* Kasparyan, 1976

***circulator*** (Panzer, 1800, *Ichneumon*) E S W I

*analis* Gravenhorst, 1829

*circulatrix* Schulz, 1906

***frigida*** Cresson, 1870 E S W I

*atra* Kriechbaumer, 1887

***podagrica*** Gravenhorst, 1829 E S I

*minuta* Gravenhorst, 1829

*silbernageli* (Kiss, 1933, *Polysphincta*) preocc.

***varipes*** Holmgren, 1856[[453]](#footnote-454) I added by Shaw (2006*b*)

***SINARACHNA*** Townes, 1960

***nigricornis*** (Holmgren, 1860, *Polysphincta*) E S

added by Fitton *et al.* (1988)

*caudata* (Thomson, 1888, *Polysphincta*)

***pallipes*** (Holmgren, 1860, *Polysphincta*) E I O’Connor (2004*b*)

***TOWNESIA*** Ozols, 1962

***tenuiventris*** (Holmgren, 1860, *Ephialtes*) E S I

*geniculata* (Brischke, 1865, *Ephialtes*)

*antefurcalis* (Thomson, 1877, *Ephialtes*)

*gracilis* (Hensch, 1930, *Ephialtes*)

***TROMATOBIA*** Förster, 1869

*AUSTROPIMPLA* Brèthes, 1913

***forsiusi*** (Hellén, 1915, *Polysphincta*) S added by Fitton *et al.* (1988)

***lineatoria*** (Villers, 1789, *Ichneumon*) E S W I M

*oculatoria* misident. Horstmann (2001*b*)[[454]](#footnote-455)

*tipulatoria* (Thunberg, 1824, *Ichneumon*)

*balanini* (Rudow, 1883, *Ephialtes*)

*multipicta* (Kiss, 1924, *Pimpla*)

*sanguinolenta* (Kiss, 1924, *Pimpla*)

*rufiventris* Hellén, 1949

*amoena* (Haupt, 1954, *Pimpla*) preocc.

*orbitalis* (Haupt, 1954, *Pimpla*) preocc.

***ornata*** (Gravenhorst, 1829, *Pimpla*) E

*soror* (Ratzeburg, 1848, *Polysphincta*)

*arachnicida* Förster, 1888 Horstmann (2000*b*)

*concors* (Kriechbaumer, 1894, *Pimpla*)

*semivaria* (Kriechbaumer, 1894, *Pimpla*)

*tricolor* (Kriechbaumer, 1894, *Pimpla*) preocc.

*kriechbaumeri* (Dalla Torre, 1901, *Pimpla*)

***ovivora*** (Boheman, 1821, *Pimpla*) E S W I M

*armillatoria* (Thunberg, 1824, *Ichneumon*)

*vexatoria* (Thunberg, 1824, *Ichneumon*)

*angens* (Gravenhorst, 1829, *Pimpla*)

*parallela* (Thomson, 1877, *Pimpla*)

*rufipleura* (Bignell, 1899, *Pimpla*)

*albipes* (Brischke, 1891, *Pimpla*) preocc.

*contraria* Förster, 1888 Horstmann (2000*b*)

*evacuans* Förster, 1888 Horstmann (2000*b*)

*brischkei* (Dalla Torre, 1901, *Pimpla*)

*obscurata* (Ulbricht, 1910, *Pimpla*) preocc., unavailable

*rugulosa* (Morley, 1914, *Apechtis*)

*obscurascens* (Strand, 1918, *Pimpla*)

*simulans* (Hensch, 1929, *Pimpla*)

***variabilis*** (Holmgren, 1856, *Pimpla*) E W I

*abdominalis* (Brullé, 1846, *Pimpla*) preocc.

*epeirae* (Bignell, 1893, *Pimpla*)

*hibernica* (Morley, 1908, *Pimpla*)

*ruficoxa* (Kokujev, 1913, *Pimpla*)

*inornata* (Ulbricht, 1926, *Pimpla*)

***ZAGLYPTUS*** Förster, 1869

***multicolor*** (Gravenhorst, 1829, *Polysphincta*) E

*fairmairii* (Laboulbene, 1858, *Pimpla*)

*ephippium* (Rudow, 1883, *Pimpla*) preocc.

*moldavicus* (Costantineanu, 1929, *Pimpla*)

*rufus* Aubert, 1959 preocc.

***varipes*** (Gravenhorst, 1829, *Polysphincta*) E S W I

*tricingulatus* (Gravenhorst, 1829, *Schizopyga*)

*cingulatus* (Kriechbaumer, 1894, *Pimpla*) preocc.

*variipes* Dalla Torre, 1901 preocc.

*rufithorax* (Habermehl, 1917, *Polysphincta*)

*silbernageli* (Kiss, 1926, *Polysphincta*)

***ZATYPOTA*** Förster, 1869

*POLYSPHINCTOPSIS* Habermehl, 1917

*LYCORINOPSIS* Haupt, 1954

***albicoxa*** (Walker, 1874, *Glypta*) E added by Hudson (1985)

*colorata* (Rudow, 1883, *Pimpla*)

*eximia* (Schmiedeknecht, 1907, *Polysphincta*)

*nigriventris* (Habermehl, 1917, *Polysphinctopsis*)

***bohemani*** (Holmgren, 1860, *Polysphincta*) E S W M

***discolor*** (Holmgren, 1860, *Polysphincta*) E S I

O’Connor (2004*b*)

*thoracica* (Brischke, 1864, *Polysphincta*)

***percontatoria*** (Müller, 1776, *Ichneumon*) E S W

*phoenicea* (Haliday, 1839, *Pimpla*)

*gracilis* (Holmgren, 1860, *Polysphincta*) Horstmann (2000*b*)

*scutellaris* (Holmgren, 1860, *Polysphincta*)

*pulchrator* (Thomson, 1877, *Polysphincta*)

*pulchratrix* (Schulz, 1906, *Polysphincta*)

*decorata* (Haupt, 1954, *Lycorinopsis*)

*rhombifer* (Haupt, 1954, *Lycorinopsis*)

species excluded from the British and Irish list by Fitton *et al.* (1988)

[***anomala*** (Holmgren, 1860, *Polysphincta*)[[455]](#footnote-456)

*minor* (Kolarov, 1982, *Sinarachna*) Zwakhals (2006)]

Tribe PIMPLINI Wesmael, 1845

THERONIINI Cushman & Rohwer, 1920

***APECHTHIS*** Förster, 1869

*EPHIALTES* Schrank, 1802 nom. ob.

*APECHTIS* Thomson, 1889

*PARAPECHTHIS* Blanchard, 1936

*TAIWATHERONIA* Sonan, 1936

***compunctor*** (Linnaeus, 1758, *Ichneumon*) E W

*brassicariae* (Poda, 1761, *Ichneumon*)

*cunctator* (Scopoli, 1763, *Ichneumon*)

*annulosa* (Gmelin, 1790, *Ichneumon*)

*cylindrica* (Gmelin, 1790, *Ichneumon*) preocc.

*melanoxantha* (Gmelin, 1790, *Ichneumon*)

*imminuitor* (Christ, 1791, *Ichneumon*) Horstmann (2000*b*)

*vigilans* (Christ, 1791, *Ichneumon*) Horstmann (2000*b*)

*varicornis* (Fabricius, 1793, *Ichneumon*)

*conjunctor* (Panzer, 1804, *Ichneumon*)

*varicator* (Thunberg, 1824, *Ichneumon*)

*lativentris* (Rudow, 1881, *Pimpla*)

*rufipes* (Rudow, 1883, *Pimpla*) preocc.

***quadridentata*** (Thomson, 1887, *Pimpla*) E S W I

*resinator* misident.

***rufata*** (Gmelin, 1790, *Ichneumon*) E S W I

*flavonotata* (Holmgren, 1860, *Pimpla*)

*rufithorax* (Strobl, 1902, *Pimpla*)

*pectoralis* (Ulbricht, 1909, *Pimpla*) unavailable

***ITOPLECTIS*** Förster, 1869

*NESOPIMPLA* Ashmead, 1906

*EXERISTESOIDES* Uchida, 1928

***alternans*** (Gravenhorst, 1829, *Pimpla*) E S W I

*examinanda* (Ratzeburg, 1852, *Pimpla*)

*tricolor* (Ratzeburg, 1852, *Pimpla*) preocc.

*tricincta* (Thomson, 1877, *Pimpla*) preocc.

*spiracularis* (Morley, 1908, *Pimpla*)

*ruficoxis* (Ulbricht, 1916, *Pimpla*) unavailable

***aterrima*** Jussila, 1965 E S W I added by Fitton *et al.* (1988)

*kolthoffi* misident.

*enslini* (Ulbricht, 1916, *Pimpla*) preocc., unavailable

*ultimator* Aubert, 1966

***clavicornis*** (Thomson, 1889, *Pimpla*) E S I

*curticauda* misident.

***enslini*** (Ulbricht, 1911, *Pimpla*) E S added by Fitton *et al.* (1988)

*insignis* misident. Shaw (2006*b*)

*ignalinoensis* (Strand, 1918, *Pimpla*)

*griseanae* Perkins, 1957

***maculator*** (Fabricius, 1775, *Ichneumon*) E S W I M

*arlequinata* (Geoffroy, 1785, *Ichneumon*)

*plaesseus* (Geoffroy, 1785, *Ichneumon*)

*scanica* (Villers, 1789, *Ichneumon*) preocc.

*laetatoria* (Thunberg, 1824, *Ichneumon*)

*vincta* (Vollenhoven, 1873, *Pimpla*)

*maculatrix* (Schulz, 1906, *Pimpla*)

***melanocephala*** (Gravenhorst, 1829, *Pimpla*) E W

*ephippium* (Brullé, 1846, *Pimpla*)

*bicolor* (Boie, 1855, *Pimpla*) preocc.

*ragusae* (De Stefani, 1885, *Pimpla*)

*cleopatra* (Schmiedeknecht, 1897, *Pimpla*)

*burtoni* (Morley, 1946, *Pimpla*)

***viduata*** (Gravenhorst, 1829, *Pimpla*) E added by Shaw (2006*b*)

*atrocoxalis* (Cresson, 1870, *Pimpla*)

*ovalis* (Thomson, 1877, *Pimpla*)

*meridionalis* (Kriechbaumer, 1887, *Pimpla*)

*annulata* (Ulbricht, 1911, *Pimpla*) unavailable

***PIMPLA*** Fabricius, 1804

*COCCYGOMIMUS* Saussure, 1892

*HABROPIMPLA* Cameron, 1900

*LISSOTHERONIA* Cameron, 1905

*PHYTODIAETOIDES* Morley, 1913

*PIMPLIDEA* Viereck, 1914

*COELOPIMPLA* Brèthes, 1916

*DIHYBOPLAX* Enderlein, 1919

*LIOTHERONIA* Enderlein, 1919

*NEOGABUNIA* Brèthes, 1927

*OPODACTYLA* Seyrig, 1932

*OXYPIMPLA* Noskiewicz & Chudoba, 1951

*JAMAICAPIMPLA* Mason, 1975

***aethiops*** Curtis, 1828 E

*aterrima* Gravenhorst, 1829

*parnarae* Viereck, 1912

***arctica*** Zetterstedt, 1838 S

*heraclii* Boie, 1855

*coxator* Ruthe, 1859

*heraclei* Dalla Torre, 1901

*coxatrix* Schulz, 1906

***contemplator*** (Müller, 1776, *Ichneumon*) E S W I M

*geniculata* (Geoffroy, 1785, *Ichneumon*)

*rufistigma* Morley, 1908

*rufitibia* Morley, 1908

***flavicoxis*** Thomson, 1877[[456]](#footnote-457) E S W I M

***insignatoria*** (Gravenhorst, 1807, *Cryptus*)[[457]](#footnote-458) E S W I M

added by Horstmann (2000*b*)

*mixta* Ratzeburg, 1848

*coxalis* Habermehl, 1917 (*turionellae* f.) Horstmann (2000*b*)

*scutellaris* Habermehl, 1917 (*turionellae* f.) preocc.

Horstmann (2000*b*)

*conmixta* Kiss, 1929 (*turionellae* var.) Horstmann (2000*b*)

***melanacrias*** Perkins, 1941 E S W I M

*geniculata* Hensch, 1929 preocc., invalid

***rufipes*** (Miller, 1759, *Ichneumon*) E S W I M

*hypochondriaca* (Retzius, 1783, *Ichneumon*)

Horstmann (1999*a*)

*compunctor* (Geoffroy, 1785, *Ichneumon*) preocc.

*inguinalis* (Geoffroy, 1785, *Ichneumon*)

*instigator* (Fabricius, 1793, *Ichneumon*) preocc.

*intermedia* Holmgren, 1860

*aegyptiaca* Schmiedeknecht, 1897

*instigatrix* Schulz, 1906

*scutellaris* Ulbricht, 1909 preocc., unavailable

*sibirica* Meyer, 1926

***sodalis*** Ruthe, 1859 S

*cheloniae* Giraud, 1869 Horstmann (2001*c*)

*nordenskioldii* Holmgren, 1872

*longiceps* Thomson, 1877

***spuria*** Gravenhorst, 1829 E S W I M

*bilineata* Brullé, 1846

*strigipleuris* Thomson, 1877 Shaw (2006*a*)

*dubitata* Pérez, 1895

*nilotica* Schmiedeknecht, 1914

*turionelloides* Aubert, 1959

***turionellae*** (Linnaeus, 1758, *Ichneumon*) E S W I

*variegata* (Schrank, 1785, *Ichneumon*)

*leucogonos* (Gmelin, 1790, *Ichneumon*)

*rufescens* (Gmelin, 1790, *Ichneumon*) preocc.

*examinator* (Fabricius, 1804, *Cryptus*)

*cingulator* (Thunberg, 1824, *Ichneumon*)

*turionator* (Thunberg, 1824, *Ichneumon*)

*opacellata* Desvignes, 1868

*examinatrix* Schulz, 1906

*pubescens* Hellén, 1915

*padellae* Torka, 1918

*rufoannula* Schmiedeknecht, 1934

*freyi* Hellén, 1949

*variegata* Constantineanu, 1954 preocc.

***wilchristi*** Fitton, Shaw & Gauld, 1988[[458]](#footnote-459) E S W M

added by Fitton *et al.* (1988)

doubtfully placed species of *Pimpla*

[***cossivora*** (Curtis, 1826, *Lissonota*) nom. dub.]

***THERONIA*** Holmgren, 1859

*PSEUDACOENITES* Kriechbaumer, 1892

*POECILOPIMPLA* Cameron, 1903

*ERYTHROTHERONIA* Cameron, 1905

*ORIENTOTHERONIA* Morley, 1913

***atalantae*** (Poda, 1761, *Ichneumon*) E

*speculator* (Scopoli, 1763, *Ichneumon*)

*acuminator* (Müller, 1776, *Ichneumon*)

*melanops* (Schrank, 1781, *Ichneumon*)

*nigroculus* (Schrank, 1781, *Ichneumon*)

*quadripunctata* (Schrank, 1781, *Ichneumon*)

*vincta* (Schrank, 1781, *Ichneumon*)

*scutellata* (Geoffroy, 1785, *Ichneumon*)

*albiscutata* (Gmelin, 1790, *Ichneumon*)

*crassipes* (Rossi, 1790, *Ichneumon*) preocc.

*incisa* (Gmelin, 1790, *Ichneumon*)

*superba* (Christ, 1791, *Ichneumon*) preocc.

*vulpes* (Christ, 1791, *Ichneumon*)

*varia* (Olivier, 1792, *Ichneumon*) preocc.

*flavicans* (Fabricius, 1793, *Ichneumon*)

*varia* (Fabricius, 1793, *Ichneumon*) preocc.

*variatoria* (Fabricius, 1804, *Cryptus*)

*colonator* (Thunberg, 1824, *Ichneumon*)

*femoralis* Benoit, 1953

Subfamily POEMENIINAE Narayanan & Lal, 1953[[459]](#footnote-460)

Tribe POEMENIINI Narayanan & Lal, 1953

***DEUTEROXORIDES*** Viereck, 1914

***elevator*** (Panzer, 1799, *Ichneumon*) E I

*albitarsus* (Gravenhorst, 1829, *Xorides*)

*nigricornis* Clément, 1938

*nigritarsus* Clément, 1938

***PODOSCHISTUS*** Townes, 1957

***scutellaris*** (Desvignes, 1856, *Xorides*) E W

*wahlbergi* (Holmgren, 1860, *Xorides*)

*erosus* (Tschek, 1869, *Xorides*)

***POEMENIA*** Holmgren, 1859

*OPHIODES* Hartig, 1847 preocc.

*CALLICLISIS* Förster, 1869

*PHTHINODES* Tschek, 1869

*LISSONOTOPSIS* Habermehl, 1917

***collaris*** (Haupt, 1917, *Calliclisis*) E added by Fitton *et al.* (1988)

*picta* (Haupt, 1938, *Calliclisis*)

***hectica*** (Gravenhorst, 1829, *Ephialtes*) E S I

*montana* (Hartig, 1847, *Ophiodes*)

*tipularia* Holmgren, 1860

***notata*** Holmgren, 1859 E added by Fitton *et al.* (1988)

*novakii* Strobl, 1902

*rufa* (Habermehl, 1918, *Lissonotopsis*)

*rufa* (Habermehl, 1918, *Xorides*) preocc.

*intermedia* Constantineanu & Constantineanu, 1969

*moldavica* Constantineanu & Constantineanu, 1969

Tribe PSEUDORHYSSINI Wahl & Gauld, 1998

***PSEUDORHYSSA*** Merrill, 1915

***alpestris*** (Holmgren, 1860, *Rhyssa*) E W Formstone (1999)

*ruficoxis* (Kriechbaumer, 1887, *Rhyssa*)

*hungarica* (Mocsáry, 1905, *Rhyssa*)

Subfamily RHYSSINAE Morley, 1913[[460]](#footnote-461)

***RHYSSA*** Gravenhorst, 1829

*CRYPTOCENTRUM* Kirby, 1837

*PARARHYSSA* Walsh, 1873

***persuasoria*** (Linnaeus, 1758, *Ichneumon*) E S W I M

Hayes (1982), Cowin & Williamson (1940)

*marginalis* Brullé, 1846

*lineolata* Kriechbaumer, 1887 preocc.

*gloriosa* Rudow, 1889

***RHYSSELLA*** Rohwer, 1920

***approximator*** (Fabricius, 1793, *Ichneumon*) E S

*curvipes* (Gravenhorst, 1829, *Rhyssa*)

*rugicollis* (Zetterstedt, 1838, *Tryphon*)

*bellator* (Schiødte, 1839, *Rhyssa*) Horstmann (2004*b*)

*approximatrix* (Schulz, 1906, *Rhyssa*)

*silbernageli* (Kiss, 1926, *Rhyssa*)

Subfamily STILBOPINAE Townes & Townes, 1949

***PANTELES*** Förster, 1869[[461]](#footnote-462)

*BRACHYPIMPLA* misident.

***schuetzeanus*** (Roman, 1925, *Brachypimpla*) E S I

*schnetzeanus* misspelling[[462]](#footnote-463)

***STILBOPS*** Förster, 1869[[463]](#footnote-464)

*APHANOROPTRUM* Förster, 1869

*APHANOROPTRA* Thomson, 1877

*APHANORRHOPTRUM* Dalla Torre, 1901

*ERITRACHYNUS* Schmiedeknecht, 1913

***asper*** (Schmiedeknecht, 1913, *Eritrachynus*) E S

added by Fitton (1984)

***limneriaeformis*** (Schmiedeknecht, 1888, *Pimpla*) S I

***ruficornis*** (Gravenhorst, 1829, *Lissonota*) E

*abdominalis* (Gravenhorst, 1829, *Pimpla*)

*nematorum* (Rudow, 1881, *Pimpla*)

*longiceps* (Strobl, 1903, *Polyblastus*)

***vetula*** (Gravenhorst, 1829, *Pimpla*) E S W I

*chrysostomus* (Gravenhorst, 1829, *Phytodietus*)

*pallipes* (Gravenhorst, 1829, *Lissonota*)

*pallidipes* (Marshall, 1872, *Lissonota*)

*varicauda* (Capron, 1888, *Pimpla*)

Subfamily TERSILOCHINAE Schmiedeknecht, 1910[[464]](#footnote-465)

PHRUDINAE Townes & Townes, 1949

***ALLOPHROIDES*** Horstmann, 1971

***boops*** (Gravenhorst, 1829, *Porizon*)

*italicus* (Gravenhorst, 1829, *Porizon*)

*breviventris* (Hellén, 1958, *Allophrys*)

***ANEUCLIS*** Förster, 1869

***melanaria*** (Holmgren, 1860, *Thersilochus*) E I

*diversa* (Szépligeti, 1899, *Isurgus*)

*petiolaris* (Szépligeti, 1899, *Isurgus*)

***ASTRENIS*** Förster, 1869[[465]](#footnote-466)

*MENGERSENIA* Schmiedeknecht, 1907

*HAMBERGIELLA* Roman, 1909

***brunneofacies*** Vikberg, 2000 E S added by Vikberg & Koponen (2000)

***nigrifacies*** Vikberg, 2000 E S added by Vikberg & Koponen (2000)

***paradoxus*** (Schmiedeknecht, 1907, *Mengersenia*) E

added by Gauld & Fitton (1980)

***sinuatus*** (Roman, 1909, *Hambergiella*) E S I

***BARYCNEMIS*** Förster, 1869

*LEPTOPYGUS* Förster, 1869

*CRATOPHION* Thomson, 1889

*CYRTOPHION* Thomson, 1889

***agilis*** (Holmgren, 1860, *Porizon*) E S NHM, NMS, det. Khalaim & Horstmann, added here

***angustipennis*** (Holmgren, 1860, *Porizon*) E S I

added by Horstmann (1981*a*)

***bellator*** (Müller, 1776, *Ichneumon*) E S

*laeviceps* (Thomson, 1889, *Porizon*)

*leviceps* Dalla Torre, 1901 preocc.

*pfankuchi* Lange, 1911

***blediator*** (Aubert, 1970, *Leptopygus*) E W added by Wyatt & Foster (1989)

***confusa*** Horstmann, 1981 S NHM, NMS, det. Broad & Horstmann, added here

***dissimilis*** (Gravenhorst, 1829, *Porizon*) E

*erythrura* (Strobl, 1904, *Porizon*)

***exhaustator*** (Fabricius, 1798, *Ichneumon*) E

*obtusator* (Panzer, 1809, *Ophion*)

***gravipes*** (Gravenhorst, 1829, *Porizon*) E S I

*hostilis* (Gravenhorst, 1829, *Porizon*)

***guttulator*** (Thunberg, 1824, *Ichneumon*)

*caudatula* (Thomson, 1889, *Porizon*)

***harpura*** (Schrank, 1802, *Ichneumon*) E S W I

*bedeguaris* (Panzer, 1809, *Ophion*)

***punctifrons*** Horstmann, 1981 E I added by Horstmann (1981*a*)

***DIAPARSIS*** Förster, 1869

subgenus ***DIAPARSIS*** Förster, 1869

*DIAPARSUS* Thomson, 1889

***carinifer*** (Thomson, 1889, *Thersilochus*) E S W I

*carinata* (Bridgman, 1889, *Thersilochus*)

*vernalis* (Szépligeti, 1899, *Thersilochus*)

***multiplicator*** Aubert, 1969 E

***nutritor*** (Fabricius, 1804, *Ophion*)

*gemina* (Holmgren, 1860, *Thersilochus*)

*genalis* (Thomson, 1889, *Thersilochus*)

*rugosa* (Szépligeti, 1905, *Temelucha*)

*nutritrix* Schulz, 1906

***punctipleuris*** Horstmann, 1981 E NHM, det. Khalaim, added here

subgenus ***ISCHNOBATIS*** Förster, 1869

***stramineipes*** (Brischke, 1880, *Thersilochus*) E I

*rufiventris* (Brischke, 1880, *Thersilochus*)

*flavicornis* (Thomson, 1889, *Thersilochus*)

*petiolata* (Szépligeti, 1899, *Thersilochus*)

subgenus ***NANODIAPARSIS*** Horstmann, 1971

***aperta*** (Thomson, 1889, *Thersilochus*) E NMS, NHM, det. Horstmann and Khalaim, added here

***frontella*** (Holmgren, 1860, *Thersilochus*) E

subgenus ***PSEUDANEUCLIS*** Horstmann, 1971

***rara*** (Horstmann, 1971, *Pseudaneuclis*) E NHM, det. Horstmann, added here

***EPISTATHMUS*** Förster, 1869

***crassicornis*** Horstmann, 1971 E S I added by Horstmann (1981*a*)

***GELANES*** Horstmann, 1981

***fusculus*** (Holmgren, 1860, *Thersilochus*) E S NMS, det. Horstmann, NHM, det. Khalaim, added here

***simillimus*** Horstmann, 1981 E S I added by Horstmann (1981*a*)

***HETEROCOLA*** Förster, 1869

subgenus ***HETEROCOLOIDES*** Horstmann, 1971

***linguaria*** (Haliday, 1839, *Porizon*) E I[[466]](#footnote-467)

*punctulata* (Szépligeti, 1899, *Ischnobatis*)

***PHRADIS*** Förster, 1869

*EUTOMUS* Förster, 1869

*ISURGUS* Förster, 1869

***brevis*** (Brischke, 1880, *Thersilochus*) E I added by Horstmann (1981*a*)

*temporalis* (Thomson, 1889, *Thersilochus*)

*styriacus* (Strobl, 1904, *Thersilochus*)

***interstitialis*** (Thomson, 1889, *Thersilochus*) E S W I

*brachygaster* (Szépligeti, 1899, *Isurgus*)

***minutus*** (Bridgman, 1889, *Thersilochus*) E W I

***monticola*** Szépligeti, 1899 E NHM, det. Ely, added here

***morionellus*** (Holmgren, 1860, *Thersilochus*) E I

*lanceolatus* (Szépligeti, 1899, *Isurgus*)

*oudesmani* (Smits van Burgst, 1913, *Isurgus*)

***nigritulus*** (Gravenhorst, 1829, *Porizon*) E

*albipennis* (Szépligeti, 1899, *Isurgus*)

***polonicus*** Horstmann, 1981 E S NHM, NMS, added here

***rufiventris*** Horstmann, 1981 E NHM, det. Ely, added here

***terebrator*** Horstmann, 1981 E NHM, det. Ely, added here

***thyridialis*** Horstmann, 1981 E I NHM, det. Ely, added here

***PHRUDUS*** Förster, 1869

*PHRUDUS* Bridgman, 1886 preocc.

*KTENOSTILPNUS* Strobl, 1901

*VENDOLUS* Roman, 1914

***badensis*** Hilpert, 1987 E added by Shaw (1991)

***defectus*** Stelfox, 1966 E S I M

***monilicornis*** Bridgman, 1886 E S W I

*aequearticulatus* (Strobl, 1901, *Ktenostilpnus*)

*stilpninus* (Roman, 1914, *Vendolus*)

***PROBLES*** Förster, 1869

subgenus ***EUPORIZON*** Horstmann, 1971

***brevicauda*** Horstmann, 1981 E added by Horstmann (1981*a*)

***exilis*** (Holmgren, 1860, *Thersilochus*) S NMS, det. Horstmann, added here

***gilvipes*** (Gravenhorst, 1829, *Porizon*) E

*pallipes* (Holmgren, 1860, *Thersilochus*)

*orchesiae* (Morley, 1915, *Thersilochus*)

***longicaudator*** Aubert, 1972 E I added by Horstmann (1981*a*)

***marginatus*** (Bridgman, 1886, *Thersilochus*) E I

***montanus*** Horstmann, 1971 S I added by Horstmann (1981*a*)

***nigriventris*** Horstmann, 1971 I added by Horstmann (1981*a*)

***rufipes*** (Holmgren, 1860, *Thersilochus*) E I

*flavigaster* (Szépligeti, 1899, *Ischnobatis*)

***truncorum*** (Holmgren, 1860, *Thersilochus*) E I

subgenus ***MICRODIAPARSIS*** Horstmann, 1971

***caudiculatus*** Khalaim, 2007 E added by Khalaim (2007)

***microcephalus*** (Gravenhorst, 1829, *Porizon*) E S I

*quercetorum* (Szépligeti, 1899, *Thersilochus*)

*ruficoxis* (Seyrig, 1927, *Diaparsis*)

***neoversutus*** (Horstmann, 1967, *Diaparsis*) E I

*parviceps* (Szépligeti, 1899, *Thersilochus*) preocc.

***versutus*** (Holmgren, 1860, *Thersilochus*) E

*parviceps* (Thomson, 1899, *Thersilochus*)

subgenus ***PROBLES*** Förster, 1869

***erythrostomus*** (Gravenhorst, 1829, *Porizon*) E S I

*minator* (Gravenhorst, 1829, *Porizon*)

*melanarius* Szépligeti, 1899

***flavipes*** (Szépligeti, 1899, *Ischnobatis*) E I added by Horstmann (1981*a*)

subgenus ***Rugodiaparsis*** Horstmann, 1971

***crassipes*** (Thomson, 1889, *Thersilochus*) E S W

NHM, NMS, det. Horstmann, added here

***PYGMAEOLUS*** Hellén, 1958

***nitidus*** (Bridgman, 1889, *Thersilochus*) E S I

***SATHROPTERUS*** Förster, 1869

***pumilus*** (Holmgren, 1860, *Thersilochus*) E I added by Horstmann (1981*a*)

***SPINOLOCHUS*** Horstmann, 1971

***laevifrons*** (Holmgren, 1860, *Thersilochus*) E S I

added by Horstmann (1981*a*)

*levifrons* (Dalla Torre, 1901, *Cyrtophion*) preocc.

***TERSILOCHUS*** Holmgren, 1859

subgenus ***GONOLOCHUS*** Förster, 1869

***caudatus*** (Holmgren, 1860, *Thersilochus*) E I

*pratensis* (Szépligeti, 1899, *Thersilochus*)

*salinus* (Kiss, 1924, *Temelucha*)

***rugulosus*** Horstmann, 1981 E NHM, det. Khalaim, added here

subgenus ***PECTINOLOCHUS*** Aubert, 1960

*POLEMOLOCHUS* Aubert, 1964

***intermedius*** Horstmann, 1981 E NMS, det. Horstmann, added here

***lapponicus*** Hellén, 1958 E W I added by Horstmann (1981*a*)

***spiracularis*** Horstmann, 1971 E NHM, det. Broad and Khalaim, added here

***striola*** (Thomson, 1889, *Thersilochus*) E S I added by Horstmann (1981*a*)

*unguiculator* (Aubert, 1960, *Thersilochus*)

***terebrator*** (Horstmann, 1971, *Pectinolochus*) E I

added by Horstmann (1981*a*)

subgenus ***TERSILOCHUS*** Holmgren, 1859

*THERSILOCHUS* misspelling

***cognatus*** (Holmgren, 1860, *Thersilochus*) E W I

*jocator* Holmgren, 1859 unavailable Horstmann (2005*b*)

***curvator*** Horstmann, 1981 E I added by Horstmann (1981*a*)

*saltator* misident.

***heterocerus*** (Thomson, 1889, *Thersilochus*) E I

*stanionyteus* Jonaitis, 1974

*vicinus* Jonaitis, 1974

***liopleuris*** (Thomson, 1889, *Thersilochus*) E I

***longicaudatus*** Horstmann, 1971 E I added by Horstmann (1981*a*)

***longicornis*** (Thomson, 1889, *Thersilochus*) E S I

added by Horstmann (1981*a*)

***microgaster*** (Szépligeti, 1899, *Isurgus*) E added by Barari *et al.* (2005)

***nitidipleuris*** Horstmann, 1971 E I added by Horstmann (1981*a*)

***obliquus*** (Thomson, 1889, *Thersilochus*) E I added by Horstmann (1981*a*)

***obscurator*** (Aubert, 1959, *Thersilochus*) E S I

added by Horstmann (1981*a*)

***ruberi*** Horstmann, 1981 I added by Horstmann (1981*a*)

***triangularis*** (Gravenhorst, 1807, *Ophion*) E

*minutus* (Szépligeti, 1899, *Isurgus*)

***tripartitus*** (Brischke, 1880, *Thersilochus*) E I added by Horstmann (1981*a*)

*melanogaster* (Thomson, 1889, *Thersilochus*)

*nigricans* (Szépligeti, 1899, *Thersilochus*)

Subfamily TRYPHONINAE Shuckard, 1840

Tribe ECLYTINI Townes & Townes, 1945[[467]](#footnote-468)

***ECLYTUS*** Holmgren, 1857[[468]](#footnote-469)

subgenus ***ANOPLECTES*** Kriechbaumer, 1896

***multicolor*** (Kriechbaumer, 1896, *Anoplectes*) E S I

added by Fitton & Ficken (1990)

*praeclerus* Schmiedeknecht, 1912

subgenus ***ECLYTUS*** Holmgren, 1857

*ZAPEDIAS* Förster, 1869 Bennett (2015)

***difficilis*** Kasparyan, 1977 S added by Shaw & Kasparyan (2005)

***egregius*** Kasparyan, 1977 S added by Shaw & Kasparyan (2005)

***exornatus*** (Gravenhorst, 1829, *Mesoleptus*) E S W I

***haustatorius*** Kasparyan, 1977 E S added by Shaw & Kasparyan (2005)

[***ornatus*** Holmgren, 1857 E S[[469]](#footnote-470)]

Tribe IDIOGRAMMATINI Cushman, 1942[[470]](#footnote-471)

***IDIOGRAMMA*** Förster, 1869

*MACROCHASMUS* Thomson, 1888

***euryops*** Förster, 1869 S

Tribe OEDEMOPSINI Woldstedt, 1877[[471]](#footnote-472)

THYMARIDINI Schmiedeknecht, 1911

***CLADEUTES*** Townes, 1969

***discedens*** (Woldstedt, 1874, *Perilissus*) E I added by Fitton & Ficken (1990)

*haematothorax* (Strobl, 1903, *Eclytus*)

*lepidus* Townes, 1969

***HERCUS*** Townes, 1969

***fontinalis*** (Holmgren, 1857, *Eclytus*) E S W I

*frontalis* (Zetterstedt, 1838, *Bassus*) nom. oblitum

***NELIOPISTHUS*** Thomson, 1883

***elegans*** (Ruthe, 1855, *Phytodiaetus*) E

*ops* (Morley, 1908, *Oedematopsis*)

***OEDEMOPSIS*** Tschek, 1869

*CAMPOTHREPTUS* Förster, 1869

*HYBOPHANES* Förster, 1869

*OEDEMATOPSIS* Morley, 1908

*ODEMOPSIS* misspelling

***scabricula*** (Gravenhorst, 1829, *Tryphon*) E S I M

O'Connor (2003)

*dorsata* (Zetterstedt, 1838, *Bassus*)

*pulchra* (Zetterstedt, 1839, *Bassus*)

*rogenhoferi* Tschek, 1869

*limbata* Thomson, 1883

***THYMARIS*** Förster, 1869

*THYMARUS* Thomson, 1883

***niger*** (Taschenberg, 1865, *Hemiteles*) E S

*fenestralis* Morley, 1908

*modestus* Schmiedeknecht, 1912

*simplicicornis* Kiss, 1924 Horstmann (1998*b*)

*tristrigator* Aubert, 1960

***srikem*** Fitton & Ficken, 1990 E W I added by Fitton & Ficken (1990)

***tener*** (Gravenhorst, 1829, *Mesoleptus*)[[472]](#footnote-473) E S

*contaminatus* (Gravenhorst, 1829, *Hemiteles*)

*marchicus* (Hartig, 1838, *Ischnoceros*)

*pulchricornis* Brischke, 1880

*compressus* (Thomson, 1883, *Thymarus*)

Tribe PHYTODIETINI Hellén, 1915

NETELIINI Townes, 1938

***NETELIA*** Gray, 1860[[473]](#footnote-474)

*PANISCUS* misident.

subgenus ***Bessobates*** Townes, Townes & Gupta, 1961

***cristata*** (Thomson, 1888, *Parabatus*) E S W I M

*frankii* (Brauns, 1889, *Parabatus*) [[474]](#footnote-475)

***latungula*** (Thomson, 1888, *Parabatus*) E S I

***pallescens*** (Schmiedeknecht, 1910, *Parabatus*) E S I

added by Broad & Shaw (in prep.)

***virgata*** (Geoffroy, 1785, *Ichneumon*) E S W I M

**sp. R** Broad & Shaw, in prep. S added by Broad & Shaw (in prep.)

subgenus ***NETELIA*** Gray, 1860[[475]](#footnote-476)

*BUCHECKERIUS* Schulz, 1906

***dilatata*** (Thomson, 1888, *Paniscus*) E

*brachycera* (Thomson, 1888, *Paniscus*)

*capito* (Kokujev, 1889, *Paniscus*)

*genalis* (Kokujev, 1889, *Paniscus*)

*schirjajewi* (Kokujev, 1889, *Paniscus*)

*sibiricola* (Kokujev, 1889, *Paniscus*)

*nigricans* (Kriechbaumer, 1898, *Paniscus*)

*nigridorsum* (Meyer, 1929, *Paniscus*)

***fuscicarpus*** (Kokujev, 1899, *Paniscus*) E W added by Broad & Shaw (in prep.)

*desertus* (Kokujev, 1915, *Paniscus*)

*maltractus* (Roman, 1938, *Paniscus*)

*ambiguator* Aubert, 1969

***fuscicornis*** (Holmgren, 1860, *Paniscus*) E S

*gracilipes* (Thomson, 1888, *Paniscus*)

*intersita* (Kokujev, 1889, *Paniscus*)

*montana* (Kokujev, 1889, *Paniscus*)

*praetermissa* (Kokujev, 1889, *Paniscus*)

***infractor*** Delrio, 1971 E S W I added by Broad & Shaw (in prep.)

***melanura*** (Thomson, 1888, *Paniscus*) E S

***ocellaris*** (Thomson, 1888, *Paniscus*) E

***opacula*** (Thomson, 1888, *Paniscus*) E

***testacea*** (Gravenhorst, 1829, *Paniscus*)[[476]](#footnote-477) E S

added by Horstmann (1992*b*)

***vinulae*** (Scopoli, 1763, *Ichneumon*) E S

*inquinata* (Gravenhorst, 1829, *Paniscus*) Horstmann (1998*b*)

*vinulae* (Stephens, 1829, *Ophion*) preocc. Horstmann (2000*b*)

*cephalotes* (Holmgren, 1860, *Paniscus*)

**sp. W** Broad & Shaw, in prep. E W added by Broad & Shaw (in prep.)

subgenus ***PARABATES*** Förster, 1869

*PARABATUS* Thomson, 1888

***nigricarpa*** (Thomson, 1888, *Parabatus*) E S

*semifusca* (Strobl, 1904, *Parabatus*)

subgenus ***PAROPHELTES*** Cameron, 1907

**sp. C** Broad & Shaw, in prep. E added by Broad & Shaw (in prep.)

***inedita*** (Kokujev, 1899, *Paniscus*) E S W I[[477]](#footnote-478) added by Shaw (2001)

*longipes* misident.

*ornata* misident.

*thomsonii* misident.

***millieratae*** (Kriechbaumer, 1897, *Parabatus*) E

added by Broad & Shaw (in prep.)

***ornata*** (Vollenhoven, 1873, *Paniscus*) E[[478]](#footnote-479)

*longipes* (Brauns, 1889, *Paniscus*)[[479]](#footnote-480)

*catagrapha* (Kokujev, 1915, *Paniscus*)

*ignobilis* (Kokujev, 1915, *Paniscus*)

*versicolor* (Kokujev, 1915, *Paniscus*)

*decorator* (Seyrig, 1927, *Paniscus*)

***tarsata*** (Brischke, 1880, *Paniscus*) E S W I

subgenus ***PROSTHODOCIS*** Enderlein, 1912

**sp. A** Broad & Shaw, in prep. E I added by Broad & Shaw (in prep.)

**sp. B** Broad & Shaw, in prep. S I added by Broad & Shaw (in prep.)

***PHYTODIETUS*** Gravenhorst, 1829[[480]](#footnote-481)

subgenus ***Neuchorus*** Uchida, 1931

***elongator*** Aubert, 1963 E added by Kasparyan & Shaw (2008)

*iassiensis* Constantineanu, 1929 invalid

***maculator*** Kasparyan & Shaw, 2008 E added by Kasparyan & Shaw (2008)

Species excluded from the British and Irish list

[***obscurus*** (Ratzeburg, 1852, *Lissonota*)[[481]](#footnote-482)

*rufipes* Holmgren, 1860 Horstmann (1998*b*)

*orbitalis* Ulbricht, 1911 unavailable]

subgenus ***Phytodietus*** Gravenhorst, 1829

*PHYTODIAETUS* Aggasiz, 1846

*PHYTODIAETUS* Morley, 1913 preocc.

***astutus*** Gravenhorst, 1829 E S W

*obscurus* Desvignes, 1856 preocc.

*continuus* Thomson, 1887 Horstmann (1998*b*)

*britannicus* (Habermehl, 1923, *Barytarbes*)

Horstmann (2000*d*)

***basalis*** Kasparyan, 1993 E S added by Kasparyan & Shaw (2008)

***femoralis*** Holmgren, 1860 S added by Kasparyan & Shaw (2008)

***gelitorius*** (Thunberg, 1824, *Ichneumon*) E S W I

*polyzonius* (Thunberg, 1824, *Ichneumon*) preocc.

*coryphaeus* Gravenhorst, 1829

*carinatus* Hellén, 1915

*coxator* (Aubert, 1963, *Lathrolestes*)

***geniculatus*** Thomson, 1877 E S W I

***griseanae*** Kerrich, 1962 E S W

***montanus*** Tolkanitz, 1979 E S W I added by Kasparyan & Shaw (2008)

***ornatus*** Desvignes, 1856 E S W I

*rubricosus* Thomson, 1877

*rufipictus* Brischke, 1880

*pictus* (Habermehl, 1923, *Barytarbes*) Horstmann (2004*c*)

***polyzonias*** (Forster, 1771, *Ichneumon*) E Fitton (1976)

*segmentator* Gravenhorst, 1829

***variegatus*** (Fonscolombe, 1854, *Lissonota*) E S

added by Kasparyan & Shaw (2008)

*albipes* Holmgren, 1856

Tribe SPHINCTINI Förster, 1869

***SPHINCTUS*** Gravenhorst, 1829

***serotinus*** Gravenhorst, 1829 E[[482]](#footnote-483)

Tribe TRYPHONINI Shuckard, 1840[[483]](#footnote-484)

EXENTERINI Förster, 1869 Bennett (2015)[[484]](#footnote-485)

CTENISCINI Thomson, 1883

GRYPOCENTRINI Townes & Townes, 1949

***ACROTOMUS*** Holmgren, 1857

*DELOTOMUS* Förster, 1869

***lucidulus*** (Gravenhorst, 1829, *Tryphon*) E S W

*sexcinctus* (Gravenhorst, 1829, *Tryphon*)

*auriculatus* (Thomson, 1883, *Delotomus*)

***succinctus*** (Gravenhorst, 1829, *Tryphon*) E S W I

***COSMOCONUS*** Förster, 1869

***ceratophorus*** (Thomson, 1888, *Tryphon*) E S

***elongator*** (Fabricius, 1775, *Ichneumon*) E S I M

*elliotti* (Morley, 1911, *Tryphon*)

***meridionator*** Aubert, 1963 E S added by Shaw & Kasparyan (2005)

***nigriventris*** Kasparyan, 1971 E S added by Shaw & Kasparyan (2005)

***CTENISCUS*** Haliday, 1832

*EUDIABORUS* Kerrich, 1952

***maculiventris*** (Ashmead, 1896, *Diaborus*)[[485]](#footnote-486) S

added by Kerrich (1952)

***nigrifrons*** (Thomson, 1883, *Diaborus*) E

***pedatorius*** (Panzer, 1809, *Bassus*) E S I

*sexlituratus* (Gravenhorst, 1829, *Tryphon*)

*filipalpis* (Thomson, 1883, *Diaborus*)

*moravicus* (Gregor, 1937, *Diaborus*)

***scalaris*** (Gravenhorst, 1829, *Tryphon*) E S Rahoo & Luff (1988)

*pallitarsis* (Thomson, 1883, *Diaborus*)

*palliditarsis* (Dalla Torre, 1901, *Diaborus*)

***CTENOCHIRA*** Förster, 1855

*CTENACME* Förster, 1869

*GEMOPHAGA* Förster, 1869

*SCOPIORUS* Förster, 1869

*CTENACMUS* Thomson, 1883

*CTENACMA* Schulz, 1906

*EXOCHOBLASTUS* Schmiedeknecht, 1912

*SCOPIMENUS* Roman, 1937

*COELOPROSOPON* Bauer, 1958

***angulata*** (Thomson, 1883, *Polyblastus*) S M

***angustata*** (Roman, 1909, *Polyblastus*)

***arcuata*** (Holmgren, 1857, *Polyblastus*) E[[486]](#footnote-487)

*antennator* Aubert, 1965

[**?*breviseta*** (Ratzeburg, 1852, *Pimpla*)[[487]](#footnote-488)]

*aberrans* (Ruthe, 1855, *Tryphon*) Horstmann (2002*c*)

**?*gelida*** Kasparyan, 1973[[488]](#footnote-489) S added by Shaw & Kasparyan (2005)

***genalis*** (Thomson, 1883, *Polyblastus*) E added by Kasparyan (1973)[[489]](#footnote-490)

***gilvipes*** (Holmgren, 1857) E S M

*albiventris* (Brischke, 1892, *Polyblastus*)

***grossa*** (Brischke, 1871, *Polyblastus*) E S[[490]](#footnote-491)

*annulicornis* (Giraud, 1872, *Polyblastus*)

***haemosterna*** (Haliday, 1839, *Tryphon*)[[491]](#footnote-492) E S I

Horstmann (2002*c*)

*senilis* (Holmgren, 1857, *Polyblastus*)

*nigripalpis* (Thomson, 1883, *Polyblastus*)

*subrufa* (Bridgman, 1887, *Polysphincta*) Kasparyan & Tolkanitz (2000)

*haematosterna* (Dalla Torre, 1901, *Polyblastus*)

***marginata*** (Holmgren, 1857, *Polyblastus*) E S W M

*fractigena* (Heinrich, 1953, *Scopiorus*)

***meridionator*** Aubert, 1969[[492]](#footnote-493) E added by Shaw & Kasparyan (2005)

***pastoralis*** (Gravenhorst, 1829, *Tryphon*)

*mutabilis* (Holmgren, 1857, *Polyblastus*)

*nitidiventris* (Holmgren, 1857, *Polyblastus*)

***pratensis*** (Gravenhorst, 1829, *Tryphon*) E

***propinqua*** (Gravenhorst, 1829, *Tryphon*) E S I

*obscura* (Stephens, 1835, *Tryphon*) Kasparyan & Tolkanitz (2000)

*caudata* (Holmgren, 1856, *Poyblastus*)

***romani*** (Pfankuch, 1925, *Poyblastus*) E M

*pygobarba* (Roman, 1937, *Scopimenus*)

***rubranator*** Aubert, 1965 S added by Shaw & Kasparyan (2005)

***rufipes*** (Gravenhorst, 1829, *Tryphon*) E S

*anilis* (Holmgren, 1857, *Polyblastus*)

*glabella* (Holmgren, 1857, *Polyblastus*)

*limosa* (Holmgren, 1857, *Polyblastus*)

*mixta* (Holmgren, 1857, *Polyblastus*)

*nigella* (Holmgren, 1857, *Polyblastus*)

*praedator* (Holmgren, 1857, *Polyblastus*)

*holmgreni* (Woldstedt, 1874, *Polyblastus*) preocc.

*woldstedtii* (Dalla Torre, 1901, *Polyblastus*)

***sanguinatoria*** (Ratzeburg, 1852, *Tryphon*) E

***sphaerocephala*** (Gravenhorst, 1829, *Tryphon*) E S M

*bisculpta* (Gravenhorst, 1829, *Tryphon*)

*trisculpta* (Stephens, 1835, *Tryphon*)

*bifasciata* (Zetterstedt, 1838, *Tryphon*)

*trisculpta* (Holmgren, 1856, *Polyblastus*) preocc.

***validicornis*** (Brischke, 1871, *Polyblastus*) E S I

added by Shaw & Kasparyan (2005)

*fusicornis* (Thomson, 1883, *Polyblastus*)

*insculpta* (Habermehl, 1922, *Polyblastus*)

***xanthopyga*** (Holmgren, 1857, *Polyblastus*) E S

*rivalis* (Holmgren, 1857, *Polyblastus*)

***CYCASIS*** Townes, 1965

***rubiginosa*** (Gravenhorst, 1829, *Tryphon*) E S

*insidiator* (Holmgren, 1857, *Acrotomus*)

*morio* (Holmgren, 1857, *Exenterus*)

*binotata* (Thomson, 1883, *Delotomus*)

*parvula* (Thomson, 1883, *Delotomus*)

***DYSPETES*** Förster, 1869

*DYSPETUS* Thomson, 1883

***arrogator*** Heinrich, 1949 E I

*rufatus* Gregor, 1929 unavailable Horstmann (2005*b*)

***luteomarginatus*** Habermehl, 1925[[493]](#footnote-494) E S W I M

NMS, det. Horstmann, NHM, det. Broad, added here

*chrysogaster* (Gmelin, 1790, *Ichneumon*) preocc.

Horstmann (2006*a*)

*praerogator* (Thomson, 1883, *Dyspetus*) unavailable

Horstmann (2005*b*)

*fracticeps* (Townes & Townes, 1950, *Dyspetus*)

Horstmann (2006*a*)

***ERIDOLIUS*** Förster, 1869

*ANISOCTENION* Förster, 1869

***alacer*** (Gravenhorst, 1829, *Tryphon*) E S

*xanthopus* (Holmgren, 1857, *Acrotomus*)

***aurifluus*** (Haliday, 1839, *Tryphon*) E I

*geniculosus* (Schiødte, 1839, *Exenterus*)

*approximatus* (Holmgren, 1857, *Exenterus*)

***basalis*** (Stephens, 1835, *Tryphon*) E S W I M

*connatus* (Holmgren, 1857, *Exenterus*)

*flavilabris* (Holmgren, 1857, *Exenterus*)

*gracilis* (Holmgren, 1857, *Exenterus*)

*hostilis* (Holmgren, 1857, *Exenterus*)

*limbatellus* (Holmgren, 1857, *Exenterus*)

*umbellatarum* (Woldstedt, 1874, *Exenterus*)

*rufofasciatus* (Strobl, 1903, *Polyblastus*) Horstmann (2012*c*)

*minutulus* (Pfankuch, 1907, *Cteniscus*)

***bimaculatus*** (Holmgren, 1856, *Exenterus*) E S W I

*zonellus* (Holmgren, 1857, *Exenterus*)

*alpinus* (Roman, 1909, *Cteniscus*)

***consobrinus*** (Holmgren, 1857, *Exenterus*) I

***curtisii*** (Haliday, 1839, *Tryphon*) E S I

***dorsator*** (Thunberg, 1824, *Ichneumon*) E I

*mitigosus* (Gravenhorst, 1829, *Tryphon*)

*lineola* (Stephens, 1835, *Tryphon*)

*similatorius* (Schiødte, 1839, *Exenterus*)

*limbatus* (Holmgren, 1856, *Exenterus*)

*alpicola* (Holmgren, 1857, *Exenterus*)

*borealis* (Holmgren, 1857, *Exenterus*)

*frigidus* (Holmgren, 1857, *Exenterus*)

*brevigena* (Thomson, 1883, *Cteniscus*)

*punctipes* (Thomson, 1883, *Cteniscus*)

*punctipleuris* (Thomson, 1883, *Cteniscus*)

*signifer* (Thomson, 1883, *Cteniscus*)

*albicollis* (Habermehl, 1925, *Cteniscus*)

***elegans*** (Stephens, 1835, *Tryphon*) E S

*aulicus* (Roman, 1914, *Cteniscus*)

***ermolenkoi*** Kasparyan, 1990 E added by Shaw & Kasparyan (2005)

***flavomaculatus*** (Gravenhorst, 1829, *Tryphon*) E S

*praeustus* (Holmgren, 1857, *Exenterus*)

*pumilus* (Holmgren, 1857, *Exenterus*)

*ustulatus* (Holmgren, 1857, *Exenterus*)

*quadrinotatus* (Thomson, 1883, *Cteniscus*)

*t-nigrum* (Thomson, 1883, *Cteniscus*)

*facialis* (Roman, 1913, *Cteniscus*)

***gnathoxanthus*** (Gravenhorst, 1829, *Tryphon*) E S I

*hachfeldi* (Ulbricht, 1926, *Polyblastus*)

***hofferi*** (Gregor, 1937, *Cteniscus*) E I

***lineiger*** (Thomson, 1883, *Cteniscus*) S added by Shaw & Kasparyan (2005)

*nordstromi* (Kerrich, 1952, *Cteniscus*)

***pachysoma*** (Stephens, 1835, *Tryphon*) E I

*colorator* (Zetterstedt, 1838, *Tryphon*)

***pictus*** (Gravenhorst, 1829, *Tryphon*) E S W I M

*marginatus* (Thomson, 1833, *Cteniscus*)

*crassiceps* (Szépligeti, 1901, *Diaborus*)

***romani*** (Kerrich, 1952, *Cteniscus*) I

***rufilabris*** (Holmgren, 1857, *Exenterus*) E I Kerrich (1962)

*genalis* (Thomson, 1883, *Cteniscus*)

***rufonotatus*** (Holmgren, 1857, *Exenterus*) S I

*breviventris* (Thomson, 1883, *Cteniscus*)

*fulvipes* (Kriechbaumer, 1896, *Exenterus*)

***similis*** (Holmgren, 1857, *Exenterus*) E added by Ely (2010)

***taigensis*** Kasparyan, 1985 S added by Shaw & Kasparyan (2005)

***ERROMENUS*** Holmgren, 1857

*ANIAROPHRON* Förster, 1869

*TRICHOCALYMMA* Förster, 1869

*TRICHOCALYMMUS* Thomson, 1887

***analis*** Brischke, 1871 E S

***bibulus*** Kasparyan, 1973 E S W

***brunnicans*** (Gravenhorst, 1829, *Tryphon*) E S

*brunicans* Dalla Torre, 1901 preocc.

***calcator*** (Müller, 1776, *Ichneumon*) E S

*erythropus* (Gmelin, 1790, *Ichneumon*)

*carinatus* (Holmgren, 1857, *Polyblastus*)

*oelandicus* (Holmgren, 1857, *Polyblastus*)

*scutellaris* (Holmgren, 1857, *Polyblastus*)

***junior*** (Thunberg, 1824, *Ichneumon*) E S

*frenator* (Gravenhorst, 1829, *Exochus*)

*arenicola* Thomson, 1883

***plebejus*** (Woldstedt, 1878, *Trichocalymma*) E

*bipunctatus* (Woldstedt, 1878, *Trichocalymma*)

*brevitarsis* Thomson, 1883

***punctatus*** (Woldstedt, 1878, *Trichocalymma*) S W

added by Shaw & Kasparyan (2005)

*simplex* Thomson, 1883

*defectivus* Strobl, 1903 preocc.

***punctulatus*** Holmgren, 1857 E S I

*niger* (Szépligeti, 1901, *Aniarophron*)

***zonarius*** (Gravenhorst, 1820, *Ichneumon*) E S W

*obscuratus* Habermehl, 1925

***EXCAVARUS*** Davis, 1897

***apiarius*** (Gravenhorst, 1829, *Tryphon*) E W

*obscuratorius* (Panzer, 1809, *Ichneumon*) preocc.

***EXENTERUS*** Hartig, 1837

*ACTENONYX* Förster, 1869

*PICROSCOPUS* Förster, 1869

***abruptorius*** (Thunberg, 1824, *Ichneumon*) E S

*cingulatorius* Holmgren, 1857

***adspersus*** Hartig, 1838 E

*lepidus* Holmgren, 1857

*laricinus* Thomson, 1888

***amictorius*** (Panzer, 1801, *Ichneumon*) E

*marginatorius* (Fabricius, 1793, *Ichneumon*) preocc.

*sulcatorius* (Thunberg, 1824, *Ichneumon*)

*claripennis* Thomson, 1883

[***confusus*** Kerrich, 1952[[494]](#footnote-495)]

***ictericus*** (Gravenhorst, 1829, *Tryphon*) S added by Shaw & Kasparyan (2005)

***oriolus*** Hartig, 1838 E

*flavellus* Thomson, 1883

*brunnescens* Fahringer, 1941

[***tricolor*** Roman, 1913[[495]](#footnote-496)]

[***vellicatus*** Cushman, 1940[[496]](#footnote-497)]

doubtfully placed species of *Exenterus*

[***anceps*** (Stephens, 1835, *Tryphon*) [[497]](#footnote-498) E nom. dub.]

***EXYSTON*** Schiødte, 1839

*ANECPHYSIS* Förster, 1869

*DIABORUS* Förster, 1869

*TRICAMPTUS* Förster, 1869

*PAREXYSTON* Kerrich, 1952

***calcaratus*** Thomson, 1883 E I

***pratorum*** (Woldstedt, 1874, *Exenterus*) E S I

*sedulus* (Woldstedt, 1878, *Cteniscus*)

*brevipetiolatus* Thomson, 1883

*melanurus* Ulbricht, 1926 unavailable

***sponsorius*** (Fabricius, 1781, *Ichneumon*) E S W I

*cinctulus* (Gravenhorst, 1820, *Ichneumon*)

*conopsator* (Thunberg, 1824, *Ichneumon*)

*carinatus* Thomson, 1883

***subnitidus*** (Gravenhorst, 1829, *Tryphon*) E I

*phaeorrhaeus* (Haliday, 1839, *Tryphon*)

***GRYPOCENTRUS*** Ruthe, 1855

*APIMELES* Förster, 1869

***albipes*** Ruthe, 1855 E S

***apicalis*** Thomson, 1883

***basalis*** Ruthe, 1855 E S

***bilobus*** Kasparyan, 1976 S added by Shaw & Kasparyan (2005)

***cinctellus*** Ruthe, 1855 E

***incisulus*** Ruthe, 1855

*erythrurus* Ulbricht, 1926

***KRISTOTOMUS*** Mason, 1962

***laetus*** (Gravenhorst, 1829, *Mesoleptus*) E I

*cephalotes* (Gravenhorst, 1829, *Tryphon*)

*orbitatorius* (Schiødte, 1839, *Exenterus*)

*calcaratus* (Thomson, 1883, *Delotomus*)

*marginatus* (Thomson, 1883, *Delotomus*)

*dioszeghyi* (Kiss, 1924, *Cteniscus*) Horstmann (2009*b*)

***laticeps*** (Gravenhorst, 1829, *Tryphon*) E

***pumilio*** (Holmgren, 1857, *Exenterus*) E

***ridibundus*** (Gravenhorst, 1829, *Tryphon*) E

***triangulatorius*** (Gravenhorst, 1829, *Tryphon*) E S I

*mesoleptoides* (Stephens, 1835, *Tryphon*)

*coarctatus* (Holmgren, 1857, *Acrotomus*)

***MONOBLASTUS*** Hartig, 1837

*COELOCONUS* Förster, 1869

*XIPHURUS* Kriechbaumer, 1896 preocc. Horstmann (2002*c*)

*IDOTHRICHUS* Schmiedeknecht, 1907

*PSEUDOPSILOSAGE* Gregor, 1929

***brachyacanthus*** (Gmelin, 1790, *Ichneumon*) E S I

*testaceus* (Gmelin, 1790, *Ichneumon*)

*oraniensis* (Schmiedeknecht, 1912, *Psilosage*)

***caudatus*** (Hartig, 1837, *Tryphon*) E NHM, det. Broad, added here

*lateralis* (Giraud, 1872, *Tryphon*) preocc.

*sericeus* (Brischke, 1892, *Phaestus*)

*lateralis* Kriechbaumer, 1896 preocc.

***marginellus*** (Gravenhorst, 1829, *Lissonota*) E

*compunctor* misident.

***NELEGES*** Förster, 1869

*ANELPISTUS* Brauns, 1898 preocc.

*BRAUNSIANUS* Berg, 1898

***proditor*** (Gravenhorst, 1829, *Tryphon*) E

*bidentatus* (Brauns, 1898, *Anelpistus*)

*bidentatus* (Strobl, 1903, *Tryphon*) preocc.

*bimucronatus* (Strobl, 1903, *Erromenus*)

***ORTHOMISCUS*** Mason, 1955

***unicinctus*** (Holmgren, 1857, *Exenterus*) E

*macrocephalus* (Holmgren, 1857, *Exenterus*)

***OTOBLASTUS*** Förster, 1869

***luteomarginatus*** (Gravenhorst, 1829, *Tryphon*) E

***POLYBLASTUS*** Hartig, 1837

subgenus ***COPHENCUS*** Townes & Townes, 1949

***macrocentrus*** Thomson, 1888 E S W

subgenus ***LABROCTONUS*** Förster, 1869

*NEMIOBLASTUS* Thomson, 1883

***alternans*** Schiødte, 1838[[498]](#footnote-499)

*albicoxa* Thomson, 1883

***melanostigmus*** Holmgren, 1857 E S M

*grammicus* Holmgren, 1857

*lucidus* Brischke, 1892

***nanus*** Kasparyan, 1973 E added by Shaw & Kasparyan (2005)

***pallicoxa*** Thomson, 1888 E I M

*pallidicoxa* Dalla Torre, 1901

***stenocentrus*** Holmgren, 1857[[499]](#footnote-500) E S M

*albicoxa* Thomson, 1883

***westringi*** Holmgren, 1857 E S

subgenus ***POLYBLASTUS*** Hartig, 1837

***cancer*** (Hartig, 1837, *Tryphon*) E S M

*palaemon* Schiødte, 1838

*holosericeus* (Ratzeburg, 1848, *Tryphon*)

*pyramidatus* Holmgren, 1857

***cothurnatus*** (Gravenhorst, 1829, *Tryphon*) E S

*drewseni* Schiødte, 1838

*unicinctus* Bridgman, 1889

*intermedius* Ulbricht, 1916 unavailable

*hungaricus* (Kiss, 1926, *Trematopygus*)

***pedalis*** (Cresson, 1864, *Tryphon*)[[500]](#footnote-501) E S

*carbonarius* misident.

*rhenanus* Ulbricht, 1926 unavailable

*rufifemur* Hedwig, 1943 unavailable

***pinguis*** (Gravenhorst, 1920, *Ichneumon*)

*petryi* Schmiedeknecht, 1912

***subalpinus*** Holmgren, 1857 S

***tener*** Habermehl, 1909 E S W

***tuberculatus*** Teunissen, 1953 E added by Shaw & Kasparyan (2005)

***varitarsus*** (Gravenhorst, 1829, *Tryphon*) E S W I M

*strobilator* misident.

*albovinctus* (Gravenhorst, 1829, *Tryphon*)

*affinis* Woldstedt, 1874

*subtilis* Thomson, 1883

*variitarsus* Dalla Torre, 1901 preocc.

*rufus* Kiss, 1926

***wahlbergi*** Holmgren, 1857 E S I

*wesmaeli* Holmgren, 1857

doubtfully placed species of *Polyblastus*

[***bridgmani*** Parfitt, 1882 E nom. dub. Fitton (1976)]

[***parvulus*** (Gravenhorst, 1829, *Tryphon*) nom. dub.]

***SMICROPLECTRUS*** Thomson, 1883

*MICROPLECTRON* Förster, 1869 preocc.

***bohemani*** (Holmgren,1857, *Exenterus*) E

***erosus*** (Holmgren,1857, *Exenterus*) E

***excisus*** Kerrich, 1952 E Kerrich (1962)

***heinrichi*** Kerrich, 1952 S I

***jucundus*** (Holmgren, 1857, *Exenterus*) E

***nigricornis*** Kasparyan, 1976 E S added by Shaw & Kasparyan (2005)

***perkinsorum*** Kerrich, 1952 E

***quinquecinctus*** (Gravenhorst, 1820, *Ichneumon*) E S

*trianguligena* Kerrich, 1952

***TRYPHON*** Fallén, 1813[[501]](#footnote-502)

subgenus ***STENOCROTAPHON*** Kasparyan, 1969

***obtusator*** (Thunberg, 1824, *Ichneumon*) E

?*subrufus* (Gmelin, 1790, *Ichneumon*)

*consobrinus* Holmgren, 1857

***subsulcatus*** Holmgren, 1857 E

subgenus ***SYMBOETHUS*** Förster, 1869

***bidentatus*** Stephens, 1835[[502]](#footnote-503) E S W

*incestus* Holmgren, 1857

*incertus* Brischke, 1871

*tricolor* Rudow, 1910 preocc.

*abnormis* Habermehl, 1925

*rufescens* (Kiss, 1926, *Erromenus*)

***brunniventris*** Gravenhorst, 1829 E S

***duplicatus*** (Heinrich, 1953, *Symboethus*)[[503]](#footnote-504) E S

*discedens* (Heinrich, 1953, *Symboethus*)

***exclamationis*** Gravenhorst, 1829 E

*connectens* Roman, 1909

***fulviventris*** Holmgren, 1857 E S added by Kasparyan (1973)[[504]](#footnote-505)

***heliophilus*** Gravenhorst, 1829 E

*bicornutus* Holmgren, 1856

*confinis* Holmgren, 1856

*maculatus* (Pfankuch, 1924, *Symboethus*) unavailable

subgenus ***TRYPHON*** Fallén, 1813

*OTITOCHILUS* Förster, 1869

*PSILOSAGE* Förster, 1869

***abditus*** Kasparyan, 1969 E S

*pleuralis* Thomson, 1883 preocc.

***atriceps*** Stephens, 1835 E W

*ephippium* Holmgren, 1857

***bidentulus*** Thomson, 1883 E

*separandus* Schmiedeknecht, 1912

***latrator*** (Fabricius, 1781, *Ichneumon*) E S W I M

*auricularis* Thomson, 1883 Horstmann (2001*b*)

***nigripes*** Holmgren, 1857 E

***relator*** (Thunberg, 1824, *Ichneumon*) E S

*vulgaris* Holmgren, 1857

*erythrogaster* Thomson, 1883

***rutilator*** (Linnaeus, 1761, *Ichneumon*) E S W

*impraegnator* (Schrank, 1781, *Ichneumon*)

*cepae* (Geoffroy, 1785, *Ichneumon*)

*anodon* (Schrank, 1802, *Ichneumon*)

*ceparum* (Schrank, 1802, *Ichneumon*)

*insultator* (Gravenhorst, 1807, *Ichneumon*)

*quadratus* Stephens, 1835

***signator*** Gravenhorst, 1829 E S W M

*facialis* Stephens, 1835

*nigrifacies* Ulbricht, 1926 unavailable

***thomsoni*** Roman, 1939 E S W

***trochanteratus*** Holmgren, 1857 E S W M

doubtfully placed species of *Tryphon*

[***flavilabris*** Stephens, 1835 nom. dub. E Fitton (1976)]

[***thoracicus*** Stephens, 1835 nom. dub. E Fitton (1976)]

[***zonatus*** Stephens, 1835 nom. dub. E Fitton (1976)]

Subfamily XORIDINAE Shuckard, 1840[[505]](#footnote-506)

***ISCHNOCEROS*** Gravenhorst, 1829

*MITROBORIS* Holmgren, 1859

***caligatus*** (Gravenhorst, 1829, *Xylonomus*) E S I

*seticornis* Kriechbaumer, 1879

***rusticus*** (Geoffroy, 1785, *Ichneumon*) E S W I

*striatus* (Brullé, 1846, *Odontomerus*)

*cornutus* (Ratzeburg, 1848, *Xorides*)

*filicornis* Kriechbaumer, 1879

*caper* (Hedwig, 1957, *Eclytus*)

***ODONTOCOLON*** Cushman, 1942

*ODONTOMERUS* Gravenhorst, 1829 preocc.

***dentipes*** (Gmelin, 1790, *Ichneumon*) E S W I

*femoratum* (Olivier, 1811, *Ophion*)

*pinetorum* (Thomson, 1877, *Odontomerus*)

***quercinum*** (Thomson, 1877, *Odontomerus*) E

*liogaster* (Szépligeti, 1914, *Odontomerus*)

*simile* (Habermehl, 1920, *Odontomerus*)

*brunneiventre* (Telenga, 1930, *Odontomerus*)

***XORIDES*** Latreille, 1809[[506]](#footnote-507)

*XYLONOMUS* Gravenhorst, 1829

*GONOPHONUS* Förster, 1869

*MOEROPHORA* Förster, 1869

*RHADINA* Förster, 1869 preocc.

*SICHELIA* Förster, 1869

*STEROTRICHUS* Förster, 1869

*RHADINOPIMPLA* Schulz, 1911

*NEOXYLONOMUS* Clément, 1938

*XYLONOMINUS* Clément, 1938

***brachylabis*** (Kriechbaumer, 1889, *Xylonomus*) E

*brachylabris* misspelling

***csikii*** Clément, 1938 E

***fuligator*** (Thunberg, 1824, *Ichneumon*) E W

*sordator* (Thunberg, 1824, *Ichneumon*)

*pilicornis* (Gravenhorst, 1829, *Xylonomus*)

***gravenhorstii*** (Curtis, 1831, *Xylonomus*) E W I

*securicornis* (Holmgren, 1860, *Xylonomus*)

*glyptus* (Thomson, 1877, *Xylonomus*)

*clavicornis* (Kriechbaumer, 1879, *Xylonomus*)

*distinguendus* (Magretti, 1884, *Xylonomus*)

*hungaricus* (Szépligeti, 1899, *Sichelia*)

*seticornis* (Strobl, 1902, *Xylonomus*)

*rufoscutellatus* (Habermehl, 1918, *Xylonomus*)

*kokujevi* (Meyer, 1922, *Xylonomus*)

*rufus* (Kiss, 1924, *Xylonomus*)

*caucasicus* (Shestakov, 1925, *Xylonomus*)

*romani* Clément, 1938

***irrigator*** (Fabricius, 1793, *Ichneumon*) E

*planus* Šedivý, 1958

***niger*** (Pfeffer, 1913, *Xylonomus*) E

*bicolor* Clément, 1938

***praecatorius*** (Fabricius, 1793, *Ichneumon*) E S

?*articulatus* (Geoffroy, 1785, *Ichneumon*)

?*falsatorius* (Olivier, 1792, *Ichneumon*)

*parvulus* (Gravenhorst, 1829, *Xylonomus*)

*praecatorius* (Marshall, 1872, *Xylonomus*) preocc.

*rufopictus* (Kiss, 1926, *Xylonomus*)

*ruficoxis* (Kiss, 1929, *Xylonomus*) preocc.

*temporalis* (Kiss, 1929, *Xylonomus*)

*holsaticus* Heinrich, 1951

***rufipes*** (Gravenhorst, 1829, *Xylonomus*) E

***rusticus*** (Desvignes, 1856, *Xylonomus*) E

**References**

Achterberg, C. van 1999 The West Palaearctic species of the subfamily Paxylommatinae (Hymenoptera: Ichneumonidae), with special reference to the genus *Hybrizon* Fallén. *Zoologische Mededelingen, Leiden* **73**, 11-26.

Aeschliman, J.-P. 1983 Note sur les Métopiines ouest-palaéarctiques, avec description de deux espèces nouvelles (Hymenoptera, Ichneumonidae). *Annales de la Société Entomologique de France* **19**, 3-6.

Aeschliman, J.-P. 1989 Révision des espèces ouest-paléarctiques du genre *Hypsicera* Latreille (Hymenoptera: Ichneumonidae). *Annales de la Société Entomologique de France* **25**, 33-39.

Allen, A. A. 2007 A record of *Afrephialtes cicatricosus* (Ratzeburg) (Hymenoptera: Ichneumonidae) from South Devon. *British Journal of Entomology and Natural History* **20**, 45.

Anderson, A., Broad, G. & Baur, H. 2006 New Irish records of Ichneumonidae, Braconidae and Pteromalidae (Hymenoptera) collected from agricultural grasslands. *Irish Naturalists Journal* **28**, 246-248.

anon. 2001 14 December 2000. *British Journal of Entomology and Natural History* **14**, 125.

Ashmole, N. P., Nelson, J. M. Shaw, M. R. & Garside, A.. 1983 Insects and spiders on snowfields in the Cairngorms, Scotland. *Journal of Natural History* **17**, 599-613.

Askew, R. R. 2000 An aggregation of Parasitic Hymenoptera. *Entomologist's Monthly Magazine* **136**, 147.

Aubert, J.F. 1978 *Les ichneumonides ouest-palearctiques et leurs hôtes 2. Banchinae et Suppl. aux Pimplinae*. Laboratoire d'Evolution des Etres Organises, Paris & EDIFAT-OPIDA, Echauffour.

Aubert, J. F. 1985 Ichneumonides Scolobatinae des collections suédoises (suite) et du Musée de Léningrad. *Bulletin de la Société Entomologique de Mulhouse* **1985**, 49-58.

Aubert, J.F. 1988 Troisème prélude à une révision des Ichneumonides Scolobatinae: les *Rhorus* Foerst., du groupe de *neustriae* Schrk. *Bulletin de la Société Entomologique de Mulhouse* **1988**, 1-10.

Aubert, J. F. 1994 Supplément aux Ichneumonides *Rhorus* Foerst. (1). *Bulletin de la Société Entomologique de Mulhouse* **1994**, 61-64.

Aubert, J. F. 2000 Les ichneumonides ouest-paléartiques et leurs hôtes. 3 Scolobatinae (= Ctenopelmatinae) et supplement aux volumes précédents. *Littererae Zoologicae, Actes du Musée cantonal de Zoologie, Lausanne* **5**, 1-310.

Azidah, A. A., Fitton, M. G. & Quicke, D. L. J. 2000 Identification of the *Diadegma* species (Hymenoptera: Ichneumonidae, Campopleginae) attacking the diamondback moth, *Plutella xylostella* (Lepidoptera : Plutellidae). *Bulletin of Entomological Research* **90**, 375-389.

Barari H., Ferguson, A.W., Piper, R.W., Smith, E., Quicke, D.L.J. & Williams, I.H. 2005 The separation of two hymenopteran parasitoids, *Tersilochus obscurator* and *Tersilochus microgaster* (Ichneumonidae), of stem-mining pests of winter oilseed rape using DNA, morphometric and ecological data. *Bulletin of Entomological Research* **95**, 299-307.

Bass, J. A. B. & Cooling, D. A. 1983 As association between a muscid, an ichneumonid and a simuliid recorded from a reservoir outfall in southern England. *Entomologist's Gazette* **34**, 125-127.

Bauer, R. 2001 Bemerkungen über die Ichneumoniden der Alpen mit einigen Neubeschreibungen Teil III (Hymenoptera, Ichenumonidae, Ichneumoninae). *Entomofauna* **22**, 245-269.

Bennett, A. M. R. 2001 Phylogeny of the Agriotypinae (Hymenoptera: Ichneumonidae), with comments on the subfamily relationships of the basal Ichneumonidae. *Systematic Entomology* **26**, 329-356.

Bennett, A. M. R. 2015 Revision of the world genera of Tryphoninae (Hymenoptera: Ichneumonidae). *Memoirs of the American Entomological Institute* **86**, viii+1-387.

Bennett, F. D., Askew, R. R. & Shaw, M. R. 2002 A second rearing of *Telepsogina adelognathi* Hedqvist, 1958 (Hym., Pteromalidae, Miscogastrinae). *Entomologist's Monthly Magazine* **138**, 59-61.

Billany, D. J., Carter, C. I., Winter, T. G. & Fielding, N. J. 1983 The effects of climate and parasites on *Gilpinia hercyniae* (Hartig) (Hymenoptera: Diprionidae) in Britain. *Entomologist's Monthly Magazine* **119**.

Bordera, S., González-Moreno, A., Khalaim, A. I. & Sääksjärvi, I. E. 2014 Revision of North American species of *Clistopyga* (Hymenoptera: Ichneumonidae: Pimplinae). *Canadian Entomologist* **146**: 355-414.

Boston, M. R. 1986 First Irish record of *Neorhacodes enslini* (Ruschka) (Hymenoptera: Ichneumonidae). *Irish Naturalists' Journal* **22**, 116.

Boston, M. R. and R. Nash. 1989 Twenty-one species of Hymenoptera (Ichneumonidae: Ichneumoninae) new to Ireland, with a list of ichneumonine species in the Ulster Museum, Belfast. *Irish Naturalists' Journal* **23**, 134-137.

Broad, G. R. 2004 Generic synonymies affecting the Orthocentrinae (Hym., Ichneumonidae), with notes on the composition of the subfamily. *Entomologist's Monthly Magazine* **140**, 297-299.

Broad, G. R. 2010 Status of *Batakomacrus* Kolarov (Hymenoptera: Ichneumonidae: Orthocentrinae), with new generic combinations and description of a new species. *Zootaxa* **2394**, 51-68.

Broad, G. R. & Davis, A. M. 2015 *Lymantrichneumon disparis* (Poda, 1761) (Hymenoptera: Ichneumonidae), a genus and species new to Britain. *Entomologist’s Gazette* **66**, 216-218.

Broad, G. R. & Shaw, M. R. 2005 The species of four genera of Metopiinae (Hymenoptera: Ichneumonidae) in Britain, with new host records and descriptions of four new species. *Journal of Natural History* **39**, 2389-2407.

Broad, G. R. & Shaw, M. R. 2016 The British species of *Enicospilus* (Hymenoptera: Ichneumonidae: Ophioninae). *European Journal of Taxonomy*, **187**, 1-31.

Brock, J. P. 1982 A systematic study of the genus *Ophion* in Britain (Hymenoptera, Ichneumonidae). *Tijdschrift voor Entomologie* **125**, 57-97.

Brock, J. P. & Shaw, M. R. 1997 *Perithous albicinctus* (Gravenhorst), a large pimpline ichneumon-wasp new to Britain (Hymenoptera: Ichneumonidae). *Entomologist's Gazette* **48**, 49-50.

Burks, R. A. 2012 Formation of family group names using the stem of -gaster, with special reference to names based on *Miscogaster* and *Sphegigaster* (Hymenoptera: Chalcidoidea: Pteromalidae). *Zootaxa* **3389**, 61-64.

Carlson, R. W. 1979 Family Ichneumonidae. Stephanidae. In *Catalog of Hymenoptera in America north of Mexico*. (ed. K. V. Krombein, P. D. Hurd Jr., D. R. Smith & B. D. Burks), pp. 315-741. Smithsonian Institution Press, Washington.

Carr, L. A. 1924 The Ichneumonidae of the Lichfield district, Staffordshire. *Transactions of the North Staffordshire Field Club* **58 (Appendix)**, 1-70.

Cowin, W. S. 1953 Entomological Notes. *The Peregrine* **2**, 22-23.

Cowin, W. S. & Williamson, K. 1940 Manx entomological notes. *The Entomologist* **73**, 67-68.

Cox, M. L. 2007 *Atlas of the Seed and Leaf Beetles of Britain and Ireland. Pisces Publications, Newbury.*

Dasch, C. 1992 The ichneumon-flies of America north of Mexico. Pt. 12. Subfamilies Microleptinae, Helictinae, Cylloceriinae and Oxytorinae (Hymenoptera: Ichneumonidae). *Memoirs of the American Entomological Institute* **52**, 1-470.

Delrio, G. 1975 Révision des espèces ouest-paléarctiques du genre *Netelia* Gray (Hym., Ichneumonidae). *Studi Sassaresi sez. III.- Annali della Facolta di Agraria dell'Università di Sassari* **23**, 1-126.

Diller, E. 1981 Bemerkungen zur Systematik der Phaeogenini mit einem vorläufigen Katalog der Gattungen (Hymenoptera, Ichneumonidae). *Entomofauna* **2**, 93-111.

Diller, E. 1982 Untersuchungen über Arten der Gattungen *Diplazon* Viereck, 1914, und *Sussaba* Cameron, 1909 (Hymenoptera, Ichneumonidae, Diplazontinae). *Entomofauna* **3**, 65-80.

Diller, E. & Schönitzer, K. 2003 Revision einiger westpaläarktischer und nearktischer Arten der Gattung *Colpognathus* Wesmael, [1845] (Hymenoptera, Ichneumonidae, Ichneumoninae, Alomyini). *Entomofauna* **24**, 333-344.

Diller, E. & Shaw, M. R. 2014 Western Palaearctic Oedicephalini and Phaeogenini (Hymenoptera: Ichneumonidae, Ichneumoninae) in the National Museums of Scotland, with distributional data including 28 species new to Britain, rearing records, and descriptions of two new species of *Aethecerus* Wesmael and one of *Diadromus* Wesmael. *Entomologist's Gazette* **65**: 109-129.

Diller, E. & Tereshkin, A. M. 2005 Neue Erkenntnisse zur Gattung *Herpestomus* Wesmael, [1845] mit Beschreibung neuer Arten (Hymenoptera, Ichneumonidae, Ichneumoninae, Phaeogenini). *Entomofauna* **26**, 305-312.

Donisthorpe, H. S. J. K. 1927 *The Guests of British Ants: their habits and life-histories*. London: George Routledge and Sons Ltd.

Edgar, W. D. 1971 Aspects of the ecology and energetics of the egg sac parasites of the wolf spider *Pardosa lugubris* (Walckenaer). *Oecologia* **7**, 155-163.

Ely, W. A. 2001 First UK record of *Adelognathus stelfoxi* Fitton, Gauld & Shaw (Hym.: Ichneumonidae). *The Entomologist's Record and Journal of Variation* **113**, 140.

Ely, W. A. 2002 *Tycherus nigridens* (Wesmael, 1845) (Hym.: Ichneumonidae) new to Britain. *The Entomologist's Record and Journal of Variation* **114**, 109.

Ely, W. A. 2010 *Eridolius similis* (Holmgren) (Hymenoptera, Ichneumonidae, Tryphoninae) at Blacktoft Sands NR. *Yorkshire Naturalists' Union Bulletin* **54**, 33.

Field, J. P. & Foster, W. A. 1988 The bees and wasps of Scolt Head Island national nature reserve, Norfolk. *British Journal of Entomology and Natural History* **1**, 79-83.

Fitton, M. G. 1975 A review of the British species of *Tryphon* Fallén (Hym.,Ichneumonidae). *Entomologist's Monthly Magazine* **110**, 153-171.

Fitton, M. G. 1976 The western palaearctic Ichneumonidae (Hymenoptera) of British authors. *Bulletin of the British Museum (Natural History), Entomology* **32**, 301-373.

Fitton, M. G. 1981 The British Acaenitinae (Hymenoptera: Ichneumonidae). *Entomologist’s Gazette* **32**, 185-192.

Fitton, M. G. 1984 A review of the British Collyriinae, Eucerotinae, Stilbopinae and Neorhacodinae (Hymenoptera: Ichneumonidae). *Entomologist’s Gazette* **35**, 185-195.

Fitton, M. G. 1985 The British species of *Cidaphus* (Hymenoptera: Ichneumonidae). *Entomologist’s Gazette* **36**, 293-297.

Fitton, M. G. 1987 A review of the *Banchus*-group of ichneumon-flies, with a revision of the Australian genus *Philogalleria* (Hymenoptera: Ichneumonidae). *Systematic Entomology* **12**, 33-45.

Fitton, M. G. & Boston, M. 1988 The British species of *Phthorima* (Hymenoptera: Ichneumonidae). *Entomologist's Gazette* **39**, 165-170.

Fitton, M. G. & Ficken, L. 1990 British ichneumon-flies of the tribe Oedemopsini (Hymenoptera: Ichneumonidae). *The Entomologist* **109**, 200-214.

Fitton, M. G. & Gauld, I. D. 1980 A review of the British Cremastinae (Hymenoptera: Ichneumonidae), with keys to the species. *Entomologist’s Gazette* **31**, 63-71.

Fitton, M. G. & Rotheray, G. E. 1982 A key to the European genera of diplazontine ichneumon-flies, with notes on the British fauna. *Systematic Entomology* **7**, 311-320.

Fitton, M. G., Gauld, I. D. & Shaw, M. R. 1982 The taxonomy and biology of the British Adelognathinae (Hymenoptera: Ichneumonidae). *Journal of Natural History* **16**, 275-283.

Fitton, M. G., Shaw, M. R. & Gauld, I. D. 1988 Pimpline Ichneumon-flies. Hymenoptera, Ichneumonidae (Pimplinae). *Handbooks for the Identification of British Insects* **7(i)**.
[truncated: 138,384 more chars]
